# Assigning Significance in Label-Free Quantitative Proteomics to Include Single-Peptide-Hit Proteins with Low Replicates

**DOI:** 10.1155/2010/731582

**Published:** 2010-07-25

**Authors:** Qingbo Li

**Affiliations:** ^1^Center for Pharmaceutical Biotechnology, College of Pharmacy, University of Illinois at Chicago, Chicago, IL 60607, USA; ^2^Department of Microbiology and Immunology, College of Medicine, University of Illinois at Chicago, Chicago, IL 60612, USA

## Abstract

When sample replicates are limited in a label-free proteomics experiment, selecting differentially regulated proteins with an assignment of statistical significance remains difficult for proteins with a single-peptide hit or a small fold-change. This paper aims to address this issue. An important component of the approach employed here is to utilize the rule of Minimum number of Permuted Significant Pairings (MPSP) to reduce false positives. The MPSP rule generates permuted sample pairings from limited analytical replicates and simply requires that a differentially regulated protein can be selected only when it is found significant in designated number of permuted sample pairings. Both a power law global error model with a signal-to-noise ratio statistic (PLGEM-STN) and a constant fold-change threshold were initially used to select differentially regulated proteins. But both methods were found not stringent enough to control the false discovery rate to 5% in this study. On the other hand, the combination of the MPSP rule with either of these two methods significantly reduces false positives with little effect on the sensitivity to select differentially regulated proteins including those with a single-peptide hit or with a <2-fold change.

## 1. Introduction

The increasing use of liquid chromatography/mass spectrometry (LC/MS) instrumentation for proteomics studies at a large scale stimulates the development and improvement of data analysis tools. The precise retrieval of biological information from a large LC/MS dataset critically depends on algorithms for data interpretation, which remains a current bottleneck in the rapid advance of proteomics technology [1]. The quantitation of differentially regulated proteins represents a major type of proteomics application in biological studies. Protein quantitation with LC/MS data includes three conceptually different methods, that is, spectral counting, differential stable isotope labeling, and label-free LC/MS measurements by using extracted ion chromatographic intensities [2]. Due to the increased time and complexity of sample preparation in stable isotope labeling, cost of labeling reagents and requirement of higher starting sample amount, however, researchers are increasingly using label-free proteomics for faster and simpler protein quantitation [3]. 

Multiple algorithms and software solutions for label-free proteomics data analysis have been developed [2]. These algorithms and software solutions provide quantitation of protein differential abundances but do not always provide a statistical significance assessment of differential abundances. Algorithms for statistical significance analysis in label-free proteomics with spectral counting were investigated [4, 5]. In label-free quantitation with extracted ion chromatographic intensities, there are still needs to improve approaches for assessing statistical significance, especially for low-replicate datasets [6]. 

Most proteomics studies infer proteins with ≥2 identified peptides as reliable protein identifications and usually disregard proteins with a single-peptide hit as unreliable for quantitation. This “two-peptide” rule was recently challenged with the evidence that it reduced protein identifications more in a target database than in a decoy database, and thus increased false discovery rates in protein identification [7]. Indeed, it was shown that proteins with a single-peptide hit could represent 30% of the proteins identified with ≥2 MS2 spectrum matches at *P* < .01 [6]. Because those single-peptide proteins had ≥2 MS2 spectrum matches (*P* < .01) in multiple LC/MS analyses under the same condition, they had an adequate level of statistical confidence to be included for quantitation. 

But the inclusion of single-peptide proteins in a differential quantitative proteomics analysis raises two issues. The first is that a conventional statistical test such as a *t*-test can not be applied toward these single-peptide proteins when the *t*-test relies on multiple quantified peptides as replicates to calculate the *t*-statistic for the protein relative abundance [6]. The second is that many single-peptide proteins are at a lower abundance and thus noisy. More stringent thresholds are needed to control the false discovery rate when these single-peptide proteins are included for the selection of differentially regulated proteins. 

Pavelka et al. applied a power law global error model (PLGEM) and the signal-to-noise ratio (STN) statistic [8] to select differentially regulated proteins based on a spectral counting quantitation method [4]. The PLGEM-STN statistic utilized a resampling approach to estimate the null distribution from replicates of a sample. After the error model was calculated from a pool of resampling statistics that constituted the null distribution, a set of STN thresholds were applied at a specified confidence level toward samples with any level of replicates. The PLGEM-STN method is attractive in that it could be applied toward samples with no replicates if several replicates for one sample are provided to estimate the null distribution. It is also applicable to proteins with any number of identified peptides. The PLGEM-STN method, however, has not been demonstrated for label-free quantitation with extracted ion chromatographic intensities. 

In this paper, the PLGEM-STN statistic was applied toward a LC/MS dataset obtained with a high-resolution mass spectrometer [9]. The peptide and protein abundances were quantified with a label-free approach based on extracted ion chromatographic intensities [6]. The false discovery rate was estimated at different confidence levels of the PLGEM-STN statistics. The PLGEM-STN statistic alone did not provide a desired level of false discovery rate control. Insufficient stringency in false discovery rate control was similar to the situation when a *t*-test statistic was used alone [6]. With the combination of a *t*-test and the rule of Minimum number of Permuted Significant Pairings (MPSP), however, the false discovery rate was significantly reduced in that study [6]. 

In this study, the combination of MPSP and PLGEM-STN was tested for controlling the false discovery rate in order to extend the selection of differentially regulated proteins to those with lower fold-changes and to those with single-peptide hits. The combination of MPSP and fold-change thresholds was also compared with the PLGEM-STN-MPSP approach.

## 2. Materials and Methods

### 2.1. Cell Cultures and Proteins Samples

The *Mycobacterium smegmatis* (Msm) strain mc^2^ 155 was obtained from the American Type Culture Collection (ATCC; Rockville, Md) and cultured in 7H9 media [10]. A pH 5.0 and a pH 7.0 Msm culture were grown in triplicate in unlabeled media and harvested as described previously [6, 9]. A cell pellet was collected from a 30-ml culture aliquot for each culture replicate in a log phase. A [^15^N]-labeled Msm culture was also grown for use as a control to determine false positive rates in protein quantitation [10]. Hereafter, the Stressed pH 5 culture is named as **S**, the Reference pH 7 culture as **R**, and the Control culture as **C**. 

As described previously [10], the medium for growing ^15^N labeled cells consisted of (g/L) 99At% (^15^NH_4_)_2_SO_4_: 0.5; glucose: 2; Tween 80: 0.5; citric acid: 0.094; biotin: 0.0005; pyridoxine: 0.001; NaCl: 0.1; Na_2_HPO_4_: 2.5; KH_2_PO_4_: 1; MgSO_4_ · 6H_2_O: 0.1; CuSO_4_ · 5H_2_O: 0.001; ZnSO_4_ · 6H_2_O: 0.002; CaCl_2_ · 2H_2_O: 0.0007; ferric ammonium citrate: 0.04; pH 5.0. The single ^15^N labeled cell culture was grown at 50 ml in a loosely capped 250-ml nephelo culture flask under shaking at 37°C. Thirty milliliter of the ^15^N labeled reference culture was collected at OD 1.1 in the late-log phase.

### 2.2. Protein Sample Preparation

Preparation of proteins from the cell pellets of cultures S, R, and C was described previously [6, 10]. The S triplicates were pooled to generate protein sample S_P_ and the R triplicates were pooled to generate protein sample R_P_ [6]. In addition, the S triplicates S_A_, S_B_, and S_C_ were also individually processed. These five protein samples; that is, S_P_, R_P_, S_A_, S_B_, and S_C_ were, respectively, mixed with an equal amount of proteins from the [^15^N]-labeled C culture. After mixing with the labeled proteins from culture C, the five protein samples were separated on a 1D-SDS/PAGE gel, divided into five fractions, and processed for in-gel digestion and peptide extraction for LC/MS analysis as described in [9, 10]. For the pooled samples S_P_ and R_P_, all five fractions were analyzed by LC/MS. For S_A_, S_B_, and S_C_, only the center fractions were analyzed by LC/MS.

### 2.3. Peptide Analysis

The peptide extract from each gel fraction was constituted in ~25 *μ*l 5% formic acid and was analyzed in duplicate injections with a nanoLC/LTQ-FTMS system (Thermo Finnigan; San Jose, CA) [6]. In each LC/MS injection, 5 *μ*l of peptide extract solution was separated on a 150-mm × 75-*μ*m C18 reverse phase column with 5% to 35% acetonitrile (v/v) gradient in 0.1% trifluoroacetic acid over 60 minutes. The LTQ-FTMS was operated in a data-dependent acquisition mode with up to 10 MS/MS spectra acquired following each MS scan. The acquired RAW data files were imported into BioWorks for peptide and protein identification. The BioWorks (Thermo Finnigan; San Jose, CA) software was on a stand-alone workstation and utilized Sequest as the search engine. The RAW data files were searched against an NCBI Msm database in two separate BioWorks searches. One search corresponded to [^14^N]-labeled peptides and proteins. The other corresponded to [^15^N]-labeled peptides and proteins. The precursor ion tolerance was set at ±1.5 Da to include the peptides, which precursor ions had one ^13^C isotope. Trypsin was designated as the digestion enzyme with two allowed missed cleavages. Peptide and protein probabilities were calculated in BioWorks. Only the peptide charge states (PCSs) with *P* < .01 were accepted for subsequent quantitation. Lists of PCSs selected at *P* < .01 were exported from BioWorks into Excel spreadsheets. The Excel spreadsheets containing the accepted PCSs, along with RAW data files, were processed for quantitation as previously described [10–12]. The abundance of a PCS was represented by the extracted ion chromatographic intensity. The LC/MS raw data associated with this paper can be downloaded from http://proteomecommons.org/ Tranche (see supplementary material available online at doi:10.1155/2010/731582). 

### 2.4. Protein Quantitation

 Protein abundances were quantified with a label-free approach as described in [6, 9]. The abundance of a protein was calculated as the sum of the extracted ion chromatographic intensities of the PCSs detected for that protein [9]. The unlabeled protein samples were named as S_A_, S_B_, S_C_, S_P_, and R_P_. The [^15^N]-labeled protein sample from culture C had five sample preparation replicates because it was mixed with each of the five unlabeled proteins samples. Accordingly, each sample preparation replicate of the culture C protein sample was named by adding the prefix “c” before the unlabeled protein sample with which it was run together. For example, the labeled sample that was mixed with S_P_ was named cS_P_, and so forth. Thus, the labeled C culture protein sample had five replicates that were named as cS_A_, cS_B_, cS_C_, cS_P_, and cR_P_, respectively. Because each sample was analyzed in duplicate LC/MS injections, the LC/MS injections were named by adding the subscript 1 or 2 to each protein sample (see Table 1). 

Therefore, the LC/MS analysis of the five protein samples led to 20 quantitation categories (Table 1). Here, a quantitation category referred to one LC/MS injection of a protein sample in unlabeled or labeled form. Because each protein sample contained the unlabeled proteins from culture S or R, and the labeled proteins from control culture C, one LC/MS injection generated four quantitation categories with two belonging to the unlabeled protein sample and two to the labeled protein sample. The five unlabeled protein samples (S_A_, S_B_, S_C_, S_P_, and R_P_), the five sample preparation replicates of the labeled control protein sample (cS_A_, cS_B_, cS_C_, cS_P_, and cR_P_), and the 20 quantitation categories arising from the duplicate analysis of these samples are summarized in Table 1.

### 2.5. Normalization among Sample Fractions

The complete analysis of the five gel fractions for S_P_ and R_P_ resulted in the quantitation of 5134 PCSs and 1032 proteins (see Tables 1 and 2 in Supplementary Material available online at doi:10.1155/2010/731582). In the label-free quantitation approach employed here, the abundance of a PCS (*A*_PCS_) was represented by the extracted ion chromatographic intensity of the PCS, and the abundance of a protein (*A*_PRO_) was represented by the sum of the extracted ion chromatographic intensities of the PCSs that belonged to the protein. 

Because the sample fractionation efficiency might dictate the approach to normalize the samples, the fractionation resolution was examined by plotting a histogram for the percentage of the detected PCSs versus the number of gel fractions in which they were present (Figure 2). The result shows that 82.8% of the PCSs were present in a single gel fraction and 96.3% were present in ≤2 gel fractions. Thus, a majority of PCSs were detected only in one gel fraction. These PCSs were called single-band PCSs. 

The selection of the single-band PCSs was for the purpose of normalizing PCS abundances in different fractions [13]. In each fraction, the PCS abundances were normalized in the following two steps. In the first normalization step, the PCS abundances were normalized by the median extracted ion chromatographic intensity sought from the single-band PCSs. Then, the median-normalized PCSs intensities were multiplied by the total intensity of the same fraction averaged over all of the samples. 

In these two steps of normalization, the first median-normalization step improves the comparability of PCSs in each fraction across different samples. The second normalization step retained the relative fraction intensity information across the five fractions, so that the *A*_PCS_ values correlated more adequately to their protein abundances in the samples. This two-step normalization approach is depicted in Figure 3 as well. 

It is critical to perform the second step of normalization because it preserves the information about the abundance of a protein in a sample. The information about the abundance of a protein in the samples will be indispensable to perform the power law global error and signal-to-noise statistic modeling as described later. 

After PCS normalization, the protein abundance was calculated by summing the *A*_PCS_ values of that protein in each sample [14, 15].

## 3. Results

The purpose of this study was two-fold. One was to extend the selection of differentially regulated proteins to those that had single-peptide hits. The other was to select differentially regulated proteins at smaller fold-changes and at a false discovery rate ≤.05. The approaches to achieve this two-fold purpose were investigated under a scenario where the number of sample replicates was too small to apply other typical statistics such as a *t*-test. More importantly, a conventional *t*-test alone might not provide the necessary specificity in the label-free quantitation of differentially regulated proteins. Therefore, in a prior test, it was found necessary to insert an additional measure, such as the MPSP rule [6]. 

**Figure 1:**
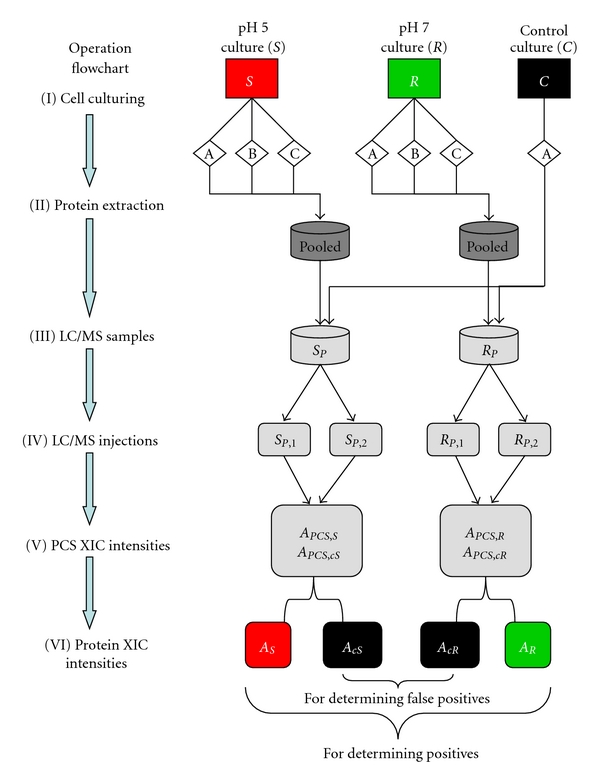
Experimental outline of the label-free protein quantitation approach to assess the acid stress response between the unlabeled stressed culture (S) and the unlabeled reference culture (R) with the [^15^N]-labeled culture as control (C). The procedures are divided into six stages (I–VI). Briefly, equal amounts of protein extract from the S culture triplicates were pooled. Equal amounts of protein extract from the R culture triplicates were also pooled. Into these two pooled unlabeled protein samples, an equal amount of protein extract from the C culture was added. This resulted in the two pooled samples; that is, S_P_ and R_P_. The proteins differentially expressed between the S and R cultures were determined based on comparison of the abundances of the unlabeled proteins that is, *A*_S_ and *A*_R_, between samples S_P_ and R_P_. For the purpose of false discovery rate assessment, the abundances of the [^15^N]-labeled proteins that is, *A*_cS_ and *A*_cR_, were quantified and compared between S_P_ and R_P_ in the same way as between *A*_S_ and *A*_R_. The proteins found differentially expressed between *A*_S_ and *A*_R_ were considered positives, because they reflected the difference between the S and R cultures. The proteins found differentially expressed between *A*_cS_ and *A*_cR_ in the labeled form were false positives, because difference was not expected from the identical C sample that was run concurrently with two unlabeled samples in separate runs.

**Figure 2:**
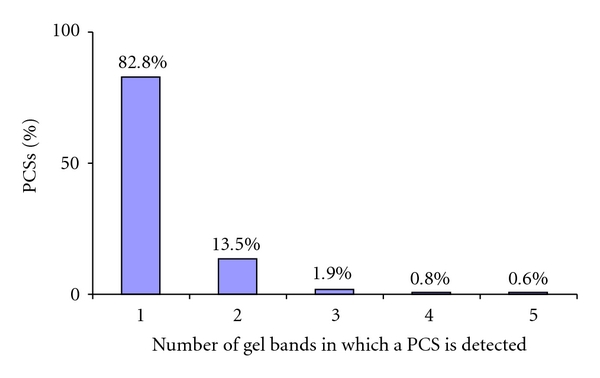
Histogram of the detected 5134 PCSs categorized based on the number of gel fractions in which they were present.

The biological sample model used in the study was the proteome response of an acid stressed Msm culture (S) in reference to a neutral pH culture (R) [9]. Both S and R cultures were unlabeled. The proteins from a [^15^N]-labeled control culture (C) was used as an internal standard to mix with the proteins from the unlabeled cultures (Figure 1). Because the proteins from the control culture were analyzed repeatedly with two other unlabeled samples, the repeated analyses of the labeled control provided replicates to construct a null distribution in which no true differentially regulated proteins were present. The null distribution was used to derive an error model. Such an error model could not be derived from the pair of unlabeled protein samples S_P_ and R_P_ that did not have protein sample replicates. 

With the null distribution provided by the labeled control sample, different approaches were experimented with to select differentially regulated proteins by using the combination of MPSP, PLGEM-STN, and fold-change. Differentially regulated proteins were selected from the unlabeled sample pair S_P_ and R_P_. The other three samples S_A_, S_B_, and S_C_ were used to evaluate the source of variability but not for the selection of differentially regulated proteins. The naming of these samples and their LC/MS runs is delineated in Table 1. 

This Results section consists of the following two subsections.

1. Analyze the source of variability in the peptide and protein quantitation processes. An overview of this subsection is presented in Figure 4.
2. Perform multistep extended selection of differentially regulated proteins. These steps are summarized in Table 2.

### 3.1. The Source of Variability in the Label-Free LC/MS Data

An observed differential abundance of a PCS or protein between samples arose not only from the difference in biological samples but also from measurement noise that included the variability among LC/MS injection replicates, sample preparation replicates, biological replicates, or the data processing method. 

To assist in the assessment of the source of variability in the label-free quantitation of the LC/MS data, the 3rd of the five fractions of an SDS/PAGE gel lane was processed for LC/MS analysis for the protein samples S_A_, S_B_, S_C_, S_P_, and R_P_ with duplicate injections for each sample (Table 1) [6]. The five samples with two LC/MS injections per sample resulted in 10 LC/MS runs. These 10 LC/MS runs of the 3rd fraction allowed the quantitation of 349 proteins for the 3rd fraction [6]. Because a protein was quantified in both the unlabeled form (for culture S or R) and the labeled form (for culture C), there were 20 quantitation categories for each protein. Thus, these 349 proteins and the 20 quantitation categories formed a 349 × 20 matrix. The 349 × 20 matrix was examined by a clustering analysis [16] to obtain an overview of the correlation among the protein samples and LC/MS injections with the purpose to reveal the major source of variability. The naming of the 20 quantitation categories was shown in Table 1. 

From the clustering tree of the 20 quantitation categories shown in Figure 4, it could be seen that the distance between each pair of duplicate LC/MS injections was the shortest compared to those between any other sample pairings. The closest distance of the duplicate LC/MS injections for a sample indicated that the variability between LC/MS injections was the smallest, which also excluded that the label-free data analysis methodology [6] would introduce a significant variability. 

In Figure 4, it was also apparent that the unlabeled and labeled quantitation categories were separated into two distinct branches represented by nodes I and II, respectively. The separation of the unlabeled and labeled quantitation categories into the two distinct clusters indicated that the difference between cultures C and S or C and R was larger than the difference between S and R. From the tree branch under node II, it could be seen that the distance between the unlabeled protein samples S_P_ and R_P_ was larger than the distance among the S culture replicates; that is, S_A_, S_B_, and S_C_. The result indicated that the difference between cultures S and R exceeded the difference among the S culture replicates, suggesting that the variability in biological sample replicates was less than the actual difference between the biological samples treated with different conditions.

Therefore, the clustering result in Figure 4 indicated that the variability increased in the order of LC/MS injections < sample preparation replicates (under node I) ~biological replicates (under node III) < biological samples (between nodes III and IV). Because these differences were evaluated based on the proteomic quantitation data, a variability observed among biological replicates also included the variability introduced during sample preparation for LC/MS analysis. The similarity between the variability observed among the sample preparation replicates and the variability observed among the biological replicates suggested that the variability among biological replicates was not larger than the variability among sample preparation replicates.

### 3.2. Extended Selection of Differentially Regulated Proteins

This subsection describes the multiple steps leading to the extended selection of differentially regulated proteins from all quantified proteins including those with only a single-peptide hit. The proteins with a single-peptide hit represent 1/3 of the identified proteins. Therefore, it is desirable to have a procedure to select regulated proteins from all of the proteins including those with a single-peptide hit to maximize the potential of the global protein expression profiling.

The major steps to establish the criteria for extended selection of differentially regulated proteins are summarized in Table 2, and are described in detail in the following.

#### 3.2.1. The Null Distribution

Based on the evaluation with the clustering analysis (Figure 4), the variability among sample preparation replicates appeared to be comparable with the variability among biological replicates. Samples S_P_ and R_P_ represented the average of triplicate biological replicates for cultures S and R, respectively, because each of them was the pooled sample of three biological replicates. The pooling process further reduced the biological variability between S_P_ and R_P_. Therefore, the [^15^N]-labeled control sample replicates (Table 1) were adequate to represent a null distribution in which there was no differentially regulated protein. 

The null distribution afforded an estimation of measurement noise. The determined measurement noise was then used to estimate the false discovery rate for the selected differentially regulated proteins between samples S_P_ and R_P_. The null distribution provided a reference for setting thresholds to maximize the selection of differentially regulated proteins (positives) while minimizing false positives. In Figure 5, such a null distribution was illustrated with the scatter plot represented by the pink dots.

To investigate the relationship between measurement variability and protein abundance *A*_PRO_, relative standard deviation (rSTD) was plotted against the mean *A*_PRO_ value for each protein in the unlabeled protein samples (blue trace) or the labeled control protein samples (pink trace) (Figure 5). The rSTD-*A*_PRO_ trace in pink reflected the local noise of the null distribution. The local noise of the null distribution was mainly due to the variability that was introduced during sample preparation (Figure 4). The rSTD-*A*_PRO_ trace in pink clearly suggested that the *A*_PRO_ measurement noise had a reciprocal dependence on the *A*_PRO_ amplitude. The rSTD-*A*_PRO_ trace in blue reflected both sample preparation variability and biological sample difference between cultures S and R. Thus, the blue trace had higher rSTD values than the pink trace throughout the *A*_PRO_ range.

#### 3.2.2. Modeling of Local Noise in the Null Distribution

Because of the reciprocal dependence of *A*_PRO_ rSTD on the *A*_PRO_ value, a universal 3-fold-change cutoff missed some positives at higher *A*_PRO_ values where a <3-fold change was already significantly different from the local noise. Missed positives at higher *A*_PRO_ values could be observed in Figure 5 by examining the spread of the two scatter plots in the high *A*_PRO_ ranges. At *A*_PRO_ > 1000, the rSTD was a few times smaller than that at *A*_PRO_ of ~100. From the figure, it could be seen that it was possible to detect a <2-fold change for the proteins with *A*_PRO_ > 1000. To the contrary, at *A*_PRO_ < 10, a 3-fold change threshold was not sufficient to eliminate many false positives. Therefore, a criterion adaptive to the dependence of *A*_PRO_ noise on *A*_PRO_ values would uncover more differentially regulated proteins. This extended selection of differentially regulated proteins could be achieved by penalizing proteins with higher *A*_PRO_ values less than proteins with lower *A*_PRO_ values. Such an adaptive criterion, however, requires a systematic modeling of the noise to establish the thresholds according to local variability.

The issue of the dependence of variability on mean gene expression level was addressed for gene differential expression studies with DNA microarray. For example, Pavelka et al. proposed a power law global error model (PLGEM) [8] in combination with the signal-to-noise-ratio (STN) test statistic [17] for the identification of differentially expressed genes in microarray data. The PLGEM-STN approach estimated the null distribution by a resampling process. The approach could be applied to a varying number of replicates [8]. Pavelka et al. further applied the approach to spectral count-based quantitative proteomics data [4]. The PLGEM-STN statistic, however, has not been demonstrated for label-free proteomics data based on the quantitation of peptide and protein extracted ion chromatographic intensities.

In this study, the PLGEM-STN statistic was experimented with for the selection of differentially regulated proteins quantified with label-free proteomics based on protein extracted ion chromatographic intensities. The PLGEM-STN analysis was performed in four major steps for the dataset shown in Figure 5(see Scheme S1 in Supplementary Material available online at doi:10.1155/2010/731582).There were two reasons for the choice of the PLGEM-STN method. First, the PLGEM-STN method allowed statistical analyses of the proteins quantified with a single PCS because the PLGEM-STN statistic did not rely on multiple PCSs of a protein like a *t*-test [6]. Because single-peptide proteins constituted a third of the quantified proteins (Figure 6), being able to quantify these single-peptide proteins was important to maximize the potential value of the data. Second, the PLGEM-STN method took into account the dependence of *A*_PRO_ noise on *A*_PRO_ levels. A threshold adjustable to the local dependence of *A*_PRO_ noise on *A*_PRO_ levels allowed the selection of differentially regulated proteins with a smaller fold-change threshold at a higher *A*_PRO_ level. Therefore, the PLGEM-STN method potentially could select more differentially regulated proteins by applying a smaller fold-change threshold in the higher *A*_PRO_ range where the variability was smaller. This possibility was tested as shown in the following.

#### 3.2.3. Selection of Differentially Regulated Proteins with PLGEM-STN

Table 3 shows the result of the PLGEM-STN analysis for the unlabeled samples S_P_ and R_P_ and the labeled sample replicates cS_P_ and cR_P_. cS_P_ and cR_P_ were the labeled control samples analyzed concurrently with S_P_ and R_P_, respectively. The differentially regulated proteins found between S_P_ and R_P_ were positives, and those found between cS_P_ and cR_P_ were false positives. Because each protein sample was analyzed with duplicate LC/MS injections, permutation of the four LC/MS injections for a sample pair resulted in four permuted sample pairings [6]. These four permuted sample pairings were numbered as I to IV in Table 3. In each column for a permuted sample pairing in Table 3, the numbers of false positives and positives and the false discovery rate were listed. The false positives were determined as the differentially regulated proteins for the sample pair cS_P_/cR_P_. The positives were determined as the differentially expressed proteins for the sample pair S_P_/R_P_. For the labeled protein sample pair cS_P_/cR_P_, the four permuted sample pairings were cS_P,1_/cR_P,1_, cS_P,1_/cR_P,2_, cS_P,2_/cR_P,1_, and cS_P,2_/cR_P,2_. For the unlabeled sample pair S_P_/R_P_, the four permuted sample pairings were S_P,1_/R_P,1_, S_P,1_/R_P,2_, S_P,2_/R_P,1_, and S_P,2_/R_P,2_. The naming of the LC/MS injections noted in the permuted sample pairings is shown in Table 1.

In Table 3, the positives and false positives were selected with the PLGEM-STN method at the confidence level of 0.01 and 0.002, respectively. The results indicate that the numbers of positives or false positives were not the same among the four permuted sample pairings. To estimate an average false discovery rate, the numbers of positives and false positives were respectively averaged among the four permuted sample pairings. The false discovery rate was then calculated as the ratio of the average number of false positives divided by the average number of positives. The false discovery rate was determined at two different PLGEM-STN confidence levels (Table 3). With a receiver operating characteristic analysis, the PLGEM-STN approach is examined over a broader confidence level range (Figure 7) and will be compared with another approach that is to be described below.

#### 3.2.4. Addition of the MPSP Rule

Initially, the PLGEM approach was carried out by comparing the duplicate LC/MS injections from the two samples R and S without permutation pairings. But the false discovery rate stayed high unless the sensitivity was severely compromised to reduce the false discovery rate. For example, at a confidence level of 0.0001, only 16 differentially regulated proteins were selected at 6% false discovery rate (data not shown). With all of the permutation pairs and a combination of PLGEM and MPSP, 44 differentially regulated proteins were selected at a false discovery rate of 5% (Table 3). Therefore, utilizing all possible permutation pairs with a combination of PLGEM and MPSP results in a higher sensitivity to uncover differentially regulated proteins.

Because of the variable numbers of positives and false positives among the four permuted sample pairings, it was necessary to determine a consensus list of differentially regulated proteins from the four permuted sample pairings. Previously, the rule of MPSP was applied to determine the consensus list of differentially regulated proteins from four permuted sample pairings [6]. The MPSP rule required that only those proteins that were found differentially regulated in a certain number of permuted sample pairings were counted as positives (for S_P_/R_P_) or false positives (for cS_P_/cR_P_). When a sample pair such as S_P_/R_P_ had no sample replicates but had duplicate LC/MS injections, MPSP was found to be optimum at four [6]. Setting MPSP at four meant that a differentially regulated protein had to be found differentially regulated in all of the four permuted sample pairings.

#### 3.2.5. Selection of Differentially Regulated Proteins with the PLGEM-STN-MPSP Approach

The application of the MPSP rule towards the PLGEN-STN results decreased both false positives and positives (Table 3). But the false discovery rate was also decreased relative to that when only the PLGEM-STN statistic was applied. From Table 3, it could be seen that the number of true positives, which was estimated from the difference between the numbers of positives and false positives, remained about the same. Therefore, the combination of the MPSP rule with the PLEGM-STN method reduced the false discovery rate by 2-3 times without compromising the sensitivity. 

As summarized in Figure 7, the receiver operating characteristic analysis clearly shows that the PLGEM-STN-MPSP approach significantly reduces false positives to improve the specificity without significantly affecting the sensitivity. Compared to the use of the PLGEM-STN statistic alone, the combination of PLGEM-STN and MPSP performs better in controlling false discovery rates without compromising the sensitivity to select differentially regulated proteins.

#### 3.2.6. Selection of Differentially Regulated Proteins with a Fold-Change-MPSP Approach

The use of MPSP with fold-change criteria was also examined (Table 4). With fold-change criteria alone, the false discovery rate did not drop below 46% at 2- to 4-fold changes (Table 4) or even at a 5-fold change (See Figure S3 supplementary material available online at doi:10.1155/2010/731582). With the combination of MPSP and the fold-change criteria, the false discovery rate was reduced from 46% to 21% at 2- and 3-fold changes. At a 4-fold change, the false discovery rate was reduced to 4%. Compared to the combination of PLGEM-STN and MPSP, however, the combination of fold-change and MPSP reduced more true positives at the similar false discovery rate of 4%-5%. Therefore, the application of MPSP with the fold-change criteria reduced sensitivity. The reduced sensitivity was due to the increase in the fold-change threshold.

With the 4-fold-change-MPSP and the PLGEM-STN-MPSP approaches, 26 and 44 proteins were respectively selected as differentially regulated at a false discovery rate of 4% or 5% (Tables 3 and 4). Among these 26 and 44 proteins, there were 55 unique proteins(see Table S1 in Supplementary Material available online at doi:10.1155/2010/731582).These 55 unique proteins included all of the 20 high-confidence differentially regulated proteins identified previously with an empirical fold-change and abundance level cutoff approach [9].

#### 3.2.7. Comparison of the PLGEM-STN-MPSP and Fold-Change-MPSP Approaches

Only 15 proteins were common between the two sets of differentially regulated proteins selected with the 4-fold-change-MPSP and the PLGEM-STN-MPSP approaches (Figure 8(a)). The 4-fold-change-MPSP approach selected more single-PCS proteins than the PLGEM-STN-MPSP approach (Figure 8(b)). The PLGEM-STN-MPSP approach selected proteins with a fold-change as low as 1.8-fold (Figure 8(c)). However, these differentially regulated proteins selected with PLGEM-STN-MPSP had a protein abundance higher than most of the differentially regulated proteins selected with the 4-fold-change-MPSP approach (Figure 8(d)). Thus, the two approaches complement each other and could be used simultaneously.

## 4. Discussion

### 4.1. Motivation of the Extensive Label-Free Quantitative Proteomics Analysis

Despite the relative complexity in label-free proteomics data analysis and the demand of more stringently controlled LC/MS experimental conditions, there are strong motivations stemming from biological and experimental perspectives to use the label-free approach, as discussed below.

As shown in Figure 4, the unlabeled and labeled quantitation categories are separated into two distinct clusters. One includes the quantitation categories from the labeled control culture C (under node I). The other includes the quantitation categories from the two unlabeled cultures S and R (under node II). Thus, there was a larger difference between the labeled (C) and either of the two unlabeled samples (S or R) than between the two unlabeled cultures (S and R). The number of differentially regulated proteins between the labeled culture and either of the unlabeled culture was about three times as many as that between the two unlabeled cultures. Compared to the difference between the two unlabeled cultures, the difference between the labeled culture and either of the unlabeled cultures was larger. This larger difference was probably because the labeled culture was cultured in a synthetic minimal medium while the two unlabeled cultures were grown in a commercial 7H9 broth that was richer in ingredients. Another factor was that the acidic growth condition was a relatively mild stress so that not many proteins were differentially regulated. 

The apparent difference in proteome profile for cells cultured in different media is actually a strong motivation for this study. In microbiological works, it is not always convenient to make a [^15^N]-labeled medium with complex ingredients required to cultivate bacteria under more physiologically relevant conditions. Even some of the stable-isotope-labeled media are technically feasible to make, they often bear a costly price tag. For microbiological works, one might not want to be restricted by the type of medium that can be used because of the stable isotope labeling limitation. For example, some mycobacteria are difficult to cultivate on simple synthetic media and prefer complex media. Thus, unlabeled media are always convenient choices if the down-stream proteomic analysis is established to proceed with the quantitation. 

For such reasons, the focus of this study was on the comparison of protein expression profiles between the two unlabeled cultures S and R. The labeled control culture C was used as an internal standard to estimate false discovery rates.

### 4.2. The Use of a [^15^N]-Labeled Internal Standard for Null Distribution Construction in this Study

The label-free quantitation scheme presented in this study incorporated a labeled internal control to provide replicates for noise modeling without a requirement of other unlabeled sample replicates. The inclusion of a labeled internal control facilitates the control of false discovery rates. 

Internal standards are commonly used to improve reliability of quantitative proteomics such as to aid in removing outlier data and to detect fluctuation in instrument performance [18].

Compared to other synthetic peptide internal standards [18, 19], the [^15^N]-labeled control culture C provides more comprehensive peptide internal standards. For most of the peptides, the extracted ion chromatographic intensities can be matched among the three protein samples originated from the two unlabeled (S and R) cultures and the labeled (C) culture. The C protein sample was mixed and run together with either S or R protein sample, so that the reliability of the internal standards was improved.

For constructing the null distribution for the error model in PLGEM-STN, it would be ideal to have the labeled internal standard identical to an unlabeled sample in protein composition. As mentioned above, however, that requirement could restrict the culturing conditions available for biological experiments. Thus, it is acceptable and sometimes necessary to use a labeled protein mixture sample as internal standard, even though the internal standard sample might be somewhat different from the unlabeled samples in protein abundance profiles.

Nevertheless, the null distribution is only utilized to establish the relation between the signal-to-noise ratio and the peptide abundance in the PLGEM-STN method. There is no requirement of direct one-to-one comparison between the labeled and unlabeled version of a protein during this process. Therefore, the difference in proteome composition between the labeled internal standard sample C and the two unlabeled samples S and R is not expected to affect the modeling parameters derived from the null distribution constructed from the labeled C sample. 

One could choose to run multiple replicates of an unlabeled sample and use the replicates to construct the null distribution [4, 6]. That approach would require more LC/MS runs as discussed previously [6].

### 4.3. The Label-Free Data Analyses and Selection of Differentially Regulated Proteins

The LC/MS data used in this work was acquired with a high-resolution mass spectrometer that resolved peptide peaks from a complex sample mixture to allow the determination of the extracted ion chromatographic intensities of peptides and proteins. Repeated LC/MS injections showed the highest reproducibility among several other types of replicates (Figure 4), indicating that the major variability of the label-free quantitation did not lie within the LC/MS separation and the data analysis method. Rather, sample preparation replicates represented a major source of the variability. With a labeled control sample to run concurrently with each of the unlabeled samples, replicates for the labeled control sample were obtained. The replicates of the control sample provided data to model the noise in the label-free quantitation with extracted ion chromatographic intensities (Figure 5). 

**Figure 3:**
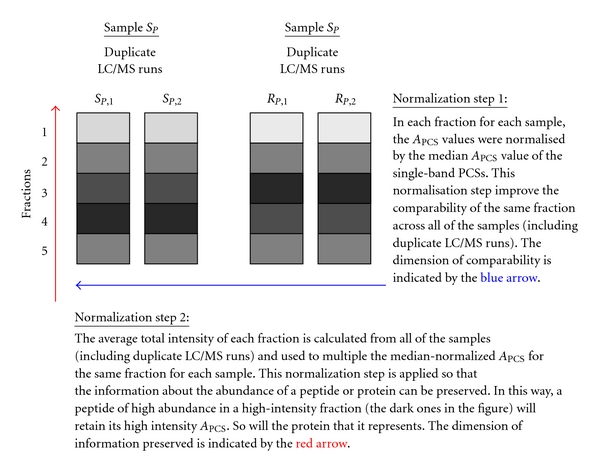
Schematic representation of the two-step normalization process. The gray boxes represent the fractions in the samples. Here, samples include the replicate LC/MS runs of a sample (see Figure 1 and Table 1 for the definitions). The shades indicate the hypothetical total peptide/protein intensities in the fractions. The degrees of darkness are for illustrative purpose and do not represent the actual experimental data.

**Figure 4:**
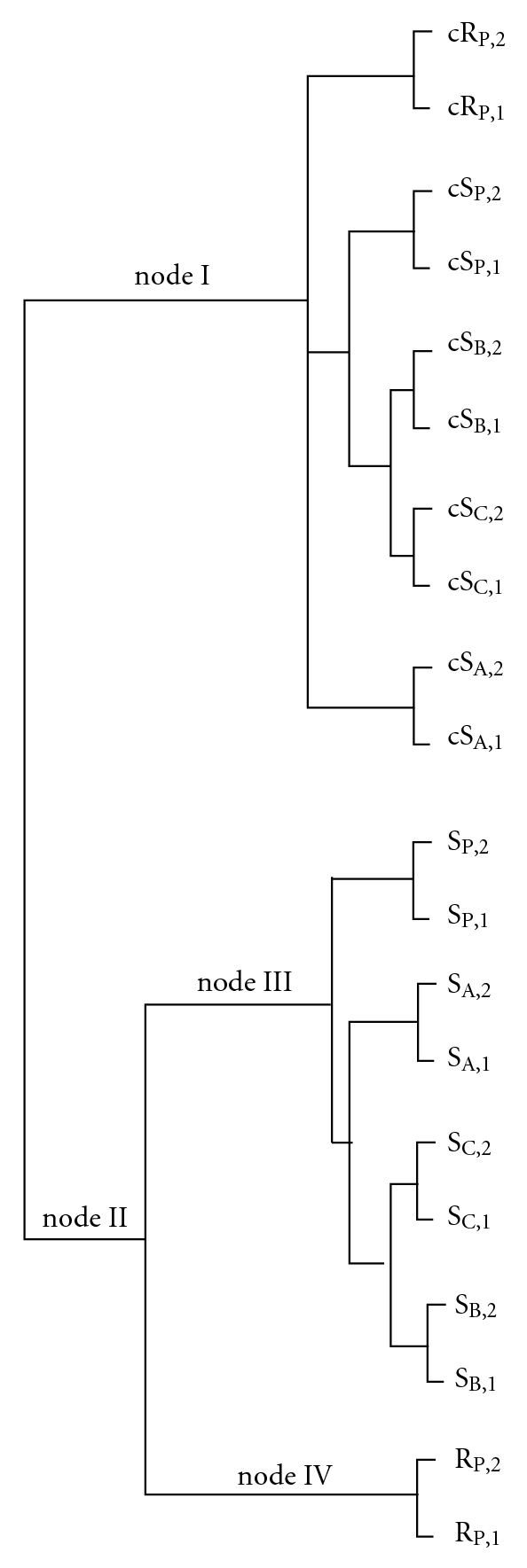
Clustering of the 20 quantitation categories based on the 349 proteins quantified from the 3rd gel fraction for the five protein samples S_P_, R_P_, S_A_, S_B_, and S_C_. Each protein sample contained the proteins from an unlabeled culture plus the labeled proteins from the control culture. Because a sample was run twice in LC/MS analysis, each protein sample had four quantitation categories of which two were for the unlabeled proteins and two for the labeled proteins. For example, S_P_ had S_P,1_ and S_P,2_ for the unlabeled proteins from culture S and cS_P,1_ and cS_P,2_ for the labeled proteins from culture C. The subscripts “1” and “2” indicate the duplicate LC/MS injections for a sample.

**Figure 5:**
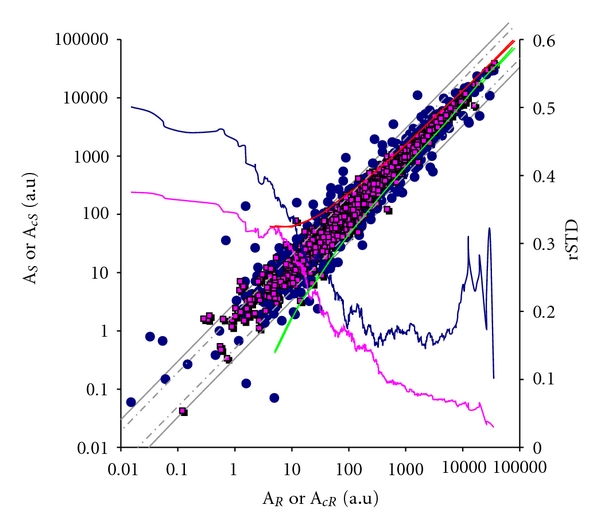
*A*_PRO_ scatter plots, local variability, and thresholds for selecting differentially regulated proteins. The blue dots represent the *A*_PRO_ scatter plot of *A*_S_ versus *A*_R_ corresponding to the unlabeled proteins in sample S_P_ versus R_P_. *A*_S_ is the average of *A*_S,1_ and *A*_S,2_. *A*_R_ is the average of *A*_R,1_ and *A*_R,2_. The red dots represent the *A*_PRO_ scatter plot of *A*_cS_ versus *A*_cR_ corresponding to the labeled proteins in control sample replicate cS_P_ versus cR_P_. *A*_cS_ is the average of *A*_cS,1_ and *A*_cS,2_. *A*_cR_ is the average of *A*_cR,1_ and *A*_cR,2_. *A*_S,1_, *A*_S,2_, *A*_R,1_, *A*_R,2_, *A*_cS,1_, *A*_cS,2_, *A*_cR,1_, and *A*_cR,2_ were the *A*_PRO_ values for the eight quantitation categories defined in Table 1. To evaluate the local noise of *A*_PRO_ measurement, the relative standard deviation (rSTD) for each protein was calculated from its four unlabeled *A*_PRO_ values *A*_S,1_, *A*_S,2_, *A*_R,1_, and *A*_R,2_ (the blue trace) or its four labeled *A*_PRO_ values *A*_cS,1_, *A*_cS,2_, *A*_cR,1_, and *A*_cR,2_ (the pink trace). The rSTD- *A*_PRO_ traces were smoothed with a 100-point moving box. The grey straight lines indicated a 3-fold (solid line) and a 2-fold (dashed line) change threshold. The solid red and green curves represent the fold-change thresholds established with the PLGEM-STN statistics based on the local variance in the null distribution (the pink-dot scatter plot).

We performed a two-step normalization procedure in which the information about the abundance of a peptide or protein in a sample was preserved (Figure 3). The preservation of the information about the abundance of a peptide or protein in the samples is critical for performing the PLGEM-STN analysis. In addition, because protein extracted ion chromatographic intensity was represented by the sum of the PCS extracted ion chromatographic intensities belonging to that protein, the summation weighed the low-intensity PCSs less than the high-intensity PCSs. Such a summation of PCS extracted ion chromatographic intensities probably suppressed noise from lower-intensity PCSs. When a protein abundance ratio is calculated as the average of PCS abundance ratios without weighing, the noise from a lower-intensity PCS would be amplified. We have avoided this potential issue by summing the PCS intensities to represent protein abundances before calculating protein abundance ratios.

**Figure 6:**
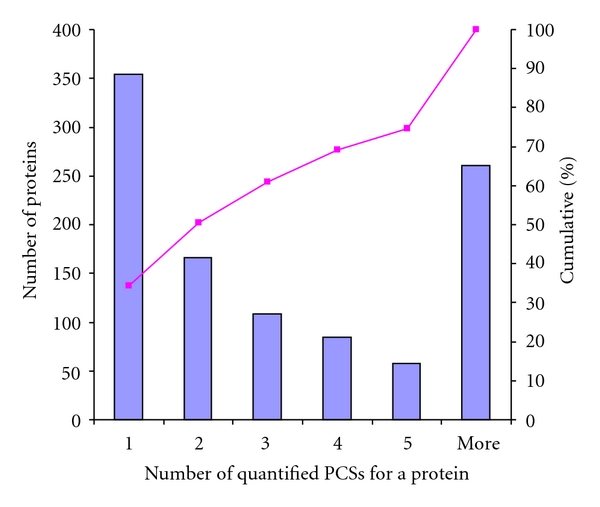
Histogram of PCS numbers for the 1032 quantified proteins.

Single-peptide proteins made up about 35% of the quantified proteins (Figure 6). Selection of differentially regulated proteins from these single-peptide proteins required a significance assessment method that did not rely on multiple-peptide detection to calculate a statistic about the confidence of a protein differential abundance. The use of a statistic that does not rely on the detection of multiple peptides is especially useful when the sample replicates are too low to use a typical statistical test such as a *t*-test. PLGEM-STN was a method that fits this criterion.

**Figure 7:**
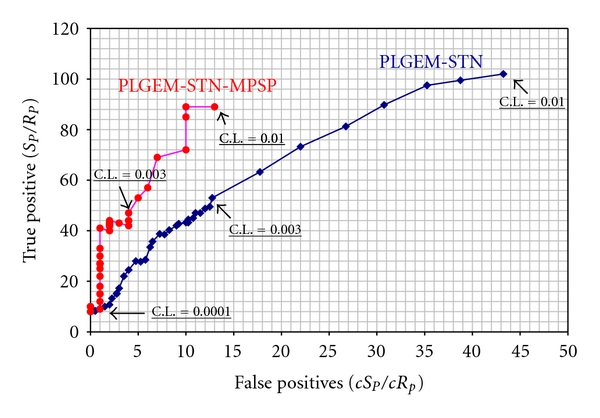
Receiver operating characteristic analysis of the PLGEM-STN approach with (red curve) or without (blue curve) the combination with MPSP. *Positives* are the differentially regulated proteins selected from the comparison of proteins abundances between samples S_P_ and R_P_ (see Table 1 and Figure 1 for definition). *False positives* are the differentially regulated proteins selected from the comparison of proteins abundances between samples cS_P_ and cR_P_ (see Table 1 for definition). *True positives* are estimated by subtracting false positives from the positives. For each approach that is, PLGEM-STN-MPSP or PLGEM-STN, 37 data points at different confidence levels (C.L.) are plotted in this figure, starting from C.L. = 0.0001 up to C.L. = 0.01. The relationship between STN thresholds and C.L. can be found in Figure S2. The increment is 0.001 between C.L. of 0.0001 and 0.003 (30 data points). Between C.L. of 0.0.003 and 0.01, the increment is 0.01 (7 data points). The use of PLGEM here is similar to that in Table 3.

**Figure 8:**
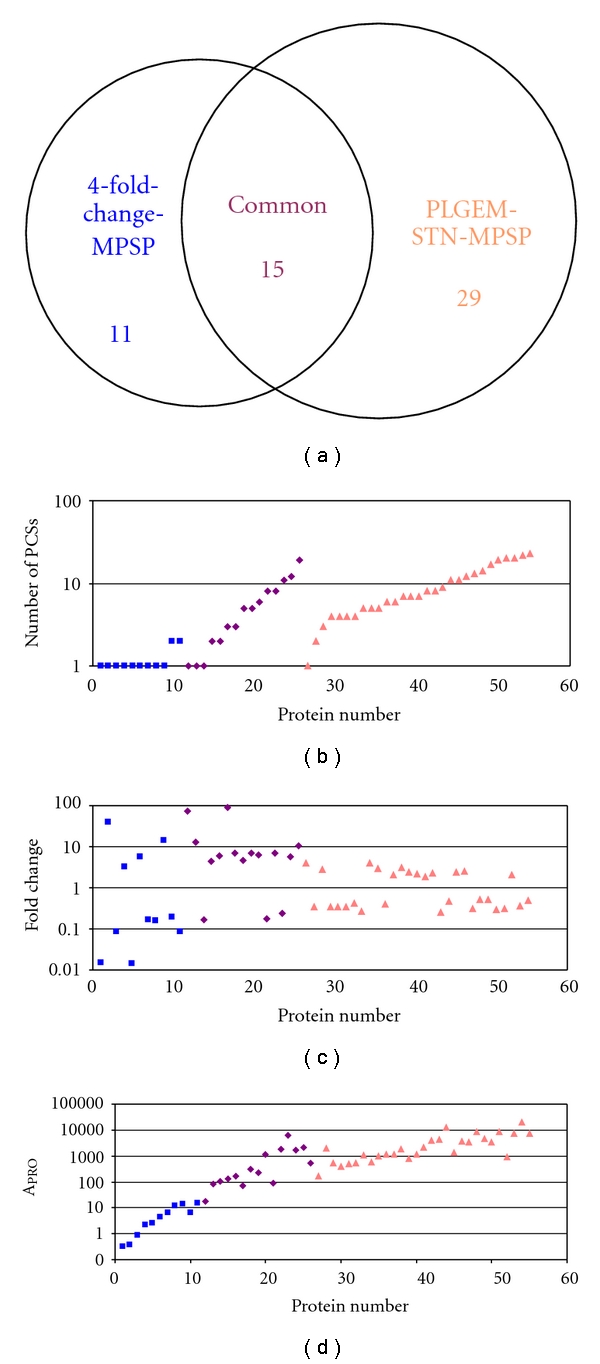
Comparison of the 26 and 44 differentially regulated proteins respectively selected by the 4-fold-change-MPSP and PLGEM-STN-MPSP approaches at a 5% false discovery rate. (a) Overlap of the two sets of differentially regulated proteins. Panels (b–d) show the distributions of (b) the number of detected PCSs, (c) the fold changes, and (d) the abundances of the quantified proteins. The blue square, the purple diamond, and the tan triangle markers represent the differentially regulated proteins selected by 4-fold-change-MPSP only, by both, and by PLGEM-STN-MPSP respectively. The protein number was from 1 to 55 on the x-axis representing the 55 unique proteins ranked according to their *A*_PRO_ in each of the three groups (blue, purple, or tan).

However, PLGEM-STN alone was not strict enough to control the false discovery rate without further diminishing the number of positives (Figure 7). The lack of stringency by using the PLGEM-STN method alone was similar to that by using the *t*-test alone [6]. In that prior study, the lack of specificity with a *t*-test alone was overcome by introducing the rule MPSP. The MPSP rule simply required that a protein be selected as differentially regulated only when it was repeatedly found so in certain number of permuted sample pairings. The MPSP rule was introduced to deal with datasets with small replicates where other more sophisticated statistical tests could not be applied [6]. Although the MPSP rule was originally used in combination with a *t*-test statistic and a fold-change threshold, this study shows that it can be used in combination with other types of statistical tests such as the PLGEM-STN method (Figure 7).

The combination of the MPSP rule allowed the selection of differentially regulated proteins at a false discovery rate <5%, which would have been impossible for a fold-change method, at least for the data used in this study (see Figure S3 supplementary material available online at doi:10.1155/2010/731582). The MPSP rule significantly reduced false positives while keeping the number of true positives relatively constant, thus effectively improving the statistical confidence of the selected differentially regulated proteins by lowering the false discovery rate (Table 4). The results from this and the prior study [6] suggest that MPSP is a rule that can be used in combination with different types of statistics to select differentially regulated proteins. 

The label-free quantitation simplified cell culturing and sample preparation. Another useful aspect of the label-free quantitation is that peptide cross-reference could be used to increase the number of proteins quantified in all of the samples run under the same condition [13]. Lipton et al. [20] introduced the concept of accurate mass and elution time peptide tag for global protein quantitation using high resolution mass spectrometry. One advantage of this method over using the spectral counting method is that the large number of identifications that occur in a LC/MS injection can be used as the basis for improved quantitation of another LC/MS injection [13, 21, 22]. The accurate mass and elution time peptide tag approach uses the extracted ion chromatographic intensities as the quantitative measurement of peptides and proteins. The linear response of peptide extracted ion chromatographic intensities to protein quantities was demonstrated [23–25]. This method was thus used to improve the comparability of proteins quantified between samples, among LC/MS injections, and for different isotopic forms of a protein [14]. The quantitation of 349 proteins from a single gel fraction for several samples clearly demonstrated the power of the peptide cross-reference feature in extracted ion chromatographic intensity-based label-free quantitative proteomics [6].

One drawback of extracted ion chromatographic intensity-based label-free quantitative proteomics is that the success of an analysis critically depends upon the reproducibility of LC/MS runs that have to be maintained across multiple samples. The reproducibility of LC/MS runs across multiple samples is a prerequisite to reliable peptide cross-reference [13]. With the advancement in LC/MS instrumentation and the availability of improved LC/MS chromatogram alignment methods [26, 27], the reproducibility of LC/MS runs is unlikely to remain an obstacle for the increasing use of label-free quantitative proteomics.

## 5. Conclusion

A label-free quantitative proteomics scheme was demonstrated to select differentially regulated proteins with single-peptide hits and with <2-fold changes at a 5% false discovery rate. 

The label-free quantitation scheme incorporated a labeled internal control into multiple unlabeled samples to facilitate error modeling when there were no replicates for the unlabeled samples. The error modeling allowed the use of the PLGEM-STN statistic to facilitate the selection of differentially regulated proteins with single-peptide hits. The PLGEM-STN statistic also facilitated the selection of differentially regulated proteins at different fold-change thresholds according to the local abundance level of the proteins. While the PLGEM-STN statistic uncovered more differentially regulated proteins at higher abundance with smaller fold-changes, the PLGEM error modeling of local variance versus abundance overpenalized the proteins with lower abundance. With a constant fold-change threshold, however, differentially regulated proteins with higher abundance were overlooked. Thus, the results from this study showed that the PLGEM-STN and a constant fold-change threshold were complementary to each other and could be used simultaneously. But, neither the PLGEM-STN nor the 4-fold-change criterion alone was stringent enough for selecting differentially regulated proteins at a 5% false discovery rate.

MPSP was introduced and shown to be a rule that could decrease false discovery rates when being used in combination with the PLGEM-STN statistic or the 4-fold-change threshold. The MPSP rule played a critical role in extending the selection of differentially regulated proteins to those with a single-peptide hit or with a lower fold-change in label-free proteomics when sample replicates were limited. Although the approaches were demonstrated for a representative replicate-limited scenario, they potentially can also be applicable to a situation where more sample replicates are available.

## Supplementary Material

The Supplementary Materials contains three major parts as outlined in the Table of Contents in the PDF file of the Supplementary Materials. Part I contains the web link to download the raw data of the 20 LC/MS runs for the pH 5 (Sp) and pH 7 (Rp) samples fractionated with SDS/PAGE gel separation. Descriptions for the LC/MS runs are provided. Part II contains the details of PLGEM-STN noise modeling, the use of the combination of MPSP with the PLGEM-STN or the fold-change method, and the list of the differentially regulated proteins selected by the combination of MPSP with PLGEM-STN and fold-change methods. Part III contains the lists of peptides and proteins quantified from the 20 LC/MS runs of the SDS/PAGE gel-fractionated Sp and Rp samples.

## Figures and Tables

**Table 1:** The five unlabeled protein samples from culture S or R, the five protein sample preparation replicates of the labeled proteins from culture C, and the corresponding 20 quantitation categories (see Methods)^a^.

	Unlabeled protein sample from culture S or R					[^15^N]-labeled protein sample from control culture C				
	S_P_	R_P_	S_A_	S_B_	S_C_	cS_P_	cR_P_	cS_A_	cS_B_	cS_C_
Quantitation category	S_P,1_	R_P,1_	S_A,1_	S_B,1_	S_C,1_	cS_P,1_	cR_P,1_	cS_A,1_	cS_B,1_	cS_C,1_
	S_P,2_	R_P,2_	S_A,2_	S_B,2_	S_C,2_	cS_P,2_	cR_P,2_	cS_A,2_	cS_B,2_	cS_C,2_

^a^S_A_, S_B_, and S_C_ were the unlabeled protein samples from the S culture triplicates. S_P_ was the pool of S_A_, S_B_, and S_C_. R_P_ was the pool of the unlabeled proteins from the culture R triplicates. The subscripts “1” and “2” represent the LC/MS duplicates. Only the 3rd gel fraction for samples S_A_, S_B_, and S_C_ along with the labeled control in them was analyzed by LC/MS. All of the five gel fractions were analyzed with LC/MS for samples S_P_ and R_P_ along with the labeled control in them.

**Table 2:** Overview of the key steps in extended selection of differentially regulated proteins.

Step	Procedure	Justification	Utilized data
1	Establish a null distribution	A null distribution affords an estimation of measurement noise originated from biological sample preparations and analytical procedure. The noise will dictate the threshold cutoff to distinguish regulated proteins from unregulated ones.	Protein abundances *A*_cS_ and *A*_cR_ in the four quantitation categories cS_P,1_, cS_P,2_, cR_P,1_, and cR_P,2_ (Figure 1; Table 1). These four quantitation categories represent the replicate analyses of the same [^15^N]-labeled control protein sample run together with the other two unlabeled protein samples. Thus, regulated proteins are not expected from any pairing between these four quantitation categories.
2	Model local noise in the null distribution	The measurement noise is not evenly distributed throughout the range of different peptide and protein abundances. Instead, the noise is locally dependent upon the signal strength that is, peptide and protein abundances, in a region. Thus, the threshold to select regulated proteins could be different at different protein abundance level. Modeling the distribution of noise according to protein abundances will help to discern more subtle changes for more abundant proteins while reduce the false positives for less abundant proteins.	

3	Select regulated proteins with PLGEM-STN statistic	PLGEM-STN statistic has been used in analyzing microarray data and spectral-count based quantitative proteomics data. The PLGEM approach establishes the distribution of noise according to gene/protein abundance level. In combination with STN statistic, adaptive thresholds are applied according to the protein abundance levels to maximize the selection of regulated proteins at higher abundance level while reduce the false positives for lower abundance proteins.	*For determining false positives*: Use the protein abundances *A*_cS_ and *A*_cR_ in the four quantitation categories cS_P,1_, cS_P,2_, cR_P,1_, and cR_P,2_ (Figure 1; Table 1). *For determining positives*: Use the protein abundances *A*_S_ and *A*_R_ in the four quantitation categories S_P,1_, S_P,2_, R_P,1_, and R_P,2_ (Figure 1; Table 1). S_P,1_ and S_P,2_ represent the duplicate analyses of the unlabeled protein sample S_P_ originated from the acid stressed culture S. R_P,1_ and R_P,2_ represent the duplicate analyses of the unlabeled protein sample R_P_ originated from the reference neutral pH culture R. Thus, regulated proteins are expected from any pairings between these four quantitation categories that is, S_P,1_, S_P,2_, R_P,1_, and R_P,2_.
4	Apply the MPSP rule	Due to the imperfection commonly found in many data sets and statistical models, the PLGEM-STN was not stringent enough to reduce false discovery rates in the label-free quantitative proteomics analysis. The MPSP rule is introduce to further reduce false discovery rates. The MPSP rule simply requires that a protein is accepted as a regulated one only if it is found regulated in multiple permutations of sample pairings using any kind of statistics, such as a *t*-test, PLGEM-STN, or even a fold-change threshold.	
5	Select regulated proteins with the PLGEM-STN-MPSP approach	The use of a combination of PLGEM-STN-MPSP approach reduces false discovery rates compared to PLGEM-STN statistic alone.	
6	Select regulated proteins with a fold-change-MPSP approach	The PLGEM-STN statistic over-penalizes the proteins with low abundances. A fold-change threshold in combination with MPSP is found more effective to select regulated proteins in the lower abundance region.	
7	Comparison of the PLGEM-STN-MPSP and fold-change-MPSP approaches	While the PLGEM-STN-MPSP approach over-penalizes lower-abundance proteins, the fold-change-MPSP approach over-penalizes the higher-abundance proteins. Thus, the two approaches are complimentary and can be used in combination.	

**Table 3:** Numbers of differentially regulated proteins selected with PLGEM-STN alone or in combination with MPSP^a^.

PLGEM-STN confidence level	FP, P, and FDR	PLGEM-STN					PLGEM-STN-MPSP
		Permuted sample pairings				Average	
		I	II	III	IV		
0.01	FP (cS_P_/ cR_P_)	31	68	22	46	42	13
	P (S_P_/R_P_)	141	155	134	148	145	101
	FDR	0.22	0.44	0.16	0.31	0.29	0.13

0.002	FP (cS_P_/ cR_P_)	6	15	3	9	8	2
	P (S_P_/R_P_)	47	50	46	51	49	44
	FDR	0.13	0.30	0.07	0.18	0.16	0.05

^a^False positives (FP) were selected from sample pair cS_P_/cR_P_. Positives (P) were selected from sample pair S_P_/R_P_. False discovery rate (FDR) was FP/P. The four permuted sample pairings (I–IV) were generated from the four LC/MS injections for a sample pair. See text.

**Table 4:** Numbers of differentially regulated proteins selected with fold-change threshold alone or in combination with MPSP.

Fold change	FP, P, and FDR	Fold-change					Fold-change-MPSP
		Permuted samplepairings				Average	
		I	II	III	IV		
2	FP (cS_P_/cR_P_)	68	77	118	45	77	22
	P (S_P_/R_P_)	171	154	186	147	165	104
	FDR	0.40	0.50	0.63	0.31	0.47	0.21

3	FP (cS_P_/cR_P_)	30	33	47	20	33	9
	P (S_P_/R_P_)	66	70	85	60	70	42
	FDR	0.45	0.47	0.55	0.33	0.47	0.21

4	FP (cS_P_/cR_P_)	17	24	32	10	21	1
	P (S_P_/R_P_)	42	50	53	35	45	26
	FDR	0.40	0.48	0.60	0.29	0.47	0.04

## Abbreviations

PLGEM: Power Law Global Error Model
STN: Signal-To-Noise ratio
MPSP: Minimum number of Permuted Significant Pairings.
