# Supplementary material for: Assigning Significance in Label-Free Quantitative Proteomics to Include Single-Peptide-Hit Proteins with Low Replicates
Source: Int J Proteomics. 2010 Jul 25;2010:731582. doi: 10.1155/2010/731582 (PMC2997754; doi:10.1155/2010/731582)
Supplement: Supplementary file 1 — The Supplementary Materials contains three major parts as outlined in the Table of Contents in the PDF file of the Supplementary Materials. Part I contains the web link to download the raw data of the 20 LC/MS runs for the pH 5 (Sp) and pH 7 (Rp) samples fractionated with SDS/PAGE gel separation. Descriptions for the LC/MS runs are provided. Part II contains the details of PLGEM-STN noise modeling, the use of the combination of MPSP with the PLGEM-STN or the fold-change method, and the list of the differentially regulated proteins selected by the combination of MPSP with PLGEM-STN and fold-change methods. Part III contains the lists of peptides and proteins quantified from the 20 LC/MS runs of the SDS/PAGE gel-fractionated Sp and Rp samples. [file 731582.f1.pdf]

Supplementary Materials for

Assigning significance in label-free quantitative proteomics to include single-peptide-hit proteins with low replicates

Qingbo Li

Center for Pharmaceutical Biotechnology, College of Pharmacy, University of Illinois at Chicago, Chicago, IL 60607

Department of Microbiology and Immunology, College of Medicine, University of Illinois at Chicago, Chicago, IL 60612

Description of the Supplementary Materials

The Supplementary Materials contains three major parts as outlined in the Table of Contents.

- Part I contains the web address to download the raw data of the 20 LC/MS runs for the pH 5 (S_P) and pH 7 (R_P) samples fractionated with SDS/PAGE gel separation. Descriptions for the data are provided.
- Part II contains the details of PLGEM-STN noise modeling, the use of the combination of MPSP with the PLGEM-STN or the fold-change method, and the list of the differentially regulated proteins selected by the combination of MPSP with PLGEM-STN and fold-change methods.
- Part III contains the lists of peptides and proteins quantified from the 20 LC/MS runs of the SDS/PAGE gel-fractionated S_P and R_P samples as described in part I.

Table of Contents

PART I. Downloadable LC/MS raw data files ... 3
PART II. Details of PLGEM-STN-MPSP and fold-change-MPSP analyses ... 4
Scheme S1. Major steps in PLGEM-STN ... 4
Figure S1. Residual analysis of the PLGEM fitting ... 5
Figure S2. The STN upper and lower thresholds at different confidence levels. ... 6
Figure S3. ROC analysis of the fold-change approach with (pink curves) and without (blue curves) the combination with MPSP. ... 6
Table S1. Examples to select regulated proteins with PLGEM-STN alone or in combintaiotn with MPSP. ... 7
Table S2. The 26 and 44 differentially regulated proteins identified at a false discovery rate of 0.05 by 4-fold-change-MPSP and PLGEM-STN-MPSP respectively. ... 7
PART III. Lists of peptides and proteins quantified from the S_P and R_P samples ... 9
Supplemental Table I. List of quantified proteins ... 10
Supplemental Table II. List of quantified peptides ... 94

PART I. Downloadable LC/MS raw data files

The raw LC/MS data associated with this manuscript may be downloaded from the ProteomeCommons.org Tranche network using the following hash:

xPDQl5X75crsPyDibct4onLk9wYo04dJerHJbnoFM9nSHEy4ZJw+TTg6DwtdSmHxNoDoJk3e
kX4HADPJYFiJvGNuXQMAAAAAAAACtA==

The above address contains the raw data of the 20 LC/MS runs for the pH 5 (S_P) and pH 7 (R_P) samples fractionated with SDS/PAGE gel separation. The 20 RAW files and one Excel spreadsheet were zipped into one file. The Excel spreadsheet lists the 20 RAW file names, the corresponding samples, 1D SDS/PAGE gel fractions, and LC/MS injections performed for each fraction, as following.

LC/MS injection	LC/MS data file name	Sample	Gel fraction
1	J037ID04	pooled pH7 (Rp)	1
2	J037ID05		1
3	J037ID09		2
4	J037ID10		2
5	J037ID13		3
6	J037ID14		3
7	J037ID17		4
8	J037ID18		4
9	J037ID21		5
10	J037ID22		5
11	J037ID42	pooled pH5 (Sp)	1
12	J037ID43		1
13	J037ID47		2
14	J037ID48		2
15	J037ID52		3
16	J037ID53		3
17	J037ID56		4
18	J037ID57		4
19	J037ID61		5
20	J037ID62		5

PART II. Details of PLGEM-STN-MPSP and fold-change-MPSP analyses

Scheme S1. Major steps in PLGEM-STN

These steps used the algorithm and followed the procedures described by Pavelka et al. (*BMC Bioinformatics* **2004,** 5, 203).

Step 1: For the 1032 proteins, the measurement noise was fit with the PLGEM by using the four A_{PRO} quantitation categories including $cS_{P,1}$, $cS_{P,2}$, $cR_{P,1}$, and $cR_{P,2}$ (Table 1). The PLGEM fitting generated the following error model

$$\ln(STD) = 0.746\ln(\overline{A}_C) - 0.776 + \varepsilon \qquad (1)$$

where *STD* and $\overline{A}_C$ respectively were the standard deviation and mean of the A_{PRO} values of $cS_{P,1}$, $cS_{P,2}$, $cR_{P,1}$, and $cR_{P,2}$. ε was the error term. The PLGEM fitting was performed with 100 partitions and a 0.5 modeling quantile. The fitting had an R^2 of 0.97 and the distribution of the model residuals ε followed a normal distribution as expected (**Figure S1**).

Step 2: For each protein, the 4128 A_{PRO} values of $cS_{P,1}$, $cS_{P,2}$, $cR_{P,1}$, and $cR_{P,2}$ were re-sampled with replacement for 500 iterations to calculate the null distribution of the PLGEM-STN statistic with the fitted PLGEM parameters from Equation (1). By pooling the statistics from the 500 iterations for the 1032 proteins, a null distribution with 51,600 PLGEM-STN statistics were generated. From the null distribution, STN thresholds were determined at a specific confidence level. A confidence level was a rough estimate of the false positive rate. For example, at a confidence level of 0.01, we would expect approximately 10 false positives from the 1032 proteins.

Step 3: The observed PLGEM-STN statistics were calculated for the four permuted pairings among $cS_{P,1}$, $cS_{P,2}$, $cR_{P,1}$, and $cR_{P,2}$ i.e., (I) $cS_{P,1}/cR_{P,1}$, (II) $cS_{P,1}/cR_{P,2}$, (III) $cS_{P,2}/cR_{P,1}$, and (IV) $cS_{P,2}/cR_{P,2}$. Similarly, the observed PLGEM-STN statistics were calculated for the four permuted sample pairings of measurements among $S_{P,1}$, $S_{P,2}$, $R_{P,1}$, and $R_{P,2}$ i.e., (I) $S_{P,1}/R_{P,1}$, (II) $S_{P,1}/R_{P,2}$, (III) $S_{P,2}/R_{P,1}$, and (IV) $S_{P,2}/R_{P,2}$ (see Table 2 in main text).

Step 4: Differentially expressed proteins were selected by choosing a confidence level (**Figure S2**). At a confidence level, a set pf STN thresholds were selected. The STN thresholds consisted of a negative threshold for down-regulated proteins and a positive threshold for up-regulated proteins. Based on the assessment of false discovery rates, two confidence levels were selected (see Table 2). At the confidence level of 0.002, the STN thresholds (-4.47, 4.58) result in two false positives in the sample pair cS_P/cR_P with 44 positives in the sample pair S_P/R_P when the MPSP rule was applied. At the confidence level of 0.01, the STN thresholds (-2.74, 2.79) result in 13 false positives in the sample pair cS_P/cR_P with 101 positives in the sample pairs S_P/R_P when the MPSP rule was applied. Coincidently, with the combination with the MPSP rule, the false positive rates approximated the STN confidence levels.

Figure S1. Residual analysis of the PLGEM fitting

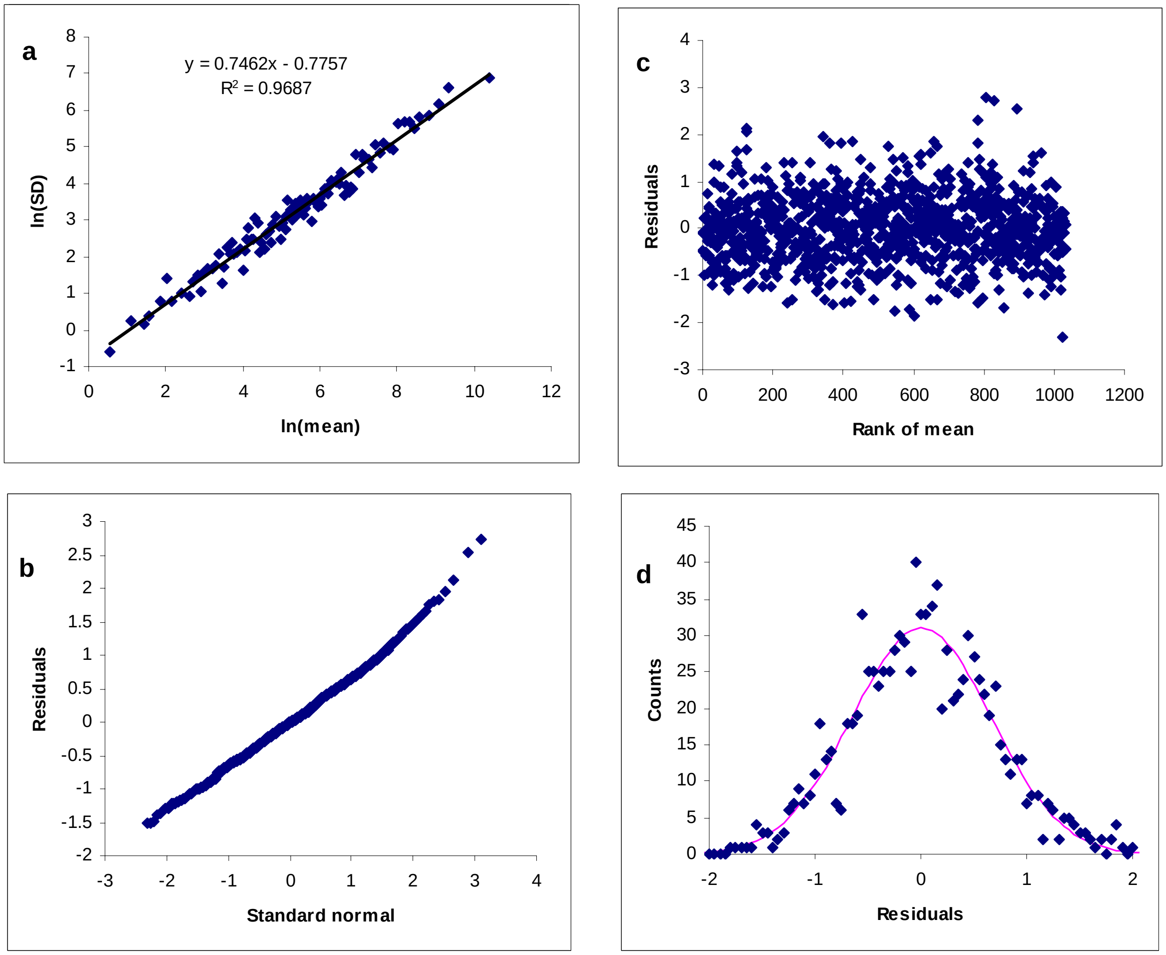

Figure S2. The STN upper and lower thresholds at different confidence levels.

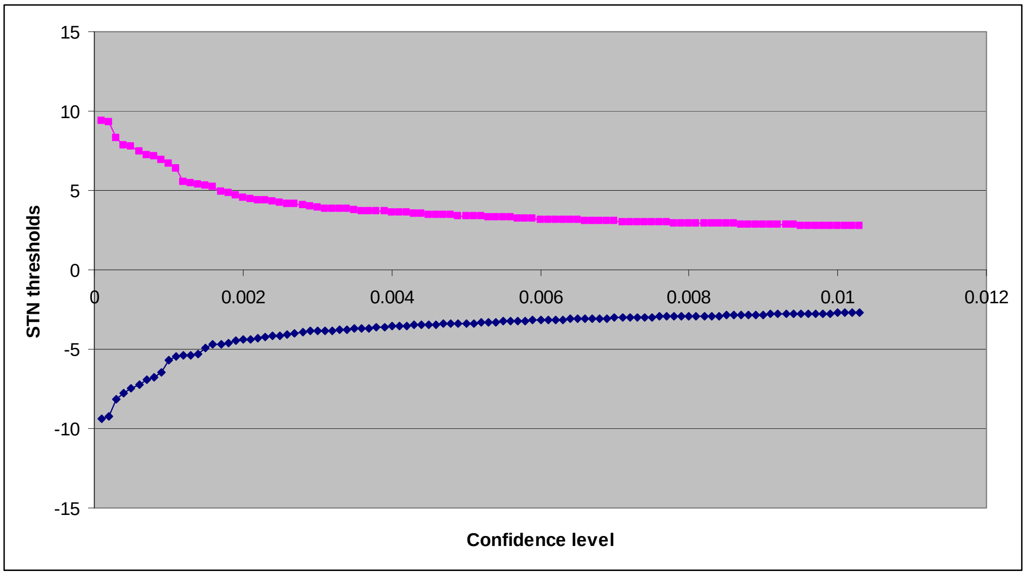

Figure S3. ROC analysis of the fold-change approach with (pink curves) and without (blue curves) the combination with MPSP.

Left panel: ROC curves. The insert shows the region with low number of false positives.

Right panel: false discovery rate at different fold changes.

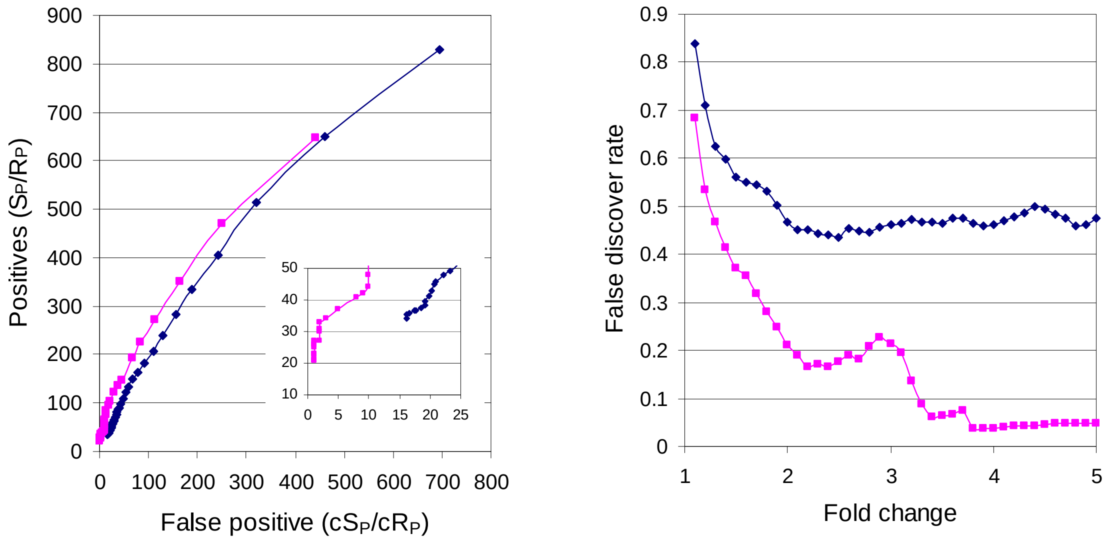

Table S1. Numbers of differentially regulated proteins selected with PLGEM-STN alone or in combination with MPSP. [a]

PLGEM-STN confidence level	FP, P, and FDR	PLGEM-STN					PLGEM-STN-MPSP
		Permuted sample pairings				Average	
		I	II	III	IV		
0.01	FP (cS_P/cR_P)	31	68	22	46	42	13
	P (S_P/R_P)	141	155	134	148	145	101
	FDR	0.22	0.44	0.16	0.31	0.29	0.13
0.002	FP (cS_P/cR_P)	6	15	3	9	8	2
	P (S_P/R_P)	47	50	46	51	49	44
	FDR	0.13	0.30	0.07	0.18	0.16	0.05

[a] False positives (FP) were selected from sample pair cS_P/cR_P. Positives (P) were selected from sample pair S_P/R_P. False discovery rate (FDR) was FP/P. The four permuted sample pairings (I-IV) were generated from the four LC/MS injections for a sample pair. See text.

Table S2. The 26 and 44 differentially regulated proteins identified at a false discovery rate of 0.05 by 4-fold-change-MPSP and PLGEM-STN-MPSP respectively.

Index	Protein locus	Approach	PCS counts	Average A_{PRO}	Fold-change
1	MSMEG_1683	4-fold-change-MPSP	1	0.3	0.02
2	MSMEG_1143	4-fold-change-MPSP	1	0.4	40.73
3	MSMEG_4888	4-fold-change-MPSP	1	1	0.08
4	MSMEG_5244	4-fold-change-MPSP	1	2	3.16
5	MSMEG_1605	4-fold-change-MPSP	1	3	0.01
6	MSMEG_3710	4-fold-change-MPSP	1	4	5.61
7	MSMEG_4075	4-fold-change-MPSP	1	7	0.17
8	MSMEG_6210	4-fold-change-MPSP	1	12	0.16
9	MSMEG_4454	4-fold-change-MPSP	1	14	14.42
10	MSMEG_2116	4-fold-change-MPSP	2	6	0.20
11	MSMEG_1052	4-fold-change-MPSP	2	15	0.08
12	MSMEG_1888	both	1	18	74.03

13	MSMEG_3932	both	1	86	13.19
14	MSMEG_0067	both	1	102	0.17
15	MSMEG_1475	both	2	132	4.49
16	MSMEG_5136	both	2	166	5.85
17	MSMEG2164	both	3	70	91.81
18	MSMEG_5245	both	3	313	6.84
19	MSMEG_3945	both	5	228	4.71
20	MSMEG_5246	both	5	1114	6.89
21	MSMEG_5739	both	6	86	6.24
22	MSMEG_3962	both	8	1770	0.18
23	MSMEG_1037	both	8	6326	6.87
24	MSMEG_1682	both	11	1669	0.24
25	MSMEG_6454	both	12	2064	5.80
26	MSMEG_2956	both	19	513	10.63
27	MSMEG_5243	PLGEM-STN-MPSP	1	168	4.07
28	MSMEG_6097	PLGEM-STN-MPSP	2	1940	0.35
29	MSMEG_0969	PLGEM-STN-MPSP	3	509	2.85
30	MSMEG_3058	PLGEM-STN-MPSP	4	379	0.33
31	MSMEG_5225	PLGEM-STN-MPSP	4	479	0.34
32	MSMEG_4935	PLGEM-STN-MPSP	4	518	0.34
33	MSMEG_2078	PLGEM-STN-MPSP	4	1045	0.42
34	MSMEG_1680	PLGEM-STN-MPSP	5	555	0.27
35	MSMEG_2669	PLGEM-STN-MPSP	5	967	4.00
36	MSMEG_3950	PLGEM-STN-MPSP	5	1157	3.00
37	MSMEG_0919	PLGEM-STN-MPSP	6	1170	0.41
38	MSMEG_5715	PLGEM-STN-MPSP	6	1779	2.01
39	MSMEG_5419	PLGEM-STN-MPSP	7	782	3.01
40	MSMEG_2408	PLGEM-STN-MPSP	7	1163	2.35
41	MSMEG_3106	PLGEM-STN-MPSP	7	2157	2.17
42	MSMEG_4891	PLGEM-STN-MPSP	8	3994	1.85
43	MSMEG_0777	PLGEM-STN-MPSP	8	4362	2.30
44	MSMEG_4298	PLGEM-STN-MPSP	9	12334	0.25
45	MSMEG_3564	PLGEM-STN-MPSP	11	1361	0.47
46	MSMEG_3811	PLGEM-STN-MPSP	11	3688	2.39
47	MSMEG_4281	PLGEM-STN-MPSP	12	3286	2.51
48	MSMEG_0911	PLGEM-STN-MPSP	13	8247	0.31
49	MSMEG_3247	PLGEM-STN-MPSP	14	4396	0.52
50	MSMEG_3642	PLGEM-STN-MPSP	17	3295	0.52
51	MSMEG_4936	PLGEM-STN-MPSP	19	8312	0.29
52	MSMEG_1681	PLGEM-STN-MPSP	20	934	0.31
53	MSMEG_3143	PLGEM-STN-MPSP	20	7199	2.10
54	MSMEG_6759	PLGEM-STN-MPSP	22	20370	0.36
55	MSMEG_6638	PLGEM-STN-MPSP	23	7523	0.48

PART III. Lists of peptides and proteins quantified from the S_P and R_P samples

(In the following pages, the following two tables are provided)

Supplemental Table I. List of quantified proteins

Supplemental Table II. List of quantified peptides

Supplemental Table I. List of quantified proteins

Proteins				Protein quantitaion A_{PRO}							
				**unlabled proteins**				**labeled proteins**			
Locus	old locus	Description	**PCS counts**	**$S_{P,1}$**	**$S_{P,2}$**	**$R_{P,1}$**	**$R_{P,2}$**	**$cS_{P,1}$**	**$cS_{P,2}$**	**$cR_{P,1}$**	**$cR_{P,2}$**
MSMEG_0001	MSMEG 0001	DNA polymerase III, beta subunit (dnaN)	3	271.6	264.2	308.8	286.0	336.4	288.3	373.4	383.1
MSMEG_0002	MSMEG 0002	gnd	6	275.8	286.0	526.0	498.5	571.9	584.8	693.9	710.1
MSMEG_0005	MSMEG 0005	DNA gyrase, B subunit (gyrB)	14	1778.4	1844.9	2046.6	1962.3	1027.5	1022.2	1020.4	1014.7
MSMEG_0006	MSMEG 0006	DNA gyrase, A subunit (gyrA)	19	2510.5	2600.0	3065.9	3082.2	1072.4	1138.9	1250.9	1439.0
MSMEG_0023	MSMEG 0023	conserved hypothetical protein	3	469.2	540.0	268.0	277.3	384.8	452.1	391.0	383.9
MSMEG_0024	MSMEG 0024	COG0652:Peptidyl-prolyl cis-trans isomerase (rotamase) - cyclophilin	12	5140.5	5341.9	3559.3	3629.5	3524.0	3559.9	3474.5	3561.4
MSMEG_0033	MSMEG 0033	ppp (ppp)	1	316.7	251.6	259.6	232.3	77.2	85.2	48.9	56.2
MSMEG_0034	MSMEG 0034	FHA domain protein	1	11.4	11.7	18.8	21.0	25.5	31.0	21.4	20.1
MSMEG_0042	MSMEG 0042	transcription regulator, TetR family	1	16.4	19.0	9.1	13.0	9.8	10.5	12.5	9.8
MSMEG_0048	MSMEG 0048	conserved hypothetical protein	2	39.0	44.8	94.6	90.9	43.0	54.3	33.7	31.7

MSMEG _0057	MSMEG 0056	conserved hypothetical protein	1	6.9	5.9	18.2	18.1	53.1	45.5	31.6	32.7
MSMEG _0059	MSMEG 0058	ATPase, AAA family	4	85.5	121.9	53.1	112.4	387.0	435.4	234.4	341.0
MSMEG _0065	MSMEG 0064	conserved hypothetical protein	1	12.3	16.7	16.5	25.7	15.7	24.3	8.8	16.2
MSMEG _0066	MSMEG 0065	ea	1	81.1	76.4	159.9	177.5	116.5	101.3	117.6	125.8
MSMEG _0067	MSMEG 0066	conserved hypothetical protein	1	25.9	33.2	175.2	172.2	97.8	113.1	133.9	146.8
MSMEG _0076	MSMEG 0074	conserved hypothetical protein	1	0.2	0.6	0.2	0.7	22.4	33.0	8.6	26.9
MSMEG _0081	MSMEG 0078	conserved hypothetical protein	1	391.5	406.5	175.3	206.3	7.4	11.2	0.6	5.5
MSMEG _0084	MSMEG 0082	phosphocarrier protein HPr, putative	1	4.2	3.4	4.8	6.1	11.9	8.9	16.1	15.1
MSMEG _0091	MSMEG 0089	transcriptional regulatory protein, putative	1	12.2	18.0	15.0	19.0	57.7	54.5	17.9	27.8
MSMEG _0092	MSMEG 0090	transcriptional regulator, TetR family domain protein	10	393.0	462.9	461.7	485.9	674.2	693.2	495.3	546.3
MSMEG _0108	MSMEG 0105	acyl-CoA dehydrogenase (acdA-2)	1	112.1	94.9	108.9	108.8	56.6	53.8	92.5	91.2
MSMEG _0110	MSMEG 0107	NAD(P) transhydrogenase, alpha subunit (pntA)	3	38.6	42.5	80.5	83.9	160.7	151.5	180.3	135.4

MSMEG _0120	MSMEG 0116	transcriptional regulator, TetR family domain protein	1	8.8	8.2	9.9	13.5	99.9	102.9	52.0	70.2
MSMEG _0131	MSMEG 0125	substrate--CoA ligase (fadD5)	1	1.7	1.3	7.1	6.2	7.1	9.1	4.1	13.8
MSMEG _0166	MSMEG 0160	transcription regulator GntR family VC1335	1	23.1	24.1	25.5	31.2	23.8	22.9	41.6	39.1
MSMEG _0215	MSMEG 0205	PUTATIVE PIRIN-LIKE PROTEIN	1	2.9	1.8	0.8	2.0	6.1	3.6	3.7	4.1
MSMEG _0216	MSMEG 0206	3-hydroxyacyl-CoA dehydrogenase type II	7	3042.6	2954.1	2364.7	2533.3	2517.6	2481.8	3143.6	3358.8
MSMEG _0220	MSMEG 0210	lysophospholipase	1	4.5	4.4	3.4	3.0	11.6	17.9	9.4	19.5
MSMEG _0224	MSMEG 0214	O-methyltransferase	2	283.5	270.6	112.9	109.6	180.1	180.9	117.3	136.4
MSMEG _0229	MSMEG 0219	dihydroxy-acid dehydratase (ilvD)	6	2491.7	2401.7	1938.3	1870.8	2347.5	2118.9	2124.5	1892.6
MSMEG _0233	MSMEG 0223	lppS (lppS)	1	90.9	101.6	79.3	110.1	132.2	141.7	182.7	176.3
MSMEG _0234	MSMEG 0224	peptidase, M13 family (pepO)	15	3600.0	3541.5	2259.9	1961.9	1726.7	1681.1	1944.2	1651.3
MSMEG _0238	MSMEG 0229	CoA-binding domain protein	7	988.2	878.7	1162.1	1242.7	329.7	337.2	261.3	286.8
MSMEG _0239	MSMEG 0230	O-acetylhomoserine/O-acetylserine sulfhydrylase family protein (cysD)	3	579.2	521.1	627.6	576.8	462.1	429.1	477.8	470.7

MSMEG _0255	MSMEG 0246	Phosphoenolpyruvate carboxykinase	23	4385.9	4450.2	5396.0	5233.5	911.9	1024.4	911.7	1005.4
MSMEG _0312	MSMEG 0301	2-deydro-3-deoxyphosphogluconate aldolase/4-hydroxy-2-oxoglutarate al	1	13.8	13.0	7.0	10.5	155.6	143.7	130.8	116.0
MSMEG _0317	MSMEG 0306	conserved hypothetical protein	2	47.9	39.6	138.0	167.0	54.5	56.3	98.2	124.2
MSMEG _0356	MSMEG 0347	conserved hypothetical protein	1	24.6	29.9	8.2	9.2	12.9	19.2	9.7	8.0
MSMEG _0363	MSMEG 0355	transcriptional regulator, TetR family domain protein	1	8.7	8.8	1.8	3.1	13.0	10.4	8.9	9.9
MSMEG _0370	MSMEG 0363	conserved hypothetical protein	10	190.3	182.3	157.7	161.1	151.7	146.3	110.7	127.5
MSMEG _0371	MSMEG 0364	MaoC domain protein	3	58.9	64.0	60.3	59.0	145.2	147.2	103.1	107.0
MSMEG _0372	MSMEG 0365	fabG4 (fabG4)	8	2356.9	2008.7	1591.9	1563.1	1754.8	1616.4	2802.0	2426.0
MSMEG _0373	MSMEG 0366	fadA2 (fadA2)	3	716.2	720.4	451.3	452.2	830.6	840.4	786.3	754.6
MSMEG _0380	MSMEG 0373	TmtpA (mmpS1)	1	53.0	80.4	57.8	76.8	54.0	61.9	43.1	46.7
MSMEG _0384	MSMEG 0377	glucose-1-phosphate thymidylyltransferase (rfbA)	1	32.9	37.8	50.0	55.8	323.7	314.1	331.9	321.6
MSMEG _0386	MSMEG 0379	UDP-glucose 4-epimerase, putative	1	16.1	23.5	9.4	10.9	25.3	31.1	10.6	18.1

MSMEG _0387	MSMEG 0380	O-methyltransferase I	1	93.2	93.9	68.0	85.3	20.8	24.1	22.0	30.6
MSMEG _0388	MSMEG 0381	methyltransferase	6	287.7	282.5	482.6	534.0	426.9	445.1	566.9	595.9
MSMEG _0391	MSMEG 0384	methyltransferase	2	216.5	215.0	264.6	265.0	349.3	355.9	382.7	387.7
MSMEG _0393	MSMEG 0386	probable glycosyltransferase	4	31.0	40.6	51.1	58.1	216.6	228.5	209.4	206.4
MSMEG _0394	MSMEG 0387	hypothetical protein	4	17.7	12.4	71.0	58.4	41.9	32.3	52.8	54.9
MSMEG _0400	MSMEG 0390	peptide synthetase	51	1423.6	1446.9	2042.5	2110.7	2085.5	2001.0	2361.2	2055.5
MSMEG _0401	MSMEG 0391	pyoverdine synthetase	19	935.6	915.9	1122.6	1157.7	817.0	792.0	779.6	773.5
MSMEG _0402	MSMEG 0392	peptide synthetase	45	1477.5	1457.4	1635.2	1616.7	1504.2	1483.1	1438.3	1356.6
MSMEG _0406	MSMEG 0396	fadE5 (fadE5)	4	190.9	196.8	77.6	85.9	401.7	397.1	280.4	268.9
MSMEG _0408	MSMEG 0398	polyketide synthase	19	215.6	207.4	266.8	263.2	199.1	217.5	217.7	224.5
MSMEG _0415	MSMEG 0405	Flavin reductase like domain, putative	4	204.8	168.8	237.5	230.4	84.1	94.0	82.8	85.5
MSMEG _0422	MSMEG 0413	carboxyphosphonoenolpyruvate phosphonomutase-like protein	8	266.5	275.3	320.9	340.4	1047.3	1014.4	859.2	865.6
MSMEG _0456	MSMEG 0447	DNA gyrase, A subunit, putative	2	160.5	166.9	187.7	223.9	190.7	201.5	109.8	68.8
MSMEG _0457	MSMEG 0448	DNA gyrase subunit B, putative	2	68.6	77.7	76.8	93.8	13.9	18.2	13.0	18.6
MSMEG _0464	MSMEG 0456	phosphomethylpyrimidine kinase (thiD)	4	336.3	337.6	304.0	310.8	396.6	392.8	370.8	330.5

MSMEG _0530	MSMEG 0520	oxidoreductase, short-chain dehydrogenase/reductase family (fabG)	2	35.8	49.4	88.1	82.1	277.8	299.1	302.5	286.4
MSMEG _0531	MSMEG 0521	acyl-CoA dehydrogenase (acd-2)	2	262.5	265.3	246.8	224.3	192.9	191.0	154.3	166.2
MSMEG _0550	MSMEG 0541	ABC transporter, periplasmic substrate-binding protein	6	860.7	912.2	1271.2	1275.6	759.9	829.7	466.5	498.2
MSMEG _0551	MSMEG 0542	aliphatic sulfonates ABC transporter, ATP-binding protein	1	36.7	37.5	53.6	56.1	18.5	18.7	17.7	18.9
MSMEG _0572	MSMEG 0563	conserved hypothetical protein	5	70.9	81.1	69.2	79.8	3809.9	4353.6	3506.2	3006.0
MSMEG _0599	MSMEG 0590	fadD2 (fadD2)	2	25.9	34.1	37.8	30.2	168.0	184.1	165.1	151.9
MSMEG _0603	MSMEG 0594	fadE6 (fadE6)	1	18.6	16.7	31.5	39.3	54.4	43.1	49.5	35.3
MSMEG _0613	MSMEG 0603	conserved hypothetical protein	1	1.2	0.8	0.5	0.5	4.0	2.6	6.7	21.3
MSMEG _0614	MSMEG 0604	Mycobacterium tuberculosis paralogous family	1	87.6	98.7	25.1	28.0	32.4	34.0	15.7	24.0
MSMEG _0628	MSMEG 0619	Amidohydrolase family superfamily	1	69.2	74.4	58.9	64.3	78.0	98.7	72.6	81.0
MSMEG _0636	MSMEG 0627	conserved hypothetical protein	4	414.5	411.7	528.7	562.8	128.4	128.9	86.0	91.1

MSMEG _0640	MSMEG 0632	oligopeptide ABC transporter, ATP-binding protein (oppD)	1	11.0	10.3	19.5	15.8	49.3	46.7	20.3	27.9
MSMEG _0643	MSMEG 0635	heme-binding lipoprotein, putative	15	471.5	502.7	886.4	946.4	3387.0	3207.8	2579.2	2775.8
MSMEG _0683	MSMEG 0675	conserved hypothetical protein	2	31.3	36.6	26.9	30.8	47.2	62.2	40.2	48.2
MSMEG _0688	MSMEG 0680	aminotransferase, classes I and II	1	72.7	69.1	71.6	59.1	307.2	370.6	160.9	248.7
MSMEG _0690	MSMEG 0682	ferredoxin, 4Fe-4S	13	426.0	461.6	478.0	489.4	558.5	614.3	474.4	496.3
MSMEG _0692	MSMEG 0684	conserved hypothetical protein	4	94.5	111.5	95.1	109.6	222.3	230.2	237.1	231.5
MSMEG _0695	MSMEG 0689	conserved hypothetical protein	1	22.0	26.3	7.3	14.5	21.5	25.8	15.9	24.0
MSMEG _0702	MSMEG 0697	Luciferase-like monooxygenase superfamily	13	4940.7	5033.1	3123.9	3675.7	2897.4	2807.5	2589.2	2991.9
MSMEG _0703	MSMEG 0698	conserved hypothetical protein	4	71.1	79.5	56.2	67.3	279.2	262.1	249.7	246.6
MSMEG _0709	MSMEG 0704	DNAk protein	18	5357.7	5422.4	5344.4	4749.6	5089.5	5021.7	5512.3	4774.0
MSMEG _0710	MSMEG 0705	grpE protein (grpE)	3	265.1	332.2	148.8	219.5	227.4	283.6	131.4	181.7
MSMEG _0732	MSMEG 0726	heat shock protein	3	170.1	178.0	177.1	194.4	429.5	420.0	471.2	478.6
MSMEG _0741	MSMEG 0736	conserved hypothetical protein	6	388.3	421.9	495.6	482.5	403.0	426.7	397.5	413.5

MSMEG_0759	MSMEG0755	adenylosuccinate synthetase (purA)	5	393.8	381.8	504.2	542.7	643.3	624.3	733.7	779.0
MSMEG_0760	MSMEG0756	thioesterase family protein domain protein	2	59.9	64.2	56.4	68.6	104.1	92.3	98.9	107.1
MSMEG_0769	MSMEG0763	O-succinylhomoserine sulfhydrylase (metZ)	3	647.0	652.3	473.5	480.8	858.4	902.3	784.8	834.6
MSMEG_0777	MSMEG0772	F420-dependent glucose-6-phosphate dehydrogenase (mer-2)	8	6045.2	6120.4	2586.2	2696.6	4377.5	4331.6	3767.0	3865.6
MSMEG_0779	MSMEG0774	3-oxoacyl-(acyl-carrier-protein) reductase	2	163.2	174.6	110.4	116.3	324.3	336.4	315.8	330.9
MSMEG_0783	MSMEG0778	phosphate acetyltransferase (pta)	1	30.3	36.1	36.5	30.2	26.7	39.5	30.7	34.2
MSMEG_0789	MSMEG0785	thiamine-phosphate pyrophosphorylase (thiE)	2	128.9	132.8	97.5	93.3	124.5	123.8	114.5	107.9
MSMEG_0793	MSMEG0788	thiazole biosynthesis protein ThiG (thiG)	2	109.8	118.3	100.0	100.5	85.5	88.3	159.6	167.2
MSMEG_0814	MSMEG0807	hypothetical protein	5	383.9	408.9	520.0	486.4	418.8	439.5	505.3	474.0
MSMEG_0825	MSMEG0817	phosphomethylpyrimidine kinase (thiD)	5	556.9	564.6	716.2	760.6	917.0	893.1	851.9	948.0
MSMEG_0826	MSMEG0818	thiamin biosynthesis protein ThiC	1	111.6	116.1	45.8	53.2	15.3	16.4	5.2	17.5

MSMEG _0832	MSMEG 0826	peptide deformylase (def)	1	145.7	158.1	141.0	139.2	166.1	178.0	205.1	208.6
MSMEG _0834	MSMEG 0828	conserved hypothetical protein	1	75.9	81.9	84.2	98.6	64.4	85.4	21.2	48.2
MSMEG _0835	MSMEG 0829	copper/zinc superoxide dismutase (sodC)	2	699.0	657.1	829.0	730.8	1081.7	950.3	1179.2	1000.1
MSMEG _0858	MSMEG 0852	cell division control protein 48, AAA family (cdc48-1)	2	1398.3	1509.9	1251.2	1153.3	1183.4	1196.1	1113.4	1081.2
MSMEG _0859	MSMEG 0851	Aha1 domain family	5	182.8	189.2	140.8	158.9	209.0	214.5	206.5	215.4
MSMEG _0861	MSMEG 0854	phosphatidylserine decarboxylase (psd)	3	52.2	88.9	28.0	36.7	4.2	5.9	4.1	3.4
MSMEG _0863	MSMEG 0856	oxidoreductase, short-chain dehydrogenase/reductase family	2	114.4	120.2	126.1	144.8	263.9	268.2	308.2	262.8
MSMEG _0876	MSMEG 0871	oxidoreductase, short-chain dehydrogenase/reductase family	2	71.4	83.4	50.4	50.1	85.5	99.3	106.2	116.1
MSMEG _0877	MSMEG 0870	dihydrodipicolinate synthase family protein	2	95.3	105.3	88.6	103.1	64.3	65.3	49.0	62.2
MSMEG _0880	MSMEG 0873	60 kd chaperonin , fragment	24	24758.5	25511.7	25648.9	26468.9	16427.4	17058.2	15398.6	16674.9
MSMEG _0882	MSMEG 0875	conserved hypothetical protein	1	415.7	464.3	449.9	477.5	426.3	467.0	377.2	392.1
MSMEG _0884	MSMEG 0877	glyoxalase family protein	1	21.2	23.7	26.1	31.5	24.6	31.1	27.9	34.6

MSMEG _0887	MSMEG 0880	conserved hypothetical protein	1	149.1	162.2	91.1	108.7	215.2	206.9	164.4	198.9
MSMEG _0889	MSMEG 0882	gabD1 (gabD1)	1	6.9	3.6	9.3	7.9	221.1	208.1	307.2	248.2
MSMEG _0899	MSMEG 0893	prolyl oligopeptidase family protein (pep)	4	188.3	178.2	172.3	127.2	206.1	224.7	223.8	213.7
MSMEG _0903	MSMEG 0897	dihydrolipoamide dehydrogenase (lpdA)	13	7390.5	7313.7	7873.1	8058.1	7997.3	8028.5	9042.2	9388.6
MSMEG _0905	MSMEG 0899	conserved hypothetical protein	2	65.4	75.5	76.2	38.2	88.1	55.1	153.7	67.5
MSMEG _0909	MSMEG 0903	conserved hypothetical protein	2	72.8	80.2	67.9	70.7	57.5	56.9	58.6	64.0
MSMEG _0911	MSMEG 0904	isocitrate lyase (aceA)	13	3803.7	4014.2	12486.0	12684.8	707.5	855.3	595.7	709.7
MSMEG _0912	MSMEG 0905	fadB2 (fadB2)	9	1326.4	1308.0	2031.8	2031.5	1790.3	1694.5	1966.9	1883.2
MSMEG _0913	MSMEG 0906	methoxy mycolic acid synthase 1 (umaA1)	15	2960.1	3072.9	4195.9	4127.8	1531.9	1602.4	1539.5	1641.4
MSMEG _0916	MSMEG 0909	transcriptional regulator, TetR family	1	39.5	37.9	36.8	45.8	53.7	49.2	17.9	31.7
MSMEG _0918	MSMEG 0911	Helix-turn-helix domain protein	3	35.7	40.8	67.5	65.2	369.8	380.5	384.1	368.5
MSMEG _0919	MSMEG 0912	conserved hypothetical protein	6	638.7	717.5	1634.0	1690.6	6661.6	7415.7	6870.1	7187.6
MSMEG _0924	MSMEG 0916	carbon-nitrogen hydrolase family protein, putative	1	66.6	73.0	59.2	70.1	15.2	16.4	12.6	17.2

MSMEG _0927	MSMEG 0919	conserved hypothetical protein	3	34.0	35.6	21.4	25.1	52.2	54.9	36.6	39.2
MSMEG _0930	MSMEG 0922	oxidoreductase, short chain dehydrogenase/reductase family	6	524.6	525.3	423.9	358.3	386.7	399.0	453.6	416.4
MSMEG _0935	MSMEG 0927	phosphoglycerate mutase	15	3506.4	3534.0	3005.8	2927.4	7198.7	7540.3	15103.5	16617.2
MSMEG _0937	MSMEG 0929	DNA-binding response regulator RegX3 (regX3)	1	17.1	21.2	26.2	32.9	4.9	7.5	6.5	7.2
MSMEG _0943	MSMEG 0934	pyrroline-5-carboxylate reductase (proC)	4	653.9	714.8	633.3	646.9	333.8	325.0	372.3	381.2
MSMEG _0944	MSMEG 0935	excisionase, putative	2	61.4	62.7	61.7	58.7	237.5	239.5	225.2	218.4
MSMEG _0948	MSMEG 0939	Actinomycete protein of unknown function (DUF875) superfamily	8	287.1	216.0	251.4	262.3	140.0	99.6	118.9	119.9
MSMEG _0954	MSMEG 0944	uroporphyrin-III C-methyltransferase/uroporphyrinogen-III synthase (h	6	447.5	482.2	534.5	535.2	298.2	317.0	266.5	265.2
MSMEG _0965	MSMEG 0955	mspA-Gen	4	460.7	497.0	298.3	398.1	496.8	533.9	433.3	551.1
MSMEG _0969	MSMEG 0959	glutamate-1-semialdehyde-2,1-aminomutase (hemL)	3	755.0	752.0	270.8	258.0	446.7	573.4	229.4	231.4
MSMEG _0970	MSMEG 0960	phosphoglycerate mutase family protein	1	213.5	204.5	184.7	187.9	136.4	126.7	107.0	105.2

MSMEG _0987	MSMEG 0977	hypothetical protein	1	38.7	0.0	39.8	49.8	114.4	0.0	121.1	110.3
MSMEG _1010	MSMEG 0998	transcriptional regulator, TetR family	3	269.5	283.3	244.7	289.4	444.2	480.3	378.5	413.0
MSMEG _1011	MSMEG 0999	oxidoreductase, short-chain dehydrogenase/reductase family	1	36.8	35.8	50.7	51.0	42.3	40.1	43.5	44.3
MSMEG _1019	MSMEG 1007	ribonucleoside-diphosphate reductase alpha chain (nrdE)	16	2251.5	2203.6	2320.3	2113.5	4569.1	4413.5	4763.6	4449.6
MSMEG _1021	MSMEG 1010	Glyoxalase/Bleomycin resistance protein/Dioxygenase family protein	1	39.6	39.0	56.5	59.5	39.9	42.9	45.8	48.0
MSMEG _1028	MSMEG 1015	probable electron transfer oxidoreductase, putative	7	2963.0	2868.2	2283.2	2360.4	2255.9	2362.1	1810.6	2022.2
MSMEG _1033	MSMEG 1019	ribonucleoside-diphosphate reductase, beta subunit	2	600.0	607.0	413.7	443.3	1210.9	1165.2	824.9	828.3
MSMEG _1037	MSMEG 1024	oxidoreductase, zinc-binding (adhA)	8	10758.9	11327.1	1547.6	1671.9	1518.3	1726.3	1145.6	1357.9
MSMEG _1038	MSMEG 1025	YCII-related domain superfamily	2	368.4	410.9	222.1	208.9	247.6	266.5	239.1	214.0
MSMEG _1041	MSMEG 1028	phosphoserine phosphatase (serB2)	1	118.8	153.6	40.6	63.8	81.8	96.1	41.5	73.7
MSMEG _1046	MSMEG 1033	ABC transporter, ATP-binding protein	5	607.4	617.0	625.6	622.7	960.7	904.8	984.6	1010.9

MSMEG _1047	MSMEG 1034	NUDIX domain protein	7	465.3	496.7	554.0	582.1	423.0	424.5	409.1	444.1
MSMEG _1048	MSMEG 1035	echA17 (echA17)	1	12.6	9.2	4.7	5.7	3.2	4.2	4.9	6.6
MSMEG _1049	MSMEG 1036	conserved hypothetical protein	2	133.3	110.6	121.3	106.1	28.1	26.7	18.9	32.9
MSMEG _1051	MSMEG 1038	immunogenic protein MPB64/MPT64	2	84.2	87.7	60.2	56.5	93.7	91.2	72.8	80.8
MSMEG _1052	MSMEG 1039	sodium:alanine symporter family protein	2	2.4	2.4	28.2	28.8	116.8	93.4	95.5	84.6
MSMEG _1060	MSMEG 1045	lysyl tRNA synthetase-like protein, putative	4	723.2	789.4	840.5	1007.8	1190.8	1317.0	823.1	940.4
MSMEG _1061	MSMEG 1046	Phosphohydrolase (MutT/nudixfamil)	3	227.4	347.8	162.4	176.4	233.2	370.4	162.7	190.1
MSMEG _1075	MSMEG 1059	naphthoate synthase (menB)	2	65.2	58.6	50.0	46.9	12.5	10.7	4.7	3.9
MSMEG _1076	MSMEG 1060	Protein hdeA precursor, putative	1	13.5	12.3	7.3	5.4	10.1	8.9	9.4	6.5
MSMEG _1077	MSMEG 1061	conserved hypothetical protein	1	50.1	54.5	34.4	38.8	72.2	77.8	62.7	65.6
MSMEG _1078	MSMEG 1062	hydrolase, haloacid dehalogenase-like family, putative	1	35.6	35.1	13.9	14.9	34.7	30.3	16.8	25.4
MSMEG _1090	MSMEG 1078	glutamyl-tRNA(Gln) amidotransferase, A subunit	1	14.6	21.5	17.5	25.2	82.5	115.1	97.0	134.0

MSMEG _1115	MSMEG 1105	ubiquinone/menaquinone biosynthesis methlytransferase (ubiE)	1	4.7	3.5	0.9	6.0	19.4	19.1	54.8	32.0
MSMEG _1133	MSMEG 1122	grcC1 (grcC1)	1	32.0	37.9	63.4	72.9	58.3	75.1	79.6	65.1
MSMEG _1134	MSMEG 1123	PROBABLE METALLOPROTEASE ZINC TRANSMEMBRANE PROTEIN	1	31.6	34.0	39.8	40.8	47.9	51.3	29.1	23.4
MSMEG _1143	MSMEG 1133	putative secreted protein	1	0.6	0.8	0.0	0.1	11.3	31.7	10.5	27.8
MSMEG _1165	MSMEG 1158	Protein of unknown function (DUF520) superfamily	6	210.1	180.1	170.0	188.3	354.3	309.3	363.4	380.4
MSMEG _1188	MSMEG 1181	hypothetical protein	1	66.1	57.5	47.0	48.8	61.4	53.5	56.8	51.8
MSMEG _1203	MSMEG 1198	methoxy mycolic acid synthase 1	1	79.8	73.6	104.0	90.9	134.0	129.0	136.5	151.6
MSMEG _1215	MSMEG 1208	probable serine/threonine protein kinase, putative	1	21.3	20.8	19.2	18.3	15.9	19.4	27.8	20.3
MSMEG _1225	MSMEG 1218	conserved hypothetical protein	1	50.3	29.0	17.6	13.6	176.7	153.3	143.7	150.9
MSMEG _1244	MSMEG 1236	hypothetical protein	5	33.6	30.4	39.8	42.0	46.6	39.5	33.6	35.6
MSMEG _1247	MSMEG 1240	hypothetical protein	3	57.7	66.5	159.6	177.3	422.4	419.7	494.4	501.6
MSMEG _1252	MSMEG 1245	hypothetical protein	1	4.8	3.9	2.3	3.5	49.0	48.1	19.6	23.1

MSMEG _1253	MSMEG 1247	conserved hypothetical protein	5	38.5	41.6	66.6	68.6	106.5	113.6	100.9	112.4
MSMEG _1254	MSMEG 1248	RNA helicase, putative	3	61.1	57.4	43.4	28.1	26.6	28.6	38.9	36.5
MSMEG _1255	MSMEG 1249	COG0210:Superfamily I DNA and RNA helicases	6	355.6	312.9	326.9	289.4	517.2	433.5	488.0	472.4
MSMEG _1265	MSMEG 1257	hypothetical protein	5	746.8	770.3	682.6	701.4	819.4	854.6	851.1	908.4
MSMEG _1285	MSMEG 1277	SEC-C motif domain protein	2	27.0	32.8	61.9	54.1	252.8	246.8	338.4	236.6
MSMEG _1292	MSMEG 1284	blr6160	1	12.0	11.0	5.0	5.1	20.8	20.1	23.3	15.9
MSMEG _1295	MSMEG 1287	transthyretin family protein	1	1.5	2.0	2.8	4.6	31.5	32.0	29.8	28.5
MSMEG _1296	MSMEG 1288	uricase	2	175.0	174.5	108.0	112.4	446.4	474.2	276.6	325.6
MSMEG _1331	MSMEG 1324	enoyl-CoA hydratase/isomerase family protein (fad-1)	1	8.6	6.9	12.0	9.3	7.4	5.9	10.6	8.2
MSMEG _1334	MSMEG 1327	metallo-beta-lactamase family protein	3	373.2	436.6	304.5	341.0	196.1	252.6	194.8	240.1
MSMEG _1340	MSMEG 1333	Actinomycete protein of unknown function (DUF875) superfamily	4	398.9	410.2	376.7	420.2	371.5	391.2	340.2	338.5
MSMEG _1341	MSMEG 1334	MaoC like domain, putative	3	805.1	980.9	854.9	935.8	830.4	1023.5	814.6	821.6
MSMEG _1342	MSMEG 1335	conserved hypothetical protein	2	117.6	108.8	118.2	117.0	60.4	55.3	52.6	50.1

MSMEG _1344	MSMEG 1337	preprotein translocase SecE subunit (secE)	1	39.0	50.8	62.6	65.7	15.3	20.9	10.6	13.9
MSMEG _1345	MSMEG 1338	transcription antitermination protein NusG CC3205	3	1578.6	1556.8	1429.2	1395.2	1207.5	1202.4	1299.7	1319.5
MSMEG _1346	MSMEG 1339	ribosomal protein L11 (rplK)	2	898.3	854.8	755.2	818.2	887.7	857.4	831.4	844.0
MSMEG _1347	MSMEG 1340	ribosomal protein L1 (rplA)	12	5653.8	5799.5	6845.4	6718.3	3103.5	3156.6	3636.0	3672.4
MSMEG _1350	MSMEG 1343	cyclopropane-fatty-acyl-phospholipid synthase 1	6	620.1	623.2	1023.2	988.5	333.1	339.4	306.3	326.3
MSMEG _1352	MSMEG 1345	carboxyl esterase	1	5.1	8.2	2.9	6.3	6.2	9.1	5.1	6.5
MSMEG _1364	MSMEG 1357	ribosomal protein L10 (rplJ)	8	2030.8	2153.6	2125.1	2324.0	1543.7	1555.7	1564.5	1558.3
MSMEG _1365	MSMEG 1358	ribosomal protein L7/L12 (rplL)	8	2361.9	2459.4	2425.9	2353.6	1793.1	1858.8	1777.9	1603.0
MSMEG _1367	MSMEG 1360	DNA polymerase (rpoB) (rpoB)	65	10898.8	10871.6	9413.9	9419.9	6709.2	6553.5	6175.8	5977.2
MSMEG _1368	MSMEG 1361	DNA-directed RNA polymerase, beta' subunit (rpoC)	92	12627.2	12407.2	10791.6	10347.5	7929.8	7804.8	8539.1	7951.0
MSMEG _1383	MSMEG 1376	endonuclease IV, putative	2	162.3	153.3	137.7	138.2	91.5	97.0	108.3	105.1
MSMEG _1384	MSMEG 1378	Protein of unknown function (DUF861) family	1	27.8	39.8	23.3	23.8	83.8	93.5	78.0	79.7
MSMEG _1393	MSMEG 1387	conserved hypothetical protein	1	12.2	18.0	14.5	18.5	179.7	203.0	194.3	169.7

MSMEG _1397	MSMEG 1392	transcriptional regulator, putative	1	3.5	6.1	2.1	3.2	85.9	85.6	63.6	56.4
MSMEG _1398	MSMEG 1393	ribosomal protein S12 (rpsL)	1	102.0	92.8	104.9	121.6	46.8	43.5	40.8	42.9
MSMEG _1399	MSMEG 1394	ribosomal protein S7 (rpsG)	11	6966.3	7674.3	6422.3	6712.5	3597.2	4047.4	3524.0	3437.6
MSMEG _1400	MSMEG 1395	translation elongation factor EF-G (fusA) (fusA)	5	833.1	678.9	720.7	601.5	1014.7	790.1	868.7	762.9
MSMEG _1401	MSMEG 1397	translation elongation factor Tu (tuf)	28	29767.6	29016.4	35032.6	34965.8	32108.9	31686.1	33537.3	33587.9
MSMEG _1416	MSMEG 1415	ferredoxin reductase	2	283.2	262.4	298.2	288.7	355.0	274.4	362.7	365.2
MSMEG _1430	MSMEG 1427	transcriptional regulatory protein, putative	1	4.4	3.4	1.7	1.4	7.2	8.6	4.0	4.8
MSMEG _1435	MSMEG 1433	ribosomal protein S10 (rpsJ)	4	1349.3	1431.0	1650.6	1694.9	862.1	885.8	979.3	922.2
MSMEG _1436	MSMEG 1434	ribosomal protein L3 (rplC)	9	2484.4	2118.7	3667.7	3440.1	1277.2	1035.2	1496.9	1417.7
MSMEG _1437	MSMEG 1435	ribosomal protein L4/L1 family (rplD)	4	1387.7	1071.1	1377.7	1400.8	776.1	567.9	736.2	748.4
MSMEG _1438	MSMEG 1436	ribosomal protein L23 (rplW)	2	1155.3	1180.7	1534.5	1588.6	767.5	814.0	841.2	805.7
MSMEG _1439	MSMEG 1437	ribosomal protein L2 (rplB)	7	1900.5	1757.9	1447.5	1424.6	1134.6	1057.9	1061.0	1075.3
MSMEG _1440	MSMEG 1438	ribosomal protein S19 (rpsS)	11	658.8	529.1	721.4	741.2	290.2	246.0	279.3	283.4
MSMEG _1441	MSMEG 1439	ribosomal protein L22	7	1873.5	2096.6	1919.5	1959.3	856.0	934.9	888.1	865.0
MSMEG _1442	MSMEG 1440	rpsC (rpsC)	7	2139.3	2223.0	1708.1	1686.9	1413.7	1636.5	1141.6	1171.1
MSMEG _1443	MSMEG 1441	ribosomal protein L16 (rplP)	5	3114.7	2770.5	3355.2	3190.8	2217.1	1889.5	2237.7	1965.1

MSMEG _1445	MSMEG 1443	rpsQ (rpsQ)	1	8.7	0.0	11.5	11.1	4.4	0.0	3.3	3.7
MSMEG _1465	MSMEG 1462	ribosomal protein L14 (rplN)	6	984.3	983.4	1190.1	1134.0	594.0	621.7	613.0	567.5
MSMEG _1466	MSMEG 1463	ribosomal protein L24 (rplX)	4	564.4	637.9	556.3	558.7	298.5	338.2	250.5	224.9
MSMEG _1467	MSMEG 1464	ribosomal protein L5 (rplE)	12	3293.3	3371.4	3847.1	3836.2	2541.9	2628.3	2507.0	2466.7
MSMEG _1469	MSMEG 1466	ribosomal protein S8 (rpsH)	3	896.2	947.0	851.0	909.1	489.7	503.3	468.9	462.4
MSMEG _1470	MSMEG 1467	ribosomal protein L6 (rplF)	7	3716.9	4025.7	4164.3	4337.2	2244.7	2341.6	2327.8	2280.6
MSMEG _1471	MSMEG 1468	ribosomal protein L18 (rplR)	2	211.6	190.4	230.6	238.9	142.0	129.1	147.4	146.8
MSMEG _1472	MSMEG 1469	ribosomal protein S5 (rpsE)	11	7926.4	7751.0	8363.6	7987.2	5055.9	4926.6	5165.6	5088.7
MSMEG _1473	MSMEG 1470	ribosomal protein L30 (rpmD)	3	797.1	857.2	796.5	833.1	421.5	456.5	507.6	491.6
MSMEG _1474	MSMEG 1471	ribosomal protein L15 (rplO)	6	2722.3	3096.4	2574.3	2746.4	2407.1	2747.2	2638.6	2625.1
MSMEG _1475	MSMEG 1472	Rif17, putative	2	215.3	214.9	43.5	53.3	71.0	65.7	68.5	77.6
MSMEG _1476	MSMEG 1473	signal peptide peptidase SppA, 67K type (sppA)	1	108.9	102.2	33.9	44.4	136.8	128.6	161.7	124.3
MSMEG _1479	MSMEG 1476	Mycobacterium tuberculosis paralogous family	2	94.1	114.2	33.5	39.0	61.7	72.7	38.4	64.0
MSMEG _1482	MSMEG 1479	Mycobacterium tuberculosis paralogous family	1	162.5	129.6	80.8	87.8	67.2	62.1	70.4	109.5
MSMEG _1485	MSMEG 1482	methionine aminopeptidase, type I (map)	4	92.5	108.6	117.3	100.4	28.8	30.1	50.7	52.1
MSMEG _1492	MSMEG 1490	transcriptional regulator, MarR family family	1	3.3	3.3	3.5	3.1	3.7	4.3	3.6	3.7

MSMEG _1498	MSMEG 1497	methylmalonate-semialdehyde dehydrogenase (mmsA)	2	283.2	304.2	303.9	301.5	241.1	315.8	294.1	285.3
MSMEG _1512	MSMEG 1511	dTDP-glucose 4,6-dehydratase (rfbB)	2	112.6	117.5	79.6	89.9	141.1	157.2	136.0	144.5
MSMEG _1513	MSMEG 1512	conserved hypothetical protein	7	497.2	505.6	584.5	615.9	802.4	767.0	1721.0	1555.5
MSMEG _1521	MSMEG 1520	ribosomal protein S13p/S18e (rpsM)	9	983.6	841.1	921.0	984.3	542.3	442.5	518.8	535.1
MSMEG _1522	MSMEG 1521	rpsK (rpsK)	6	2671.6	2365.3	2996.3	3015.5	1489.5	1231.5	1414.2	1328.1
MSMEG _1523	MSMEG 1522	ribosomal protein S4 (rpsD)	8	4836.3	4716.1	4910.1	5026.4	2778.6	2713.5	2566.2	2682.1
MSMEG _1524	MSMEG 1523	DNA-directed RNA polymerase, alpha subunit	18	13359.6	12108.1	12236.1	11869.3	9878.1	9281.2	9968.2	9716.3
MSMEG _1525	MSMEG 1524	ribosomal protein L17 (rplQ)	9	1930.4	1993.8	2303.5	2249.1	1014.1	1027.4	1262.5	1202.9
MSMEG _1528	MSMEG 1527	serine esterase, cutinase family	4	220.5	239.4	184.5	198.4	167.6	184.4	167.5	163.3
MSMEG _1536	MSMEG 1535	FtsK/SpoIIIE family protein	1	45.2	40.4	35.9	36.8	25.0	27.3	29.8	31.2
MSMEG _1556	MSMEG 1553	ribosomal protein L13 (rplM)	9	2980.6	2874.7	3061.2	3121.3	1778.0	1654.0	1687.2	1553.0
MSMEG _1557	MSMEG 1554	ribosomal protein S9 (rpsI)	9	7013.5	7462.0	7573.6	7664.4	3777.7	3833.8	4262.2	4143.8
MSMEG _1567	MSMEG 1564	conserved hypothetical protein	2	62.0	62.1	103.2	100.4	168.1	156.8	151.8	158.3
MSMEG _1574	MSMEG 1571	glutamate decarboxylase	7	584.1	570.1	430.4	403.8	739.7	711.1	747.9	681.1

MSMEG _1577	MSMEG 1574	Uncharacterised P-loop hydrolase UPF0079	1	49.4	56.1	22.1	35.2	49.5	60.2	24.9	39.6
MSMEG _1581	MSMEG 1579	hypothetical protein	1	12.3	13.4	9.7	10.5	6.4	9.9	4.3	5.4
MSMEG _1582	MSMEG 1580	chaperonin, 10 kDa (groES)	8	4198.2	4908.0	5099.3	5117.4	3016.9	3365.7	3704.4	3465.0
MSMEG _1583	MSMEG 1581	KCS	14	4682.2	4494.9	4481.3	4347.6	4232.6	4166.8	3723.3	3736.7
MSMEG _1602	MSMEG 1600	inosine-5-monophosphate dehydrogenase (guaB)	6	612.9	559.6	542.1	518.3	581.1	508.1	876.8	770.8
MSMEG _1603	MSMEG 1601	IMP dehydrogenase family protein	5	908.0	852.9	705.8	698.5	1156.9	1085.3	1377.8	1105.7
MSMEG _1605	MSMEG 1603	phosphate transport system regulatory protein, putative	1	0.1	0.1	4.5	5.4	0.4	0.3	0.7	0.7
MSMEG _1610	MSMEG 1608	GMP synthase (guaA)	7	1322.3	1168.8	1040.6	1035.7	1565.4	1480.3	1582.4	1767.3
MSMEG _1614	MSMEG 1612	amino acid ABC transporter, ATP-binding protein (glnQ)	1	76.4	83.8	80.4	84.7	2.0	1.0	1.0	1.9
MSMEG _1634	MSMEG 1633	conserved hypothetical protein	3	1352.7	1445.0	1911.4	1959.6	962.1	1030.3	980.0	958.4
MSMEG _1635	MSMEG 1634	nitroreductase family protein superfamily	9	2864.3	2830.6	1672.3	1670.1	1526.7	1433.0	1607.8	1564.4
MSMEG _1642	MSMEG 1641	ABC transporter, ATP-binding protein	9	275.3	283.5	254.8	281.3	209.2	216.0	173.5	201.8

MSMEG _1647	MSMEG 1645	methylenetetrahydrofolate dehydrogenase/methenyltetrahydr ofolate cycl	7	610.8	640.5	527.1	547.7	780.7	778.1	753.3	788.7
MSMEG _1650	MSMEG 1649	methyltransferase, putative	1	6.0	14.2	5.5	7.1	31.5	29.5	35.6	38.1
MSMEG _1652	MSMEG 1651	metC (metC)	8	2217.4	2304.7	2089.1	2239.6	1387.9	1452.6	1434.5	1487.9
MSMEG _1654	MSMEG 1652	isocitrate dehydrogenase, NADP-dependent	44	14265.5	14955.9	11008.7	10559.0	8088.0	8085.0	9068.2	8476.9
MSMEG _1655	MSMEG 1653	tropinesterase homolog tpeA	2	36.5	36.3	62.6	51.9	6.1	5.6	5.4	9.5
MSMEG _1656	MSMEG 1654	exodeoxyribonuclease III (xth)	1	21.7	18.1	31.2	22.0	23.2	30.0	18.0	21.4
MSMEG _1662	MSMEG 1660	aminotransferase, class III	5	396.7	323.6	597.7	553.1	513.3	456.0	555.1	581.0
MSMEG _1663	MSMEG 1661	regulatory protein AsnC, putative	2	128.2	130.1	127.1	107.6	72.7	72.9	47.1	50.2
MSMEG _1665	MSMEG 1662	aldehyde dehydrogenase family protein	1	291.2	284.9	234.4	207.1	167.1	209.1	248.1	227.8
MSMEG _1669	MSMEG 1666	sdhB (sdhB)	5	854.6	862.6	1527.6	1630.5	825.7	834.3	810.9	860.5
MSMEG _1670	MSMEG 1667	succinate dehydrogenase, flavoprotein subunit (sdhA)	8	1044.7	986.7	1679.9	1458.4	1278.6	1200.1	1055.8	953.9
MSMEG _1671	MSMEG 1668	succinate dehydrogenase, hydrophobic membrane anchor protein	1	9.2	5.8	19.8	23.3	5.1	2.1	4.2	7.2
MSMEG _1672	MSMEG 1669	sdhC (sdhC)	1	230.3	264.8	397.5	393.6	290.3	319.5	263.5	239.4

MSMEG _1676	MSMEG 1673	adenosine deaminase (add)	2	236.7	194.7	97.8	135.4	135.9	90.6	143.2	168.2
MSMEG _1679	MSMEG 1676	amiB (amiB)	3	161.7	184.4	387.2	364.4	476.1	518.3	786.6	783.6
MSMEG _1680	MSMEG 1677	conserved hypothetical protein	5	227.8	243.3	885.2	863.8	618.3	637.9	604.4	591.1
MSMEG _1681	MSMEG 1678	YER057c/YjgF/UK114 family protein	20	448.0	425.5	1378.7	1482.6	2084.2	1993.9	1505.1	1501.3
MSMEG _1682	MSMEG 1679	monooxygenase, putative	11	646.1	632.8	2722.7	2676.2	214.2	188.4	185.3	217.9
MSMEG _1683	MSMEG 1680	cytosine/purine/uracil/thiamine/allantoin permease family protein	1	0.0	0.0	0.8	0.5	34.4	43.3	37.1	31.7
MSMEG _1694	MSMEG 1690	uracil phosphoribosyltransferase	2	39.5	38.5	49.6	55.1	78.4	77.9	41.8	49.7
MSMEG _1696	MSMEG 1692	putative MarR-family transcriptional regulator	1	176.9	182.5	186.4	190.3	177.8	161.8	224.5	207.6
MSMEG _1701	MSMEG 1697	purine nucleotide phosphorylase	2	21.7	22.2	21.5	22.9	30.9	27.6	25.2	34.0
MSMEG _1704	MSMEG 1700	sugar ABC transporter, periplasmic sugar-binding protein	4	182.6	187.8	167.8	145.6	1088.7	1093.9	1031.4	1076.1
MSMEG _1712	MSMEG 1708	periplasmic ribose-binding protein	1	1.3	5.4	2.8	6.0	19.7	16.2	13.6	12.5
MSMEG _1713	MSMEG 1709	L-ribulokinase (araB)	1	18.2	24.0	19.4	25.9	28.7	32.7	14.8	18.1
MSMEG _1734	MSMEG 1730	conserved hypothetical protein	4	91.4	56.6	83.0	85.8	115.1	56.7	83.6	92.8

MSMEG _1735	MSMEG 1731	alpha keto acid dehydrogenase complex, E3 component, lipoamide dehydr	3	322.8	312.1	297.1	266.7	429.5	440.2	397.0	433.2
MSMEG _1753	MSMEG 1750	MTA/SAH nucleosidase	1	88.2	91.6	58.7	55.2	91.7	94.7	84.7	80.4
MSMEG _1757	MSMEG 1754	lhr (lhr)	1	1.6	2.3	2.4	3.6	14.0	14.8	12.7	14.0
MSMEG _1760	MSMEG 1758	bll7251	1	71.8	76.3	43.5	57.6	136.0	134.6	95.7	116.8
MSMEG _1803	MSMEG 1801	rsbW (rsbW)	3	343.9	393.1	289.5	305.8	449.8	537.8	458.9	438.2
MSMEG _1807	MSMEG 1807	biotin carboxylase/biotin carboxyl carrier protein	31	27068.7	27292.5	25172.7	26657.6	32691.1	32943.1	31227.7	32493.4
MSMEG _1808	MSMEG 1808	Fe-S metabolism associated domain subfamily (sufE)	1	81.1	91.4	79.6	73.2	109.6	133.6	63.3	76.1
MSMEG _1809	MSMEG 1809	thiosulfate sulfurtransferase (cysA-1)	12	2814.1	2713.0	1997.6	1840.6	2299.2	2149.3	1631.4	1572.7
MSMEG _1812	MSMEG 1811	conserved hypothetical protein	1	133.5	124.8	116.4	136.7	97.6	96.3	78.9	85.4
MSMEG _1813	MSMEG 1812	propionyl-CoA carboxylase, beta subunit (accD5)	12	4791.4	4857.2	4043.6	4160.0	5556.5	5490.3	4411.4	4577.8
MSMEG _1819	MSMEG 1818	phosphoribosylaminoimidazole carboxylase, ATPase subunit (purK)	2	207.2	159.5	109.7	119.0	132.2	131.1	249.3	244.7

MSMEG _1822	MSMEG 1821	biotin--acetyl-CoA-carboxylase ligase	1	29.8	30.2	25.0	22.6	38.8	39.1	30.2	35.1
MSMEG _1825	MSMEG 1824	dTDP-4-dehydrorhamnose reductase (rfbD)	1	121.3	119.6	135.5	130.5	57.3	60.6	109.2	105.7
MSMEG _1828	MSMEG 1826	rmlA2 (rmlA2)	2	208.0	166.8	122.9	95.5	80.0	87.2	74.8	76.4
MSMEG _1832	MSMEG 1831	conserved hypothetical protein	1	20.5	0.0	17.9	18.5	36.5	0.0	38.6	39.7
MSMEG _1834	MSMEG 1833	pmmA (pmmA)	1	10.9	9.4	5.8	5.4	5.3	5.6	5.0	4.6
MSMEG _1836	MSMEG 1835	mannose-6-phosphate isomerase, class I (manA)	1	139.3	142.2	105.6	97.9	105.8	131.8	90.7	94.0
MSMEG _1843	MSMEG 1842	adenosylhomocysteinase (ahcY)	11	3786.7	3450.0	5195.4	5273.6	3576.1	3349.7	3634.0	3976.3
MSMEG _1873	MSMEG 1876	Thymidylate kinase	1	65.8	68.7	42.4	48.7	117.0	119.7	65.4	89.7
MSMEG _1874	MSMEG 1877	DNA-binding response regulator MtrA (mtrA)	3	366.1	412.5	308.9	349.7	375.5	367.9	425.7	426.1
MSMEG _1878	MSMEG 1881	S30AE family protein	4	42.1	46.4	46.6	62.5	499.0	549.0	367.9	407.4
MSMEG _1881	MSMEG 1882	preprotein translocase, SecA subunit (secA)	18	2310.9	2296.2	2338.4	2344.1	2716.1	2848.1	2518.8	2194.3
MSMEG _1888	MSMEG 1890	Mycobacterium tuberculosis paralogous family	1	33.6	37.8	1.1	0.3	3.5	3.0	1.3	2.0

MSMEG _1890	MSMEG 1892	3-phosphoshikimate 1-carboxyvinyltransferase (aroA)	1	4.5	4.1	2.1	1.9	2.1	1.6	2.0	1.4
MSMEG _1903	MSMEG 1907	CAIB/BAIF family family	2	74.3	53.0	28.7	8.9	9.4	5.5	21.0	4.2
MSMEG _1913	MSMEG 1917	ybaK/ebsC protein	2	64.6	69.7	38.5	43.6	77.2	87.3	82.8	92.4
MSMEG _1914	MSMEG 1918	RNA polymerase sigma-70 factor, ECF subfamily	4	350.3	396.5	323.4	366.3	342.4	365.1	317.7	338.1
MSMEG _1926	MSMEG 1929	phosphoglycerate mutase family protein	1	40.8	32.4	37.5	22.7	65.7	68.7	65.8	71.2
MSMEG _1927	MSMEG 1930	Soj family protein (probable)	1	29.6	34.0	27.7	27.3	93.3	98.5	110.3	122.4
MSMEG _1934	MSMEG 1938	conserved hypothetical protein	1	168.1	190.8	138.3	153.1	254.9	261.5	250.7	246.8
MSMEG _1935	MSMEG 1939	transcriptional regulator, TetR family domain protein	1	100.5	117.8	74.2	97.9	116.3	128.4	62.9	97.4
MSMEG _1940	MSMEG 1944	hydrolase, alpha/beta hydrolase fold family	1	32.7	32.2	33.9	27.3	67.1	62.2	67.2	75.4
MSMEG _1951	MSMEG 1955	maebl, putative	2	9.1	12.7	31.4	37.8	141.9	151.7	136.8	151.5
MSMEG _1954	MSMEG 1958	ABC transporter, ATP-binding protein, putative	1	28.3	30.6	13.9	24.0	74.1	50.0	57.2	67.6
MSMEG _1959	MSMEG 1961	Uncharacterised protein family (UPF0182) superfamily	7	536.9	514.4	630.1	579.0	907.3	891.0	736.0	805.3

MSMEG _1979	MSMEG 1982	Uncharacterized ACR, YneC family COG1359 subfamily	1	17.6	35.0	10.4	16.2	161.0	320.5	94.6	138.8
MSMEG _1981	MSMEG 1984	conserved hypothetical protein	3	189.3	222.0	186.7	212.1	251.2	248.0	223.0	197.6
MSMEG _1996	MSMEG 1997	putative N5, N10-methylenetetrahydromethanopterin reductase-related p	9	543.3	549.9	295.1	346.7	490.2	482.6	454.0	484.6
MSMEG _2010	MSMEG 2010	conserved hypothetical protein	2	107.7	106.8	138.1	130.1	143.0	147.5	206.2	184.0
MSMEG _2019	MSMEG 2018	oxidoreductase, short chain dehydrogenase/reductase family	1	10.9	9.8	3.1	6.2	19.8	25.7	14.2	17.1
MSMEG _2025	MSMEG 2027	transcriptional regulator, putative	1	19.1	20.4	16.1	20.2	28.2	28.7	19.8	23.0
MSMEG _2026	MSMEG 2028	oxidoreductase, short-chain dehydrogenase/reductase family	8	2269.2	2296.1	1669.3	1643.4	1233.8	1191.6	1297.9	1332.8
MSMEG _2027	MSMEG 2029	conserved hypothetical protein	2	101.9	108.0	47.2	53.0	114.0	127.0	119.9	114.8
MSMEG _2033	MSMEG 2034	fadB4 (fadB4)	3	476.4	472.1	383.4	354.7	651.5	726.3	492.8	491.2
MSMEG _2045	MSMEG 2046	xenobiotic compound monooxygenase, DszA family	2	150.1	117.8	132.1	113.7	141.4	103.2	440.4	498.4
MSMEG _2078	MSMEG 2080	esterase, putative, antigen 85-B	4	598.3	633.1	1515.3	1434.4	1126.8	1141.8	1322.3	1259.4

MSMEG _2079	MSMEG 2081	alcohol dehydrogenase, zinc-containing	1	206.3	214.9	101.6	125.3	116.3	110.2	74.6	83.3
MSMEG _2080	MSMEG 2082	fadE23 (fadE23)	1	49.1	40.6	31.9	32.2	150.5	150.3	106.6	136.5
MSMEG _2081	MSMEG 2083	fadE24 (fadE24)	4	343.0	355.6	316.4	322.2	611.2	673.4	814.6	784.8
MSMEG _2089	MSMEG 2090	ftsE (ftsE)	1	48.4	32.4	31.7	43.8	29.8	25.8	19.7	39.7
MSMEG _2090	MSMEG 2091	ftsE protein, putative	1	12.3	7.7	11.3	10.3	4.7	2.1	2.6	2.9
MSMEG _2092	MSMEG 2093	D-aminopeptidase putative	2	97.3	66.7	102.1	102.6	149.7	145.0	188.3	185.9
MSMEG _2111	MSMEG 2114	chorismate mutase, putative, putative	1	30.4	23.4	25.9	30.2	31.5	24.9	26.4	30.9
MSMEG _2112	MSMEG 2115	putative secreted protein	2	16.5	14.4	7.9	9.6	53.0	38.2	53.7	53.5
MSMEG _2116	MSMEG 2119	Phosphotransferase system IIC components, glucose/maltose/N-acetylglu	2	1.9	2.3	9.7	11.8	17.4	18.8	28.4	29.2
MSMEG _2117	MSMEG 2120	PTS system, IIABC components (EC2.7.1.69)	2	143.1	128.2	94.2	89.4	210.3	205.7	191.8	178.6
MSMEG _2118	MSMEG 2121	glucosamine-6-phosphate isomerase (nagB)	1	73.6	72.9	59.3	49.5	84.8	88.4	85.1	84.7
MSMEG _2136	MSMEG 2139	phosphoglucomutase, alpha-D-glucose phosphate-specific (pgm)	3	172.2	136.0	156.9	143.6	259.9	242.1	327.2	321.3

MSMEG _2155	MSMEG 2157	NADPH quinone oxidoreductase, putative	1	1.6	2.7	3.7	3.9	34.2	24.8	34.8	29.5
MSMEG _2171	MSMEG 2175	CAIB/BAIF family protein	1	0.2	0.3	0.1	0.2	5.0	5.0	0.9	1.5
MSMEG _2174	MSMEG 2178	COG3973:Superfamily I DNA and RNA helicases	3	215.8	186.7	138.4	139.3	59.0	56.9	68.4	56.3
MSMEG _2198	MSMEG 2203	HesA/MoeB/ThiF family protein (moeY)	7	519.2	542.7	495.6	506.0	1014.4	1028.5	1131.9	1105.2
MSMEG _2201	MSMEG 2206	MaoC family protein	8	1317.6	1419.7	1404.6	1549.8	1963.3	2081.8	1851.5	1895.6
MSMEG _2225	MSMEG 2230	transcriptional regulator, TetR family, putative	2	44.1	40.8	49.9	61.7	104.6	105.2	85.8	106.0
MSMEG _2263	MSMEG 2266	F420-nonreducing hydrogenase, putative	1	76.7	86.5	249.3	239.3	62.2	69.4	106.7	99.9
MSMEG _2271	MSMEG 2274	hydrogenase accessory protein HypB (hypB)	1	27.0	35.3	61.5	66.6	109.9	152.2	64.8	58.7
MSMEG _2280	MSMEG 2283	pyruvate dehydrogenase	5	145.9	137.8	228.0	170.0	415.9	396.6	297.4	293.4
MSMEG _2351	MSMEG 2350	electron transfer flavoprotein, beta subunit (etfB)	10	4118.4	4359.8	4330.1	4323.6	3104.9	3314.1	3660.5	3753.1
MSMEG _2352	MSMEG 2351	electron transfer flavoprotein, alpha subunit (etfA)	14	12624.2	13306.3	10985.1	11299.6	12476.7	12353.4	9224.6	9681.0
MSMEG _2362	MSMEG 2361	DNA ligase, NAD-dependent (ligA)	9	768.6	724.8	867.7	747.3	714.0	726.9	725.7	718.1

MSMEG _2365	MSMEG 2364	glutamyl-tRNA(Gln) amidotransferase subunit A (gatA)	4	597.2	593.8	375.5	374.0	527.2	594.3	798.0	804.8
MSMEG _2366	MSMEG 2365	Phosphofructokinase	1	62.0	66.7	36.5	38.8	466.1	504.4	335.6	387.0
MSMEG _2367	MSMEG 2366	glutamyl-tRNA(Gln) amidotransferase, B subunit (gatB)	4	555.1	509.5	547.9	486.6	330.4	306.0	476.6	390.4
MSMEG _2372	MSMEG 2371	acetolactate synthase, large subunit, biosynthetic type (ilvB)	2	104.6	93.6	113.7	86.6	47.9	37.8	68.2	58.5
MSMEG _2373	MSMEG 2372	acetolactate synthase, small subunit (ilvN)	5	190.3	170.7	173.0	170.4	34.0	27.5	19.1	22.0
MSMEG _2374	MSMEG 2373	ketol-acid reductoisomerase (ilvC)	16	4212.1	3415.9	4885.9	4546.4	3695.0	3132.8	4371.0	4339.8
MSMEG _2375	MSMEG 2374	tryptophan repressor binding protein (wrbA)	1	91.3	94.0	70.4	231.7	107.3	105.9	108.8	126.3
MSMEG _2378	MSMEG 2377	D-3-phosphoglycerate dehydrogenase (serA)	10	2109.1	1871.5	2164.8	2050.8	2845.0	2485.2	3189.9	2921.8
MSMEG _2379	MSMEG 2378	3-isopropylmalate dehydrogenase (leuB)	4	1340.9	1366.2	832.9	771.1	1041.3	1073.5	951.3	923.7
MSMEG _2382	MSMEG 2381	2-hydroxyhepta-2,4-diene-1,7-dioate isomerase/5-carboxymethyl 2-oxo-h	4	344.4	363.5	315.3	345.2	365.9	374.8	314.7	413.8

MSMEG _2383	MSMEG 2382	glutamyl-tRNA synthetase (gltX)	5	630.7	597.5	676.1	604.2	697.7	688.6	954.8	893.6
MSMEG _2387	MSMEG 2386	3-isopropylmalate dehydratase, large subunit (leuC)	1	98.0	132.2	55.9	50.8	108.9	129.7	84.0	89.2
MSMEG _2388	MSMEG 2387	3-isopropylmalate dehydratase, small subunit (leuD)	8	3016.7	3233.8	2678.8	2776.0	2830.1	2929.8	2711.8	2885.2
MSMEG _2389	MSMEG 2388	histone-like protein	11	2725.2	2647.1	4589.1	4069.9	4321.5	4001.2	5727.8	5105.6
MSMEG _2391	MSMEG 2390	polyphosphate kinase (ppk)	13	1726.7	1741.3	1374.8	1385.3	2566.7	2584.8	2633.6	2637.9
MSMEG _2397	MSMEG 2396	transcriptional regulator, AsnC family, putative	1	28.9	28.5	11.9	12.9	31.5	38.9	39.3	29.4
MSMEG _2399	MSMEG 2398	uracil-DNA glycosylase (ung)	1	88.7	78.1	79.1	77.1	58.9	49.9	54.0	49.8
MSMEG _2407	MSMEG 2405	oxidoreductase, aldo/keto reductase family	8	876.4	795.4	914.7	960.0	663.9	573.2	654.2	679.5
MSMEG _2408	MSMEG 2406	oxidoreductase, aldo/keto reductase family	7	1580.9	1677.0	649.5	746.1	663.9	695.0	642.2	728.9
MSMEG _2410	MSMEG 2408	u0002a	4	311.4	351.0	255.6	308.7	199.3	214.7	171.2	219.5
MSMEG _2412	MSMEG 2410	pyruvate carboxylase (pyc)	42	4319.5	4219.8	3929.2	3870.8	7325.7	7347.2	6443.9	6230.5
MSMEG _2413	MSMEG 2411	methyltransferase, putative	1	22.0	26.8	14.1	13.2	27.7	32.7	19.5	21.6
MSMEG _2414	MSMEG 2412	pantetheine-phosphate adenylyltransferase (coaD)	3	275.4	278.8	233.8	231.9	400.7	417.4	355.7	345.7

MSMEG _2415	MSMEG 2413	conserved hypothetical protein	1	4.7	4.9	2.6	4.7	172.6	161.9	245.4	209.8
MSMEG _2418	MSMEG 2416	ribonuclease III (rnc)	3	115.2	121.9	85.4	79.3	214.6	238.1	213.4	244.4
MSMEG _2421	MSMEG 2420	conserved hypothetical protein	5	161.6	121.3	120.2	132.5	151.2	82.2	149.2	154.2
MSMEG _2423	MSMEG 2422	chromosome partition protein SMC, putative	1	15.4	15.9	14.6	16.0	19.1	19.4	19.2	18.3
MSMEG _2424	MSMEG 2423	signal recognition particle receptor FtsY (ftsY)	2	163.8	185.6	167.2	194.1	135.0	155.7	100.4	180.0
MSMEG _2426	MSMEG 2425	Nitrogen regulatory protein P-II	11	154.0	157.2	138.5	148.9	3182.0	3381.9	3861.3	3603.8
MSMEG _2427	MSMEG 2426	protein-P-II uridylyltransferase (glnD)	11	40.8	48.0	34.8	42.8	1558.7	1534.8	1792.5	1920.2
MSMEG _2435	MSMEG 2433	ribosomal protein S16 (rpsP)	9	4271.6	4436.9	5028.2	5536.0	3100.8	3221.2	3503.8	3678.0
MSMEG _2437	MSMEG 2435	16S rRNA processing protein RimM VC0562	1	10.3	11.9	10.7	13.5	17.0	17.9	12.9	12.4
MSMEG _2440	MSMEG 2438	50S ribosomal protein L19 (rplS)	4	1147.6	1328.3	1401.8	1302.4	605.7	688.5	609.8	532.7
MSMEG _2443	MSMEG 2441	conserved hypothetical protein	4	123.0	136.6	124.0	125.0	116.2	124.9	114.5	99.8
MSMEG _2450	MSMEG 2446	4-aminobutyrate aminotransferase (goaG)	1	23.2	19.8	26.3	23.8	2.2	2.6	1.5	1.8
MSMEG _2468	MSMEG 2467	CAIB/BAIF family protein, putative	1	11.8	12.0	8.6	10.3	7.6	9.0	3.8	5.8

MSMEG _2511	MSMEG 2512	siderophore utilization protein (viuB)	2	391.6	424.7	260.8	283.5	166.7	177.0	146.5	150.9
MSMEG _2516	MSMEG 2520	Luciferase-like monooxygenase superfamily	4	322.7	340.3	145.5	150.1	195.7	220.3	206.5	204.4
MSMEG _2519	MSMEG 2522	ribosomal protein S2 (rpsB)	6	1155.7	1087.1	847.1	823.2	997.7	1128.8	508.4	615.2
MSMEG _2524	MSMEG 2527	ABC transporter, ATP-binding protein homolog	3	3.1	3.2	4.2	3.5	160.4	174.4	203.4	196.4
MSMEG _2525	MSMEG 2528	amino acid permease family protein, putative	1	25.9	33.8	20.5	27.6	472.9	452.2	483.0	466.9
MSMEG _2526	MSMEG 2529	amine oxidase	20	292.3	288.9	277.0	240.8	3106.7	2945.1	3605.6	3377.1
MSMEG _2528	MSMEG 2531	glycerate kinase	3	280.0	250.6	158.9	144.4	728.3	668.7	535.2	527.4
MSMEG _2540	MSMEG 2543	uridylate kinase	3	210.0	213.2	195.2	194.4	254.9	268.7	198.3	197.1
MSMEG _2541	MSMEG 2544	ribosome recycling factor (frr)	3	65.6	70.6	86.6	82.6	170.4	146.0	181.3	188.5
MSMEG _2553	MSMEG 2555	transcriptional regulator, TetR family domain protein	1	10.2	10.1	6.2	6.7	9.4	11.7	3.4	5.7
MSMEG _2580	MSMEG 2581	1-hydroxy-2-methyl-2-(E)-butenyl 4-diphosphate synthase (ispG)	3	551.0	539.9	366.6	393.2	421.3	452.4	331.8	375.4
MSMEG _2587	MSMEG 2588	methionine aminopeptidase, type I (map)	1	36.4	37.8	13.0	23.4	5.7	6.4	4.8	5.2

MSMEG _2594	MSMEG 2595	asparagine synthase (glutamine-hydrolyzing)	3	345.5	346.8	304.0	343.6	47.0	55.4	25.8	24.3
MSMEG _2598	MSMEG 2599	oxidoreductase, short-chain dehydrogenase/reductase family	2	86.0	87.0	113.8	138.1	358.1	370.6	322.2	376.2
MSMEG _2606	MSMEG 2607	transcriptional regulator, ArsR family (smtB)	1	8.1	9.6	11.2	9.8	26.4	22.0	21.5	21.3
MSMEG _2613	MSMEG 2614	malate:quinone-oxidoreductase (mqo)	5	408.6	425.5	567.0	501.5	238.8	222.0	284.0	254.2
MSMEG _2616	MSMEG 2617	cob(I)alamin adenosyltransferase (cobO)	1	37.7	37.0	39.1	34.9	54.0	52.0	49.8	54.7
MSMEG _2621	MSMEG 2622	prolyl-tRNA synthetase (proS)	19	2122.7	2107.7	1843.7	1719.9	2535.0	2552.4	2656.2	2769.3
MSMEG _2628	MSMEG 2627	translation initiation factor IF-2 (infB)	20	2250.1	2134.9	2007.8	1826.5	1879.2	1774.3	1827.8	1753.5
MSMEG _2641	MSMEG 2640	EchA16_2 (fad)	4	686.3	711.9	466.1	484.5	470.3	471.9	523.8	526.3
MSMEG _2653	MSMEG 2652	riboflavin biosynthesis protein RibF (ribF)	1	13.6	13.2	12.5	17.5	15.5	15.6	10.9	10.8
MSMEG _2654	MSMEG 2653	ribosomal protein S15 (rpsO)	8	2556.8	2424.1	2763.4	2779.7	1155.4	1117.5	1333.8	1316.9
MSMEG _2656	MSMEG 2655	polyribonucleotide nucleotidyltransferase VC0647	32	8207.5	7959.7	7920.5	7377.8	6056.4	5841.6	7674.9	7217.5
MSMEG _2658	MSMEG 2657	beta-lactamase	3	106.8	120.1	57.5	83.5	23.0	31.2	22.7	34.3

MSMEG _2664	MSMEG 2662	dihydrodipicolinate reductase (dapB)	2	87.9	87.5	81.2	79.9	87.5	87.1	103.1	98.0
MSMEG _2667	MSMEG 2666	conserved hypothetical protein	4	292.2	273.4	160.4	150.8	495.0	535.5	380.1	422.0
MSMEG _2669	MSMEG 2667	dienelactone hydrolase family protein	5	1576.9	1515.3	362.9	413.8	583.8	545.5	521.7	587.2
MSMEG _2671	MSMEG 2669	dihydrofolate reductase (folA)	1	37.5	38.5	21.7	26.4	62.0	58.4	51.6	64.4
MSMEG _2684	MSMEG 2682	dihydrodipicolinate synthase (dapA)	3	208.2	217.3	243.0	232.9	1135.4	1233.4	1438.1	1440.4
MSMEG _2688	MSMEG 2686	Uncharacterized ACR, YneC family COG1359 subfamily	1	58.5	57.4	23.2	29.7	26.0	25.7	22.7	29.1
MSMEG _2698	MSMEG 2697	conserved hypothetical protein	4	238.3	228.5	203.2	207.6	215.2	206.3	180.9	166.6
MSMEG _2723	MSMEG 2725	recA protein (recA)	1	16.0	8.4	2.5	2.6	2.7	0.5	0.2	0.4
MSMEG _2727	MSMEG 2729	glnH, putative	11	1208.2	1278.3	2126.9	2148.0	5105.6	5717.8	4873.5	5160.4
MSMEG _2731	MSMEG 2734	Domain of Unknown Function (DUF349) family	5	1045.3	1189.9	1033.2	1035.5	806.8	981.5	716.2	815.8
MSMEG _2739	MSMEG 2742	conserved hypothetical protein	2	42.5	43.4	56.3	61.7	109.2	101.6	99.4	97.8
MSMEG _2740	MSMEG 2743	LexA repressor (lexA)	1	14.6	12.3	17.3	16.2	52.8	27.4	19.6	21.6

MSMEG_2743	MSMEG 2745	conserved hypothetical protein TIGR00244	1	25.9	24.5	21.2	24.1	35.6	32.1	35.2	27.7
MSMEG_2744	MSMEG 2746	phenazine biosynthesis protein, PhzF family, putative	3	315.2	333.2	230.2	219.2	310.6	320.5	364.6	389.5
MSMEG_2750	MSMEG 2753	siderophore biosynthesis iron binding repressor (dtxR)	1	79.6	85.3	110.7	93.5	171.7	184.0	248.6	239.9
MSMEG_2758	MSMEG 2759	RNA polymerase sigma factor MysA (mysA)	9	810.8	781.6	735.7	659.2	585.5	531.8	526.8	472.8
MSMEG_2760	MSMEG 2760	polyphosphate glucokinase	3	130.4	148.1	180.6	194.0	474.5	500.5	506.2	551.0
MSMEG_2762	MSMEG 2761	inositol monophosphatase family protein (suhB)	8	486.6	489.1	440.9	450.9	443.7	449.2	516.7	569.9
MSMEG_2763	MSMEG 2763	conserved hypothetical protein	1	21.2	22.3	35.8	38.2	18.4	19.2	18.4	17.4
MSMEG_2765	MSMEG 2764	deoxyuridine 5-triphosphate nucleotidohydrolase (dut)	2	326.2	336.2	268.4	284.4	223.6	269.3	216.9	247.8
MSMEG_2766	MSMEG 2765	hypothetical protein	3	185.5	265.0	124.7	175.9	231.1	341.0	160.2	236.7
MSMEG_2769	MSMEG 2769	ceoB protein (trkA)	1	21.1	25.2	13.6	17.9	23.3	27.2	15.0	23.1
MSMEG_2771	MSMEG 2770	ceoB protein	1	93.6	96.7	95.0	110.8	273.2	285.4	189.3	237.6

MSMEG _2776	MSMEG 2775	1-deoxy-D-xylulose-5-phosphate synthase (dxs)	1	12.4	14.1	13.0	9.1	1.2	1.0	2.8	2.6
MSMEG _2780	MSMEG 2780	uroporphyrinogen decarboxylase (hemE)	1	50.0	54.4	16.2	24.8	51.1	44.7	85.2	88.5
MSMEG _2782	MSMEG 2782	conserved hypothetical protein	4	368.5	347.7	190.8	187.3	150.3	143.6	130.4	127.8
MSMEG _2784	MSMEG 2785	methionine-R-sulfoxide reductase (msrB)	2	28.0	18.8	33.8	38.6	49.5	38.4	59.7	55.4
MSMEG _2787	MSMEG 2788	RibD C-terminal domain	1	54.6	61.5	49.4	55.4	62.9	58.4	72.3	73.7
MSMEG _2788	MSMEG 2789	AFG1-like ATPase superfamily	1	44.0	54.5	40.4	50.8	149.5	157.3	136.7	127.6
MSMEG _2789	MSMEG 2790	acetyltransferase, GNAT family	1	16.4	0.0	7.6	8.6	12.0	0.0	9.0	14.1
MSMEG _2800	MSMEG 2802	NADH-dependent FMN reductase, putative	1	90.8	98.0	82.3	103.0	101.0	112.8	108.8	116.5
MSMEG _2839	MSMEG 2841	Predicted RNA binding protein, contains S1 domain	12	762.0	746.6	894.0	938.3	580.1	638.8	422.2	562.0
MSMEG _2897	MSMEG 2903	conserved hypothetical protein	2	19.0	20.6	13.5	11.5	15.0	17.5	22.2	19.3
MSMEG _2900	MSMEG 2908	hydrolase, alpha/beta fold family domain protein	1	17.6	14.0	16.0	14.0	11.4	13.0	3.4	3.8
MSMEG _2931	MSMEG 2941	threonyl-tRNA synthetase (thrS)	8	887.3	871.5	714.4	695.6	874.2	883.8	968.5	952.0

MSMEG _2940	MSMEG 2951	conserved hypothetical protein TIGR01033	2	381.9	374.3	319.7	331.6	141.1	126.5	75.3	93.6
MSMEG _2941	MSMEG 2952	conserved hypothetical protein	2	132.7	150.7	117.9	123.5	160.2	191.5	192.1	197.1
MSMEG _2942	MSMEG 2953	glyoxalase family protein superfamily	1	27.4	0.0	16.6	14.8	152.3	0.0	156.3	137.4
MSMEG _2956	MSMEG 2165	substrate--CoA ligase, putative	19	965.0	908.2	80.7	97.1	751.6	695.9	641.0	563.7
MSMEG _2960	MSMEG 2972	Preprotein translocase subunit, putative	3	170.9	225.2	125.0	168.4	155.4	205.9	106.2	152.8
MSMEG _2961	MSMEG 2973	protein-export membrane protein SecD (secD)	1	96.4	122.5	105.4	123.2	63.0	83.1	65.6	75.4
MSMEG _2964	MSMEG 2976	adenine phosphoribosyltransferase	3	55.1	44.3	26.3	27.3	21.4	20.6	13.6	15.1
MSMEG _2974	MSMEG 2987	peptidyl-prolyl cis-trans isomerase, cyclophilin-type	1	144.6	195.2	110.1	112.6	140.2	218.1	94.5	102.9
MSMEG _2975	MSMEG 2988	1 histidyl-tRNA synthetase (hisS)	3	354.7	354.6	267.3	255.5	312.5	297.4	379.7	381.8
MSMEG _2978	MSMEG 2990	branched-chain amino acid ABC transporter, ATP-binding protein (livF)	1	0.1	0.0	0.0	0.0	26.6	30.3	8.9	19.5
MSMEG _2982	MSMEG 2994	urea/short-chain amide ABC transporter, periplasmic urea/short-chain	9	438.1	524.3	313.2	348.1	3646.3	3677.7	3843.3	3937.1

MSMEG _2983	MSMEG 2995	conserved hypothetical protein	1	16.9	18.9	17.0	17.4	7.8	8.4	12.3	8.5
MSMEG _3003	MSMEG 3017	aspartyl-tRNA synthetase (aspS)	7	1626.8	1681.4	1147.2	1208.5	2360.2	2240.0	1571.5	1824.2
MSMEG _3004	MSMEG 3018	conserved hypothetical protein	1	22.5	24.9	12.5	16.2	13.1	14.8	7.0	8.8
MSMEG _3013	MSMEG 3027	Transcriptional regulator family	1	10.5	10.6	5.0	9.5	6.9	9.0	9.9	9.7
MSMEG _3016	MSMEG 3030	transglutaminase family protein	11	42.1	41.4	54.1	61.2	265.8	241.6	241.7	250.1
MSMEG _3019	MSMEG 3033	conserved hypothetical protein	1	26.4	31.5	24.1	22.4	62.3	67.7	59.1	54.9
MSMEG _3025	MSMEG 3039	alanyl-tRNA synthetase (alaS)	16	2115.6	2189.7	2480.7	2522.1	1221.1	1280.1	1284.6	1365.9
MSMEG _3026	MSMEG 3040	DNA integration/recombination/invertio n protein	1	9.3	8.7	7.0	6.3	19.7	18.9	14.7	18.4
MSMEG _3033	MSMEG 3046	3-dehydroquinate synthase (aroB)	2	161.9	160.0	104.9	116.5	242.1	285.7	161.4	169.8
MSMEG _3035	MSMEG 3049	translation elongation factor P (efp)	1	204.7	223.0	181.7	201.2	179.4	185.6	149.5	172.0
MSMEG _3045	MSMEG 3059	conserved hypothetical protein	1	18.3	18.2	20.0	31.5	25.7	26.2	11.9	19.5
MSMEG _3046	MSMEG 3060	carbamoyl-phosphate synthase, small subunit (carA)	2	453.0	547.8	392.3	493.9	327.8	354.6	324.3	349.4

MSMEG _3047	MSMEG 3061	carbamoyl-phosphate synthase, large subunit (carB)	34	1735.4	1694.8	1356.1	1368.6	2569.5	2452.5	2343.8	2287.9
MSMEG _3050	MSMEG 3063	integration host factor (mIHF)	4	684.6	708.2	771.6	757.7	588.4	607.5	690.5	644.0
MSMEG _3054	MSMEG 3065	phosphopantothenoylcysteine decarboxylase/phosphopantothen ate--cystei	1	75.3	76.7	65.4	69.1	107.1	82.4	94.4	113.3
MSMEG _3055	MSMEG 3066	S-adenosylmethionine synthetase (metK)	22	15934.0	14837.9	12951.0	11986.5	8464.1	8083.1	9320.3	8848.9
MSMEG _3058	MSMEG 3069	D-methionine-binding lipoprotein MetQ	4	186.8	192.4	595.0	541.1	279.4	281.2	268.2	255.1
MSMEG _3066	MSMEG 3077	ribulose-phosphate 3-epimerase (rpe)	1	76.2	78.3	66.1	66.3	112.7	120.0	104.9	114.6
MSMEG _3070	MSMEG 3080	lipoprotein, 27 kDa	4	723.2	733.9	484.6	497.3	787.2	769.2	682.8	651.2
MSMEG _3071	MSMEG 3081	riboflavin synthase, alpha subunit (ribE)	3	125.4	115.2	101.6	94.4	92.1	71.0	57.0	60.2
MSMEG _3072	MSMEG 3082	3,4-dihydrox	5	472.6	462.6	420.5	425.5	221.1	263.6	196.8	245.2
MSMEG _3073	MSMEG 3083	6,7-dimethyl-8-ribityllumazine synthase (ribE)	6	614.3	644.5	405.5	449.5	756.3	784.0	633.5	660.4
MSMEG _3081	MSMEG 3090	Uncharacterized BCR, COG1481	2	216.5	213.5	117.8	115.3	316.8	403.0	199.3	200.8
MSMEG _3084	MSMEG 3093	glyceraldehyde-3-phosphate dehydrogenase, type I (gap)	20	23839.1	23167.3	17589.7	18172.6	30114.8	28999.0	26785.4	27859.8

MSMEG _3085	MSMEG 3094	phosphoglycerate kinase (pgk)	17	7334.3	7169.7	6312.7	6158.5	7285.9	7090.7	7836.6	7385.0
MSMEG _3086	MSMEG 3095	triosephosphate isomerase (tpiA)	14	2053.3	2140.4	2092.5	2038.8	2594.7	2613.9	2678.3	2592.3
MSMEG _3097	MSMEG 3107	phosphoenolpyruvate carboxylase (ppc)	7	325.4	349.2	300.2	380.8	534.4	513.1	639.2	622.1
MSMEG _3099	MSMEG 3109	6-phosphogluconolactonase (pgl)	3	308.3	283.2	257.9	257.7	1203.9	1182.3	1358.1	1333.7
MSMEG _3100	MSMEG 3110	opcA protein (opcA)	1	126.3	137.6	66.8	81.2	214.9	209.4	78.9	101.4
MSMEG _3101	MSMEG 3111	glucose-6-phosphate 1-dehydrogenase (zwf)	3	207.8	186.7	212.6	204.2	152.7	175.5	159.3	176.7
MSMEG _3102	MSMEG 3112	transaldolase (tal)	11	4289.7	4076.3	4154.3	3744.0	2872.4	2764.4	3089.9	2850.9
MSMEG _3103	MSMEG 3113	transketolase (tkt)	35	11537.0	11624.4	9263.4	9301.0	11329.9	11298.6	10124.2	10212.8
MSMEG _3106	MSMEG 3115	quinone oxidoreductase CC3759	7	2993.3	2911.5	1338.9	1384.7	3030.7	2897.0	2177.0	2258.1
MSMEG _3122	MSMEG 3131	Pps1 sans inteine	1	68.2	58.0	51.6	61.3	6.1	2.6	5.0	4.7
MSMEG _3124	MSMEG 3133	MW07	3	301.6	293.7	368.0	335.5	170.1	195.2	226.7	230.9
MSMEG _3127	MSMEG 3136	mrp protein homolog	2	60.8	59.5	64.3	65.7	37.8	34.7	42.7	45.5
MSMEG _3142	MSMEG 3149	transcriptional regulator, TetR family domain protein	1	14.2	13.0	5.3	3.7	14.6	16.2	17.1	15.3
MSMEG _3143	MSMEG 3151	acn (acn)	20	9235.6	10268.2	4569.6	4720.7	4895.7	5198.7	4810.3	4951.8
MSMEG _3147	MSMEG 3155	ATPase, MoxR family (moxR)	4	1468.7	1282.4	1173.9	1066.1	1265.4	1088.9	1384.7	1361.0

MSMEG _3150	MSMEG 3158	hypothetical protein	6	1907.2	1980.4	1893.1	1764.7	1930.0	1917.9	1869.2	1771.3
MSMEG _3151	MSMEG 3159	enoyl-(acyl-carrier-protein) reductase (NADH)	7	1865.1	1864.3	2213.9	2049.5	1452.6	1487.2	1861.7	1674.8
MSMEG _3155	MSMEG 3163	Membrane protease subunits, stomatin/prohibitin homologs	2	75.8	64.3	99.3	94.9	192.4	230.9	157.1	145.7
MSMEG _3158	MSMEG 3166	methylmalonyl-CoA mutase, small subunit (mutA)	7	342.7	354.8	842.8	764.9	959.6	833.6	752.1	711.4
MSMEG _3159	MSMEG 3167	Methylmalonyl-CoA mutase, N-terminal domain/subunit (mutA)	18	1513.7	1663.6	2696.4	2775.3	2870.4	2935.1	2349.7	2411.6
MSMEG _3160	MSMEG 3168	LAO/AO transport system ATPase	1	3.8	3.3	5.4	4.4	1.6	2.4	2.7	1.8
MSMEG _3169	MSMEG 3176	isoleucyl-tRNA synthetase (ileS)	15	605.9	642.9	540.6	574.8	534.1	580.8	430.7	517.4
MSMEG _3178	MSMEG 3185	DNA polymerase III, alpha subunit (dnaE)	3	46.5	44.1	32.9	36.8	44.7	42.1	72.9	66.5
MSMEG _3183	MSMEG 3190	threonine dehydratase, biosynthetic (ilvA)	2	303.5	286.3	218.1	250.4	264.8	288.3	173.3	243.5
MSMEG _3185	MSMEG 3193	glycosyl hydrolase, family 13 (treY)	2	32.3	39.2	32.3	21.9	18.5	22.3	32.0	20.6
MSMEG _3204	MSMEG 3214	conserved hypothetical protein	3	11.2	13.5	7.6	11.6	32.0	31.2	25.9	33.9

MSMEG _3205	MSMEG 3215	histidinol dehydrogenase (hisD)	1	40.8	33.9	53.1	41.8	30.6	28.2	57.2	48.0
MSMEG _3208	MSMEG 3218	imidazole glycerol phosphate synthase, glutamine amidotransferase sub	1	28.4	38.9	18.6	21.0	39.2	30.0	44.1	45.5
MSMEG _3209	MSMEG 3219	bifunctional HisA/TrpF protein	6	455.6	488.8	457.0	468.3	314.1	339.8	304.5	346.7
MSMEG _3210	MSMEG 3220	inositol monophosphatase family protein	1	14.0	15.0	8.4	9.3	24.0	33.1	20.1	30.9
MSMEG _3211	MSMEG 3221	imidazoleglycerol phosphate synthase, cyclase subunit (hisF)	1	9.8	9.4	8.4	11.5	8.8	10.6	7.9	11.7
MSMEG _3215	MSMEG 3225	ABC transporter, ATP-binding protein	2	392.2	416.4	178.8	186.9	161.4	175.4	222.2	206.9
MSMEG _3219	MSMEG 3229	indole-3-glycerol phosphate synthase (trpC)	5	386.5	422.3	504.7	504.9	51.0	64.8	49.1	68.3
MSMEG _3220	MSMEG 3230	tryptophan synthase, beta subunit (trpB)	3	55.4	53.4	40.2	54.7	138.0	145.0	182.2	195.2
MSMEG _3221	MSMEG 3231	tryptophan synthase, alpha subunit (trpA)	1	122.8	110.8	116.6	112.3	156.8	139.5	177.2	166.6
MSMEG _3222	MSMEG 3232	lgt (lgt)	3	46.6	44.3	28.3	31.9	46.5	50.7	25.7	38.4
MSMEG _3225	MSMEG 3235	glutamate synthase, large subunit	18	376.9	394.1	183.9	210.8	925.7	912.5	1245.7	1161.2

MSMEG _3227	MSMEG 3237	pyruvate kinase (pyk)	15	4777.7	4707.9	5243.4	5369.9	7627.2	7824.2	8471.8	8030.5
MSMEG _3234	MSMEG 3244	conserved hypothetical protein	1	2.8	3.2	3.6	4.6	13.5	14.6	14.8	13.2
MSMEG _3235	MSMEG 3245	glutamine ABC transporter, periplasmic glutamine-binding protein (gln	8	3004.0	3192.6	3888.8	4046.4	4849.7	5000.7	4213.8	4387.4
MSMEG _3244	MSMEG 3254	hypothetical protein	1	17.8	21.4	21.6	39.7	64.4	61.4	60.3	47.2
MSMEG _3246	MSMEG 3257	response regulator	6	496.2	523.0	462.9	431.1	809.4	872.6	750.2	788.7
MSMEG _3247	MSMEG 3258	branched-chain amino acid ABC transporter, periplasmic amino acid-bin	14	2999.8	2998.5	5766.5	5820.5	7886.7	8031.3	9444.4	9871.1
MSMEG _3253	MSMEG 3264	conserved hypothetical protein	5	666.1	738.6	611.2	659.8	990.1	1029.6	965.4	1017.7
MSMEG _3255	MSMEG 3266	Tat (twin-arginine translocation) pathway signal sequence domain prot	2	122.3	148.6	71.5	72.7	357.0	381.3	318.0	351.3
MSMEG _3257	MSMEG 3269	xylulokinase (xylB)	3	740.3	740.2	544.5	582.1	486.0	537.9	383.0	438.0
MSMEG _3278	MSMEG 3290	hypothetical protein	3	54.2	54.5	60.1	58.2	80.0	80.8	71.6	71.7
MSMEG _3280	MSMEG 3293	Bacterial extracellular solute-binding protein domain protein	4	170.3	162.7	193.5	204.4	487.7	493.8	277.5	368.1

MSMEG _3284	MSMEG 3296	transcriptional regulator, MarR family family	1	19.7	23.2	12.9	16.2	50.7	61.6	38.9	53.0
MSMEG _3287	MSMEG 3299	ephB (ephA)	2	82.5	90.7	112.1	129.3	90.4	103.4	79.8	101.1
MSMEG _3309	MSMEG 3318	pyridoxal-phosphate dependent TrpB-like enzyme	1	232.7	176.4	158.6	221.6	116.4	77.3	158.3	223.6
MSMEG _3379	MSMEG 3385	oxidoreductase, short chain dehydrogenase/reductase family	1	30.5	32.5	20.7	20.3	33.7	33.2	21.3	25.3
MSMEG _3380	MSMEG 3387	conserved hypothetical protein	1	163.2	180.8	114.3	131.9	144.1	149.0	234.2	219.2
MSMEG _3381	MSMEG 3388	acyl carrier protein phosphodiesterase	1	175.1	182.1	117.0	126.9	214.8	216.9	269.6	281.0
MSMEG _3394	MSMEG 3400	cupin domain protein	1	11.7	9.4	13.8	17.8	3.3	4.9	1.6	3.6
MSMEG _3434	MSMEG 3442	putative FK-506 binding protein, peptidyl-prolyl cis-trans isomerase	1	91.1	85.6	109.5	114.2	36.5	45.9	74.4	82.8
MSMEG _3461	MSMEG 3467	catalase/peroxidase HPI (katG)	23	2658.2	2565.4	2620.8	2810.2	6105.1	5786.4	6293.2	6306.4
MSMEG _3465	MSMEG 3471	acyl-CoA synthase (fadD28)	1	1623.0	1669.7	978.8	993.6	92.2	71.0	55.5	65.0
MSMEG _3478	MSMEG 3483	transcriptional regulator, putative	4	176.2	162.1	133.9	127.5	145.0	147.7	127.8	134.2
MSMEG _3479	MSMEG 3484	thiol peroxidase (tpx)	13	3661.3	4011.8	5089.5	5512.1	4580.6	5057.5	5365.7	5285.5

MSMEG _3489	MSMEG 3494	GDSL-like Lipase/Acylhydrolase, putative	2	23.5	5.2	22.3	24.8	181.5	6.8	160.1	148.7
MSMEG _3500	MSMEG 3504	Uncharacterized BCR	2	12.3	10.2	22.4	23.5	6.0	7.2	9.5	11.4
MSMEG _3505	MSMEG 3509	6-aminohexanoate-cyclic-dimer hydrolase (nylA)	1	210.2	202.9	74.4	80.4	247.8	255.1	171.9	175.2
MSMEG _3507	MSMEG 3511	Fructose-bisphosphate aldolase class-I	14	4026.5	4267.4	4766.6	4753.6	4723.7	4877.0	3205.0	3202.2
MSMEG _3512	MSMEG 3516	competence/damage-inducible protein CinA	1	93.9	86.8	121.9	79.4	98.9	85.5	133.3	110.6
MSMEG _3515	MSMEG 3518	oxidoreductase, short-chain dehydrogenase/reductase family (fabG3)	1	57.1	67.0	40.9	56.2	43.7	60.5	41.7	44.9
MSMEG _3564	MSMEG 3564	bacterioferritin (bfr)	11	837.2	894.7	1822.0	1890.7	4388.5	4579.8	4337.3	4209.4
MSMEG _3569	MSMEG 3569	conserved hypothetical protein	1	11.9	11.6	6.8	6.8	4.4	3.4	4.9	6.1
MSMEG _3574	MSMEG 3574	TPR Domain domain protein	2	159.9	159.8	183.8	174.0	104.5	108.8	108.5	110.4
MSMEG _3575	MSMEG 3576	yfhC protein VC0864	1	71.1	74.8	74.6	64.4	90.3	94.1	95.5	92.4
MSMEG _3580	MSMEG 3581	esterase, putative, antigen 85-C	3	473.7	441.2	300.8	291.0	35.5	30.0	29.1	34.1
MSMEG _3595	MSMEG 3599	hypothetical protein	1	12.0	7.9	7.2	8.9	9.4	5.3	9.2	10.2
MSMEG _3596	MSMEG 3600	hypothetical protein	4	46.1	49.1	32.8	39.9	56.5	62.0	50.3	56.4

MSMEG _3598	MSMEG 3602	sugar-binding transcriptional regulator, LacI family	1	110.4	96.2	147.3	130.4	173.8	176.1	167.4	157.8
MSMEG _3609	MSMEG 3614	Luciferase-like monooxygenase superfamily	1	27.6	29.3	28.9	26.5	41.3	41.0	79.9	88.4
MSMEG _3618	MSMEG 3624	hypothetical protein	2	1119.4	1096.9	724.9	784.7	1308.8	1337.4	1018.8	1240.4
MSMEG _3619	MSMEG 3625	oxidoreductase, short chain dehydrogenase/reductase family superfamil	2	324.6	331.0	260.0	283.8	90.0	102.9	127.6	127.2
MSMEG _3620	MSMEG 3626	Luciferase-like monooxygenase superfamily	8	1946.8	2043.9	989.8	1037.0	1326.0	1458.7	796.8	914.8
MSMEG _3621	MSMEG 3627	NADH dehydrogenase (ndh)	2	47.0	46.3	57.2	58.0	38.7	36.9	44.7	55.5
MSMEG _3623	MSMEG 3628	urease accessory protein UreG (ureG)	1	223.3	239.4	391.1	310.8	328.9	344.4	434.5	418.4
MSMEG _3625	MSMEG 3631	urease, alpha subunit (ureC)	6	376.1	349.0	257.4	272.7	713.0	724.1	380.7	368.4
MSMEG _3627	MSMEG 3633	urease, gamma subunit (ureA)	1	4.2	5.5	8.6	7.5	10.1	9.3	16.9	16.5
MSMEG _3628	MSMEG 3634	ComA2 protein, putative	2	117.6	114.2	89.3	100.2	89.5	79.0	80.8	85.8
MSMEG _3632	MSMEG 3638	6-phosphogluconate dehydrogenase, decarboxylating (gnd)	10	2145.9	2026.0	2447.9	2157.4	3292.2	3321.0	3472.3	3193.0

MSMEG _3634	MSMEG 3639	IMP dehydrogenase family protein	5	690.5	634.4	511.4	481.8	473.7	477.6	652.2	579.6
MSMEG _3640	MSMEG 3646	malate synthase G (glcB)	19	5180.1	5406.0	3809.9	3688.9	2737.0	2702.0	2926.6	2744.0
MSMEG _3642	MSMEG 3648	glycine dehydrogenase (gcvP)	17	2286.0	2194.4	4507.4	4192.3	2241.2	2157.4	2501.3	2362.9
MSMEG _3647	MSMEG 3653	FHA-domain-containing proteins	4	886.3	960.7	741.0	760.1	1178.4	1183.2	1358.1	1386.7
MSMEG _3648	MSMEG 3654	glycine cleavage system H protein (gcvH)	2	752.1	878.4	674.3	748.9	913.8	1024.4	861.4	870.6
MSMEG _3654	MSMEG 3660	preprotein translocase, SecA subunit (ATPase,RNAhelic)	7	423.3	414.9	383.5	391.4	501.6	489.6	606.1	626.2
MSMEG _3673	MSMEG 3679	4-alpha-glucanotransferase (malQ)	1	16.1	20.8	26.9	20.0	47.0	56.6	50.7	49.2
MSMEG _3710	MSMEG 3716	cytochrome b561, putative	1	14.1	0.0	0.9	2.1	4.2	0.0	1.1	1.4
MSMEG _3715	MSMEG 3722	non-ribosomal peptide synthetase	1	29.7	29.2	21.4	22.6	64.0	69.8	49.4	50.5
MSMEG _3740	MSMEG 3746	ribosomal large subunit pseudouridine synthase B (rluB)	2	130.7	147.7	158.9	154.3	164.2	171.5	165.4	176.6
MSMEG _3745	MSMEG 3751	MutT/nudix family protein	1	66.4	73.5	35.7	23.7	47.1	54.0	27.5	6.0
MSMEG _3746	MSMEG 3752	CTP synthase (pyrG)	10	648.7	655.5	769.9	717.6	2225.1	2219.2	2263.1	2098.2

MSMEG _3754	MSMEG 3760	conserved hypothetical protein	1	60.5	61.5	41.6	51.3	55.1	42.9	20.5	29.3
MSMEG _3758	MSMEG 3764	tyrosyl-tRNA synthetase (tyrS)	2	76.2	85.3	62.9	60.4	143.8	143.4	194.6	181.5
MSMEG _3767	MSMEG 3773	poly-beta-hydroxybutyrate polymerase/very long chain acyl-CoA dehydro	6	127.3	144.4	98.8	116.3	275.0	281.8	240.3	257.0
MSMEG _3770	MSMEG 3776	argininosuccinate synthase (argG)	7	3020.4	3092.0	2663.7	2729.4	886.2	985.2	942.1	993.0
MSMEG _3773	MSMEG 3779	acetylornithine aminotransferase	1	35.9	18.2	27.2	31.7	12.2	10.9	21.4	27.5
MSMEG _3775	MSMEG 3781	arginine biosynthesis bifunctional protein ArgJ (argJ)	4	588.4	699.7	552.9	585.4	318.8	345.3	301.4	324.7
MSMEG _3776	MSMEG 3782	N-acetyl-gamma-glutamyl-phosphate reductase (argC)	1	19.0	20.7	8.9	6.2	86.1	96.1	62.6	111.9
MSMEG _3777	MSMEG 3783	phenylalanyl-tRNA synthetase, beta subunit (pheT)	14	2247.7	2347.4	2516.4	2437.9	2043.8	2170.3	2480.6	2559.6
MSMEG _3778	MSMEG 3784	phenylalanyl-tRNA synthetase, alpha subunit (pheS)	1	33.4	36.9	22.2	28.4	18.2	21.0	25.5	8.1
MSMEG _3791	MSMEG 3797	ribosomal protein L20 (rplT)	5	902.3	884.8	807.2	917.3	595.9	576.3	556.6	583.0
MSMEG _3793	MSMEG 3799	translation initiation factor IF-3 (infC)	4	852.7	879.6	1064.1	1046.1	4362.8	4921.7	4523.2	4833.0

MSMEG _3808	MSMEG 3814	excinuclease ABC, A subunit (uvrA)	4	205.6	197.2	215.1	178.3	431.5	379.9	469.7	444.3
MSMEG _3810	MSMEG 3815	metallo-beta-lactamase superfamily protein	3	154.2	174.3	230.8	270.8	150.1	151.8	163.5	180.6
MSMEG _3811	MSMEG 3816	universal stress protein family domain protein	11	4651.0	5750.7	2128.5	2221.2	4640.6	5545.3	4298.2	4284.6
MSMEG _3816	MSMEG 3820	excinuclease ABC, B subunit (uvrB)	1	6.5	8.0	13.0	11.0	34.8	30.4	61.4	39.2
MSMEG _3831	MSMEG 3837	dephospho-CoA kinase, putative	1	10.0	7.8	7.7	6.0	7.1	5.4	6.7	5.0
MSMEG _3833	MSMEG 3838	rpsA (rpsA)	18	8923.2	8541.4	8540.4	7728.0	8084.7	7790.2	6975.7	6900.7
MSMEG _3839	MSMEG 3843	DNA polymerase I (polA)	13	1344.4	1327.0	1402.9	1237.9	1552.7	1594.0	1863.2	1647.2
MSMEG _3859	MSMEG 3862	apolipoprotein n-acyltransferase Lnt/dolichol-phosphate-mannosyl tra	1	16.9	19.6	15.6	15.1	33.8	35.6	19.4	16.7
MSMEG _3863	MSMEG 3866	conserved hypothetical protein	2	12.4	9.2	15.9	15.1	19.3	14.1	27.2	24.2
MSMEG _3864	MSMEG 3867	cobalamin biosynthesis protein N	6	167.7	160.0	105.4	111.4	117.2	128.8	107.4	110.7
MSMEG _3872	MSMEG 3875	precorrin-8X methylmutase	1	20.1	21.7	35.2	37.3	50.8	51.4	58.1	61.3
MSMEG _3881	MSMEG 3884	proline dipeptidase (pepQ)	2	304.6	233.3	247.4	248.2	344.2	298.0	379.3	384.2
MSMEG _3889	MSMEG 3893	repressor protein, putative	1	17.6	19.2	4.8	6.7	4.6	7.0	0.4	2.4

MSMEG _3895	MSMEG 3899	proteasome beta subunit	5	939.7	929.2	544.8	554.8	672.0	627.5	633.3	670.3
MSMEG _3897	MSMEG 3901	Putative proteasome component family	2	226.8	231.9	214.3	231.6	303.6	323.1	262.1	308.6
MSMEG _3902	MSMEG 3906	COG0464:ATPases of the AAA+ class	12	1888.0	1981.4	1636.4	1636.6	926.1	970.1	811.1	854.1
MSMEG _3906	MSMEG 3910	conserved hypothetical protein	2	59.8	50.9	94.7	93.7	89.0	89.9	104.3	99.1
MSMEG _3918	MSMEG 3923	hypothetical protein	1	6.7	6.9	10.6	16.7	75.7	77.1	64.2	81.0
MSMEG _3932	MSMEG 3937	heat shock protein, Hsp20 family (hsp)	1	168.0	149.7	10.8	13.7	3.9	3.7	3.5	4.6
MSMEG _3945	MSMEG 3950	universal stress protei	5	373.7	377.8	75.7	84.3	33.5	33.2	40.0	49.2
MSMEG _3950	MSMEG 3957	universal stress protei	5	1721.1	1750.7	572.7	584.0	152.7	144.3	146.0	143.0
MSMEG _3962	MSMEG 3970	lactate 2-monooxygenase	8	520.5	558.5	2994.6	3004.6	2952.5	2966.4	3174.4	2997.8
MSMEG _3980	MSMEG 3987	transcriptional regulator, GntR family	1	10.7	12.9	8.0	10.4	13.7	10.5	8.5	8.5
MSMEG _4042	MSMEG 4047	transcriptional regulator, GntR family, putative	2	106.7	104.6	140.9	116.9	150.0	143.0	131.3	155.1
MSMEG _4075	MSMEG 4082	blr3446	1	1.8	2.1	10.8	12.1	46.5	29.2	64.3	61.0
MSMEG _4125	MSMEG 4132	oxidoreductase, short-chain dehydrogenase/reductase family, putative	1	28.5	27.3	39.2	34.0	16.1	12.0	19.9	18.9

MSMEG _4180	MSMEG 4185	ATP phosphoribosyltransferase (hisG)	2	124.3	137.4	111.3	116.3	99.7	101.1	98.8	108.1
MSMEG _4185	MSMEG 4189	5-methyltetrahydrofolate--homocysteine methyltransferase (metH)	10	322.7	307.7	310.5	305.8	503.8	314.5	143.0	149.9
MSMEG _4188	MSMEG 4192	oxidoreductase, short-chain dehydrogenase/reductase family	3	82.4	77.9	77.7	79.3	123.8	119.0	92.0	112.7
MSMEG _4189	MSMEG 4193	cysteinyl-tRNA synthetase (cysS)	2	367.4	397.0	246.5	263.1	183.3	207.1	161.7	184.3
MSMEG _4193	MSMEG 4198	alpha-ribazole-5-phosphate phosphatase	2	99.9	90.8	66.8	64.3	107.3	78.4	84.6	90.7
MSMEG _4199	MSMEG 4204	conserved hypothetical protein TIGR00481	3	145.4	145.4	115.5	111.1	204.8	209.9	177.9	201.8
MSMEG _4205	MSMEG 4210	transcriptional regulator, LuxR family domain protein	1	60.5	64.0	35.6	37.5	90.9	100.8	71.3	81.1
MSMEG _4207	MSMEG 4213	universal stress protein family, putative	4	40.1	45.5	32.5	42.8	1020.8	1082.7	1019.5	982.9
MSMEG _4208	MSMEG 4214	COG3333:Uncharacterized BCR	1	36.9	39.0	6.2	29.1	83.3	129.4	18.7	77.7
MSMEG _4210	MSMEG 4216	Uncharacterized BCR	16	366.8	412.1	343.1	393.3	4426.2	4495.8	3840.4	3965.2
MSMEG _4217	MSMEG 4223	cell division protein DivIVA	7	788.7	742.0	749.3	794.1	1253.5	1151.2	1061.7	1215.5

MSMEG _4222	MSMEG 4228	cell division protein FtsZ (ftsZ)	3	176.4	177.4	109.8	99.4	330.0	331.6	320.4	338.0
MSMEG _4237	MSMEG 4241	SEQ ID NO 45F	1	27.3	28.2	14.5	19.6	12.6	15.6	12.1	16.6
MSMEG _4240	MSMEG 4244	idsA2 (idsA2)	5	601.9	619.1	464.0	470.5	580.9	599.4	496.4	527.3
MSMEG _4244	MSMEG 4247	3-deoxy-7-phosphoheptulonate synthase	1	364.5	351.8	327.3	331.6	262.4	271.1	276.4	284.2
MSMEG _4251	MSMEG 4254	conserved hypothetical protein	1	16.6	20.7	16.9	11.6	22.2	28.1	28.3	21.4
MSMEG _4254	MSMEG 4256	long-chain-fatty-acid--CoA ligase (fadD-2)	8	1285.1	1205.3	873.7	910.1	1190.5	1085.6	941.4	913.3
MSMEG _4258	MSMEG 4262	anthranilate phosphoribosyltransferase (trpD)	3	695.0	663.3	555.4	507.3	469.3	485.7	579.5	525.0
MSMEG _4262	MSMEG 4265	Rieske Fe-S Protein	5	908.0	782.5	921.1	1042.3	542.3	389.3	690.3	548.8
MSMEG _4263	MSMEG 4266	cytochrome B6, putative	2	47.7	44.7	69.4	61.3	45.3	41.6	84.2	73.7
MSMEG _4268	MSMEG 4271	cytochrome c oxidase, subunit II, putative	1	98.6	103.3	41.7	40.9	26.7	30.9	44.4	53.2
MSMEG _4269	MSMEG 4272	asparagine synthase (glutamine-hydrolyzing)	4	214.8	223.8	218.7	163.0	238.1	237.9	365.0	280.5
MSMEG _4270	MSMEG 4273	probable carbohydrate kinase	5	1511.7	1489.3	1781.7	1745.2	959.9	986.0	915.8	943.1
MSMEG _4272	MSMEG 4275	HesB/YadR/YfhF family protein	4	175.1	177.7	112.5	117.8	14.9	25.7	13.7	15.6
MSMEG _4273	MSMEG 4276	conserved hypothetical protein	1	13.7	17.2	13.1	17.2	7.8	10.0	6.7	10.2

MSMEG _4276	MSMEG 4280	branched-chain amino acid aminotransferase (ilvE)	6	1193.8	1082.0	853.4	796.0	1322.9	1257.6	1184.6	1231.3
MSMEG _4278	MSMEG 4281	glycine cleavage system T protein (gcvT)	4	719.8	657.4	360.1	340.1	700.9	727.6	492.6	554.9
MSMEG _4281	MSMEG 4283	cytosol aminopeptidase (pepA)	12	4788.3	4610.7	1886.3	1860.5	1896.6	1785.6	2010.9	2081.0
MSMEG _4283	MSMEG 4286	2-oxo acid dehydrogenases acyltransferase (catalytic domain) protein	19	5757.5	5669.6	6353.9	6768.9	8815.4	8503.2	10561.4	10900.3
MSMEG _4287	MSMEG 4290	conserved hypothetical protein	1	47.5	43.4	72.9	75.7	29.1	17.6	31.3	38.1
MSMEG _4288	MSMEG 4292	conserved hypothetical protein	1	13.7	15.4	14.6	20.8	6.3	7.0	2.6	7.6
MSMEG _4290	MSMEG 4293	glutamine synthetase, type I (glnA)	24	11712.2	11947.1	8646.5	8716.8	38488.9	39697.5	36050.3	35924.0
MSMEG _4291	MSMEG 4294	D-tyrosyl-tRNA(Tyr) deacylase (dtd)	1	29.1	12.0	21.8	23.9	48.0	16.2	37.2	39.8
MSMEG _4293	MSMEG 4296	glutamate-ammonia-ligase adenylyltransferase, putative	1	16.8	27.8	16.3	22.5	84.5	100.6	84.2	92.7
MSMEG _4294	MSMEG 4297	glutamine synthetase, type I (glnA)	4	464.1	475.3	559.1	576.9	1064.6	1098.4	1230.5	1276.1

MSMEG _4298	MSMEG 4301	3-methyl-2-oxobutanoate hydroxymethyltransferase (panB)	9	5011.7	4740.7	19350.9	20231.2	252.0	258.9	371.7	289.5
MSMEG _4299	MSMEG 4302	enoyl-CoA hydratase/isomerase PhaB (b1394)	2	211.1	221.6	158.5	175.8	63.6	64.5	54.4	60.6
MSMEG _4301	MSMEG 4304	acyl-CoA synthase (fadD28)	3	344.9	342.9	336.5	328.1	187.6	211.8	129.8	154.1
MSMEG _4302	MSMEG 4305	Adenylate cyclase, putative	1	124.6	127.3	94.1	111.1	182.0	190.6	183.4	206.3
MSMEG _4306	MSMEG 4311	Uncharacterized ACR, COG1579 superfamily	2	54.0	57.7	77.4	76.7	65.4	68.7	73.1	74.7
MSMEG _4313	MSMEG 4318	fosfomycin resistance protein fosB, putative	6	1100.5	1090.6	899.4	936.9	882.9	887.0	887.3	877.8
MSMEG _4320	MSMEG 4325	bacterioferritin comigratory protein, ahpC/TSA family (ahpE)	2	50.7	33.5	40.9	45.0	127.8	37.0	69.2	79.0
MSMEG _4321	MSMEG 4326	conserved hypothetical protein	1	36.8	43.2	29.7	35.8	60.9	73.2	62.1	74.1
MSMEG _4323	MSMEG 4328	pyruvate dehydrogenase E1 component (aceE)	18	4139.1	4358.0	3426.9	3641.9	5655.4	5782.0	5518.8	5912.3
MSMEG _4325	MSMEG 4330	malonyl CoA-acyl carrier protein transacylase (fabD)	4	589.4	613.8	658.9	615.1	505.1	515.6	636.8	583.7

MSMEG _4326	MSMEG 4331	acyl carrier protein (acpP)	4	1008.4	1065.7	1038.4	1118.1	1144.2	1241.7	1392.0	1390.7
MSMEG _4328	MSMEG 4334	3-oxoacyl- synthase (kasB)	6	1817.2	1959.8	1642.6	1644.6	1853.6	1847.3	1914.9	1916.9
MSMEG _4329	MSMEG 4335	methylmalonyl-CoA decarboxylase, subunit alpha (mmdA)	4	340.7	314.3	478.4	435.4	413.2	385.7	417.4	492.4
MSMEG _4330	MSMEG 4336	short chain dehydrogenase	1	3.7	3.4	6.3	4.9	7.5	5.4	8.0	5.3
MSMEG _4337	MSMEG 4343	conserved hypothetical protein	2	321.3	336.6	292.1	307.4	150.3	149.4	179.6	190.0
MSMEG _4340	MSMEG 4346	adhE2 (adhE2)	8	1545.7	1444.3	1134.3	987.4	1395.6	1280.6	1608.9	1346.0
MSMEG _4342	MSMEG 4347	metallo-beta-lactamase family protein	1	184.7	235.7	99.3	98.2	189.5	190.2	268.0	237.7
MSMEG _4358	MSMEG 4364	short chain dehydrogenase	1	28.3	34.3	28.6	37.6	48.5	113.3	54.3	53.1
MSMEG _4362	MSMEG 4368	universal stress protein family domain protein	2	60.1	66.2	59.3	58.4	99.1	99.2	92.1	92.5
MSMEG _4367	MSMEG 4372	formamidase	3	518.0	508.2	248.6	310.2	1496.7	1554.7	1181.3	1292.1
MSMEG _4381	MSMEG 4385	AMIDASE	1	18.2	19.8	6.3	7.0	58.4	67.4	31.9	53.3
MSMEG _4396	MSMEG 4399	isochorismatase family protein family	7	1556.9	1613.5	1724.9	1791.9	1121.8	1095.3	1234.1	1360.1
MSMEG _4401	MSMEG 4404	phosphoglycolate phosphatase, putative	5	852.7	866.8	581.7	619.6	558.4	599.6	487.5	526.0
MSMEG _4454	MSMEG 4449	low-specificity L-threonine aldolase	1	24.5	28.7	1.3	3.3	0.1	1.0	0.9	0.2

MSMEG _4463	MSMEG 4458	conserved hypothetical protein	2	21.7	22.5	10.5	10.8	13.1	13.6	8.4	10.9
MSMEG _4471	MSMEG 4466	transcriptional regulator, MarR family family	1	38.4	45.4	28.9	34.0	73.8	83.8	58.0	59.5
MSMEG _4474	MSMEG 4469	acyl-CoA oxidase homolog (proteinforMGC:9)	9	1112.1	1058.7	940.6	796.6	1112.0	1076.7	1363.4	1156.3
MSMEG _4475	MSMEG 4470	conserved hypothetical protein	1	11.8	8.5	6.0	8.4	15.9	13.0	12.5	16.3
MSMEG _4476	MSMEG 4471	hypothetical protein	1	19.2	0.0	15.6	17.0	15.7	0.0	8.6	5.6
MSMEG _4485	MSMEG 4480	glycyl-tRNA synthetase (glyS)	5	694.3	726.9	538.1	468.1	516.4	583.2	477.4	487.5
MSMEG _4486	MSMEG 4481	transcriptional regulator (smtB)	2	25.7	26.4	31.2	29.4	11.1	11.9	16.4	16.8
MSMEG _4487	MSMEG 4482	furB (furB)	1	453.1	513.7	392.8	402.7	37.8	43.3	36.7	35.0
MSMEG _4489	MSMEG 4484	conserved hypothetical protein	1	28.4	33.5	35.5	35.8	58.8	56.4	63.9	56.6
MSMEG _4490	MSMEG 4485	undecaprenyl diphosphate synthase (uppS)	1	27.4	27.6	25.7	21.7	30.0	24.1	10.2	8.7
MSMEG _4527	MSMEG 4520	nitrite reductase (nirA)	4	402.5	426.5	193.3	204.1	240.0	237.6	124.2	133.9
MSMEG _4528	MSMEG 4521	phosophoadenylyl-sulfate reductase (cysH)	2	170.1	192.4	123.0	123.4	60.2	58.3	66.4	72.9
MSMEG _4533	MSMEG 4526	subI (subI)	8	2169.4	2352.7	1875.1	1806.2	1589.4	1687.5	1406.1	1552.7
MSMEG _4535	MSMEG 4528	glycosyl hydrolase, family 15	19	1939.8	1983.1	1736.5	1692.6	3300.2	3278.2	3363.9	3127.2

MSMEG _4553	MSMEG 4547	GGDEF family protein VCA0785	2	96.9	105.2	96.3	100.5	98.3	102.1	85.9	80.1
MSMEG _4569	MSMEG 4566	conserved hypothetical protein	1	12.7	13.6	8.1	6.5	49.3	51.7	33.2	35.2
MSMEG _4570	MSMEG 4567	u1937b; B1937_F1_4	1	100.0	102.4	99.2	153.1	163.6	181.3	95.1	224.3
MSMEG _4571	MSMEG 4568	ribosomal protein S20 (rpsT)	1	138.4	131.5	279.5	262.8	43.9	45.1	97.2	96.9
MSMEG _4617	MSMEG 4609	glutamine-dependent NAD+ synthetase (nadE)	4	420.6	404.0	432.7	312.2	1828.0	1874.4	1411.5	1649.9
MSMEG _4624	MSMEG 4616	ribosomal protein L27 (rpmA)	4	932.4	942.4	855.6	943.4	391.5	391.4	398.2	406.0
MSMEG _4625	MSMEG 4617	ribosomal protein L21 (rplU)	4	1094.3	1000.5	1353.3	1453.6	807.0	724.8	742.9	810.1
MSMEG _4626	MSMEG 4618	rne (rne)	9	346.7	350.3	292.8	280.6	261.8	278.1	272.4	279.4
MSMEG _4627	MSMEG 4619	nucleoside diphosphate kinase (ndk)	3	1118.1	1140.2	954.8	993.1	629.2	626.6	745.9	684.0
MSMEG _4630	MSMEG 4622	valyl-tRNA synthetase (valS)	12	1845.3	1850.3	1603.6	1548.4	1146.7	1212.4	1327.4	1352.9
MSMEG _4636	MSMEG 4627	hypothetical protein	1	0.3	0.0	0.0	0.1	309.1	0.0	297.9	321.0
MSMEG _4645	MSMEG 4636	alpha oxoglutarate ferredoxin oxidoreductase, beta subunit (orB)	4	737.6	719.1	987.6	956.2	356.2	333.8	301.6	305.1
MSMEG _4646	MSMEG 4637	ferredoxin oxidoreductase, alpha subunit	15	1942.8	1951.1	3218.5	2991.5	934.4	942.3	978.7	952.2
MSMEG _4647	MSMEG 4638	putative sugar kinase	1	18.5	17.2	22.2	17.2	13.1	15.6	24.5	13.9

MSMEG _4664	MSMEG 4654	iolD protein	1	13.2	10.9	27.0	18.9	61.0	67.5	90.5	51.4
MSMEG _4668	MSMEG 4658	oxidoreductase alpha (molybdopterin) subunit	1	8.0	18.8	15.4	19.9	0.8	1.6	0.9	0.8
MSMEG _4672	MSMEG 4662	ATP-dependent Clp protease, proteolytic subunit ClpP (clpP)	6	2276.5	2260.3	1814.9	1916.3	1602.4	1540.2	1476.4	1418.0
MSMEG _4673	MSMEG 4663	ATP-dependent Clp protease, proteolytic subunit ClpP (clpP)	9	5605.8	5772.7	4743.9	4662.0	5780.4	5740.6	5551.5	5394.9
MSMEG _4674	MSMEG 4664	trigger factor (tig)	10	4105.1	4340.8	2914.1	2901.0	3150.9	3231.6	2601.9	2560.1
MSMEG _4679	MSMEG 4669	hypothetical protein	1	4.1	6.4	7.9	10.6	2.4	5.5	5.6	2.5
MSMEG _4687	MSMEG 4676	cytosine deaminase	4	162.5	155.6	165.1	178.1	325.1	303.1	282.8	277.7
MSMEG _4688	MSMEG 4677	DSBA-like thioredoxin domain family	1	59.5	69.9	48.3	37.2	29.4	44.3	40.2	33.5
MSMEG _4690	MSMEG 4679	pepD (pepD)	16	3104.3	2986.0	2944.6	2664.0	2214.2	2196.5	2674.2	2500.4
MSMEG _4692	MSMEG 4681	conserved hypothetical protein	4	662.2	681.3	688.5	713.2	358.0	371.5	330.6	329.0
MSMEG _4693	MSMEG 4682	conserved hypothetical protein	1	35.0	35.6	30.5	33.6	6.3	7.5	7.1	5.7
MSMEG _4698	MSMEG 4687	conserved hypothetical protein	1	169.2	162.7	138.0	140.2	191.6	250.6	158.1	175.7

MSMEG _4699	MSMEG 4688	NAD-glutamate dehydrogenase (NAD-GDH)	20	494.2	490.0	531.8	538.3	569.1	593.6	778.1	784.2
MSMEG _4700	MSMEG 4689	ABC-type transport system, ATPase component (AB041227)	11	1333.2	1272.7	841.7	731.0	1887.8	1866.8	1993.2	1727.8
MSMEG _4709	MSMEG 4698	enoyl-CoA hydratase/isomerase family protein (crt)	5	289.6	296.6	213.8	248.6	463.5	462.6	520.4	552.3
MSMEG _4714	MSMEG 4704	maoC protein (maoC)	1	15.8	17.2	14.1	19.4	22.2	20.1	7.1	16.2
MSMEG _4716	MSMEG 4706	biotin carboxylase (accA2)	1	11.0	14.7	17.7	22.5	26.7	35.6	31.0	35.6
MSMEG _4722	MSMEG 4713	oxidoreductase, short chain dehydrogenase/reductase family	6	592.9	589.9	555.1	550.5	645.9	673.9	693.5	720.6
MSMEG _4724	MSMEG 4715	exonuclease family	1	24.1	27.8	18.6	20.0	33.3	33.4	28.7	31.7
MSMEG _4727	MSMEG 4717	pks2 (pks2)	2	5.2	5.2	10.4	8.9	122.7	114.0	155.1	164.3
MSMEG _4742	MSMEG 4734	oxidoreductase, short-chain dehydrogenase/reductase family, putative	7	744.3	689.0	918.3	924.1	1342.4	1221.4	1227.1	1248.7
MSMEG _4752	MSMEG 4742	conserved hypothetical protein	1	220.0	228.2	209.0	205.9	229.0	249.5	229.1	217.2
MSMEG _4753	MSMEG 4743	bcp (bcp)	13	3255.8	3501.9	2274.8	2434.0	3881.1	4207.1	3563.0	3527.3
MSMEG _4755	MSMEG 4745	ArgE/DapE/Acy1 family protein	2	181.0	173.7	107.4	105.4	232.3	229.3	244.5	244.1

MSMEG _4757	MSMEG 4747	fatty-acid synthase (fas)	164	15609.0	15000.1	19787.8	19270.5	17072.0	16547.1	17004.2	15853.9
MSMEG _4847	MSMEG 4835	transcriptional regulator, TetR family domain protein	3	59.5	52.4	38.2	32.3	57.5	44.8	26.0	30.2
MSMEG _4888	MSMEG 4874	transcription regulator, LacI family	1	0.1	0.1	1.2	2.0	1.7	1.4	0.7	1.1
MSMEG _4891	MSMEG 4876	alkyl hydroperoxide reductase C	8	5046.3	5321.6	2821.1	2788.2	183.8	180.6	132.6	157.3
MSMEG _4901	MSMEG 4886	ribonuclease PH	2	173.2	201.4	231.8	238.4	242.2	289.0	240.9	293.8
MSMEG _4905	MSMEG 4890	cysteine synthase B	2	146.4	142.7	109.5	125.7	75.5	78.0	32.9	41.1
MSMEG _4906	MSMEG 4891	ThiS family domain protein	2	111.2	132.6	107.0	118.9	57.4	57.5	50.2	63.4
MSMEG _4911	MSMEG 4896	nicotinate phosphoribosyltransferase, putative	1	30.3	26.1	35.2	35.7	61.3	42.3	87.0	66.9
MSMEG _4914	MSMEG 4899	unnamed protein product	1	24.2	26.5	26.0	31.2	15.0	10.5	13.7	14.6
MSMEG _4915	MSMEG 4900	glycogen phosphorylase family protein (glgP)	19	1593.3	1603.3	1878.1	1778.7	2551.5	2515.6	3180.0	2860.7
MSMEG _4916	MSMEG 4901	alpha-amylase family protein	2	42.1	46.0	56.1	56.9	14.6	13.2	13.4	13.4
MSMEG _4917	MSMEG 4903	thioredoxin, putative	2	60.9	46.4	36.9	44.6	49.3	33.1	34.9	41.4
MSMEG _4918	MSMEG 4902	1,4-alpha-glucan branching enzyme (glgB)	3	244.5	231.1	180.6	212.8	314.3	316.5	301.1	334.1
MSMEG _4920	MSMEG 4905	acetyl-CoA acetyltransferase (atoB-1)	24	12838.6	11985.0	16220.4	15867.1	11936.8	11349.0	11340.5	10998.0

MSMEG _4921	MSMEG 4906	4-hydroxyphenylpyruvate dioxygenase C terminal domain containing prot	5	677.1	741.3	832.7	888.2	550.9	617.8	495.6	548.0
MSMEG _4932	MSMEG 4917	UDP-N-acetylglucosamine 1-carboxyvinyltransferase (murA)	4	334.4	336.5	314.5	339.9	298.7	268.2	213.5	229.0
MSMEG _4934	MSMEG 4918	ATP:cob(I)alamin adenosyltransferase, putative	3	421.5	423.6	356.7	373.7	247.9	257.9	222.8	242.0
MSMEG _4935	MSMEG 4920	ATP synthase F1, epsilon subunit (atpC)	4	261.2	268.7	793.6	749.6	305.6	325.9	379.5	344.5
MSMEG _4936	MSMEG 4921	ATP synthase F1, beta subunit (atpD)	19	3670.3	3748.4	12917.0	12912.8	8423.8	8748.2	9190.1	9573.7
MSMEG _4937	MSMEG 4922	ATP synthase F1, gamma subunit (atpG)	6	111.2	139.3	298.1	326.9	197.8	225.6	175.6	214.2
MSMEG _4940	MSMEG 4926	ATP synthase b chain (atpF)	2	92.2	114.8	234.1	243.8	82.0	101.4	83.4	83.8
MSMEG _4950	MSMEG 4935	peptide chain release factor 1 (prfA)	1	16.6	18.1	7.4	14.7	2.5	2.4	1.5	0.5
MSMEG _4951	MSMEG 4936	ribosomal protein L31 (rpmE)	3	693.3	782.3	763.5	779.8	360.3	428.9	381.5	408.5
MSMEG _4955	MSMEG 4941	homoserine kinase (thrB)	1	46.7	49.8	56.2	56.4	45.8	42.5	63.6	66.4
MSMEG _4956	MSMEG 4942	threonine synthase (thrC)	4	586.5	499.6	379.4	409.1	339.8	309.1	223.2	294.9
MSMEG _4957	MSMEG 4943	homoserine dehydrogenase	2	299.0	269.0	310.4	290.6	360.6	329.2	464.6	407.8

MSMEG _4958	MSMEG 4944	diaminopimelate decarboxylase (lysA)	2	144.3	131.5	152.5	126.1	443.4	445.5	417.7	427.7
MSMEG _4959	MSMEG 4945	arginyl-tRNA synthetase (argS)	3	248.6	240.5	266.2	259.5	325.7	309.9	370.9	340.8
MSMEG _4971	MSMEG 4957	enoyl-(acyl-carrier-protein) reductase II (fabK)	1	33.2	41.9	11.1	13.3	97.7	96.8	149.6	153.0
MSMEG _4974	MSMEG 4960	RrF2 family protein	2	15.2	16.3	16.5	20.1	17.3	16.9	21.4	21.1
MSMEG _4976	MSMEG 4962	isochorismatase family protein	10	1453.4	988.7	1621.3	1723.8	1305.6	1172.8	1127.4	1191.8
MSMEG _4978	MSMEG 4965	sulfate adenylytransferase, large subunit/adenylylsulfate kinase (cys	1	9.1	10.2	3.9	9.5	4.8	3.9	1.2	3.9
MSMEG _4985	MSMEG 4972	carbonic anhydrase-related protein	6	2222.3	1831.3	1817.4	1829.9	1643.6	1379.1	1557.7	1427.4
MSMEG _4997	MSMEG 4983	ABC transporter, ATP-binding protein	1	1.4	0.2	0.1	0.0	2.3	0.8	0.6	0.6
MSMEG _5005	MSMEG 4993	conserved hypothetical protein	2	154.0	169.3	115.1	131.0	109.0	113.8	100.0	97.2
MSMEG _5006	MSMEG 4992	phosphohistidine phosphatase SixA, putative	1	3.9	0.0	4.4	5.2	3.0	0.0	2.8	2.6
MSMEG _5022	MSMEG 5007	flavin-containing monooxygenase	3	11.1	11.3	43.8	35.3	63.9	70.4	112.3	75.8
MSMEG _5037	MSMEG 5024	glycolate oxidase, subunit GlcD (glcD)	1	118.4	150.3	87.1	113.0	46.1	61.0	46.7	63.8

MSMEG _5042	MSMEG 5030	ATP-dependent RNA helicase DeaD	11	481.5	488.3	322.1	394.9	499.1	521.4	511.2	618.0
MSMEG _5048	MSMEG 5036	conserved hypothetical protein	1	8.3	12.6	3.9	4.5	16.4	23.2	9.0	17.1
MSMEG _5049	MSMEG 5037	2-oxoglutarate dehydrogenase E1 component	54	6644.3	6329.4	5708.7	5617.2	7350.1	6826.6	6972.3	6472.8
MSMEG _5053	MSMEG 5041	short chain dehydrogenase family protein	1	14.6	10.7	16.4	16.0	21.8	20.6	24.2	23.3
MSMEG _5055	MSMEG 5043	malate oxidoreductase (oxaloacetatedec)	4	1131.6	1065.4	955.3	954.0	1273.6	1193.4	1218.4	1146.6
MSMEG _5058	MSMEG 5046	sugC (sugC)	2	83.7	83.0	165.1	145.1	139.3	151.7	224.9	186.4
MSMEG _5062	MSMEG 5050	conserved hypothetical protein	1	48.0	55.4	56.8	66.0	90.6	100.4	81.0	77.3
MSMEG _5064	MSMEG 5052	citrate lyase, beta subunit	3	157.2	176.6	128.2	137.4	135.3	145.0	95.1	96.6
MSMEG _5073	MSMEG 5061	O-methyltransferase superfamily	3	32.4	31.8	20.1	21.6	174.9	190.3	146.3	168.9
MSMEG _5086	MSMEG 5074	fadD6 (fadD6)	5	378.9	348.8	287.8	311.8	439.7	400.1	352.6	358.9
MSMEG _5104	MSMEG 5090	tetrahydrodipicolinate N-succinyltransferase (dapD)	5	1116.5	1157.0	612.9	646.5	708.7	758.4	711.5	667.7
MSMEG _5117	MSMEG 5103	Proline dehydrogenase superfamily	1	47.7	46.5	66.5	60.6	40.3	27.0	56.2	37.7

MSMEG _5119	MSMEG 5104	delta-1-pyrroline-5-carboxylate dehydrogenase (pruA)	7	81.8	79.0	97.8	91.7	916.1	832.5	589.2	523.1
MSMEG _5121	MSMEG 5107	aminotransferase, classes I and II	3	303.2	313.9	255.5	267.4	229.5	253.7	212.9	234.4
MSMEG _5122	MSMEG 5108	ferredoxin	1	198.0	204.9	212.7	233.9	282.0	276.4	316.4	300.6
MSMEG _5129	MSMEG 5117	Uncharacterized LmbE-like protein, COG2120 superfamily	4	361.3	333.6	231.5	253.7	294.6	304.0	270.2	291.6
MSMEG _5132	MSMEG 5120	GTP-binding protein TypA (typA)	5	265.2	289.5	156.5	208.8	745.0	730.0	579.6	655.5
MSMEG _5136	MSMEG 5121	conserved hypothetical protein	2	264.0	304.6	47.0	50.3	4.2	6.9	4.8	2.6
MSMEG _5150	MSMEG 5135	pterin-4-alpha-carbinolamine dehydratase	1	9.8	10.6	15.7	17.1	31.6	29.6	36.8	34.9
MSMEG _5164	MSMEG 5148	alcohol dehydrogenase, zinc-containing	2	101.6	103.9	19.5	32.1	993.1	971.9	429.3	504.7
MSMEG _5170	MSMEG 5154	conserved hypothetical protein	2	154.2	202.7	104.1	119.3	475.9	697.2	370.5	445.3
MSMEG _5183	MSMEG 5166	3-hydroxyacyl-CoA dehydrogenase FadB2x	7	1068.7	1079.6	769.3	781.3	1401.2	1378.7	1559.7	1553.9
MSMEG _5184	MSMEG 5167	alpha-methylacyl-CoA racemase, putative	1	64.6	71.5	41.7	45.7	438.2	638.3	258.5	260.2
MSMEG _5191	MSMEG 5175	anhydrase, family 3 protein	3	123.4	110.1	80.0	84.5	62.1	50.2	56.2	55.1

MSMEG_5197	MSMEG 5179	acyl-CoA dehydrogenase (fadE20)	2	71.0	69.8	98.9	97.9	67.4	104.8	61.7	82.2
MSMEG_5198	MSMEG 5180	echA1 (echA1)	2	70.6	83.5	117.5	136.0	52.1	58.0	53.3	78.5
MSMEG_5199	MSMEG 5181	thiolase family protein	2	435.2	435.5	519.1	531.8	406.1	384.0	375.0	434.8
MSMEG_5211	MSMEG 5192	aminotransferase, class III	7	499.8	491.8	479.7	487.9	547.7	551.4	581.0	608.8
MSMEG_5215	MSMEG 5199	conserved hypothetical protein	1	3.4	2.7	2.9	3.6	2.2	3.7	2.3	1.5
MSMEG_5225	MSMEG 5209	conserved hypothetical protein	4	244.3	242.4	675.3	754.9	873.9	882.0	936.2	938.6
MSMEG_5239	MSMEG 5224	fructose-1,6-bisphosphatase, class II (glpX)	4	1019.0	1028.6	610.1	601.7	1029.2	1059.7	805.8	840.4
MSMEG_5240	MSMEG 5225	fum (fum)	11	3706.6	3464.2	3787.2	3569.9	4030.1	3917.4	4933.0	4722.4
MSMEG_5243	MSMEG 5228	conserved hypothetical protein	1	263.5	274.0	61.2	71.8	15.7	17.8	19.4	17.8
MSMEG_5244	MSMEG 5229	DNA-binding response regulator,	1	6.6	0.0	1.2	0.9	8.7	0.0	2.0	2.7
MSMEG_5245	MSMEG 5230	universal stress protein family domain protein	3	537.1	555.2	76.2	83.7	41.7	51.0	31.0	37.0
MSMEG_5246	MSMEG 5231	conserved hypothetical protein	5	1936.4	1954.3	267.8	298.4	529.5	461.9	857.7	857.1
MSMEG_5248	MSMEG 5233	acyl-	6	895.5	939.4	726.2	786.6	393.0	420.4	420.1	469.8
MSMEG_5249	MSMEG 5234	serine hydroxymethyltransferase	9	3714.6	4001.3	3373.7	3404.1	4253.2	4573.5	4598.2	4794.2

MSMEG _5252	MSMEG 5236	pantothenate kinase VC0320	2	79.3	82.1	61.6	62.1	76.6	78.6	37.5	58.1
MSMEG _5258	MSMEG 5243	steroid Delta-isomerase	1	70.4	88.8	65.4	85.1	64.2	82.7	54.9	62.5
MSMEG _5263	MSMEG 5248	transcription elongation factor G greA	2	421.2	480.9	417.8	441.8	333.6	361.1	291.5	286.4
MSMEG _5265	MSMEG 5251	cystathionine beta-lyase	4	1757.5	1586.7	1280.8	1279.2	1116.8	1050.3	1130.8	1215.3
MSMEG _5270	MSMEG 5255	cystathionine beta-synthase	4	641.3	573.8	467.6	426.7	518.2	481.5	413.8	416.3
MSMEG _5273	MSMEG 5258	thiolase, putative	9	6050.6	5837.2	4485.1	4341.0	6404.6	6524.6	5928.3	6004.2
MSMEG _5274	MSMEG 5259	phosphoribosylglycinamide formyltransferase 2 (purT)	1	117.5	114.3	150.2	136.3	328.1	359.6	339.7	360.9
MSMEG _5285	MSMEG 5270	Patatin-like phospholipase family	1	161.4	164.7	39.2	46.7	22.7	23.1	14.4	17.1
MSMEG _5358	MSMEG 5339	Acetamidase/Formamidase family family	2	192.3	213.1	134.4	131.9	96.6	99.6	117.3	106.1
MSMEG _5359	MSMEG 5340	cyanate hydratase (cynS)	7	50.2	53.0	60.8	64.8	1050.3	1129.9	1124.2	1041.8
MSMEG _5397	MSMEG 5377	ATP-dependent DNA helicase RecQ, interruption-C	1	7.9	6.2	7.5	5.2	35.9	21.3	43.1	20.4
MSMEG _5404	MSMEG 5383	prpE protein VC1340	3	259.4	262.4	93.4	109.1	145.9	181.2	128.7	198.8
MSMEG _5413	MSMEG 5393	exopolyphosphatase	1	22.9	27.1	26.0	26.3	294.8	325.0	171.6	186.4
MSMEG _5415	MSMEG 5395	enolase (eno)	14	12106.2	11488.7	9458.8	8682.8	10548.6	10209.8	11963.1	11385.6
MSMEG _5419	MSMEG 5399	putative lipoprotein	7	1215.7	1133.2	396.9	383.8	415.0	422.3	465.2	400.1

MSMEG _5423	MSMEG 5404	transcription-repair coupling factor (mfd)	9	209.7	202.7	128.5	130.3	225.9	226.5	204.0	211.8
MSMEG _5424	MSMEG 5405	transcriptional regulator, TetR family	1	7.6	6.6	2.1	2.5	27.0	27.1	25.1	34.1
MSMEG _5427	MSMEG 5408	ribose-phosphate pyrophosphokinase (PRPPsynthetase)	10	1088.0	1209.9	968.9	971.8	570.4	623.1	323.0	348.2
MSMEG _5431	MSMEG 5412	ribosomal 5S rRNA E-loop binding protein Ctc/L25/TL5	3	1877.8	1827.8	2318.4	2376.1	1445.1	1384.4	1529.0	1546.3
MSMEG _5435	MSMEG 5415	pks16 (pks16)	8	1102.1	1021.2	1336.0	1507.5	704.4	772.1	392.3	498.3
MSMEG _5438	MSMEG 5418	dimethyladenosine transferase (ksgA)	1	182.5	162.2	219.1	198.8	527.8	496.8	451.0	444.7
MSMEG _5441	MSMEG 5421	methionyl-tRNA synthetase (metG)	1	87.1	87.9	87.9	76.1	88.3	72.4	68.2	69.5
MSMEG _5471	MSMEG 5450	UTP-glucose-1-phosphate uridylyltransferase (galU)	2	171.0	171.0	174.3	209.7	154.0	148.3	212.8	201.8
MSMEG _5479	MSMEG 5458	conserved hypothetical protein	1	43.2	46.5	31.9	30.8	0.4	0.6	0.0	1.1
MSMEG _5485	MSMEG 5465	molybdopterin biosynthesis protein (mog)	3	453.1	484.0	492.6	494.2	693.5	707.2	774.8	704.3
MSMEG _5486	MSMEG 5466	heat shock protein HtrA, putative	1	29.2	19.7	11.2	13.0	26.8	14.2	14.9	21.4

MSMEG _5488	MSMEG 5468	DNA-binding response regulator	1	45.8	52.3	37.2	39.2	90.7	88.3	112.3	116.7
MSMEG _5489	MSMEG 5469	ribosomal protein L32, putative	2	550.8	561.5	519.6	584.0	229.8	229.0	256.1	278.7
MSMEG _5512	MSMEG 5490	Mg-chelatase subunit ChlI	6	534.2	529.8	365.1	375.5	543.9	547.0	605.9	600.0
MSMEG _5515	MSMEG 5493	phosphoribosylaminoimidazolecarboxamide formyltransferase/IMP cyclohy	4	120.7	116.7	97.5	92.5	94.4	93.1	90.5	106.9
MSMEG _5520	MSMEG 5498	N5,N10-methylenetetrahydromethanopterin reductase-related protein	3	231.0	210.0	324.0	302.8	122.8	118.6	85.9	103.9
MSMEG _5524	MSMEG 5502	hypothetical protein	15	3426.2	3383.8	5592.9	5294.5	4452.4	4459.5	4674.9	4374.8
MSMEG _5525	MSMEG 5503	carbamoyl-phosphate synthase, large subunit (carB)	13	12254.2	11646.1	12668.7	12267.9	11324.9	11027.3	11061.8	10519.7
MSMEG _5534	MSMEG 5512	ATP-dependent DNA helicase PcrA (pcrA)	1	4.0	4.2	8.2	9.5	0.1	0.0	0.2	0.1
MSMEG _5538	MSMEG 5516	NAD-dependent aldehyde dehydrogenases	6	1127.2	1066.8	851.1	705.7	675.1	638.9	694.6	674.0
MSMEG _5541	MSMEG 5517	glucose-6-phosphate isomerase (pgi)	5	1804.1	1755.4	1438.7	1286.8	1161.3	1161.2	1357.9	1203.2
MSMEG _5553	MSMEG 5530	methyltransferase (multi-domain)	1	60.8	55.7	71.0	74.1	153.4	138.6	110.1	115.2
MSMEG _5592	MSMEG 5572	Rif11	3	73.2	100.2	68.8	92.8	200.0	238.1	229.1	262.2

MSMEG _5612	MSMEG 5590	acetyltransferase, GNAT family	2	138.2	131.2	220.9	226.8	84.5	94.4	59.1	88.1
MSMEG _5615	MSMEG 5592	conserved hypothetical protein	1	34.2	44.1	25.4	33.1	58.4	67.9	46.4	51.5
MSMEG _5634	MSMEG 5613	conserved hypothetical protein	1	23.6	15.5	18.2	19.0	20.6	13.0	10.6	12.9
MSMEG _5639	MSMEG 5618	enoyl-CoA hydratase (fad-1)	12	1979.6	1985.5	1682.1	1685.3	1463.5	1433.9	1298.4	1309.3
MSMEG _5650	MSMEG 5630	acetyl-CoA synthetase (acs-6)	1	2.0	3.1	4.8	5.7	22.2	38.4	5.4	10.4
MSMEG _5662	MSMEG 5641	DNA-binding response regulator PrrA (prrA)	5	443.7	483.6	433.0	470.4	695.1	707.9	763.1	761.9
MSMEG _5664	MSMEG 5643	peptidyl-prolyl cis-trans isomerase FkbP	5	332.5	300.1	266.3	285.7	252.1	238.6	280.3	271.9
MSMEG _5672	MSMEG 5650	citrate synthase I (gltA)	15	8510.7	8503.2	5881.6	5877.6	7366.9	7400.8	7112.2	7159.4
MSMEG _5675	MSMEG 5653	pyridoxamine 5-phosphate oxidase (pdxH)	2	226.1	253.5	186.0	181.6	122.0	147.6	134.6	144.5
MSMEG _5676	MSMEG 5654	putative citrate synthase 2 (citA)	2	200.8	186.5	84.2	78.2	259.5	226.4	132.9	125.5
MSMEG _5680	MSMEG 5658	glyoxalase family protein	8	585.3	524.7	433.2	451.0	567.7	523.2	477.7	468.2
MSMEG _5684	MSMEG 5662	phosphoserine aminotransferase, putative	6	1667.0	1455.0	1334.7	1224.0	1277.1	1081.6	1274.1	1280.2
MSMEG _5690	MSMEG 5667	hypothetical protein	3	249.2	274.2	253.5	278.7	325.1	354.9	283.7	296.9
MSMEG _5694	MSMEG 5670	conserved hypothetical protein	1	26.8	0.0	13.5	15.9	98.4	0.0	159.6	132.1

MSMEG _5696	MSMEG 5672	cspB (cspB)	2	178.2	186.7	197.1	209.3	208.9	219.7	183.2	190.9
MSMEG _5701	MSMEG 5677	molybdopterin converting factor, subunit 2	1	23.6	26.0	22.0	22.3	28.8	28.9	27.9	24.8
MSMEG _5702	MSMEG 5678	mog (mog)	3	117.2	123.4	55.4	58.7	130.5	133.7	131.0	126.9
MSMEG _5703	MSMEG 5679	molybdenum cofactor biosynthesis protein C (moaC)	1	105.3	0.0	42.8	72.2	89.6	0.0	43.7	74.9
MSMEG _5706	MSMEG 5682	Helicase conserved C-terminal domain protein	1	51.2	52.9	35.2	34.6	39.0	39.1	25.3	34.3
MSMEG _5707	MSMEG 5683	conserved hypothetical protein	2	166.2	136.5	164.6	175.8	197.6	166.8	208.0	211.4
MSMEG _5715	MSMEG 5691	Luciferase-like monooxygenase superfamily	6	2379.2	2375.8	1167.9	1194.2	2476.0	2331.5	1936.1	1873.6
MSMEG _5720	MSMEG 5695	fatty oxidation complex, alpha subunit (fadB)	20	1958.0	1877.3	2636.2	2290.2	875.8	835.6	1032.7	895.8
MSMEG _5721	MSMEG 5696	thiolase	3	434.0	474.0	435.0	422.9	190.6	218.2	175.6	197.3
MSMEG _5725	MSMEG 5700	aminotransferase, class I	2	331.0	339.1	199.4	196.3	220.0	257.6	276.2	318.8
MSMEG _5727	MSMEG 5702	allantoicase	1	51.6	48.0	36.1	36.4	98.6	98.2	89.7	73.2
MSMEG _5732	MSMEG 5707	N5,N10-methylene-tetrahydromethanopterin reductase (mer), putative	1	45.9	53.2	21.2	29.1	59.3	76.5	47.9	59.8

MSMEG _5733	MSMEG 5708	universal stress protein family domain protein	2	125.1	123.5	54.0	48.9	4.0	2.9	2.0	2.0
MSMEG _5734	MSMEG 5709	far (far)	2	363.7	334.8	206.0	199.6	244.0	249.1	149.8	200.3
MSMEG _5739	MSMEG 5713	substrate--CoA ligase, putative	6	155.0	141.1	22.6	25.0	52.7	47.1	38.9	34.6
MSMEG _5773	MSMEG 5743	fatty acid desaturase (desA1)	1	206.6	219.8	149.7	144.5	2.4	4.5	1.9	1.8
MSMEG _5776	MSMEG 5747	phosphate transport system regulatory protein, putative	3	295.6	326.4	195.6	197.6	370.9	403.8	375.2	398.8
MSMEG _5784	MSMEG 5755	transcriptional regulator (drrA)	2	77.0	86.0	98.2	105.6	243.1	265.7	244.2	270.8
MSMEG _5789	MSMEG 5759	thiosulfate sulfurtransferase (cysA-1)	16	9903.7	9840.0	9702.1	9446.5	4363.8	4447.5	4109.9	4146.9
MSMEG _5790	MSMEG 5760	conserved hypothetica	3	2104.9	2348.3	1742.3	1792.2	354.5	399.7	439.9	426.6
MSMEG _5792	MSMEG 5762	conserved hypothetical protein	2	124.2	126.9	104.4	114.0	141.3	158.1	112.7	118.7
MSMEG _5795	MSMEG 5764	aminotransferase, class IV	2	70.1	65.7	66.4	68.9	52.6	45.3	44.3	47.3
MSMEG _5798	MSMEG 5766	phosphoribosylformylglycinamidine cyclo-ligase (purM)	2	151.1	141.2	146.3	121.6	601.6	548.2	675.8	609.3
MSMEG _5804	MSMEG 5771	hypothetical protein	1	308.8	303.3	336.9	326.2	4.5	9.5	1.1	4.0
MSMEG _5824	MSMEG 5789	phosphoribosylformylglycinamidine synthase II (purL)	16	2922.0	3087.7	2215.8	2568.4	1992.9	2067.5	1926.2	2238.6

MSMEG _5830	MSMEG 5795	Linocin_M18 bacteriocin protein superfamily	4	592.6	618.9	750.5	748.1	894.0	878.3	962.7	963.7
MSMEG _5831	MSMEG 5796	phosphoribosylformylglycinamidine synthase I (purQ)	4	1036.0	1100.8	793.8	793.5	625.2	658.8	648.8	641.4
MSMEG _5832	MSMEG 5797	phosphoribosylformylglycinamidine synthase, PurS protein (purS)	1	136.9	179.8	94.0	107.7	233.6	303.6	228.0	250.6
MSMEG _5834	MSMEG 5800	metallo-beta-lactamase superfamily domain protein	1	21.5	16.1	9.9	14.6	22.5	17.7	16.2	19.4
MSMEG _5835	MSMEG 5801	3-ketosteroid-delta-1-dehydrogenase, putative	1	16.3	13.0	54.9	53.1	5.3	7.8	20.5	25.1
MSMEG _5837	MSMEG 5803	glutathione peroxidase family protein	2	439.1	499.7	243.7	266.6	338.7	382.0	261.4	268.2
MSMEG _5839	MSMEG 5806	protease II	5	430.7	441.0	407.7	413.4	258.2	281.7	234.2	298.7
MSMEG _5841	MSMEG 5807	phosphoribosylaminoimidazole-succinocarboxamide synthase (purC)	7	443.0	478.8	371.7	395.9	562.7	600.2	481.0	500.3
MSMEG _5847	MSMEG 5813	adenylosuccinate lyase (purB)	4	649.6	580.5	544.6	534.1	1212.1	1246.5	1623.7	1580.4
MSMEG _5852	MSMEG 5818	phosphoribosylamine--glycine ligase (purD)	9	1437.6	1166.3	1133.1	1173.0	1148.9	946.4	1322.3	1354.7
MSMEG _5871	MSMEG 5835	HIT family protein (pkcI)	7	634.9	663.7	804.3	808.5	604.2	618.5	760.5	697.8

MSMEG _5872	MSMEG 5837	DNA-binding response regulator PhoP (phoP)	2	405.6	394.6	174.0	171.8	166.5	171.9	135.3	146.0
MSMEG _5873	MSMEG 5838	conserved hypothetical protein	1	39.6	34.0	22.4	28.5	34.0	36.1	29.1	34.3
MSMEG _5884	MSMEG 5848	2-hydroxy-3-oxopropionate reductase, putative	3	258.3	239.9	190.7	190.9	246.5	247.7	177.0	183.3
MSMEG _5892	MSMEG 5854	alpha,alpha-trehalose-phosphate synthase (otsA)	2	90.4	98.3	103.7	102.3	138.7	152.2	157.1	156.6
MSMEG _5893	MSMEG 5856	conserved hypothetical protein	1	68.6	82.5	70.3	73.4	23.4	21.2	16.6	15.5
MSMEG _5925	MSMEG 5884	iron-sulfur cluster-binding protein, rieske family domain protein	1	35.5	31.6	20.5	32.5	4.7	2.6	5.0	5.2
MSMEG _5935	MSMEG 5893	ATP-dependent DNA helicase RecQ (recQ-1)	1	21.1	21.4	23.9	28.2	29.4	30.1	28.5	35.1
MSMEG _5937	MSMEG 5895	HMGL-like domain protein	2	400.4	360.5	408.8	375.0	260.0	225.1	201.9	189.0
MSMEG _5939	MSMEG 5896	acetaldehyde dehydrogenase (acetylating) (EC 1.2.1.10) Rv3535c	3	178.9	188.0	204.2	227.2	107.7	118.5	96.5	119.8
MSMEG _5940	MSMEG 5897	2-oxo-hepta-3-ene-1,7-dioate hydratase (hpaH)	2	158.6	166.2	300.7	266.5	295.2	312.5	344.1	340.7
MSMEG _5943	MSMEG 5899	MaoC family protein (ufaA2)	3	220.0	244.3	348.1	391.1	380.1	389.1	374.5	381.7

MSMEG _6008	MSMEG 5965	fadA6 (fadA6)	2	123.7	161.7	96.5	126.8	147.5	144.0	175.0	186.4
MSMEG _6024	MSMEG 5983	acetoacetyl-CoA synthase	3	108.2	106.3	71.4	80.0	71.8	69.8	42.8	70.4
MSMEG _6073	MSMEG 6035	spoU rRNA methylase family protein	1	19.1	16.1	9.8	9.0	13.9	12.1	7.6	8.2
MSMEG _6075	MSMEG 6037	2C-methyl-D-erythritol 2,4-cyclodiphosphate synthase (ispF)	1	5.8	0.0	5.4	4.4	21.4	0.0	12.0	15.9
MSMEG _6076	MSMEG 6038	2-C-methyl-D-erythritol 4-phosphate cytidylyltransferase (ispD)	2	40.8	39.2	34.8	34.2	94.6	87.5	101.4	125.0
MSMEG _6077	MSMEG 6039	CarD-like transcriptional regulator family	1	30.6	26.1	18.5	20.7	1.9	1.7	0.5	0.2
MSMEG _6082	MSMEG 6044	carbonic anhydrase (icfA)	4	291.7	307.3	419.0	417.5	612.4	659.1	614.8	574.4
MSMEG _6086	MSMEG 6049	Antibiotic biosynthesis monooxygenase domain protein	4	500.7	597.7	491.9	461.2	455.4	529.2	648.9	606.1
MSMEG _6091	MSMEG 6054	clpC (clpC)	25	3502.1	3281.9	3883.1	3651.7	5182.1	4894.8	6314.3	6124.2
MSMEG _6092	MSMEG 6056	LSR2 protein precursor (15 kDa antigen) (A15)	3	555.2	448.8	501.9	529.3	259.2	276.7	324.2	315.5
MSMEG _6094	MSMEG 6057	lysyl-tRNA synthetase (lysS)	5	432.3	432.5	185.8	225.8	505.8	497.1	310.5	394.1
MSMEG _6096	MSMEG 6059	transcriptional activator, putative, Baf family subfamily	1	75.4	75.1	103.2	93.0	41.3	49.5	42.8	57.5

MSMEG _6097	MSMEG 6060	pantoate--beta-alanine ligase (panC)	2	989.8	1004.7	2888.0	2875.8	1387.3	1308.3	1635.2	1616.1
MSMEG _6105	MSMEG 6068	ATP-dependent metalloprotease FtsH	5	395.6	406.1	560.7	518.8	396.6	399.6	315.1	322.0
MSMEG _6110	MSMEG 6073	hypoxanthine phosphoribosyltransferase (hpt)	3	28.8	31.8	25.5	34.6	95.4	96.1	42.1	59.2
MSMEG _6114	MSMEG 6077	inorganic pyrophosphatase (ppa)	6	414.3	346.2	425.2	468.1	410.9	331.7	398.6	432.5
MSMEG _6127	MSMEG 6090	anti-anti-sigma factor family	2	7.9	1.2	12.3	16.6	12.3	7.5	20.0	22.6
MSMEG _6128	MSMEG 6092	response regulator	1	16.4	18.1	35.6	39.4	29.7	36.5	30.4	31.4
MSMEG _6142	MSMEG 6105	NAD-dependent epimerase/dehydratase family protein	10	1161.0	1116.4	732.6	760.0	748.5	756.0	619.4	627.9
MSMEG _6157	MSMEG 6120	DNA topoisomerase I (topA)	13	667.9	662.1	683.0	649.6	784.7	816.1	757.5	849.7
MSMEG _6159	MSMEG 6122	probable cold shock protein	1	10.7	15.0	33.5	38.3	7.9	10.0	31.9	35.4
MSMEG _6179	MSMEG 6139	acetyl-CoA synthase	16	2127.0	2140.0	2827.0	2713.6	4649.9	4332.5	4787.4	4495.6
MSMEG _6189	MSMEG 6148	cyclic nucleotide-binding domain protein	7	3634.0	3792.3	3803.1	3567.2	4352.4	4325.3	4330.0	4149.2
MSMEG _6191	MSMEG 6150	Putative translation initiation inhibitor	5	445.8	364.2	330.1	296.2	289.6	225.4	285.4	237.1
MSMEG _6195	MSMEG 6153	Anion-transporting ATPase superfamily	1	150.2	144.7	151.8	162.0	192.5	208.7	206.0	226.5

MSMEG _6207	MSMEG 6167	conserved hypothetical protein	6	73.2	53.7	74.2	72.7	171.8	130.9	167.5	164.8
MSMEG _6210	MSMEG 6170	hypothetical protein	1	2.5	3.9	18.0	22.7	110.0	124.1	99.6	109.6
MSMEG _6213	MSMEG 6172	Mn-containing catalase (dividedwithOB16)	1	11.3	14.5	6.7	12.2	93.9	110.0	109.7	118.2
MSMEG _6215	MSMEG 6174	Protein of unknown function (DUF541) superfamily	1	143.6	135.4	166.0	158.7	178.9	153.0	139.3	161.8
MSMEG _6229	MSMEG 6190	glycerol kinase (glpK)	4	277.0	265.8	658.1	683.1	398.7	405.4	411.9	444.1
MSMEG _6242	MSMEG 6203	alcohol dehydrogenase II	1	449.0	424.8	476.7	443.2	60.3	73.4	109.3	106.8
MSMEG _6243	MSMEG 6204	conserved hypothetical protein	1	7.4	6.7	24.0	10.1	10.1	12.2	11.8	11.7
MSMEG _6256	MSMEG 6217	aspartate-semialdehyde dehydrogenase (asd)	7	1305.0	1226.9	1178.4	1152.0	1554.7	1487.1	1414.1	1475.3
MSMEG _6257	MSMEG 6218	aspartate kinase, monofunctional class	3	928.3	1008.2	753.6	742.2	1246.4	1383.8	1198.0	1167.2
MSMEG _6271	MSMEG 6231	2-isopropylmalate synthase (leuA)	4	404.3	439.3	299.9	430.8	205.6	213.1	185.2	224.7
MSMEG _6280	MSMEG 6240	conserved hypothetical protein TIGR00103, putative	3	65.0	69.5	60.2	73.6	113.2	101.1	120.0	144.6
MSMEG _6282	MSMEG 6242	conserved hypothetical protein	6	784.4	882.9	518.3	557.3	2557.3	3016.3	2680.7	2582.9

MSMEG _6283	MSMEG 6243	FAD binding domain protein	1	32.0	25.0	71.0	55.3	37.6	34.8	23.2	23.2
MSMEG _6284	MSMEG 6244	cyclopropane-fatty-acyl-phospholipid synthase	4	1141.1	1077.3	1087.2	996.0	1076.7	1081.4	1298.2	1257.3
MSMEG _6285	MSMEG 6245	DNA polymerase III, subunit	3	192.2	198.3	193.0	196.9	180.2	191.8	186.7	208.5
MSMEG _6286	MSMEG 6246	conserved hypothetical protein	3	284.3	242.2	258.3	256.3	146.9	142.7	239.7	252.3
MSMEG _6288	MSMEG 6249	conserved hypothetical protein	1	10.5	13.8	1.8	12.4	6.9	11.1	0.1	9.5
MSMEG _6309	MSMEG 6269	amino acid ABC transporter, ATP-binding protein	1	43.8	44.4	60.0	56.1	251.4	233.3	255.6	195.2
MSMEG _6317	MSMEG 6277	arylesterase	2	638.6	695.8	436.2	460.8	454.2	464.5	348.9	373.0
MSMEG _6329	MSMEG 6290	conserved hypothetical protein	2	29.7	34.2	56.0	54.7	38.5	48.1	42.1	39.8
MSMEG _6337	MSMEG 6297	fadE36 (fadE36)	1	45.8	58.6	42.3	54.3	340.7	332.4	231.1	251.7
MSMEG _6351	MSMEG 6314	histidinol-phosphate aminotransferase (hisC)	4	1143.0	1092.2	933.8	883.0	697.2	722.9	640.4	611.6
MSMEG _6352	MSMEG 6315	conserved hypothetical protein	3	87.1	94.7	64.6	78.1	45.9	58.6	41.1	47.1
MSMEG _6353	MSMEG 6316	enoyl-CoA hydratase/isomerase family protein (fad-1)	8	672.9	718.6	644.1	657.5	684.3	733.4	608.6	680.7
MSMEG _6354	MSMEG 6317	serine esterase, cutinase family	1	5.1	3.7	6.1	5.2	53.8	48.8	61.3	52.2

MSMEG _6363	MSMEG 6325	conserved hypothetical protein	3	261.0	254.8	175.4	178.1	143.1	206.3	98.4	124.3
MSMEG _6365	MSMEG 6327	conserved hypothetical protein	1	115.9	124.3	123.0	126.3	108.2	123.7	137.8	142.9
MSMEG _6384	MSMEG 6346	catalase/peroxidase HPI (katG)	10	1355.4	1376.5	2392.4	2210.0	1121.3	1168.9	1431.4	1195.3
MSMEG _6385	MSMEG 6347	putative protein belonging to the short-chain alcohol dehydrogenase	3	367.6	353.4	274.0	308.5	380.1	348.5	311.7	344.2
MSMEG _6391	MSMEG 6353	propionyl-CoA carboxylase, beta subunit (pccB)	11	3131.8	2948.2	3140.8	2855.8	3389.0	3260.6	2999.8	2698.6
MSMEG _6392	MSMEG 6354	POLYKETIDE SYNTHASE PKS13	56	2971.6	2870.5	3227.1	3257.4	5675.2	5399.8	5144.1	4852.1
MSMEG _6393	MSMEG 6355	acyl-CoA synthase (fadD32)	26	6175.9	5755.1	7214.2	7245.2	9720.8	8839.8	9187.1	8986.5
MSMEG _6398	MSMEG 6360	esterase, putative, antigen 85-A	9	1520.5	1598.7	2290.2	2480.5	525.0	566.4	443.3	484.4
MSMEG _6399	MSMEG 6361	esterase, putative, antigen 85-C	2	26.3	29.4	73.3	69.5	29.5	44.0	37.7	38.4
MSMEG _6402	MSMEG 6364	PAP2 superfamily domain protein	5	119.6	97.6	95.4	90.8	92.6	77.5	75.9	75.2
MSMEG _6403	MSMEG 6365	conserved hypothetical protein	5	196.9	104.2	181.1	111.1	107.5	58.2	111.0	82.4

MSMEG _6404	MSMEG 6366	UDP-galactopyranose mutase (glf)	6	538.0	461.9	600.8	583.5	367.8	345.3	670.4	689.7
MSMEG _6408	MSMEG 6371	Acyltransferase domain protein	1	110.5	136.2	166.5	162.3	219.3	283.1	228.4	262.4
MSMEG _6409	MSMEG 6372	Acyltransferase domain protein	1	71.5	73.6	56.7	81.7	834.4	873.0	1192.1	1196.5
MSMEG _6410	MSMEG 6373	iron-sulfur cluster-binding protein, rieske family domain protein	3	263.9	249.7	324.4	288.3	249.8	274.7	272.9	252.5
MSMEG _6412	MSMEG 6375	conserved hypothetical protein	6	697.0	688.0	819.2	821.9	751.5	760.6	762.2	777.2
MSMEG _6413	MSMEG 6376	seryl-tRNA synthetase (serS)	1	128.7	119.4	130.4	130.1	23.5	26.6	17.8	16.8
MSMEG _6418	MSMEG 6381	pheA (pheA)	2	94.7	103.8	134.5	136.0	88.7	89.1	68.2	80.7
MSMEG _6422	MSMEG 6385	bfrB (bfrB)	3	58.4	71.3	167.2	170.0	418.1	461.1	452.1	444.8
MSMEG _6425	MSMEG 6388	rhodanese-like domain protein	1	31.5	37.7	25.0	27.2	76.0	56.7	46.9	63.3
MSMEG _6427	MSMEG 6390	superoxide dismutase (mn)	5	11360.2	12830.2	6802.7	8154.8	5403.9	6087.0	3957.8	4732.8
MSMEG _6431	MSMEG 6392	conserved hypothetical protein	13	1507.7	1442.4	1492.3	1555.2	927.4	903.9	938.8	959.5
MSMEG _6434	MSMEG 6397	conserved hypothetical protein	2	379.1	367.8	323.6	345.0	515.4	538.3	271.5	331.0
MSMEG _6439	MSMEG 6402	protein of unknown function, TIGR01935	2	576.1	587.1	444.3	422.7	387.6	378.9	401.6	344.9
MSMEG _6440	MSMEG 6403	arylesterase/monoxygenase	1	30.7	22.2	6.7	5.3	8.4	5.8	0.7	1.8

MSMEG _6452	MSMEG 6415	aldehyde dehydrogenase family protein	6	1705.9	1574.7	1795.3	1580.8	1546.2	1431.6	1878.9	1728.1
MSMEG _6454	MSMEG 6416	Luciferase-like monooxygenase superfamily	12	3758.4	3282.9	609.6	605.1	379.9	334.9	408.1	421.2
MSMEG _6457	MSMEG 6419	conserved hypothetical protein	1	8.9	14.5	42.9	52.0	37.5	44.9	37.4	53.1
MSMEG _6458	MSMEG 6420	gltD (gltD)	1	115.3	124.9	129.0	125.8	73.6	81.6	9.2	14.2
MSMEG _6459	MSMEG 6421	glutamate synthase, large subunit	19	592.2	561.8	772.4	740.0	674.2	673.1	651.8	719.5
MSMEG _6464	MSMEG 6424	uncharacterized domain 1 protein	1	1.3	1.4	3.9	2.9	4.7	3.2	5.7	4.4
MSMEG _6467	MSMEG 6427	Dps family protein, putative	1	108.1	106.8	62.7	63.2	255.7	235.2	259.6	226.8
MSMEG _6471	MSMEG 6431	conserved hypothetical protein	1	84.6	80.7	59.1	45.0	30.9	25.1	57.7	36.0
MSMEG _6477	MSMEG 6437	peptide methionine sulfoxide reductase (msrA)	1	44.5	48.9	30.4	31.3	6.6	10.7	2.9	5.4
MSMEG _6502	MSMEG 6463	hypothetical protein	4	354.4	283.9	394.5	411.4	304.9	282.1	326.7	297.0
MSMEG _6506	MSMEG 6467	unnamed protein product	1	39.3	44.9	29.9	30.7	288.7	309.1	430.3	313.7
MSMEG _6507	MSMEG 6469	glycogen operon protein GlgX (glgX)	9	778.0	792.4	468.0	458.0	764.3	828.2	730.0	731.8
MSMEG _6511	MSMEG 6473	bll7818 (fadE26)	1	185.1	194.2	143.6	153.9	136.8	138.7	101.3	106.3
MSMEG _6512	MSMEG 6474	bll7817 (fadE18)	5	634.5	601.8	486.8	462.3	928.8	846.4	825.5	805.0

MSMEG _6515	MSMEG 6477	alpha-amylase family protein	1	50.3	72.7	19.1	42.7	58.3	96.5	33.5	94.0
MSMEG _6520	MSMEG 6479	orotate phosphoribosyltransferase (EC 2.4.2.10)	3	130.3	109.7	131.0	146.2	305.2	271.4	273.2	291.0
MSMEG _6524	MSMEG 6483	ABC transporter, periplasmic binding protein	1	103.0	109.7	178.1	167.1	374.6	390.2	350.4	406.2
MSMEG _6535	MSMEG 1396	translation elongation factor G (fusA)	30	8506.0	8169.9	9041.9	8032.6	10787.6	10293.7	10491.8	9816.5
MSMEG _6561	MSMEG 6523	ltp2, putative	1	3.7	0.0	1.6	1.5	6.0	0.0	5.1	5.7
MSMEG _6565	MSMEG 6527	conserved hypothetical protein	5	736.0	783.8	584.9	598.3	649.5	686.1	619.4	621.9
MSMEG _6574	MSMEG 6535	conserved hypothetical protein	1	66.4	66.7	29.2	31.0	49.4	52.2	33.9	35.4
MSMEG _6587	MSMEG 6549	ATP-dependent helicase hrpA (hrpA)	1	3.7	4.4	1.8	2.7	5.9	6.5	3.2	5.0
MSMEG _6596	MSMEG 6557	conserved hypothetical protein	5	709.3	746.0	603.9	701.2	278.1	289.4	191.0	194.9
MSMEG _6622	MSMEG 6584	P450 heme-thiolate protein	1	235.3	286.3	177.9	164.1	35.2	46.3	42.8	45.9
MSMEG _6630	MSMEG 6591	glyoxalase family protein superfamily	5	298.0	88.3	293.3	308.8	304.7	139.2	356.8	314.4
MSMEG _6636	MSMEG 6597	superoxide dismutase (mn)	4	1738.9	1771.1	1406.5	1392.7	1423.3	1395.8	1116.5	1095.5

MSMEG _6638	MSMEG 6600	5-methyltetrahydropteroyltriglutamate--homocysteine S-methyltransfera	23	4736.4	5085.6	10217.6	10050.7	1772.5	1880.5	2128.0	2035.7
MSMEG _6733	MSMEG 6692	carbon-nitrogen hydrolase family protein	1	82.1	87.0	78.6	97.0	92.6	97.4	95.6	112.9
MSMEG _6740	MSMEG 6701	1-aminocyclopropane-1-carboxylate deaminase	3	243.5	281.3	167.6	185.8	342.0	410.6	340.8	336.3
MSMEG _6741	MSMEG 6702	ANTAR domain protein	1	36.9	43.1	32.2	38.9	56.3	65.9	66.3	83.8
MSMEG _6743	MSMEG 6704	glyoxalase family protein, putative	1	40.0	39.7	53.1	49.8	35.8	38.0	36.6	31.5
MSMEG _6746	MSMEG 6707	oxidoreductase, aldo/keto reductase family	10	2381.4	2320.5	1546.8	1540.2	2409.3	2334.9	2561.4	2400.5
MSMEG _6754	MSMEG 6715	MaoC like domain protein	2	390.6	402.4	295.7	322.3	197.4	230.7	141.8	176.3
MSMEG _6756	MSMEG 6717	glycerol kinase (glpK)	1	24.8	20.2	30.3	28.4	51.0	46.8	47.0	43.2
MSMEG _6759	MSMEG 6720	glycerol kinase (glpK)	22	10985.4	10760.3	29889.0	29843.9	17852.3	17052.6	18325.0	18830.9
MSMEG _6760	MSMEG 6722	Aha1 domain superfamily	1	78.4	76.9	36.1	43.3	22.0	17.6	17.1	19.4
MSMEG _6761	MSMEG 6721	glpD2 (glpD2)	5	342.0	366.1	708.5	742.3	175.3	198.0	119.8	162.4
MSMEG _6776	MSMEG 6737	transcription regulator LysR family VC0896	1	81.7	61.5	71.2	62.4	181.3	117.4	77.3	74.0
MSMEG _6787	MSMEG 6749	sugar-phosphate isomerases, RpiB/LacA/LacB family	2	36.7	21.0	27.1	32.4	244.9	205.7	306.4	294.6

MSMEG _6816	MSMEG 6779	molybdopterin oxidoreductase	10	209.8	238.5	102.5	178.3	2213.4	2093.3	2019.7	1882.6
MSMEG _6822	MSMEG 6786	conserved hypothetical protein	3	589.7	718.2	502.6	674.9	766.6	866.8	745.0	799.7
MSMEG _6854	MSMEG 6817	aconitase (acn)	2	133.9	155.1	93.1	120.5	1.7	1.9	0.8	1.2
MSMEG _6875	MSMEG 6839	Endoribonuclease L-PSP family	1	71.0	71.4	30.0	32.6	102.1	115.8	70.0	112.0
MSMEG _6894	MSMEG 6855	ribosomal protein L9 (rplI)	8	2354.4	2556.9	2372.6	2453.8	1501.4	1647.5	1626.2	1462.8
MSMEG _6896	MSMEG 6857	single-strand binding protein homolog	5	1549.5	1680.8	1293.1	1457.8	804.5	840.8	840.5	855.3
MSMEG _6897	MSMEG 6858	ribosomal protein S6 (rpsF)	5	430.1	375.9	494.0	487.3	361.2	313.7	413.5	377.5
MSMEG _6904	MSMEG 6866	1L-myo-inositol-1-phosphate synthase	12	2975.1	2919.7	3401.1	3365.2	930.4	1017.2	962.7	1190.6
MSMEG _6907	MSMEG 6868	conserved hypothetical protein	2	163.6	148.3	124.9	127.9	191.7	175.3	209.4	176.4
MSMEG _6913	MSMEG 6872	transcriptional regulator, MarR family	1	11.2	0.0	11.1	12.1	12.9	0.0	9.2	9.9
MSMEG _6917	MSMEG 6875	leucyl-tRNA synthetase (leuS)	20	1716.7	1502.8	1190.7	1322.2	2054.4	2184.7	1760.2	2127.8
MSMEG _6926	MSMEG 6882	pcnA (pcnA)	1	0.7	0.6	1.2	1.2	1.8	1.4	0.3	0.4
MSMEG _6929	MSMEG 6886	integral membrane protein MviN, putative	1	16.0	13.3	16.8	16.9	42.5	46.3	33.2	43.7
MSMEG _6933	MSMEG 6889	thioredoxin-disulfide reductase (trxB)	3	417.4	433.6	406.6	424.1	230.2	218.6	119.5	131.2

MSMEG _6934	MSMEG 6890	thioredoxin (trx)	4	146.7	103.3	93.1	102.0	234.5	175.7	183.9	193.3
MSMEG _6941	MSMEG 6896	R3H domain protein	6	517.4	506.3	552.3	549.2	307.9	297.3	382.0	392.0
MSMEG _6947	MSMEG 6901	chromosomal replication initiator protein DnaA (dnaA)	2	32.0	38.3	8.6	19.1	108.2	124.0	33.7	51.0
MSMEG 1843	MSMEG 1843	adenosylhomocysteinase	3	906.3	977.0	1142.5	1202.9	1005.5	1070.7	764.0	880.3
MSMEG 2968	MSMEG 2968	substrate--CoA ligase, putative	3	149.7	126.6	1.3	1.8	17.6	18.4	3.1	7.5
MSMEG 3192	MSMEG 3192	maltooligosyltrehalose synthase (treY)	2	176.9	181.7	113.8	119.4	176.0	178.5	205.5	178.1
MSMEG 3892	MSMEG 3892	conserved hypothetical protein	1	22.1	19.6	8.9	11.4	7.5	8.0	1.7	3.9
MSMEG 5534	MSMEG 5534	conserved hypothetical protein	2	112.2	119.9	72.5	79.3	68.3	72.7	45.2	51.1

Supplemental Table II. List of quantified peptide charge states (PCSs).

Proteins		PCS		PCS quantitaion							
				A PCS							
				from protein sample S_P				from protein sample R_P			
Locus	old locus	**Tryptic peptide sequence**	**Chai**	$S_{P,1}$	$S_{P,2}$	$cS_{P,1}$	$cS_{P,2}$	$R_{P,1}$	$R_{P,2}$	$cR_{P,1}$	$cR_{P,2}$
MSMEG _0001	MSMEG 0001	K.AGTDGNQVHLALGSGAS VGK.D	2	31.7	22.0	119.1	57.9	32.2	35.9	126.5	142.4
		K.AGTDGNQVHLALGSGAS VGK.D	3	139.7	143.2	146.8	149.6	169.0	141.4	175.3	161.8
		R.EDFADAVAWVAR.S	2	100.3	99.1	70.5	80.9	107.5	108.7	71.6	78.9
MSMEG _0002	MSMEG 0002	-.MQLGLIGLGK.M	2	21.3	24.7	58.8	62.8	48.8	50.2	62.2	65.6
		K.ALKEDPGFAEISGYTEDS GEGR.W	3	132.2	140.4	222.3	227.2	270.4	257.4	332.2	341.9
		K.EDPGFAEISGYTEDSGEG R.W	2	26.1	26.2	51.9	51.8	23.4	25.0	22.9	27.8
		R.SWLQSLLAK.A	2	33.1	31.8	62.4	59.4	60.3	54.2	68.5	58.5
		R.VPMPVIAASLFAR.F	2	61.0	62.9	172.3	182.7	114.7	105.1	206.0	212.2
		R.WTVEEAIAHR.V	3	2.1	0.1	4.2	0.8	8.4	6.6	2.1	4.0
MSMEG _0005	MSMEG 0005	K.EYGADSITILEGLEAVR.K	2	193.3	184.6	71.9	58.9	231.9	192.3	80.0	72.8
		K.FDGETYAVSGGLHGVGV SVVNALSTR.L	3	261.1	243.9	124.9	122.2	302.3	275.2	120.0	125.1
		K.GLTIELTDER.V	2	19.4	14.7	10.6	11.7	25.9	13.8	5.1	10.2
		K.IC#NEQLQHWFEANPAEA K.T	3	229.0	262.4	112.6	106.6	244.6	267.2	144.6	115.5
		K.IHADGSVEVR.D	2	4.6	3.3	1.0	0.7	5.3	4.7	1.1	1.3
		K.NTEVQSIITALGTGIHDEF DISK.L	3	312.0	366.7	120.4	136.9	357.3	372.4	154.3	154.0
		K.SELYVVEGDSAGGSAK.S	2	17.3	13.9	2.3	3.6	8.2	11.0	0.5	1.1
		K.TPIQQSIIDFDGK.G	2	200.5	220.0	401.9	406.1	153.2	177.9	268.9	282.2

		R.DSMFQAILPLR.G	2	79.7	71.0	15.6	15.7	92.4	70.3	22.1	15.8
		R.EGLAAVISVK.V	2	20.4	14.7	14.5	11.7	28.6	20.0	7.0	6.5
		R.STDPSKSELYVVEGDSAG GSAK.S	3	29.7	32.6	30.2	19.4	42.6	49.6	27.6	23.5
		R.VFHYPGGLVDYVK.H	2	95.2	98.0	41.6	50.6	105.0	111.4	54.8	66.3
		R.VTAEEVVDDVVKDTAEAP K.T	2	96.9	98.4	30.2	29.1	143.7	122.6	45.2	75.2
		R.VTAEEVVDDVVKDTAEAP K.T	3	219.1	220.8	49.8	49.0	305.8	273.8	89.1	65.4
MSMEG _0006	MSMEG 0006	K.ALDALDEVIALIR.A	2	147.2	146.2	62.6	59.3	226.9	170.7	98.9	70.7
		K.ALDALDEVIALIR.A	3	10.1	8.7	0.3	0.3	11.5	13.6	0.9	1.7
		K.IEAEIADLEDILAKPER.Q	2	68.2	70.8	104.8	95.1	80.6	84.7	167.0	249.0
		K.IEAEIADLEDILAKPER.Q	3	126.3	119.1	43.3	44.4	151.4	135.8	47.9	46.4
		K.LAGISNIEDQSSDR.V	2	37.5	30.2	11.7	9.0	30.9	32.3	14.3	12.5
		K.SYEDAPYLVLATR.N	2	175.1	201.4	50.2	52.4	247.3	227.3	70.5	77.7
		R.EIDEETVDFIPNYDGR.V	2	116.8	116.9	21.0	30.6	127.7	145.1	26.9	34.3
		R.GQHVANLLAFQPEER.I	2	81.5	79.6	10.3	10.7	106.2	117.6	14.0	21.1
		R.GVVEIEEDSR.G	2	1.5	0.8	0.6	0.1	1.0	2.0	0.1	0.2
		R.IEPVDIQQEMQR.S	2	115.8	102.0	26.5	27.3	94.0	94.4	13.7	20.8
		R.IVPADGEVSDEDLIAR.E	2	591.8	565.4	220.5	206.4	625.5	668.0	221.8	244.3
		R.LLSLNVVRPDTYLLVATSG GYAK.R	3	288.4	318.9	106.6	128.3	355.4	407.4	136.2	147.6
		R.LMNLAEGDTLIAIAR.N	2	135.0	121.3	160.1	116.8	210.5	135.8	186.7	132.1
		R.LYVDHQLDVIVR.R	3	54.8	46.5	4.6	3.7	78.6	56.4	5.4	3.7
		R.NADEDEAAESISESDADT AESPEA	2	12.9	24.0	0.2	1.6	2.6	22.8	0.1	3.1
		R.SGGIVAINLR.E	2	6.1	2.8	3.4	2.5	8.6	8.3	10.9	6.1
		R.SVAETMGNYHPHGDASIY DTLVR.M	3	100.3	110.7	33.8	53.3	114.6	123.9	24.2	57.4
		R.TSIVITELPYQVNHDNFITS IAEQVR.D	3	193.7	249.0	98.2	166.7	238.5	282.6	96.1	155.8
		R.VKGPDFPTSGLIVGTQGIE DTYK.T	3	247.6	285.8	113.7	130.7	354.1	353.7	115.3	154.6
MSMEG _0023	MSMEG 0023	R.GVLGIGADTAQATAAELR. R	2	237.3	284.0	175.7	216.3	148.5	145.3	215.5	199.4

		R.SRPQEPPTLAPSVEVAPKP	2	23.5	22.5	11.6	13.9	9.0	8.2	4.5	5.3
		R.SRPQEPPTLAPSVEVAPKP	3	208.4	233.5	197.6	221.9	110.6	123.8	171.0	179.2
MSMEG_0024	MSMEG0024	K.DYSTENASGGTSGPFYDGAVFHR.V	2	194.5	189.1	83.2	88.0	119.3	111.7	65.3	82.4
		K.DYSTENASGGTSGPFYDGAVFHR.V	3	1935.2	2251.3	1318.3	1484.3	1253.2	1364.6	1228.1	1307.4
		K.TVNNFVGLAQGTK.D	2	99.5	85.2	245.1	221.7	75.6	74.8	197.3	215.3
		K.VVDAIASTPTDR.S	2	3.1	2.8	0.5	0.5	2.9	2.4	0.7	0.8
		R.HTIFGEVVDEESQK.V	2	646.9	453.0	462.5	316.8	506.6	467.2	483.6	452.4
		R.HTIFGEVVDEESQK.V	3	413.9	349.8	240.0	208.5	329.9	287.8	299.4	248.6
		R.RHTIFGEVVDEESQK.V	3	86.7	95.8	19.5	24.2	48.2	54.5	19.0	26.7
		R.SDRPTEPVVIESITIA	2	91.8	99.2	30.4	29.5	67.8	72.7	28.0	29.2
		R.VIDGFM#IQGGDPTGTGR.G	2	201.5	195.6	102.2	110.3	123.7	138.0	100.2	111.7
		R.VIDGFM*IQGGDPTGTGR.G	2	219.7	221.9	103.6	113.3	130.9	144.3	100.3	112.4
		R.VIDGFMIQGGDPTGTGR.G	2	1158.7	1306.7	870.0	921.5	840.1	860.5	912.3	931.1
		R.VIDGFMIQGGDPTGTGR.G	3	89.1	91.4	48.8	41.3	61.2	50.9	40.4	43.4
MSMEG_0033	MSMEG0033	K.EALQIEDVAESADR.L	2	316.7	251.6	77.2	85.2	259.6	232.3	48.9	56.2
MSMEG_0034	MSMEG0034	R.ITLGNQPVLIGR.A	2	11.4	11.7	25.5	31.0	18.8	21.0	21.4	20.1
MSMEG_0042	MSMEG0042	R.FAADDTPSPLTEAFAQLR.D	2	16.4	19.0	9.8	10.5	9.1	13.0	12.5	9.8
MSMEG_0048	MSMEG0048	R.DPTVTVLIEDGHTYDTLR.G	2	5.9	11.8	12.3	19.6	16.0	15.9	4.4	7.1
		R.GVSIDGTAEIVDDPETLLR.V	2	33.0	33.0	30.7	34.6	78.6	75.0	29.3	24.6
MSMEG_0057	MSMEG0056	R.ALEGLVEFLPSK.T	2	6.9	5.9	53.1	45.5	18.2	18.1	31.6	32.7
MSMEG_0059	MSMEG0058	R.ALGGVLFIDEAYTLVQER.N	2	1.4	4.0	39.6	51.5	1.1	2.8	14.4	36.9
		R.IEFDSYSPDEIVEISK.V	2	28.5	38.0	195.7	210.2	20.2	37.1	127.6	162.2

		R.NGQTDPFGAEALDTLLAR.M	2	28.7	43.5	60.8	64.7	16.5	28.2	38.3	58.6
		R.SDPWDPSSVVADTSAR.D	2	27.0	36.3	90.8	108.9	15.3	44.3	54.0	83.3
MSMEG_0065	MSMEG 0064	K.GVIAQVESTGSALAAQMVGQAGTAAQAALAR.F	3	12.3	16.7	15.7	24.3	16.5	25.7	8.8	16.2
MSMEG_0066	MSMEG 0065	K.ASLTTLASAWGGTGSEAYQAVQAR.W	3	81.1	76.4	116.5	101.3	159.9	177.5	117.6	125.8
MSMEG_0067	MSMEG 0066	R.ILAIDADPDGGNLADR.A	2	25.9	33.2	97.8	113.1	175.2	172.2	133.9	146.8
MSMEG_0076	MSMEG 0074	R.EFEETEWASEWDR.Q	2	0.2	0.6	22.4	33.0	0.2	0.7	8.6	26.9
MSMEG_0081	MSMEG 0078	R.ERLDALVADITAEDV	2	391.5	406.5	7.4	11.2	175.3	206.3	0.6	5.5
MSMEG_0084	MSMEG 0082	K.GAQVTVSSDDADVLAK.V	2	4.2	3.4	11.9	8.9	4.8	6.1	16.1	15.1
MSMEG_0091	MSMEG 0089	R.VWSSLDPDVYTVQSVVDR.L	2	12.2	18.0	57.7	54.5	15.0	19.0	17.9	27.8
MSMEG_0092	MSMEG 0090	K.FVAWAVNDAIER.G	2	52.4	63.1	120.4	141.2	34.3	43.6	55.3	64.4
		K.FVAWAVNDAIER.G	3	7.1	5.6	1.3	0.4	6.6	7.3	1.7	1.5
		R.ADLTRPAINHYFGSK.R	3	24.1	23.0	74.0	65.1	39.4	42.1	61.6	76.1
		R.EVVEQTNATVIAAGVAR.A	2	17.2	22.3	155.3	155.0	32.3	33.0	120.1	131.2
		R.HPDLVSEEQDALLNSR.K	2	112.3	133.7	154.2	142.4	80.2	89.8	98.6	116.1
		R.KFVAWAVNDAIER.G	2	83.9	112.9	77.9	95.2	150.0	157.3	72.1	75.1
		R.KFVAWAVNDAIER.G	3	37.9	33.6	22.3	22.7	47.3	40.1	25.2	25.8
		R.SEELAAIVDKFEMLMANK.L	2	3.3	4.9	11.2	8.0	5.3	5.0	7.0	5.3
		R.SEELAAIVDKFEMLMANK.L	3	9.6	9.1	29.4	30.6	16.5	13.4	26.1	23.7
		R.SSAAFLVTSVLESQR.H	2	45.3	54.7	28.2	32.7	49.8	54.1	27.5	27.1
MSMEG_0108	MSMEG 0105	K.GVSC#FLVPGDLPGLSFGKPEEK.M	3	112.1	94.9	56.6	53.8	108.9	108.8	92.5	91.2
MSMEG_0110	MSMEG 0107	K.VNAPSEGEIAALR.D	2	13.0	17.9	123.9	111.0	31.7	34.8	137.0	101.6

		R.DGATLISLISPALRPDLVEQLGTR.N	3	4.3	4.1	4.0	5.5	5.7	7.0	2.8	4.5
		R.LPAQSSQLYGTNLVNLLK.L	2	21.3	20.5	32.9	35.0	43.1	42.1	40.5	29.3
MSMEG_0120	MSMEG0116	R.VESPLGIDVQAGSEEQR	2	8.8	8.2	99.9	102.9	9.9	13.5	52.0	70.2
MSMEG_0131	MSMEG0125	K.ALEVVDALPR.N	2	1.7	1.3	7.1	9.1	7.1	6.2	4.1	13.8
MSMEG_0166	MSMEG0160	K.AIAAHDVEQATDIMR.R	2	23.1	24.1	23.8	22.9	25.5	31.2	41.6	39.1
MSMEG_0215	MSMEG0205	R.GVSVTDWLQSR.H	2	2.9	1.8	6.1	3.6	0.8	2.0	3.7	4.1
MSMEG_0216	MSMEG0206	K.GGVVGLTLPVAR.D	2	556.6	536.9	332.4	310.3	499.8	443.0	378.6	331.1
		K.VLAINLVGTFDC#IR.I	2	270.8	238.3	416.3	387.6	179.4	153.4	435.3	381.3
		K.VLAINLVGTFDC*IR.I	2	270.8	238.3	416.3	387.6	179.0	153.4	435.3	381.3
		K.VVIADLQADKGEALAK.E	2	356.9	315.8	379.6	399.5	347.3	303.1	1042.5	1278.8
		R.ALVNSAGIGWAQR.T	2	914.6	868.1	519.6	518.5	707.4	975.1	480.2	566.8
		R.VNTVAPGLIDTPIYGEGEGSEAFK.A	2	300.1	341.4	201.8	214.2	206.2	236.1	185.7	202.3
		R.VNTVAPGLIDTPIYGEGEGSEAFK.A	3	373.0	415.3	251.7	264.1	245.7	269.2	186.0	217.2
MSMEG_0220	MSMEG0210	K.LVLDDVTSWIVSHL	2	4.5	4.4	11.6	17.9	3.4	3.0	9.4	19.5
MSMEG_0224	MSMEG0214	K.LLSLLAGISGAR.R	2	96.5	89.0	36.1	31.2	44.8	41.2	27.7	29.2
		R.VEILVGTALDTLPK.L	2	187.0	181.6	144.0	149.7	68.1	68.4	89.6	107.1
MSMEG_0229	MSMEG0219	K.GTLDVLVDEEEFEAR.K	2	396.0	366.6	348.7	316.5	263.4	254.1	364.4	303.0
		K.SAGFDSDVFEGTAR.V	2	75.6	60.6	331.3	249.9	37.2	30.5	167.4	119.8
		R.AMNNPIHPTGGITILHGSLAPEGAVVK.S	4	163.9	147.5	284.9	250.7	98.5	89.9	147.6	153.3
		R.EAEVELTLDDFTR.V	2	68.9	74.9	77.4	80.2	40.8	37.7	51.7	40.7
		R.EVIADSVETVVQAER.L	2	1753.8	1710.2	1267.8	1185.8	1488.3	1436.6	1381.0	1247.0
		R.LDLASVFFYNGSIMPGVAK.L	2	33.5	41.8	37.4	35.8	10.1	21.9	12.4	28.8

MSMEG _0233	MSMEG 0223	R.VGDYLVATVDDSTHQMEIR.R	3	90.9	101.6	132.2	141.7	79.3	110.1	182.7	176.3
MSMEG _0234	MSMEG 0224	K.ALIEQYEQFTPR.G	2	649.2	697.0	319.5	332.7	378.0	373.8	336.4	302.5
		K.EAPVIDGLTGVQR.V	2	333.3	244.7	192.3	122.3	247.3	105.7	250.5	107.7
		K.MFGLIYGGDHTDTAAR.I	2	104.1	107.4	12.1	20.3	44.8	63.7	19.9	39.5
		K.MFGLIYGGDHTDTAAR.I	3	130.0	118.6	36.7	34.9	88.1	85.4	50.0	53.5
		K.YDGDGNLVDWWTDADR.T	2	205.5	231.1	72.2	79.2	95.6	117.1	78.6	88.8
		R.AGLLTDDLVAEDFAFYGR.T	2	402.8	371.3	193.5	173.5	279.0	238.3	256.0	201.7
		R.AVSVVENLMGDALGK.L	2	613.6	616.3	440.0	423.0	523.9	377.0	508.4	413.0
		R.DEQHAEILAGYPVHIAK.M	2	38.8	40.1	6.2	6.5	16.8	18.8	3.1	8.3
		R.DEQHAEILAGYPVHIAK.M	3	190.1	191.9	49.1	65.1	101.0	101.8	48.3	48.3
		R.DLITEAAASGAADGTDEQR.I	2	260.2	259.4	74.5	77.4	111.5	125.6	59.9	81.5
		R.IGDLYASFMDTQTIAER.G	2	169.2	153.1	45.8	37.8	111.4	86.7	59.7	37.4
		R.TGVGGGAGVYVDTDSK.N	2	117.1	103.0	52.1	47.4	59.0	68.9	44.1	54.3
		R.VFFGWAQVWR.T	2	54.3	65.1	8.7	16.4	34.4	37.4	10.6	18.8
		R.VSIDELDWM*TPETR.A	2	33.4	29.6	7.6	9.2	11.1	12.6	5.9	0.8
		R.VSIDELDWMTPETR.A	2	298.4	312.9	216.2	235.3	158.1	149.2	212.8	195.0
MSMEG _0238	MSMEG 0229	K.EIEGTPVYPSLSELPVVPDLVDVFR.R	3	52.1	62.9	46.5	57.4	43.9	85.6	29.8	55.2
		K.STGDYDIYLVNPTIK.E	2	160.0	168.6	174.0	181.1	190.2	198.8	137.1	131.4
		K.TLWLQLGLR.H	2	17.9	16.2	8.1	7.7	24.2	25.2	5.6	6.2
		R.ASYFVWTYLK.S	2	642.5	524.1	20.1	21.4	763.1	785.2	18.8	23.0
		R.DGEAAGLQVVQDR.C	2	50.0	48.7	46.6	43.1	52.5	60.9	40.9	43.1
		R.FAGGLHLAGFNTGVIDSR.R	3	62.7	56.1	33.1	25.4	83.9	84.5	27.7	27.2
		R.SVAIVGASANTSR.A	2	3.0	2.2	1.4	1.0	4.4	2.6	1.4	0.6
MSMEG _0239	MSMEG 0230	K.FIGGHGTTLGGVAVDAGR.F	3	470.6	405.9	425.8	388.3	426.5	403.5	429.2	425.7

		K.FINSVQLASHLANIGDVR.T	3	43.0	36.2	11.9	1.8	100.5	80.8	23.9	19.2
		R.FGIDTTFVGGTDPADYAAAVTDR.T	2	65.6	79.0	24.5	39.0	100.6	92.5	24.6	25.9
MSMEG _0255	MSMEG 0246	K.AYFIAAAFPSAC#GK.T	2	615.1	598.0	49.7	56.1	693.7	645.0	69.6	53.1
		K.HQGLLAWVQEVAELTQPDR.V	2	41.3	48.8	1.8	2.1	38.3	159.8	0.6	3.2
		K.HQGLLAWVQEVAELTQPDR.V	3	187.4	239.9	24.9	34.1	199.8	289.0	26.2	30.7
		K.LPTGLKDEFDALK.H	2	309.7	304.1	274.2	316.0	414.0	419.3	218.3	300.9
		K.LPTGLKDEFDALK.H	3	367.6	330.4	41.2	30.7	486.0	446.8	42.9	49.0
		K.QPNSYLALSDPSDVAR.V	2	22.9	27.7	15.2	4.9	48.5	58.2	40.2	13.6
		K.TDDGDVWWEGLEGDPQHLIDWK.G	3	118.2	185.5	8.0	24.9	114.4	177.2	7.8	15.8
		K.TIAAGNTVFTNVAK.T	2	238.7	187.9	138.7	133.6	341.6	172.2	145.8	142.5
		K.TNLAM*LQPTIEGWR.A	2	74.5	76.0	7.0	7.2	69.6	76.8	6.9	5.2
		K.VFFVNWFR.R	2	206.1	243.7	17.9	22.3	333.3	277.0	30.7	19.1
		R.AELPLIEEWFEFVGEK.L	2	65.8	89.9	10.0	14.1	63.7	105.9	5.5	15.0
		R.AELPLIEEWFEFVGEK.L	3	13.2	18.2	0.8	0.6	13.8	18.8	0.7	1.0
		R.AETVGDDIAWMR.F	2	235.6	241.3	22.6	31.5	293.3	254.6	26.6	25.0
		R.DWQHGVFIGATLGSEQTAAAEGK.V	3	340.1	323.2	21.0	38.4	449.1	398.0	27.2	63.1
		R.EIDAGPTNNWM*DPAEM*R.G	2	15.5	13.8	0.7	1.0	10.3	13.3	1.3	3.7
		R.EIDAGPTNNWMDPAEMR.G	2	169.8	160.8	5.2	3.8	147.2	159.1	1.3	3.7
		R.EIWSFGSGYGGNALLGK.K	2	440.7	433.0	145.7	156.6	636.5	510.0	159.1	153.8
		R.FLWPGFGENSR.V	2	320.1	326.5	31.7	40.0	459.1	418.6	50.9	40.2
		R.IASAMAHDEGWLAEHMLILK.L	3	173.2	162.6	44.2	52.4	202.8	170.3	30.5	31.5
		R.LC#AHLVEAGTFQK.L	2	88.4	67.5	15.6	9.2	110.9	83.9	3.0	4.0
		R.LC#AHLVEAGTFQK.L	3	129.5	105.7	21.9	22.7	162.8	138.4	12.2	14.2
		R.LYATNPEFGFFGVAPGTNWSSNPNAMK.T	3	187.5	246.0	11.9	21.6	89.0	215.4	4.1	16.7

		R.VVFADGSDEEYER.L	2	25.0	19.6	2.1	0.6	18.2	25.9	0.4	0.6
MSMEG _0312	MSMEG 0301	K.ALVEGGLPVIELTLR.T	2	13.8	13.0	155.6	143.7	7.0	10.5	130.8	116.0
MSMEG _0317	MSMEG 0306	K.VTTNEESVESQVAAASDE RDR.I	3	36.1	26.5	43.4	40.5	117.2	144.5	89.2	113.7
		K.YSSLYEDDADASVTAR.A	2	11.8	13.2	11.1	15.8	20.8	22.5	9.0	10.5
MSMEG _0356	MSMEG 0347	K.FSGQGLAGELVSAGLSDA K.A	2	24.6	29.9	12.9	19.2	8.2	9.2	9.7	8.0
MSMEG _0363	MSMEG 0355	R.TAVLAFLTYIDR.N	2	8.7	8.8	13.0	10.4	1.8	3.1	8.9	9.9
MSMEG _0370	MSMEG 0363	K.FGVIDTIDNAMVR.D	2	31.8	26.0	47.5	38.6	23.6	26.4	34.8	43.4
		K.LRPSDYIFVGGTEAK.V	2	7.3	8.9	6.0	8.5	14.1	8.5	6.6	6.3
		K.SDPQFAQQALSAFR.K	2	44.2	45.7	41.1	42.3	36.4	40.3	25.5	30.6
		K.SSLLDAISLAFLPSHR.R	2	9.3	9.2	9.9	10.2	9.8	6.9	2.1	3.4
		K.SSLLDAISLAFLPSHR.R	3	10.3	9.7	0.8	1.2	9.2	7.6	1.1	1.0
		K.SVGGLEQFVR.E	2	19.6	16.0	13.5	13.0	11.1	12.0	8.5	12.9
		K.VAALQADLAAAEDVYAAA R.K	2	19.9	17.9	5.9	9.7	18.8	19.0	5.9	10.1
		R.LQDELDLLVSDNPDVEK.L	2	17.8	18.3	1.1	0.8	15.9	14.4	1.2	1.3
		R.VNTGLGAVEFNR.G	2	26.2	26.2	6.3	6.8	15.0	19.1	8.1	6.2
		R.WAAGGYDASVFR.E	2	3.7	4.2	19.6	15.2	3.7	6.8	16.8	12.3
MSMEG _0371	MSMEG 0364	R.AVVGALPFVTR.S	2	55.7	62.4	143.9	146.8	54.2	54.7	99.9	104.5
		R.HYAAVGGDHNPIHTNAIA AK.L	3	0.3	0.1	0.2	0.1	1.1	0.4	0.2	0.4
		R.HYAAVGGDHNPIHTNAIA AK.L	4	2.9	1.6	1.1	0.3	5.1	3.9	3.0	2.1
MSMEG _0372	MSMEG 0365	K.AGMIGLAEALAPVLADK.G	2	251.8	216.8	173.9	152.4	124.5	107.7	144.4	117.4
		K.ELYTFFTPLLR.N	2	114.8	103.5	66.9	74.5	94.5	82.9	97.7	79.3
		R.GIGATIAEVFAR.D	2	137.9	119.7	50.1	51.0	87.8	73.5	70.6	59.6
		R.TALADDYNLVSNNIGGR. W	2	239.3	228.2	246.4	234.3	178.9	161.1	423.3	372.2

		R.TALADDYNLVSNNIGGR.W	3	28.9	25.6	59.0	27.5	125.6	92.7	806.1	480.2
		R.VVVVGTTPAEAGSVHAQVVQR.A	2	36.4	18.5	14.9	4.0	16.8	17.7	16.7	13.4
		R.VVVVGTTPAEAGSVHAQVVQR.A	3	1472.1	1232.5	1112.4	1040.3	921.2	984.3	1218.0	1278.0
		R.YRPGDPPLAGSLLIGGSGR.V	3	75.8	63.8	31.2	32.4	42.6	43.1	25.3	25.9
MSMEG_0373	MSMEG0366	K.LNVNGSSLAAGHPFAATGGR.I	3	135.7	159.2	127.3	104.3	111.1	122.6	157.0	153.0
		K.LPASLGVEIPVNSEPR.T	2	348.1	353.9	500.8	558.4	201.6	200.6	380.5	380.9
		R.FNLAGEQLGAVIGGAVLK.H	2	232.5	207.3	202.5	177.7	138.6	129.1	248.8	220.7
MSMEG_0380	MSMEG0373	K.VSNQTNAFTSC#LLK.A	2	53.0	80.4	54.0	61.9	57.8	76.8	43.1	46.7
MSMEG_0384	MSMEG0377	R.DILVITTPADAPAFR.R	2	32.9	37.8	323.7	314.1	50.0	55.8	331.9	321.6
MSMEG_0386	MSMEG0379	K.WTLDDGLPALLDWIAK.S	2	16.1	23.5	25.3	31.1	9.4	10.9	10.6	18.1
MSMEG_0387	MSMEG0380	R.SVTMTADDSVPEETSIGR.I	2	93.2	93.9	20.8	24.1	68.0	85.3	22.0	30.6
MSMEG_0388	MSMEG0381	R.DLGLDWPADALTMIGMTR.L	2	25.1	26.1	31.9	39.6	47.2	57.6	40.5	47.4
		R.LDGDLYESTIQALDGLYER.L	2	56.4	44.2	57.2	63.8	77.0	97.8	62.8	71.0
		R.LHLAAPILAVSEQDVR.A	2	38.2	42.6	67.9	69.7	79.7	73.8	80.6	81.1
		R.LHLAAPILAVSEQDVR.A	3	89.6	91.1	134.5	131.4	178.7	174.0	171.0	166.8
		R.VWLC#DSFAGVPAPDAAK.Y	2	41.1	40.8	67.7	70.3	50.8	65.6	106.0	114.8
		R.VWLC*DSFAGVPAPDAAK.Y	2	37.4	37.6	67.7	70.3	49.2	65.1	106.0	114.8
MSMEG_0391	MSMEG0384	K.TFYLLDTFAGLDPR.Y	2	69.7	66.7	101.5	92.0	74.4	73.3	105.9	107.2
		R.LSPGAFVLLDDYANR.G	2	146.9	148.3	247.8	263.9	190.2	191.8	276.8	280.5
MSMEG_0393	MSMEG0386	K.NLDALESVVDR.V	2	18.1	22.8	55.1	58.0	24.5	34.1	51.2	58.0
		R.FDVFLTEVAR.V	2	0.8	2.2	50.4	42.6	4.1	4.2	74.6	60.8

		R.TFRPATYTGLDLNSDGINFC*R.R	3	7.4	6.4	76.6	93.8	12.4	14.7	60.1	71.5
		R.VLRPGGYFLYTDAR.P	3	4.7	9.2	34.5	34.1	10.1	5.1	23.5	16.1
MSMEG_0394	MSMEG0387	R.GLDWLAAPK.R	2	10.9	7.0	22.5	15.9	55.2	45.1	29.3	27.3
		R.GSSPIEGTQVSAEDSAVDGK	2	5.5	4.3	15.3	13.0	8.6	10.1	17.4	21.8
		R.GSSPIEGTQVSAEDSAVDGK	3	0.8	0.6	2.6	1.3	1.3	1.8	2.2	3.7
		R.VPLFFMLMPR.G	2	0.4	0.5	1.6	2.1	5.8	1.5	3.9	2.1
MSMEG_0400	MSMEG0390	K.AGAAYVPVDPAVPAAR.V	2	106.8	101.5	138.5	130.6	170.5	154.2	158.1	135.9
		K.DAAPVAAVSSADLC#TR.L	2	28.7	31.5	21.0	22.9	36.4	42.7	22.7	25.8
		K.DAAPVAAVSSADLC#TR.L	3	6.3	6.5	0.9	1.9	11.0	15.8	0.3	1.4
		K.DAAPVAAVSSADLC*TR.L	2	27.9	30.3	21.0	22.7	36.4	42.2	22.5	25.7
		K.IDGVVDHGLLQQAIR.Q	2	9.7	10.7	53.2	42.1	25.8	20.6	81.4	47.8
		K.IDGVVDHGLLQQAIR.Q	3	29.4	32.2	24.2	22.8	41.1	46.8	27.8	29.7
		K.LQTVVFGGEALEPHR.L	2	14.8	14.6	26.6	20.4	36.4	25.9	20.0	16.7
		K.LQTVVFGGEALEPHR.L	3	74.4	70.4	43.6	43.7	99.8	108.3	48.1	53.8
		K.NAYTPSASVPMDPAIVGR.I	2	22.2	23.3	19.2	20.0	31.5	33.8	18.7	18.5
		K.NLTHLIAVLEER.L	2	15.2	13.0	11.3	12.0	26.6	24.5	14.9	13.4
		K.NLTHLIAVLEER.L	3	11.8	9.0	8.4	7.0	18.5	18.3	6.7	8.6
		K.PAALPAVQPDTDTHASAGR.F	3	3.0	3.3	1.0	1.7	4.2	5.0	0.5	1.5
		K.TGAAYLPIDPAHPDAR.I	2	11.8	8.9	4.5	14.9	42.0	21.1	22.5	10.9
		K.TGAAYLPIDPAHPDAR.I	3	48.4	45.6	39.4	39.9	68.6	72.4	41.8	41.5
		K.YPVALTVADVPWAQVVAGEATLGAVVK.D	3	11.3	13.5	10.5	13.6	10.5	19.4	5.4	11.9
		R.ATSGVATTVTDLLGQLQR.D	2	69.6	65.1	102.2	118.1	114.9	93.5	166.2	140.1
		R.AVPDGLTYGLLR.Y	2	36.9	34.3	40.2	33.0	62.1	54.8	47.8	40.7
		R.DAAQAAWGSVLEGFETPTLVAPPAQAGPR.G	3	33.3	47.9	41.0	51.4	22.9	52.4	11.6	38.7

R.DHNDTVDHEHM*ALNEIHR.L	4	3.6	1.7	2.8	0.9	2.9	3.0	1.0	1.3
R.DHNDTVDHEHM*ALNEIHR.L	5	3.0	1.2	1.7	0.6	2.4	2.3	0.5	0.8
R.DHNDTVDHEHMALNEIHR.L	3	2.9	2.1	2.4	2.0	8.0	4.2	5.0	3.6
R.DHNDTVDHEHMALNEIHR.L	4	10.7	10.9	8.8	9.9	12.0	15.8	6.9	8.9
R.DHNDTVDHEHMALNEIHR.L	5	9.2	9.0	7.6	7.2	11.2	13.0	6.0	7.6
R.EYNHYPLSMVATPGHEVVLR.V	4	15.7	18.8	10.1	12.9	19.3	25.6	10.3	14.9
R.FILDDASPVAVVTTAELR.S	2	50.6	43.2	69.0	52.2	83.6	76.4	74.1	55.8
R.FILDDASPVAVVTTAELR.S	3	5.9	7.4	6.5	13.2	9.8	13.3	4.4	12.7
R.FVAC#PFGAPGAR.M	2	13.4	15.5	8.9	10.0	18.6	22.1	5.3	10.0
R.IELGEVQSALAALDGVESAAAIM*R.E	3	11.7	14.8	8.4	13.3	11.8	16.5	10.6	11.0
R.ILLEDLNIAWAQHHSGQQVQLPAGGTSFLR.W	4	21.2	20.8	31.0	26.5	25.6	29.4	30.5	31.1
R.ISTIYTSLVTGK.P	2	36.3	33.9	19.1	17.9	19.7	22.5	8.2	7.1
R.ISTIYTSLVTGKPVPEAYFGSLR.D	3	16.6	16.8	14.3	11.3	35.4	39.0	18.4	20.0
R.LADHAHTAEVTAQADAWR.E	3	13.7	12.8	11.3	9.3	21.3	22.7	10.0	13.9
R.LADHAHTAEVTAQADAWR.E	4	14.2	14.7	8.9	8.1	16.6	22.6	5.8	8.7
R.LATIGNHSALSESAPDAVSIPER.F	3	91.3	110.9	111.5	125.4	130.2	131.8	91.2	90.9
R.LAVNFIPSR.L	2	35.0	33.3	30.7	31.6	51.5	50.3	26.6	29.3
R.LGAGAGELSEELWR.I	2	133.8	148.5	136.4	141.3	154.6	200.5	127.3	146.0
R.LGAIVAAMAADPAMPLAAVR.L	2	14.5	12.7	469.5	396.6	18.8	18.6	646.2	409.5
R.LLM*VPDAVVR.S	2	7.9	8.2	47.2	41.9	13.8	13.1	54.0	47.7
R.LPPYMVPTAIVVLPALPLTSSNK.L	3	18.0	26.3	13.1	22.3	19.9	29.5	5.7	15.5

		R.LSGWMHAHPGMPR.M	3	3.8	4.3	7.1	4.1	4.2	5.6	1.8	1.3
		R.LSTIDLLDPQEHDRLDEWGNR.A	3	10.8	11.2	12.3	11.9	26.0	23.1	10.3	10.2
		R.LVGYITGTADPVDIR.A	2	71.1	69.8	142.5	126.1	98.3	101.7	172.9	135.3
		R.NFVTWLGAQDR.D	2	31.9	25.8	27.4	23.9	40.2	40.8	19.3	21.6
		R.PAPALESIPASFAESVR.R	2	59.1	52.3	97.4	81.4	83.6	75.7	106.7	88.4
		R.PAPALESIPASFAESVR.R	3	7.8	7.7	2.8	5.3	8.4	12.1	0.7	3.1
		R.PVDLAGADSMVGLLINTVPVR.A	3	9.5	10.0	9.4	9.4	6.3	7.6	3.6	5.5
		R.SVIAILGAFK.A	2	23.7	19.5	19.3	15.5	42.1	35.2	25.5	19.1
		R.TGEAVVTILAILK.T	2	3.0	3.1	1.4	1.8	16.2	10.8	6.5	3.5
		R.VAEAPDAAALTFEGR.T	2	71.7	74.0	84.3	86.0	118.4	107.4	101.4	94.4
		R.VQDSDGEWSLLVPDAATVDAR.D	2	14.3	14.0	11.7	12.0	21.5	24.5	5.8	12.0
		R.WGADGQLQYVGR.A	2	26.4	30.5	44.1	50.4	31.0	42.2	41.7	52.3
MSMEG_0401	MSMEG 0391	K.YPVALDFDR.N	2	33.9	31.2	26.0	29.7	51.3	53.5	31.4	30.4
		R.ALPAPEYQDADQYR.A	2	79.2	79.6	73.3	72.3	105.0	106.4	76.6	70.6
		R.ALPAPEYQDADQYR.A	3	4.7	6.9	3.2	3.1	3.9	9.8	0.5	4.2
		R.AQLNPATGSMLTAAWVPETR.Q	2	13.5	13.0	64.5	67.4	20.0	21.4	70.7	62.4
		R.AQLNPATGSMLTAAWVPETR.Q	3	14.9	21.3	12.6	22.6	19.7	26.2	6.5	17.3
		R.AVESFQVSAETTQALGELAR.S	2	13.6	12.6	2.1	2.2	19.5	17.2	5.5	3.5
		R.EYNHYPLSVQAVPGHELGLR.V	3	28.9	26.8	54.8	45.4	48.8	45.1	60.6	53.9
		R.FVSWLAEQDNDAAR.A	2	186.7	166.0	151.4	132.7	140.4	139.5	118.9	110.9
		R.GVFSGFETPTLVGTPGLALGR.R	2	81.2	75.2	64.9	56.6	98.1	92.1	66.4	60.7
		R.ILLEDLNIAWAQHR.S	2	5.5	3.1	4.3	4.3	21.6	10.4	7.9	4.1
		R.ILLEDLNIAWAQHR.S	3	22.7	21.2	17.2	19.4	45.0	40.0	21.3	21.1
		R.LDPDAVSLTAAGGR.V	2	39.3	34.4	28.6	25.2	31.3	37.4	16.1	18.6
		R.LGAGAADLSDDLWR.L	2	141.8	151.7	40.3	41.5	147.9	174.5	36.1	50.0
		R.SDIDAQIAQISAAER.A	2	41.7	48.1	46.1	45.0	58.6	59.1	45.2	44.1

		R.SGQPVVLPTPGTSFGR.W	2	87.0	87.1	93.5	89.8	129.9	124.1	97.2	83.8
		R.SVPDGLTYGLLR.Y	2	31.7	29.9	23.9	22.9	48.1	47.3	21.3	25.3
		R.TADVLATADVWR.T	2	26.9	24.5	36.8	34.5	33.4	38.6	36.2	40.3
		R.TEELFGDADLSR.T	2	56.5	57.0	55.1	55.7	81.1	75.3	52.9	50.1
		R.YLNPDVELDNPDPTIGFNYLGR.L	3	26.0	26.4	18.4	21.6	19.1	39.7	8.1	22.2
MSMEG_0402	MSMEG0392	K.GATFVLDEWLR.P	2	5.3	4.6	1.4	1.9	5.0	4.0	1.5	1.7
		K.GQPNLGLDEQTWQR.L	2	29.1	30.0	13.9	14.6	27.2	30.8	9.1	13.4
		K.LDGVDQAVVIAR.E	2	67.1	59.0	38.1	41.4	73.4	66.1	39.8	37.3
		K.LKPFNFVSTSDVGR.Q	3	23.5	24.3	31.2	30.6	23.9	27.0	21.0	20.7
		K.PGATVAFLIPR.S	2	17.1	16.2	14.4	15.6	23.7	20.5	17.6	18.0
		K.TGAAYLPIDPSVPAAR.L	2	84.4	84.6	76.7	69.5	91.9	90.4	79.5	73.9
		K.TGAAYLPVDPAHPDAR.I	2	30.3	23.7	65.1	45.8	34.4	29.1	49.5	44.5
		K.TGAAYLPVDPAHPDAR.I	3	32.8	33.1	22.0	23.0	37.5	38.6	19.6	24.1
		K.TLAGIFAHVLGVER.I	2	5.2	4.6	2.2	2.2	15.8	10.3	8.5	5.5
		K.TLAGIFAHVLGVER.I	3	2.8	2.3	0.6	0.5	7.2	5.7	2.8	1.7
		K.VSADTTVGDLLR.Q	2	31.6	28.8	32.8	32.1	36.1	34.5	21.7	21.9
		K.WAGEVLLR.E	2	16.9	13.9	0.3	0.0	1.8	2.3	0.4	0.1
		K.YPVALTVENLPWEQVVAGDAALGGIVK.A	3	31.2	35.6	24.9	26.9	19.8	36.0	12.4	23.5
		R.APSTPAEQILADVYAQVLGVER.V	3	12.4	15.8	10.8	14.8	12.0	14.9	6.4	10.3
		R.ASWTWAPSAFDEEQATR.L	2	28.5	30.2	24.5	24.8	36.6	39.2	17.5	25.7
		R.AVLTDPGPTPVTIPALFAEQVVR.A	3	21.9	26.7	17.6	23.9	23.5	33.4	17.4	23.3
		R.AVLTQPVDSPQSIPEVFAR.Q	2	26.6	25.3	16.9	17.0	32.1	29.9	13.8	16.3
		R.EVLDGFETPTLVAPPGVPGPR.G	2	27.4	26.5	24.3	42.3	39.5	35.4	73.1	24.6
		R.FEAALQGLSDQQR.Q	2	41.8	43.8	37.1	37.9	43.7	48.7	36.6	41.2
		R.GDDLTPDHEIEQLC#AAER.S	3	12.8	14.7	13.0	17.0	16.8	19.6	12.9	14.9
		R.IEDFGEWLQR.F	2	15.4	14.4	10.3	9.8	16.3	16.4	10.4	8.1
		R.IELGEIQAALAK.L	2	34.5	31.8	22.7	30.9	48.9	41.9	35.4	37.9

		R.LEATFDTDPLLR.K	2	70.7	60.1	89.1	67.6	67.0	59.1	91.1	68.8
		R.LEFMLGDAKPVAAVTTGDLR.S	3	27.7	30.9	73.1	58.3	34.8	30.9	90.1	66.7
		R.LLEAMIDDPAR.R	2	11.1	12.1	160.1	178.9	8.9	11.7	75.1	70.4
		R.LVGYITGTADPAAAR.S	2	60.1	65.8	51.5	53.9	62.6	67.1	46.3	50.7
		R.LVGYITGTADPAQLR.T	2	48.2	50.8	43.1	46.5	62.8	56.8	45.1	49.0
		R.LVVVPESVGSSPDDLHNLLITEK.V	3	130.3	130.3	120.1	111.6	154.7	161.1	120.4	120.5
		R.NFVNWLATQDATAAK.A	2	45.1	46.1	50.7	52.1	68.9	61.6	63.9	61.1
		R.PADLAGAESMVGLLINTVPIR.A	3	20.0	19.0	13.1	15.2	14.0	15.8	9.9	11.2
		R.RLEATFDTDPLLR.K	3	3.4	2.9	4.2	3.3	5.2	6.4	7.3	5.9
		R.SADAIVAILAVLK.T	2	4.4	3.5	2.5	2.1	11.0	6.8	6.8	4.2
		R.SGEAILSILSVLK.T	2	16.0	15.6	12.3	11.7	20.5	15.9	14.2	10.5
		R.SLPEGLTYGLLR.Y	2	25.0	23.4	12.6	11.6	29.1	30.0	10.5	11.1
		R.TATTLPRPDGAVQTVLLTGATGFLGR.Y	3	42.9	57.3	57.5	50.8	61.6	60.3	64.4	55.5
		R.TVDALTDDVLVEAR.N	2	35.1	34.6	34.1	30.2	42.6	44.4	33.4	29.5
		R.VAVAAGESHVVDEGVGPVVATPIMR.W	3	110.4	107.8	63.4	68.5	103.6	82.0	59.1	61.6
		R.VEYDTSVFDVSEIETLIDR.F	2	77.3	70.8	101.3	93.0	65.6	64.3	83.4	81.9
		R.VSFAAVHGSDVTEVHAR.D	3	5.8	5.4	5.7	3.2	9.4	7.1	5.7	5.2
		R.VSFAAVHGSDVTEVHAR.D	4	13.7	11.1	7.3	5.8	14.5	15.0	5.3	6.3
		R.YDELASDVDLSR.T	2	40.5	36.8	32.5	29.3	32.5	39.7	23.8	27.6
		R.YDVTDVLPLTPVQQGLLFHSTFAR.G	3	51.6	47.3	44.2	40.1	56.7	60.2	51.0	46.2
		R.YLLLEWLR.Q	2	7.3	6.4	3.1	3.0	10.9	8.4	5.2	3.3
		R.YLNPEVDLGGSDPTIGFNYLGR.L	2	12.5	9.9	6.4	5.3	10.0	14.8	3.6	4.8
		R.YLNPEVDLGGSDPTIGFNYLGR.L	3	20.0	19.4	15.5	18.7	21.2	22.8	16.1	16.0
MSMEG_0406	MSMEG0396	K.AIATLSTGYLNALEYAK.E	2	105.0	109.8	172.1	160.7	36.7	45.9	99.7	87.0

		R.DKGQALAYVAGEIEQFIK.N	2	3.5	4.4	32.1	32.9	1.5	1.0	21.7	25.8
		R.DQVFNLFEVFGVDK.V	2	58.6	60.7	127.5	140.1	34.1	35.8	124.4	127.5
		R.VNDLLLPIVK.G	2	23.8	21.9	70.0	63.3	5.3	3.2	34.6	28.7
MSMEG_0408	MSMEG0398	K.DLESAVATLVADGVR.C	2	6.1	7.8	5.2	4.9	8.8	10.2	4.3	4.9
		K.DLESAVATLVADGVR.C	3	1.3	1.6	0.2	0.1	2.3	2.7	0.1	0.1
		K.TFDASADGYVR.S	2	2.1	1.3	0.2	0.1	1.2	1.9	0.0	0.0
		K.VLGSARPPILDLVLPSAAGEATGDSELLK.Q	3	30.2	33.0	28.3	33.9	37.7	37.4	38.1	38.9
		R.AGFVTEFLQR.E	2	8.9	9.4	1.1	1.2	11.2	10.3	0.4	1.7
		R.ELFDTEPVFAETVR.K	2	17.7	17.6	43.0	45.8	22.3	24.4	49.8	51.1
		R.ELFDTQPVFAQTVR.Q	2	41.9	26.2	66.0	63.4	35.5	25.7	55.1	60.6
		R.ESAIELLGALADDRPAPGLVR.G	3	11.0	12.3	3.8	6.1	24.0	17.5	9.3	7.7
		R.FPGAASAEEYWALLR.D	2	8.3	7.9	5.0	3.7	9.3	11.6	3.7	4.7
		R.GAGFSGVVWR.C	2	2.5	2.1	2.3	1.1	2.3	4.2	0.2	0.1
		R.IHTYAADVGDEAQVADLLAR.I	3	12.1	14.8	2.9	4.0	18.1	19.6	4.0	5.6
		R.KLDLPTYPFEHR.Q	3	4.7	4.1	0.3	0.4	5.8	6.0	0.2	1.1
		R.LEAEITNLLSAVHAAQGPSVK.L	3	18.2	18.1	16.1	20.8	26.9	25.6	21.9	21.8
		R.LKDVFFYEPIILPEK.A	3	5.2	6.0	6.1	8.2	8.6	9.1	6.5	7.8
		R.LSDAADYLLVEVLGLGEQTNVR.Q	3	5.1	5.8	1.2	1.0	8.6	8.4	3.1	2.6
		R.LTETDVPEPDEGYVQVR.V	2	15.8	17.5	10.6	11.5	17.2	19.6	12.2	8.9
		R.PLLDVLFATDR.E	2	8.3	6.8	1.2	1.0	9.9	7.3	1.3	0.8
		R.SPIWLVTR.D	2	5.0	4.5	3.0	7.9	5.2	7.1	5.6	3.9
		R.TGAVLTGQTPLDAQYWR.R	2	11.3	10.6	2.7	2.5	12.0	14.8	1.9	2.0
MSMEG_0415	MSMEG0405	K.DLPSLGISVLGEAHDTAAR.T	2	31.8	0.0	4.6	0.0	25.8	27.1	3.5	5.1
		K.LKDLPSLGISVLGEAHDTAAR.T	3	28.4	29.1	13.1	13.5	43.3	44.5	13.8	13.7
		R.VSDIVVNDDVAPIVFHR.S	2	10.1	10.0	17.5	21.0	31.6	18.2	17.8	18.8

		R.VSDIVVNDDVAPIVFHR.S	3	134.5	129.8	48.8	59.4	136.8	140.6	47.8	47.9
MSMEG _0422	MSMEG 0413	K.SDGENMAFEDLLTR.V	2	62.1	68.3	174.8	170.3	55.0	59.6	161.5	173.1
		R.AAADAAGVHVVINAR.T	2	5.4	5.3	30.4	24.0	5.1	4.9	19.4	13.5
		R.AAADAAGVHVVINAR.T	3	18.3	16.5	64.9	58.3	28.7	23.5	52.2	47.1
		R.FTSAVSLPVSVTVPPEDTDK.A	2	7.3	6.7	51.6	53.2	2.3	5.2	8.0	14.0
		R.ITFGPFLQWALGAQAK.E	2	44.6	40.3	224.7	203.8	72.0	69.3	188.0	190.4
		R.LAVDAGFAALTVGSHPVADSIGK.S	3	87.5	96.1	286.9	286.7	104.8	111.6	277.0	270.8
		R.LIEGLLDAGVVGFNLEDTVHK.E	3	11.7	12.0	87.4	85.6	16.7	14.2	59.9	53.3
		R.MSEAAAAGADSLFPAGLR.D	2	29.6	30.2	126.7	132.5	36.2	52.2	93.1	103.3
MSMEG _0456	MSMEG 0447	K.LIPGPDWGSGASVVGTAGLR.E	2	66.5	62.8	171.1	171.6	85.5	78.9	97.9	39.4
		R.VDPASVNPQGVAGNGVAGVK.L	2	94.0	104.1	19.6	29.9	102.1	145.0	11.9	29.4
MSMEG _0457	MSMEG 0448	R.ILFDPAIAPDSTLDIGEVLLR.A	2	40.4	45.6	3.3	5.1	39.9	60.1	1.9	6.2
		R.LRPAVNLGLDVLNTALR.E	3	28.2	32.1	10.6	13.1	36.9	33.7	11.0	12.3
MSMEG _0464	MSMEG 0456	K.GGVEFPGSDAVDVLFDR.D	2	109.5	114.8	96.8	94.8	95.2	96.9	81.6	81.8
		R.EILPLATVTTPNLFEAR.T	2	140.7	137.4	171.7	173.9	117.2	122.9	175.3	144.0
		R.TLSGMDEITTVDDLIEAAR.R	2	47.5	47.0	41.2	38.7	52.0	49.3	50.7	46.2
		R.VAGAGC*TLAAAITAELAK.G	2	38.5	38.4	86.9	85.4	39.6	41.8	63.3	58.5
MSMEG _0530	MSMEG 0520	R.GIGAEVAQQLATSGTHSIVNYR.A	3	32.8	45.5	266.4	289.2	78.2	71.4	284.2	273.1
		R.LDALVLTALGGPR.V	2	2.9	4.0	11.3	9.9	9.9	10.8	18.4	13.3
MSMEG _0531	MSMEG 0521	K.IFTIFEGTSEIQR.L	2	159.1	156.8	53.2	49.7	147.7	118.3	60.0	61.7

		K.LGAFC#SSEPDAGSDVGAIR.T	2	103.4	108.5	139.7	141.3	99.1	106.0	94.3	104.5
MSMEG_0550	MSMEG0541	K.VVDVYSQPEWTALVVAK.D	2	224.6	219.1	141.2	152.2	255.3	261.2	105.4	104.0
		R.EDFSAAHPDTVQAVVNSYEEAR.T	3	193.7	208.7	180.8	212.6	386.9	410.9	106.5	126.7
		R.LDYAYYNPLSLVVR.D	2	144.4	165.4	146.1	173.0	171.7	169.7	80.1	89.1
		R.NPEFNSYGVLNVR.E	2	87.9	98.5	95.2	96.3	119.6	117.2	67.0	72.0
		R.SNALDFGSTAGSAALVAR.A	2	130.0	139.1	118.0	124.9	213.1	206.3	68.0	73.7
		R.TWALEHPAELTTLLAQEAK.I	3	80.1	81.3	78.6	70.8	124.7	110.3	39.5	32.8
MSMEG_0551	MSMEG0542	R.VLLLDEPLAALDALTR.L	2	36.7	37.5	18.5	18.7	53.6	56.1	17.7	18.9
MSMEG_0572	MSMEG0563	K.GFESAIYFYGPGSINC*LATR.G	2	13.3	18.8	145.8	160.1	18.8	17.6	148.3	144.0
		K.SSLAEIPHPALPK.G	2	1.5	1.9	1719.2	2141.9	0.9	1.8	1944.1	1508.1
		K.TGDSGFPGEQNINDALATFIK.E	2	10.0	13.1	1049.9	1126.6	8.3	10.2	919.4	820.3
		K.TGDSGFPGEQNINDALATFIK.E	3	0.9	1.0	352.9	276.7	1.1	1.0	292.5	273.0
		R.EEDLIEGVIPAHPLDVQDALIYYAR.K	3	45.2	46.2	542.1	648.3	40.0	49.2	201.8	260.6
MSMEG_0599	MSMEG0590	R.EVIFLDELPR.N	2	6.7	12.2	27.3	26.8	9.9	9.4	32.9	33.2
		R.IIMLNTEFSGPQIK.E	2	19.2	21.9	140.7	157.3	27.9	20.8	132.2	118.7
MSMEG_0603	MSMEG0594	R.DGQFDQIEQNHAGQLIAEGHAAK.V	3	18.6	16.7	54.4	43.1	31.5	39.3	49.5	35.3
MSMEG_0613	MSMEG0603	R.LDGIALLITTGFLPQR.F	2	1.2	0.8	4.0	2.6	0.5	0.5	6.7	21.3
MSMEG_0614	MSMEG0604	R.LPWADGTVVYELDVPK.V	2	87.6	98.7	32.4	34.0	25.1	28.0	15.7	24.0
MSMEG_0628	MSMEG0619	R.YVAEAIDAGTQIFK.A	2	69.2	74.4	78.0	98.7	58.9	64.3	72.6	81.0
MSMEG_0636	MSMEG0627	K.NWDEFADTLTEDVR.G	2	91.7	60.0	27.8	19.3	119.3	135.9	21.7	24.4

		R.GDYGQSLGEALHFTDR.A	2	71.4	80.2	12.6	16.7	85.0	78.7	7.8	5.3
		R.GDYGQSLGEALHFTDR.A	3	65.8	62.6	20.4	17.1	94.4	93.3	11.6	12.6
		R.VAHPEITVDGDEATGTWYLQDR.V	3	185.6	208.9	67.7	75.7	230.1	254.9	44.9	48.8
MSMEG_0640	MSMEG0632	K.SVTAQTLIGLTR.A	2	11.0	10.3	49.3	46.7	19.5	15.8	20.3	27.9
MSMEG_0643	MSMEG0635	K.DAGADEVADAHVDK.I	2	1.7	1.1	10.0	5.1	9.0	6.6	9.8	8.1
		K.DAGADEVADAHVDK.I	3	3.6	2.2	19.9	12.4	12.1	10.9	17.2	18.9
		K.GEEGAEVAPGLAEDMPEVSPDGK.T	2	43.9	52.5	385.9	374.5	25.5	63.1	166.6	241.5
		K.MNELLTQQLTDETK.K	2	109.1	104.5	676.8	668.9	262.0	293.9	666.1	739.3
		K.MNELLTQQLTDETKK.G	2	11.3	13.4	94.2	79.5	24.0	27.1	85.3	95.7
		K.MNELLTQQLTDETKK.G	3	29.1	35.9	201.6	186.5	39.9	39.4	156.6	190.8
		K.TDFM#HDPPDADRLPEVK.A	4	3.3	4.1	26.6	28.5	0.7	2.3	2.9	10.3
		K.TDFMHDPPDADRLPEVK.A	4	9.3	9.7	99.1	92.0	5.0	8.6	27.9	32.4
		R.AYMEQAVWAPYGNEQLATFLSDR.M	2	3.7	8.2	47.2	64.1	1.2	7.9	14.6	36.4
		R.DITVWTDDEPDR.K	2	35.8	40.4	339.1	263.9	17.4	20.6	124.7	109.1
		R.IGEYYHDVLTQLGFNATLK.V	2	16.0	13.6	104.6	102.7	16.5	16.4	71.4	88.4
		R.IGEYYHDVLTQLGFNATLK.V	3	144.5	151.1	746.5	722.5	323.8	297.2	724.1	652.1
		R.QAVNYAIDPEALNR.V	2	23.0	30.5	230.1	225.2	40.9	46.3	197.2	221.7
		R.SYHHVLFNQDFTSFALK	2	2.3	4.0	93.2	100.3	4.2	5.5	49.9	87.7
		R.SYHHVLFNQDFTSFALK	3	34.8	31.5	312.3	281.7	104.2	100.7	264.9	243.2
MSMEG_0683	MSMEG0675	R.HDDLASALAATDLDEDDLAG	2	21.0	24.7	32.4	42.9	16.8	20.9	29.1	35.2
		R.WAEAAGISVTPGR.M	2	10.3	11.8	14.8	19.4	10.0	9.9	11.1	13.0
MSMEG_0688	MSMEG0680	K.AIVVINPNNPTGAVYSR.E	2	72.7	69.1	307.2	370.6	71.6	59.1	160.9	248.7
MSMEG_0690	MSMEG0682	K.AEAAAPPVEEAAPEAEAPAAPAAGGAEAK.P	3	26.9	36.5	31.7	43.0	11.9	20.8	13.7	26.7

		K.AEVLDVAQLLLNSLDTSK.V	2	39.9	38.7	44.4	44.2	40.3	38.7	39.6	36.4
		K.AEVLDVAQLLLNSLDTSK.V	3	17.2	17.7	20.7	19.4	17.9	16.4	18.5	15.7
		K.APYILGEKPSPLESTPEGGLGEK.A	3	96.5	108.5	135.8	154.9	111.2	115.7	120.6	134.2
		K.AVAELLATAGVK.F	2	27.4	26.7	25.1	26.4	35.7	31.8	21.6	23.4
		K.FLVLGTGETC#TGDSAR.R	2	32.6	37.5	47.4	52.2	38.4	39.8	39.5	40.9
		K.FLVLGTGETC*TGDSAR.R	2	32.6	37.5	47.3	52.2	38.4	39.8	39.5	40.9
		K.GYLDFTTC#TEC#GR.C	2	27.3	26.7	31.0	33.4	31.1	29.9	28.5	27.0
		K.GYLDFTTC*TEC*GR.C	2	27.3	26.7	31.0	33.4	31.1	29.9	28.5	27.0
		K.KTAAAAPAEKPAATEAPEASATPAAPAAPVK.G	4	0.7	1.4	0.1	0.1	7.0	9.8	0.2	0.3
		K.TAAAAPAEKPAATEAPEASATPAAPAAPVK.G	3	5.5	6.6	4.8	6.7	20.0	24.5	3.4	5.5
		R.ITTQITEVFGQTR.L	2	52.1	49.2	79.7	82.2	54.4	50.8	65.7	65.3
		R.VMITDGVDDVAASR.N	2	40.2	47.8	59.6	66.1	40.5	41.5	55.0	52.9
MSMEG_0692	MSMEG0684	R.DRAPEVGELGGDGLALTGFVER.T	3	55.8	64.5	134.8	119.9	42.8	58.5	167.3	153.9
		R.GELALAEALDAMAR.E	2	6.3	5.4	10.4	8.9	5.8	4.5	7.5	5.8
		R.LRAPVVTSPQPQLVAAAGAALLADR.A	3	16.2	15.1	38.1	34.6	24.8	23.5	42.9	39.7
		R.NKPAFAPDGIPAALVPDSMAALAALK.A	3	16.1	26.5	38.9	66.9	21.7	23.2	19.3	32.1
MSMEG_0695	MSMEG0689	R.SAAEHLLLAVESELSSFNDPGER.E	3	22.0	26.3	21.5	25.8	7.3	14.5	15.9	24.0
MSMEG_0702	MSMEG0697	K.DSPGLPKPVQTPHPPIIIGGQGAK.R	3	1001.5	915.4	635.1	533.2	612.9	728.6	564.8	647.9
		K.DSPGLPKPVQTPHPPIIIGGQGAK.R	4	717.3	687.6	406.2	340.5	425.6	521.9	308.6	425.5
		K.LGPFIEAGVQR.V	2	121.8	85.4	57.8	32.2	81.4	83.0	46.9	54.3
		K.MAGDGLPGPTDSWVTLAGIAR.E	2	285.4	306.5	165.7	181.6	217.2	230.1	162.0	194.4
		K.RTPALAAQFASEFNIPFVPLDTLK.T	3	297.8	311.8	154.5	166.6	169.9	190.5	128.0	150.1

		R.LGYSAFFR.S	2	210.5	196.4	101.8	91.5	178.6	174.3	138.5	123.3
		R.SNSPLVGTSTEIVDK.L	2	92.9	73.4	34.0	32.3	47.2	47.7	30.3	31.4
		R.TPALAAQFASEFNIPFVPLDTLK.T	3	118.9	118.6	67.1	70.1	52.3	68.0	42.6	51.4
		R.VFVEPQQGATYSDQLAVAQAAER.L	2	322.5	377.4	171.6	200.1	189.3	238.4	144.1	180.8
		R.VFVEPQQGATYSDQLAVAQAAER.L	3	1185.0	1382.9	794.7	866.9	762.9	999.6	736.1	845.1
		R.VYLQVLDMSDLDHVEFFANEVVR.Q	3	62.2	48.4	40.9	34.8	13.9	20.5	12.1	20.9
		R.YPGPLAISVAQVDEM*SGGR.V	2	93.8	99.6	5.4	8.3	68.9	64.7	5.2	4.7
		R.YPGPLAISVAQVDEMSGGR.V	2	431.3	429.5	262.7	249.2	303.7	308.5	269.8	262.2
MSMEG_0703	MSMEG0698	K.GPAVTVTGAPGELLLFVAGR.D	2	14.7	15.8	82.7	76.3	9.7	10.1	57.8	65.8
		K.LLDPLVNVAEMFIHHEDVR.R	4	11.2	10.3	32.7	29.9	8.6	7.3	26.0	23.3
		R.IASGPPLLSPFK.L	2	11.1	12.1	75.4	78.1	6.6	6.7	52.3	46.4
		R.VEFTGDENSIAALR.A	2	34.1	41.4	88.5	77.8	31.3	43.2	113.6	111.1
MSMEG_0709	MSMEG0704	K.ALEGTDISAIK.S	2	10.6	11.2	14.4	21.1	10.4	10.0	17.5	25.1
		K.DAGISVSDIDHVVLVGGSTR.M	2	198.8	186.4	170.1	162.9	191.5	189.1	210.1	199.5
		K.DAGISVSDIDHVVLVGGSTR.M	3	384.5	363.5	407.4	343.2	488.7	311.7	507.9	327.8
		K.DVLLLDVTPLSLGIETK.G	2	577.4	612.7	644.3	688.6	668.3	629.9	838.3	738.7
		K.EAGQIAGLNVLR.I	2	684.3	601.0	788.1	661.0	799.4	486.6	878.3	511.5
		K.GVNPDEVVAVGAALQAGVLK.G	2	1021.5	956.0	917.7	844.2	970.5	765.9	964.9	763.7
		K.GVNPDEVVAVGAALQAGVLK.G	3	329.7	371.6	267.3	270.6	339.8	259.2	305.0	221.1
		K.LLGSFELTGIPPAPR.G	2	328.3	388.8	311.6	343.2	446.0	353.2	503.0	382.9
		K.RHVGTDWNIEIDDKK.Y	4	27.6	32.8	8.3	13.1	22.9	41.4	7.6	23.1
		R.AVGIDLGTTNSVVAVLEGGDPVVVANSEGSR.T	3	236.7	348.6	175.1	280.1	172.6	306.0	129.6	267.4
		R.HVGTDWNIEIDDK.K	2	57.1	49.8	40.3	43.0	52.1	58.0	23.2	44.9

		R.HVGTDWNIEIDDKK.Y	2	84.1	76.1	99.9	75.8	89.4	85.3	86.0	83.1
		R.HVGTDWNIEIDDKK.Y	3	530.2	464.7	476.3	405.3	440.0	453.6	410.6	415.1
		R.IVNEPTAAALAYGLDK.G	2	182.2	177.4	272.2	251.6	133.6	154.3	241.7	243.4
		R.IVNEPTAAALAYGLDK.G	3	178.1	168.7	130.9	123.7	155.8	133.3	107.8	108.0
		R.NQAESLVYQTEK.F	2	22.9	19.8	12.7	9.6	15.5	18.8	9.9	11.7
		R.SETFTTADDNQPSVQIQVYQGER.E	2	162.8	204.2	107.2	164.3	98.4	182.7	64.3	151.9
		R.SETFTTADDNQPSVQIQVYQGER.E	3	340.8	389.1	245.7	320.3	249.6	310.4	206.7	254.9
MSMEG_0710	MSMEG0705	K.ANVVSELLGVLDDLDR.A	2	65.8	71.4	62.7	79.0	40.7	49.0	41.0	49.2
		K.ANVVSELLGVLDDLDR.A	3	13.7	10.9	6.7	4.8	7.9	8.6	4.6	4.4
		R.EPAATPQGSAPASAAPETGGDSDEVTELK.A	3	185.5	250.0	158.0	199.7	100.2	161.9	85.8	128.2
MSMEG_0732	MSMEG0726	K.AHPDVFDVLLQVLDEGR.L	3	13.4	15.6	11.0	11.7	19.5	26.2	26.3	30.9
		R.ITDSALVAAATLSDR.Y	2	47.8	57.9	274.5	279.6	70.2	57.0	315.5	294.5
		R.VTSPDPEGTYQALEK.Y	2	109.0	104.4	144.0	128.6	87.4	111.2	129.4	153.1
MSMEG_0741	MSMEG0736	K.EC#LTQSAAFLAATER.L	2	62.2	67.6	51.6	57.0	92.1	81.4	51.9	53.0
		K.EC*LTQSAAFLAATER.L	2	62.2	67.6	51.6	57.0	92.1	81.4	51.9	53.0
		R.AHQHLEVYSGLPNYR.N	3	44.4	51.3	40.6	49.3	52.7	60.0	27.5	30.6
		R.EIEALGFGSLFLPETVAK.E	2	157.4	159.5	185.0	187.2	190.3	185.3	206.7	204.4
		R.FVLGLGVSHGPLVER.G	2	39.4	48.9	46.2	46.7	46.0	56.6	45.4	51.8
		R.IGVWWASDTWSMR.D	2	22.7	26.9	28.0	29.5	22.4	17.8	14.0	20.8
MSMEG_0759	MSMEG0755	K.ALEPAEVLENLLEQAEGFK.H	2	85.1	102.1	101.0	112.2	77.6	90.4	72.5	93.8
		K.ALEPAEVLENLLEQAEGFK.H	3	93.0	101.2	84.1	97.1	82.6	102.5	78.0	90.0
		R.KALEPAEVLENLLEQAEGFK.H	3	55.2	60.3	61.1	60.8	75.9	70.6	74.3	78.3
		R.VADVLDEQVLAEK.I	2	145.2	98.0	389.2	344.5	249.0	253.5	496.2	504.3
		R.VNGITDYFLTK.L	2	15.4	20.3	7.8	9.7	19.0	25.7	12.6	12.6
MSMEG_0760	MSMEG0756	R.AQDLAVSADPDAATWDK.A	2	26.3	29.5	8.3	9.4	26.5	31.4	2.7	7.1

		R.VPELPGAGSLLAPPWTVEK.F	2	33.6	34.7	95.8	83.0	29.9	37.2	96.2	100.0
MSMEG_0769	MSMEG 0763	K.YPFLQSHPQYELAK.R	3	79.8	83.1	30.9	26.7	64.5	65.0	34.3	33.2
		R.IPAPLPDGVSQATIGVR.G	2	352.4	345.8	603.6	608.7	272.0	261.6	542.1	571.2
		R.VLGGAILGDQEYIDGPVQK.L	2	214.8	223.4	223.9	266.9	136.9	154.2	208.4	230.2
MSMEG_0777	MSMEG 0772	K.GASIYDVPEGGIPVYIAAGGPVVAK.Y	2	676.0	769.5	507.6	593.1	264.9	253.1	400.5	413.2
		K.GASIYDVPEGGIPVYIAAGGPVVAK.Y	3	474.0	513.0	438.2	461.9	262.2	265.3	438.8	488.9
		K.ISYDTDPELALENTR.F	2	1864.6	1941.5	1205.4	1229.6	829.7	842.1	1068.6	1047.2
		K.NLVLGTSVLTPTFR.Y	2	1454.0	1337.9	1178.3	1021.0	572.2	574.4	990.3	973.6
		K.WGLNHLVFHAPGHDQR.R	4	113.4	83.1	44.3	35.9	19.3	36.8	8.6	40.0
		R.FWAPLSLTAEQK.H	2	799.4	737.9	669.7	563.5	403.1	396.0	661.1	624.6
		R.IFLGVGTGEALNEIATGYAGEWPEFK.E	3	530.6	620.9	294.4	369.7	178.4	257.1	175.3	241.9
		R.WIVASDPDEAVEK.V	2	133.5	116.6	39.5	56.9	56.3	71.9	23.8	36.3
MSMEG_0779	MSMEG 0774	R.STADIDAC*VADLDQLGSGK.V	2	23.3	15.5	44.9	43.2	4.0	4.7	47.6	47.3
		R.VVLTSSITGPITGYPGWSHYGATK.A	3	139.9	159.1	279.4	293.2	106.4	111.6	268.2	283.6
MSMEG_0783	MSMEG 0778	R.GALVEDIVNTVAITAIQAQGR.S	3	30.3	36.1	26.7	39.5	36.5	30.2	30.7	34.2
MSMEG_0789	MSMEG 0785	R.ADIALAAGADVLHLGQDDLPLDVAR.G	3	115.3	120.0	114.7	115.6	93.1	88.1	110.9	104.1
		R.GDLAEFADAALAGGVDLIQLR.D	3	13.6	12.8	9.8	8.2	4.4	5.2	3.6	3.8
MSMEG_0793	MSMEG 0788	R.GAAEAVLTAQLAR.E	2	34.2	36.5	19.6	23.5	35.4	33.3	42.0	39.9
		R.VDAETGTGVLDLLNR.L	2	75.7	81.9	65.9	64.7	64.6	67.2	117.6	127.3
MSMEG_0814	MSMEG 0807	K.LAGLGLAGVGVAHFAK.P	2	29.9	34.2	21.0	19.6	48.0	42.5	19.8	24.5
		K.LAGLGLAGVGVAHFAK.P	3	81.9	72.0	72.3	59.0	110.0	105.9	90.3	83.8

		K.PQLFESITKPAFPR.D	3	103.9	104.8	92.4	76.4	143.8	143.0	109.7	103.5
		R.IGAVGAVGYLTYLAGNAVR.N	2	106.9	156.2	183.8	253.0	145.3	121.2	225.5	210.0
		R.IGAVGAVGYLTYLAGNAVR.N	3	61.3	41.6	49.4	31.5	73.0	73.9	60.0	52.3
MSMEG_0825	MSMEG0817	K.GFHEVPVDIIAGQIQVVAEDIGIQAAK.T	3	127.8	147.6	208.2	226.2	87.9	118.8	111.6	150.4
		K.LLPLTPPGQTPTR.V	2	156.8	124.0	258.5	213.0	223.8	236.2	244.0	288.8
		R.LITGIEVVDEATQR.D	2	129.7	140.2	196.9	205.1	179.5	181.7	198.0	205.1
		R.NELFPIASLVTPNLDEVR.L	2	111.1	114.1	194.2	182.1	168.7	167.7	224.1	218.2
		R.VMTIAGTDSGGGAGIQADLR.T	2	31.6	38.7	59.2	66.7	56.4	56.2	74.2	85.5
MSMEG_0826	MSMEG0818	R.NSPVPVGTVPMYQALEK.V	2	111.6	116.1	15.3	16.4	45.8	53.2	5.2	17.5
MSMEG_0832	MSMEG0826	R.VTGLDADGSPITLEGEDLFAR.M	2	145.7	158.1	166.1	178.0	141.0	139.2	205.1	208.6
MSMEG_0834	MSMEG0828	R.VFNISDVAGAAENTANR.L	2	75.9	81.9	64.4	85.4	84.2	98.6	21.2	48.2
MSMEG_0835	MSMEG0829	K.LVTTTDAFTAEDLLDGAK.T	2	693.8	651.2	1071.5	940.0	828.1	728.9	1178.9	997.7
		R.YQQVNGAPGPDQTTMATGDAGSR.V	3	5.1	5.9	10.2	10.3	0.9	2.0	0.4	2.5
MSMEG_0859	MSMEG0851	R.EIAAPSDVIFELIADPAQQPR.W	2	13.3	15.7	14.1	17.8	11.9	10.8	16.1	15.8
		R.EIAAPSDVIFELIADPAQQPR.W	3	75.4	68.2	91.0	82.5	54.0	65.8	84.3	99.7
		R.IAWRPAVAGEQPLGHLWR.W	4	40.6	39.8	33.0	33.5	38.6	41.7	44.8	44.1
		R.PAVAGEQPLGHLWR.W	3	21.6	25.4	30.0	30.0	9.8	11.6	15.2	16.1
		R.TLVTHTYDWTQLTDEKR.F	3	31.9	40.0	40.9	50.6	26.5	29.0	46.1	39.6
MSMEG_0858	MSMEG0852	R.DSAPSLVFLDEIDALAPR.R	2	44.8	49.6	54.9	57.4	46.6	48.8	53.4	59.3
		R.ENAAVLVSPVTVYGAR.S	2	1353.5	1460.3	1128.5	1138.7	1204.6	1104.5	1060.0	1021.9

MSMEG_0861	MSMEG 0854	K.LSIGDTYGLIR.Y	2	40.3	77.7	1.9	3.6	20.3	24.1	2.2	1.9
		R.AGLISAGANAAFFR.H	2	10.8	10.3	2.3	2.3	6.9	12.0	1.8	1.5
		R.FHSAELEAASEDNER.N	3	1.1	0.9	0.0	0.0	0.9	0.6	0.1	0.1
MSMEG_0863	MSMEG 0856	K.LANLLFAYELQR.R	2	19.8	20.8	210.2	206.0	24.9	23.9	266.1	214.0
		R.VAIVTGSNTGLGYETAR.A	2	94.6	99.3	53.7	62.1	101.2	120.9	42.1	48.8
MSMEG_0877	MSMEG 0870	K.AGADAVVATPPFYVAPHADEVIR.H	3	84.8	89.3	51.8	55.2	74.4	86.3	42.2	53.9
		R.IGLTAGALGAFK.A	2	10.5	16.0	12.5	10.1	14.1	16.7	6.8	8.3
MSMEG_0876	MSMEG 0871	K.LANLLFTYELQR.R	2	33.7	36.2	74.2	84.0	29.0	24.7	94.0	101.2
		R.VAIVTGANTGLGLETAK.A	2	37.7	47.2	11.3	15.3	21.4	25.4	12.2	14.9
MSMEG_0880	MSMEG 0873	K.AGAATEVELK.E	2	88.4	65.4	43.5	29.9	101.1	76.0	87.0	71.3
		K.DETTIVEGAGDAEAIQGR.V	2	3348.0	3869.0	2045.3	2291.2	2719.5	2868.0	1471.1	1737.2
		K.DETTIVEGAGDAEAIQGR.V	3	444.5	489.4	243.0	282.8	395.5	408.7	176.0	177.5
		K.EQIAATAGISAGDQSIGDLIAEAMDK.V	2	111.5	114.4	142.3	154.0	46.2	70.3	51.5	75.6
		K.EQIAATAGISAGDQSIGDLIAEAMDK.V	3	1558.7	1596.5	1036.8	1131.3	1184.0	1185.7	735.1	748.5
		K.GYISGYFVTDAER.Q	2	2103.4	2181.6	1134.7	1177.2	2130.4	2109.3	980.9	972.9
		K.KTDDVAGDGTTTATVLAQALVR.E	2	275.8	353.0	67.3	94.7	363.1	357.2	145.6	136.7
		K.KTDDVAGDGTTTATVLAQALVR.E	3	2725.0	2527.9	1432.5	1386.5	3724.7	3115.6	1812.3	1599.9
		K.KWGAPTITNDGVSIAK.E	2	294.4	267.4	231.4	216.4	309.3	317.9	228.2	256.4
		K.KWGAPTITNDGVSIAK.E	3	247.4	216.3	142.1	127.4	278.0	296.2	150.8	174.0
		K.LAGGVAVIK.A	2	11.4	8.2	0.6	0.3	17.7	15.7	1.2	0.6
		K.QIALNGGLEPGVVAEK.V	2	609.6	653.1	497.7	499.2	840.1	829.1	558.0	597.4
		K.TDDVAGDGTTTATVLAQALVR.E	2	401.2	392.6	221.9	227.2	319.6	312.1	214.9	200.2

		K.TDDVAGDGTTTATVLAQALVR.E	3	350.2	345.3	224.0	230.7	350.0	289.1	188.7	180.8
		K.VIQSGKPLLIIAEDVEGEALSTLVVNK.I	3	2378.4	2404.1	1902.4	2052.4	1664.2	2257.5	1357.8	1920.3
		K.VIQSGKPLLIIAEDVEGEALSTLVVNK.I	4	398.5	409.7	235.3	272.5	390.4	403.8	222.8	259.1
		K.VSNLPAGHGLNAATGEYEDLLAAGVADPVK.V	3	548.6	549.3	165.1	153.8	675.0	685.5	146.1	150.6
		K.VVVTKDETTIVEGAGDAEAIQGR.V	3	999.4	1017.1	1004.9	1029.9	1666.9	1753.8	1197.7	1368.9
		K.WGAPTITNDGVSIAK.E	2	2308.4	2314.8	1850.2	1760.6	2280.6	2289.8	1678.0	1586.1
		R.KVVVTKDETTIVEGAGDAEAIQGR.V	3	210.4	242.1	51.7	56.9	877.2	1030.9	245.6	296.9
		R.KVVVTKDETTIVEGAGDAEAIQGR.V	4	93.5	97.0	221.4	213.2	465.4	463.3	375.4	433.0
		R.QEAVLEDPYILLVSSK.V	2	920.3	930.1	904.5	856.0	1044.5	1023.9	995.6	1002.4
		R.SALQNAASIAALFLTTEAVVADKPEK.A	3	3807.3	3910.0	2321.0	2458.2	3376.0	3787.1	2124.3	2405.9
		R.SALQNAASIAALFLTTEAVVADKPEK.A	4	524.2	557.4	307.7	355.9	429.4	522.0	254.1	322.7
MSMEG_0882	MSMEG 0875	R.VVDGVIEVGSLGDR.T	2	415.7	464.3	426.3	467.0	449.9	477.5	377.2	392.1
MSMEG_0884	MSMEG 0877	K.ASGVDAVQAFYDAALELGAESLHAPR.L	3	21.2	23.7	24.6	31.1	26.1	31.5	27.9	34.6
MSMEG_0887	MSMEG 0880	R.VVDTNWNPPVTASAR.L	2	149.1	162.2	215.2	206.9	91.1	108.7	164.4	198.9
MSMEG_0889	MSMEG 0882	R.TPLTAEVLLR.G	2	6.9	3.6	221.1	208.1	9.3	7.9	307.2	248.2
MSMEG_0899	MSMEG 0893	K.GALTYEFLWR.M	2	10.9	11.7	2.6	4.2	11.2	10.5	3.0	3.1
		K.YPPILITTSTR.D	2	49.9	36.4	25.8	34.1	38.8	19.4	35.1	22.5
		R.HLVYEDFAAIAADLVR.R	3	21.6	22.5	12.7	11.7	18.8	15.7	16.2	12.0
		R.LLYGTAGGPLVQLK.S	2	105.9	107.7	165.0	174.7	103.5	81.7	169.4	176.1
MSMEG_0903	MSMEG 0897	K.FTGPNSLEVALNDGGTEK.V	2	1004.4	1030.0	1001.4	1023.5	1042.5	936.7	968.7	898.5
		K.NYGVDVTIVEFLPR.A	2	111.0	106.6	95.8	106.0	49.0	99.1	60.2	119.4
		K.VEFSNAIIATGSSTR.L	2	861.4	818.4	1042.0	1026.8	845.2	800.4	1123.5	1116.5

		K.WDLTVNELAR.N	2	130.3	109.1	120.8	109.1	169.3	143.8	139.7	134.3
		K.YGELLGGHLIGPDVSELLP ELTLAQK.W	3	2111.4	2252.5	2137.9	2163.2	1895.0	2177.9	2264.8	2319.8
		K.YWGGVC#LNVGC#IPSK.A	2	415.6	396.1	566.7	586.1	484.6	472.4	616.7	678.5
		K.YWGGVC*LNVGC*IPSK.A	2	412.7	396.1	555.4	584.2	483.3	471.5	608.7	676.0
		R.AAQLGLNTAIVEPK.Y	2	738.6	538.9	784.5	633.3	1282.5	1253.5	1357.5	1412.4
		R.ATFC#QPQVASFGLTEEQ AR.D	2	171.6	205.5	173.7	230.4	135.0	153.2	129.5	154.1
		R.ATFC*QPQVASFGLTEEQ AR.D	2	171.6	205.5	173.7	230.4	135.0	153.2	129.5	154.0
		R.LVPGTSLSENVVTYETQIL SR.E	2	802.1	851.9	807.4	848.3	734.7	837.4	845.1	935.6
		R.TNVGHIYAIGDVTGK.L	2	226.5	206.1	348.4	319.7	310.9	303.4	572.5	597.9
		R.TNVGHIYAIGDVTGK.L	3	233.5	197.1	189.6	167.5	306.2	255.6	225.7	191.8
MSMEG _0905	MSMEG 0899	R.ALGPIGWIAAR.A	2	9.0	8.2	9.7	6.7	5.6	6.6	17.9	14.1
		R.TLITATDEFVVTR.S	2	56.4	67.3	78.4	48.3	70.6	31.6	135.9	53.4
MSMEG _0909	MSMEG 0903	R.IADDFLAGLR.R	2	28.5	28.4	12.0	12.6	27.5	26.4	21.3	19.5
		R.TMIDMIEPIEFPELLR.L	2	44.3	51.8	45.5	44.3	40.4	44.3	37.3	44.5
MSMEG _0911	MSMEG 0904	K.AMIAAGVAGSHWEDQLA SEK.K	3	470.6	426.8	70.2	88.0	798.8	740.2	47.2	74.2
		K.VEGDTSVENWLAPIVADG EAGFGGALNVYELQK.A	3	52.7	82.8	6.2	7.1	77.7	173.9	0.5	5.6
		R.DYTPEDVVALQGTVVEEH TLAR.R	3	75.7	67.0	8.6	5.5	41.2	49.9	8.8	6.9
		R.IATTVDPNSSTTALAGSTE EGQFH.-	3	514.1	597.3	116.2	136.8	1718.5	1903.8	48.9	97.1
		R.IATTVDPNSSTTALAGSTE EGQFH	2	330.3	419.4	4.6	4.4	1147.8	1230.3	1.5	4.5
		R.IATTVDPNSSTTALAGSTE EGQFH	3	515.9	603.0	112.3	133.2	1768.0	1964.6	50.6	105.1
		R.LAADVADVPTVVIAR.T	2	1084.8	1049.1	204.1	255.4	4001.7	3969.7	325.6	270.1
		R.LAADVADVPTVVIAR.T	3	49.8	40.4	0.3	1.0	289.9	246.5	0.7	0.6
		R.NQM*SAYVELQER.E	2	13.6	10.4	35.5	24.5	47.9	54.8	0.9	0.8

		R.NQMSAYVELQER.E	2	118.6	150.4	61.0	95.0	277.2	269.6	39.9	50.8
		R.TDAEAATLITSDVDER.D	2	559.3	537.1	81.6	93.7	1759.6	1589.2	56.0	83.4
		R.TDAEAATLITSDVDER.D	3	3.5	14.2	0.0	0.5	85.4	53.9	0.0	0.1
		R.TDAEAATLITSDVDERDQPFITGER.T	3	14.7	16.3	6.9	10.2	472.2	438.4	14.9	10.4
MSMEG_0912	MSMEG 0905	K.AVVAGLSHPMGPLR.L	2	104.0	81.7	69.6	57.2	115.8	152.6	40.7	52.8
		R.AGVDVLVYDTTDAFVTAGR.N	2	123.2	110.1	209.0	182.7	287.3	217.3	226.9	161.2
		R.LSDLVGLDTLK.L	2	245.0	250.4	351.5	360.5	399.8	364.3	406.1	354.8
		R.MVEGGVATIEDVDK.A	2	219.1	203.7	340.9	290.0	256.4	299.2	318.5	349.9
		R.VGVVGAGQM#GSGIAEVSAR.A	2	35.0	30.6	30.1	28.5	48.3	51.2	30.2	34.0
		R.VGVVGAGQM*GSGIAEVSAR.A	2	35.0	30.6	30.1	28.5	47.9	51.0	30.2	34.0
		R.VGVVGAGQMGSGIAEVSAR.A	2	318.9	356.9	434.6	457.9	487.0	506.4	518.0	532.6
		R.VGVVGAGQMGSGIAEVSAR.A	3	50.5	39.8	47.4	38.7	85.1	70.5	66.5	51.6
		R.VITDPDAVLASNTSSIPIMK.I	2	195.7	204.1	277.1	250.4	304.2	319.1	329.9	312.2
MSMEG_0913	MSMEG 0906	K.FIGEEIFPGGQLPAVEDIEK.L	2	432.3	465.5	195.9	195.2	628.4	607.5	200.8	197.0
		K.KDEAIAMQGQEVYDR.Y	2	5.1	3.7	0.7	0.4	12.8	6.8	1.2	0.3
		K.KDEAIAMQGQEVYDR.Y	3	33.2	27.2	13.5	11.4	54.1	41.8	13.4	12.3
		K.LDLKPGM*TLLDVGC#GWGGALER.A	3	43.4	51.8	7.2	11.6	43.5	50.2	8.8	8.3
		K.LDLKPGMTLLDVGC#GWGGALER.A	3	161.5	166.4	70.8	73.1	242.5	241.5	78.6	74.0
		K.LDLKPGMTLLDVGC*GWGGALER.A	3	161.5	166.4	70.8	73.1	242.5	241.5	78.6	74.0
		K.YDVNVIGITLSK.A	2	346.6	364.2	285.5	309.8	451.0	441.0	308.6	366.4
		K.YLTGC#ADFFR.R	2	200.3	198.1	75.7	78.7	291.7	286.4	77.2	76.1
		R.GITNVGQFTLVK	2	267.4	278.3	230.6	226.9	423.8	389.5	218.7	221.7
		R.LQGWEEFNEPVDR.I	2	694.2	758.5	339.5	372.5	955.6	965.3	340.6	366.5
		R.M*LLHTILAHTQK.F	3	100.5	77.9	57.7	51.9	111.9	111.1	47.0	53.1
		R.MLLHTILAHTQK.F	2	87.6	81.5	19.6	20.3	102.8	116.0	14.5	24.8

		R.MLLHTILAHTQK.F	3	143.0	132.5	57.7	57.7	214.4	211.1	47.0	53.1
		R.TLDMWAANLEAK.K	2	242.2	279.6	103.9	119.2	366.4	361.7	103.6	112.7
		R.YPVFFER.A	2	41.3	21.2	2.7	0.6	54.4	56.5	1.0	0.9
MSMEG _0916	MSMEG 0909	R.VYVETVAAEPEPYR.F	2	39.5	37.9	53.7	49.2	36.8	45.8	17.9	31.7
MSMEG _0918	MSMEG 0911	K.AGVSNPYLSQIER.G	2	14.3	20.0	286.7	314.6	49.8	43.3	305.3	287.1
		R.AGILEPSEPSEVR.D	2	0.8	0.1	21.0	19.0	1.9	3.8	18.6	24.5
		R.DAIVNDVSITER.Q	2	20.6	20.7	62.0	46.9	15.8	18.1	60.2	56.9
MSMEG _0919	MSMEG 0912	K.AAESYADQATATYNK.L	2	1.1	0.6	41.0	30.7	6.9	5.2	35.1	26.3
		K.LQEELPSQFGELR.E	2	545.1	593.2	5463.7	6006.0	1306.9	1293.3	5794.8	5882.0
		K.LVGVELPK.R	2	0.0	0.2	57.4	53.7	4.8	7.5	75.4	82.5
		R.KAAESYADQATATYNK.L	3	1.4	1.5	5.2	4.1	3.7	3.6	7.3	6.2
		R.VETYTDQAVELTQEALGT VASQTR.A	2	24.9	28.3	351.8	402.3	85.8	100.9	323.1	375.7
		R.VETYTDQAVELTQEALGT VASQTR.A	3	66.2	93.7	742.5	919.0	225.8	280.1	634.4	814.9
MSMEG _0924	MSMEG 0916	R.FGVPLGPVAQPLDGPWA R.A	2	66.6	73.0	15.2	16.4	59.2	70.1	12.6	17.2
MSMEG _0927	MSMEG 0919	K.IENFIGSQLVDLLVAEQR.F	2	11.6	15.5	17.9	22.5	9.2	10.9	13.7	15.7
		K.IENFIGSQLVDLLVAEQR.F	3	10.9	10.3	15.2	14.0	7.6	7.9	11.0	11.3
		R.LPGLVSQFHPGDLSIR.R	3	11.5	9.8	19.1	18.5	4.5	6.3	11.9	12.2
MSMEG _0930	MSMEG 0922	K.EIDGTAVVADVTDDESVN ALAAQLDR.V	3	93.2	101.0	46.4	55.3	58.9	8.9	31.8	19.4
		K.GITPLVAEDIAEVIGFVASR .P	2	31.9	33.5	22.2	24.0	13.2	19.0	13.9	17.2
		K.GITPLVAEDIAEVIGFVASR .P	3	17.8	17.2	13.3	11.9	7.3	9.8	6.3	10.3
		K.TLASLGFHVIC*AAR.R	2	45.8	41.7	54.9	61.4	42.0	43.6	89.6	54.6
		R.LTEIAPGAVETEFSLVR.F	2	236.4	237.4	195.2	196.3	200.0	191.4	238.4	251.2
		R.PSHVNLDQIVIRPR.D	4	99.5	94.5	54.8	50.2	102.4	85.6	73.6	63.6

MSMEG _0935	MSMEG 0927	K.EKYGEEQFMAWR.R	2	34.1	26.2	10.5	8.0	27.7	33.1	6.7	12.3
		K.IGGEYLDPEAAAAGAAAV AAQGAK.K	2	436.1	435.9	1869.7	1988.1	352.7	393.6	5471.7	6051.5
		K.IGGEYLDPEAAAAGAAAV AAQGAK.K	3	383.0	419.6	2478.1	2737.6	319.7	316.9	6703.0	7654.0
		K.NLFTGWVDVDLTDK.G	2	84.8	73.6	75.5	69.6	51.6	44.5	51.7	43.4
		K.YGEEQFMAWR.R	2	113.8	142.0	103.2	130.7	75.6	86.7	95.6	107.6
		K.YLDGMSDEEVVGLNIPTGI PLR.Y	2	101.9	103.0	94.9	89.5	85.1	76.2	92.2	88.5
		K.YLDGMSDEEVVGLNIPTGI PLR.Y	3	156.2	152.8	170.6	165.7	119.7	115.5	171.8	165.0
		R.AITTAHLALDAADR.L	2	67.2	54.6	57.7	49.0	68.3	41.8	83.6	56.1
		R.AITTAHLALDAADR.L	3	140.5	119.0	89.2	78.5	138.8	117.9	114.8	90.2
		R.FVPYYESAILPDLK.A	2	788.0	810.9	897.7	913.5	790.8	787.6	1046.0	1089.4
		R.FVPYYESAILPDLK.A	3	20.5	16.9	6.6	14.6	18.3	18.2	12.9	15.7
		R.GGQLMAEQGVLPDVLYT SLLR.R	2	184.2	183.2	190.8	196.1	178.9	161.9	224.3	219.0
		R.GGQLMAEQGVLPDVLYT SLLR.R	3	169.0	163.9	172.0	158.2	133.6	135.6	173.0	167.0
		R.YADIDGGPLTEC#LK.D	2	414.2	417.0	491.2	471.0	325.5	299.5	429.4	429.1
		R.YADIDGGPLTEC*LK.D	2	412.8	415.4	491.2	470.3	319.6	298.4	426.9	428.3
MSMEG _0937	MSMEG 0929	R.SKIEEDPANPVHLVTVR.G	3	17.1	21.2	4.9	7.5	26.2	32.9	6.5	7.2
MSMEG _0943	MSMEG 0934	R.AVATDLVAQTMAGSAAML LDR.L	2	373.1	429.0	23.8	28.5	303.2	331.7	24.4	32.1
		R.AVATDLVAQTMAGSAAML LDR.L	3	103.5	101.0	49.6	45.2	107.4	95.7	73.8	66.2
		R.LDQVNAAGGAALDTTPAQ LR.A	2	75.3	82.4	68.5	63.4	95.3	95.1	84.9	95.1
		R.VMPNAPVIVGGGVSALAP GR.F	2	102.0	102.4	191.9	187.9	127.4	124.5	189.2	187.8
MSMEG _0944	MSMEG 0935	K.AVHDLLETSYFDAG	2	37.8	39.6	180.7	188.8	41.1	40.7	179.4	176.9
		R.AQFLTVAEVASLMR.V	2	23.5	23.1	56.8	50.7	20.5	18.0	45.9	41.5
MSMEG _0948	MSMEG 0939	K.EC#GHDNLIAPLTFLAVAG R.R	3	27.1	28.6	19.9	22.3	20.4	23.1	12.7	15.5

		K.ECGHDNLIAPLTFLAVAGR.R	3	27.2	0.0	19.9	0.0	20.4	23.0	12.6	15.5
		K.FDVPINLER.V	2	12.1	13.4	3.6	2.8	6.6	7.4	3.5	2.2
		K.LIFHRPIVVGDK.L	3	67.5	52.1	29.0	19.9	56.6	59.8	24.7	25.1
		R.EFSAAVKDDHPAHFDEAAAK.E	3	4.6	1.8	1.4	0.3	9.4	7.1	4.5	3.1
		R.EFSAAVKDDHPAHFDEAAAK.E	4	17.4	9.8	6.8	3.2	25.9	27.6	11.4	10.9
		R.VQLDLFDKFDVPINLER.V	3	52.2	45.5	32.0	28.4	37.5	38.7	24.4	22.5
		R.YPDYFEVGR.E	2	79.0	64.7	27.5	22.6	74.5	75.6	25.1	25.0
MSMEG_0954	MSMEG 0944	R.ALYGWTVLVPR.T	2	33.9	37.8	19.2	18.2	34.0	35.7	20.5	13.5
		R.GWEIEDVTAYR.T	2	29.1	38.2	1.5	3.0	17.4	21.0	0.4	1.0
		R.ITFVGSGPGDPGLLTTR.A	2	196.1	191.7	57.3	56.3	253.3	227.4	29.9	37.6
		R.LIAGDPLSVDAVITEINALAK.T	2	49.6	57.8	5.9	5.8	55.7	80.3	4.8	10.4
		R.LIAGDPLSVDAVITEINALAK.T	3	34.2	53.7	2.9	5.1	44.8	58.8	6.1	6.6
		R.LVTHGALPIEVPTIAVEPPR.S	3	104.5	103.1	211.4	228.6	129.2	112.0	204.9	196.1
MSMEG_0965	MSMEG 0955	R.LIASTGDSVTTYGEPWNM*N	2	26.5	32.7	24.3	34.3	23.1	36.1	8.2	18.8
		R.LIASTGDSVTTYGEPWNMN	2	39.4	0.0	42.7	0.0	27.1	43.4	10.8	27.4
		R.TLTVQQWDTFLNGVFPLDR.N	2	303.9	359.2	319.9	376.9	214.5	270.7	322.0	375.6
		R.TLTVQQWDTFLNGVFPLDR.N	3	90.8	105.0	110.0	122.7	33.6	48.0	92.4	129.3
MSMEG_0969	MSMEG 0959	K.VAEHGLSFGAPTPSETELAAEIIDR.V	3	351.6	338.5	192.5	246.0	116.0	104.0	74.4	84.9
		R.FITSAGGYWLTDADGNR.Y	2	148.2	147.6	186.4	245.6	46.4	52.3	74.3	71.8
		R.SGWYGLDPVEADLLTFGK.V	2	255.2	265.9	67.7	81.7	108.4	101.7	80.7	74.7

MSMEG _0970	MSMEG 0960	K.AAGHEAVC#VSHQLPVET LR.R	3	213.5	204.5	136.4	126.7	184.7	187.9	107.0	105.2
MSMEG _0987	MSMEG 0977	R.ATGTDAEESAVAVLPAAE TAVAR.E	3	38.7	0.0	114.4	0.0	39.8	49.8	121.1	110.3
MSMEG _1010	MSMEG 0998	K.IVGVIEQDTGARPISDDLP ALVR.T	3	233.9	243.5	426.5	460.3	232.3	268.4	366.7	396.3
		R.LWLSALWGGLPE	2	12.6	13.9	14.5	15.2	8.6	11.7	10.1	15.1
		R.PISDDLPALVR.T	2	23.1	25.9	3.3	4.8	3.8	9.3	1.7	1.5
MSMEG _1011	MSMEG 0999	R.IGVSVVVPGAVK.T	2	36.8	35.8	42.3	40.1	50.7	51.0	43.5	44.3
MSMEG _1019	MSMEG 1007	K.AMDSPDFAQTIEVAIR.A	2	112.2	103.9	338.3	297.1	139.6	106.8	354.7	280.3
		K.DISC#NLGSLNIAK.A	2	170.7	177.1	289.0	282.2	168.7	163.6	305.5	308.3
		K.DISC*NLGSLNIAK.A	2	153.1	163.4	280.9	273.5	152.0	155.6	297.5	308.3
		K.KNEDMYLFSPYDVER.V	2	46.6	54.5	71.5	78.0	21.9	41.2	45.0	72.7
		K.KNEDMYLFSPYDVER.V	3	61.7	55.4	100.4	88.3	36.9	40.5	76.7	86.3
		K.LDYLIENEYYER.E	2	190.5	202.3	412.4	406.4	126.2	148.9	363.2	324.9
		K.LLEDSFSYANQLGAR.Q	2	339.0	327.2	656.7	585.6	374.3	345.8	756.4	647.3
		K.NIENQSSGVIPIMK.L	2	311.1	287.3	757.5	714.6	288.4	281.0	676.7	629.1
		K.RGGGVALLLSNIR.E	3	17.7	11.1	20.7	16.1	31.9	28.6	45.0	39.7
		K.TLSLGVVIPDITFELAK.K	2	103.6	110.9	237.9	262.2	133.0	125.5	304.1	297.6
		K.YASGEFFDKYTDQVWEP K.T	3	93.2	50.3	56.4	89.8	125.2	64.8	109.8	135.3
		R.FPTFLGAFK.Y	2	48.9	46.0	113.5	108.2	47.1	39.2	109.0	92.1
		R.GEPVSC#FLLR.I	2	55.2	56.2	116.7	106.6	64.4	48.4	106.5	95.3
		R.GGGVALLLSNIR.E	2	94.3	85.5	198.6	174.0	107.7	88.9	211.4	169.4
		R.LFADADIHIPTQDDWK.Q	3	111.0	104.3	126.6	135.2	110.6	84.1	101.3	114.1
		R.VYGVPFADVSVTEK.Y	2	342.7	368.1	791.9	795.7	392.5	350.4	900.5	849.2
MSMEG _1021	MSMEG 1010	R.ISLITLGVDNLAASR.R	2	39.6	39.0	39.9	42.9	56.5	59.5	45.8	48.0
MSMEG _1028	MSMEG 1015	K.IDSEYNGYSAIAIR.S	2	391.4	408.3	182.1	212.1	401.8	414.4	190.4	252.5
		K.NWQSINATQFLGEFLR.S	2	418.9	429.1	301.2	320.1	277.5	294.5	262.0	285.3
		K.VYGDAVIAADGAYSPIK.R	2	333.4	326.3	433.0	528.9	247.2	258.1	113.1	140.2

		K.VYGDAVIAADGAYSPIKR.A	3	135.7	136.4	169.5	198.0	166.6	148.1	239.6	221.0
		R.SEMHANRPDSDSLDIYLK.L	3	322.0	283.8	195.8	174.1	154.8	172.0	84.4	106.4
		R.VTVFSPSEWELSFPR.R	2	1019.2	952.0	692.5	654.5	853.5	787.9	792.4	692.9
		R.YDLVIAGGGPSGSAAAWQAAQTGAK.V	3	342.4	332.3	281.7	274.4	181.8	285.5	128.7	323.9
MSMEG_1033	MSMEG1019	K.ALMNLGYEALFPR.D	2	70.3	67.3	217.8	197.2	24.8	30.3	95.6	96.8
		K.VPVSNDIQSWHTLTDNEK.Q	3	529.8	539.7	993.1	968.0	388.9	413.0	729.3	731.5
MSMEG_1037	MSMEG1024	K.AMGADVTVLSQSLK.K	2	1359.9	1357.8	189.3	207.1	216.2	201.1	225.0	213.4
		K.LDGALVELGLPEHPMEVPAFPLLAQR.R	3	2375.9	2643.9	399.2	438.2	327.0	376.2	280.6	319.9
		K.VAVIGLGGLGHVAVK.L	2	416.3	375.2	60.0	72.2	21.5	16.8	36.1	60.3
		K.VAVIGLGGLGHVAVK.L	3	1012.1	867.8	24.5	25.2	121.9	111.9	14.1	14.8
		R.FVIDTASLR.S	2	76.2	43.7	16.5	12.5	11.9	14.1	17.8	20.9
		R.IPDSLPLDAAAPLLC#AGITTYSPLR.H	3	1301.0	1445.6	149.4	171.0	174.7	201.9	111.5	155.4
		R.IPDSLPLDAAAPLLC*AGITTYSPLR.H	3	1301.0	1445.6	149.4	171.0	174.7	201.9	111.5	155.4
		R.LGASAYYATSDPETFDK.L	2	2916.5	3147.8	530.0	629.0	499.8	547.9	349.0	417.7
MSMEG_1038	MSMEG1025	R.PAHVAWLNDEVAAGR.I	2	121.0	128.3	87.2	88.8	75.8	60.7	87.5	70.7
		R.PAHVAWLNDEVAAGR.I	3	247.3	282.6	160.5	177.7	146.4	148.2	151.6	143.3
MSMEG_1041	MSMEG1028	R.VATLAGLPASVLDEVAEQLELTPGAR.T	3	118.8	153.6	81.8	96.1	40.6	63.8	41.5	73.7
MSMEG_1046	MSMEG1033	K.AFGQAIALEVIDGR.Y	2	125.0	135.6	259.3	275.9	118.4	125.2	311.1	340.3
		K.VVASGLLTDVLTSENLSK.A	2	211.3	212.6	405.3	382.3	194.3	192.1	378.8	378.5
		K.VVASGLLTDVLTSENLSK.A	3	31.8	33.2	54.5	53.4	28.7	31.1	58.2	55.0
		R.DLVVSAGYAVLGR.W	2	169.1	181.8	136.5	128.4	223.8	203.1	138.5	137.5

		R.IAAATEHPSSGTAYVLGE R.L	3	70.1	53.7	104.9	64.8	60.4	71.1	97.9	99.7
MSMEG _1047	MSMEG 1034	R.AALANHSLSFADFLR.D	2	39.4	47.5	65.8	80.3	72.6	66.5	70.1	73.5
		R.AALANHSLSFADFLR.D	3	64.3	65.5	30.7	33.8	80.1	81.9	28.4	33.4
		R.ADLEVFLMR.R	2	20.8	18.4	68.6	47.0	25.7	22.6	41.4	37.8
		R.ADLLRPWANWVTPK.E	2	182.3	203.7	139.1	136.1	199.2	228.2	140.3	169.5
		R.ADLLRPWANWVTPK.E	3	43.4	41.0	13.8	17.6	58.9	65.2	19.2	24.4
		R.LGVDEGLAEALVC#AAAR.E	2	105.1	107.6	103.6	107.6	102.8	103.3	109.4	102.5
		R.LGVDEGLAEALVC#AAAR.E	3	10.0	12.9	1.5	2.0	14.6	14.4	0.3	3.0
MSMEG _1048	MSMEG 1035	R.FSDHPVDVLAAAK.A	3	12.6	9.2	3.2	4.2	4.7	5.7	4.9	6.6
MSMEG _1049	MSMEG 1036	K.LPFLSGWR.R	2	40.7	35.0	1.3	0.7	38.6	35.8	0.5	0.0
		R.AAALEAVVDLHTFDPADL ER.M	3	92.5	75.5	26.8	26.0	82.7	70.3	18.4	32.9
MSMEG _1051	MSMEG 1038	K.RAPITFDTLFK.P	2	8.8	9.4	30.2	26.0	6.0	3.2	26.3	36.9
		R.PLDVIFPIVAR.E	2	75.5	78.3	63.5	65.2	54.2	53.3	46.5	43.9
MSMEG _1052	MSMEG 1039	R.GIYSNEAGQGTGPHAAAA AEVTHPAK.Q	3	0.2	0.3	12.2	10.5	3.9	3.4	13.0	9.6
		R.GIYSNEAGQGTGPHAAAA AEVTHPAK.Q	4	2.2	2.1	104.6	82.9	24.4	25.4	82.5	75.0
MSMEG _1060	MSMEG 1045	K.DVDTVEFSYR.G	2	54.2	40.7	86.4	63.1	68.6	74.5	50.0	59.5
		K.IVTIDYIDDLDGVPIDEK.D	2	363.6	459.5	652.9	817.6	340.7	464.8	325.2	438.0
		K.IVTIDYIDDLDGVPIDEKDV DTVEFSYR.G	3	94.1	96.0	99.5	114.1	96.7	109.5	98.4	105.7
		R.GEDYTLVLTTK.N	2	211.3	193.1	352.0	322.1	334.6	359.0	349.4	337.3
MSMEG _1061	MSMEG 1046	R.SDTWKPETWNPTAVPSV AQLAK.A	3	180.0	321.2	197.4	345.2	124.6	137.5	130.9	154.2
		R.VIDDTTIAIPERPGNK.R	2	32.2	26.6	27.4	25.2	26.2	25.6	25.4	27.4
		R.VIDDTTIAIPERPGNK.R	3	15.2	0.0	8.3	0.0	11.7	13.3	6.3	8.5
MSMEG _1075	MSMEG 1059	R.TGYQYASGETAETVDPAR .A	2	11.6	8.7	4.9	3.0	5.8	8.0	0.8	2.1

		R.VPGFDDLTDITYHR.H	3	53.6	49.9	7.6	7.7	44.2	38.9	3.9	1.8
MSMEG _1076	MSMEG 1060	K.GTDAAQLEITGTR.V	2	13.5	12.3	10.1	8.9	7.3	5.4	9.4	6.5
MSMEG _1077	MSMEG 1061	R.YVVGGLPGADWTANLR.A	2	50.1	54.5	72.2	77.8	34.4	38.8	62.7	65.6
MSMEG _1078	MSMEG 1062	R.VIDPDSWTAYPDTATVLK.S	2	35.6	35.1	34.7	30.3	13.9	14.9	16.8	25.4
MSMEG _1090	MSMEG 1078	R.LEEVEPLLTAFVTTTPELALEQAK.A	3	14.6	21.5	82.5	115.1	17.5	25.2	97.0	134.0
MSMEG _1115	MSMEG 1105	R.NLTGGIVALHAATKPA	2	4.7	3.5	19.4	19.1	0.9	6.0	54.8	32.0
MSMEG _1133	MSMEG 1122	R.FGATFSGANDEQIER.L	2	32.0	37.9	58.3	75.1	63.4	72.9	79.6	65.1
MSMEG _1134	MSMEG 1123	R.LYISDTANPNAFATGR.N	2	31.6	34.0	47.9	51.3	39.8	40.8	29.1	23.4
MSMEG _1143	MSMEG 1133	R.QLLACGLAVLVVAGLISTVLLVK.A	2	0.6	0.8	11.3	31.7	0.0	0.1	10.5	27.8
MSMEG _1165	MSMEG 1158	K.GADLDVALQFVNYR	2	96.7	88.9	192.5	177.8	88.9	93.2	215.2	218.9
		K.GDEVIELTSSTEER.V	2	42.3	36.9	65.5	60.1	27.8	29.8	60.5	62.6
		K.KRDDLQAVIALLK.G	3	20.3	21.3	24.6	27.7	13.5	16.0	22.7	27.1
		K.VDRQEVDNALNQAAK.E	2	11.9	3.9	18.3	6.3	11.2	14.1	19.0	19.4
		K.VDRQEVDNALNQAAK.E	3	21.4	10.4	31.6	14.0	20.2	27.0	34.0	40.6
		R.DDLQAVIALLK.G	2	17.5	18.8	21.9	23.4	8.3	8.3	12.0	11.8
MSMEG _1188	MSMEG 1181	R.LDLDSVAAELR.S	2	66.1	57.5	61.4	53.5	47.0	48.8	56.8	51.8
MSMEG _1203	MSMEG 1198	K.YDVDVIGLTLSK.N	2	79.8	73.6	134.0	129.0	104.0	90.9	136.5	151.6
MSMEG _1215	MSMEG 1208	R.FVEQIFSAEQQPK.E	2	21.3	20.8	15.9	19.4	19.2	18.3	27.8	20.3
MSMEG _1225	MSMEG 1218	R.QYASTALHPRWR.A	2	50.3	29.0	176.7	153.3	17.6	13.6	143.7	150.9
MSMEG _1244	MSMEG 1236	K.TIGLLNLVSSGGR.I	2	2.8	2.1	10.4	8.1	3.1	3.8	16.3	14.7
		R.LAAEHAPSEDSTAVAQMPGR.K	3	6.1	5.9	4.3	4.5	4.7	6.4	3.4	3.6

		R.LGLSSLLSDVADTVYLR.T	2	4.7	5.2	3.6	4.8	5.0	5.9	1.8	3.0
		R.TAGPTFLNVATALFR.E	2	5.2	4.9	16.7	11.6	11.1	8.7	7.0	4.7
		R.TLFSFVTGAEPAALPR.Y	2	14.8	12.2	11.7	10.6	16.0	17.3	5.1	9.6
MSMEG _1247	MSMEG 1240	K.VVQVGGVEILDELPK.K	2	10.5	11.8	227.1	210.3	61.4	47.6	279.6	235.2
		R.LAQDLGDQPWTVVLEILD SK.K	2	18.3	21.5	70.1	80.7	33.6	56.4	67.6	111.2
		R.SDVSDEELFTAVLQTFGR. R	2	28.9	33.3	125.1	128.7	64.6	73.3	147.3	155.2
MSMEG _1252	MSMEG 1245	R.TFDAAADTGQVGGLR.R	2	4.8	3.9	49.0	48.1	2.3	3.5	19.6	23.1
MSMEG _1253	MSMEG 1247	R.AAGTGFTNVDMPVQR.F	2	34.0	35.4	37.4	39.5	36.9	34.7	13.9	19.2
		R.FLAGDYSQPVEVIDFER.L	2	0.6	0.9	9.6	9.6	7.3	9.3	11.0	11.3
		R.LTEFAEPNALWK.L	2	2.9	3.6	17.1	16.0	5.2	5.1	31.9	29.7
		R.LVLDPYGQGLLLR.L	2	0.3	0.6	19.0	18.9	6.9	7.2	20.5	23.4
		R.VSTESLEPGSTEPAPQTD VAIGKPVR.S	3	0.7	1.1	23.4	29.5	10.3	12.3	23.6	28.8
MSMEG _1254	MSMEG 1248	K.GVWEFLVR.R	2	0.4	0.3	0.6	1.3	2.2	1.9	1.8	2.5
		K.TESFLLPILDSLVR.E	2	1.0	0.9	12.0	11.7	7.4	6.4	18.8	14.6
		R.VLQALQC*EVDAEQLLK.G	2	59.8	56.2	14.0	15.7	33.7	19.7	18.2	19.4
MSMEG _1255	MSMEG 1249	K.SWLVEDGVEPESIAVLTR. S	2	39.9	49.4	45.4	50.2	28.8	37.3	37.4	57.2
		R.IADKPGDPGVYVGGIDAL GR.D	3	161.2	150.2	256.4	217.5	112.5	109.9	171.9	172.7
		R.IVLTTFNATLAQGLK.T	2	22.7	25.3	60.2	56.2	57.7	37.1	101.4	76.9
		R.SVVPPSPKPVLSYLAGVG YTLADLTER.L	3	4.1	6.2	17.6	20.6	15.7	27.3	35.9	57.0
		R.VFGHVFEFGSK.R	3	116.8	68.6	125.6	66.5	95.8	56.3	117.5	78.7
		R.VIEGGDFAAWR.T	2	10.9	13.3	11.9	22.5	16.4	21.5	23.9	29.9
MSMEG _1265	MSMEG 1257	K.IAC#DAVLVPTDDHFTIEA PWR.G	3	190.3	194.8	148.3	145.5	182.1	193.6	189.0	183.7

		K.IAC*DAVLVPTDDHFTIEAPWR.G	3	190.3	194.1	147.7	145.5	181.1	193.6	189.0	182.5
		K.TAGSPLDSVGHLVEEFLK.D	2	73.3	67.7	116.9	106.9	83.2	65.4	140.1	120.3
		K.TAGSPLDSVGHLVEEFLK.D	3	138.0	133.5	163.1	149.1	132.9	117.6	179.6	169.6
		R.GVALTLYADPLDR.Q	2	154.9	180.2	243.4	307.6	103.4	131.2	153.4	252.3
MSMEG_1285	MSMEG 1277	R.ALAEQWLLVDR.S	2	14.0	14.4	223.0	200.8	34.4	28.9	306.8	190.3
		R.VLPTGSIMQFFGGIER.V	2	13.1	18.4	29.7	45.9	27.4	25.2	31.6	46.4
MSMEG_1292	MSMEG 1284	R.LPVAALVTGAGTNLLAAADVLR.S	3	12.0	11.0	20.8	20.1	5.0	5.1	23.3	15.9
MSMEG_1295	MSMEG 1287	R.IPDLYTGPASGIHR.L	3	1.5	2.0	31.5	32.0	2.8	4.6	29.8	28.5
MSMEG_1296	MSMEG 1288	R.GDFSGAHLTGDQSQVLPTDSQK.Q	3	171.9	170.8	408.8	431.1	105.9	109.8	257.7	308.8
		R.VMATSLIAQWR.F	2	3.0	3.7	37.6	43.1	2.1	2.5	18.9	16.8
MSMEG_1331	MSMEG 1324	R.AQQVATEAAALHTNAHVASK.L	4	8.6	6.9	7.4	5.9	12.0	9.3	10.6	8.2
MSMEG_1334	MSMEG 1327	K.LPGDPTVYPGHWYSAEPSAPLEEVKR.S	4	140.9	173.6	66.2	96.0	117.9	135.8	85.9	112.1
		R.LVAGDTLFLEGC#GR.T	2	98.0	109.9	76.3	93.7	77.8	82.2	63.4	76.4
		R.SVPVHVNSHEADWVSQVTGIAR.S	3	134.2	153.1	53.6	63.0	108.8	123.1	45.5	51.6
MSMEG_1340	MSMEG 1333	K.NIITNDAGEIVQEAYTTLAGR.A	2	120.0	198.8	129.1	197.0	138.4	138.9	138.9	123.9
		K.NIITNDAGEIVQEAYTTLAGR.A	3	186.7	131.8	191.0	146.4	158.8	190.4	159.9	167.0
		R.LYC#DVYVHAVR.R	3	17.9	13.1	6.6	4.7	17.8	16.8	4.1	3.8
		R.YPDFYEVGR.E	2	74.2	66.6	44.6	43.1	61.7	74.1	37.3	43.7
MSMEG_1341	MSMEG 1334	K.LVTIAISATAGGK.K	2	111.6	118.4	114.2	116.6	114.7	112.9	103.0	92.6
		R.FTAVVPVPNDGVGAEITFNGR.V	2	102.9	100.7	98.2	94.4	112.6	117.7	98.8	99.2
		R.GDLVNYAGVSGDLNPIHWDDEIAK.Q	3	590.6	761.7	618.0	812.5	627.6	705.3	612.7	629.8

MSMEG _1342	MSMEG 1335	K.YPDPFLIGR.E	2	86.8	84.0	49.4	45.7	90.6	93.0	43.8	42.5
		R.FGADIVVTR.N	2	30.8	24.8	11.1	9.6	27.7	24.0	8.8	7.6
MSMEG _1344	MSMEG 1337	R.RGVAESTDEPETAAEAAAAVEAGGK.A	3	39.0	50.8	15.3	20.9	62.6	65.7	10.6	13.9
MSMEG _1345	MSMEG 1338	R.ETPVELTFNQVAK.-	2	781.6	723.7	624.9	585.8	687.8	666.5	648.5	652.6
		R.ETPVELTFNQVAK.I	2	751.0	778.9	555.7	599.9	706.9	683.7	631.2	651.6
		R.LKPGDWYVIHSYAGYENK.V	4	46.0	54.2	26.9	16.7	34.5	44.9	20.1	15.2
MSMEG _1346	MSMEG 1339	K.ADLNANDIDAAAK.I	2	0.5	0.0	2.7	0.0	0.2	0.4	2.2	1.9
		R.GNVIPVEITVYEDR.S	2	897.8	854.8	885.0	857.4	754.9	817.9	829.2	842.0
MSMEG _1347	MSMEG 1340	K.AEQAQAAGADIVGSDDLIEK.I	2	846.4	899.8	462.9	490.3	1032.6	1085.9	549.0	591.6
		K.AEQAQAAGADIVGSDDLIEK.I	3	261.3	274.2	145.2	138.3	331.8	334.3	200.4	190.6
		K.IQGGFLDFDAAIATPDQM*AK.V	2	60.2	56.6	18.0	21.5	68.9	63.6	6.1	12.1
		K.IQGGFLDFDAAIATPDQMAK.V	2	410.7	402.0	214.7	204.9	567.6	503.4	263.7	256.7
		K.KQDATVEVAIR.L	2	23.0	17.5	11.9	8.7	45.3	32.3	16.0	12.6
		K.KQDATVEVAIR.L	3	45.6	32.1	29.0	24.1	80.5	51.4	46.0	34.6
		K.KVTVSTTTGPGIPVDPSVTR.N	2	93.1	90.6	51.2	50.1	134.7	139.0	98.5	90.2
		K.KVTVSTTTGPGIPVDPSVTR.N	3	592.3	656.5	417.2	443.1	698.8	622.6	523.6	484.2
		K.LAENYGAALDEVLR.A	2	1426.3	1518.9	776.2	776.7	1714.9	1622.2	923.7	888.8
		K.LAENYGAALDEVLR.A	3	35.0	38.5	11.4	6.3	43.1	40.0	5.7	8.8
		K.VTVSTTTGPGIPVDPSVTR.N	2	1632.5	1597.6	875.9	902.1	1871.9	1958.2	919.8	1008.9
		K.VTVSTTTGPGIPVDPSVTR.N	3	227.5	215.1	90.0	90.4	255.2	265.3	83.5	93.2
MSMEG _1350	MSMEG 1343	K.LGLHMTFEDAR.F	3	28.6	25.3	8.5	6.5	60.4	55.4	6.7	9.5
		K.LTQLQEIGPHYVR.T	2	28.5	17.6	112.9	106.5	69.8	64.9	88.4	88.0
		K.LTQLQEIGPHYVR.T	3	119.7	108.6	65.8	60.9	258.1	266.8	70.7	73.5

		K.YDVNVIGLTLSGEQR.E	2	125.5	132.4	87.2	91.6	176.9	161.4	79.2	81.6
		R.FIMTEIFPGGR.L	2	54.6	61.3	25.1	25.4	143.3	142.6	30.7	32.1
		R.IVSIGAFEHFGFER.Y	2	263.2	278.0	33.5	48.6	314.7	297.4	30.6	41.6
MSMEG _1352	MSMEG 1345	R.SPAVYTLEDM#ADDAAAL LDHLEIDR.A	3	5.1	8.2	6.2	9.1	2.9	6.3	5.1	6.5
MSMEG _1364	MSMEG 1357	K.AAGLFNAPASQVAR.L	2	216.3	173.1	141.9	111.4	224.5	293.8	143.4	172.4
		K.ASTATVVTEYR.G	2	5.3	5.2	3.8	3.1	5.7	5.1	2.0	2.5
		K.ATAVADIAEQFK.A	2	477.9	561.6	350.2	399.6	547.7	546.9	394.5	392.3
		K.RAASEAGIEGLDELFAGP TAIAFVK.G	3	13.7	13.9	7.9	9.9	38.8	41.3	21.9	23.2
		R.AASEAGIEGLDELFAGPT AIAFVK.G	2	300.2	342.8	215.9	242.4	299.8	316.6	197.7	187.3
		R.AASEAGIEGLDELFAGPT AIAFVK.G	3	446.1	457.7	380.2	367.8	389.5	490.1	321.3	361.7
		R.ALGDSATYTVAK.N	2	5.6	5.4	3.0	2.5	5.4	5.6	1.7	2.0
		R.GLTVANLAELR.R	2	565.7	593.9	440.8	418.9	613.6	624.7	482.0	416.9
MSMEG _1365	MSMEG 1358	K.AKLEAAGASVTVK.-	2	1.4	1.0	0.4	0.2	2.2	1.5	2.0	0.8
		K.AKLEAAGASVTVK	2	31.2	23.3	41.5	29.3	46.4	36.6	55.8	40.1
		K.AKLEAAGASVTVK	3	26.6	9.8	19.3	7.3	42.1	33.1	29.0	21.9
		K.DLVDSAPKPLLEK.V	2	905.1	1133.2	654.9	837.4	988.8	1011.8	687.3	644.7
		K.EM*TLLELSEFVK.Q	2	280.3	232.0	207.8	168.9	233.1	210.5	175.0	141.3
		K.EMTLLELSEFVK.Q	2	674.8	648.1	549.6	526.1	565.4	575.7	436.9	427.9
		K.LEAAGASVTVK	2	47.5	38.2	51.7	41.9	47.9	34.5	50.6	34.4
		K.LSTEELLDAFK.E	2	395.0	373.9	267.9	247.9	500.1	450.0	341.3	291.9
MSMEG _1367	MSMEG 1360	K.AGWNIDVAAGVPDWASK. L	2	281.6	249.4	166.1	146.7	256.7	277.0	172.9	168.6
		K.ALGWTNEQIVER.F	2	951.1	975.9	579.9	529.3	983.1	652.7	518.2	440.6
		K.DMTYAAPLFVTAEFINNNT GEIK.S	3	44.5	56.0	22.0	25.9	19.8	34.1	9.5	18.4
		K.DTTSGTDEALLDIYR.K	2	548.9	569.2	368.9	353.6	429.9	462.2	337.2	308.3
		K.DTTSGTDEALLDIYRK.L	2	15.3	14.1	23.9	23.8	33.7	26.9	40.1	40.9
		K.EFFGTSQLSQFM*DQNNP LSGLTHK.R	3	264.0	248.7	147.9	150.2	206.4	200.1	119.2	109.9
		K.EFFGTSQLSQFMDQNNPL SGLTHK.R	3	476.4	546.8	287.6	339.4	371.6	420.3	242.3	270.4

K.ESAQTLLENLFFK.E	2	310.9	254.8	174.3	136.1	360.5	230.8	220.8	132.8
K.ESAQTLLENLFFK.E	3	4.1	6.3	1.4	2.0	3.2	4.9	0.4	2.1
K.GENIPEPGIPESFK.V	2	579.0	690.6	380.9	423.2	551.6	665.6	406.7	433.7
K.GGEVEFVSADQVDYMDVSPR.Q	2	63.0	52.9	40.5	34.0	46.8	42.6	24.5	22.8
K.ILPVEDM#PFLPDGTPVDIILNTHGVPR.R	3	185.3	189.5	120.6	116.8	130.6	148.9	88.7	100.1
K.ILPVEDM*PFLPDGTPVDIILNTHGVPR.R	3	185.3	187.3	120.6	116.8	129.0	146.6	88.7	100.1
K.ILPVEDMPFLPDGTPVDIILNTHGVPR.R	3	436.5	498.0	295.2	308.9	217.4	374.2	169.1	257.1
K.LGLNAGKPITSSTLTEEDVVATIEYLVR.L	3	284.5	365.7	156.1	207.4	197.0	294.5	119.5	177.9
K.LGLNAGKPITSSTLTEEDVVATIEYLVR.L	4	12.2	15.8	6.7	9.6	10.7	12.5	6.4	8.3
K.LREPLEVPGLLDVQTDSFEWLVGSDR.W	3	69.2	67.2	35.8	36.0	52.5	67.5	31.6	37.2
K.PITSSTLTEEDVVATIEYLVR.L	3	19.8	21.3	8.9	11.0	16.1	15.5	7.9	8.0
K.SNAITNNSVPGAPNR.V	2	1.5	1.5	0.7	0.8	1.2	1.5	0.5	0.9
K.SQTVFM*GDFPMMTEK.G	2	38.0	30.9	12.2	13.7	18.4	20.2	3.4	5.2
K.SQTVFMGDFPM*M*TEK.G	2	37.0	34.4	8.3	12.4	19.9	20.4	3.6	5.0
K.SQTVFMGDFPM*MTEK.G	2	39.6	34.4	12.8	15.6	19.9	20.4	3.6	5.2
K.SQTVFMGDFPMM*TEK.G	2	40.2	35.6	12.8	15.9	20.9	21.1	3.7	5.0
K.SQTVFMGDFPMMTEK.G	2	122.4	110.6	82.5	70.3	82.2	82.2	60.1	56.8
K.TGVIEEVSADYITVMADDGTR.Q	2	102.4	104.1	67.8	66.2	84.2	101.6	53.3	56.3
R.AAANLGINLSR.N	2	72.3	54.6	30.2	27.2	60.2	50.2	27.1	26.1
R.AAIDAGDVVVADK.T	2	24.2	26.1	11.9	14.0	23.2	22.9	11.0	12.0
R.DIPNVSDEVLADLDER.G	2	575.2	539.7	352.0	314.6	551.7	573.9	350.3	336.7
R.DIPNVSDEVLADLDER.G	3	46.1	47.0	18.3	22.5	25.3	45.1	8.7	22.3
R.EDDDELPAGVNELVR.V	2	375.2	363.0	233.4	214.2	239.5	258.3	152.4	160.5

R.FGFSEIM*M*GTLEK.D	2	34.7	30.9	14.0	13.0	28.0	24.5	11.8	9.6
R.FGFSEIM*MGTLEK.D	2	45.8	42.3	19.8	18.0	32.0	30.2	11.8	12.7
R.FGFSEIMM#GTLEK.D	2	34.7	30.9	14.0	13.0	28.0	24.5	14.3	9.6
R.FGFSEIMM*GTLEK.D	2	34.7	30.9	14.0	13.0	28.0	24.5	14.3	9.6
R.FGFSEIMMGTLEK.D	2	149.5	134.2	100.5	85.6	141.2	109.7	107.3	73.7
R.GAWLEFDVDKR.D	2	65.1	61.6	29.1	30.5	66.1	74.7	33.8	42.9
R.KKGGEVEFVSADQVDYM DVSPR.Q	3	31.0	30.4	5.2	6.0	32.8	31.2	7.0	8.5
R.LSALGPGGLSR.E	2	3.2	4.4	16.7	15.6	2.7	3.5	13.5	19.2
R.LVEEDVLTSIHIEEHEIDAR .D	3	289.1	223.4	149.1	114.5	375.8	294.1	240.3	165.0
R.LVEEDVLTSIHIEEHEIDAR .D	4	198.0	162.8	100.3	84.4	215.7	193.1	118.2	98.4
R.M#NIGQILETHLGWVAK.A	3	54.9	61.0	37.2	39.0	34.4	41.3	23.3	25.8
R.M#TTQDVEAITPQTLINIR PVVAAIK.E	3	168.8	163.8	122.9	117.7	118.9	103.1	86.9	75.7
R.M*NIGQILETHLGWVAK.A	3	54.9	61.0	32.6	32.8	34.4	40.6	23.3	25.8
R.M*TTQDVEAITPQTLINIR. P	2	47.1	42.4	20.9	21.7	21.2	19.0	12.7	19.2
R.M*TTQDVEAITPQTLINIRP VVAAIK.E	3	130.9	122.4	82.3	74.2	118.6	103.1	86.9	75.7
R.MNIGQILETHLGWVAK.A	2	23.4	20.0	18.5	16.6	42.1	26.1	33.8	18.6
R.MNIGQILETHLGWVAK.A	3	63.6	61.3	32.6	32.8	86.0	73.4	52.6	38.9
R.MTTQDVEAITPQTLINIR.P	2	47.6	46.6	20.9	21.7	52.4	49.7	32.3	41.1
R.MTTQDVEAITPQTLINIR.P	3	54.6	47.6	9.0	14.9	24.9	35.2	5.1	15.1
R.MTTQDVEAITPQTLINIRP VVAAIK.E	3	130.9	122.4	82.3	74.2	154.6	143.2	105.5	96.9
R.RMNIGQILETHLGWVAK.A	3	2.7	2.1	0.2	0.0	7.8	8.1	1.6	1.5
R.SEAPLVGTGMELR.A	2	344.3	334.7	204.8	189.7	290.5	273.0	185.7	154.4
R.SGEPFPYPVTVGYMYILK. L	2	154.6	148.8	97.0	85.8	142.4	155.0	109.1	103.8
R.SPGVYFDETIDK.S	2	436.0	408.0	343.0	307.6	340.3	387.0	277.4	241.9

		R.SPGVYFDETIDKSTEK.T	3	47.4	54.9	1.2	2.1	49.2	53.1	9.1	15.2
		R.STGPYSM#ITQQPLGGK.A	2	105.1	96.7	57.5	53.8	64.3	62.2	35.9	38.1
		R.STGPYSM*ITQQPLGGK.A	2	102.9	95.2	54.0	49.2	62.2	60.9	33.8	36.2
		R.STGPYSMITQQPLGGK.A	2	329.7	306.9	218.9	201.4	287.2	247.4	202.1	166.0
		R.TVGELIQNQIR.V	2	423.0	427.0	318.1	312.7	376.9	347.2	315.3	266.9
		R.VEAGQVIADGPC#TQNGEMALGK.N	2	66.3	64.5	39.0	40.6	62.4	64.8	40.3	35.4
		R.VEAGQVIADGPC#TQNGEMALGK.N	3	141.6	178.7	103.3	132.5	91.4	134.6	64.9	96.4
		R.VEAGQVIADGPC*TQNGEMALGK.N	3	141.6	178.7	103.3	132.5	91.4	134.6	64.9	96.4
		R.VFSREDDDELPAGVNELVR.V	2	19.2	20.3	4.2	4.9	37.2	35.1	12.1	14.1
		R.VFSREDDDELPAGVNELVR.V	3	290.0	308.2	167.3	192.8	336.0	361.9	212.8	242.5
		R.VNPFGFIETPYR.K	2	426.6	355.1	295.4	250.9	373.9	355.6	294.4	257.3
MSMEG_1368	MSMEG1361	-.M#LDVNFFDELR.I	2	102.5	96.0	52.8	47.6	64.3	56.1	37.4	30.5
		-.M*LDVNFFDELR.I	2	44.6	48.7	23.5	24.0	50.1	47.7	35.2	28.8
		-.MLDVNFFDELR.I	2	44.6	48.7	23.5	24.0	50.1	47.7	35.2	28.8
		K.ALEEFYPADNPIITIVK.S	2	847.0	779.7	684.6	658.8	911.0	697.8	1032.3	816.9
		K.ALEEFYPADNPIITIVK.S	3	49.0	60.3	26.7	32.0	31.3	49.0	18.9	39.8
		K.ASLATDSWLSAASFQETTR.V	2	285.4	246.2	138.9	123.6	248.1	227.9	157.5	138.2
		K.ASLATDSWLSAASFQETTR.V	3	267.3	233.9	14.5	22.3	56.3	83.2	3.3	23.3
		K.DAPEQGVYSSPAEAIMAMDR.G	2	118.2	107.3	54.3	52.4	75.8	77.5	36.6	45.6
		K.DAPEQGVYSSPAEAIMAMDR.G	3	81.2	79.5	40.1	39.8	29.9	49.6	13.9	31.9
		K.HIEVIVR.Q	2	5.8	4.2	1.5	1.3	4.4	3.7	1.1	1.3
		K.ITIVPDDGGEEVVYDK.L	2	894.2	964.7	632.4	671.0	727.5	786.1	603.5	647.6
		K.LEADLAELEAEGAK.S	2	665.5	716.3	465.5	528.3	541.2	532.6	479.2	444.9
		K.LEADLAELEAEGAK.S	3	8.1	11.8	0.9	2.6	3.6	8.5	0.3	2.0

K.LIENFDIDAEAESLR.E	2	458.7	446.0	264.2	231.5	441.9	407.9	287.4	254.1
K.LIENFDIDAEAESLR.E	3	24.6	25.5	6.7	9.0	14.5	23.1	3.2	9.0
K.LM*ALELFKPFVM*K.R	3	11.9	10.5	0.8	1.7	8.2	9.7	0.7	2.6
K.LM*ALELFKPFVMK.R	3	20.8	25.6	3.7	5.8	19.3	18.6	5.1	5.4
K.LMALELFK.P	2	22.1	19.2	4.9	5.7	13.7	11.7	4.5	3.4
K.LMALELFKPFVM*K.R	2	5.3	6.9	7.9	10.0	7.9	8.4	9.0	10.9
K.LMALELFKPFVMK.R	2	16.1	15.6	12.0	12.2	28.4	15.1	22.5	13.7
K.LVDIGEAVGIVAAQSIGEPGTQLTM*R.T	3	34.4	56.6	81.0	65.7	38.6	42.1	84.0	78.4
K.LVDIGEAVGIVAAQSIGEPGTQLTMR.T	3	175.1	182.1	111.7	116.1	131.7	136.2	111.9	106.4
K.LVDIGEAVGIVAAQSIGEPGTQLTMR.T	4	26.7	28.2	9.8	11.9	19.6	22.3	8.4	9.6
K.QLIVDEVLYR.E	2	59.6	48.9	34.5	27.8	77.4	70.8	57.1	47.8
R.AQKLEADLAELEAEGAK.S	2	10.8	8.2	9.6	6.5	27.6	19.7	26.5	18.0
R.AQKLEADLAELEAEGAK.S	3	8.2	9.8	17.4	16.3	24.6	28.0	29.3	30.0
R.DAHVETSAFAR.T	2	1.4	0.9	0.3	0.1	0.9	0.7	0.1	0.1
R.DAHVETSAFAR.T	3	1.4	0.7	0.1	0.0	0.6	0.6	0.0	0.0
R.EGLTVLEYFINTHGAR.K	2	72.3	73.7	42.9	44.0	102.8	74.8	80.2	59.9
R.EGLTVLEYFINTHGAR.K	3	172.3	191.4	79.4	87.5	202.7	177.2	119.9	96.6
R.ELDRLDEIWNTFTK.L	2	88.9	81.1	40.9	41.4	99.6	80.8	56.8	44.5
R.ELDRLDEIWNTFTK.L	3	56.4	67.8	27.0	30.2	55.2	57.3	33.2	32.8
R.FATSDLNDLYR.R	2	494.0	484.1	270.9	275.3	354.6	350.9	232.6	242.7
R.FPM*IVVAQTVDK.L	2	114.7	122.7	51.4	67.2	77.0	82.1	39.5	46.3
R.FPMIVVAQTVDK.L	2	411.3	411.0	276.8	238.2	361.2	294.4	301.8	214.8
R.GHDLGDPAIDALLAAGITTVK.V	2	37.0	36.9	33.4	31.3	39.5	35.0	50.0	45.5
R.GHDLGDPAIDALLAAGITTVK.V	4	5.9	6.3	33.2	12.7	11.1	9.4	7.2	19.2
R.HNELSTLEAEM*AVEK.K	2	16.5	13.8	4.7	3.8	12.2	9.7	2.8	1.1
R.HNELSTLEAEM*AVEKK.A	3	1.7	2.0	0.9	1.3	2.9	3.5	0.7	2.0
R.HNELSTLEAEMAVEK.K	2	105.2	100.5	48.8	47.5	70.6	78.2	41.5	43.0
R.HNELSTLEAEMAVEK.K	3	104.2	95.7	44.3	40.5	61.7	68.3	29.3	34.0
R.IGLATADDIR.N	2	18.8	20.6	7.9	11.0	15.3	16.3	7.4	9.0

R.ILM*LSSNNILSPASGK.P	2	48.9	58.3	14.5	14.8	43.4	43.7	5.4	6.2
R.ILM*LSSNNILSPASGKPLAM*PR.L	3	42.0	43.7	25.4	32.8	23.4	30.0	23.6	28.6
R.ILMLSSNNILSPASGK.P	2	172.4	167.2	91.7	89.7	84.1	85.8	52.7	55.5
R.ILMLSSNNILSPASGKPLAM*PR.L	3	141.7	121.5	174.9	160.6	88.2	82.6	147.7	154.3
R.ILMLSSNNILSPASGKPLAMPR.L	3	291.7	281.5	174.9	160.6	204.1	220.0	147.7	154.3
R.LDEIWNTFTK.L	2	61.4	55.3	31.1	26.9	32.5	30.7	14.6	14.6
R.LDM*VTGLYYLTTLVEGATGEYQAATK.D	3	35.8	45.8	19.4	24.3	10.9	23.4	7.0	14.3
R.LGIQAFEPQLVEGK.A	2	472.7	544.4	288.7	310.7	413.3	477.3	347.4	373.8
R.LGYLLDLAPK.D	2	418.9	389.3	203.0	179.1	397.4	364.7	229.1	203.8
R.LIDLGAPEIIVNNEK.R	2	1202.8	1213.7	1032.4	1041.9	1119.2	1182.7	1044.5	1020.9
R.LIDLGAPEIIVNNEKR.M	2	13.0	9.8	0.8	1.8	28.4	18.8	6.7	6.7
R.LVDVSQDVIVR.E	2	421.0	416.6	280.0	272.3	352.3	323.1	281.2	246.2
R.M#GHIELAAPVTHIWYFK.G	3	74.9	59.6	27.0	23.1	59.6	44.7	24.6	16.0
R.M#LQESVDALFDNGR.R	2	63.2	60.6	26.4	26.6	19.5	21.0	9.1	9.2
R.M*GHIELAAPVTHIWYFK.G	3	47.3	37.3	23.1	17.2	58.0	43.2	24.6	16.0
R.M*GHIELAAPVTHIWYFK.G	4	59.5	50.5	22.3	18.9	36.9	38.3	17.9	21.5
R.M*LQESVDALFDNGR.R	2	63.2	60.6	26.4	26.6	19.5	21.0	9.1	9.2
R.MGHIELAAPVTHIWYFK.G	3	47.3	37.3	23.1	17.2	59.4	46.3	44.0	30.5
R.MGHIELAAPVTHIWYFK.G	4	59.5	50.5	22.3	18.9	59.7	65.5	30.1	32.4
R.MLQESVDALFDNGR.R	2	72.2	68.6	32.2	31.4	50.7	51.4	31.4	31.2
R.QRPQVWDVLEEVIAEHPVLLNR.A	3	9.3	9.4	3.3	3.9	11.1	10.3	6.8	5.6
R.QRPQVWDVLEEVIAEHPVLLNR.A	4	9.2	10.2	3.1	4.2	8.9	11.4	5.4	6.2
R.RLVDVSQDVIVR.E	2	26.9	25.7	55.1	43.6	56.9	43.5	109.4	80.3
R.RLVDVSQDVIVR.E	3	33.4	33.4	61.1	45.1	46.7	48.6	84.7	79.4
R.RVTIIDSGSTEFLPGSLTER.A	2	3.1	4.3	1.4	1.1	7.9	7.8	1.0	0.8

R.RVTIIDSGSTEFLPGSLTER.A	3	102.3	105.9	57.4	64.3	148.6	168.4	93.6	112.6
R.RVVAEGGEPAAGRPVLM*GITK.A	3	13.6	12.6	3.1	3.2	13.6	10.9	3.5	3.5
R.RVVAEGGEPAAGRPVLMGITK.A	3	49.6	46.3	16.1	16.9	61.7	54.9	21.8	26.1
R.SVIVVGPQLK.L	2	24.4	24.7	5.8	9.2	21.0	19.7	4.1	6.1
R.SVLTC#TSATGVC#AMC#YGR.S	2	37.6	34.2	16.7	20.3	36.1	28.3	22.7	19.8
R.SVLTC*TSATGVC*AMC*YGR.S	2	37.6	34.2	16.7	20.3	36.1	28.3	22.7	19.8
R.TFHQGGVTGGADIVGGLPR.V	2	65.3	59.5	36.2	35.3	82.2	52.3	53.0	37.8
R.TFHQGGVTGGADIVGGLPR.V	3	441.3	392.8	258.4	244.2	370.1	326.8	265.7	246.6
R.TLATDAVDANGNVIIER.G	2	352.2	354.7	220.9	218.1	331.3	308.4	294.0	238.5
R.TLATDAVDANGNVIIER.G	3	46.1	45.2	43.5	51.4	32.6	45.4	42.3	56.9
R.VM*FNELLPK.S	2	57.4	52.6	25.4	28.2	40.4	39.4	22.8	23.0
R.VMFNELLPK.S	2	217.5	197.9	130.0	124.6	173.1	158.1	131.1	119.7
R.VRLEESDKFFK.I	2	15.6	13.1	1.8	0.9	29.9	18.1	14.7	7.8
R.VRLEESDKFFK.I	3	48.6	43.8	18.4	17.3	61.7	65.6	33.3	36.1
R.VTIIDSGSTEFLPGSLTER.A	2	623.3	563.7	352.3	316.1	465.8	442.7	344.8	301.5
R.VVAEGGEPAAGRPVLM*GITK.A	2	3.6	3.0	8.0	6.8	5.3	2.8	11.9	6.4
R.VVAEGGEPAAGRPVLMGITK.A	2	39.0	32.8	15.0	9.3	54.1	29.0	25.5	12.1
R.YGEYFTGAM#GAESIK.K	2	65.1	64.4	25.1	29.6	43.5	37.0	16.1	18.6
R.YGEYFTGAM#GAESIKK.L	3	44.1	42.6	20.7	20.9	30.8	33.7	18.8	20.1
R.YGEYFTGAM*GAESIK.K	2	65.1	64.4	24.7	29.2	43.5	37.0	15.8	18.3
R.YGEYFTGAM*GAESIKK.L	2	5.8	5.7	1.4	2.5	9.1	6.6	5.9	3.0

		R.YGEYFTGAM*GAESIKK.L	3	44.1	42.6	20.7	20.9	30.8	33.7	18.8	20.1
		R.YGEYFTGAMGAESIK.K	2	204.0	191.7	168.6	157.9	125.1	119.3	135.9	127.7
		R.YGEYFTGAMGAESIKK.L	2	56.1	53.5	50.6	47.2	58.9	47.2	61.4	47.3
		R.YGEYFTGAMGAESIKK.L	3	87.0	80.5	42.1	37.3	79.4	78.0	45.8	43.6
MSMEG _1383	MSMEG 1376	K.ALDALDTDVPVYLENTAG GDHAMAR.H	3	118.5	116.0	78.3	86.8	101.8	102.7	95.5	94.9
		R.HANFGAGQIDPQLLAAVV K.A	3	43.8	37.3	13.2	10.2	35.9	35.5	12.8	10.3
MSMEG _1384	MSMEG 1378	K.AGTSTVWTVTETLR.K	2	27.8	39.8	83.8	93.5	23.3	23.8	78.0	79.7
MSMEG _1393	MSMEG 1387	K.IDKDLTVVGLNNTITYR.D	3	12.2	18.0	179.7	203.0	14.5	18.5	194.3	169.7
MSMEG _1397	MSMEG 1392	R.VIDDIISMLNR.V	2	3.5	6.1	85.9	85.6	2.1	3.2	63.6	56.4
MSMEG _1398	MSMEG 1393	K.LTSQVEVTAYIPGEGHNL QEHSMVLVR.G	4	102.0	92.8	46.8	43.5	104.9	121.6	40.8	42.9
MSMEG _1399	MSMEG 1394	K.RPLVNDPVYGSQLVTQLV NK.V	2	231.9	168.4	105.4	75.5	216.3	186.3	98.2	78.3
		K.RPLVNDPVYGSQLVTQLV NK.V	3	3090.8	3241.0	1548.5	1683.7	2795.7	3051.6	1502.9	1504.5
		R.ALDNVKPALEVR.S	2	22.9	20.8	11.9	14.1	22.8	23.8	11.4	12.3
		R.ALDNVKPALEVR.S	3	98.8	0.0	31.0	0.0	102.3	113.7	32.3	31.6
		R.IVYGALEQAR.E	2	30.1	31.3	10.7	12.3	29.6	34.2	10.1	12.7
		R.LANEILDASNGLGASVK.R	2	1020.9	1243.2	622.0	708.5	883.5	922.4	594.1	575.0
		R.LANEILDASNGLGASVK.R	3	134.5	128.2	50.8	52.5	117.2	117.4	44.3	48.1
		R.RVGGATYQVPVEVRPDR. S	3	272.1	256.0	103.9	101.7	294.2	289.9	123.3	113.6
		R.RVGGATYQVPVEVRPDR. S	4	342.0	309.9	137.7	127.1	353.8	371.3	157.0	152.7
		R.VGGATYQVPVEVRPDR.S	2	322.0	301.6	177.4	166.6	348.0	277.5	200.5	158.2

		R.VGGATYQVPVEVRPDR.S	3	1400.3	1973.9	797.9	1105.4	1258.9	1324.4	749.9	750.6
MSMEG _1400	MSMEG 1395	K.IGETHDGASTTDWM*EQE QER.G	3	34.4	37.9	35.2	37.2	30.4	35.5	21.9	25.9
		K.IGETHDGASTTDWMEQE QER.G	3	582.1	503.5	630.3	511.0	419.5	419.2	484.1	447.5
		R.ILYYTGVNYK.I	2	170.5	100.5	229.0	165.2	211.7	98.3	268.8	166.0
		R.NIGIM*AHIDAGK.T	2	4.5	2.8	60.1	38.3	3.8	5.1	21.8	54.1
		R.NIGIMAHIDAGK.T	2	41.6	34.2	60.1	38.3	55.4	43.5	72.1	69.4
MSMEG _6535	MSMEG 1396	K.AQVPLSEM*FGYVGDLR.S	2	179.8	190.2	97.1	97.4	150.0	153.1	84.5	80.6
		K.AQVPLSEMFGYVGDLR.S	2	634.6	715.9	721.3	789.1	683.5	634.7	781.6	683.3
		K.AQVPLSEMFGYVGDLR.S	3	40.8	57.9	35.6	49.0	51.9	42.1	41.4	45.2
		K.LGADFYFTVR.T	2	63.3	52.2	44.1	34.9	143.1	86.0	115.7	70.7
		K.LLETVAESDEALLEK.Y	2	1226.0	1186.9	1439.0	1335.9	1445.4	1229.7	1725.7	1473.8
		K.LLETVAESDEALLEK.Y	3	100.4	87.7	96.9	94.8	102.9	94.9	109.8	98.8
		K.LTVNSELYPVLC#GSAFK. N	2	161.0	151.2	191.9	185.3	142.8	140.1	183.8	185.8
		K.LTVNSELYPVLC*GSAFK. N	2	160.8	151.2	191.5	184.3	142.8	139.6	183.8	185.4
		K.VLIDLEPFVGEDGATYEFE NK.V	2	422.1	432.7	508.0	509.7	440.6	423.1	521.3	561.7
		K.VLIDLEPFVGEDGATYEFE NK.V	3	193.6	256.5	205.2	271.4	201.4	210.8	199.8	261.3
		K.VTLLDGAYHEVDSSEM*A FK.V	3	69.4	86.7	57.2	67.1	64.4	70.5	53.7	52.7
		K.VTLLDGAYHEVDSSEMAF K.V	2	65.9	60.8	59.8	63.5	81.0	60.5	77.9	67.1
		K.VTLLDGAYHEVDSSEMAF K.V	3	372.2	360.1	426.6	387.7	379.8	337.0	466.8	390.2
		K.YEVEDIPADLADK.A	2	305.0	282.2	413.2	329.6	148.7	253.5	199.9	351.1
		K.YEVEDIPADLADKAEEYR. T	2	194.0	202.2	118.2	132.5	185.2	155.3	119.7	141.2
		K.YEVEDIPADLADKAEEYR. T	3	605.3	603.8	663.1	642.2	640.5	566.6	694.0	624.6

		K.YFGGEELSVDEIK.G	2	778.3	832.1	857.3	959.0	764.4	736.9	945.2	901.5
		R.ANYSMVFDSYAEVPANVSK.E	2	319.5	289.3	284.7	259.6	282.2	254.0	324.5	259.5
		R.ASAGHIYAVIGLK.D	2	100.4	83.4	182.8	178.1	131.1	107.4	219.5	196.0
		R.ASAGHIYAVIGLK.D	3	145.5	112.5	100.6	64.0	177.5	150.6	144.1	101.6
		R.EFKVEANVGKPQVAYR.E	3	9.2	11.9	55.7	43.8	27.3	38.8	38.7	63.9
		R.GETALGEKYEVEDIPADLADKAEEYR.T	3	39.5	58.1	30.4	34.3	121.6	142.8	102.2	131.1
		R.GETALGEKYEVEDIPADLADKAEEYR.T	4	116.9	117.1	121.2	154.8	296.6	294.3	335.8	353.0
		R.KLTVNSELYPVLC#GSAFK.N	2	63.2	71.9	338.0	351.4	73.9	105.1	139.7	209.5
		R.KLTVNSELYPVLC*GSAFK.N	2	63.2	71.3	337.2	347.4	73.7	104.9	139.7	209.5
		R.KLTVNSELYPVLC*GSAFK.N	3	126.5	124.3	982.5	999.1	168.8	155.1	194.7	308.8
		R.KPSVDEPFSALAFK.I	2	660.7	562.4	695.1	635.2	608.2	399.9	787.6	727.2
		R.KPSVDEPFSALAFK.I	3	363.8	316.6	429.3	369.0	454.0	389.6	555.7	464.1
		R.VYSGVVESGSQVVNSTK.G	2	651.3	422.9	772.5	481.5	615.1	393.0	719.3	437.5
		R.VYSGVVESGSQVVNSTK.G	3	273.7	218.1	331.9	241.9	243.6	162.8	285.4	180.0
MSMEG_1401	MSMEG 1397	K.LIQPVAMDEGLR.F	2	2067.4	1676.0	2361.2	1862.2	2088.5	2519.8	2282.5	2738.7
		K.LLDQGQAGDNVGLLLR.G	2	4169.4	3997.8	4656.2	4486.7	5452.4	5134.1	5314.6	4906.1
		K.LLDQGQAGDNVGLLLR.G	3	193.4	187.0	199.2	184.5	276.0	275.2	226.2	248.2
		K.SDAVDDEELIELVEM#EVR.E	2	113.5	115.9	86.1	96.0	88.9	106.1	55.2	66.2
		K.SDAVDDEELIELVEM*EVR.E	3	103.4	110.8	99.0	104.9	88.9	96.5	51.1	62.7
		K.SDAVDDEELIELVEMEVR.E	2	466.6	471.0	496.7	505.3	487.2	571.5	435.8	561.0
		K.SDAVDDEELIELVEMEVR.E	3	223.4	227.8	234.8	243.8	229.9	293.2	221.0	266.6

		K.SVEELMEAVDASIPDPVR.E	2	1472.8	1353.1	1632.8	1566.7	1339.2	1201.7	1322.4	1220.1
		K.SVEELMEAVDASIPDPVR.E	3	15.0	22.1	26.0	38.3	19.2	16.2	10.5	11.3
		K.TTVTGVEMFR.K	2	75.2	56.4	44.7	46.9	43.5	38.7	50.8	27.8
		K.VLHDKFPDLNESR.A	3	59.2	41.9	38.8	26.3	94.9	102.1	96.9	114.0
		R.AFDQIDNAPEER.Q	2	43.0	29.4	22.9	21.8	65.2	73.3	50.7	57.2
		R.ELLAAQDFDEEAPVVR.V	2	6036.0	6152.8	6773.3	6827.6	7979.5	7852.6	7977.9	7790.8
		R.ELLAAQDFDEEAPVVR.V	3	236.9	236.9	210.1	197.0	336.6	329.6	258.4	245.1
		R.ETDKPFLMPVEDVFTITGR.G	2	637.1	726.7	752.6	855.7	359.5	361.7	362.3	378.2
		R.ETDKPFLMPVEDVFTITGR.G	3	2640.2	2867.0	3025.5	3175.8	2160.7	1905.8	2240.1	1941.0
		R.GITINISHVEYQTDK.R	2	358.3	340.4	415.0	482.5	297.1	301.6	396.5	484.0
		R.GITINISHVEYQTDK.R	3	185.5	174.4	169.7	169.5	173.2	161.2	129.0	141.9
		R.GITINISHVEYQTDKR.H	3	1082.6	817.0	1051.0	830.2	1805.6	1863.0	1396.6	1705.8
		R.GQVVVKPGTTTPHTEFEGQVYILSK.D	3	2454.0	2587.0	2536.3	2658.8	2756.5	2835.5	2647.5	2656.0
		R.GQVVVKPGTTTPHTEFEGQVYILSK.D	4	2971.1	2958.7	3101.8	3305.2	3809.0	3770.0	3517.8	3438.0
		R.GVINVNEEVEIVGIRPETTK.T	2	413.9	464.3	391.8	467.5	465.0	463.3	318.3	362.2
		R.GVINVNEEVEIVGIRPETTK.T	3	3341.3	3171.8	3458.4	3337.9	3925.6	4148.5	3673.7	3783.2
		R.HYAHVDAPGHADYIK.N	3	41.7	19.8	32.8	14.0	62.2	40.5	44.4	28.3
		R.HYAHVDAPGHADYIK.N	4	102.2	50.9	74.2	36.8	135.2	102.8	85.1	62.8
		R.KLLDQGQAGDNVGLLLR.G	2	41.0	48.8	48.8	64.0	138.2	154.9	128.8	130.0
		R.TKPHVNIGTIGHVDHGK.T	3	28.6	11.7	18.2	6.8	64.3	34.3	48.5	22.7
		R.TKPHVNIGTIGHVDHGK.T	4	194.7	99.3	150.8	73.4	290.6	211.8	194.7	138.1
MSMEG_1416	MSMEG 1415	R.IGSFPDLQGIR.V	2	112.8	90.3	153.8	113.5	119.3	113.9	99.8	144.9

		R.LADGSELPYDELVIATGLVPK.R	2	170.4	172.1	201.2	160.9	178.8	174.8	262.9	220.3
MSMEG_1430	MSMEG1427	R.LLEAATVLLR.S	2	4.4	3.4	7.2	8.6	1.7	1.4	4.0	4.8
MSMEG_1435	MSMEG1433	K.AYDHEAIDASAR.K	2	0.1	0.0	0.0	0.0	0.3	0.1	0.0	0.0
		K.AYDHEAIDASAR.K	3	1.3	0.8	0.2	0.1	1.7	1.1	0.4	0.1
		R.LIDILDPTPK.T	2	357.1	376.4	226.1	237.5	458.6	443.7	274.2	247.4
		R.TGASVVGPVPLPTEK.N	2	990.9	1053.7	635.7	648.1	1190.0	1250.1	704.7	674.7
MSMEG_1436	MSMEG1434	K.LGMTQVFDENNK.V	2	135.3	101.7	89.6	60.0	161.0	175.2	59.0	70.4
		K.VHKVDAENGVLLIK.G	3	32.9	25.6	28.9	34.9	87.3	76.3	40.1	41.0
		K.VIKPVAGQFAAAGVNPR.R	2	92.2	69.2	30.3	20.2	97.4	91.0	14.5	22.9
		K.VIKPVAGQFAAAGVNPR.R	3	462.8	429.0	223.5	187.2	472.2	511.7	157.0	181.5
		R.DGYSAVQLAYGEISPR.K	2	730.0	795.4	427.4	427.3	904.5	988.3	470.6	477.5
		R.DGYSAVQLAYGEISPR.K	3	192.9	178.5	72.4	60.1	279.0	254.4	88.7	77.8
		R.KVIKPVAGQFAAAGVNPR.R	2	48.0	23.0	33.6	15.2	127.1	93.4	47.9	33.0
		R.KVIKPVAGQFAAAGVNPR.R	3	429.5	278.0	198.1	128.7	833.0	672.1	329.6	282.3
		R.KVIKPVAGQFAAAGVNPR.R	4	360.8	218.4	173.5	101.5	706.1	577.8	289.5	231.3
MSMEG_1437	MSMEG1435	K.TDGSVELPAELFDVEPNIALMHQVVTAQLAAK.R	4	141.2	141.1	62.8	65.0	74.7	109.5	29.3	49.9
		R.APQFTGGGTVHGPKPR.D	4	1.9	1.1	0.2	0.1	3.1	2.2	0.5	0.2
		R.IHAVTELVEGQTPSTK.S	2	412.5	286.9	267.4	179.9	457.7	435.7	274.6	269.4
		R.IHAVTELVEGQTPSTK.S	3	832.1	641.9	445.7	323.0	842.2	853.3	431.8	428.9
MSMEG_1438	MSMEG1436	K.SYGLIEDNVYTFVVHPDSNK.T	2	60.7	46.7	37.0	30.9	88.7	72.0	38.0	32.0
		K.SYGLIEDNVYTFVVHPDSNK.T	3	1094.6	1134.0	730.5	783.2	1445.7	1516.6	803.2	773.7

MSMEG _1439	MSMEG 1437	R.ATVGEVGNAEQSNINWG K.A	2	1087.5	946.4	540.7	455.1	830.8	821.4	477.8	504.7
		R.ATVGEVGNAEQSNINWG K.A	3	122.7	129.4	14.8	19.3	70.9	76.3	13.6	13.2
		R.GASVSDFAEITR.S	2	11.9	11.9	8.9	15.9	14.7	19.5	7.3	11.4
		R.SAGVSIQLLGK.E	2	9.0	9.2	200.0	188.2	10.6	14.1	260.7	241.1
		R.TANIALLHYLDGEK.R	2	220.7	223.5	152.9	157.9	202.8	199.7	104.5	102.2
		R.TANIALLHYLDGEK.R	3	163.6	158.1	42.8	43.5	69.2	61.6	7.9	13.7
		R.TANIALLHYLDGEKR.Y	3	285.1	279.2	174.6	177.9	248.4	232.1	189.2	189.1
MSMEG _1440	MSMEG 1438	K.HVPVFVTEAM#VGHK.L	3	51.5	28.3	19.1	10.4	50.1	48.2	15.3	17.2
		K.HVPVFVTEAM*VGHK.L	3	51.5	28.3	18.1	9.7	50.1	48.2	14.5	16.1
		K.HVPVFVTEAMVGHK.L	3	187.8	222.5	112.9	131.5	162.1	191.3	102.0	109.7
		K.KGPFVDDHLLK.K	3	13.7	10.3	1.3	0.7	17.7	18.1	1.0	1.7
		R.KHVPVFVTEAM#VGHK.L	4	17.0	9.8	4.8	2.6	17.4	15.7	4.1	3.4
		R.KHVPVFVTEAM*VGHK.L	3	6.5	4.1	1.5	0.9	7.2	5.6	1.2	1.0
		R.KHVPVFVTEAM*VGHK.L	4	17.0	9.8	4.8	2.6	17.4	15.7	4.1	3.4
		R.KHVPVFVTEAMVGHK.L	4	25.7	29.3	8.6	10.9	25.4	27.5	4.9	6.2
		R.RSTIIPDFIGHTFAVHDGR. K	4	38.5	44.2	9.7	14.5	82.1	89.5	22.0	25.1
		R.STIIPDFIGHTFAVHDGR.K	2	37.6	24.7	19.2	13.0	44.1	32.1	16.4	12.9
		R.STIIPDFIGHTFAVHDGR.K	4	211.9	117.9	90.1	49.2	247.5	249.2	93.9	86.6
MSMEG _1441	MSMEG 1439	K.RTSHITVIVESRPPK.Q	4	8.7	7.8	1.6	1.7	8.4	8.8	1.7	1.6
		K.SVEEALDILR.W	2	707.4	762.9	360.9	367.2	772.9	762.4	423.2	393.5
		K.VIASAAANAQNNEGLDPS TLVVATVYADEGPTAK.R	3	516.6	769.6	238.8	343.6	516.4	577.6	216.3	244.2
		K.VIASAAANAQNNEGLDPS TLVVATVYADEGPTAK.R	4	485.6	424.3	186.0	165.1	454.8	473.1	166.3	167.3
		R.TSHITVIVESRPPK.Q	2	22.4	17.4	7.0	5.5	26.4	15.9	8.3	2.9
		R.TSHITVIVESRPPK.Q	3	128.1	110.2	61.1	51.4	135.7	116.2	71.5	55.1
		R.WAPQAASEPVAK.V	2	4.7	4.5	0.4	0.4	5.0	5.3	0.7	0.4

MSMEG _1442	MSMEG 1440	K.DYVKEDVAIR.K	2	14.5	8.8	14.0	8.6	13.6	12.8	16.1	14.5
		K.NPESQAQLVAQGVAEQLS NR.V	2	337.1	371.8	352.9	605.5	296.9	273.4	242.0	272.3
		K.NPESQAQLVAQGVAEQLS NR.V	3	1633.1	1715.3	942.7	929.8	1270.6	1248.6	852.1	799.3
		R.AATSDAPAAGTAAAAEAP AESTES	2	37.8	35.9	18.7	17.4	22.3	35.7	5.8	16.9
		R.AATSDAPAAGTAAAAEAP AESTES	3	25.1	25.5	8.8	9.7	13.9	22.7	2.8	7.9
		R.AGIADVEIER.T	2	41.3	34.9	68.2	61.3	27.7	30.2	14.2	51.7
		R.VDIHTARPGIVIGR.R	3	50.4	30.9	8.4	4.2	63.1	63.6	8.6	8.6
MSMEG _1443	MSMEG 1441	K.GSPEWWVANVKPGR.V	2	331.3	406.3	521.9	516.1	399.3	363.3	491.8	410.0
		K.VWINIFPDRPLTK.K	2	371.6	343.1	217.5	205.1	383.9	323.3	220.2	175.1
		K.VWINIFPDRPLTK.K	3	884.4	901.6	456.2	445.0	858.0	897.3	426.4	409.5
		R.GIASGGTSVSFGDYGIQA LEHAYITNR.Q	3	337.2	0.0	200.1	0.0	509.9	366.1	321.5	203.5
		R.VLFELSYPDEK.T	2	1190.3	1119.4	821.5	723.2	1204.0	1240.8	777.9	767.1
MSMEG _1445	MSMEG 1443	K.TAIGYVVSDK.M	2	8.7	0.0	4.4	0.0	11.5	11.1	3.3	3.7
MSMEG _1465	MSMEG 1462	K.FDENAAVIIK.N	2	167.6	160.9	121.3	112.0	197.2	185.6	118.2	106.4
		K.IVSLAPEVL	2	7.5	1.7	8.2	2.3	11.6	15.1	10.2	15.6
		R.RYAGIGDVIVATVK.D	2	94.3	98.5	104.7	110.0	147.1	132.8	111.6	95.2
		R.RYAGIGDVIVATVK.D	3	79.2	70.7	26.5	25.6	145.3	130.0	49.6	47.1
		R.YAGIGDVIVATVK.D	2	613.6	638.7	329.6	369.5	662.7	644.3	319.8	299.2
		R.YAGIGDVIVATVK.D	3	22.1	12.8	3.7	2.3	26.1	26.3	3.6	4.1
MSMEG _1466	MSMEG 1463	R.GASSGGIVTQEAPIHVSN VM*VVDSDGKPTR.V	3	27.5	34.7	6.5	7.9	26.5	26.3	1.9	4.7
		R.GASSGGIVTQEAPIHVSN VM*VVDSDGKPTR.V	4	183.4	210.0	84.8	106.2	175.8	187.0	78.2	67.1
		R.GASSGGIVTQEAPIHVSN VMVVDSDGKPTR.V	3	63.5	72.3	34.6	36.1	62.8	57.1	23.1	22.1
		R.GASSGGIVTQEAPIHVSN VMVVDSDGKPTR.V	4	290.0	320.9	172.6	188.0	291.1	288.3	147.3	131.1

MSMEG _1467	MSMEG 1464	K.LINGAINDLALITGQKPEVR .R	2	51.4	53.6	12.5	12.3	46.4	39.4	11.3	12.3
		K.LINGAINDLALITGQKPEVR .R	3	1392.4	1426.1	1202.0	1281.5	1648.7	1650.0	1231.5	1163.7
		K.VVVNMGVGDAAR.D	2	8.3	7.3	17.4	12.6	5.7	5.3	20.7	22.9
		R.ALGFPFKEN	2	112.4	106.9	33.2	27.8	151.9	173.3	33.8	45.4
		R.EALQQEFNYANVM*QIPG VVK.V	3	74.6	78.6	32.4	33.4	59.7	69.5	20.4	23.7
		R.EALQQEFNYANVMQIPGV VK.V	2	149.5	156.9	165.6	177.4	202.2	178.3	148.9	156.5
		R.EALQQEFNYANVMQIPGV VK.V	3	114.0	117.4	43.6	42.2	78.8	93.1	20.4	23.7
		R.GM#DITVVTTATNDAEGR. A	2	177.6	197.3	105.1	114.2	176.1	199.0	84.9	101.1
		R.GM*DITVVTTATNDAEGR. A	2	170.8	192.4	105.1	114.2	169.2	191.1	84.9	101.1
		R.GMDITVVTTATNDAEGR. A	2	380.5	399.3	348.0	352.1	439.2	444.0	362.5	346.8
		R.LISIALPR.I	2	580.9	552.8	415.9	395.9	769.6	689.3	422.1	402.6
		R.MWEFLDR.L	2	80.9	82.9	61.2	64.5	99.6	104.1	65.4	66.9
MSMEG _1469	MSMEG 1466	R.NANSAYHDEVTLPHSK.L	3	6.0	5.6	1.5	1.4	7.2	7.3	1.5	1.6
		R.VLGGLGVAIISTSSGLLTD R.Q	2	513.7	716.0	289.9	390.3	505.2	544.0	280.5	284.2
		R.VLGGLGVAIISTSSGLLTD R.Q	3	376.5	225.4	198.3	111.5	338.6	357.8	186.9	176.5
MSMEG _1470	MSMEG 1467	K.GTLTLDVAEPISVSR.A	2	1747.0	1798.6	1119.8	1104.4	2016.6	2094.9	1193.3	1198.6
		K.M*EIFGVGYR.V	2	108.3	95.5	77.5	92.4	174.0	177.3	100.7	102.3
		K.MEIFGVGYR.V	2	185.8	184.8	107.5	123.6	239.8	240.8	122.1	122.3
		K.VGQISAVIR.R	2	4.8	4.9	0.3	0.5	4.2	4.7	0.4	0.5
		R.AEDGAIVVTRPDDERR.S	3	0.3	0.2	0.0	0.0	0.7	0.7	0.0	0.0
		R.TLIANLVTGVTEGYTQK.M	2	1066.8	1367.9	667.4	767.8	1109.5	1140.5	647.2	606.6
		R.TLIANLVTGVTEGYTQK.M	3	603.8	573.9	272.2	252.8	619.5	678.3	264.2	250.3

MSMEG _1471	MSMEG 1468	K.AAGVETVVFDR.G	2	207.6	187.0	140.1	127.6	226.1	234.2	145.2	144.9
		K.TEGTVAHKPVGQNISEVR. R	3	4.0	3.4	1.9	1.5	4.5	4.8	2.2	1.8
MSMEG _1472	MSMEG 1469	K.SLGSDNAINVVHATVAALK .L	2	651.3	616.4	388.1	358.1	704.7	624.1	442.3	393.1
		K.SLGSDNAINVVHATVAALK .L	3	1180.8	1161.2	530.7	507.0	1316.1	1185.6	565.9	521.6
		R.AVLEC#AGVHDILAK.S	2	513.4	412.5	376.0	307.5	559.1	492.4	389.0	355.9
		R.AVLEC*AGVHDILAK.S	2	513.4	412.5	376.0	306.9	559.1	492.4	389.0	355.9
		R.GLPIEDVAPAGMLK.A	2	981.7	1009.3	725.9	731.7	1011.0	959.6	669.8	679.8
		R.RGLPIEDVAPAGMLK.A	2	50.8	52.6	324.5	340.3	86.0	73.4	462.1	423.6
		R.VPLIGSTITHPVQGEAAAG VVM*LR.P	3	111.2	131.6	81.8	90.9	44.8	49.5	65.2	81.8
		R.VPLIGSTITHPVQGEAAAG VVM*LRPASPGTGVIAGGAA R.A	4	621.5	638.4	251.5	272.8	594.8	657.0	220.3	269.5
		R.VPLIGSTITHPVQGEAAAG VVMLR.P	3	686.5	673.6	632.1	657.8	386.6	391.0	406.6	445.5
		R.VPLIGSTITHPVQGEAAAG VVMLRPASPGTGVIAGGAA R.A	4	1901.2	1946.2	1035.3	1045.6	2272.3	2286.2	1164.6	1192.1
		R.VPLIGSTITHPVQGEAAAG VVMLRPASPGTGVIAGGAA R.A	5	714.5	696.7	333.9	308.1	829.1	776.0	390.8	369.9
MSMEG _1473	MSMEG 1470	R.GLINTVHHLVEVEEVGK	2	163.5	131.3	78.5	68.5	173.3	144.5	101.7	79.1
		R.GLINTVHHLVEVEEVGK	3	543.9	673.7	297.3	362.4	512.6	584.4	331.9	346.5
		R.GLINTVHHLVEVEEVGK	4	89.7	52.2	45.7	25.6	110.6	104.2	73.9	66.0
MSMEG _1474	MSMEG 1471	K.AFPQGGTVGVDELVAK.G	2	1110.1	1505.6	1165.5	1456.8	1159.1	1250.1	1431.8	1445.7
		K.AFPQGGTVGVDELVAK.G	3	64.2	57.2	180.1	190.2	53.3	52.8	152.3	159.8
		R.FRTEYQVVNVGDINK.A	2	64.5	63.9	326.0	345.0	108.2	89.4	377.9	342.6
		R.FRTEYQVVNVGDINK.A	3	107.6	121.5	39.7	41.9	185.3	180.3	77.6	71.8
		R.KNVPVMFEGGQM*PIHM* R.L	3	7.9	7.3	2.0	2.5	3.8	7.0	1.5	2.3

		R.TEYQVVNVGDINK.A	2	1368.0	1340.9	694.0	710.9	1064.7	1166.8	597.5	602.8
MSMEG _1475	MSMEG 1472	K.ADYADVAVAGTPGEVVR. G	2	153.6	160.5	44.5	42.2	41.4	50.1	22.7	49.4
		R.LGVAVLVTSLHDPLQLAS SITAVDR.L	3	61.7	54.4	26.5	23.5	2.1	3.2	45.8	28.2
MSMEG _1476	MSMEG 1473	R.VQLPAAAPGPVQELR.D	2	108.9	102.2	136.8	128.6	33.9	44.4	161.7	124.3
MSMEG _1479	MSMEG 1476	R.AVGIEALTMLADGK.F	2	20.2	23.8	13.3	7.5	8.7	10.0	15.8	15.1
		R.GVGQAVILASGLDSR.A	2	73.9	90.4	48.4	65.2	24.9	29.1	22.5	48.9
MSMEG _1482	MSMEG 1479	K.ALADLGALPTTDR.R	2	162.5	129.6	67.2	62.1	80.8	87.8	70.4	109.5
MSMEG _1485	MSMEG 1482	R.AAHWEHTVAVTEDGPR.I	3	5.5	3.5	1.9	1.8	10.7	7.7	3.8	3.5
		R.LTDVSHAIEVETHAAEK.R	3	30.2	41.1	10.7	9.0	38.9	35.3	20.5	20.2
		R.LTDVSHAIEVETHAAEK.R	4	45.5	52.0	7.0	7.4	56.6	46.3	5.6	6.1
		R.TSAELDAMAVAGALVASA LR.A	2	11.3	12.1	9.2	11.8	10.9	11.1	20.8	22.2
MSMEG _1492	MSMEG 1490	R.LQVHVTSVTHAIR.R	3	3.3	3.3	3.7	4.3	3.5	3.1	3.6	3.7
MSMEG _1498	MSMEG 1497	K.ADYGPLVTEAALNR.V	2	114.4	133.4	94.4	150.0	108.2	129.3	105.5	119.4
		K.FIDLVNQNADELAR.L	2	168.7	170.8	146.8	165.7	195.7	172.2	188.6	165.9
MSMEG _1512	MSMEG 1511	R.FNETTPYNPSSPYSSTK.A	2	7.5	7.0	3.3	1.8	6.1	8.0	1.3	2.6
		R.YAIDPSTLQDELGWAPK.H	2	105.2	110.5	137.7	155.4	73.5	82.0	134.7	141.9
MSMEG _1513	MSMEG 1512	K.LIDAVVAHGTAGDIAAR.L	2	55.0	52.8	188.6	166.2	63.6	76.0	1039.1	869.6
		K.LIDAVVAHGTAGDIAAR.L	3	163.7	169.0	231.2	226.1	182.4	188.9	217.6	225.5
		R.AVLGEAVFLAPEHK.V	3	84.2	74.2	95.9	90.0	111.2	105.0	119.5	115.6
		R.FVLGVGVGHPEHTGEYR. K	2	58.4	66.0	26.2	21.4	58.5	72.8	21.8	26.9

		R.FVLGVGVGHPEHTGEYR.K	3	47.9	55.3	76.2	80.6	55.5	63.2	78.4	86.2
		R.KPYDALVEYLDVLDAAK.V	2	41.4	41.5	116.1	117.3	58.9	51.0	166.9	156.1
		R.KPYDALVEYLDVLDAAK.V	3	46.8	46.9	68.4	65.5	54.4	59.0	77.8	75.6
MSMEG_1521	MSMEG1520	K.DLTDDQVTVLR.D	2	187.9	137.3	100.1	70.1	155.4	181.5	93.1	98.6
		K.IEIGC*YQGLR.H	2	161.6	119.8	76.6	56.6	131.8	157.1	62.4	75.4
		R.KIEIGC#YQGLR.H	2	36.4	17.1	24.9	10.9	45.9	46.7	24.4	29.0
		R.LVGVDLPR.D	2	57.4	54.4	28.1	24.8	63.9	65.5	21.0	27.7
		R.M#EIALTYIYGIGR.T	2	252.2	266.5	150.8	142.7	200.6	202.6	124.6	117.2
		R.M*EIALTYIYGIGR.T	2	59.3	50.4	28.4	25.8	73.5	67.9	41.5	36.0
		R.MEIALTYIYGIGR.T	2	59.3	50.4	28.4	25.8	73.5	67.9	41.5	36.0
		R.SNEILAATGIDK.N	2	165.3	141.0	97.1	80.6	167.6	187.5	98.5	105.7
		R.TKDLTDDQVTVLR.D	2	4.4	4.2	7.9	5.3	8.9	7.6	11.7	9.4
MSMEG_1522	MSMEG1521	K.STFNNTIVSITDPQGNVIAWASSGHVGFK.G	3	223.8	223.3	107.5	100.3	229.7	248.6	96.0	94.3
		K.STPFAAQLAAENAAR.K	2	1680.1	1881.7	924.0	982.7	1775.2	1820.9	879.4	816.5
		K.STPFAAQLAAENAAR.K	3	176.9	189.3	64.6	75.3	196.0	198.1	60.5	55.8
		R.KSTPFAAQLAAENAAR.K	2	28.8	18.1	17.6	11.9	56.7	57.5	24.8	24.0
		R.KSTPFAAQLAAENAAR.K	3	87.5	52.9	72.1	61.4	148.2	164.8	70.6	83.2
		R.SLQAAGLEVGTISDVTPQPHNGC*RPPK.R	3	474.5	0.0	303.7	0.0	590.5	525.7	283.0	254.3
MSMEG_1523	MSMEG1522	K.SLNTLPFQIAR.E	2	990.5	941.2	515.4	495.1	992.6	1088.7	433.0	527.4
		R.AQIDVPLTEQLIVELYSK	2	362.2	320.5	289.0	243.3	350.7	308.3	267.7	221.9
		R.AQIDVPLTEQLIVELYSK	3	78.1	73.1	72.0	66.7	67.7	65.1	45.9	57.4
		R.ETAGERPIPSWLQVVGER.Q	2	284.5	269.0	166.0	174.3	323.0	300.0	156.4	161.2
		R.ETAGERPIPSWLQVVGER.Q	3	1069.2	1056.2	566.3	572.8	1115.5	1094.6	540.2	566.3
		R.LGVDLVGGDQSFEK.R	2	1540.1	1630.7	937.9	971.0	1341.5	1503.1	740.7	807.5
		R.LGVDLVGGDQSFEKR.P	3	220.2	212.5	118.1	115.9	362.9	331.8	193.8	175.2
		R.RLGVDLVGGDQSFEK.R	2	291.6	212.8	113.9	74.4	356.2	334.6	188.7	165.3

MSMEG_1524	MSMEG 1523	-.M#LISQRPTLSEETVAENR.S	3	607.4	380.1	368.4	249.9	448.8	506.6	253.8	304.9
		-.M*LISQRPTLSEETVAENR.S	3	604.8	373.0	369.5	251.4	435.9	494.6	254.1	306.1
		-.MLISQRPTLSEETVAENR.S	2	169.8	177.2	96.1	100.0	143.2	133.6	59.6	74.7
		-.MLISQRPTLSEETVAENR.S	3	2018.3	1731.5	1305.8	1150.7	1868.7	1906.2	1186.6	1320.4
		K.EDVTDIILNLK.G	2	666.3	606.3	435.3	378.8	584.4	519.6	464.4	364.3
		K.GLVVSSDDDEPVTMYLR.K	2	851.3	794.5	563.1	575.9	551.3	563.4	354.1	359.7
		K.LIIDVETK.N	2	80.6	65.1	52.6	43.1	63.9	52.3	54.0	68.7
		R.DALASAGGTLVELFGLAR.E	2	1652.3	1530.2	1198.7	1074.9	1591.1	1513.8	1298.7	1209.7
		R.DALASAGGTLVELFGLAR.E	3	257.6	240.5	143.6	140.7	318.8	280.2	208.1	179.6
		R.EGVHTVGELVAR.T	2	99.9	68.9	177.9	210.2	108.2	79.8	69.6	58.6
		R.FVIEPLEPGFGYTLGNSLR.R	2	734.9	773.0	488.0	518.6	844.9	818.9	622.1	609.4
		R.FVIEPLEPGFGYTLGNSLR.R	3	97.2	119.5	63.7	72.9	134.2	127.9	71.6	82.2
		R.IDGVLHEFTTVPGVK.E	2	1693.9	1691.3	1630.6	1656.6	1364.8	1322.8	1828.0	1808.1
		R.IDGVLHEFTTVPGVK.E	3	595.4	538.1	344.7	336.9	546.9	486.2	356.8	316.2
		R.IPVDSIYSPVLK.V	2	1439.9	1417.4	1434.6	1413.9	1612.2	1494.7	1638.0	1507.8
		R.PTLSEETVAENR.S	2	17.3	15.4	9.3	10.0	8.7	6.5	2.1	3.3
		R.TLLSSIPGAAVTSIR.I	2	1680.9	1508.0	1156.1	1060.0	1533.9	1488.6	1209.5	1112.3
		R.TLLSSIPGAAVTSIR.I	3	91.8	78.0	40.1	36.7	76.2	73.4	37.1	30.5
MSMEG_1525	MSMEG 1524	K.DVVHTLFAEIGPFYADR.N	3	26.9	27.8	7.9	8.8	26.1	23.4	3.2	4.5
		K.GDNAPMAVIELVR.E	2	42.0	42.1	26.0	29.9	33.5	34.6	11.6	19.9
		R.DKDVVHTLFAEIGPFYADR.N	4	84.9	95.8	23.9	28.6	86.0	83.3	19.3	27.7
		R.KGDNAPM*AVIELVR.E	2	122.5	122.0	83.9	107.6	177.6	197.9	98.2	108.7

		R.KGDNAPM*AVIELVR.E	3	71.3	60.4	8.7	4.8	35.2	63.0	1.7	11.2
		R.KGDNAPMAVIELVR.E	2	357.5	404.0	208.8	220.3	421.4	408.9	266.4	254.5
		R.KGDNAPMAVIELVR.E	3	326.3	307.7	153.9	131.5	415.8	380.7	193.6	166.4
		R.LGGSSSHQSALLANLATSLFEHGR.I	3	387.6	394.5	238.3	237.5	478.5	444.3	304.1	278.7
		R.LGGSSSHQSALLANLATSLFEHGR.I	4	511.5	539.5	262.6	258.3	629.4	613.1	364.4	331.2
MSMEG_1528	MSMEG 1527	K.LGLPLTASPVYGSR.A	2	81.0	86.7	60.2	67.9	64.8	71.0	51.4	51.0
		R.AIDLC#NAGDPVC#GSGDSVPAHR.A	3	58.1	66.9	42.2	46.9	48.4	50.5	45.1	42.0
		R.AIDLC*NAGDPVC*GSGDSVPAHR.A	3	57.9	66.7	41.5	46.5	48.3	50.4	44.4	40.7
		R.IGNAFVQALR.G	2	23.5	19.2	23.7	23.1	23.0	26.5	26.6	29.7
MSMEG_1536	MSMEG 1535	R.QELLRAANVGSAAEYTR.T	3	45.2	40.4	25.0	27.3	35.9	36.8	29.8	31.2
MSMEG_1556	MSMEG 1553	K.HKPTFTPNVDGGDFVIVINADK.I	3	1094.4	844.8	661.5	522.4	1137.0	1136.9	641.1	535.9
		K.VYAGPDHPHAAQQPIPFEIK.Q	3	986.0	1141.1	508.5	558.5	1067.4	1035.9	507.4	457.1
		R.LASAAATLLR.G	2	36.8	36.7	10.2	9.1	39.6	39.2	10.6	9.6
		R.SWYVIDASDVVLGR.L	2	414.7	411.3	246.6	230.5	401.4	454.6	238.7	237.9
		R.SWYVIDASDVVLGR.L	3	29.2	16.0	10.0	3.7	25.4	29.7	2.3	7.6
		R.VVENAIIGM#LPHNK.L	3	229.6	251.1	81.9	90.6	188.6	236.6	62.3	71.2
		R.VVENAIIGM*LPHNK.L	2	35.4	37.8	37.8	41.0	37.1	31.6	42.3	41.1
		R.VVENAIIGMLPHNK.L	2	35.4	37.8	171.3	150.2	37.1	31.6	129.0	142.4
		R.VVENAIIGMLPHNK.L	3	119.1	98.1	50.1	47.9	127.8	125.3	53.5	50.4
MSMEG_1557	MSMEG 1554	K.APLVTVDRVDQFDIYAHLDGGGPSGQAGALR.L	4	97.3	82.8	83.2	77.0	218.6	210.1	132.1	132.5
		R.ALILVQPEDRPALK.K	2	436.4	466.2	303.0	301.0	491.0	436.3	333.1	304.5
		R.ALILVQPEDRPALK.K	3	1570.7	1707.8	518.8	547.3	1529.1	1469.8	491.6	427.7
		R.ALILVQPEDRPALKK.A	2	5.2	7.3	0.1	0.2	16.9	12.4	0.3	0.2
		R.ALILVQPEDRPALKK.A	4	107.5	111.3	28.8	30.7	260.0	234.5	92.9	85.2
		R.EPVIIDRPIQTVGR.R	2	691.7	67.7	408.0	81.1	792.8	686.7	472.2	416.6
		R.LVPGTGQFNLDGR.T	2	2357.3	3238.7	1596.4	2000.3	2565.0	2943.1	1975.6	2015.3
		R.VDQFDIYAHLDGGGPSGQAGALR.L	2	118.1	96.9	37.5	31.3	97.6	88.0	29.6	23.4

		R.VDQFDIYAHLDGGGPSGQAGALR.L	3	1629.2	1683.2	801.9	764.8	1602.6	1583.5	734.7	738.5
MSMEG_1567	MSMEG 1564	R.ALLTGFLLATLAGDK.T	2	14.0	11.7	25.2	19.6	30.6	27.0	24.8	20.4
		R.IPGLVLTDPGDR.M	2	48.0	50.4	142.9	137.2	72.6	73.5	126.9	137.9
MSMEG_1574	MSMEG 1571	K.YGLTYPGVGFVVWR.N	2	62.4	61.0	115.3	108.0	83.5	73.1	162.1	140.9
		R.AGYTQVMQC#LSQTAR.W	2	97.1	116.6	119.9	123.6	50.1	56.2	54.3	77.0
		R.ALKDDIITVLGQLDALKPR.G	3	135.6	131.6	187.1	170.9	113.0	95.2	224.7	167.5
		R.ALKDDIITVLGQLDALKPR.G	4	29.3	21.0	22.8	20.7	30.3	24.4	31.8	30.5
		R.GQFDQLQPFAH	2	6.9	3.2	13.8	18.6	6.7	11.9	36.9	39.4
		R.LNLATFVTTWMDPEAEK.L	2	95.5	85.9	103.9	98.7	47.9	49.0	78.7	73.8
		R.PGNQVVGQYYNFLR.L	2	157.3	150.7	176.9	170.7	99.0	94.1	159.3	152.1
MSMEG_1577	MSMEG 1574	K.AGDVVVLSGPLGAGK.T	2	49.4	56.1	49.5	60.2	22.1	35.2	24.9	39.6
MSMEG_1581	MSMEG 1579	R.YAELVDAGDFDGVGQLLGR.G	2	12.3	13.4	6.4	9.9	9.7	10.5	4.3	5.4
MSMEG_1582	MSMEG 1580	K.EKPQEGTVVAVGPGR.W	3	4.0	4.0	1.6	1.4	5.2	5.4	2.1	2.4
		K.ILVQANEAETTTASGLVIPDTAK.E	2	525.9	558.9	315.5	349.8	633.6	585.3	393.5	348.8
		K.ILVQANEAETTTASGLVIPDTAK.E	3	758.6	581.2	528.2	402.8	933.4	883.3	631.3	597.5
		K.RIPLDVAEGDTVIYSK.Y	2	478.0	517.3	594.9	545.1	852.5	751.5	887.4	703.5
		K.RIPLDVAEGDTVIYSK.Y	3	606.9	654.4	420.6	426.4	1044.3	1061.3	688.2	659.0
		K.YNGEEYLILSAR.D	2	404.7	378.5	273.7	255.7	400.1	443.7	304.5	304.0
		K.YNGEEYLILSAR.D	3	64.4	42.7	17.3	10.4	76.2	57.3	24.4	17.8
		R.IPLDVAEGDTVIYSK.Y	2	1355.5	2171.0	865.1	1374.0	1154.0	1329.5	773.0	831.8
MSMEG_1583	MSMEG 1581	K.AADAVSEALLASATPVDDKK.A	2	108.3	104.7	81.8	68.0	76.7	77.3	48.5	53.4
		K.AADAVSEALLASATPVDDKK.A	3	454.1	441.8	239.8	220.4	474.2	438.6	262.1	252.5

		K.AAVEEGIVTGGGAALVQAR.S	2	323.6	303.0	182.0	200.7	255.8	254.7	112.1	174.7
		K.AFLDDLAIVTGGQVVNPDVGLLLR.E	3	164.7	221.3	125.2	184.0	217.0	208.0	78.6	120.7
		K.ISSLPDLLPLLEK.V	2	597.0	558.9	344.4	336.1	473.5	404.1	311.4	244.9
		K.SFGGPQVTNDGVTIAR.E	2	867.8	721.6	562.6	497.3	915.4	921.9	499.0	552.2
		K.TNDVAGDGTTTATVLAQALVR.A	2	149.3	154.5	177.6	182.0	66.5	73.0	64.3	57.0
		K.TNDVAGDGTTTATVLAQALVR.A	3	307.0	360.1	231.9	259.1	374.6	335.8	213.1	203.1
		R.DEQVGELVGEAMTK.V	2	283.4	327.3	406.0	462.6	172.0	204.7	222.9	245.8
		R.DKISSLPDLLPLLEK.V	2	61.9	75.6	44.2	64.5	107.5	92.5	50.7	52.3
		R.EIDLEDPYENLGAQLVK.S	2	602.9	586.1	932.6	839.3	480.2	477.3	1015.0	940.9
		R.EVGLEVLGSAR.R	2	60.0	54.7	114.6	96.5	58.3	50.1	126.9	109.6
		R.M*ILTTETAVVDKPADEDEHGHGHHHGHAH	5	43.2	43.0	14.3	18.2	15.1	12.6	0.4	3.5
		R.NVAAGANPIALGSGISK.A	2	658.9	542.2	775.7	738.0	794.5	796.9	718.3	726.1
MSMEG_1602	MSMEG 1600	K.EGVSAEAALGLLR.R	2	82.8	82.0	23.7	24.0	77.7	77.8	39.4	34.0
		R.AAAAALVEAGADAVK.V	2	170.8	133.1	330.5	247.9	180.8	162.5	441.3	405.5
		R.GPLGTVIHQLTGGLR.A	3	29.6	22.3	2.6	2.4	15.0	18.6	0.9	2.4
		R.ISGLPVVDDTGELVGIITNR.D	2	206.0	196.7	105.9	113.8	164.4	159.5	304.1	230.4
		R.ISGLPVVDDTGELVGIITNR.D	3	107.5	114.3	68.3	63.6	98.4	96.9	67.5	65.8
		R.NLPVAEQAGQVETVK.R	2	16.1	11.2	50.1	56.4	5.7	3.0	23.6	32.7
MSMEG_1603	MSMEG 1601	K.TFISELDVPVVAGGVLDHR.T	3	204.0	173.4	196.9	189.0	197.2	154.4	189.3	168.5
		R.FEIPVIAHPTDALVSPEFAIEMGR.L	3	317.9	313.4	279.4	253.1	159.3	211.7	177.6	198.6
		R.LLQQLHAAPLDPDLLGAAVAR.I	3	253.5	243.0	466.0	406.1	256.5	234.9	904.3	605.3
		R.YVHVLADGDIHSSGDLAK.A	3	60.7	57.2	160.6	180.6	35.3	44.2	33.7	53.7

		R.YVHVLADGDIHSSGDLAK.A	4	71.9	65.9	54.0	56.5	57.6	53.2	72.9	79.6
MSMEG_1605	MSMEG 1603	R.DPDTAARLRAEDDAVDAEHR.H	3	0.1	0.1	0.4	0.3	4.5	5.4	0.7	0.7
MSMEG_1610	MSMEG 1608	R.FLEALTGVTNPEGK.R	2	758.8	698.5	627.5	580.9	668.5	643.1	656.5	659.0
		R.LAGVQYHPEVLHSPHGQQVLSR.F	3	72.8	47.7	142.0	158.4	46.5	45.5	480.1	547.9
		R.LAGVQYHPEVLHSPHGQQVLSR.F	4	44.8	49.1	108.8	109.9	9.6	22.9	31.4	54.0
		R.LAGVQYHPEVLHSPHGQQVLSR.F	5	343.1	268.2	251.7	200.6	279.2	272.8	197.2	228.0
		R.VVLDITSKPPGTIEWE	2	12.6	15.4	166.5	175.6	1.2	6.6	79.0	92.9
		R.VYSEVVPHTAGVEEIK.A	2	18.7	15.7	77.8	67.0	5.4	6.9	44.1	55.9
		R.VYSEVVPHTAGVEEIK.A	3	71.5	74.3	191.1	187.9	30.2	37.8	94.0	129.6
MSMEG_1614	MSMEG 1612	R.TVLDDVSLDVRR.G	2	76.4	83.8	2.0	1.0	80.4	84.7	1.0	1.9
MSMEG_1634	MSMEG 1633	R.ETIAALGGLPGADHGEHEGR.H	3	9.5	0.0	0.4	0.0	14.8	15.1	0.5	1.3
		R.SALTEAASAQRPSPAVAAALSGVR.Q	3	1129.1	1204.5	728.1	785.6	1591.1	1632.4	783.1	756.9
		R.YDSLMAHAAAAPGSSLGQQIYVTR.V	3	214.1	240.6	233.6	244.7	305.5	312.1	196.5	200.1
MSMEG_1635	MSMEG 1634	K.APVLLIPC#LEGR.P	2	35.1	34.8	90.2	87.1	0.6	3.2	101.3	91.4
		K.QAAEVVGIPFDK.Y	2	53.6	46.8	15.4	10.0	21.6	19.0	14.2	10.5
		K.RLPAEQFAHWDTW	2	176.5	143.7	71.7	54.9	81.4	97.7	69.2	65.6
		K.YSQAGLFPIAYTIGTDFK.K	2	41.9	46.6	17.4	22.5	62.1	60.4	54.2	58.2
		R.GLGSAWTTLHLLGEGEK.Q	2	547.8	539.3	385.8	360.5	289.0	254.9	398.5	396.6
		R.GLGSAWTTLHLLGEGEK.Q	3	599.1	517.9	280.0	247.8	304.4	271.2	288.4	263.5
		R.LPAEQFAHWDTW	2	119.7	127.8	67.2	73.3	54.7	59.4	58.5	67.9
		R.SNAIPYLEQEAAER.G	2	1218.8	1310.0	585.3	558.1	825.3	868.9	610.0	599.2

		R.SNAIPYLEQEAAER.G	3	71.8	63.5	13.6	18.7	33.0	35.4	13.6	11.5
MSMEG _1642	MSMEG 1641	K.FEGHDVHAEYASLR.S	3	4.7	3.6	6.8	4.0	4.4	4.3	4.8	4.2
		K.FEGHDVHAEYASLR.S	4	5.0	3.2	5.0	3.1	2.9	3.7	2.3	3.5
		K.TLLDNISIDAR.P	2	20.3	24.5	54.7	61.6	17.0	18.5	38.2	33.2
		R.ATDNDIVIPDVLASR.H	2	11.4	10.7	19.0	19.6	13.1	12.5	18.3	19.9
		R.IGLDQLSWITPAR.W	2	13.6	12.8	11.2	11.7	12.3	14.4	9.0	14.6
		R.TPEAPPASASSEAPADLG APAK.T	2	99.1	96.9	29.8	26.8	104.2	96.5	31.2	28.4
		R.TPEAPPASASSEAPADLG APAK.T	3	26.2	31.3	9.0	11.5	22.2	30.9	7.6	14.1
		R.TPPPAPANSSQAPTTMGP AAAPR.G	3	6.1	9.8	7.2	8.6	4.0	7.1	3.7	6.6
		R.YPSAPQHGYPNGPQTGY PSGPQR.G	3	88.9	90.7	66.6	69.2	74.8	93.4	58.3	77.3
MSMEG _1647	MSMEG 1645	K.LTGDVAPDVWEVAGHVS PNPGGVGPLTR.A	3	283.0	293.5	295.4	289.6	256.9	264.1	302.1	313.7
		R.AFLLTNVVEAEESK.L	2	194.3	198.3	348.8	344.7	159.8	154.0	311.0	326.6
		R.IDPAKDADGLHPTNLGR.L	3	2.6	1.8	6.5	3.6	4.6	6.6	11.1	11.1
		R.RFDVPIAGAHVVVIGR.G	3	55.4	55.4	27.9	33.7	35.2	44.5	32.5	30.3
		R.RSENATVTLC#HTGTR.D	3	1.3	0.7	1.2	0.4	2.1	1.2	2.5	1.1
		R.RSENATVTLC*HTGTR.D	3	1.3	0.7	1.2	0.4	2.1	1.2	2.5	1.1
		R.TPGLGTVLVGDDPGSQA YVR.G	2	72.8	90.0	99.8	105.6	66.3	76.2	91.6	104.8
MSMEG _1650	MSMEG 1649	R.GRPSYPPEAIDWLLPDGA HDVLDLGAGTGK.L	3	6.0	14.2	31.5	29.5	5.5	7.1	35.6	38.1
MSMEG _1652	MSMEG 1651	K.AFVNALTLHSHVANIGDV R.S	3	168.6	153.6	344.3	333.1	230.4	210.7	274.5	255.4
		K.FPGFTEPDPSYHGVVFAE LGAPAYALK.A	3	996.2	1051.8	573.9	594.0	842.4	982.9	591.4	667.6
		K.LGIETTFVENPDDLESWR. A	2	563.7	575.9	232.8	242.5	578.5	578.6	316.4	302.0
		K.LGIETTFVENPDDLESWR. A	3	25.5	31.6	5.2	5.2	25.7	26.1	3.7	4.6
		R.DLGSAAAPFNAFLIAQGLE TLSLR.V	3	114.1	133.2	59.1	76.5	114.0	155.5	70.2	96.5

		R.IGNPTTDVVEQR.I	2	6.7	5.4	8.4	8.1	4.5	7.1	4.0	3.8
		R.LAVGLEGIDDIIADLEQGFAAAR.P	3	26.2	35.3	10.8	16.9	6.7	14.3	3.0	6.9
		R.LYGGTYNLLHYTLPK.L	2	316.5	317.9	153.5	176.3	286.8	264.5	171.3	151.1
MSMEG_1654	MSMEG 1652	K.ALAEQEEAIVSELNSVQGK.T	2	1043.0	1097.2	649.3	642.1	723.6	821.9	580.4	708.0
		K.ALAEQEEAIVSELNSVQGK.T	3	450.1	554.7	250.8	309.2	407.0	367.3	362.1	294.9
		K.ALIAFYEEQIEDAYK.T	2	1110.6	1127.3	707.1	669.1	811.6	820.7	746.5	721.9
		K.ALIAFYEEQIEDAYK.T	3	63.6	85.9	21.1	31.2	44.3	45.2	22.8	29.2
		K.FAEAAGIDVK.T	2	96.9	69.8	42.7	30.4	61.6	67.8	38.7	46.7
		K.GISNFHSPSDVIVDASM*PAM*IR.L	3	227.1	228.1	56.9	75.9	99.2	141.6	34.8	54.3
		K.GISNFHSPSDVIVDASMPAMIR.L	3	710.8	778.0	407.7	419.7	509.6	379.6	417.3	317.5
		K.GYNVPDYPANPK.T	2	40.1	32.1	39.5	34.7	25.9	22.8	33.6	38.4
		K.HVHQLVEENHLR.W	2	3.0	0.2	0.6	0.2	3.4	0.1	2.1	0.4
		K.HVHQLVEENHLR.W	3	27.2	7.8	11.5	2.3	17.1	4.9	9.5	2.2
		K.ILGSAVNPVLR.E	2	295.6	217.2	110.7	79.4	308.0	123.8	171.4	63.6
		K.IQIMPQVWAMR.Y	2	393.8	387.2	252.2	226.6	325.4	253.4	304.2	219.8
		K.KALIAFYEEQIEDAYK.T	2	71.5	75.7	107.8	91.9	75.6	82.4	213.6	168.2
		K.KALIAFYEEQIEDAYK.T	3	33.0	32.1	14.9	17.1	39.7	35.0	38.0	20.1
		K.LDAGDIIDSM*YMSK.K	2	315.8	381.7	45.4	62.6	215.7	249.9	36.4	57.6
		K.LDAGDIIDSMYM*SK.K	2	56.9	63.6	30.9	32.6	46.0	30.4	29.4	25.1
		K.LDAGDIIDSMYMSK.K	2	572.9	553.4	340.1	319.3	407.4	328.7	359.4	269.8
		K.LDAGDIIDSMYMSK.K	3	14.1	18.6	1.3	2.1	5.0	6.5	0.7	0.8
		K.LFDELGVNVNNGLSDLYDK.I	2	274.1	306.0	154.0	153.7	118.5	161.3	62.3	118.1
		K.LPNISASVPQLLAAIK.E	2	1103.9	992.2	778.6	584.0	1386.9	771.0	1285.9	674.2
		K.LPNISASVPQLLAAIK.E	3	58.0	83.6	9.5	30.2	61.5	53.8	33.7	31.3
		K.MLSIVPLMAGGGLYETGAGGSAPK.H	2	197.5	182.0	111.0	112.1	198.1	208.9	132.9	162.9
		K.SGEEIVLKPEVK.L	3	39.4	30.8	2.4	2.8	26.8	27.3	3.3	5.7
		K.TGVMFSLHVK.A	2	47.9	46.2	5.2	5.4	41.6	38.2	11.3	8.7
		K.THGQFDPTTM*GTVPNVGLM*AQK.A	3	160.6	178.8	39.7	40.1	89.4	93.6	16.8	35.4

		K.THGQFDPTTM*GTVPNVG LMAQK.A	3	160.6	178.8	54.9	54.7	95.3	114.0	39.2	48.4
		K.THGQFDPTTMGTVPNVG LMAQK.A	3	786.4	816.2	438.2	420.9	411.1	478.6	327.1	382.0
		K.TSDISVAAR.I	2	0.2	0.1	0.0	0.0	0.1	0.1	0.0	0.0
		K.TVDIGGYYYPDPEK.T	2	1131.8	1119.6	773.7	746.0	894.6	883.7	909.4	800.8
		K.VSHPIVFGHAVK.V	3	5.2	3.4	0.2	0.2	4.5	3.3	0.6	0.6
		R.DYLTDLFPILELGTSAK.M	2	925.7	1188.2	614.1	723.7	751.0	973.1	716.6	894.6
		R.DYLTDLFPILELGTSAK.M	3	54.7	65.7	27.8	36.7	46.1	58.9	32.9	49.6
		R.ILAEFGDHLTEEQR.V	2	457.9	418.7	273.2	264.4	336.1	326.1	278.5	253.7
		R.ILAEFGDHLTEEQR.V	3	781.9	750.1	385.9	373.5	511.4	520.1	336.7	377.1
		R.KFAEAAGIDVK.T	2	27.4	17.2	8.6	6.6	21.2	21.7	10.0	13.1
		R.LSGM*PVVFWLDTERPHE VELR.K	4	112.0	125.9	29.0	34.0	103.5	87.4	28.0	36.2
		R.LSGMPVVFWLDTERPHE VELR.K	3	304.2	335.3	154.0	147.3	218.0	273.4	125.7	218.0
		R.LSGMPVVFWLDTERPHE VELR.K	4	397.0	450.3	181.8	220.4	221.7	407.1	138.6	287.3
		R.MYQEMINFC#K.T	2	196.7	192.9	112.2	108.5	160.1	144.9	119.1	125.8
		R.MYQEMINFC*K.T	2	196.7	192.9	112.2	108.5	160.1	144.9	119.1	125.8
		R.VPDNLGELGALTQDPSAN IIK.L	2	577.9	621.4	273.1	312.6	411.1	390.6	337.3	307.1
		R.VPDNLGELGALTQDPSAN IIK.L	3	615.6	810.0	406.1	497.9	505.1	463.5	551.5	392.9
		R.WDSLGEFLALGASLEDM* GNK.T	2	36.2	42.2	8.1	7.2	50.5	26.3	5.9	10.6
		R.WDSLGEFLALGASLEDM GNK.T	2	90.2	96.8	43.8	47.4	58.4	105.5	45.0	78.6
MSMEG _1655	MSMEG 1653	R.GPFLLDVAHLADDIER.R	3	15.5	14.2	0.9	1.6	27.3	22.9	1.1	2.1
		R.VTELITEFVDR.H	2	21.0	22.1	5.2	4.0	35.4	29.0	4.3	7.4
MSMEG _1656	MSMEG 1654	R.IDYHLVTPGLAGR.L	2	21.7	18.1	23.2	30.0	31.2	22.0	18.0	21.4
MSMEG _1662	MSMEG 1660	K.APFEPLTPGGFR.A	2	78.9	55.1	72.4	55.8	112.1	103.1	90.4	95.6

		K.LYDLPIVGDVR.G	2	51.2	56.1	81.1	84.7	68.3	62.1	71.0	79.2
		R.GEGFFYGIELVK.D	2	22.4	19.0	32.9	53.2	33.4	32.7	30.1	45.4
		R.GFLTPALWEAGLYC#R.A	2	146.9	109.9	88.7	74.3	238.7	238.0	144.0	129.7
		R.VFFTTGGGEAVESAWK.L	2	97.3	83.6	238.3	188.0	145.3	117.2	219.5	231.1
MSMEG _1663	MSMEG 1661	R.ALPGVISTETLVYLK.L	2	30.5	27.0	66.0	64.0	28.8	26.4	40.1	42.0
		R.PGAFQLDELSK.A	2	97.7	103.1	6.6	8.8	98.4	81.1	7.0	8.2
MSMEG _1665	MSMEG 1662	K.LADEHAEQFVAEEVSQTG KPVR.L	3	291.2	284.9	167.1	209.1	234.4	207.1	248.1	227.8
MSMEG _1669	MSMEG 1666	K.DLVVNMEPFFDAYR.A	2	104.6	114.6	90.8	102.3	174.9	167.5	90.5	86.4
		R.FNPENPDAAGWQSFR.V	2	398.1	430.1	299.3	298.2	663.8	713.1	319.5	314.0
		R.GIQVTQAIQEVK.R	2	209.4	184.4	261.3	252.5	378.4	447.2	217.1	268.4
		R.LDILNEVDGVWR.C	2	107.0	96.4	148.2	148.0	230.5	223.3	156.7	159.8
		R.LLNLLHYVK.W	2	35.6	37.2	26.1	33.3	80.0	79.4	27.1	31.8
MSMEG _1670	MSMEG 1667	K.EAIDAVLDLEK.M	2	200.0	201.4	117.7	94.8	307.2	259.6	104.7	77.9
		K.LPDITEFAR.T	2	142.4	124.0	112.6	104.1	274.7	260.9	88.2	79.1
		K.TTSNAHTLTGDGLGIVFR. K	2	135.0	156.8	202.9	246.0	207.2	221.2	185.2	207.9
		K.TTSNAHTLTGDGLGIVFR. K	3	148.7	141.5	171.3	168.9	273.4	249.8	180.2	153.8
		R.GALQQSMDNNAAVFR.T	2	90.8	78.9	171.6	144.3	110.5	92.8	97.4	96.2
		R.LGTNSLLDINVFGR.R	2	141.0	125.7	217.5	193.1	211.6	141.1	202.0	129.1
		R.SMVLEVLEGR.G	2	71.6	55.3	113.8	94.8	127.7	98.7	97.1	99.9
		R.YAPTIVDLAPR.D	2	115.2	103.1	171.3	154.1	167.5	134.4	101.1	110.0
MSMEG _1671	MSMEG 1668	R.IDFNYVAER.W	2	9.2	5.8	5.1	2.1	19.8	23.3	4.2	7.2
MSMEG _1672	MSMEG 1669	R.VSPQAYNEVIETYK.T	2	230.3	264.8	290.3	319.5	397.5	393.6	263.5	239.4
MSMEG _1676	MSMEG 1673	R.IVDDISETPDGTQQLGR.L	2	210.8	169.8	127.0	80.6	96.8	134.5	137.7	162.4
		R.LAIIDEVIKPR.Y	2	25.9	24.9	9.0	10.0	1.0	0.9	5.4	5.8

MSMEG _1679	MSMEG 1676	R.GVNAADAFTIAQVAIGLLR .Q	2	23.5	24.7	165.0	176.9	76.7	92.9	502.1	477.3
		R.GVNAADAFTIAQVAIGLLR .Q	3	7.3	5.9	35.7	34.2	38.3	41.9	53.1	55.6
		R.VASTLAEGGFDVTER.Y	2	130.8	153.8	275.4	307.3	272.2	229.6	231.4	250.7
MSMEG _1680	MSMEG 1677	R.AALAAGGEAGPVR.S	2	0.2	0.2	4.9	3.2	4.9	3.2	7.3	5.4
		R.SAGM#IVVEDVSWPVTDL R.V	2	28.1	32.4	50.4	58.5	60.2	61.3	44.2	38.9
		R.SAGM*IVVEDVSWPVTDL R.V	2	35.7	41.3	69.6	80.3	108.3	106.5	59.0	54.1
		R.SAGMIVVEDVSWPVTDLR .V	2	155.6	158.2	469.4	465.4	664.1	621.8	459.9	431.5
		R.VDFADDPIGDLAALWQV WQPQKK.D	3	8.2	11.2	24.0	30.4	47.7	71.0	33.9	61.2
MSMEG _1681	MSMEG 1678	K.AM#SNIAM#LLEEAGSSLR .D	2	26.1	27.7	99.0	114.4	66.2	66.0	53.3	52.0
		K.AM#SNIAM#LLEEAGSSLR .D	3	7.3	0.0	33.1	0.0	21.1	24.5	15.2	16.5
		K.AM#SNIAMLLEEAGSSLR. D	2	8.9	9.7	41.6	44.8	21.9	20.9	20.8	20.6
		K.AM#SNIAMLLEEAGSSLR. D	3	2.6	1.8	15.4	13.9	7.8	8.4	4.6	6.8
		K.AM*SNIAM*LLEEAGSSLR. D	2	8.7	9.7	41.6	44.8	21.8	20.9	20.8	20.6
		K.AM*SNIAM*LLEEAGSSLR. D	3	2.7	1.8	22.3	21.3	9.4	10.2	13.0	14.1
		K.AM*SNIAMLLEEAGSSLR. D	2	8.7	9.7	41.6	44.8	21.8	20.9	20.8	20.6
		K.AM*SNIAMLLEEAGSSLR. D	3	2.7	1.8	22.3	21.3	9.4	10.2	13.0	14.1
		K.AMSNIAM#LLEEAGSSLR. D	2	10.8	14.6	55.0	69.6	29.9	30.7	26.8	26.6
		K.AMSNIAM#LLEEAGSSLR. D	3	5.5	1.8	29.6	13.9	16.9	17.6	13.3	15.1
		K.AMSNIAM*LLEEAGSSLR. D	2	10.8	14.6	55.0	69.6	29.8	30.7	26.8	26.6

		K.AMSNIAM*LLEEAGSSLR.D	3	5.6	1.8	36.6	21.3	18.4	19.4	21.6	22.4
		K.AMSNIAMLLEEAGSSLR.D	2	34.2	51.8	167.6	256.4	83.2	88.7	96.1	93.9
		K.AMSNIAMLLEEAGSSLR.D	3	26.6	21.8	152.0	125.0	64.7	67.6	87.4	84.4
		K.DTYPEQNLDNDLC#QAVVAGGVVYLR.G	3	2.8	3.1	17.5	25.0	12.1	27.9	16.6	18.7
		K.GVFPVSTGLVVEALAR.P	2	58.7	53.9	259.5	245.5	134.3	130.5	147.0	142.0
		K.GVFPVSTGLVVEALAR.P	3	1.6	0.5	9.5	5.3	4.4	6.2	1.9	2.2
		K.VTVYLTDIR.Y	2	184.8	163.0	799.9	683.8	647.0	710.3	718.6	719.2
		R.ESVGIGDVAAQTEK.A	2	38.8	36.5	183.0	170.7	157.4	169.7	184.1	181.7
		R.GQIGQDLETR.E	2	0.1	0.0	2.2	2.5	1.3	1.3	3.3	3.2
MSMEG_1682	MSMEG1679	K.IHGRPGFHAETSAGTIDAR.Y	3	0.1	1.6	3.1	2.4	12.6	8.7	0.9	1.5
		K.IHGRPGFHAETSAGTIDAR.Y	4	0.0	0.0	0.0	0.0	6.6	5.5	0.0	0.1
		R.GAEHVTIAVSGAHGGHTVDFR.S	2	1.5	0.8	3.1	2.9	41.3	32.8	2.3	3.5
		R.GAEHVTIAVSGAHGGHTVDFR.S	3	67.8	43.6	27.4	28.5	502.1	448.5	36.3	47.8
		R.GAEHVTIAVSGAHGGHTVDFR.S	4	112.6	106.6	58.9	52.2	738.4	838.1	40.1	60.0
		R.GAEHVTIAVSGAHGGHTVDFR.S	5	70.6	48.9	26.5	17.3	596.0	594.4	23.0	25.2
		R.GSSFLWGVWHDAK.Y	2	344.0	388.0	7.5	7.0	162.4	166.1	10.0	6.2
		R.PGFHAETSAGTIDAR.Y	2	3.4	2.2	4.9	5.7	44.0	28.7	1.3	1.8
		R.PGFHAETSAGTIDAR.Y	3	25.5	21.0	12.2	12.5	153.5	143.1	10.9	11.5
		R.RGSSFLWGVWHDAK.Y	3	7.2	13.2	12.4	7.5	58.8	55.4	4.6	5.4
		R.SLAASGITLLGR.A	2	13.4	6.8	58.2	52.2	407.1	355.0	55.9	54.9
MSMEG_1683	MSMEG1680	K.GVYDLESIFR.F	2	0.0	0.0	34.4	43.3	0.8	0.5	37.1	31.7
MSMEG_1694	MSMEG1690	R.LANPPLLVPVLR.A	2	20.9	19.2	68.5	67.6	31.8	27.0	35.1	38.6

		R.LITATVDEGLNEIAYIVPGLGDAGDR.Q	3	18.6	19.3	9.9	10.4	17.8	28.1	6.7	11.1
MSMEG_1696	MSMEG1692	R.ENLSELEFNLLMLVAPVLR.K	2	176.9	182.5	177.8	161.8	186.4	190.3	224.5	207.6
MSMEG_1701	MSMEG1697	R.IHAYEGHDLAHVVHPVR.T	3	3.8	2.6	18.0	14.4	4.6	4.0	13.2	16.2
		R.IHAYEGHDLAHVVHPVR.T	4	17.9	19.7	12.9	13.2	17.0	18.9	11.9	17.8
MSMEG_1704	MSMEG1700	K.PLPIVTGQDAELASVK.S	2	3.3	3.1	218.8	192.2	1.3	0.7	200.7	250.7
		K.SIVAGEQTQTVFK.D	2	1.9	3.6	237.5	233.5	3.4	4.2	205.3	224.1
		K.VGVLQANYIVDTLGVADGK.G	2	28.7	31.5	251.8	286.0	19.3	26.0	259.7	243.9
		K.VVPSYLLDPVSVDK.S	2	148.6	149.6	380.6	382.3	143.8	114.7	365.7	357.4
MSMEG_1712	MSMEG1708	R.TGWDAVLQETK.N	2	1.3	5.4	19.7	16.2	2.8	6.0	13.6	12.5
MSMEG_1713	MSMEG1709	R.AFLAELNPGFASFVEDK.I	2	18.2	24.0	28.7	32.7	19.4	25.9	14.8	18.1
MSMEG_1734	MSMEG1730	K.EDEEALDRWEGAELGIHK.K	3	8.0	0.0	10.7	0.0	12.2	10.4	14.7	15.0
		K.VFVVLYDMTK.E	2	14.7	14.2	5.3	5.4	16.5	16.9	0.9	2.1
		R.LTFGGEDIGWEGALATVVEDPLSK.V	3	16.8	0.0	23.6	0.0	16.4	22.2	19.9	25.2
		R.YIGVMADAAEIAGAPPEYVHDLR.T	3	51.8	42.4	75.4	51.3	38.0	36.3	48.0	50.5
MSMEG_1735	MSMEG1731	R.DQILPHEDSDAAAVLEQVFAQR.G	3	146.6	153.7	153.0	157.7	116.9	112.6	163.0	143.2
		R.IAMYHALGEAVAPIR.L	3	43.6	40.2	19.3	21.8	15.7	16.5	12.8	9.0
		R.IVILGGGPAGYEAALVAAAR.G	2	132.6	118.2	257.2	260.7	164.4	137.6	221.2	281.0
MSMEG_1753	MSMEG1750	R.IAYGTVLSGDQYLHSVPTR.D	3	88.2	91.6	91.7	94.7	58.7	55.2	84.7	80.4
MSMEG_1757	MSMEG1754	R.LLGPLTEADIAQR.C	2	1.6	2.3	14.0	14.8	2.4	3.6	12.7	14.0
MSMEG_1760	MSMEG1758	R.VVVADLDEPGAAATAAR.I	2	71.8	76.3	136.0	134.6	43.5	57.6	95.7	116.8

MSMEG _1803	MSMEG 1801	R.LAVDEAC#TTLIR.S	2	141.0	167.6	287.7	370.7	127.7	132.7	319.5	302.9
		R.VAAM*LENLAVVR.T	2	37.7	41.4	48.1	51.6	27.5	24.9	40.4	40.5
		R.VAAMLENLAVVR.T	2	165.2	184.0	114.0	115.5	134.4	148.2	99.0	94.8
MSMEG _1807	MSMEG 1807	K.AASGDAVTAPMQGTVVK.V	2	71.3	66.4	88.6	82.4	41.7	37.0	60.7	61.6
		K.APLVPGTPDPVKDADEVVAFAK.E	2	180.3	159.6	246.5	228.8	143.4	111.0	234.9	209.0
		K.APLVPGTPDPVKDADEVVAFAK.E	3	3512.6	3731.4	5757.9	5943.0	2859.0	2749.0	6234.7	6070.8
		K.DAGLASVAVYAEPDADAPHVR.L	2	552.7	559.5	432.9	463.5	510.5	571.6	390.1	502.8
		K.DAGLASVAVYAEPDADAPHVR.L	3	2380.1	2103.7	2878.9	2619.0	1909.3	1592.0	2480.9	2333.0
		K.DAGLASVAVYAEPDADAPHVR.L	4	111.5	94.5	65.1	52.8	75.1	73.8	63.0	57.3
		K.DGTITGLAVEAGAAITQGTVIAEIK	3	104.0	107.0	113.1	125.0	96.4	75.8	139.0	110.6
		K.IANGEPLDITEDPTPR.G	2	2323.9	2214.9	2473.2	2348.4	2711.1	2722.7	2887.9	2912.8
		K.IANGEPLDITEDPTPR.G	3	88.6	108.4	71.6	104.1	54.0	69.0	69.4	89.7
		K.LVEEAPAPFLTDAQR.K	2	3372.0	3278.7	3210.6	3121.1	3791.3	3834.1	3657.5	3652.1
		K.LVEEAPAPFLTDAQR.K	3	206.7	205.9	179.4	179.4	182.6	166.9	171.9	172.7
		R.AKAPLVPGTPDPVKDADEVVAFAK.E	3	590.2	614.8	3576.6	3759.3	1071.5	1058.1	1329.4	1405.8
		R.AKAPLVPGTPDPVKDADEVVAFAK.E	4	338.6	323.1	356.4	344.4	595.1	598.6	536.2	626.2
		R.ALAEFTVEGLATVIPFHR.A	3	64.4	69.6	66.8	71.5	116.4	103.0	141.9	129.0
		R.AVVSDPAFIGDGEK.F	2	240.4	196.6	343.9	298.3	159.5	98.2	335.5	273.1
		R.AVVSDPAFIGDGEKFDVHTR.W	3	94.4	94.0	121.8	140.3	180.3	183.1	275.1	297.9
		R.AVVSDPAFIGDGEKFDVHTR.W	4	159.1	137.1	164.4	151.7	496.3	486.4	443.1	449.1
		R.HVEAQVIADQHGNVVVAGTR.D	2	29.9	17.6	22.3	20.6	38.3	47.3	25.5	45.2
		R.HVEAQVIADQHGNVVVAGTR.D	3	1098.7	889.2	867.1	739.3	1220.0	1480.3	1055.4	1312.8

		R.HVEAQVIADQHGNVVVAG TR.D	4	608.2	449.7	498.4	408.3	569.4	709.7	435.1	619.5
		R.LEVSLPGDLAIGGGGGAA APGVVR.K	2	238.2	255.4	242.9	261.6	110.2	111.0	141.7	156.2
		R.LQVEHPVTEETSGIDLVR. Q	2	1133.3	1128.3	1062.9	988.2	1119.1	1274.6	1090.2	1231.7
		R.LQVEHPVTEETSGIDLVR. Q	3	4950.5	5456.6	4591.5	5106.6	3448.0	4885.4	3958.6	5069.3
		R.M#DSGVETGSVIGGQFDS MLAK.L	2	104.3	115.2	95.5	104.2	21.2	43.5	13.1	16.2
		R.M*DSGVETGSVIGGQFDS MLAK.L	2	104.3	115.2	95.5	104.2	21.2	43.5	13.1	16.2
		R.MDSGVETGSVIGGQFDS MLAK.L	2	210.7	202.9	223.6	202.2	185.9	158.7	220.6	210.9
		R.QTVVVEVGGR.R	2	5.8	4.0	4.2	3.4	4.9	5.9	4.0	6.0
		R.RLEVSLPGDLAIGGGGGA AAPGVVR.K	2	135.1	187.0	113.7	195.3	86.1	142.3	118.6	187.1
		R.RLEVSLPGDLAIGGGGGA AAPGVVR.K	3	2073.3	2242.8	2254.5	2183.4	1670.0	1697.4	2279.0	2103.5
		R.TLEEIPELFESATR.E	2	1741.4	1892.0	2225.6	2333.6	1393.8	1291.4	2144.2	1946.6
		R.TLEEIPELFESATR.E	3	244.4	271.4	245.5	258.8	291.3	236.3	277.4	218.9
MSMEG _1808	MSMEG 1808	R.LYFSAPAEAPTTR.G	2	81.1	91.4	109.6	133.6	79.6	73.2	63.3	76.1
MSMEG _1809	MSMEG 1809	K.IDWHTDLNDPHVR.D	2	111.7	115.8	19.7	14.9	116.7	122.5	9.8	10.2
		K.IDWHTDLNDPHVR.D	3	130.0	121.2	89.0	69.9	83.7	90.1	57.7	56.3
		K.IDWHTDLNDPHVR.D	4	198.6	170.0	91.9	76.4	158.4	134.3	69.2	64.3
		R.AFKEDVLEILGK.Q	2	73.7	72.7	42.0	35.8	65.4	51.6	44.4	28.9
		R.AFKEDVLEILGK.Q	3	36.3	35.7	9.2	9.6	37.1	34.7	11.2	8.3
		R.DYINGEQFAALMDR.K	2	216.0	237.2	125.0	145.4	139.4	144.0	69.5	78.0
		R.LVTADWLASNLGR.P	2	501.0	521.1	533.1	544.9	371.3	341.2	351.4	381.0
		R.NYDGSWTEWGNAVR.V	2	188.8	225.7	138.9	146.8	124.6	134.3	71.5	95.2
		R.PGLAIVESDEDVLLYDTGH IPGAVK.I	3	1197.4	1069.0	1172.7	1033.4	820.1	707.4	911.3	819.5
		R.SSHTWFVLTHLLGLPGVR .N	3	110.0	102.3	55.6	53.8	55.2	55.3	27.5	24.8

		R.SSHTWFVLTHLLGLPGVR.N	4	36.3	32.1	15.0	14.8	17.3	15.6	5.3	4.2
		R.THMPDYPEEGALR.G	3	14.3	10.4	7.0	3.6	8.3	9.7	2.7	2.2
MSMEG_1812	MSMEG 1811	R.YSVFSWQR.V	2	133.5	124.8	97.6	96.3	116.4	136.7	78.9	85.4
MSMEG_1813	MSMEG 1812	K.LLYAYGEATVAK.V	2	544.9	568.7	643.3	704.8	504.0	498.3	545.0	607.9
		K.NRPLGDGVITGYGTIDGR.D	2	403.1	430.5	483.0	572.2	361.9	382.8	254.5	301.6
		K.NRPLGDGVITGYGTIDGR.D	3	613.0	594.9	630.8	581.9	587.9	553.6	562.5	471.6
		K.SGTAHYVASGEQDAFDYVR.D	2	398.6	488.7	287.1	342.9	318.8	384.6	213.4	283.5
		K.SGTAHYVASGEQDAFDYVR.D	3	1158.7	1190.5	1167.5	1148.8	1150.5	1248.1	1128.7	1083.7
		K.TVTGEDVTMEELGGAHTHMAK.S	3	149.3	174.3	199.3	213.4	78.4	106.9	88.1	124.6
		K.TVTGEDVTMEELGGAHTHMAK.S	4	272.5	204.1	291.0	245.1	128.4	145.9	114.1	141.3
		R.ILALLDEGSFVELDALAK.H	2	186.8	179.4	183.3	155.3	137.3	127.7	128.8	108.8
		R.IQEGVVSLGLYSR.I	2	1007.5	937.5	1605.3	1430.2	739.2	668.3	1354.5	1384.2
		R.LELQQTYEDTLVNPYIAAER.G	3	56.2	88.2	65.1	95.1	36.5	42.9	21.6	70.5
		R.RTEETLHPVGEAAVDK.V	3	1.0	0.5	0.7	0.5	0.2	0.1	0.1	0.1
		R.RTEETLHPVGEAAVDKVHAK.G	4	0.0	0.0	0.0	0.0	0.4	0.7	0.0	0.1
MSMEG_1819	MSMEG 1818	K.VAQGATVVTFDHEHVPTEHLDR.L	3	83.4	76.8	74.1	89.0	38.5	50.4	192.3	196.6
		K.VAQGATVVTFDHEHVPTEHLDR.L	5	123.8	82.7	58.1	42.1	71.3	68.5	57.0	48.1
MSMEG_1822	MSMEG 1821	R.SALLTEFLTR.L	2	29.8	30.2	38.8	39.1	25.0	22.6	30.2	35.1
MSMEG_1825	MSMEG 1824	K.LAGEQAVLAAKPDAYVVR.T	3	121.3	119.6	57.3	60.6	135.5	130.5	109.2	105.7

MSMEG _1828	MSMEG 1826	R.EVFPALLADGLR.I	2	101.1	92.7	70.8	74.9	74.5	62.8	51.9	62.2
		R.LDGAVIFDGVR.V	2	106.9	74.2	9.1	12.4	48.5	32.7	22.9	14.2
MSMEG _1832	MSMEG 1831	R.VSSLDVAVDEIPR.M	2	20.5	0.0	36.5	0.0	17.9	18.5	38.6	39.7
MSMEG _1834	MSMEG 1833	R.TTEEVDAVVDR.I	2	10.9	9.4	5.3	5.6	5.8	5.4	5.0	4.6
MSMEG _1836	MSMEG 1835	K.VLAADEPLSLQAHPSAAQ AQEGFER.E	3	139.3	142.2	105.8	131.8	105.6	97.9	90.7	94.0
MSMEG _1843	MSMEG 1842	K.AGVVPPAEDDDPAEWK.V	2	1453.1	1220.0	1474.3	1237.6	2171.6	2164.9	1667.2	1788.3
		K.GVTEETTTGVLR.L	2	5.2	5.1	5.6	4.7	8.8	10.6	19.3	20.7
		K.INIKPQVDQWIFDDGK.S	2	267.9	222.0	291.0	250.0	452.0	494.5	261.4	318.2
		K.INIKPQVDQWIFDDGK.S	3	111.3	115.6	86.5	91.3	175.2	184.5	91.1	108.7
		K.KINIKPQVDQWIFDDGK.S	3	571.7	640.3	458.4	557.6	659.9	738.1	437.8	594.1
		K.NQAILGNIGHFDNEIDMAA LER.S	3	405.1	363.9	341.9	324.0	470.2	407.4	274.1	252.7
		K.VADLSLADFGR.K	2	532.6	460.0	488.1	446.2	868.3	774.3	599.2	554.4
		K.VLIC#GYGDVGK.G	2	156.0	136.0	229.9	210.5	191.3	226.8	179.6	178.4
		R.LAEHEMPGLMALR.R	3	87.6	42.5	33.9	11.7	39.6	43.7	6.1	6.9
		R.VSVTEIDPINALQALMDGF DVR.T	2	102.7	133.4	94.7	127.6	79.9	118.0	53.7	86.1
		R.VSVTEIDPINALQALMDGF DVR.T	3	93.4	111.1	71.8	88.5	78.6	110.8	44.6	67.7
MSMEG 1843	MSMEG 1843	K.DQAEYIGVDVEGPYKPEH YR.Y	3	621.1	701.1	739.9	802.4	711.1	804.2	523.2	608.2
		K.DQAEYIGVDVEGPYKPEH YR.Y	4	270.2	267.4	243.7	242.0	398.0	370.3	225.3	252.9
		R.IHVEALGGTLTK.L	2	15.1	8.5	22.0	26.4	33.4	28.4	15.6	19.1
MSMEG _1873	MSMEG 1876	R.LHLPVPDWQVLLNVPTEL AAQR.A	3	65.8	68.7	117.0	119.7	42.4	48.7	65.4	89.7
MSMEG _1874	MSMEG 1877	R.AKVEKDPENPQVVLTVR. G	3	4.6	2.6	23.2	14.2	11.6	9.7	45.7	36.7
		R.DVLLEQVWGYR.H	2	36.1	36.3	70.6	74.6	33.5	41.3	66.3	80.7
		R.GEGFDTAVIGDGSQALTA VR.E	2	325.3	373.6	281.8	279.1	263.8	298.7	313.7	308.8

MSMEG_1878	MSMEG1881	K.TIYLFDVELDHER.N	2	36.4	39.3	84.8	86.4	20.8	28.3	54.4	48.0
		R.GEAC*ADSFYTAFESAVQK.L	2	0.5	0.4	134.3	136.7	1.9	2.6	111.3	104.9
		R.TM*VVEDDRESTAGTTPERPHAEVVVK.G	4	1.0	0.9	40.9	41.5	3.5	4.1	33.3	37.4
		R.TMVVEDDRESTAGTTPERPHAEVVVK.G	4	4.3	5.8	238.9	284.4	20.4	27.4	169.0	217.1
MSMEG_1881	MSMEG1882	K.AENQTLATITLQNYFR.L	2	7.9	7.9	22.5	21.3	9.0	7.0	19.9	19.6
		K.FLAVVDDVAER.H	2	142.9	131.4	110.7	114.3	179.8	151.3	129.7	118.9
		K.GIDNEAPPLTYTGPSEDGTAQVQR.S	3	124.5	124.9	122.8	111.2	92.9	109.2	77.0	100.2
		K.GVHVVTVNDYLAR.R	2	94.5	90.9	82.9	88.6	86.0	92.6	58.5	75.9
		K.GVHVVTVNDYLAR.R	3	102.7	87.5	81.9	66.4	108.3	101.6	75.5	77.0
		K.LSGMTGTAETEAAELHEIYK.L	3	205.6	200.3	734.4	903.6	178.3	206.4	639.4	282.2
		K.TLTAVLPSYLNALSGK.G	2	275.1	253.9	344.2	319.9	322.6	280.6	430.6	361.2
		K.VADYVNALSDDVEK.L	2	243.1	236.8	151.5	137.6	252.6	270.5	159.2	189.2
		R.DLIDSDAVGEPGELTR.E	2	214.5	213.7	194.8	200.9	227.0	229.5	164.3	162.1
		R.EGYDMFVGMLEALK.E	2	23.5	28.1	6.9	8.5	20.6	17.7	10.6	6.2
		R.GHNFAIVDEVDSILIDEAR.T	3	78.3	79.5	47.4	47.2	93.4	78.1	55.3	46.6
		R.GLDPVETPEEYEAAWHEVLPQVK.A	3	286.2	297.7	199.4	220.9	247.3	274.3	163.8	214.8
		R.KVADYVNALSDDVEK.L	2	34.1	32.6	32.7	49.8	27.5	25.0	46.4	64.4
		R.LNLPDDVPIEAK.M	2	23.0	25.0	39.4	45.6	19.3	23.8	20.8	34.6
		R.NGEVLIVDEFTGR.V	2	73.9	76.0	179.1	152.1	73.3	57.0	113.2	82.7
		R.NVLLNVIDR.K	2	62.9	52.7	35.4	22.9	51.8	40.3	25.4	18.4
		R.TPLIISGPADGASHWYQEFAR.I	3	206.3	216.3	240.0	225.2	233.4	229.6	248.6	231.2
		R.VADGEDLDDLLPEAFAVAR.E	2	112.1	141.1	89.9	112.1	115.3	149.6	80.7	108.9
MSMEG_1888	MSMEG1890	R.TPLAALLDAAGLEVPRPADGR.K	3	33.6	37.8	3.5	3.0	1.1	0.3	1.3	2.0
MSMEG_1890	MSMEG1892	R.SSVTFDGDEQAR.S	2	4.5	4.1	2.1	1.6	2.1	1.9	2.0	1.4

MSMEG _1903	MSMEG 1907	R.FGLSTVDLDTSTADGAR. R	2	68.5	47.4	9.4	5.5	26.9	8.4	20.9	4.1
		R.IGALTALTLLLR.R	2	5.8	5.6	0.0	0.0	1.7	0.5	0.1	0.0
MSMEG _1913	MSMEG 1917	K.GLAVAVVPVPTK.L	2	23.3	26.8	17.3	18.5	13.7	15.4	14.9	14.9
		R.STGYVVGGISPLGQR.K	2	41.3	42.9	59.9	68.8	24.7	28.1	67.9	77.5
MSMEG _1914	MSMEG 1918	K.EIAEIMETPIGTVMSR.L	2	82.5	90.2	44.5	52.9	88.6	97.0	44.0	57.5
		R.DAIPLLDQLYGGALR.M	2	54.3	59.7	42.6	52.7	20.5	64.2	21.0	43.4
		R.MAVYYADVEGFPYK.E	2	32.5	39.8	29.2	30.2	35.7	35.5	25.2	25.2
		R.SAEVEALEALPDTEIK.A	2	181.0	206.9	226.2	229.3	178.6	169.7	227.4	211.9
MSMEG _1926	MSMEG 1929	K.LAAEPLAELQLR.D	2	40.8	32.4	65.7	68.7	37.5	22.7	65.8	71.2
MSMEG _1927	MSMEG 1930	K.LLGALPTLYDSR.T	2	29.6	34.0	93.3	98.5	27.7	27.3	110.3	122.4
MSMEG _1934	MSMEG 1938	K.IAYVEIGAADVR.R	2	168.1	190.8	254.9	261.5	138.3	153.1	250.7	246.8
MSMEG _1935	MSMEG 1939	R.LIFENDYVTEPQVAAQVK. V	2	100.5	117.8	116.3	128.4	74.2	97.9	62.9	97.4
MSMEG _1940	MSMEG 1944	R.PVVLPHNPIPTVLVR.A	3	32.7	32.2	67.1	62.2	33.9	27.3	67.2	75.4
MSMEG _1951	MSMEG 1955	K.IVEADQIEEWAEAEK.A	2	6.6	9.6	76.3	87.7	17.5	21.5	75.9	83.0
		R.LPLQLIEDQVMTR.L	2	2.5	3.1	65.6	64.0	13.9	16.3	60.9	68.4
MSMEG _1954	MSMEG 1958	K.AAQQLFTVLGELK.G	2	28.3	30.6	74.1	50.0	13.9	24.0	57.2	67.6
MSMEG _1959	MSMEG 1961	K.AEALQDLESALTAAQEAQ R.S	3	43.4	45.1	13.2	14.8	86.3	50.6	14.9	19.7
		K.NFYYFPDQLSIDR.Y	2	24.4	25.4	20.4	25.9	57.9	52.5	28.8	25.1
		R.IYFGPVISNTPADYAIVGK. T	2	68.2	61.0	264.0	229.1	111.3	91.0	229.2	231.3
		R.LLDPTIVSPAFTQFQQGK. N	2	283.5	279.9	483.6	465.2	214.1	236.5	366.7	417.4
		R.NFLFSNVIGENSK.I	2	47.4	57.7	25.0	40.9	85.4	74.7	22.6	32.0
		R.PAQAPPPPSAATPTGPVQ ISQAK.A	3	51.6	29.5	13.3	25.6	33.7	33.6	6.1	18.1

		R.TRPVFVPTAGPNDPVAR.Y	3	18.3	15.8	87.7	89.6	41.4	40.2	67.5	61.7
MSMEG_1979	MSMEG 1982	R.SLDDPNEYVLVEAFR.D	2	17.6	35.0	161.0	320.5	10.4	16.2	94.6	138.8
MSMEG_1981	MSMEG 1984	R.EVVLEVVEHDPQTGAYTVASGWGPK.A	3	62.3	73.6	122.8	115.5	72.8	80.3	129.1	102.9
		R.TIAVTALPLDQAEGADIFAR.Y	2	82.2	98.0	99.0	106.9	81.9	87.8	72.6	70.9
		R.TIAVTALPLDQAEGADIFAR.Y	3	44.8	50.3	29.4	25.6	32.0	43.9	21.3	23.8
MSMEG_1996	MSMEG 1997	K.IGISTFVNDDTIDAVTLAR.A	2	76.0	80.0	74.1	77.1	57.1	56.8	87.5	80.4
		K.PVQKPHPPIYIGGASEATVK.R	3	47.1	47.2	46.1	44.9	36.4	30.5	54.0	46.6
		K.PVQKPHPPIYIGGASEATVK.R	4	43.5	47.4	36.3	34.3	24.6	27.5	40.5	41.6
		R.GFHSLVIAEHSHIPASR.E	3	31.9	35.3	25.4	27.9	15.4	23.7	31.7	31.1
		R.GFHSLVIAEHSHIPASR.E	4	87.4	98.1	47.0	49.6	47.4	59.0	48.7	56.3
		R.HDAGWISNPLPVDFLSK.F	2	45.3	41.4	55.2	49.6	23.3	33.4	49.5	51.1
		R.LLDEQIEAIK.A	2	75.3	56.7	73.0	47.6	45.2	48.3	70.9	77.0
		R.PKPVQKPHPPIYIGGASEATVK.R	4	54.8	60.8	54.0	56.8	23.1	27.0	39.3	46.7
		R.TLDPFVTLAAAAAVTTK.I	2	82.1	83.0	79.0	94.8	22.6	40.4	31.8	53.8
MSMEG_2010	MSMEG 2010	R.EGNADYAVIALLQNPEDVDTYVDHPAHVELSR.Q	4	61.8	72.1	79.7	95.2	74.4	76.0	123.0	112.0
		R.SAVQFALPENAVLDL	2	46.0	34.7	63.3	52.3	63.6	54.2	83.2	72.0
MSMEG_2019	MSMEG 2018	R.ALFADAVATFGR.V	2	10.9	9.8	19.8	25.7	3.1	6.2	14.2	17.1
MSMEG_2025	MSMEG 2027	R.ILSAPLEPLTEGTVTSAEDLAALIAK.A	3	19.1	20.4	28.2	28.7	16.1	20.2	19.8	23.0
MSMEG_2026	MSMEG 2028	K.AVESPEPPAFLLLGSDALSAYR.R	2	264.8	271.3	158.5	152.8	178.1	180.1	160.2	173.5
		K.AVESPEPPAFLLLGSDALSAYR.R	3	134.3	132.0	56.0	57.4	94.1	90.8	51.7	57.7

		R.DVSSVADIAETAPDR.V	2	343.5	331.8	195.5	191.5	252.5	256.5	204.8	208.7
		R.FGGVDVLVNNAGYGYR.A	2	326.2	333.5	96.5	103.6	320.3	294.5	106.2	117.6
		R.TLAEAVAAAGHNLVVTAR.D	2	175.4	174.5	110.9	104.1	111.6	111.7	99.6	83.1
		R.TLFDTHIFGTVATIK.A	2	63.7	68.9	127.6	105.1	43.9	42.6	163.8	184.4
		R.TLFDTHIFGTVATIK.A	3	65.3	62.9	20.1	20.9	32.3	32.3	12.5	17.8
		R.VVAAPLDVTRPEQVTAAAQLADER.F	3	896.0	921.2	468.9	456.2	636.5	634.8	499.1	490.1
MSMEG_2027	MSMEG2029	K.LAVEAYPPYAEYQTK.T	2	55.2	64.7	48.6	63.7	24.8	26.1	43.6	43.9
		R.ILEQGTTDGVHVLDRPIVLFTTTGAK.S	4	46.6	43.3	65.4	63.3	22.4	26.9	76.3	70.9
MSMEG_2033	MSMEG2034	R.GLYQYKPDLPFSPGAEVAGVVR.S	3	248.9	257.0	411.2	457.1	217.6	204.5	380.1	359.3
		R.LLVVGFTGGEIPQVK.V	2	148.4	145.0	193.9	232.7	103.8	96.6	96.9	115.8
		R.LSGGETVLVHGAAGGIGSSVLR.L	3	79.2	70.2	46.5	36.5	62.1	53.6	15.8	16.1
MSMEG_2045	MSMEG2046	R.GPVLVGSATEVADELER.W	2	57.4	50.8	8.2	9.8	72.2	60.1	28.5	10.2
		R.VAWNIVTSYLDSAAR.N	2	92.8	67.0	133.2	93.4	59.9	53.6	411.9	488.2
MSMEG_2078	MSMEG2080	K.ANDMWGPTEDPNSAWK.R	2	68.0	81.8	129.2	142.6	158.7	182.9	135.8	142.9
		K.VQFQSGGPGSHAVYLLDGLR.A	3	325.1	337.1	611.6	613.2	837.7	777.2	741.0	689.0
		K.WETFLTSELPEWLAANR.D	2	112.0	112.5	153.1	160.8	195.6	213.4	151.8	149.1
		R.ELQAMVPDLQR.V	2	93.3	101.6	232.9	225.2	323.3	260.9	293.7	278.4
MSMEG_2079	MSMEG2081	R.LPADQIVALGALTPR.E	2	206.3	214.9	116.3	110.2	101.6	125.3	74.6	83.3
MSMEG_2080	MSMEG2082	K.ILDIFEGTQQIQQLVVAR.R	2	49.1	40.6	150.5	150.3	31.9	32.2	106.6	136.5
MSMEG_2081	MSMEG2083	K.AAELGITAVNVPEDFEGIAEHR.S	3	161.6	151.7	309.9	304.6	159.7	165.9	428.6	382.8
		K.ASGADLFDLTPDDDQK.M	2	53.4	50.3	78.6	105.6	27.4	41.0	86.7	103.3

		R.LGEDGATDQDYSEAIALAR.L	2	72.7	88.4	124.4	147.8	76.2	56.5	160.3	159.5
		R.TATDIGLALITPIVGQEWLDR.Y	3	55.3	65.2	98.3	115.4	53.2	58.8	139.0	139.2
MSMEG_2089	MSMEG 2090	R.LLLGADTPTSGDVR.V	2	48.4	32.4	29.8	25.8	31.7	43.8	19.7	39.7
MSMEG_2090	MSMEG 2091	R.FGFLINEVLTGLR.R	2	12.3	7.7	4.7	2.1	11.3	10.3	2.6	2.9
MSMEG_2092	MSMEG 2093	R.EEPWNRPLFAAPHR.L	3	82.1	51.2	72.6	57.9	72.6	68.8	86.8	83.8
		R.LMIDGVPVGR.L	2	15.3	15.5	77.1	87.1	29.4	33.8	101.6	102.1
MSMEG_2111	MSMEG 2114	K.LDPAAAPSSAPDLSESR.Q	2	30.4	23.4	31.5	24.9	25.9	30.2	26.4	30.9
MSMEG_2112	MSMEG 2115	R.AAHVLHLAQEHGEIGTEAQR.E	3	8.4	8.7	16.1	10.4	3.2	5.0	18.3	15.5
		R.AAHVLHLAQEHGEIGTEAQR.E	4	8.1	5.7	36.9	27.9	4.6	4.6	35.4	38.0
MSMEG_2116	MSMEG 2119	K.SLMLPIAVLPAAGILLR.I	2	1.9	2.3	16.8	18.4	9.6	10.7	27.5	28.3
		R.IPGFAQLQR.L	2	0.0	0.0	0.6	0.4	0.0	1.2	0.9	1.0
MSMEG_2117	MSMEG 2120	K.GLNPIVPVVIMDER.E	2	51.3	54.9	72.0	74.1	22.2	23.8	39.8	54.1
		R.TGADIATGAELFTAAK	2	91.9	73.2	138.3	131.6	72.0	65.5	152.0	124.5
MSMEG_2118	MSMEG 2121	R.IGSLAADAITALITR.K	2	73.6	72.9	84.8	88.4	59.3	49.5	85.1	84.7
MSMEG_2136	MSMEG 2139	K.YNPPNGGPADSDATSVIAK.R	2	14.8	10.6	31.9	17.7	13.0	23.6	22.2	34.6
		R.IGADPLGGASVDYWGAIAER.H	2	37.5	39.6	71.1	75.0	21.7	29.7	123.3	123.8
		R.YAELAEQYGAPSYAR.I	2	119.9	85.7	157.0	149.4	122.2	90.3	181.7	162.9
MSMEG_2155	MSMEG 2157	R.HEIVDTLDYLAR.L	3	1.6	2.7	34.2	24.8	3.7	3.9	34.8	29.5
MSMEG 2968	MSMEG 2968	R.AAGLQSYEIPR.D	2	19.3	12.6	16.5	17.7	0.3	0.4	2.3	6.5
		R.TVLLTGATGFLGR.Y	2	100.8	87.0	0.8	0.6	1.0	1.3	0.6	0.9
		R.YLALEWLER.M	2	29.6	27.1	0.3	0.1	0.0	0.0	0.2	0.0

MSMEG _2171	MSMEG 2175	K.SPLGRDALTELLRR.A	3	0.2	0.3	5.0	5.0	0.1	0.2	0.9	1.5
MSMEG _2174	MSMEG 2178	R.PFYVATAANPENMR.R	2	118.0	102.0	4.7	10.0	101.4	106.1	3.9	10.3
		R.SFTIVGDLAQR.R	2	29.4	23.5	42.2	38.8	18.7	13.6	37.7	28.0
		R.VAYLLYTYR.K	2	68.4	61.2	12.0	8.1	18.3	19.6	26.7	17.9
MSMEG _2198	MSMEG 2203	K.LADWGSGSALGDDTYER.V	2	16.9	18.9	130.4	127.6	19.2	30.1	90.7	137.2
		R.FDLDPGRPILHGLVGDIDAAR.L	3	32.3	32.8	111.5	104.9	42.4	45.9	118.1	123.3
		R.FDLDPGRPILHGLVGDIDAAR.L	4	18.3	17.4	55.9	58.5	23.7	23.4	68.4	59.5
		R.GSAVALGAAAFNAR.V	2	62.9	51.7	56.9	39.5	21.0	18.3	60.5	34.9
		R.LLTQYTSAMDVGYR.G	2	234.4	246.9	176.2	206.7	166.9	173.6	208.9	265.1
		R.LTDFDEIELPNLNR.V	2	139.9	162.9	308.4	327.8	173.3	178.6	347.8	316.8
		R.VPGTVFDLGVNK.A	2	14.5	12.1	175.2	163.4	49.1	36.1	237.5	168.4
MSMEG _2201	MSMEG 2206	K.TGPFGGTIAHGLLTLSLLPHFTHQLYR.V	4	61.8	61.6	80.1	73.6	82.8	88.0	94.4	87.0
		K.TGPFGGTIAHGLLTLSLLPHFTHQLYR.V	5	25.9	12.7	39.5	20.1	33.1	35.3	41.5	43.2
		K.VFDNLDALAAAAGTELGPTDWLEVTQDR.V	3	175.7	234.6	235.0	333.0	142.2	252.4	153.9	282.6
		R.VDNITM#AINYGYNK.V	2	81.1	96.4	100.2	113.5	72.1	73.1	79.2	69.0
		R.VDNITM*AINYGYNK.V	2	81.1	96.4	100.2	113.5	72.1	73.1	79.1	69.0
		R.VDNITMAINYGYNK.V	2	197.7	213.6	343.8	386.9	178.9	167.1	297.4	269.1
		R.VNLFADATEDHQWIHVDPEK.A	3	358.1	437.3	554.6	661.5	405.7	426.6	565.9	542.7
		R.VNLFADATEDHQWIHVDPEK.A	4	336.3	267.2	509.9	379.8	417.7	434.2	540.1	532.9
MSMEG _2225	MSMEG 2230	K.AFMGNPILC*DLILIR.S	2	14.8	15.1	38.3	38.9	17.0	16.7	38.6	37.4
		R.EFVVATPYVEAK.D	2	29.2	25.7	66.3	66.3	32.9	45.0	47.2	68.6
MSMEG _2263	MSMEG 2266	R.YAVPGLGNVPLETGPLAR.R	2	76.7	86.5	62.2	69.4	249.3	239.3	106.7	99.9
MSMEG _2271	MSMEG 2274	R.EVNPTTEILPVSAR.T	2	27.0	35.3	109.9	152.2	61.5	66.6	64.8	58.7

MSMEG _2280	MSMEG 2283	R.SDELLDLITTNVAR.R	2	14.8	16.4	68.5	66.9	50.1	35.9	102.3	75.6
		R.TPIRPEYLAGLIDR.L	2	26.6	35.4	93.9	104.6	38.7	46.9	48.0	63.8
		R.TPIRPEYLAGLIDR.L	3	96.0	77.7	120.3	115.6	125.6	75.1	99.7	108.0
		R.VPVLAIAAHIPR.S	3	2.8	1.0	43.6	29.6	3.9	3.9	10.2	8.3
		R.VYGLPGDSLNGFTDAIR.R	2	5.6	7.2	89.6	80.0	9.8	8.2	37.2	37.7
MSMEG _2351	MSMEG 2350	K.DDGLHGSDLIQTAWTLAR .A	2	201.5	195.3	137.8	140.5	234.0	229.4	188.6	176.2
		K.DDGLHGSDLIQTAWTLAR .A	3	386.1	388.3	266.2	254.1	463.9	422.1	348.9	335.4
		K.EVTVLSLAEIGVEPDEVGV ANAGSK.V	2	357.2	370.0	237.5	250.8	307.4	305.9	246.0	240.9
		K.EVTVLSLAEIGVEPDEVGV ANAGSK.V	3	636.1	690.2	453.7	481.2	514.1	548.7	422.1	462.9
		K.IAEYLVAQK.L	2	4.5	4.1	0.9	1.2	2.7	3.4	0.1	1.2
		R.AVEEALLIK.E	2	17.4	17.5	4.6	4.6	23.5	19.7	11.3	7.2
		R.EAADAVLDEINER.A	2	1954.5	2190.4	1621.9	1867.6	2206.1	2259.3	2035.6	2163.3
		R.EAADAVLDEINER.A	3	66.3	42.6	37.9	18.4	72.2	60.5	42.9	42.9
		R.EGGDSTVTVLTAGPER.A	2	202.0	168.0	90.7	67.6	212.1	203.0	99.8	90.7
		R.ETDEGVFTLEASLPAVVS VNEK.I	2	292.8	293.4	253.6	228.2	294.2	271.7	265.1	232.3
MSMEG _2352	MSMEG 2351	K.IVEELADSLGGAVGASR.A	2	1442.7	1403.3	1386.0	1443.2	1169.2	1120.6	1123.4	1539.1
		K.IVEELADSLGGAVGASR.A	3	481.4	487.0	244.5	243.1	448.2	397.0	172.0	162.3
		K.REPAVAGDRPELTEASVV VAGGR.G	3	583.9	571.1	2682.6	1906.7	536.9	513.7	873.8	845.4
		K.REPAVAGDRPELTEASVV VAGGR.G	4	450.7	466.1	198.3	218.3	390.5	390.3	121.2	115.9
		K.TIVAVNKDEEAPIFEIADLG IVGDLFK.V	3	973.4	1087.7	834.3	913.9	429.2	675.3	418.4	606.7
		K.VSAELLTAAR.A	2	37.3	32.5	29.9	24.8	34.0	24.9	13.2	16.2
		R.AAVDSGYYPGQFQVGQT GK.T	2	1873.0	1838.9	1723.5	1736.0	1577.0	1597.9	1279.4	1320.6

		R.AAVDSGYYPGQFQVGQTGK.T	3	466.6	513.0	198.4	241.2	310.6	347.8	76.1	114.0
		R.ALGEPSAVVVGKPGTAAGLTDGLK.A	2	144.9	141.5	58.3	50.2	181.7	153.0	66.1	48.4
		R.ALGEPSAVVVGKPGTAAGLTDGLK.A	3	2692.3	2957.4	1993.6	2094.7	3088.5	3321.4	2174.6	2254.7
		R.EPAVAGDRPELTEASVVVAGGR.G	2	153.3	96.0	75.1	47.8	112.5	105.6	36.3	30.7
		R.EPAVAGDRPELTEASVVVAGGR.G	3	2033.7	2369.2	2025.9	2426.6	1677.0	1618.5	2114.2	1857.2
		R.IGSGLLVDVIEVK.E	2	1041.6	1051.0	903.6	860.9	925.4	917.7	729.5	740.0
		R.PGSVEAVPADGAGEVVNVEVPAQAENATR.I	3	249.4	291.7	122.8	146.2	104.4	115.7	26.5	29.6
MSMEG_2362	MSMEG2361	K.ATGEVEAELPEHPDADER.R	3	22.3	14.4	17.1	10.7	18.6	12.4	11.9	9.6
		K.IDGVALSLVYR.D	2	11.7	8.2	8.3	7.1	18.5	13.5	3.4	15.3
		K.VAGSTVGLATLHNASEVK.R	2	21.5	20.7	26.5	26.4	26.8	19.1	33.3	37.0
		K.VAGSTVGLATLHNASEVK.R	3	101.1	94.4	96.3	103.8	80.4	74.5	76.7	77.7
		R.EFEFPTHC#PEC#GTELAPAK.E	3	78.4	77.3	84.5	109.5	50.6	50.5	63.7	79.0
		R.LTPSDEFPVPAVLEVR.G	2	201.5	193.0	162.3	152.5	214.7	221.9	165.2	156.6
		R.M#LSLDNVFDSDELTAWAAR.I	2	42.0	35.8	50.1	48.2	62.5	43.8	49.9	57.3
		R.TPDSPTQLVGGAGFATEFAPAEHLER.M	3	122.2	117.5	101.7	107.4	114.7	103.2	126.2	108.3
		R.VADFEELNAGLVAEGKPPFANPR.N	3	167.9	163.4	167.2	161.4	280.8	208.3	195.4	177.3
MSMEG_2365	MSMEG2364	K.EVSSTEVTQAHLDQIAATDDR.F	3	387.5	409.0	260.9	327.9	253.7	265.9	225.1	274.9
		R.TVLDTALLHAVIAGHDAR.D	3	71.8	53.8	220.6	215.5	65.1	68.3	554.5	509.3
		R.VGDDGTHSAEEVMALTR.A	2	60.1	56.4	14.5	18.5	19.0	19.8	0.0	1.3

		R.VGDDGTHSAEEVMALTR.A	3	77.7	74.6	31.1	32.3	37.7	20.0	18.4	19.4
MSMEG_2366	MSMEG 2365	R.IGVLTGGGDC*PGLNAVIR.A	2	62.0	66.7	466.1	504.4	36.5	38.8	335.6	387.0
MSMEG_2367	MSMEG 2366	K.VQAAGAIVGAVMK.A	2	44.6	36.6	47.6	47.5	38.3	23.9	53.2	63.8
		R.AWWGNFLVQK.A	2	65.2	63.7	14.9	20.7	54.6	71.8	21.5	15.8
		R.DLVNAGAVELVAATVDHGVSSEAAR.A	3	346.1	314.4	187.9	158.5	353.7	292.1	281.8	236.9
		R.IGLALNC#EITPWGR.F	2	99.1	94.7	79.9	79.3	101.3	98.8	120.2	73.9
MSMEG_2372	MSMEG 2371	K.AVIAELVQSLR.K	2	85.9	80.7	36.9	29.1	97.0	77.4	40.8	30.4
		K.LAEALGC#VGLR.C	2	18.7	12.9	11.0	8.7	16.7	9.1	27.4	28.1
MSMEG_2373	MSMEG 2372	K.VVDVSTESLTIEATGTPEK.L	2	62.3	62.5	4.0	5.4	44.3	43.2	0.5	1.0
		R.EIAQSGVVSVSR.G	2	6.8	5.5	1.5	1.1	7.6	4.6	1.4	0.9
		R.GQIIEAVNLFR.A	2	66.9	65.3	6.8	9.2	74.7	69.1	6.3	6.7
		R.M*TIVVSVEDSPLEQITK.Q	2	21.4	0.0	17.5	0.0	12.8	13.5	5.1	6.3
		R.RGFNIQSLAVGATEQK.D	3	32.9	37.4	4.2	11.7	33.6	40.1	5.7	7.1
MSMEG_2374	MSMEG 2373	K.AEEQGLEVDTPAEVAK.W	2	45.7	43.8	14.7	10.8	27.2	35.2	5.2	6.1
		K.APENVTVGMVAPK.G	2	31.7	33.3	8.4	12.9	7.2	9.6	3.9	6.5
		K.GEGQALALSYAAAIGGAR.A	2	1351.7	1205.3	1063.2	989.7	1275.3	1112.4	1164.2	1010.8
		K.GEGQALALSYAAAIGGAR.A	3	148.5	140.4	76.6	86.3	149.2	131.0	96.9	82.0
		K.GVPC#LIAIDQDPK.G	2	630.0	495.8	663.3	519.6	648.7	681.0	755.5	861.1
		K.GVPC*LIAIDQDPK.G	2	629.5	491.6	662.9	518.2	637.3	679.8	752.2	860.3
		K.LIVDLIYEGGIAR.M	2	265.8	210.0	229.3	187.8	276.7	273.1	240.0	258.1
		K.VAVIGYGSQGHAHSLSLR.D	4	371.8	238.9	106.2	60.0	413.9	333.4	186.9	142.5
		R.DLMSWVDRPITETA	2	333.5	282.4	577.5	521.6	342.6	268.3	416.2	329.5
		R.EKAEEQGLEVDTPAEVAK.W	2	24.8	14.6	11.8	6.2	25.5	24.7	9.4	11.4

		R.EKAEEQGLEVDTPAEVAK.W	3	138.6	103.3	102.7	78.6	121.9	145.0	81.5	113.7
		R.KVAVIGYGSQGHAHSLSLR.D	3	10.3	6.0	44.1	38.2	73.4	53.2	83.0	87.2
		R.KVAVIGYGSQGHAHSLSLR.D	4	72.1	48.0	39.5	24.0	302.2	240.3	196.8	175.4
		R.LVANVEGGNK.E	2	1.7	1.1	0.6	0.4	0.8	0.6	0.2	0.1
		R.LVANVEGGNKELEALR.K	2	27.0	16.8	12.4	14.3	135.7	131.3	80.1	88.1
		R.LVANVEGGNKELEALR.K	3	129.4	84.6	81.6	64.2	448.2	427.4	298.9	306.8
MSMEG_2375	MSMEG 2374	K.FADGNPYGVGLIANR.T	2	91.3	94.0	107.3	105.9	70.4	231.7	108.8	126.3
MSMEG_2378	MSMEG 2377	K.LAESTVAALGDEVEVR.W	2	255.1	237.1	277.3	257.2	253.9	251.4	280.2	288.3
		R.AAQLGIELLPLDDLLAR.A	2	629.6	597.3	879.3	881.3	616.7	554.5	941.5	901.3
		R.AEGHNLIVNYSDKPGALGK.I	3	28.2	18.6	72.7	48.6	19.0	9.7	36.3	23.1
		R.AEGHNLIVNYSDKPGALGK.I	4	66.8	49.4	112.1	85.6	46.6	48.5	71.1	70.3
		R.AGVGLDNVDVDAATAR.G	2	447.1	362.4	710.2	526.1	587.8	604.2	981.7	914.4
		R.GGLIDEQALADAITSGHVR.G	2	244.0	214.2	173.1	128.9	206.2	206.0	219.4	158.1
		R.GGLIDEQALADAITSGHVR.G	3	346.7	316.4	375.5	344.5	368.3	320.5	480.1	410.1
		R.GVTAEISTATESPNHR.S	2	8.1	4.0	39.3	30.7	7.4	5.5	35.3	35.5
		R.GVTAEISTATESPNHR.S	3	32.6	24.5	35.5	28.6	28.0	26.3	34.8	28.4
		R.SATTVDAEVIAAAPK.L	2	50.8	47.5	170.0	153.7	30.9	24.4	109.5	92.3
MSMEG_2379	MSMEG 2378	K.TNVLAFAGSLWSR.T	2	219.2	183.5	121.6	108.4	189.9	158.0	153.6	129.0
		R.GYDAILLGAIGDPSVPSGVLER.G	2	301.0	298.5	237.3	238.8	165.7	164.1	202.4	225.3
		R.LYPGVTGPLVGNPPIDFVVVR.E	2	547.4	569.3	476.8	506.2	297.4	296.7	381.1	376.0

		R.LYPGVTGPLVGNPPIDFVVVR.E	3	273.3	314.9	205.6	220.1	180.0	152.3	214.2	193.5
MSMEG_2382	MSMEG 2381	K.EIAEHPFGNPNFTGR.S	2	36.6	25.4	38.9	26.8	63.2	54.3	32.3	42.1
		K.EIAEHPFGNPNFTGR.S	3	91.2	69.7	78.6	57.5	81.9	80.7	73.2	94.0
		K.GHDTFC#PLGPWIVTDLDPSDVELR.T	3	109.0	135.1	124.2	145.9	85.2	106.1	104.6	138.9
		K.GHDTFC*PLGPWIVTDLDPSDVELR.T	3	107.5	133.3	124.2	144.6	85.0	104.1	104.5	138.8
MSMEG_2383	MSMEG 2382	K.TALLEGLELKPR.K	3	24.4	6.9	3.6	3.3	43.9	28.9	16.2	14.7
		R.GPTTFAAGSVPDFAITR.S	2	410.2	411.7	564.2	573.0	467.2	420.6	768.0	710.3
		R.IGVAEFVPEFAHLPSVLGEGNK.K	3	72.6	76.2	27.2	32.0	45.1	43.7	24.1	25.1
		R.TALFNWAYAR.H	2	58.5	43.9	75.9	59.7	54.1	51.9	109.2	117.3
		R.VVVLGDAWGLLK.F	2	65.0	58.8	26.8	20.6	65.8	59.1	37.2	26.3
MSMEG_2387	MSMEG 2386	K.TMAVNVDGELPPGVSAK.D	2	98.0	132.2	108.9	129.7	55.9	50.8	84.0	89.2
MSMEG_2388	MSMEG 2387	K.AGLLAAEVAQDDVELLWK.V	2	96.1	97.0	125.2	113.1	129.3	119.9	107.5	100.2
		K.GSVLVAGPDFGTGSSR.E	2	186.9	162.5	130.9	104.4	157.0	189.3	68.8	94.6
		K.VIEQNPGLEITVNLQDR.T	2	119.1	123.2	73.8	63.2	83.6	95.2	60.2	58.3
		R.LLEGLDDIGLTLR.K	2	126.2	122.3	134.1	129.2	143.6	135.6	92.5	92.4
		R.NDPSFILNLSPFDK.G	2	294.7	284.2	181.1	169.6	333.3	298.9	176.3	164.9
		R.SNVDTDQIIPAVYLK.R	2	2046.0	2312.8	2015.4	2188.6	1638.9	1740.7	2058.1	2229.9
		R.TGFEDGLFAAWR.N	2	66.9	62.2	46.4	50.0	70.6	73.8	29.1	38.8
		R.TVTAGTVVVPFR.I	2	80.8	69.5	123.1	111.8	122.6	122.6	119.2	106.2
MSMEG_2389	MSMEG 2388	-.MNKAELIDVLTTK.M	2	67.0	79.7	75.9	66.7	251.7	266.2	290.0	268.0
		-.MNKAELIDVLTTK.M	3	9.9	10.3	10.6	13.6	167.9	145.1	181.9	173.4
		K.AELIDVLTTK.M	2	1259.0	1237.4	1986.6	1874.3	1782.9	1571.3	2214.9	1988.6
		K.GDSVTITGFGVFEQR.R	2	400.8	384.4	569.6	531.3	533.2	501.0	600.8	572.9
		K.LPADGPAVK.R	2	0.5	0.5	0.6	0.4	1.3	1.1	0.7	0.8
		K.VKPTSVPAFRPGAQFK.A	2	196.0	132.3	322.9	209.4	429.6	318.0	547.8	396.4

		R.AVHKGDSVTITGFGVFEQR.R	3	11.1	16.2	10.7	14.7	140.1	137.3	192.2	191.4
		R.QATAAVENVVDTIVR.A	2	641.1	654.0	1167.9	1131.3	955.9	831.3	1411.4	1239.9
		R.QATAAVENVVDTIVR.A	3	61.0	60.5	82.6	76.1	89.3	83.5	90.4	84.8
		R.RQATAAVENVVDTIVR.A	2	45.3	41.2	56.0	46.7	133.0	130.9	111.5	107.3
		R.RQATAAVENVVDTIVR.A	3	33.4	30.6	38.3	36.8	104.1	84.4	86.2	82.2
MSMEG_2391	MSMEG 2390	K.ANALVDEQVIDALYR.A	2	250.1	253.7	346.6	328.0	269.7	227.2	429.1	366.1
		K.DRPFVPATPPAFGER.E	2	61.4	64.2	61.0	76.4	44.7	60.9	76.3	95.1
		R.DGDVLVHHPYDSFSTTVQR.F	3	104.1	98.9	122.9	130.7	60.9	82.7	124.6	155.5
		R.FIEQAAADPNVLAIK.Q	2	230.4	225.2	432.0	423.0	164.8	189.5	381.9	389.6
		R.GIC*ALRPGVPGYSDNITVR.S	3	246.0	277.0	351.3	355.7	171.6	219.4	246.9	334.9
		R.HDSHWVALPQEGQTVR.D	3	51.7	36.3	74.5	58.9	21.5	22.1	44.6	46.7
		R.HDSHWVALPQEGQTVR.D	4	38.6	28.3	32.2	21.3	21.4	18.2	22.6	21.8
		R.LEVSDDMTESMLELLLR.E	2	43.3	46.8	56.1	64.9	29.0	38.1	54.2	69.0
		R.NADFEVEEDRDEDLLQALER.E	3	175.5	169.6	182.1	180.3	131.0	110.8	198.7	167.0
		R.TLEQAGVHVVYGLIGLK.T	2	39.0	35.2	256.7	294.2	36.8	27.2	217.3	302.3
		R.TLEQAGVHVVYGLIGLK.T	3	61.1	63.6	85.1	95.9	45.3	52.7	85.5	112.1
		R.TSGDSPIVNALIDAAEAGK.Q	2	185.3	224.9	187.4	202.4	147.2	188.2	216.3	226.4
		R.VLALAADPSLPLLER.A	2	240.3	217.6	378.7	353.0	231.0	148.4	535.6	351.5
MSMEG_2397	MSMEG 2396	R.ADVVPSVQQVAGITR.T	2	28.9	28.5	31.5	38.9	11.9	12.9	39.3	29.4
MSMEG_2399	MSMEG 2398	R.ALEPVADQVAQMGEFLR.T	2	88.7	78.1	58.9	49.9	79.1	77.1	54.0	49.8
MSMEG_2407	MSMEG 2405	K.LATPDQGFTSSQAAAR.A	2	8.7	7.8	7.2	6.5	9.7	11.2	8.3	9.2
		K.YVDSWGGLMK.V	2	62.5	59.0	24.4	20.1	54.2	63.7	19.3	19.5

		R.EVNAGYNIVTEAYGPLGVGR.L	2	119.6	127.2	76.8	71.8	125.4	123.3	89.0	78.1
		R.LGLDYVDLYLIHWPGGDTSK.Y	3	98.4	103.1	71.4	75.0	83.0	89.4	60.0	63.4
		R.LIDTAAAYGNEAAVGR.A	2	295.6	250.0	279.7	230.4	293.8	332.3	252.9	294.5
		R.LLDHPAVTAIAEAHGR.T	3	102.9	85.2	89.9	79.1	112.4	117.7	85.3	95.4
		R.LLDHPAVTAIAEAHGR.T	4	163.9	142.4	97.6	75.4	208.7	201.8	126.9	110.9
		R.SVSAALEAGYR.L	2	24.8	20.6	16.9	14.9	27.4	20.7	12.3	8.4
MSMEG_2408	MSMEG 2406	K.LWNSDQGYDATLAAFDASVQR.L	3	195.6	193.3	70.9	66.1	106.8	121.6	87.1	116.9
		R.AVAAALQAGYR.H	2	67.6	52.2	31.6	24.7	51.2	35.5	52.8	32.7
		R.ITLNDGNSIPQVGLGVWQTPAEDTER.A	2	66.8	74.3	10.4	14.1	21.4	22.2	11.0	12.1
		R.ITLNDGNSIPQVGLGVWQTPAEDTER.A	3	476.1	543.9	192.1	224.5	201.9	268.4	173.9	216.9
		R.LGVDYLDLYLIHWPVPENNK.F	3	587.0	627.9	296.5	297.2	209.1	238.8	206.1	248.1
		R.WHIQLGNIVIPK.S	2	90.1	89.7	50.4	59.0	21.6	23.7	102.8	92.1
		R.WHIQLGNIVIPK.S	3	97.6	95.8	12.0	9.5	37.3	36.0	8.5	10.0
MSMEG_2410	MSMEG 2408	K.VKEIVGEVPGL	2	11.2	15.3	21.0	18.8	23.0	24.3	46.3	56.4
		K.VVLGLYEDFLC*PAC*GNFER.S	2	36.8	38.9	31.6	31.2	10.3	29.5	9.7	22.5
		R.FHTALYTPELQPQENSGIYPDNAR.L	3	192.0	212.3	103.4	118.5	155.5	180.6	83.3	101.6
		R.INGEDYSPTTPDALVAK.V	2	71.4	84.5	43.3	46.2	66.9	74.3	31.9	39.0
MSMEG_2412	MSMEG 2410	K.HSGADAVYPGYGFLSENPDLAAK.C	3	207.7	204.8	276.0	262.6	132.6	151.8	231.1	237.3
		K.LAEQIVEAGAHVLAIK.D	2	55.6	39.9	70.9	47.8	77.4	48.1	125.5	68.4
		K.LAEQIVEAGAHVLAIK.D	3	172.5	161.0	186.7	162.6	187.2	175.0	260.5	212.6
		K.LPPLDLQAPPPAGSK.Q	2	171.7	174.0	249.3	241.8	153.8	159.3	311.3	331.8
		K.LPPLDLQAPPPAGSK.Q	3	40.1	43.3	56.3	68.0	29.0	34.8	51.9	66.9
		K.LTADDEALLAQPGPK.R	2	145.1	140.1	194.0	184.6	133.9	131.7	219.6	191.0
		K.LTADDEALLAQPGPK.R	3	17.1	19.3	21.4	25.2	11.5	15.7	16.9	23.5
		K.VVELAPAPNLSDELR.Q	2	439.2	430.0	540.6	544.3	471.4	453.1	711.0	646.5

K.VVGDLALALVGAGITAEEF AEDPAK.Y	3	34.4	38.1	43.3	48.1	19.7	22.5	29.0	34.0
K.VYAPFESGLPGPTGR.V	2	250.9	253.2	392.9	380.5	223.1	231.1	421.7	444.9
K.VYAPFESGLPGPTGR.V	3	10.9	14.0	13.7	17.0	7.0	9.4	8.7	10.8
K.YDIPDSVIGFLR.G	2	14.9	19.1	21.6	28.6	9.9	9.3	23.4	19.7
R.AAYEMGIATVAVYPYEDR.N	2	72.8	50.0	68.4	58.3	61.2	50.9	70.0	63.3
R.AVC#DLEPYWEAVR.K	2	115.2	113.1	93.6	94.1	93.3	102.4	103.1	106.2
R.AVC*DLEPYWEAVR.K	2	113.2	111.4	93.5	94.1	93.0	102.0	103.1	106.2
R.AYLSVDEIIR.V	2	57.7	50.7	78.1	70.6	47.7	46.2	76.0	74.1
R.EAESAFGDASVYLEQAVL NPR.H	3	50.4	46.2	45.7	49.1	38.5	41.8	53.9	53.1
R.ESLAEAIEAASR.E	2	54.6	39.7	83.6	66.2	41.5	32.4	80.9	66.7
R.ETYGDTSSLSANQFFYGL R.Y	2	47.9	40.7	61.0	47.8	38.8	34.8	63.9	47.9
R.ETYGDTSSLSANQFFYGL R.Y	3	13.7	17.4	23.3	27.4	5.0	11.6	11.2	21.1
R.FEEIEANYAAADR.V	2	61.1	62.2	46.3	41.0	20.4	25.2	17.8	23.3
R.GELGDPPGGWPEPLR.T	2	112.2	114.1	146.2	145.2	88.7	96.7	152.7	164.2
R.GVSTNIPFLQAVIDDPDFR .A	2	122.1	108.9	140.1	127.3	121.3	132.0	160.3	173.9
R.GVSTNIPFLQAVIDDPDFR .A	3	28.0	33.2	31.6	36.4	17.2	27.4	26.5	36.1
R.IAAGETLADLGLSQDR.L	2	444.2	428.7	310.2	312.5	411.3	386.9	351.5	366.1
R.IAAGETLADLGLSQDR.L	3	27.1	30.2	34.8	40.4	12.3	21.6	22.5	33.9
R.IFDALNNVESMR.P	2	21.9	19.8	19.9	16.0	8.2	7.2	4.3	5.9
R.IFDALNNVESMRPAIDAVR .E	3	224.5	240.4	270.2	266.9	228.5	204.9	313.4	250.2
R.ILNYLADITVNKPHGERPS TVYPQDK.L	4	78.1	84.9	110.2	104.1	73.1	66.5	114.7	107.7
R.IQVEHTVTEEITDVDLVSS QLR.I	3	366.2	358.4	504.9	515.5	310.4	337.9	553.3	589.0
R.KVYAPFESGLPGPTGR.V	3	11.2	11.3	20.8	19.1	11.2	14.1	28.1	27.1
R.LKADESYQIGEVGHPVR.A	2	2.7	1.5	3.9	3.7	7.2	2.7	9.0	2.5

		R.LKADESYQIGEVGHPVR.A	3	72.0	62.4	153.0	133.4	74.1	56.6	109.1	90.2
		R.LKADESYQIGEVGHPVR.A	4	63.7	49.5	71.5	59.5	52.4	49.4	67.4	65.1
		R.LLFPGPTAEFEAHR.E	2	20.3	16.5	34.7	31.6	25.9	17.6	51.7	35.5
		R.LVELGPEGFAGWLR.E	2	161.0	179.8	273.8	281.8	211.3	188.5	386.2	340.1
		R.TTGLLM#VAPYVAR.S	2	17.8	17.3	78.4	81.0	12.0	13.6	37.2	41.2
		R.TTGLLM*VAPYVAR.S	2	17.8	17.3	78.4	81.0	12.0	13.6	37.2	41.2
		R.TTGLLMVAPYVAR.S	2	66.8	63.6	98.4	88.8	55.8	52.4	114.6	89.4
		R.VTTSFIDDRPHLLTSR.S	3	51.6	68.2	1931.8	2100.2	48.1	47.5	532.5	560.7
		R.VYTHEIPGGQLSNLR.Q	2	45.6	36.5	89.1	85.9	55.2	49.2	90.2	87.0
		R.VYTHEIPGGQLSNLR.Q	3	217.8	208.9	263.7	256.7	197.2	195.5	291.1	274.1
MSMEG _2413	MSMEG 2411	K.NIAALDVAGATVR.R	2	22.0	26.8	27.7	32.7	14.1	13.2	19.5	21.6
MSMEG _2414	MSMEG 2412	K.EVASLGGDVSALLPSPVN R.R	2	80.3	87.8	117.1	150.9	67.6	67.6	98.6	99.7
		K.HVAGVDTFFVATTPQYSF VSSSLAK.E	3	106.5	107.2	123.6	122.4	85.8	83.3	105.5	100.8
		R.VESGQGLVVDFVK.S	2	88.6	83.9	160.0	144.2	80.3	80.9	151.6	145.2
MSMEG _2415	MSMEG 2413	K.LDIGSAEFITELEK.F	2	4.7	4.9	172.6	161.9	2.6	4.7	245.4	209.8
MSMEG _2418	MSMEG 2416	K.SSILADGVESLLGAIYLEH GLTVVR.E	3	15.3	15.9	18.3	18.4	4.8	5.1	12.4	15.7
		R.ASIVNTQALADVGR.G	2	65.6	68.3	149.3	177.4	66.0	54.8	177.5	195.5
		R.LFGELLDTAPTLGAGLDW K.S	2	34.3	37.8	47.0	42.3	14.7	19.5	23.4	33.2
MSMEG _2421	MSMEG 2420	K.IALAAC#TGM*ASDQPLSR .R	2	11.9	13.2	4.8	5.3	5.2	6.6	5.7	4.8
		K.IALAAC#TGMASDQPLSR. R	2	13.0	0.0	6.1	0.0	5.2	6.6	20.5	7.0
		K.IALAAC*TGM#ASDQPLSR .R	2	12.0	0.0	4.5	0.0	7.8	10.7	5.4	4.5
		K.LEIDLSGLSEADVK.R	2	18.3	0.0	58.4	0.0	8.7	10.0	42.2	51.4
		K.SGTEVTFSVADTVLPK	2	106.4	108.1	77.5	76.9	93.4	98.6	75.6	86.4
MSMEG _2423	MSMEG 2422	R.VNPLALEEFAALEER.Y	2	15.4	15.9	19.1	19.4	14.6	16.0	19.2	18.3

MSMEG _2424	MSMEG 2423	K.RPIADVQLPEPAVEPEAPP AR.D	3	109.4	124.8	80.1	92.6	117.6	127.5	51.2	104.3
		R.IDTTGLPAVGDDATIPR.D	2	54.4	60.9	54.9	63.1	49.7	66.5	49.1	75.7
MSMEG _2426	MSMEG 2425	K.LITAIVKPFTLEDVK.T	2	6.0	8.6	217.5	240.9	6.2	4.7	435.6	320.7
		K.LITAIVKPFTLEDVK.T	3	17.6	13.5	518.6	431.2	16.2	17.1	689.0	683.8
		K.TGLEQTGILGM#TVSEVQ GYGR.Q	3	11.8	11.8	119.6	95.5	13.3	16.2	85.0	95.0
		K.TGLEQTGILGMTVSEVQG YGR.Q	2	2.7	5.2	258.9	276.0	2.2	1.8	254.6	215.2
		K.TGLEQTGILGMTVSEVQG YGR.Q	3	9.9	9.5	212.4	175.6	5.4	7.5	183.3	193.8
		K.VRVEVVVDDSAVDK.V	2	1.3	1.2	33.1	30.0	2.0	1.7	63.3	41.9
		K.VVDVIVQAAR.T	2	3.2	3.1	92.2	79.0	2.1	1.8	87.6	64.2
		K.VWVSPVETVVR.V	2	70.1	80.6	1613.5	1942.6	64.8	66.4	1898.8	1824.7
		R.GAEYSVDFVPK.V	2	30.4	21.7	39.6	31.6	24.8	30.5	48.9	54.0
		R.VEVVVDDSAVDK.V	2	0.9	1.4	42.5	48.4	0.6	0.4	35.1	24.4
		R.VEVVVDDSAVDKVVDVIV QAAR.T	3	0.2	0.5	34.2	30.9	0.8	0.7	80.1	86.1
MSMEG _2427	MSMEG 2426	K.LWYPLWDANIR.L	2	0.4	0.4	60.8	67.0	0.1	0.1	91.3	105.1
		K.VAGEDISVGLAMLDVR.H	2	10.1	9.1	118.2	140.7	15.5	12.3	126.0	117.9
		R.AGEILAAVPANHVPAPPR. L	3	0.4	0.1	120.0	109.3	1.1	0.5	137.7	146.6
		R.DALLDLYEFWLTTK.A	2	4.1	9.5	9.6	19.7	2.3	9.6	7.1	15.0
		R.ELLLAQHADEIGAALR.I	2	3.6	3.5	179.7	202.5	3.7	2.1	92.3	175.9
		R.ELLLAQHADEIGAALR.I	3	0.7	0.3	43.8	48.5	0.2	0.7	66.7	47.3
		R.HIAGDADLSSLLVGGAR.R	2	14.2	16.3	647.1	572.9	6.8	11.1	888.1	902.3
		R.LLWADGSNPGELIVQIR.T	2	6.7	8.3	95.5	99.9	4.1	5.6	132.4	109.4
		R.PANDLAAAVSQLLTGGPR .Q	3	0.0	0.1	28.2	30.2	0.2	0.0	28.9	24.3
		R.VAAASATTGLPIAVSTLSR .L	2	0.4	0.2	229.1	219.5	0.3	0.5	189.6	242.9

		R.VSRPDLLLLGALC*HDIGK.G	3	0.2	0.1	26.6	24.7	0.4	0.1	32.5	33.6
MSMEG_2435	MSMEG2433	K.EEPSLIQIDSER.A	2	732.0	563.2	489.8	365.3	793.1	911.5	436.2	482.4
		K.KDEAAEAPAEAAEAPAEAADAASES	2	388.7	467.1	335.0	410.1	543.4	585.4	441.3	467.6
		K.KDEAAEAPAEAAEAPAEAADAASES	3	477.7	630.3	386.6	477.3	542.2	688.8	412.8	528.0
		K.LDLFNAALAEAESGTTAAATTPK.K	2	576.8	727.7	404.1	464.2	728.6	738.8	486.0	443.3
		K.LDLFNAALAEAESGTTAAATTPK.K	3	1150.0	1103.6	649.7	627.8	1204.9	1356.7	631.3	646.1
		K.PSKLDLFNAALAEAESGTTAAATTPK.K	3	27.1	33.1	56.7	92.4	202.9	201.3	282.6	288.5
		R.AQYWLGVGAQPTEPVLALLK.I	2	610.1	647.0	550.6	573.1	706.1	704.9	592.4	584.7
		R.AQYWLGVGAQPTEPVLALLK.I	3	297.0	252.0	196.9	166.4	248.1	283.7	155.7	159.2
		R.YHPKEEPSLIQIDSER.A	3	12.2	12.8	31.1	44.6	58.9	65.1	65.6	78.3
MSMEG_2437	MSMEG2435	R.EILVPFVGAIVTSVSR.Q	2	10.3	11.9	17.0	17.9	10.7	13.5	12.9	12.4
MSMEG_2440	MSMEG2438	R.DDIPTFSPGDTVNVHVK.V	2	383.5	397.8	227.3	235.3	441.2	394.7	233.2	185.9
		R.RQGGGISETFTVR.K	3	3.1	2.6	3.4	1.4	11.9	10.8	2.4	2.8
		R.TFPVHSPNIDHIDVLTR.G	2	101.4	88.5	37.9	32.8	138.0	115.3	40.5	27.6
		R.TFPVHSPNIDHIDVLTR.G	3	659.7	839.3	337.0	418.9	810.7	781.6	333.8	316.3
MSMEG_2443	MSMEG2441	K.DIVGQFSYVVETER.R	2	45.5	50.4	28.7	29.9	41.4	37.9	25.4	23.1
		K.DVNIEEVEKPELR.L	2	33.1	36.9	54.1	57.8	35.3	35.3	53.4	42.4
		R.FYLANSVELIPR.N	2	36.6	40.5	30.8	32.0	39.3	42.1	31.0	30.5
		R.NADGEVYFELR.L	2	7.7	8.8	2.6	5.1	8.0	9.6	4.6	3.7
MSMEG_2450	MSMEG2446	R.SSFYAETEEQESQR.A	2	23.2	19.8	2.2	2.6	26.3	23.8	1.5	1.8
MSMEG_2468	MSMEG2467	K.IAELTESGTVATGSAQK	2	11.8	12.0	7.6	9.0	8.6	10.3	3.8	5.8
MSMEG_2511	MSMEG2512	K.AFIEVAGPDDELELK.A	2	269.9	289.1	116.1	116.4	153.9	170.6	101.7	101.0

		R.LVLGGNNAGSGTGFGTF KPSEFSDSYVK.L	3	121.7	135.6	50.6	60.6	106.9	113.0	44.8	49.9
MSMEG _2516	MSMEG 2520	R.LHTGTLVLNNDLR.H	3	27.1	18.9	1.9	1.9	5.0	3.6	3.1	2.6
		R.LTHFGPAGLLDR.I	2	68.6	65.6	135.2	157.5	32.9	41.0	132.5	141.4
		R.LTHFGPAGLLDR.I	3	98.0	94.1	9.0	5.4	31.4	30.1	9.5	7.0
		R.VADIAGLAGFSHNHDATEI R.L	3	129.0	161.7	49.7	55.5	76.3	75.3	61.3	53.4
MSMEG _2519	MSMEG 2522	K.ETVAHGGTVLFVGTK.K	2	182.9	205.5	323.6	443.3	80.0	67.6	90.3	108.9
		K.VIASAVAEGLQAR.A	2	41.4	30.8	10.7	11.3	30.0	32.2	16.3	18.2
		K.VPSAVWVVDTNK.E	2	230.5	176.9	127.6	123.9	127.0	132.9	83.9	119.7
		K.VPSAVWVVDTNKEHIAVG EAR.K	4	79.9	83.4	25.7	26.0	178.3	173.3	84.0	101.8
		R.LKELEAMEQTGGFEGR.T	2	341.2	317.8	301.7	310.8	229.9	211.0	122.8	145.8
		R.LKELEAMEQTGGFEGR.T	3	279.9	272.8	208.4	213.5	201.9	206.2	110.9	120.8
MSMEG _2524	MSMEG 2527	K.ISGQDTAQLTESQFAALR. H	2	1.6	0.6	116.1	133.8	0.3	0.9	147.9	141.0
		K.STLLNIIGGLDRPTK.G	3	0.0	0.0	25.8	24.0	0.0	0.0	32.8	40.3
		R.ATELLEMVGLGHR.I	3	1.5	2.5	18.5	16.6	3.8	2.6	22.8	15.1
MSMEG _2525	MSMEG 2528	K.VSQDDALAHLAVFDLTNQ DEAQK	3	25.9	33.8	472.9	452.2	20.5	27.6	483.0	466.9
MSMEG _2526	MSMEG 2529	K.AGNYTEQFMFDADNRPA FTEFR.Q	3	9.4	7.5	198.9	174.2	5.8	10.8	235.7	223.7
		K.DVIPVM#VQEGSVIYDR.A	2	0.2	0.3	43.0	38.8	0.2	0.1	41.8	46.7
		K.DVIPVMVQEGSVIYDR.A	2	1.8	1.3	367.1	331.1	2.0	0.5	423.2	385.4
		K.FVM#IQLAEPEKNEK.L	3	4.0	3.0	24.1	26.4	1.5	0.2	29.5	31.6
		K.FVMIQLAEPEKNEK.L	2	6.1	9.1	41.6	30.7	3.6	5.1	85.4	49.1
		K.FVMIQLAEPEKNEK.L	3	11.1	13.1	181.2	162.8	7.4	11.5	237.4	210.3
		K.IEITQPDGPSFTVDGWNV TWQK.W	3	17.5	16.9	27.0	34.2	23.9	22.6	14.0	35.6
		K.LIYEAIVDLGAR.V	2	0.6	0.8	99.3	74.9	0.1	0.4	173.2	150.5
		K.LNEFGAPVGYK.I	2	0.1	0.4	66.6	53.7	0.3	0.3	48.5	53.5
		K.LTFVGMDVPR.R	2	10.6	11.1	226.1	207.7	5.8	9.4	244.5	226.8

		K.LTGVLTTGSIPEGETPR.W	2	0.5	0.5	1009.6	965.3	1.1	1.0	1125.5	1014.7
		K.M#VAPGMYGPNHQHFFNFR.M	4	12.8	9.9	23.4	26.7	10.5	11.8	9.0	16.5
		K.MVAPGMYGPNHQHFFNFR.M	4	0.0	0.1	15.2	18.7	0.2	0.1	23.5	28.2
		R.AALSEM#VVPYGDSSPTHWNK.N	3	4.0	4.1	53.6	55.8	0.1	0.9	43.9	55.7
		R.AALSEMVVPYGDSSPTHWNK.N	3	6.0	9.2	210.4	238.4	4.6	8.1	230.5	237.4
		R.EGLTLHEITYNDR.G	2	81.2	91.1	129.5	105.6	81.2	82.3	173.8	110.9
		R.FPSYLVEHM#TGVEEK.V	3	0.0	0.0	34.5	36.7	0.0	0.0	44.0	46.5
		R.FPSYLVEHMTGVEEK.V	2	118.5	102.5	236.2	237.6	125.9	71.4	284.9	301.4
		R.FPSYLVEHMTGVEEK.V	3	7.8	7.9	115.7	123.1	2.8	4.2	132.4	146.9
		R.FVQHNLWVTK.Y	3	0.0	0.2	3.8	2.6	0.0	0.1	4.7	5.7
MSMEG_2528	MSMEG2531	K.GATPFDVETLEAALTR.L	2	98.6	88.0	228.1	221.8	52.3	44.2	186.9	151.2
		R.DVLSASTFGVGQLIASALDR.G	2	35.5	36.4	94.7	92.2	10.2	13.5	35.2	46.0
		R.LVAITVPGTPIEQALR.E	2	145.9	126.2	405.4	354.7	96.5	86.8	313.1	330.1
MSMEG_2540	MSMEG2543	R.SGAQVAVVIGGGNFFR.G	2	31.7	31.9	69.5	67.7	22.5	19.6	29.6	31.7
		R.TNPDAELITAISHR.E	2	101.7	101.6	79.9	90.3	113.6	111.6	82.2	78.2
		R.VQTAITMGQVAEPYIPLR.A	2	76.6	79.7	105.6	110.6	59.1	63.2	86.5	87.1
MSMEG_2541	MSMEG2544	K.STHTYTAQIDELVK.H	2	6.5	9.7	47.4	37.5	9.7	7.8	30.3	46.4
		R.LVVIKPYEASQLR.A	3	39.7	43.2	108.6	95.9	57.7	50.6	138.7	126.0
		R.NSDLGVNPSNDGNIIR.V	2	19.4	17.7	14.3	12.6	19.2	24.2	12.3	16.0
MSMEG_2553	MSMEG2555	R.ILVATAEVLAR.S	2	10.2	10.1	9.4	11.7	6.2	6.7	3.4	5.7
MSMEG_2580	MSMEG2581	K.TVPEAQIVETLIEEAMR.L	2	118.6	114.9	72.1	70.1	55.4	67.2	44.5	60.6
		K.VGNQILESLNLRPR.G	3	359.9	361.4	287.0	308.9	262.2	280.3	259.9	263.0
		R.HSQIPVIADIHFQPK.Y	3	72.5	63.6	62.2	73.3	48.9	45.7	27.4	51.9

MSMEG _2587	MSMEG 2588	R.IAHEYMIDHGAYPSTLGYK .G	3	36.4	37.8	5.7	6.4	13.0	23.4	4.8	5.2
MSMEG _2594	MSMEG 2595	K.YSDIVAQHFGTDHHQIR.I	3	18.5	15.8	3.6	3.0	16.6	12.5	2.0	1.2
		K.YSDIVAQHFGTDHHQIR.I	4	26.1	24.2	14.7	12.1	9.8	6.2	3.7	4.0
		R.VDGDPSGVFVTDHFGAP GAETGVDR.A	3	300.9	306.8	28.7	40.4	277.6	324.9	20.1	19.2
MSMEG _2598	MSMEG 2599	K.VAVITGGASGIGLATGR.R	2	45.7	49.9	167.8	184.1	53.2	63.2	174.8	194.8
		R.AEGATVVVGDIDPTTGK.A	2	40.3	37.1	190.3	186.6	60.6	74.9	147.4	181.4
MSMEG _2606	MSMEG 2607	R.APGIFVGDLAEVLGR.S	2	8.1	9.6	26.4	22.0	11.2	9.8	21.5	21.3
MSMEG _2613	MSMEG 2614	K.AVNVNEQFQVSR.Q	2	102.3	94.0	70.4	64.7	124.2	114.8	72.1	66.2
		K.FVFVGAGGGALPLLQK.S	2	103.4	90.5	64.0	51.4	163.2	128.5	75.2	63.3
		K.GFGGFPVGGAFLR.T	2	46.5	44.4	17.6	21.3	75.3	70.0	26.3	27.0
		K.LFEEVWSWGTK.V	2	62.5	55.9	6.6	6.0	50.7	60.8	4.9	4.2
		K.YLIGQLLLSEPAR.V	2	93.9	140.6	80.1	78.6	153.8	127.4	105.5	93.5
MSMEG _2616	MSMEG 2617	R.DAPQALIDAADLVTEMTK.I	2	37.7	37.0	54.0	52.0	39.1	34.9	49.8	54.7
MSMEG _2621	MSMEG 2622	K.DFPVILYQIQTK.Y	2	178.0	180.8	209.1	209.3	150.9	101.8	251.0	172.8
		K.DSYSFDVDDDGLK.N	2	119.1	136.5	83.1	107.6	63.1	74.2	71.9	101.4
		K.FKDAELLGMPWIVVVGR. G	3	43.1	46.3	22.2	28.0	42.3	47.5	35.3	34.6
		K.TREPGGEWELLAVGVPG DR.E	2	120.0	118.0	257.7	248.2	193.8	211.2	101.9	165.8
		R.AGAAELVADLDR.L	2	185.0	170.0	175.7	169.9	165.6	130.7	241.7	181.9
		R.AVGPGIYSWLPLGLR.V	2	157.9	152.2	170.1	159.5	187.5	152.2	293.1	243.0
		R.DFTPDGTIEAAEVR.D	2	263.9	248.6	447.0	450.1	206.9	204.3	452.3	600.7
		R.GIEIGHIFQLGR.K	2	50.3	47.9	111.6	104.0	38.0	36.2	107.3	108.2
		R.GIEIGHIFQLGR.K	3	58.1	44.1	36.0	27.6	57.9	45.8	68.6	51.9
		R.GWADGVVELR.N	2	73.2	66.4	62.0	59.1	63.5	53.3	88.1	81.3
		R.KYTDAFSADVLGEDGKPL R.L	3	136.9	135.8	137.9	153.5	119.8	109.4	151.9	183.8

		R.KYTDAFSADVLGEDGKPLR.L	4	41.2	34.1	14.5	17.3	35.9	38.1	30.5	27.2
		R.LGAALEPAEFALLDDADFAANPFLVK.G	3	106.7	172.8	107.5	187.1	50.9	119.1	74.0	176.1
		R.LTMGSYGIGVSR.L	2	198.9	175.6	293.6	247.8	152.4	107.8	233.4	181.8
		R.LVAVIAEQQHDQLGLR.W	3	96.5	72.7	85.1	55.1	83.0	77.2	84.1	96.5
		R.NNDYLLGPTHEELFTLTVK.G	3	116.7	129.8	105.8	115.0	123.5	86.6	159.3	121.9
		R.SEMNAIGAQEILLPALLPR.G	2	122.9	115.5	176.0	155.9	78.2	80.3	175.1	172.6
		R.SEMNAIGAQEILLPALLPR.G	3	16.5	21.5	7.1	17.7	10.2	9.5	9.4	14.1
		R.WTEYGDTLFR.L	2	37.7	39.1	32.9	39.4	20.5	34.6	27.5	53.8
MSMEG_2628	MSMEG2627	K.EGDVIETYELVEK.A	2	294.1	308.9	358.2	341.8	249.8	271.9	401.4	347.2
		K.PAAPKPAAPQPPAAPATPAAPAAEATPAAPAKPSAPK.P	4	100.0	103.6	68.6	72.1	76.6	94.9	63.7	73.9
		K.PAAPKPAAPQPPAAPATPAAPAAEATPAAPAKPSAPK.P	5	36.5	44.1	34.3	45.1	30.1	40.7	33.8	46.6
		K.PAAPQPPAAPATPAAPAAEATPAAPAKPSAPK.P	3	60.6	63.5	37.2	39.8	27.3	30.7	19.8	20.6
		K.PAAPQPPAAPATPAAPAAEATPAAPAKPSAPK.P	4	51.9	66.7	41.7	49.3	19.6	24.4	21.9	23.2
		K.RAEYENMQAPVVGGVR.L	2	18.4	16.1	6.4	5.2	12.3	8.4	9.6	5.1
		K.RAEYENMQAPVVGGVR.L	3	47.2	49.4	28.4	27.7	26.7	29.4	24.9	27.7
		K.VGNIAGC#LVTSGIMR.R	2	243.6	228.9	186.4	190.9	263.5	241.5	182.1	213.5
		K.VGNIAGC*LVTSGIMR.R	2	242.9	228.5	186.4	190.9	263.2	241.0	182.1	213.5
		R.AASAAPSAPAAPAPGATPGPR.P	2	1.1	1.3	0.6	0.5	0.4	0.5	0.2	0.2
		R.AASAAPSAPAAPAPGATPGPRPTPGATPGPR.P	4	87.0	101.6	60.5	65.1	66.1	81.3	58.4	59.7

		R.AEYENMQAPVVGGVR.L	2	61.9	55.4	30.9	30.1	39.7	33.2	25.1	21.5
		R.DGYEC#GLTLTYNDIK.E	2	22.7	21.8	38.3	40.7	15.0	19.3	33.4	37.2
		R.DGYEC*GLTLTYNDIK.E	2	22.7	21.8	38.2	40.7	15.0	19.3	33.4	37.2
		R.DNVVVAQNLTVSSLR.R	2	260.9	244.4	176.1	165.7	288.4	234.6	207.0	182.8
		R.GQGGNGASAPAAPKPGAPAPSPATAAK.P	3	9.0	9.4	6.1	6.7	7.2	7.4	6.6	6.5
		R.ISLEDLDSALK.E	2	160.4	143.2	93.6	91.7	149.5	126.6	108.1	88.9
		R.ISLEDLDSALKETSQLNLILK.G	3	2.1	2.3	2.5	2.3	12.7	9.2	12.6	8.0
		R.VGNNPFSTQQPADRPIPR.P	2	8.2	6.4	2.5	3.7	10.8	5.7	5.7	2.4
		R.VGNNPFSTQQPADRPIPR.P	3	518.7	417.8	482.3	364.2	433.9	306.5	397.8	338.0
MSMEG_2641	MSMEG2640	K.ELGDTTELPDISPK.W	2	387.6	370.7	292.4	282.7	287.2	290.7	315.4	308.8
		R.AGLVTEVVAHDDLLTAAR.R	2	56.5	60.5	59.8	59.8	36.4	36.1	71.2	87.3
		R.IDALQTGGALWAEAEAAR.Q	3	84.5	95.6	12.2	12.5	36.2	40.3	20.0	12.3
		R.MSLTGDYLSAQDALR.A	2	157.7	185.0	105.8	116.8	106.4	117.4	117.3	117.9
MSMEG_2653	MSMEG2652	R.YQAAVSVGTNPTFSGR.T	2	13.6	13.2	15.5	15.6	12.5	17.5	10.9	10.8
MSMEG_2654	MSMEG2653	K.EILGQYGLHDTDTGSPEAQVALLTK.R	2	74.6	66.3	19.8	17.7	77.9	73.3	21.2	17.4
		K.EILGQYGLHDTDTGSPEAQVALLTK.R	3	2002.9	2069.0	968.7	1006.9	2205.0	2215.0	1131.9	1114.5
		K.KEILGQYGLHDTDTGSPEAQVALLTK.R	3	82.5	76.4	20.1	19.1	81.1	84.7	18.2	24.4
		K.KEILGQYGLHDTDTGSPEAQVALLTK.R	4	109.2	0.0	46.2	0.0	109.9	130.6	54.2	61.4
		K.RIQDLTEHLK.V	3	5.2	3.5	0.7	0.3	6.2	5.4	0.9	1.4
		K.YVAQVDVAR.Y	2	8.1	6.2	2.7	1.9	9.2	6.7	3.2	1.8
		R.GLLLLVGR.R	2	228.8	170.6	90.4	67.1	212.9	205.9	88.1	82.3
		R.RGLLLLVGR.R	2	45.4	32.0	6.8	4.4	61.2	58.1	16.1	13.5
MSMEG_2656	MSMEG2655	K.ALC#AAQQELADR.A	2	5.1	4.5	4.0	4.4	3.9	4.8	3.7	4.4

K.APVAGIAMGLVSDDVDVDGK.V	2	382.0	351.3	269.1	258.9	300.2	239.7	253.3	212.2
K.APVAGIAMGLVSDDVDVDGK.V	3	155.8	168.4	81.7	85.5	128.3	110.0	91.8	56.4
K.DFVTALQLDTK.L	2	307.8	275.5	212.6	208.3	360.7	256.4	338.9	288.1
K.DHFDFFPLTVDVEER.M	2	158.4	183.1	94.3	114.1	168.4	187.3	149.6	164.0
K.DHFDFFPLTVDVEER.M	3	168.1	160.8	93.6	98.1	198.8	166.7	167.0	133.4
K.INAIANPQLPK.V	2	32.9	30.6	11.9	15.9	29.8	23.7	15.1	12.4
K.ISLVLVAEESAESAESAGDK.G	2	165.1	137.8	110.8	92.1	134.8	102.8	106.4	81.1
K.ISLVLVAEESAESAESAGDKGAEK.A	3	272.0	256.7	275.2	323.1	455.0	384.9	439.8	455.6
K.LDGIPSQVLAGALSQAK.D	2	893.1	894.8	678.7	701.0	940.7	883.7	963.7	992.6
K.LDGIPSQVLAGALSQAK.D	3	126.2	149.1	72.3	83.5	133.4	126.0	98.3	93.4
K.LRVEIADIDNR.G	3	57.8	35.9	19.0	9.5	61.0	44.4	40.8	20.8
K.TTDFGAFVSLLPGR.D	2	563.8	560.9	521.8	514.8	612.4	521.9	771.4	674.9
K.TTDFGAFVSLLPGR.D	3	20.4	21.6	2.5	4.8	28.6	24.1	10.5	11.2
R.ALSAEVAVIPR.A	2	418.3	306.1	334.5	235.8	490.2	246.9	477.8	242.1
R.ALVPVLPSIEEFPYAIR.Q	2	1542.5	1454.9	1094.6	944.3	1341.6	1537.4	1286.6	1354.2
R.ALVPVLPSIEEFPYAIR.Q	3	117.7	151.4	75.9	104.6	99.4	124.3	100.2	111.5
R.AVFDMVVAGR.I	2	279.3	197.8	159.6	113.9	302.3	169.3	238.4	128.1
R.GETQILGVTTLDM*IK.M	2	132.4	134.8	85.7	91.6	106.3	99.3	107.2	94.5
R.GETQILGVTTLDMIK.M	2	550.3	531.4	478.2	440.5	612.4	453.1	639.3	490.4
R.IALIDGTWVAFPTVEQLER.A	2	527.7	527.9	406.6	386.9	470.2	591.9	481.3	577.9
R.LAQQAAGSAVAYLDDETM*LLSATTASK.N	3	136.4	146.1	74.7	78.3	116.6	104.9	62.8	70.7
R.LIDRPLRPSFVDGLR.N	4	14.4	13.9	7.0	8.6	7.8	9.5	4.2	6.7
R.LTILDVMAEAIDRPDEM#SPYAPR.I	3	101.5	109.4	63.6	64.0	70.3	73.2	57.9	66.0
R.LTILDVMAEAIDRPDEM*SPYAPR.I	3	101.5	109.0	63.6	64.0	70.2	73.1	57.9	66.0

		R.LTILDVMAEAIDRPDEMSPYAPR.I	3	354.1	467.6	249.2	315.7	215.9	356.6	208.7	328.3
		R.LTILDVMAEAIDRPDEMSPYAPR.I	4	11.9	23.1	1.9	7.4	7.1	10.7	2.6	5.1
		R.PLRPSFVDGLR.N	3	60.3	33.5	17.6	4.2	39.6	9.1	11.0	0.7
		R.TDEIKVEVLER.L	2	123.1	94.8	228.0	195.3	123.4	90.2	261.5	176.3
		R.TDEIKVEVLER.L	3	26.7	21.0	4.8	2.9	30.7	30.2	10.1	11.3
		R.YMHHYNFPPYSTGETGR.V	3	165.5	163.4	102.2	105.9	86.8	119.9	72.4	105.2
		R.YVALTDILGAEDAFGDMDFK.V	2	235.7	242.6	161.2	163.6	173.7	201.9	144.7	181.9
MSMEG_2658	MSMEG 2657	K.TGSGDYGSTNDVGIAYGPQGQR.I	3	26.8	25.9	10.3	9.6	12.6	21.3	5.3	12.1
		R.NNASIGIYAVDLDSNR.T	2	39.8	46.6	4.4	10.8	16.7	27.6	6.0	9.5
		R.WETELNSAVPGDPR.D	2	40.2	47.7	8.2	10.9	28.2	34.6	11.3	12.8
MSMEG_2664	MSMEG 2662	R.KGLPPNPDATSTGLDGAR.G	3	5.4	4.0	2.8	2.2	5.3	5.8	3.4	4.5
		R.KISGLQGLTVGIEPLLDLS	2	82.5	83.5	84.7	84.9	75.9	74.1	99.8	93.6
MSMEG_2667	MSMEG 2666	K.AAHLPSAIDGPTVGSSPLK.L	3	114.1	90.4	146.0	147.8	63.9	58.2	198.0	228.7
		K.GTSVGGIMLAMVESVDHVTETANR.L	3	30.9	27.8	12.8	15.0	11.2	11.2	6.2	6.4
		R.DTGFGENPATLAYAR.S	2	34.6	39.8	31.7	37.8	15.4	21.6	17.4	22.9
		R.LPDVPIVALVETAR.G	2	112.5	115.3	304.6	334.9	70.0	59.7	158.5	164.0
MSMEG_2669	MSMEG 2667	K.LAGFGYVVLVPDVYYR.I	2	328.9	364.1	129.8	137.2	49.0	59.1	76.7	104.9
		R.IAVEDDPDSPHLLADK.I	2	243.6	182.8	93.4	73.9	67.5	71.2	90.6	89.7
		R.IAVEDDPDSPHLLADK.I	3	588.3	526.7	217.8	204.5	133.5	153.1	206.0	238.3
		R.IPNWGPINLNTVFSDQDER.K	2	403.1	432.0	135.0	123.5	110.0	128.6	141.0	146.3
		R.VAAALSFHGGR.I	2	13.0	9.7	7.8	6.4	2.8	1.7	7.4	8.1
MSMEG_2671	MSMEG 2669	R.LIWAQSTSGIIGR.D	2	37.5	38.5	62.0	58.4	21.7	26.4	51.6	64.4
MSMEG_2684	MSMEG 2682	R.DMLTAFNSGDVATAR.K	2	85.6	88.9	67.3	66.4	117.2	112.4	71.0	77.0
		R.KINVNLAPLAAAQAR.L	2	20.1	19.0	846.4	966.7	32.4	28.4	923.1	965.0

		R.LPQIPATTEEVEALAADMR.A	2	102.5	109.5	221.7	200.2	93.4	92.1	444.0	398.3
MSMEG_2688	MSMEG2686	K.THGGAPAIGALFGAIGSLLDGAPDIK.T	3	58.5	57.4	26.0	25.7	23.2	29.7	22.7	29.1
MSMEG_2698	MSMEG2697	R.DYFDYLDIFK.A	2	64.5	66.8	65.2	64.6	56.8	60.2	57.1	52.4
		R.GLLGM*VVPSLR.A	2	36.0	34.6	31.4	28.2	26.9	28.2	20.6	19.5
		R.GLLGMVVPSLR.A	2	36.0	34.6	31.4	28.2	26.9	28.2	20.6	19.5
		R.TDVLTFGPLR.V	2	101.9	92.5	87.2	85.3	92.5	91.0	82.6	75.2
MSMEG_2723	MSMEG2725	R.AEIEGEMGDSHVGLQAR.L	2	16.0	8.4	2.7	0.5	2.5	2.6	0.2	0.4
MSMEG_2727	MSMEG2729	K.EKVDFAGPYLITGQSLLVR.A	2	31.8	37.2	68.4	77.9	51.2	53.2	93.9	105.5
		K.EKVDFAGPYLITGQSLLVR.A	3	78.2	76.0	143.0	144.9	139.1	135.8	192.2	177.7
		K.NGAVDALTTDEVILAGYAAQSPGTFK.L	3	226.6	284.7	2689.8	3302.0	571.9	658.1	2356.4	2754.9
		K.NLGPAGIQAPQPPAVDRD.-	2	1.1	3.9	127.7	129.4	3.6	4.3	150.8	115.3
		K.NLGPAGIQAPQPPAVDRD	2	158.0	150.1	685.0	721.0	247.0	237.9	381.3	411.8
		K.QPDGTLTGFDVDVAK.Y	2	42.1	43.7	96.5	92.5	55.7	54.3	104.0	96.9
		K.VDFAGPYLITGQSLLVR.A	2	237.0	250.2	468.7	451.7	312.2	309.5	500.4	478.9
		K.VDFAGPYLITGQSLLVR.A	3	11.6	14.3	29.1	26.9	21.3	20.9	20.8	27.3
		K.YVANELGYSEDQIEWK.E	2	131.5	135.4	256.6	268.5	199.8	216.3	309.5	319.3
		R.IKDEYPGVQLQQYDTYSAC#VEALK.N	3	145.6	141.5	270.8	251.8	263.5	229.3	384.4	337.3
		R.IKDEYPGVQLQQYDTYSAC*VEALK.N	3	144.7	141.3	269.9	251.1	261.4	228.5	379.8	335.3
MSMEG_2731	MSMEG2734	K.EALAAEAEDIAANSTQWK.A	2	92.2	90.4	79.0	49.8	87.4	75.6	83.4	63.9
		K.SAAAALAESLPTASVLGDVDALSAR.L	3	87.2	97.0	107.0	110.0	110.1	90.2	145.5	119.0

		R.AEALADSTDWGATSAAF R.D	2	165.0	167.5	70.9	93.9	155.2	147.1	84.3	104.5
		R.LSSILEQADEAAQNER.A	2	172.9	160.5	178.8	193.1	201.5	175.6	188.4	191.3
		R.VDPDGTVWLITGSGER.V	2	528.0	674.5	371.1	534.7	479.1	547.0	214.6	337.1
MSMEG _2739	MSMEG 2742	K.DLQIPDDPASAIAAAR.R	2	22.5	22.8	89.7	81.6	37.6	35.2	87.5	80.5
		R.YRDEDAAGVSDADPYAP GADTR.A	3	20.0	20.6	19.5	20.1	18.7	26.4	11.9	17.3
MSMEG _2740	MSMEG 2743	R.ELVGEGSLFLLK.V	2	14.6	12.3	52.8	27.4	17.3	16.2	19.6	21.6
MSMEG _2743	MSMEG 2745	R.FTTVETAVLAVVK.R	2	25.9	24.5	35.6	32.1	21.2	24.1	35.2	27.7
MSMEG _2744	MSMEG 2746	K.FGNPLGVVDNSTVDPADR .Q	2	120.7	124.1	112.2	120.9	77.1	75.0	141.0	133.0
		R.IFTPASELPFAGHPTVGAA WYLR.D	3	164.3	170.1	167.4	166.3	144.1	127.5	200.4	220.1
		R.WSPEGWVQVAGR.V	2	30.3	39.0	31.0	33.2	8.9	16.6	23.2	36.4
MSMEG _2750	MSMEG 2753	R.LTELPVGMPVAVVVR.Q	2	79.6	85.3	171.7	184.0	110.7	93.5	248.6	239.9
MSMEG _2758	MSMEG 2759	K.ASGDFVWDEEESEALR.Q	2	90.1	91.3	35.2	31.3	79.0	63.7	29.6	26.3
		K.RIEAGLYATQK.L	2	0.5	0.4	0.3	0.1	0.3	0.8	1.1	1.1
		K.RIEAGLYATQK.L	3	1.1	0.6	0.1	0.0	0.5	0.9	0.1	0.2
		K.VALLNAEEEVELAK.R	2	240.2	221.3	158.9	124.0	219.9	166.6	151.3	122.7
		R.ELLQDLGREPTPEELAK.E	3	107.6	97.1	136.2	114.8	111.8	126.0	116.8	123.0
		R.GMAFLDLIQEGNLGLIR.A	2	101.7	98.3	75.7	72.6	100.6	98.9	77.7	75.8
		R.IEAGLYATQK.L	2	2.1	1.5	2.8	2.8	1.0	1.7	0.6	2.4
		R.IPVHMVEVINK.L	3	35.6	20.6	9.4	4.4	40.1	10.0	11.7	1.5
		R.TLDEIGQVYGVTR.E	2	232.0	250.5	166.9	181.8	182.5	190.6	138.1	119.7
MSMEG _2760	MSMEG 2760	K.LGVTYPGVVTGGIVR.T	2	66.2	81.1	293.5	324.7	108.2	110.4	315.0	347.2
		R.FKLDTPQPATPEAVAK.T	2	10.3	8.7	72.2	68.8	16.4	17.2	74.3	79.2
		R.FKLDTPQPATPEAVAK.T	3	53.9	58.3	108.8	106.9	56.0	66.4	116.9	124.5

MSMEG _2762	MSMEG 2761	K.STPTDPVTVVDTETER.W	2	139.3	138.5	110.1	122.9	118.7	127.2	130.0	142.4
		K.VIAAAPGVATALR.D	2	14.6	9.4	4.0	0.8	12.1	12.2	3.8	7.0
		R.AEVFGAAGASNDDAAGG QAVR.T	2	4.8	4.5	3.3	2.5	2.2	2.7	13.3	41.4
		R.AVAEQLADEAAELVLR.R	2	125.2	131.0	125.2	124.1	105.0	94.1	124.8	114.5
		R.AVAEQLADEAAELVLR.R	3	24.2	24.5	13.7	17.7	19.8	20.9	16.1	20.0
		R.LAVLRPGEHVLGEEEGGK QEGR.A	3	43.2	37.8	56.5	46.5	49.5	47.6	96.4	91.3
		R.LAVLRPGEHVLGEEEGGK QEGR.A	4	129.3	136.3	127.6	131.2	126.0	138.9	127.8	148.7
		R.RAEVFGAAGASNDDAAG GQAVR.T	3	6.0	7.3	3.1	3.4	7.6	7.3	4.3	4.6
MSMEG _2763	MSMEG 2763	R.RTETDDVSEDSLEELK.A	3	21.2	22.3	18.4	19.2	35.8	38.2	18.4	17.4
MSMEG _2765	MSMEG 2764	K.VSLINLDPQTPVVISR.G	2	158.7	157.1	144.4	176.1	151.5	159.0	170.4	193.2
		R.VGLSIVNSPGTIDAGYR.G	2	167.6	179.1	79.1	93.1	116.9	125.4	46.5	54.6
MSMEG _2766	MSMEG 2765	R.AAAAAQAAAQQNPAAQQ PQQQEGEQPQPPEPVAR.R	3	26.4	38.0	46.5	58.4	15.5	25.1	29.1	45.8
		R.AAAAAQAAAQQNPAAQQ PQQQEGEQPQPPEPVAR.R	4	138.2	203.7	158.4	252.8	94.7	134.3	111.4	167.0
		R.AAAAAQAAAQQNPAAQQ PQQQEGEQPQPPEPVAR.R	5	20.9	23.3	26.2	29.7	14.6	16.5	19.7	23.8
MSMEG _2769	MSMEG 2769	R.MLASLVEEAVAVGDLVR.L	2	21.1	25.2	23.3	27.2	13.6	17.9	15.0	23.1
MSMEG _2771	MSMEG 2770	R.LGIPTVATVPWTTDR.L	2	93.6	96.7	273.2	285.4	95.0	110.8	189.3	237.6
MSMEG _2776	MSMEG 2775	R.SYAPTIGGLAEHLAALR.L	3	12.4	14.1	1.2	1.0	13.0	9.1	2.8	2.6

MSMEG _2780	MSMEG 2780	K.AAGIGLDIVPDVGPVIDNPIR.T	2	50.0	54.4	51.1	44.7	16.2	24.8	85.2	88.5
MSMEG _2782	MSMEG 2782	R.ADVIDEAATFLK.Q	2	168.2	158.0	91.6	86.4	79.1	73.3	72.6	64.5
		R.ISDPVWSSVALHR.P	3	76.7	73.4	20.2	23.5	35.1	34.7	5.6	8.8
		R.ISDPVWSSVALHRPAEFNK.S	4	75.8	81.2	27.3	23.0	57.1	56.8	35.6	35.0
		R.ISVENLIAK.L	2	47.9	35.0	11.2	10.7	19.5	22.5	16.6	19.5
MSMEG _2784	MSMEG 2785	K.LSLTDDEWR.E	2	17.7	11.5	45.6	37.0	14.7	18.5	55.2	47.4
		R.YC#INSISLR.L	2	10.4	7.3	3.9	1.4	19.0	20.1	4.5	8.0
MSMEG _2787	MSMEG 2788	R.IENYSGVQLSVPQR.Q	2	54.6	61.5	62.9	58.4	49.4	55.4	72.3	73.7
MSMEG _2788	MSMEG 2789	R.GVSIAATSNTLPEQLGEGR.F	2	44.0	54.5	149.5	157.3	40.4	50.8	136.7	127.6
MSMEG _2789	MSMEG 2790	R.LGAGIENDYVMVRPV	2	16.4	0.0	12.0	0.0	7.6	8.6	9.0	14.1
MSMEG _2800	MSMEG 2802	K.LTGSAPEHVIDVVDLGAGLLGWGDPK.V	3	90.8	98.0	101.0	112.8	82.3	103.0	108.8	116.5
MSMEG _2839	MSMEG 2841	K.LMISASVDAR.I	2	10.8	10.5	253.5	241.6	10.0	11.7	155.5	214.2
		K.SIGVGQYQHDVTPGSLAR.S	2	32.7	26.9	38.0	38.6	29.5	37.2	28.9	55.1
		K.SIGVGQYQHDVTPGSLAR.S	3	158.6	144.1	31.6	35.8	151.8	165.2	30.4	41.7
		R.AGVTLAELIGDTR.T	2	84.3	78.5	9.6	11.5	118.7	89.8	11.8	12.6
		R.ELPDLDVTVR.G	2	63.1	51.6	19.8	34.9	79.0	68.8	28.0	24.9
		R.IRDGEDPLDASGVHPESYPVVR.R	3	64.6	61.9	10.1	15.7	63.2	85.2	10.9	13.6
		R.LLADPNLVPEEVAGEFLNENVADAAAALEGAR.H	3	40.2	70.3	4.5	11.2	21.6	74.6	1.3	13.2
		R.PAFSTATFAAGVEK.V	2	146.4	137.6	53.7	80.6	222.1	210.0	61.5	69.0
		R.RLQDPLAELVK.I	2	16.6	12.1	142.5	145.2	34.0	24.4	74.0	90.7
		R.SLDAVVEDAVNAVGVDLNTASVPLLAR.V	3	43.5	65.7	6.5	12.8	32.9	68.9	4.5	16.9
		R.TTLGLDPGFR.T	2	11.5	10.7	1.9	3.4	8.6	10.5	2.3	3.2
		R.VSGVTESLAEAIVAHR.E	3	89.7	76.8	8.4	7.5	122.5	92.0	13.1	7.1

MSMEG _2897	MSMEG 2903	K.ATAVSVLEAAGHTVR.E	2	5.5	8.5	8.9	10.6	2.8	2.9	9.1	9.0
		K.ATAVSVLEAAGHTVR.E	3	13.6	12.1	6.1	7.0	10.7	8.6	13.0	10.3
MSMEG _2900	MSMEG 2908	R.AAVVFADALGLDR.V	2	17.6	14.0	11.4	13.0	16.0	14.0	3.4	3.8
MSMEG _2931	MSMEG 2941	K.DYGLDDYYLELSTK.D	2	74.1	77.0	208.3	219.5	60.2	52.1	215.6	193.4
		K.HLEAGYEFVNTPHITK.E	3	131.0	122.7	100.4	97.4	101.2	129.9	88.1	135.5
		K.VPFMLLAGDKDAEAGAVSFR.F	3	53.7	60.2	35.5	27.2	52.1	38.9	69.5	37.0
		K.YVGSDEIWEEATETLR.E	2	170.3	165.6	154.2	141.4	135.4	126.6	174.0	161.8
		R.EVAEASGLDLVPDPGGAAFYGPK.I	2	118.9	123.4	87.1	93.4	88.5	87.2	106.5	104.5
		R.IYGTAWESQEALDR.H	2	211.2	202.1	186.9	195.7	151.4	168.4	163.2	193.2
		R.LFEFGSVYR.Y	2	109.1	97.3	90.0	93.4	103.4	74.1	130.8	108.1
		R.LLEFVLSLLK.D	2	19.0	23.3	11.9	15.8	22.3	18.5	20.6	18.6
MSMEG _2940	MSMEG 2951	R.NGGNMADPGSVAYLFSR.K	2	143.0	150.5	21.5	24.3	114.1	104.6	14.6	14.0
		R.VGGGDPGGNPTLYDAIQK.A	2	238.9	223.8	119.5	102.2	205.6	227.0	60.7	79.6
MSMEG _2941	MSMEG 2952	R.TTIVRPGGLTNDAGTGLVR.I	3	48.2	57.2	55.2	66.8	43.4	48.3	64.8	74.5
		R.VLVAVLDAPQTAGR.T	2	84.6	93.5	105.0	124.6	74.5	75.2	127.2	122.6
MSMEG _2942	MSMEG 2953	R.HFYGGVLGLPQGR.S	2	27.4	0.0	152.3	0.0	16.6	14.8	156.3	137.4
MSMEG _2956	MSMEG 2165	K.IGPDKDIPHVTADVIVK.Y	3	25.8	25.0	464.4	403.8	0.4	0.2	423.6	342.7
		K.IGPDKDIPHVTADVIVK.Y	4	27.5	27.6	9.2	8.1	0.2	0.2	9.2	7.4
		K.LEAVFGDSPLVR.Q	2	127.3	112.2	23.9	20.4	15.1	16.2	33.5	33.0
		K.RPEITAEMFDEDGYYR.T	3	31.0	30.5	2.8	2.4	15.3	11.6	2.8	2.6
		K.VIASSVDFLADAVALVESGPAPSR.L	3	21.1	23.6	0.7	0.9	2.5	2.7	2.4	1.7
		R.ALAADHLEVIAGDKGEADLGLDHDTWQR.L	4	32.3	22.7	2.9	2.1	2.6	2.2	1.0	1.6
		R.FVSALTGSAPISAEMK.S	2	133.2	140.2	220.6	230.5	4.3	4.4	152.2	152.6

		R.IDHLFETDPQFAAARPDE AISAAAADPELR.L	4	45.9	47.6	10.9	11.2	1.3	1.7	4.3	5.9
		R.IQAVTNAWHNHPVNAGD R.V	3	39.1	23.2	3.4	3.9	0.0	0.1	2.5	3.6
		R.IWDMLFQEYQSR.L	2	35.1	35.8	1.6	1.2	3.6	3.9	1.3	1.3
		R.LDATFDTGDATLLEHYR.A	3	139.9	125.5	0.4	0.3	29.5	43.2	0.4	0.4
		R.LEQLYTDLAEGQANELR.E	2	73.1	70.8	0.1	0.2	1.1	1.5	0.6	0.0
		R.LEQLYTDLAEGQANELR.E	3	20.7	22.2	1.7	2.3	0.1	0.0	1.4	2.6
		R.LVVFDYSHEVDDQR.E	2	19.5	17.5	0.2	0.3	0.2	0.1	0.4	0.8
		R.RIDHLFETDPQFAAARPD EAISAAAADPELR.L	4	43.7	39.2	1.3	2.0	2.5	5.9	1.7	2.6
		R.SDLSTFLEDLALVR.P	2	2.8	2.8	0.6	0.4	0.2	0.5	0.1	0.3
		R.SDLSTFLEDLALVRPTQLN FVPR.I	3	11.5	12.4	1.9	3.2	0.4	0.6	2.5	1.9
		R.SYLLAVVVPTEEALSR.W	2	97.8	94.4	4.6	1.9	1.0	1.3	0.6	1.7
		R.VDDYATWLSR.F	2	37.5	35.0	0.5	0.9	0.3	0.6	0.6	1.2
MSMEG _2960	MSMEG 2972	R.DRIDSGLAQDDDELSEHT NDTDGYKND	4	37.6	54.7	29.7	41.9	37.3	52.7	26.3	40.5
		R.IDSGLAQDDDELSEHTND TDGYK.N	3	40.0	51.0	33.3	42.4	16.8	19.9	11.0	18.7
		R.IDSGLAQDDDELSEHTND TDGYKND	3	93.3	119.6	92.4	121.6	70.9	95.7	68.9	93.6
MSMEG _2961	MSMEG 2973	R.DALNQAQQIISAR.V	2	96.4	122.5	63.0	83.1	105.4	123.2	65.6	75.4
MSMEG _2964	MSMEG 2976	R.GFLLGAAVATR.L	2	11.3	12.7	6.1	11.0	4.9	4.9	4.1	6.9
		R.NVVIIDDVLATGGTLAAAA R.L	3	17.8	14.7	5.8	4.9	8.7	9.1	4.9	3.2
		R.VVTDALADIASGADLVAGL DAR.G	3	26.0	16.9	9.6	4.7	12.7	13.3	4.5	4.9
MSMEG _2974	MSMEG 2987	K.IAAGGVEGGGPDGKPALD VELK.S	3	144.6	195.2	140.2	218.1	110.1	112.6	94.5	102.9

MSMEG _2975	MSMEG 2988	K.GVPDYLPPDSAQFVAVR.D	2	117.7	114.4	85.6	86.3	81.6	80.2	119.6	101.6
		R.AGYGDIELPIFEDTALFAR.G	2	183.9	191.3	191.7	181.1	138.9	139.8	211.0	246.7
		R.ELLQEFLFK.L	2	53.2	48.9	35.2	30.0	46.8	35.6	49.0	33.5
MSMEG _2978	MSMEG 2990	K.ALIAEQLDLFPALK.E	2	0.1	0.0	26.6	30.3	0.0	0.0	8.9	19.5
MSMEG _2982	MSMEG 2994	K.AIQDNADGVSFDAPEGK.V	2	25.1	15.9	218.2	167.5	16.8	22.6	243.7	279.7
		K.ASETDAANAESC*VDTSGPTIK.V	2	0.0	0.0	68.6	62.0	1.7	3.6	76.7	90.3
		K.GEDYTPLGSTDFSTIVNK.V	2	3.1	7.6	716.7	795.2	16.2	2.6	815.6	844.5
		K.LAVEEINADGGVLGK.Q	2	94.6	129.6	1049.7	970.0	26.9	30.6	1136.1	1072.7
		K.QIELIGEDGASEPTVFAEK.A	2	250.6	300.9	77.3	90.7	175.7	199.7	92.2	105.4
		K.SLYLVGSDYVFPQTANR.I	2	31.5	21.4	656.6	702.1	58.2	68.1	615.0	660.3
		K.VGSLNSLSGTMAISEVTVR.D	2	9.7	13.2	248.4	292.3	1.6	1.2	159.3	171.2
		R.IGEIRPDGLIYTVWESK.G	2	12.3	21.5	237.5	266.6	8.8	10.3	248.7	283.7
		R.IGEIRPDGLIYTVWESK.G	3	11.1	14.2	373.5	331.3	7.4	9.4	456.2	429.2
MSMEG _2983	MSMEG 2995	K.AAYDADPYAYLQK.A	2	16.9	18.9	7.8	8.4	17.0	17.4	12.3	8.5
MSMEG _3003	MSMEG 3017	K.AVWATIGYDLPLPLPR.I	2	85.5	77.3	1082.3	937.1	94.1	90.9	513.3	605.8
		K.GLAYVLVGEDGTLGGPVAK.N	2	252.3	260.6	573.6	541.7	203.9	198.6	395.1	392.3
		K.SGGGVDPLTDAPAPITPQQR.K	2	131.9	134.8	215.3	234.5	115.1	144.3	184.1	303.1
		R.ITALLAGVDSIR.E	2	131.2	119.6	138.9	150.4	162.3	119.9	164.3	135.5
		R.LQPGSFYALPQSPQLFK.Q	2	223.5	222.0	200.8	186.6	222.1	198.7	192.1	199.0
		R.THAAGSLRPADAGQTVTLAGWVAR.R	3	772.7	824.2	82.0	99.0	330.6	425.4	70.8	105.5

		R.VFQAPYVGAVVMPGGAS QPR.R	3	29.6	42.8	67.3	90.7	19.1	30.6	51.8	83.1
MSMEG _3004	MSMEG 3018	K.LYSDTAALDDFNSK.V	2	22.5	24.9	13.1	14.8	12.5	16.2	7.0	8.8
MSMEG _3013	MSMEG 3027	R.LSDLVEAGLLER.R	2	10.5	10.6	6.9	9.0	5.0	9.5	9.9	9.7
MSMEG _3016	MSMEG 3030	R.DIADVAADLR.A	2	0.7	1.1	11.1	9.2	1.1	2.9	9.2	10.2
		R.GAIEPWNVLGEESTAGGT AR.Y	2	14.1	11.5	109.0	88.0	8.8	9.2	104.6	89.9
		R.HLLTDITGNTHR.A	3	0.4	0.0	4.4	0.8	0.4	0.1	2.2	1.4
		R.IVLLDGDSPAGLR.L	2	1.7	2.2	31.0	28.6	3.2	3.8	29.2	35.2
		R.LGLLELR.G	2	0.0	0.0	3.9	2.9	0.1	0.1	0.8	2.6
		R.LPAGDPVGADEDLAEDGA DAR.A	2	0.1	0.5	10.7	12.2	0.0	0.8	7.7	10.4
		R.LTATDAPQPWVLDR.A	2	4.0	3.8	38.4	39.3	3.4	6.0	39.5	43.5
		R.SLVAWFWDEPLR.A	2	0.3	0.1	6.4	6.3	0.9	1.0	9.3	7.9
		R.SYDTSPVNAVEAESR.R	2	14.3	14.4	20.2	20.0	27.2	28.6	16.4	20.2
		R.TATVVDADGAPTTALVAEI R.D	2	0.3	0.7	15.8	18.8	0.7	0.8	11.9	13.8
		R.VTLPYTDAAWASIVDLGG R.V	2	6.2	7.0	14.9	15.6	8.2	7.9	10.9	15.1
MSMEG _3019	MSMEG 3033	R.AEDPEYGLAFDLLSSEFD K.V	2	26.4	31.5	62.3	67.7	24.1	22.4	59.1	54.9
MSMEG _3025	MSMEG 3039	K.EDFEILGPLPR.K	2	26.5	25.2	8.0	13.4	16.6	13.7	14.5	10.4
		K.GAAGIDAALAALR.A	2	99.1	88.0	29.4	25.9	126.4	90.9	56.7	42.2
		K.LLGVEQPVMGDLIATVR.D	2	240.4	237.7	217.6	227.2	254.6	226.5	247.6	243.2
		K.LLGVEQPVMGDLIATVR.D	3	19.4	21.9	1.5	6.4	21.4	17.8	2.2	4.8
		K.RVPVVSHDGLEADRVELIL DR.T	4	65.5	63.8	29.4	37.3	65.8	86.7	30.6	61.6
		R.AAAVNAVAGAETVGK.V	2	7.4	4.6	8.1	7.0	6.3	5.9	8.9	10.1
		R.ALMAGLASSLK.V	2	56.0	33.0	16.1	8.9	68.2	23.8	37.2	5.5
		R.FDFNWQGPLSDDQR.T	2	124.5	131.3	41.9	50.4	127.8	133.7	62.0	66.9

		R.GEGTSKEDFEILGPLPR.K	2	38.7	43.6	14.0	9.0	37.0	46.2	7.7	12.6
		R.GEGTSKEDFEILGPLPR.K	3	66.7	62.5	21.6	23.2	86.3	68.7	45.4	32.7
		R.IAVAEETAFNR.T	2	29.5	25.8	14.8	9.6	24.7	30.7	13.6	18.1
		R.TAAIIIADGVSPGNEGR.G	2	648.9	600.0	357.1	315.7	1044.3	1080.7	374.0	341.9
		R.TPDIDEVGITTR.H	2	33.4	27.4	12.9	15.0	33.0	22.0	10.8	7.4
		R.TQIEEVTNQAVEADYEVH TFVTELEK.A	3	341.6	377.7	205.5	220.7	245.7	315.2	164.5	223.4
		R.VAC#LLQGVDNVYETDLL RPVIDK.V	3	159.0	223.7	121.6	155.3	162.7	179.8	106.6	142.7
		R.VAC*LLQGVDNVYETDLL RPVIDK.V	3	159.0	223.7	121.6	155.3	159.9	179.8	102.5	142.7
MSMEG _3026	MSMEG 3040	R.ASASAHDAVDVAEQLAR. R	3	9.3	8.7	19.7	18.9	7.0	6.3	14.7	18.4
MSMEG _3033	MSMEG 3046	K.VAVLHQPVLNQTAEAIR.S	3	107.5	104.5	183.4	229.1	78.6	95.4	125.4	136.6
		R.GVDIVHVPTTLLGMVDAA VGGK.T	3	54.4	55.5	58.7	56.6	26.2	21.1	36.0	33.2
MSMEG _3035	MSMEG 3049	R.DGSDFVFMDSEDFEQHP LPESLVGR.L	3	204.7	223.0	179.4	185.6	181.7	201.2	149.5	172.0
MSMEG _3045	MSMEG 3059	R.LPALPDSVGPALVAPTK.G	2	18.3	18.2	25.7	26.2	20.0	31.5	11.9	19.5
MSMEG _3046	MSMEG 3060	K.AGVFSGDALADVDALLER. V	2	351.0	424.0	212.8	211.9	349.5	421.2	270.4	277.2
		R.VAITAQNHGFALEGEAGE K.F	3	102.0	123.8	115.0	142.7	42.8	72.7	53.9	72.2
MSMEG _3047	MSMEG 3061	K.AGEVNMVINTPYGNSGPR .V	2	47.0	45.1	52.3	51.5	37.7	32.8	46.5	40.6
		K.ETPAC#FEPTLDYVVVK.A	2	56.1	48.9	111.5	98.7	56.3	48.9	124.0	107.1
		K.ETPAC*FEPTLDYVVVK.A	2	55.8	48.9	110.6	98.7	56.3	48.9	123.8	107.1
		K.FPGADATLTTTMK.S	2	92.6	96.7	101.5	108.2	66.1	70.3	85.9	97.7
		K.HYEDPSAADPRPSAVEVI K.A	3	76.3	69.6	93.1	77.6	66.4	70.8	96.4	86.8

K.LAIGYTLDEILNDITK.E	2	45.5	52.5	49.2	50.2	34.9	42.7	50.5	57.4
K.LAIGYTLDEILNDITK.E	3	5.7	7.1	6.4	7.4	4.5	5.4	6.0	6.1
K.RLEDAGVPIVGTSPK.A	2	22.2	18.9	26.9	20.5	21.8	16.6	32.5	21.4
K.RLEDAGVPIVGTSPK.A	3	45.2	40.5	53.6	46.0	25.4	31.2	38.6	46.8
K.SQTAAYGSLPTEGTVFVSVANR.D	2	18.6	17.2	26.4	24.9	13.8	13.6	18.0	23.6
K.SQTAAYGSLPTEGTVFVSVANR.D	3	80.5	87.8	94.5	100.2	45.6	66.1	64.9	82.4
K.STGEVMGIDHDFGTAFAK.S	2	12.2	10.4	8.2	7.9	10.9	7.8	6.9	8.6
K.TPYHYSSYELDPAAETEVAPQTEKPK.V	4	185.8	214.4	219.0	237.7	112.4	129.5	167.6	185.9
R.ADGAQIDSLLGPEMK.S	2	36.6	33.1	47.7	46.8	24.7	24.9	36.4	39.3
R.AELVEAPVLDADLLR.R	2	201.5	189.0	377.1	375.6	192.9	181.9	431.1	400.4
R.AGFWTAPDPDTTVEQLLTNLR.T	2	34.3	32.2	45.3	40.2	15.5	25.2	23.8	39.8
R.AGFWTAPDPDTTVEQLLTNLR.T	3	26.6	33.9	34.3	46.1	13.5	26.0	17.1	39.0
R.DLGIAILR.E	2	38.7	33.4	40.6	34.0	28.7	27.9	38.1	36.4
R.ETVAELGLPVVVR.P	2	16.7	17.4	25.1	29.3	12.4	12.9	25.7	27.5
R.EVGVDTGGC#NIQFAVNPR.D	2	38.4	39.0	49.2	47.9	41.4	38.9	57.9	54.1
R.EVGVDTGGC*NIQFAVNPR.D	2	38.4	39.0	49.2	47.9	41.4	38.9	57.9	54.1
R.GDIGVMSLQELHSELGSR.R	2	23.3	19.5	19.0	12.9	19.2	14.2	20.6	11.8
R.GDIGVMSLQELHSELGSR.R	3	50.1	49.2	55.6	48.7	40.4	35.9	56.5	51.6
R.GHFGEVLTAAGLPAPK.Y	2	31.5	26.6	43.9	39.5	31.8	25.6	51.0	45.1
R.GHFGEVLTAAGLPAPK.Y	3	78.4	72.1	70.3	63.4	61.1	59.7	64.5	62.5
R.GMEIVYDEETLR.G	2	68.4	73.7	95.2	94.4	45.2	49.9	80.5	80.1
R.IAADIGYPVLVR.P	2	61.7	58.2	231.0	198.7	36.1	36.3	73.4	69.0
R.IYDMELALR.L	2	47.9	41.9	63.1	51.7	35.3	34.9	55.6	52.5
R.KHYEDPSAADPRPSAVEVIK.A	3	9.9	6.5	5.9	3.9	10.2	7.2	12.8	7.1

		R.LEDAGVPIVGTSPK.A	2	19.8	20.8	21.7	20.7	11.3	13.9	24.0	18.5
		R.LIVIEMNPR.V	2	31.6	35.6	87.2	97.9	22.1	25.7	90.9	92.3
		R.QISALRPELAGEAGVR.A	3	9.5	10.0	47.1	40.1	10.6	10.4	19.7	16.1
		R.VMLGATITQLR.D	2	45.1	39.6	81.4	80.3	23.7	24.2	86.7	95.4
		R.YGVELIGADFEAIQR.G	2	83.9	66.0	126.5	103.0	86.1	69.3	157.9	123.9
MSMEG_3050	MSMEG 3063	K.AQEIM#TELEIAPTR.R	2	114.1	127.0	91.1	102.6	103.5	102.9	84.5	82.9
		K.AQEIM*TELEIAPTR.R	2	112.2	125.0	91.1	102.6	101.3	100.9	84.5	82.9
		K.QVLTDAETDEVLGK.M	2	147.0	152.7	145.8	149.6	158.8	162.0	169.1	157.4
		K.VSALLEALPK.V	2	311.2	303.5	260.4	252.7	408.1	392.0	352.4	320.7
MSMEG_3054	MSMEG 3065	R.LPEAEEITTLAQLLLER.A	2	75.3	76.7	107.1	82.4	65.4	69.1	94.4	113.3
MSMEG_3055	MSMEG 3066	K.AAPVGLFVETFGSETVDPAK.I	2	1623.9	1454.6	741.5	610.7	1386.7	1220.5	905.4	775.8
		K.AIGEVFDLRPAAIVR.D	2	698.0	680.6	355.9	367.0	615.5	526.7	402.6	371.5
		K.AIGEVFDLRPAAIVR.D	3	1059.1	925.7	440.2	383.7	967.2	846.4	606.6	522.3
		K.FVLGGPM*GDAGLTGR.K	2	210.9	195.0	491.7	576.0	264.2	277.4	501.2	458.1
		K.FVLGGPMGDAGLTGR.K	2	1361.3	1376.9	709.7	727.8	715.7	675.1	501.2	458.1
		K.GFDGASC#GVNVAIGAQSPDIAQGVDTAHETR.V	3	384.0	426.3	274.4	306.8	349.6	349.6	301.9	315.3
		K.IC*DAISDSVLDALLEQDPK.S	2	301.5	324.6	151.9	165.7	165.3	168.9	108.4	100.5
		K.IC*DAISDSVLDALLEQDPK.S	3	227.0	219.6	77.2	81.0	137.0	124.2	74.1	62.6
		K.IIVDTYGGWAR.H	2	393.0	259.6	146.7	106.1	292.4	277.7	164.3	159.8
		K.NVVAAGLAER.V	2	9.8	6.2	2.5	3.1	9.2	10.7	1.1	2.2
		K.TQVTIQYDGTTPVR.L	2	1793.8	1687.2	1161.4	1103.5	1465.9	1357.7	1368.7	1348.9
		K.VVNTVLADLGHETLDTSDYR.L	2	472.1	422.1	225.6	205.4	309.5	310.6	182.0	188.4
		K.VVNTVLADLGHETLDTSDYR.L	3	3394.3	3103.3	1481.4	1303.9	2800.1	2650.4	1737.6	1642.3
		R.DLDLLRPIYAPTAAYGHFGR.T	3	1117.1	1124.1	498.0	492.1	961.6	874.7	591.7	578.4
		R.DLDLLRPIYAPTAAYGHFGR.T	4	562.3	535.6	253.0	219.8	485.5	477.8	310.0	310.4

		R.EKVVNTVLADLGHETLDTSDYR.L	3	291.6	226.6	265.3	212.9	391.4	364.3	342.7	309.2
		R.ILDIGYDSSTK.G	2	20.9	19.9	196.8	238.8	12.7	8.7	265.8	256.5
		R.KIIVDTYGGWAR.H	3	32.6	12.5	0.7	0.0	61.5	49.1	20.8	8.4
		R.LDTVVLSTQHADGIDLEGTLTPDIR.E	3	683.6	528.8	458.1	384.0	603.1	530.5	493.7	456.4
		R.TDIELPWEQTNK.V	2	716.4	671.3	383.6	378.1	558.9	508.1	280.2	293.9
		R.TDIELPWEQTNKVDDLK.S	3	305.9	332.9	38.6	66.9	134.5	123.3	55.3	96.3
		R.VEVQVAYAIGK.A	2	274.9	304.4	109.9	149.7	263.7	254.0	105.1	133.8
MSMEG_3058	MSMEG3069	K.DAGLDFDIR.V	2	1.9	1.4	2.8	1.5	21.8	17.7	3.3	1.6
		K.HLVTLPTDGPVNVHTIGNPK.N	3	126.2	132.3	181.5	190.9	430.3	374.8	197.4	174.3
		K.KLTVAATQVPHAEILEFIK.N	3	16.5	13.0	19.7	14.5	46.3	54.1	18.0	27.8
		K.LTVAATQVPHAEILEFIK.N	3	42.1	45.7	75.4	74.3	96.6	94.5	49.5	51.4
MSMEG_3066	MSMEG3077	K.AGLSVKPGTPLEPYLEILR.E	3	76.2	78.3	112.7	120.0	66.1	66.3	104.9	114.6
MSMEG_3070	MSMEG3080	K.LDGDLTNAPAVAAEGTADLIAFGQK.I	2	96.6	106.3	127.0	123.4	58.0	65.5	106.9	98.1
		K.LDGDLTNAPAVAAEGTADLIAFGQK.I	3	463.0	495.2	546.6	566.9	317.2	333.4	501.7	488.1
		K.TQQSVHLLLTVQGK.V	2	52.5	35.4	53.4	30.1	29.8	25.2	35.0	23.7
		K.TQQSVHLLLTVQGK.V	3	111.1	97.0	60.1	48.8	79.6	73.2	39.1	41.3
MSMEG_3071	MSMEG3081	-.M*FTGIVEELGVLVDK.A	2	56.4	46.7	72.0	53.2	37.6	37.6	31.9	36.9
		R.LGGHIVQGHVDGTGSVISR.T	2	2.1	0.0	0.1	0.0	1.7	1.0	0.0	0.1
		R.LGGHIVQGHVDGTGSVISR.T	3	66.8	68.5	19.9	17.8	62.3	55.8	25.1	23.2
MSMEG_3072	MSMEG3082	K.HGTAYTVTVDAK.K	2	13.3	9.1	9.9	8.7	10.7	10.0	2.6	2.9
		K.LQAYQLQDAGEDTVDANLK.L	2	131.6	143.9	25.8	59.9	84.8	105.1	24.8	39.0
		K.SIYEDVEHVALVR.G	2	96.9	89.1	120.6	120.3	93.6	75.2	90.6	93.8

		R.ALANPGSVADDFTKPGHVVPLR.A	4	203.3	191.5	60.6	72.3	193.1	199.3	73.5	97.9
		R.VGLDGYGLHIIER.V	3	27.4	29.1	4.2	2.5	38.3	35.8	5.3	11.6
MSMEG_3073	MSMEG3083	K.GAQAAAAALSTAVTLR.E	2	68.3	85.4	46.2	68.3	36.2	34.8	34.0	32.8
		K.VAADAGIPDPTVVR.V	2	171.6	164.6	225.7	222.2	97.8	115.2	176.2	196.5
		R.AGLPGSTEDKGAQAAAAALSTAVTLR.E	3	63.2	70.6	68.1	72.9	75.7	82.3	92.0	95.0
		R.VLGAIEIPVVAQALAR.T	2	248.1	256.8	327.6	328.5	164.7	176.7	282.7	278.1
		R.VLGAIEIPVVAQALAR.T	3	10.8	0.0	7.8	0.0	4.0	5.2	2.3	5.2
		R.VSLDEATPVANGVLTTNTEEQAR.A	3	52.2	67.1	80.8	92.2	27.1	35.4	46.2	52.8
MSMEG_3081	MSMEG3090	R.FAGGLHIVAGR.V	2	11.9	11.7	6.2	8.8	11.9	17.3	18.2	23.3
		R.GLPAQVVGGSVGDAEAAWR.G	2	204.6	201.9	310.6	394.2	105.9	98.0	181.1	177.5
MSMEG_3084	MSMEG3093	K.AIGLVLPELK.G	2	1347.6	1229.3	1619.7	1477.6	1200.9	1082.5	1776.1	1594.1
		K.EGPAALPWGDLGVDVVVESTGIFTNAAK.A	2	73.7	75.4	78.1	88.0	15.0	45.3	14.8	57.5
		K.EGPAALPWGDLGVDVVVESTGIFTNAAK.A	3	399.5	412.2	468.2	488.9	145.7	276.0	203.9	399.0
		K.GLM#TTIHAYTQDQNLQDGPHK.D	3	278.0	210.2	284.1	222.0	206.1	200.1	221.9	228.5
		K.GLM#TTIHAYTQDQNLQDGPHK.D	4	269.5	217.1	227.4	163.3	216.9	219.8	183.1	175.3
		K.GLM*TTIHAYTQDQNLQDGPHK.D	3	495.7	437.6	457.8	402.9	469.3	459.7	490.5	498.7
		K.GLM*TTIHAYTQDQNLQDGPHK.D	4	269.5	217.1	227.4	163.3	216.9	219.8	183.1	175.3
		K.GLMTTIHAYTQDQNLQDGPHK.D	2	179.7	256.9	209.8	326.0	46.3	56.7	58.7	84.4
		K.GLMTTIHAYTQDQNLQDGPHK.D	3	2908.0	3013.2	3684.2	3802.3	1282.0	1261.2	1901.6	1875.7
		K.NTDIEIVAVNDLTDNATLAHLLK.F	2	125.2	153.5	175.4	206.6	77.3	89.3	107.6	155.4

		K.NTDIEIVAVNDLTDNATLAHLLK.F	3	1458.6	1391.2	2050.7	1897.8	1187.2	1122.3	1988.8	1968.8
		K.VLNDEFGIVK.G	2	88.9	71.4	202.3	190.8	90.2	94.7	179.6	195.7
		K.VVSWYDNEWGYSNR.L	2	1676.9	1810.9	2085.9	2252.5	1088.7	1240.0	1599.7	1953.9
		K.YYDAPIVSSDIVTDPHSSLYDAGLTK.V	2	459.8	578.6	565.8	654.8	281.6	335.4	430.3	493.5
		K.YYDAPIVSSDIVTDPHSSLYDAGLTK.V	3	5939.0	5492.0	6941.5	6339.5	4640.2	4891.5	7082.3	7082.5
		R.AAALNIVPTSTGAAK.A	2	160.2	119.2	275.6	251.3	137.7	131.7	273.4	288.2
		R.LADLVALVGK.S	2	853.7	591.8	1163.5	687.3	879.2	834.0	1464.8	1344.2
		R.LPQDVSLEGDDTIVIGDTK.I	2	587.8	590.9	745.5	808.9	485.6	464.1	740.4	773.2
		R.VPIPTGSVTDLTAELAK.S	2	5700.5	5740.0	7947.2	7867.8	4565.1	4691.8	7354.0	7803.8
		R.VPIPTGSVTDLTAELAK.S	3	567.5	558.9	704.6	707.5	357.8	456.8	530.6	712.2
MSMEG_3085	MSMEG 3094	K.ALAALVEGPDGSPGVFVSDGFGVVHR.K	3	1552.4	1365.1	1051.2	976.2	1293.8	1251.3	1190.3	1090.4
		K.FAADAEAETVAADR.I	2	336.9	334.2	320.6	305.5	235.6	223.6	230.8	227.1
		K.FAADAEAETVAADRIPDGK.M	3	311.1	341.6	260.0	340.7	419.2	370.1	460.5	413.7
		K.GAFSVVGGGDSAAAVR.R	2	310.5	301.7	360.3	378.4	287.3	258.3	320.0	346.0
		K.GVAEAIIGATGK.G	2	26.4	15.2	6.7	6.7	21.4	20.0	20.2	6.3
		K.LAVIENLATK.A	2	27.2	20.4	13.7	21.6	15.4	10.1	16.0	22.4
		K.LSLAPVAAALGEK.L	2	502.7	432.0	600.6	550.7	437.7	384.2	671.2	585.1
		K.TLDDLLAEGVQGR.G	2	300.1	271.3	384.0	366.6	288.7	269.9	523.9	442.0
		K.TVFWNGPMGVFEFPAFAAGTK.G	2	161.2	184.8	162.8	202.4	63.8	81.8	66.3	99.2
		K.VLQQLTSSTDRPYAVVLGGSK.V	2	280.3	317.1	269.1	313.7	233.2	213.8	234.0	238.3
		K.VLQQLTSSTDRPYAVVLGGSK.V	3	1360.8	1323.1	1112.5	1142.5	1027.9	1167.3	1045.6	1139.0
		R.AEGLTDGDVLLLENIR.F	2	360.9	357.2	329.8	337.9	413.8	369.5	430.7	384.4
		R.HVQLAGDVVGTDALAR.A	2	336.1	400.8	376.7	331.3	352.5	375.7	462.5	478.8

		R.LGLPEDGFSHISTGGGAS LEYLEGK.E	3	474.4	437.1	469.9	455.2	389.2	357.1	448.8	426.1
		R.LLDEYADVIHLPVDIVVAD K.F	3	330.0	339.2	306.1	321.2	253.6	212.7	298.7	258.2
		R.RLLDEYADVIHLPVDIVVA DK.F	3	82.5	82.0	764.9	494.7	56.4	58.3	883.5	770.4
		R.SDLNVPLDDDGNITDPGR. V	2	580.8	647.0	497.1	545.2	523.3	534.8	533.5	457.6
MSMEG _3086	MSMEG 3095	K.HGLTPIVC#IGEQLEVR.E	3	35.3	36.9	12.1	9.6	27.3	19.3	14.0	12.5
		K.IAFALPDKYFDK.V	3	25.6	23.7	33.0	33.8	30.6	28.4	42.9	40.7
		K.LGC#TFVIVGHSER.R	3	56.6	44.2	29.1	22.4	61.5	49.2	37.3	28.6
		K.LGC*TFVIVGHSER.R	2	26.9	27.6	43.6	27.2	29.6	28.0	49.7	47.9
		K.M#NLNHFEAIALVQK.I	3	52.1	56.1	37.1	38.8	36.4	41.5	30.2	31.8
		K.M*NLNHFEAIALVQK.I	2	73.2	83.3	142.9	145.2	73.3	74.3	133.4	117.6
		K.M*NLNHFEAIALVQK.I	3	52.1	56.1	37.1	38.8	36.4	41.5	30.2	31.8
		K.MNLNHFEAIALVQK.I	2	108.1	105.2	162.4	145.2	112.0	106.9	182.9	179.8
		K.MNLNHFEAIALVQK.I	3	177.1	180.3	210.3	209.0	179.4	169.1	223.8	207.4
		R.EAGNHVEFNVNSLR.G	2	110.1	76.0	70.3	46.5	106.0	95.7	88.5	77.0
		R.GELANLSSSEIAAGVR.V	2	635.5	725.3	1118.5	1190.4	693.6	690.1	1037.5	1027.6
		R.GELANLSSSEIAAGVR.V	3	49.7	43.5	63.7	51.8	47.5	52.1	43.6	44.4
		R.GSLAGLTPEQIGQTVIAYE PVWAIGTGR.V	2	24.8	39.0	23.3	34.7	12.4	28.7	15.4	32.7
		R.LTYGAQDLSQHDSGAYT GEISGAFLAK.L	3	626.2	643.4	611.2	620.6	646.5	614.2	748.9	712.5
MSMEG _3097	MSMEG 3107	R.AVHVAAGKPPQDSSLAAT YR.K	3	37.2	44.6	66.2	49.7	28.8	35.9	89.1	53.2
		R.FPYLEPLNHLQVELLR.R	3	14.1	20.6	60.3	54.0	15.1	19.1	102.1	60.3
		R.GGGPSYDAILAQPPGAVK .G	2	109.9	118.9	178.6	182.4	102.9	132.4	166.8	210.5
		R.LITGQDDLLADNAALAR.S	2	72.7	70.4	140.6	136.8	61.9	88.2	157.6	182.6
		R.LLGAILGDTVR.E	2	32.6	27.9	45.3	44.7	40.8	30.2	69.4	57.3
		R.YQTPAEFLADLDAVDGSL R.A	2	54.8	61.6	36.6	35.2	46.1	70.4	47.8	47.8
		R.YQTPAEFLADLDAVDGSL R.A	3	4.0	5.2	6.8	10.2	4.6	4.5	6.3	10.4

MSMEG _3099	MSMEG 3109	K.ADAVAAAVGGADPVDIPA AGAVGR.E	2	91.7	102.9	93.4	100.4	77.3	89.2	102.6	117.1
		R.GQATIVLTGGGTGIGLLK. R	2	91.6	81.9	985.2	982.1	70.4	63.8	1114.4	1072.8
		R.LVDAISSAIGER.G	2	125.0	98.4	125.3	99.8	110.1	104.8	141.2	143.8
MSMEG _3100	MSMEG 3110	R.VLTLVIAPHTEDLVEDSIEA ANFASR.E	3	126.3	137.6	214.9	209.4	66.8	81.2	78.9	101.4
MSMEG _3101	MSMEG 3111	K.LMPAIYDLANR.G	2	37.2	33.4	22.7	25.9	10.9	17.1	5.4	6.8
		K.VVGLLDEEGFAK.D	2	38.1	21.9	58.3	46.2	61.0	55.7	68.3	75.9
		R.FVQGTFDDDTAFTR.L	2	132.6	131.4	71.6	103.4	140.7	131.3	85.6	94.0
MSMEG _3102	MSMEG 3112	K.GTAYDAQVNELAAR.G	2	61.9	54.0	225.1	165.0	69.6	84.4	128.0	166.6
		K.IHSVASFFVSR.V	2	283.7	219.1	109.9	104.2	302.0	216.7	185.2	124.2
		K.LAAIGIDLPDVFR.V	2	572.6	547.8	350.1	333.2	498.8	446.6	347.8	304.3
		K.SWQELLDATQGQLDAAK. K	2	517.1	477.8	299.0	312.0	450.7	406.2	290.8	278.9
		K.SWQELLDATQGQLDAAK. K	3	73.3	72.8	20.8	26.9	53.0	66.0	13.6	17.9
		K.TILQAIELWK.I	2	321.9	266.4	188.0	142.0	236.7	218.8	154.3	143.2
		K.TLEAVADHGEITGNTIAGT AASSQETFDK.L	3	794.1	768.9	449.1	431.3	817.9	726.0	529.2	498.7
		R.LAHDTDKTILQAIELWK.I	3	24.1	29.8	12.8	12.4	77.0	83.2	59.3	58.8
		R.LAYAAYEEVFGSDR.F	2	475.5	434.3	385.5	387.9	496.4	474.0	429.5	444.6
		R.LQTGNLTELINTR.S	2	928.6	984.8	664.8	700.1	959.5	852.9	764.0	626.3
		R.LVMDAYLEGLEK.A	2	236.9	220.7	167.4	149.4	192.6	169.2	188.1	187.3
MSMEG _3103	MSMEG 3113	K.AHDEWQVSFDEWAEREP ER.K	3	78.7	91.0	53.9	80.0	33.7	49.8	23.6	45.2
		K.AHDEWQVSFDEWAEREP ERK.E	3	48.4	67.3	57.5	45.9	39.0	83.9	51.2	93.7
		K.AHDEWQVSFDEWAEREP ERK.E	4	54.5	51.8	57.2	55.4	71.1	42.5	71.2	57.3
		K.GANSFGPPSISTEDWNAT WYGR.T	2	236.9	264.4	186.8	226.3	153.2	243.9	125.9	245.0
		K.GANSFGPPSISTEDWNAT WYGR.T	3	180.0	222.1	114.4	168.7	195.7	171.0	80.5	141.2

K.GVEITTGPLGQGLASAVGMAMAAR.Y	2	129.4	122.4	108.5	112.0	107.3	111.9	104.5	121.7
K.GVEITTGPLGQGLASAVGMAMAAR.Y	3	193.9	224.6	186.6	198.3	186.6	154.1	211.6	166.0
K.ILGFDPEKNFEVR.D	2	44.1	46.9	113.3	117.1	74.9	52.8	218.7	193.5
K.ISIEHNTDIAFSEDVVAR.Y	3	268.1	238.7	220.1	202.6	241.6	204.5	240.4	193.9
K.LPEGWDADLPHWDLDAK.P	3	79.4	89.8	64.9	70.4	35.9	36.6	28.4	34.9
K.LPEGWDADLPHWDLDAKPVATR.A	3	389.5	405.8	343.7	353.4	416.8	407.5	386.9	388.8
K.LPEGWDADLPHWDLDAKPVATR.A	4	666.9	718.7	531.3	542.1	640.9	645.6	524.3	557.9
K.LPELWGGSADLAGSNNTTIK.G	2	649.3	637.2	641.4	604.7	606.9	579.7	622.0	633.1
K.LVGDTGEIVSIEHYGESADDK.T	2	138.7	152.4	108.8	120.6	113.6	100.3	89.2	89.4
K.LVGDTGEIVSIEHYGESADDK.T	3	2751.3	2937.0	2513.6	2660.0	1595.1	2008.6	1757.9	2030.8
K.M*NTGGVHGSALGADEVAATK.K	3	10.3	8.7	5.0	4.6	2.7	3.4	1.5	0.8
K.MNTGGVHGSALGADEVAATK.K	3	50.3	39.6	39.9	32.0	33.6	34.2	29.9	34.5
K.MNTGGVHGSALGADEVAATKK.I	3	4.1	2.8	3.4	2.4	5.7	5.1	4.6	5.1
K.NFEVRDEVIAHTR.E	3	28.0	17.9	7.7	10.5	36.1	34.1	25.9	25.2
R.AASGAVLSAVGPK.L	2	10.5	9.2	3.1	2.1	7.4	14.6	2.6	4.5
R.AYGGTFLQFSDYM#R.P	2	76.4	77.5	92.4	77.5	50.4	41.4	57.7	43.0
R.AYGGTFLQFSDYM*R.P	2	76.4	77.5	85.4	72.7	50.4	41.4	50.3	40.9
R.AYGGTFLQFSDYMR.P	2	549.2	522.1	544.2	504.5	494.8	382.0	554.3	412.9
R.AYGWHVQEVQGGENVVGIEQALEEAR.K	3	1493.8	1530.5	1338.6	1341.2	1158.1	1267.6	1104.5	1251.2
R.EFGFTPEAVAAAAER.S	2	1384.0	1353.7	1348.4	1351.8	1222.5	1100.5	1432.2	1225.1
R.EFGFTPEAVAAAAER.S	3	35.3	34.5	20.4	24.3	34.4	29.0	27.2	24.8
R.EHAM*GAILSGIVLHGPTR.A	3	54.8	50.7	20.8	29.1	37.0	40.4	19.4	21.9

		R.EHAMGAILSGIVLHGPTR.A	2	75.4	74.2	330.2	313.7	55.2	66.2	339.6	312.8
		R.EHAMGAILSGIVLHGPTR.A	3	356.6	337.6	431.3	415.7	237.8	264.8	355.6	373.0
		R.EHAMGAILSGIVLHGPTR.A	4	166.2	125.5	91.4	57.5	97.1	117.8	67.0	68.0
		R.GSSSGPVGMILTR.Q	2	111.4	69.9	55.1	30.1	141.2	44.6	96.7	20.1
		R.KVTDKPSFIALR.T	3	116.4	54.5	106.1	58.7	98.0	38.6	89.5	35.3
		R.QPIPVLEGTDPEGVAR.G	2	114.0	116.4	403.0	403.1	136.1	152.2	299.6	359.4
		R.TIIGYPAPNK.M	2	5.8	5.7	34.2	16.9	3.8	4.9	47.6	16.5
		R.VAVEAAVAQSWYK.L	2	909.0	845.6	1067.4	993.0	848.7	725.6	982.4	945.2
MSMEG_3106	MSMEG 3115	K.ADAIGVNFIDTYFR.S	2	370.5	364.0	424.2	408.0	173.2	165.1	291.5	277.3
		K.ITVGGTYPLAEASR.A	2	643.5	600.9	449.4	418.8	270.5	307.9	266.2	320.5
		R.AGELINAIADGSIK.I	2	453.1	464.3	574.4	562.7	222.2	224.4	457.7	491.7
		R.ELPFVVGTEVC#GTVAAIGNDVAALK.V	3	445.2	412.4	446.9	398.6	200.0	194.3	313.1	305.1
		R.ELPFVVGTEVC*GTVAAIGNDVAALK.V	3	443.7	412.4	443.1	398.4	200.0	194.3	311.5	302.4
		R.GTLALFGASSGPVPPFDPQR.L	2	598.0	622.1	655.5	683.4	266.9	290.9	483.9	520.9
		R.LNAAGSVFLTR.P	2	39.3	35.4	37.1	27.1	5.9	7.8	53.1	40.3
MSMEG_3122	MSMEG 3131	K.YGYGWADSDVAGASAQR.G	2	68.2	58.0	6.1	2.6	51.6	61.3	5.0	4.7
MSMEG_3124	MSMEG 3133	K.AAMDELEIDPAFGER.S	2	119.7	111.7	68.0	74.1	142.9	134.5	95.0	96.7
		K.IAILDETDSGLDVDALR.V	2	147.9	152.1	90.8	108.5	187.4	170.8	119.9	120.6
		R.HEILQLSLLKPK.I	3	34.1	29.8	11.4	12.6	37.7	30.2	11.9	13.7
MSMEG_3127	MSMEG 3136	R.INWVWNPPWGPDK.I	2	21.9	22.7	3.1	3.0	21.4	22.1	1.0	1.9
		R.TALVGAGLVDELR.I	2	38.9	36.8	34.8	31.7	42.9	43.6	41.8	43.7
MSMEG_3142	MSMEG 3149	K.NLSAVLDLVEASVR.Q	2	14.2	13.0	14.6	16.2	5.3	3.7	17.1	15.3
MSMEG_3143	MSMEG 3151	K.DHIEAIANWDPNAEPSIEIQFTPAR.V	3	253.6	264.5	431.7	384.2	115.3	148.2	419.6	352.6
		K.DIHNYVEQNHPTPETK.L	3	30.9	27.8	13.0	10.1	12.2	12.7	10.7	11.3

		K.LSGEIKPGVTATDVVLTVTDM#LR.R	3	39.1	43.1	26.8	26.8	18.7	14.5	23.4	15.6
		K.LSGEIKPGVTATDVVLTVTDM*LR.R	3	221.5	268.0	100.9	121.9	105.2	83.8	95.4	80.3
		K.LSGEIKPGVTATDVVLTVTDMLR.R	2	60.2	74.9	10.9	18.8	30.5	31.6	18.4	20.2
		K.LSGEIKPGVTATDVVLTVTDMLR.R	3	2424.2	3109.4	1182.8	1389.2	1215.2	1293.5	1259.4	1266.3
		K.LSGEIKPGVTATDVVLTVTDMLR.R	4	64.7	90.2	14.6	23.4	28.3	34.9	17.1	21.8
		K.PGVTATDVVLTVTDMLR.R	2	15.7	18.0	25.6	20.3	2.4	2.8	30.4	30.9
		K.VLAENLLR.T	2	34.9	28.8	8.7	15.7	17.7	11.8	7.2	8.2
		K.VVPPGTGIVHQVNIEYLAR.T	2	282.1	279.0	148.7	154.1	167.8	162.4	108.4	136.9
		R.ATLGNMSPEFGSTAAIFPIDEETINYLR.L	3	563.0	837.9	268.0	380.8	132.5	302.7	130.9	302.3
		R.DTLTVGDQSYEIYR.L	2	2094.4	2154.7	1201.7	1198.1	1046.6	1063.9	1188.2	1202.0
		R.DTLTVGDQSYEIYR.L	3	40.6	44.4	5.0	2.1	17.4	18.1	0.5	2.8
		R.KDIHNYVEQNHPTPETK.L	4	3.7	1.9	0.4	0.2	0.8	0.6	0.3	0.2
		R.TDEQLALVEAYAK.A	2	1993.2	1948.6	1020.1	1002.2	1069.2	1003.9	1004.9	1023.3
		R.TDEQLALVEAYAK.A	3	10.3	9.5	3.6	3.6	0.3	2.8	0.6	2.6
		R.VIMQDFTGVPC#IVDLATMR.E	3	141.2	190.0	58.0	78.4	69.7	75.1	60.2	69.2
		R.VIMQDFTGVPC*IVDLATMR.E	2	283.7	279.2	119.0	123.7	118.4	140.8	107.7	134.8
		R.VIMQDFTGVPC*IVDLATMR.E	3	162.0	213.6	75.5	101.9	77.3	86.3	71.5	87.0
		R.WGQGAFDDFK.V	2	516.7	384.9	180.7	143.1	324.0	230.4	255.5	183.7
MSMEG_3147	MSMEG 3155	R.DYVIPQDVVEVIPDVLR.H	2	325.2	238.5	262.3	171.6	234.1	186.3	246.5	220.1
		R.IQFTPDLVPTDIVGTR.I	2	763.6	727.3	768.4	729.3	635.3	575.5	795.7	815.5
		R.LQDVAANTFVHHALVDYVVR.I	3	280.1	253.0	203.6	163.6	204.5	210.5	282.8	264.0
		R.LQDVAANTFVHHALVDYVVR.I	4	99.8	63.6	31.1	24.4	100.1	93.8	59.7	61.5

MSMEG _3150	MSMEG 3158	K.AGVTANVVAPGYIDTEMTR.A	2	334.3	364.7	268.2	300.9	360.5	358.1	280.1	303.4
		K.EVEEHQGPVEVLVANAGISK.D	2	119.9	124.9	76.1	71.0	108.0	107.3	78.1	65.6
		K.EVEEHQGPVEVLVANAGISK.D	3	685.1	687.6	661.3	663.5	668.7	610.2	658.7	613.3
		R.FEEVINTNLTGAFR.C	2	343.6	358.2	277.7	262.5	313.1	271.9	246.4	209.9
		R.IQAGALDFIPAK.R	2	365.7	388.0	616.5	588.4	298.1	286.0	526.7	501.6
		R.IQAGALDFIPAKR.V	3	58.6	56.9	30.1	31.5	144.8	131.2	79.1	77.5
MSMEG _3151	MSMEG 3159	K.AVLPIMNPGGGIVGMDFDPTR.A	2	283.6	297.4	205.7	227.1	312.2	324.2	264.5	266.1
		K.PAPLLELDVQNEEHLSTLADR.I	3	707.3	714.1	534.8	543.1	678.7	607.5	547.6	463.3
		K.VAQEAGAELVLTGFDR.L	2	364.8	355.1	368.9	376.0	486.7	389.3	485.1	415.1
		R.AMPAYNWMTVAK.S	2	159.6	167.5	132.5	144.8	170.8	189.8	170.4	182.7
		R.APLGWNMKDPTPVAK.T	3	24.4	26.2	30.4	48.9	97.8	92.8	109.2	96.7
		R.LPKPAPLLELDVQNEEHLSTLADR.I	3	147.7	153.7	93.7	80.5	229.7	217.2	147.7	140.6
		R.LPKPAPLLELDVQNEEHLSTLADR.I	4	177.9	150.4	86.7	66.8	238.1	228.8	137.2	110.5
MSMEG _3155	MSMEG 3163	K.VWLVPSDFGTALQGFTK.M	2	70.4	59.6	185.6	225.5	93.8	89.7	152.7	140.8
		R.AAAYLQAQGQAK.A	2	5.4	4.7	6.8	5.5	5.5	5.2	4.3	4.9
MSMEG _3158	MSMEG 3166	K.INAVEVLSNLLTGLGA	2	4.8	0.0	141.7	0.0	0.0	0.0	0.0	0.0
		K.VVESFPALGADGGADGDVK.F	2	48.9	55.1	115.4	135.0	71.1	80.6	72.4	102.7
		R.DPWVNMLR.T	2	2.9	0.9	3.4	5.3	8.1	4.6	1.5	2.9
		R.ILVDGGLSVPDALR.Q	2	99.1	108.1	291.9	310.7	324.4	284.9	268.0	242.3
		R.LSIDFGADPLTAPLSGR.A	2	140.3	145.7	268.8	261.0	324.5	309.9	285.3	269.9
		R.VLLLPLGPLAEHNIR.A	3	41.2	40.0	126.7	113.1	93.2	64.5	112.7	81.3
		R.YGTEASAAVAAAR.A	2	5.5	5.1	11.6	8.5	21.4	20.4	12.3	12.3
MSMEG _3159	MSMEG 3167	K.AGLDAGLDIDK.F	2	4.6	2.0	14.5	11.4	16.8	17.9	13.8	15.3
		K.AGLDAGLDIDKFAPR.L	2	1.6	2.0	136.4	160.6	31.4	38.0	135.7	157.7

		K.AGLDAGLDIDKFAPR.L	3	3.2	5.7	5.8	5.0	76.8	77.4	17.4	24.0
		K.EVAEHGGMAQAISEGIPK.L	2	144.3	189.0	204.9	247.3	73.8	150.5	69.6	188.5
		K.GLSVAFDLATHR.G	2	4.4	4.7	60.0	56.8	43.2	32.9	47.9	48.4
		K.YQVDEDTEIEVLK.V	2	106.3	108.2	80.4	73.8	188.2	187.3	81.1	85.3
		R.AAAASGPAGEDGLGNNLLALAIDAAR.A	2	15.4	18.6	39.4	38.1	68.8	75.3	35.0	54.9
		R.AAAASGPAGEDGLGNNLLALAIDAAR.A	3	70.6	78.6	154.8	145.8	271.6	235.9	231.6	170.3
		R.AERDEAATQAALDELTR.A	2	518.2	510.2	777.9	739.8	339.7	316.6	459.2	413.6
		R.AERDEAATQAALDELTR.A	3	111.2	125.6	192.0	180.7	155.5	188.8	121.3	136.3
		R.GPYPTMYVNQPWTIR.Q	2	76.3	83.3	266.9	285.7	148.3	171.9	125.6	177.9
		R.IISDIFAYTSAK.M	2	90.0	92.2	154.4	165.2	304.9	263.0	195.9	190.9
		R.LLWSELVSEFEPK.S	2	107.6	128.2	230.8	266.6	387.7	371.0	328.8	306.4
		R.PIDPWGGSYYVEWLTHQLAER.A	3	58.4	70.5	78.0	106.8	83.5	140.9	49.0	82.6
		R.SFADVPLEGETPAAAATPEAR.D	2	89.3	97.6	286.7	248.4	192.4	237.9	238.5	223.1
		R.THSQTSGWSLTAQDVFNNVAR.T	3	72.8	103.1	142.4	153.6	209.7	172.8	151.0	81.4
		R.VQGDVGMAGVAIDSILDMR.Q	2	30.7	29.5	39.3	43.7	71.6	71.4	39.8	48.5
		R.VQGDVGMAGVAIDSILDMR.Q	3	8.7	14.7	5.7	5.8	32.6	25.9	8.5	6.4
MSMEG_3160	MSMEG 3168	K.STTIEALGMHLIEAGHR.V	2	3.8	3.3	1.6	2.4	5.4	4.4	2.7	1.8
MSMEG_3169	MSMEG 3176	K.AGEGVVNPDGTLTAGPVVLQPEEYTSK.L	3	55.7	66.3	30.0	39.8	25.5	48.3	15.4	30.5
		K.AVELSDDVPAYGR.F	2	89.3	90.1	91.2	98.1	76.4	79.5	87.8	85.9
		K.DPDLVAAMDQVR.E	2	15.3	19.7	10.6	13.7	8.7	14.7	8.2	16.5
		K.LTVAVQDPDSLRPFADLIADELNVK.A	3	33.7	43.7	104.3	115.6	24.3	33.8	34.4	91.6
		K.YTNEWQAYVTR.Q	2	46.2	45.6	39.1	36.8	35.5	37.2	28.1	33.8
		R.FGWDTHGLPAELEVQR.Q	3	33.2	29.8	30.0	28.7	23.3	27.7	23.7	26.6

		R.FWDEDPDAIDTLHTVLEV TC#R.L	3	14.1	19.2	5.9	10.6	14.4	13.3	6.2	7.9
		R.HETYEHSYPHC#WR.C	4	4.7	2.0	1.9	0.9	1.3	1.4	0.2	0.5
		R.IDVYGSLDELER.D	2	156.3	153.1	96.2	89.0	166.1	154.9	76.6	75.0
		R.LAAPLLPLATEVIWR.G	2	86.5	92.9	82.9	92.0	113.2	105.1	117.8	107.4
		R.LAAPLLPLATEVIWR.G	3	11.9	13.0	6.6	6.9	13.3	10.9	9.6	6.8
		R.M*VELNQQITWYPEHVK.D	3	15.8	20.6	11.7	14.7	7.2	11.1	2.1	6.5
		R.MVELNQQITWYPEHVK.D	3	15.8	20.6	11.7	14.7	14.6	18.2	16.0	19.1
		R.PFIDQLTRPNPDDPTGK.S	3	27.3	26.4	12.2	19.5	16.6	18.7	4.6	9.3
		R.YWGTPIPVWK.S	2	0.0	0.0	0.0	0.0	0.0	0.0	0.0	0.0
MSMEG _3178	MSMEG 3185	K.ALWDTILPFAGYAFNK.S	2	3.3	3.6	4.5	3.8	3.2	5.0	7.1	4.2
		R.GDTLGVFQLDGGPMR.D	2	27.5	25.6	14.8	14.9	18.0	19.5	23.1	20.6
		R.VAQVITFGTIK.T	2	15.7	14.9	25.4	23.4	11.7	12.3	42.7	41.7
MSMEG _3183	MSMEG 3190	R.ITALTAPLSAADIDEAAR.R	2	220.5	220.1	195.5	198.8	161.6	189.2	163.8	222.0
		R.LSEATGANVYLK.R	2	83.0	66.2	69.3	89.5	56.5	61.2	9.6	21.5
MSMEG 3192	MSMEG 3192	R.ADELFAELPVALLER.L	2	53.8	64.0	48.3	51.0	42.6	39.1	69.5	53.7
		R.LVSLNEVGGEPALFGVTT AEFHQR.A	3	123.1	117.7	127.6	127.5	71.2	80.2	136.0	124.4
MSMEG _3185	MSMEG 3193	R.FFSITSLAALR.Q	2	13.2	13.0	13.2	12.3	11.0	8.9	24.9	10.6
		R.IDHPDGLTDPAGYLAWLR .E	3	19.1	26.3	5.3	10.0	21.3	13.0	7.1	9.9
MSMEG _3204	MSMEG 3214	K.DNVFAVILAHGK.T	2	1.3	5.1	4.3	10.6	1.1	0.7	3.2	3.6
		K.DNVFAVILAHGK.T	3	1.4	0.3	4.5	1.2	1.1	1.2	5.1	3.9
		R.ILPAGSDDPSLPR.I	2	8.6	8.1	23.1	19.5	5.3	9.6	17.5	26.5
MSMEG _3205	MSMEG 3215	R.ELLSQALENLDADVR.A	2	40.8	33.9	30.6	28.2	53.1	41.8	57.2	48.0
MSMEG _3208	MSMEG 3218	R.VVVLDYGSGNLR.S	2	28.4	38.9	39.2	30.0	18.6	21.0	44.1	45.5

MSMEG _3209	MSMEG 3219	K.DGTLTGPNLDLLAAVAGR. T	2	107.8	110.8	57.6	61.6	97.0	89.4	62.1	61.1
		R.GLILLPAVDVVNGQAVR.L	2	66.6	80.3	81.7	86.4	83.6	86.1	80.6	94.6
		R.GWETDGGDLWEVLDR.L	2	22.0	22.9	11.8	12.7	14.7	18.0	5.7	9.9
		R.TDAPVIASGGVSSLDDLR. A	2	156.0	160.6	108.8	123.6	141.5	154.5	107.2	113.6
		R.VAVGLDAQTDGAGGFR.L	2	52.2	54.1	12.6	15.4	56.4	54.2	10.3	18.6
		R.VNIGTAALENPQWC#AR. A	2	50.9	60.2	41.5	40.0	63.9	66.1	38.6	48.9
MSMEG _3210	MSMEG 3220	K.LTALVATAAEILDAASVPF VAGHR.A	3	14.0	15.0	24.0	33.1	8.4	9.3	20.1	30.9
MSMEG _3211	MSMEG 3221	R.GTGIDAIEWATR.G	2	9.8	9.4	8.8	10.6	8.4	11.5	7.9	11.7
MSMEG _3215	MSMEG 3225	R.AVETVTTDHVTTAFAHPV TVTYDEGR.W	3	88.1	84.9	22.1	36.6	25.6	31.5	24.6	18.9
		R.LLLLDEPTTGLDVAAR.E	2	304.1	331.5	139.3	138.8	153.2	155.4	197.6	187.9
MSMEG _3219	MSMEG 3229	R.FNGSLDDLDAVR.A	2	18.6	14.4	4.7	5.6	25.5	34.7	2.4	8.7
		R.GALASIGDPAELAQAYQD GGAR.V	2	46.1	47.9	10.1	8.9	64.8	68.8	13.0	15.5
		R.SAVADLVTAGTHPSC#PK. P	2	25.9	31.8	15.4	23.8	21.1	22.0	10.2	10.3
		R.SAVADLVTAGTHPSC#PK PAR	3	277.0	306.7	9.8	14.3	340.9	325.8	10.7	16.1
		R.SAVADLVTAGTHPSC#PK PAR	4	19.0	21.5	11.1	12.2	52.4	53.5	12.8	17.8
MSMEG _3220	MSMEG 3230	K.INNVLGQALLAR.Q	2	35.6	28.6	110.6	109.4	31.1	36.9	168.9	171.4
		R.LVPEALMAVIEEVTAAYEK .C	2	10.7	14.6	14.6	22.0	2.3	7.6	4.3	10.7
		R.LVPEALMAVIEEVTAAYEK .C	3	9.1	10.2	12.8	13.6	6.8	10.3	9.0	13.0
MSMEG _3221	MSMEG 3231	R.EVSDIPVGVGLGVR.S	2	122.8	110.8	156.8	139.5	116.6	112.3	177.2	166.6

MSMEG _3222	MSMEG 3232	K.AADETEEGDEESLVDEAGK.E	2	10.8	9.3	9.5	8.4	4.7	7.0	3.3	4.4
		R.AEPAAPAAVATAPVEPEK.G	2	22.1	21.8	20.1	19.7	16.9	18.4	14.0	13.9
		R.LGNYFNQELYGR.P	2	13.7	13.1	16.8	22.6	6.7	6.5	8.3	20.1
MSMEG _3225	MSMEG 3235	K.AQLSAAEPYEEWLHAGLLDLK.T	3	21.7	27.6	46.6	49.3	9.3	12.1	65.0	62.6
		K.GMLTTMQLPQYFLDLR.D	2	8.6	7.2	19.5	18.3	6.7	5.9	20.0	21.0
		K.LVSSVGVGTVAAGVSK.A	2	34.7	33.8	84.1	88.4	16.5	14.3	115.7	103.9
		K.TGVRPPVPLEEVEPVESIVTR.F	3	76.2	80.7	164.0	152.2	39.7	47.3	216.4	209.1
		R.APIPSLLAVSAVHHHLVR.T	4	9.5	10.2	22.1	19.2	1.8	3.7	26.5	29.8
		R.EGELTGIEPAAAVR.N	2	37.2	37.4	88.5	99.1	21.1	21.9	110.0	112.7
		R.FGVTSDYLVNATDIQIK.M	2	27.6	27.9	62.5	58.1	13.6	20.3	78.9	80.9
		R.GGEGLAEALDDLR.R	2	7.1	4.9	9.7	9.8	3.1	2.9	17.7	15.7
		R.IVSDGLIALENLEHR.G	2	4.3	3.2	44.1	29.1	2.2	2.0	84.8	38.7
		R.IVSDGLIALENLEHR.G	3	16.8	14.4	39.5	38.8	6.1	7.4	56.9	55.0
		R.LEVGGEYAFR.R	2	11.0	11.5	7.5	7.1	10.9	14.0	6.4	9.2
		R.TLIQLAEGALEDAHPVR.L	3	16.7	18.6	40.2	42.4	9.8	8.9	60.2	47.6
		R.VC#HLDTC#PVGVATQNPELR.A	3	24.4	30.9	76.6	84.6	11.5	12.9	94.0	96.2
		R.VC*HLDTC*PVGVATQNPELR.A	3	24.4	30.9	76.0	84.4	11.5	12.9	93.6	94.5
		R.VILASEAGVLDVPSGEIVAK.G	2	27.3	24.6	70.6	62.5	11.4	10.4	105.1	92.9
		R.VMGPEENLLEPTAASC*R.Q	2	1.8	4.8	13.8	16.1	0.3	0.9	13.6	18.0
		R.YLAELGFR.S	2	7.5	7.1	22.0	17.2	1.7	2.8	24.8	21.8
		R.YTGSTLGTSLLSDWPR.R	2	20.1	18.5	38.4	36.2	6.9	10.2	56.0	51.6
MSMEG _3227	MSMEG 3237	K.ALVAFTQSGDTVR.R	2	63.5	53.3	320.8	262.6	133.3	87.3	400.5	330.9

		K.DIEDLEFAVR.L	2	129.4	115.0	159.3	166.0	176.6	166.0	182.1	160.1
		K.GVSLPGMNVSVPAMSEK.D	2	766.2	920.1	570.3	586.9	746.0	783.5	292.9	362.5
		K.IVC#TLGPATSTDETVR.A	2	20.5	20.3	29.7	26.8	32.7	35.6	40.2	48.7
		R.AASDVTGHAVGIMADLQGPK.I	2	99.1	106.3	134.6	170.8	72.6	69.7	71.9	93.9
		R.AEASDVANAVLDGADAVMLSGETSVGK.Y	3	423.2	423.6	623.8	611.9	373.9	319.4	460.7	382.1
		R.ALVEAGMDVAR.L	2	180.1	168.5	201.3	205.3	113.7	106.5	141.7	154.8
		R.GDLGVELPLEEVPLVQK.R	2	729.2	660.0	1166.2	1094.3	1047.4	932.3	1588.7	1422.1
		R.IIQAVEDNSVVVPPLTHVPR.T	2	110.0	125.5	249.6	333.7	119.6	127.4	296.7	310.6
		R.IIQAVEDNSVVVPPLTHVPR.T	3	1717.2	1576.3	3525.3	3703.4	1901.8	2221.3	4430.1	4188.3
		R.IIQAVEDNSVVVPPLTHVPR.T	4	40.1	39.0	28.0	36.6	51.1	53.6	42.4	44.5
		R.LGVDLVALSFVR.S	2	79.1	74.9	117.9	112.7	128.1	124.9	151.2	159.4
		R.SPADIELVHEVM#DR.V	3	1.8	0.8	6.9	3.7	5.2	6.3	10.0	11.4
		R.SPADIELVHEVMDR.V	2	272.6	292.7	332.2	365.3	210.1	205.2	223.9	232.3
		R.SPADIELVHEVMDR.V	3	145.8	131.5	161.3	144.2	131.3	131.0	138.5	128.7
MSMEG_3234	MSMEG 3244	R.KLGAALSTGSPSAPPVS	2	2.8	3.2	13.5	14.6	3.6	4.6	14.8	13.2
MSMEG_3235	MSMEG 3245	K.AGEVFDSAPYGWPVAK.G	2	1117.6	1220.2	850.8	880.2	1224.1	1250.6	737.4	780.9
		K.GSPLAQSLQQALQHLIDNGEYEK.I	3	390.4	409.2	817.8	813.4	516.8	564.0	605.3	610.3
		K.IIPSIQGGTFNVGMSSFTDTK.E	2	106.9	107.0	188.5	192.5	219.8	185.2	167.2	164.5
		K.KVAVQATTVQETDELPAR.S	3	39.9	31.9	51.0	43.7	59.4	70.3	35.2	48.3
		K.LIVGVNIPYAPNEFK.D	2	450.9	454.5	1241.2	1245.7	474.1	454.0	1277.5	1227.3
		K.LIVGVNIPYAPNEFKDPSGK.I	3	438.1	455.5	774.9	822.6	657.3	700.0	608.9	642.4
		K.VAVQATTVQETDELPAR.S	2	379.5	418.8	820.4	868.0	620.0	700.7	706.2	825.1

		K.VAVQATTVQETDELPAR.S	3	80.7	95.5	105.2	134.6	117.3	121.6	76.1	88.6
MSMEG _3244	MSMEG 3254	R.DPRRDGEHALSAGNPVG VVPR.A	2	17.8	21.4	64.4	61.4	21.6	39.7	60.3	47.2
MSMEG _3246	MSMEG 3257	R.FAELSALETEVATLSER.L	2	120.1	117.7	188.2	194.4	97.6	94.7	188.1	177.1
		R.FAELSALETEVATLSER.L	3	28.7	29.6	34.7	37.9	25.3	22.3	35.8	37.9
		R.IAPIVILTAFSQR.E	2	53.3	53.9	100.7	95.6	32.5	34.6	74.6	83.6
		R.LDLAEMLR.E	2	21.6	21.2	30.2	30.4	13.2	14.4	23.4	25.4
		R.VAEVVLETLDDTK.Q	2	175.1	200.3	280.2	350.7	162.6	165.2	277.2	287.7
		R.VLIAEDEALIR.L	2	97.4	100.2	175.5	163.6	131.7	99.9	151.1	177.0
MSMEG _3247	MSMEG 3258	K.ATGVAPQIVDEPFIIGVVG PAFSGETK.A	2	124.8	159.8	647.2	735.5	111.2	190.3	611.6	779.0
		K.ATGVAPQIVDEPFIIGVVG PAFSGETK.A	3	359.7	347.0	1516.6	1506.4	595.5	660.4	2064.5	2290.8
		K.DGGVEATFISADGTKDPE FVK.Q	3	133.4	149.6	224.1	242.3	316.0	359.0	457.7	427.3
		K.FGQEPGTYSTEGYDLGTI LLK.G	2	297.1	302.3	691.3	694.0	498.1	448.3	755.3	694.5
		K.GAAPDSVFYSGYYSEAAP FVQQLK.D	2	118.1	140.5	270.9	325.3	214.9	194.1	307.1	288.8
		K.GAAPDSVFYSGYYSEAAP FVQQLK.D	3	244.6	234.0	379.8	356.4	365.5	374.8	424.6	439.5
		K.GALLSC#PC#GPATAEFA EEYTQK.F	2	94.8	111.0	250.6	330.9	173.8	202.2	279.2	316.2
		K.GALLSC*PC*GPATAEFAE EYTQK.F	2	94.8	111.0	250.6	330.9	173.7	202.2	278.9	316.2
		K.GALLSC*PC*GPATAEFAE EYTQK.F	3	64.5	81.5	210.0	231.5	155.2	176.6	248.7	268.9
		K.VC#VVDDSTDYGLGLAEA VR.T	2	249.3	227.1	552.3	520.4	765.8	688.1	674.4	656.4
		K.VC*VVDDSTDYGLGLAEA VR.T	2	246.9	215.9	546.6	501.2	735.0	662.2	656.0	639.3
		R.GLANDGVQGPSVANYMK. N	2	353.8	371.1	748.1	843.9	341.8	362.9	507.6	552.9

		R.TTLGPVADASC#NISVK.K	2	307.5	272.3	797.2	703.7	659.4	648.7	1089.4	1100.1
		R.TTLGPVADASC*NISVK.K	2	310.9	275.4	801.4	708.8	660.7	650.9	1089.5	1101.2
MSMEG _3253	MSMEG 3264	K.TLPAVWDLAAPSTVLAVR. S	2	40.5	43.3	93.9	105.4	55.5	63.6	103.7	112.7
		R.GFWQGYFAAR.S	2	16.3	11.4	10.1	11.2	20.8	23.9	10.1	15.3
		R.LEPVHAVTYFSGESR.S	2	60.1	56.6	38.6	32.0	66.4	70.8	29.4	35.2
		R.LEPVHAVTYFSGESR.S	3	37.3	43.0	59.5	50.2	55.6	56.3	54.0	55.6
		R.LVVAAGDIPVDTPMGLR.R	2	512.0	584.3	787.9	830.8	412.9	445.2	768.0	798.9
MSMEG _3255	MSMEG 3266	K.VTASVAAALPAGAAASESI ADSAVAEK.L	3	63.6	87.2	234.2	258.3	33.5	37.4	212.9	246.1
		R.LTAAALALSVVPGSLGSH SFWK.E	3	58.7	61.4	122.7	123.0	38.0	35.2	105.2	105.3
MSMEG _3257	MSMEG 3269	R.GADGLTLVPYLEGER.S	2	136.7	133.4	114.5	113.2	150.5	143.8	168.3	159.0
		R.PVDVPPPGEYVALGAAR. Q	2	396.5	387.0	289.3	315.0	241.3	265.1	121.8	171.1
		R.SASSADDLVSELGGPDE WAK.T	2	207.1	219.8	82.2	109.7	152.7	173.2	92.9	107.9
MSMEG _3278	MSMEG 3290	K.VSVTVQLDAR	2	0.0	0.3	2.5	3.1	0.3	0.1	1.7	1.4
		R.NFLGEGSSITLVGLDGAD R.T	2	30.4	32.7	48.1	50.7	34.3	33.3	46.6	45.5
		R.VPILGSLVR.R	2	23.8	21.5	29.5	27.0	25.4	24.7	23.3	24.8
MSMEG _3280	MSMEG 3293	K.IDDLWDPAFK.G	2	5.4	4.4	26.9	33.0	24.9	26.1	21.0	33.0
		K.LVAFTQFVPVLSDMTDEL AK.I	2	37.8	39.0	206.4	200.4	21.8	26.7	125.8	182.8
		R.KQDIGADLVVPTEFMAAR. L	2	75.9	69.4	85.7	104.9	95.5	98.9	27.2	57.2
		R.LMGLNWLNEINESR.V	2	51.2	49.8	168.7	155.5	51.3	52.8	103.5	95.1
MSMEG _3284	MSMEG 3296	R.LLQLQPSNVSTTVR.H	2	19.7	23.2	50.7	61.6	12.9	16.2	38.9	53.0
MSMEG _3287	MSMEG 3299	R.VGELVEDVVALIDASGAD R.V	2	69.1	78.6	70.3	83.9	95.8	110.5	64.2	83.8

		R.VGELVEDVVALIDASGAD R.V	3	13.4	12.1	20.1	19.5	16.3	18.8	15.6	17.3
MSMEG _3309	MSMEG 3318	K.VIAAEPAAC#PSITQGEYR. Y	2	116.4	88.2	58.2	38.7	79.3	110.8	79.2	111.8
		K.VIAAEPAAC*PSITQGEYR. Y	2	116.4	88.2	58.2	38.7	79.3	110.8	79.2	111.8
MSMEG _3379	MSMEG 3385	K.AGVASLIDSMAVELAR.H	2	30.5	32.5	33.7	33.2	20.7	20.3	21.3	25.3
MSMEG _3380	MSMEG 3387	R.GLVEDIVPDPTGAFYLK.L	2	163.2	180.8	144.1	149.0	114.3	131.9	234.2	219.2
MSMEG _3381	MSMEG 3388	K.AWLDYVHVIGQTSPAAQG VC#PLK.G	3	175.1	182.1	214.8	216.9	117.0	126.9	269.6	281.0
MSMEG _3394	MSMEG 3400	K.VISLADAPEIPGR.R	2	11.7	9.4	3.3	4.9	13.8	17.8	1.6	3.6
MSMEG _3434	MSMEG 3442	R.GAPVDFPLNGVVAGFQK. A	2	91.1	85.6	36.5	45.9	109.5	114.2	74.4	82.8
MSMEG _3461	MSMEG 3467	K.DGAGAGTIPDPFGGPGR. N	2	328.1	324.9	581.8	537.8	499.8	473.2	698.6	635.1
		K.DGGYNVTVPFAPGR.T	2	172.3	156.5	486.1	463.9	143.3	156.3	482.6	488.6
		K.GPDTITSGLEVVWTNTPT K.W	2	162.3	90.8	315.3	212.5	84.6	234.7	286.6	418.7
		K.NWDVNEPAELATVLPVLE R.I	2	66.0	68.3	192.6	184.0	61.4	71.0	189.5	214.7
		K.STVLDSGLSTGQLIK.T	2	198.8	212.1	628.8	635.0	222.5	201.1	711.7	664.3
		K.THGAGDASLVGPEPEAAP IEQQGLGWK.S	3	209.2	225.2	658.7	649.7	154.8	210.5	415.3	568.4
		K.VSLADLIVLAGSAAIEK.A	2	71.9	72.1	191.4	196.8	84.5	78.4	237.6	223.9
		K.VSLADLIVLAGSAAIEK.A	3	25.7	24.5	63.0	57.1	37.0	29.2	93.9	75.2
		K.WTATANDLVFGSHSVLR. A	2	24.5	24.9	198.6	196.8	33.3	36.8	228.2	216.9
		K.WTATANDLVFGSHSVLR. A	3	133.4	114.5	141.7	119.9	133.9	142.0	209.6	145.4
		R.AVAEVYAQSDNGER.F	2	6.3	5.0	23.4	17.0	3.8	5.1	17.5	21.5
		R.AYFLGVTAPQLTALVGGL R.A	2	110.9	114.9	231.6	226.1	109.4	139.0	276.3	314.5
		R.AYFLGVTAPQLTALVGGL R.A	3	11.2	17.1	25.4	33.7	18.0	18.1	35.7	40.0

		R.EDIWEPEEILFGEEDEWLGTDKR.Y	3	26.6	44.5	61.4	97.5	18.5	40.8	54.2	109.1
		R.FAPINSWPDNVSLDK.A	2	363.2	359.3	672.5	657.8	313.7	317.5	632.4	686.1
		R.IQQDFNASASGGK.K	2	6.3	4.1	19.6	12.1	3.0	2.9	11.8	13.1
		R.LHPHPPQGNPLGEDFDYAEEFAK.L	3	239.6	244.6	531.4	486.8	207.9	220.7	538.2	452.8
		R.LHPHPPQGNPLGEDFDYAEEFAK.L	4	73.3	45.9	186.9	123.3	99.8	10.1	228.4	44.8
		R.NPTMLVTDISMR.V	2	50.7	54.1	201.4	164.3	52.1	34.2	178.6	137.1
		R.NQDWWPNQIDVSR.L	2	130.7	138.0	277.6	303.9	108.1	128.3	276.9	323.1
		R.TDASQENTDVESFAVLEPR.A	2	123.0	113.9	179.6	187.3	105.1	121.9	194.8	224.1
		R.WLDHPEELSEAFAK.A	2	83.2	71.0	148.2	138.8	76.6	93.5	182.2	179.4
		R.WLDHPEELSEAFAK.A	3	40.9	39.3	88.1	84.4	49.4	45.1	112.4	109.6
MSMEG_3465	MSMEG3471	R.VITAAPNFAYEWTAQR.G	2	1623.0	1669.7	92.2	71.0	978.8	993.6	55.5	65.0
MSMEG_3478	MSMEG3483	K.APTLADTAMFAR.H	2	60.3	51.5	49.7	48.1	45.8	46.6	40.1	47.1
		K.TFAHYVAQDVHYLR.D	3	52.9	58.0	51.9	56.8	38.6	41.4	35.9	46.5
		K.TFAHYVAQDVHYLR.D	4	39.4	33.0	20.8	22.9	29.1	25.8	26.0	22.0
		R.LGATLGETEQATAQR.H	2	23.7	19.6	22.7	19.9	20.4	13.7	25.8	18.5
MSMEG_3479	MSMEG3484	K.AASSGATVLC#VSK.D	2	8.9	7.7	7.6	6.3	11.9	7.7	8.5	4.9
		K.AASSGATVLC*VSK.D	2	8.9	7.7	7.6	6.3	11.9	7.7	8.5	4.9
		K.SVLLNIFPSVDTPVC#ATSVR.T	2	543.2	771.5	713.2	993.7	993.7	947.5	961.5	827.9
		K.SVLLNIFPSVDTPVC#ATSVR.T	3	227.3	170.5	307.6	223.3	316.1	388.1	330.1	367.2
		K.SVLLNIFPSVDTPVC*ATSVR.T	2	575.2	806.5	843.1	1129.4	1089.5	1040.6	1200.5	1068.8
		K.SVLLNIFPSVDTPVC*ATSVR.T	3	229.9	173.8	341.3	259.2	329.6	407.8	386.6	434.4
		R.FC#GAEGIENVTTASAFR.S	2	870.5	887.4	1059.0	1049.3	992.2	1061.7	1122.5	1055.3
		R.FC#GAEGIENVTTASAFR.S	3	116.7	88.0	116.8	98.3	124.1	120.9	89.2	92.3

		R.FC*GAEGIENVTTASAFR.S	2	98.3	115.4	172.2	193.2	203.9	227.5	328.9	322.1
		R.FCGAEGIENVTTASAFR.S	2	98.5	115.4	172.2	193.2	205.4	230.4	329.7	322.1
		R.GNPINTVGELPAVGSSAPGFTLTGTDLGEVTNDQFSGK.S	3	752.5	737.0	732.8	798.8	650.3	928.0	493.4	694.6
		R.SSFGEDFGITIADGPM*AGLLGR.A	2	62.9	65.4	47.2	50.6	69.8	60.9	41.7	34.1
		R.SSFGEDFGITIADGPMAGLLGR.A	2	68.5	65.4	60.1	55.8	91.2	83.3	64.6	56.9
MSMEG_3489	MSMEG3494	R.AVKPDDSLVVFYGSR.N	2	18.4	0.0	172.5	0.0	10.8	13.4	153.1	137.5
		R.GSIFADLTPR.A	2	5.1	5.2	9.0	6.8	11.5	11.4	7.0	11.2
MSMEG_3500	MSMEG3504	K.LAAEQLIVDLR.S	2	7.0	5.9	5.3	6.1	11.2	14.4	7.0	8.1
		R.LAVGSALFGLVR.A	2	5.3	4.2	0.7	1.1	11.2	9.1	2.5	3.3
MSMEG_3505	MSMEG3509	R.HLNAVITDLFER.G	2	210.2	202.9	247.8	255.1	74.4	80.4	171.9	175.2
MSMEG_3507	MSMEG3511	K.GLAEEADGVQLMK.P	2	209.8	211.6	151.4	139.5	159.9	145.7	77.2	80.6
		K.GLAEEADGVQLMKPMPTLDDLLAR.G	3	90.0	107.7	100.9	119.0	120.5	120.3	100.1	112.6
		K.LLAQNTGVIASFSR.A	2	1205.3	1374.4	1401.5	1550.9	1439.4	1511.4	1146.7	1215.0
		K.LSLPTVANHYK.S	3	66.5	39.3	37.6	18.2	66.7	70.6	11.4	12.0
		K.M#TSGQGFIAALDQSGGSTPK.A	2	93.0	95.4	102.9	110.9	87.8	81.6	39.3	47.0
		K.M*TSGQGFIAALDQSGGSTPK.A	2	92.9	95.4	102.9	110.9	87.8	81.6	39.3	47.0
		K.MTSGQGFIAALDQSGGSTPK.A	2	152.2	158.7	132.0	138.5	225.5	216.1	99.8	92.8
		K.PMPTLDDLLAR.G	2	130.4	134.5	162.8	165.2	96.5	99.4	19.2	22.5
		R.LYGVEESAYSNEDEMFDLIHQMR.S	3	328.1	356.0	320.8	320.4	390.7	408.4	176.9	161.7
		R.SVIGAANPTGVAAVVAQQFEVAK.Q	2	402.9	434.4	549.8	551.4	497.9	503.4	369.6	351.4

		R.SVIGAANPTGVAAVVAQQ FEVAK.Q	3	546.5	546.7	671.8	683.0	725.6	692.3	450.0	437.5
		R.TIEGKPSATYLWEDK.G	2	96.3	99.7	71.8	82.7	73.6	88.6	62.6	86.5
		R.VLAAILFEQTM*DR.T	2	142.7	146.1	293.0	276.4	251.5	221.6	243.6	199.5
		R.VLAAILFEQTMDR.T	2	469.7	467.6	624.4	610.0	543.2	512.6	369.4	335.9
MSMEG _3512	MSMEG 3516	R.YEPAAADAYGQLLDVLR. D	2	93.9	86.8	98.9	85.5	121.9	79.4	133.3	110.6
MSMEG _3515	MSMEG 3518	K.AAGGGSIINVSSIEGLR.G	2	57.1	67.0	43.7	60.5	40.9	56.2	41.7	44.9
MSMEG _3564	MSMEG 3564	K.LGDALYAAQC#VSRPPGS A	2	155.7	189.3	517.8	593.5	342.9	351.1	528.7	498.6
		K.LGDALYAAQC*VSRPPGS A	2	103.9	120.4	737.6	826.5	218.6	220.6	752.7	709.1
		K.LGDALYAAQC*VSRPPGS A	3	40.5	43.1	164.7	187.7	81.7	89.1	161.6	169.6
		K.LLNEQLTSELTAINQYFLH SK.M	3	255.1	246.5	1402.8	1382.7	615.9	692.4	1476.8	1472.1
		K.M#QDNWGFTELAEHTR.A	2	0.2	0.1	5.1	6.4	0.2	0.3	3.1	2.6
		K.M#QDNWGFTELAEHTR.A	3	51.2	46.7	240.3	230.1	83.7	77.9	146.8	138.5
		K.M*QDNWGFTELAEHTR.A	3	36.2	36.7	192.5	175.8	79.5	70.8	146.8	138.5
		K.MQDNWGFTELAEHTR.A	3	36.0	36.4	194.4	175.8	79.3	82.5	182.6	182.7
		R.ILLLDGLPNYQR.L	2	154.5	170.7	912.5	973.9	307.2	289.7	922.4	871.3
		R.ILLLDGLPNYQR.L	3	0.0	0.0	5.6	8.6	0.0	0.0	0.9	5.3
		R.LLEQILADEETHIDYLETQL QLMDK.L	3	3.7	4.7	15.0	18.9	12.9	16.2	15.1	21.1
MSMEG _3569	MSMEG 3569	R.TDALANIALLR.R	2	11.9	11.6	4.4	3.4	6.8	6.8	4.9	6.1
MSMEG _3574	MSMEG 3574	K.AWDAFDPVDLAASLTR.A	2	103.5	94.1	98.8	98.1	67.7	63.3	107.2	105.0
		R.GPLNFYSLYR.A	2	56.4	65.7	5.8	10.7	116.0	110.7	1.3	5.4
MSMEG _3575	MSMEG 3576	R.EALDDGDEPFGSVLVDHT GTTLFEDR.N	3	71.1	74.8	90.3	94.1	74.6	64.4	95.5	92.4
MSMEG _3580	MSMEG 3581	K.WETFLTQELPAWLEANR. G	2	261.3	224.5	4.5	5.2	173.0	149.5	2.4	8.1

		R.NDPMVNINQLVANNTR.L	2	60.4	65.8	9.3	5.5	33.2	41.6	6.2	3.5
		R.VQFQGGGPHAVYLLDGLR.A	3	152.0	150.9	21.7	19.4	94.6	99.8	20.5	22.5
MSMEG_3595	MSMEG3599	R.LADRPGGWVGLAR.L	3	12.0	7.9	9.4	5.3	7.2	8.9	9.2	10.2
MSMEG_3596	MSMEG3600	R.AIIGDDDLTPEILTDFVTALHK.A	3	17.2	19.4	29.1	31.4	13.6	16.6	29.3	29.7
		R.AMTDVLAAFDDAAR.E	2	9.3	8.6	6.9	8.5	8.0	7.3	6.1	6.8
		R.GVSSIVLDPNNDLSR.L	2	16.9	19.7	18.6	20.6	10.4	14.2	14.3	18.3
		R.HC#LEHDTVEELTR.L	3	2.6	1.5	1.9	1.4	0.9	1.8	0.6	1.5
MSMEG_3598	MSMEG3602	R.TGVPIVGIDGIEDGLNAVK.S	2	110.4	96.2	173.8	176.1	147.3	130.4	167.4	157.8
MSMEG_3609	MSMEG3614	R.INVAPGSSVSQVADAVK.I	2	27.6	29.3	41.3	41.0	28.9	26.5	79.9	88.4
MSMEG_3618	MSMEG3624	K.FTDANKPAGQIWAGVVGQPVAPGTPR.G	3	607.4	575.5	788.1	784.8	379.0	399.9	599.0	724.3
		K.TVAEGAEPPNDTSVLLGR.L	2	512.0	521.4	520.7	552.6	345.8	384.8	419.7	516.1
MSMEG_3619	MSMEG3625	K.AALSDWTAGQADFFGTR.G	2	248.8	249.9	51.7	58.1	196.5	210.1	48.5	46.3
		R.LALFLSTPAAR.H	2	75.7	81.1	38.4	44.8	63.6	73.7	79.0	80.9
MSMEG_3620	MSMEG3626	K.AITTLDVVSR.G	2	9.0	10.4	0.9	1.6	4.0	5.7	0.4	0.4
		K.LDEALQIILPMLK.G	2	229.0	218.4	238.9	211.0	78.1	72.1	100.9	86.0
		R.FRDHIPLMIGGSGEK.K	3	101.1	105.9	38.6	37.8	48.9	56.7	15.7	19.6
		R.HFDHLNVIAGFDELAR.K	3	154.1	154.5	115.0	118.9	48.2	51.3	58.6	52.0
		R.HFDHLNVIAGFDELAR.K	4	27.9	25.2	2.7	1.3	5.5	5.6	0.0	1.1
		R.LALQIPNFSYGTGVEELFPTVIAQAK.E	3	629.4	723.2	538.9	657.0	246.7	347.0	347.8	478.7
		R.PGDITAIGEALKPLISG	2	49.3	43.4	32.2	39.8	6.2	6.7	24.1	18.5
		R.VQLGTLVTGNTYR.N	2	747.2	762.9	358.7	391.2	552.3	491.8	249.3	258.6
MSMEG_3621	MSMEG3627	K.DLAEQSGVELDR.A	2	25.6	26.5	31.6	30.4	21.6	30.9	35.5	40.9
		R.TRIEELEEIAAAVQDTEK.A	3	21.5	19.9	7.0	6.5	35.6	27.1	9.3	14.7
MSMEG_3623	MSMEG3628	R.AERPTALISLTEDPTAGPVLDWVR.E	3	223.3	239.4	328.9	344.4	391.1	310.8	434.5	418.4

MSMEG _3625	MSMEG 3631	K.GNTVSADSMWEQLR.G	2	135.6	138.6	160.8	158.7	136.3	121.0	79.8	70.0
		K.LADLVLWEPAFFGVR.P	2	23.0	28.1	94.1	119.4	6.7	14.0	37.2	55.8
		R.M#LESLDSWPMNIVLLGK.G	2	12.4	17.8	18.2	21.3	4.3	14.9	2.3	6.0
		R.MLESLDSWPMNIVLLGK.G	2	96.2	66.4	72.1	90.5	43.1	63.3	34.6	65.6
		R.SGGPELAGDEAVFGGGK.V	2	75.2	55.1	226.5	184.2	53.8	41.7	153.4	103.2
		R.SNYAALFGPTTGDR.I	2	33.7	43.1	141.2	150.1	13.3	17.8	73.5	67.9
MSMEG _3627	MSMEG 3633	R.LLISYAAELAR.R	2	4.2	5.5	10.1	9.3	8.6	7.5	16.9	16.5
MSMEG _3628	MSMEG 3634	K.GFDSEIGLNYTELGPDGGR.A	2	86.5	92.3	66.7	64.1	63.5	73.0	58.5	64.1
		R.AIGSGTVTAVSTPIHR.G	2	31.2	21.9	22.8	14.9	25.8	27.2	22.2	21.7
MSMEG _3632	MSMEG 3638	K.ATVGLASGALGATPSDAADFIEAVR.R	2	141.4	148.7	183.3	204.8	162.1	130.1	176.3	169.3
		K.ATVGLASGALGATPSDAADFIEAVR.R	3	229.4	202.8	272.2	256.5	267.7	223.7	350.6	309.3
		K.SALDLGVPVTGIAEAVFAR.A	2	776.4	717.0	1110.7	1052.8	741.0	650.4	1052.0	932.6
		K.SALDLGVPVTGIAEAVFAR.A	3	36.4	44.8	24.7	34.3	40.5	44.0	32.5	34.8
		K.TGKPLVDVIVDEAEQK.G	2	167.2	199.6	667.2	798.3	195.2	224.1	473.3	551.5
		K.TGKPLVDVIVDEAEQK.G	3	152.9	123.1	138.6	126.2	181.7	147.9	174.9	151.0
		R.GDLDSFLVEITAK.V	2	73.7	60.6	77.2	70.1	102.3	74.8	105.6	90.6
		R.IKDAYDAEPDLPTLLAAPYFR.D	3	208.4	186.8	286.0	262.6	391.1	305.1	589.2	488.4
		R.LPAALIQGLR.D	2	44.0	33.6	91.7	76.8	73.7	71.8	79.5	66.4
		R.SETIAEFIDALEKPR.K	2	316.1	309.0	440.6	438.5	292.6	285.6	438.4	399.1
MSMEG _3634	MSMEG 3639	K.VVLGVQSAAGFAEGHPLPAGW	2	120.3	122.2	68.1	73.3	83.6	82.9	58.7	66.6
		R.DIALSDFVTAPVGTDPR.E	2	251.4	227.2	224.9	211.3	178.7	183.9	298.9	248.4
		R.DLIEAGASIVK.V	2	56.1	43.9	30.8	25.3	43.1	29.1	46.3	49.8

		R.GGVEDLLDHITSGVR.S	3	60.5	50.4	22.6	21.0	35.3	33.9	34.1	29.0
		R.RGGIVVLPQDLPITAVSETVDFVK.S	3	202.3	190.7	127.4	146.7	170.7	151.9	214.2	185.8
MSMEG_3640	MSMEG3646	K.GMWAMTDLMADMVEQK.I	2	73.1	73.6	39.2	46.0	25.3	34.2	14.3	21.5
		K.IDDGALEVALGDDK.V	2	359.8	343.0	196.2	184.9	215.9	202.5	189.5	193.2
		K.TPDGEGELTLPGR.S	2	51.9	40.3	19.5	11.9	26.9	25.8	10.9	10.0
		K.VLHDFITNEALPGTGVDPDSFWSGVDK.V	3	1086.0	1293.6	468.5	535.2	588.2	918.6	339.3	551.6
		K.VPDIHDVALM*EDR.A	2	24.8	18.6	34.1	30.6	14.1	8.6	43.1	50.2
		K.VPDIHDVALM*EDR.A	3	62.6	41.1	45.5	30.2	40.6	20.5	28.4	22.5
		K.VPDIHDVALMEDR.A	2	187.9	203.1	177.3	180.2	133.6	136.3	167.6	146.3
		K.VPDIHDVALMEDR.A	3	388.5	357.3	210.6	190.0	249.9	234.1	178.6	171.4
		R.GFLDEVVPLAEGSWSEATGLK.I	2	394.4	425.3	197.1	209.6	264.1	335.5	187.3	259.7
		R.GFLDEVVPLAEGSWSEATGLK.I	3	305.6	391.1	125.2	154.2	207.2	274.4	130.3	171.6
		R.HGVITEEDVK.T	2	0.4	0.2	10.6	7.8	0.1	0.2	7.9	6.7
		R.ISSQLLANWLR.H	2	420.1	394.4	163.7	161.9	413.1	300.0	240.4	189.6
		R.RDELQAQIDK.W	2	2.0	1.2	0.0	0.0	0.7	1.2	0.1	0.1
		R.SHVLEPVDPVAYK.Q	2	134.5	90.5	71.2	47.0	112.8	38.6	80.2	25.7
		R.TGDEIHTSMEAGPM*VR.K	3	1.7	1.3	0.2	0.2	0.7	0.7	0.2	0.3
		R.TGDEIHTSMEAGPMVR.K	2	47.4	32.2	102.3	73.2	21.6	14.2	88.5	50.9
		R.TGDEIHTSMEAGPMVR.K	3	42.8	35.5	13.4	13.1	16.7	19.4	6.9	7.5
		R.VEDVLGLPANTLK.V	2	799.6	848.0	421.5	415.4	766.9	591.5	611.4	452.0
		R.VVFINTGFLDR.T	2	797.1	815.7	441.0	410.7	711.5	532.6	601.9	413.3
MSMEG_3642	MSMEG3648	K.ALPTGILDALDAAGR.A	2	229.4	205.9	195.0	173.8	658.4	474.4	325.6	227.2
		K.FFDTVLVR.V	2	148.0	137.2	106.5	89.0	334.3	241.2	181.4	150.3
		K.LNAAAEMEPITWTEFSR.Q	2	349.0	323.1	99.0	87.7	432.4	376.7	75.1	80.7
		K.TFC#IPHGGGGPGVGPVAVR.A	2	11.5	15.0	17.6	31.9	35.4	55.2	8.7	64.2

		K.TFC#IPHGGGGPGVGPVAVR.A	3	97.7	89.9	61.8	72.4	200.3	236.8	69.3	105.7
		R.AATLTAIASANYVAR.R	2	87.6	79.1	368.3	388.7	208.3	206.1	302.4	311.8
		R.APGLAELPPAATEEEALAELR.A	2	209.1	216.1	202.6	170.3	428.8	443.4	270.3	190.8
		R.APGLAELPPAATEEEALAELR.A	3	16.8	17.1	73.0	57.6	22.9	29.7	66.5	65.1
		R.ARPLGIEIVTADLSK.G	2	126.1	134.3	100.0	113.1	330.9	279.5	136.0	119.5
		R.ARPLGIEIVTADLSK.G	3	110.3	100.1	62.6	58.6	304.4	238.4	96.0	74.4
		R.GAPHTADC#LLVADWNRPYTR.E	4	30.2	30.2	20.5	19.6	95.4	107.3	25.7	22.9
		R.LVGVSVDSDGSPAYR.L	2	162.8	129.1	137.9	115.3	282.1	211.1	135.9	105.6
		R.NILENPAWYTAYTPYQPEISQGR.L	3	67.7	82.7	98.5	103.1	107.6	152.6	39.9	75.7
		R.RVHAHADAIASALGDAVVHDK.F	3	5.2	13.1	18.4	19.5	7.8	48.6	5.0	21.2
		R.RVHAHADAIASALGDAVVHDK.F	4	30.5	34.0	13.4	18.3	43.8	96.1	4.8	24.7
		R.VGSGEWPVDDNPLR.G	2	514.4	510.2	624.2	597.4	858.4	813.9	724.8	675.2
		R.VHAHADAIASALGDAVVHDK.F	4	89.5	77.3	41.8	41.1	156.3	181.2	33.8	47.8
MSMEG_3647	MSMEG3653	R.EPVDSAVLANGDEVQIGK.F	2	346.5	394.7	403.5	412.7	206.6	250.2	236.4	282.3
		R.FLLDQPTTSAGR.H	2	9.2	0.0	1.4	0.0	6.8	8.2	0.3	1.0
		R.HPDSDIFLDDVTVSR.R	2	389.7	422.2	581.1	594.9	379.0	356.7	926.6	922.6
		R.HPDSDIFLDDVTVSR.R	3	140.9	143.8	192.3	175.6	148.5	145.1	194.7	180.8
MSMEG_3648	MSMEG3654	R.VEAGTLDEALGGLLDAEGYR.A	2	680.1	824.6	832.2	961.3	611.1	681.4	793.3	796.3
		R.VEAGTLDEALGGLLDAEGYR.A	3	72.1	53.8	81.7	63.2	63.2	67.5	68.1	74.3
MSMEG_3654	MSMEG3660	K.LEDLAGASDITQFLAIAR.E	2	63.4	66.0	75.5	82.8	72.1	62.0	94.0	101.5
		K.LGVSPIPPNTPNIR.K	2	97.3	97.1	93.6	84.2	96.9	89.7	122.2	128.4
		K.TGQPVLVGTHDVAESEELHEK.L	3	73.4	69.3	84.2	86.1	58.3	66.7	109.2	126.9
		K.TGQPVLVGTHDVAESEELHEK.L	4	103.5	99.7	70.5	76.3	69.2	86.5	73.1	92.4

		K.TLAGAIAAAGYALGGR.R	2	47.3	48.2	81.0	73.6	52.8	48.1	98.2	70.0
		R.TTGLRPFDVQLLAALR.M	3	21.8	22.4	21.7	24.0	19.0	25.7	23.0	41.7
		R.VHVITINDYLAR.R	3	16.6	12.2	75.0	62.5	15.2	12.9	86.4	65.3
MSMEG _3673	MSMEG 3679	R.VPLSGPDGRPLLLEEVFD DVR.A	3	16.1	20.8	47.0	56.6	26.9	20.0	50.7	49.2
MSMEG _3710	MSMEG 3716	R.GPPLPSTVGALER.K	2	14.1	0.0	4.2	0.0	0.9	2.1	1.1	1.4
MSMEG _3715	MSMEG 3722	R.VRLEKVFDC*GDPLLLNR. F	2	29.7	29.2	64.0	69.8	21.4	22.6	49.4	50.5
MSMEG _3740	MSMEG 3746	K.AGVELDDGPAHVDDFAVV DTVPGK.T	3	82.3	99.5	79.2	91.2	93.0	94.9	77.4	91.5
		R.LLAAVDFPVQELVR.T	2	48.4	48.2	84.9	80.3	65.9	59.5	87.9	85.1
MSMEG _3745	MSMEG 3751	R.VLVDLDSTPGFSDESVR.V	2	66.4	73.5	47.1	54.0	35.7	23.7	27.5	6.0
MSMEG _3746	MSMEG 3752	K.HLFVTGGVVSSLGK.G	2	47.9	48.4	99.1	108.3	53.3	57.5	70.2	98.7
		K.VLHREELDAYVVR.R	3	13.6	16.7	16.4	18.0	25.4	25.2	19.0	25.5
		K.YIDLSDAYLSVAEALR.A	2	132.3	124.9	139.1	141.1	170.1	141.4	174.7	139.3
		R.DVDWTEWDDLLR.R	2	25.7	39.5	29.7	43.7	27.6	37.0	25.1	34.9
		R.DVDWTEWDDLLRRVHEP QETVR.I	3	17.7	19.5	621.2	638.6	10.6	9.4	775.7	720.6
		R.NLSGSANVTTGQVYSSVI AK.E	2	77.4	77.9	306.2	254.2	84.0	88.3	236.7	263.6
		R.RGEYLGDTVQVIPHITDEI K.S	3	109.3	138.0	126.0	146.4	137.7	146.6	141.4	173.8
		R.RGEYLGDTVQVIPHITDEI K.S	4	24.2	20.6	14.7	13.0	28.8	29.2	12.3	16.2
		R.SIGITPDALILR.C	2	86.2	78.1	759.3	768.2	108.9	86.4	690.9	539.6
		R.SVGITEANSAEFDPK.T	2	114.3	92.0	113.2	87.7	123.6	96.6	117.3	86.2
MSMEG _3754	MSMEG 3760	R.SPEAEALTGEDADELR.I	2	60.5	61.5	55.1	42.9	41.6	51.3	20.5	29.3
MSMEG _3758	MSMEG 3764	R.GELADLDESTLSAALR.E	2	62.4	75.2	133.0	133.6	55.8	54.5	186.7	175.4
		R.TVAEGGVYVNNAR.V	2	13.8	10.2	10.8	9.9	7.1	5.9	7.9	6.1
MSMEG _3767	MSMEG 3773	R.AAPQADVYEYLIR.A	2	33.7	41.8	116.9	110.8	34.5	40.6	117.9	113.0

		R.AGIDPWVIDFGEPDKVEG GMER.T	3	3.3	4.1	13.1	12.4	3.8	3.3	7.3	9.1
		R.GPVFATTISDAAGR.L	2	11.0	12.4	58.4	62.4	12.1	12.3	45.0	43.8
		R.GVFAPADTWVSTEYLFR. R	2	62.9	69.6	64.2	72.7	25.7	43.8	38.5	68.4
		R.IDIPGWLAR.T	2	14.0	13.0	10.6	9.9	19.9	13.7	23.1	15.4
		R.TLADHVVALSEAIDTVR.E	3	2.4	3.5	11.9	13.5	2.7	2.6	8.4	7.3
MSMEG _3770	MSMEG 3776	K.AIAFAEENAIPINVTK.R	2	1988.1	2071.0	357.4	370.8	1836.9	1954.9	369.7	398.9
		K.GVPVSIDGRPVSVLQAIEE LNR.R	3	226.4	221.9	136.0	142.4	154.6	140.8	124.2	108.9
		R.ALESFVAHTQEHVSGEIR. L	3	174.9	222.3	66.7	145.0	137.3	110.7	68.4	85.0
		R.AVETGFLEHLWNAPTK.D	2	128.4	115.5	72.7	60.0	105.2	117.1	103.5	102.4
		R.FEVGFASLAPDLQVLAPV R.D	2	314.6	297.6	162.0	167.1	276.0	264.9	194.0	192.3
		R.PVSVLQAIEELNR.R	2	65.7	49.5	20.9	19.0	32.7	24.4	18.7	15.9
		R.SPFSIDQNVWGR.A	2	122.3	114.1	70.6	81.0	121.0	116.7	63.5	89.7
MSMEG _3773	MSMEG 3779	K.TLSHGIEELGHPLVDK.V	3	35.9	18.2	12.2	10.9	27.2	31.7	21.4	27.5
MSMEG _3775	MSMEG 3781	K.AAPVQWSQQVLTTGNLR. A	2	378.5	472.6	86.1	97.0	426.6	437.4	102.3	105.6
		K.TALFGSDANWGR.V	2	27.0	21.3	13.2	14.3	13.5	19.7	7.3	9.0
		R.TTDLSHAYVEENSAYSS	2	49.9	56.9	15.8	13.7	32.3	38.7	11.1	14.5
		R.VLAAVGIAPVTLDPQR.I	2	132.9	148.8	203.6	220.3	80.5	89.7	180.7	195.6
MSMEG _3776	MSMEG 3782	K.VDLLGAEVIGSAR.A	2	19.0	20.7	86.1	96.1	8.9	6.2	62.6	111.9
MSMEG _3777	MSMEG 3783	R.AAGVEFTFR.A	2	35.7	10.5	12.2	15.1	77.7	20.6	7.9	4.6
		R.AGAPDWDVTPEELEQTFI R.I	2	138.9	136.2	128.0	121.2	138.5	161.4	141.0	167.3
		R.DGAGELLEDVR.L	2	25.2	16.9	32.7	15.7	33.5	9.7	20.3	15.1
		R.EPAGPWGPGRPTEAADA FEAVR.V	3	356.9	370.7	287.9	314.6	376.2	386.2	372.9	390.3

		R.FGLRPVTGIDPAAVSPW WMQR.R	3	193.3	284.1	52.3	73.1	147.0	278.2	84.6	106.4
		R.GAADVALFGIQQVVQPTD ATK.A	2	90.7	91.6	110.8	104.9	110.8	107.5	121.3	147.1
		R.IGHEVEEIITAGPVSGPLT VGR.V	3	204.5	252.5	197.1	246.1	218.3	240.6	277.0	263.8
		R.LEGLEIIPSVLPTAPAGR.G	2	236.0	225.7	390.7	396.7	296.3	242.9	389.2	381.5
		R.LFDVYTGPQIGEGR.K	2	298.3	288.9	345.2	338.6	243.9	238.3	377.9	403.9
		R.RPTDEEIAALDASLPR.Q	2	64.8	66.0	48.4	50.6	61.1	92.0	65.0	95.1
		R.RPTDEEIAALDASLPR.Q	3	84.9	82.4	59.6	72.9	106.1	117.8	84.6	118.6
		R.SVDPAISVAALDR.C	2	279.3	243.7	121.7	118.7	401.6	257.8	175.9	117.6
		R.TTQVLNPLESDRPHLATT LLPGLLEALSR.N	4	165.5	203.1	182.4	224.8	197.1	206.2	228.8	258.3
		R.VIEIEELTEFK.K	2	73.7	75.2	74.9	77.4	108.1	78.6	134.1	90.0
MSMEG _3778	MSMEG 3784	R.DLPVYIISIGR.T	2	33.4	36.9	18.2	21.0	22.2	28.4	25.5	8.1
MSMEG _3791	MSMEG 3797	K.AKEQQLHSLTYAYR.D	3	13.5	5.5	5.9	1.5	20.3	16.3	8.6	5.8
		K.EQQLHSLTYAYR.D	2	8.0	5.6	2.4	1.6	9.6	9.0	3.6	1.9
		K.NLAELAVSDPAAFTALVDV AR.A	2	502.9	564.9	321.6	356.4	446.0	494.3	305.6	308.9
		K.NLAELAVSDPAAFTALVDV AR.A	3	283.4	222.8	184.8	143.2	240.7	278.5	155.3	171.1
		R.AALPEDVNAPSGEAA	2	94.5	86.0	81.3	73.7	90.5	119.4	83.5	95.3
MSMEG _3793	MSMEG 3799	R.LGADVAEYGFVETSAK.Q	2	126.6	127.8	335.2	348.7	127.5	115.3	308.9	322.4
		R.LIGPGGEQVGIVR.I	2	187.2	152.1	186.8	148.9	133.7	167.7	153.4	185.5
		R.VAADADLDLVEVAPNARP PVC#K.I	3	269.5	300.1	1921.2	2215.5	401.4	381.5	2035.8	2162.6
		R.VAADADLDLVEVAPNARP PVC*K.I	3	269.5	299.7	1919.6	2208.6	401.4	381.5	2025.2	2162.6
MSMEG _3808	MSMEG 3814	K.EIDVAFPLGVLTSVTGVSG SGK.S	2	72.7	65.3	36.0	33.8	80.4	71.3	29.9	39.9
		K.HDIEVVVDR.L	2	3.9	2.9	0.4	0.3	2.7	3.9	0.1	0.6
		R.STVGTITEVYDYLR.L	2	96.4	102.6	95.4	88.3	107.5	83.0	102.1	73.8
		R.VDGVVYPLTDPPK.L	2	32.6	26.5	299.8	257.5	24.5	20.1	337.5	329.9

MSMEG_3810	MSMEG 3815	K.ASVGPMDNNC#YLVTC#R.S	2	43.5	42.2	40.9	43.7	52.7	64.3	46.9	50.9
		K.ASVGPMDNNC*YLVTC*R.S	2	42.4	41.6	40.8	43.6	52.7	64.3	46.9	50.9
		R.TVVYPGHGDDTTLGAERPSLAEWR.E	3	68.3	90.6	68.3	64.4	125.3	142.3	69.7	78.9
MSMEG_3811	MSMEG 3816	K.AAGATDIEERPVVGAPVDALVELADEVK.A	3	465.3	462.2	480.4	475.9	245.7	273.8	500.7	521.2
		K.AAGATDIEERPVVGAPVDALVELADEVK.A	4	101.2	70.5	82.6	49.4	41.4	47.7	64.8	69.6
		K.ADLLVVGNVGLSTIAGR.L	2	227.8	369.7	217.1	335.6	109.4	116.3	218.2	207.8
		K.ADLLVVGNVGLSTIAGR.L	3	70.5	43.7	52.4	29.7	36.7	36.2	59.4	50.3
		K.LIIATAYFPQSEDSR.A	2	2020.0	2651.7	2231.4	2786.6	961.8	1038.3	2079.5	2186.3
		K.LIIATAYFPQSEDSR.A	3	121.9	69.3	86.3	49.6	47.9	55.6	71.4	76.7
		K.M#AGNAPIYAILR.E	2	527.9	526.5	413.3	408.0	225.8	225.5	354.3	351.7
		K.MAGNAPIYAILR.E	2	443.3	492.6	508.0	504.2	242.0	220.4	561.8	468.3
		K.TDVLIVHTS	2	4.8	5.0	2.1	1.3	1.2	1.0	0.8	1.5
		R.PVVGAPVDALVELADEVK.A	2	459.0	899.7	422.1	797.2	156.8	144.4	298.6	262.6
		R.PVVGAPVDALVELADEVK.A	3	209.2	159.8	145.0	107.9	59.8	62.0	88.7	88.6
MSMEG_3816	MSMEG 3820	K.TLAAQLANELR.E	2	6.5	8.0	34.8	30.4	13.0	11.0	61.4	39.2
MSMEG_3831	MSMEG 3837	R.IAAQATVEER.R	2	10.0	7.8	7.1	5.4	7.7	6.0	6.7	5.0
MSMEG_3833	MSMEG 3838	K.FAAAEAEAANAPVSNGSSR.S	2	76.8	54.7	41.8	28.6	76.8	69.4	33.6	32.4
		K.FAAAEAEAANAPVSNGSSR.S	3	14.7	13.3	12.8	12.3	3.3	5.4	2.6	5.1
		K.GGLILDIGLR.G	2	194.1	142.3	110.2	74.8	217.3	165.8	117.6	67.2
		K.HDVDPNEVVSVGDEVEALVLTK.E	2	135.9	120.8	145.0	127.6	66.5	70.1	81.1	76.9
		K.HDVDPNEVVSVGDEVEALVLTK.E	3	1059.0	1165.5	940.7	1028.7	1281.8	972.2	1002.7	784.0
		K.VDRDEVLLDIGYK.T	2	485.5	508.4	623.2	626.3	519.7	563.4	545.1	610.5

		K.VDRDEVLLDIGYK.T	3	318.4	272.2	236.4	187.0	294.9	264.5	188.9	166.0
		K.YFNDGDIVEGTIVK.V	2	1861.4	1811.7	1681.5	1660.3	1860.8	1755.7	1532.3	1546.1
		R.AWGTIEELKEKDEAVK.G	2	57.1	53.0	202.8	213.7	125.4	162.0	187.6	402.1
		R.AWGTIEELKEKDEAVK.G	3	148.3	121.3	451.3	403.9	337.6	341.0	552.7	560.4
		R.GFLPASLVEM*R.R	2	155.7	144.2	84.9	75.8	131.6	125.0	45.8	39.5
		R.GFLPASLVEMR.R	2	643.6	583.8	464.4	409.7	426.8	350.2	295.7	229.2
		R.HVEVPDQVVQVGDDAM*VK.V	3	107.0	118.7	145.8	171.4	46.5	47.5	94.4	129.2
		R.HVEVPDQVVQVGDDAMVK.V	2	629.5	651.3	489.8	490.9	334.1	379.0	281.2	374.2
		R.HVEVPDQVVQVGDDAMVK.V	3	553.4	482.2	604.3	530.8	253.3	234.1	251.8	263.7
		R.SEESSGGTLASDAQLAALR.E	2	1313.8	1289.1	983.4	971.4	1225.6	1069.0	980.3	922.6
		R.VEEGIEGLVHISELSER.H	2	480.9	404.8	367.4	311.9	564.1	515.0	323.7	310.1
		R.VEEGIEGLVHISELSER.H	3	688.0	603.8	498.8	465.3	774.2	638.9	458.7	381.9
MSMEG_3839	MSMEG 3843	K.AADDTPTLMLLDGNSLAFR.A	2	37.6	46.7	34.1	33.7	34.2	37.9	35.7	49.8
		K.TGYTTDADALQSLFEK.T	2	85.8	85.6	90.9	92.0	107.7	79.2	127.8	107.4
		K.VTVDGLLNSVASDGR.I	2	130.2	161.7	76.9	94.6	119.5	113.3	83.0	81.0
		K.YGLTPQQYPDFAALR.G	2	194.3	178.3	234.2	175.4	179.5	150.0	270.3	159.4
		R.AALNAPIQGSAADIIK.V	2	175.5	166.2	212.6	218.9	168.9	184.3	203.3	229.9
		R.AC#AVLDLADALDQELAR.I	3	16.0	21.3	9.4	13.0	22.5	17.9	20.6	15.5
		R.AC*AVLDLADALDQELAR.I	2	98.6	92.6	46.2	45.3	127.3	96.4	77.8	54.0
		R.AFSVPIDEVTPELR.R	2	141.9	146.7	484.4	573.4	152.0	132.1	552.2	524.6
		R.AFYALPAENFK.T	2	54.1	44.0	48.9	38.4	59.1	47.6	58.3	54.8
		R.DAGLIEAFNTGEDLHSFVASR.A	3	117.9	105.4	102.7	85.5	148.2	84.2	151.5	92.0
		R.LFDDLEFR.V	2	42.6	37.0	20.3	17.4	44.7	42.9	35.9	24.6
		R.LSSTEPNLQNIPIR.T	2	203.3	205.1	169.3	180.7	187.4	215.1	208.9	224.7
		R.SFTLDDLAVR.Y	2	46.7	36.4	22.8	25.6	51.7	36.8	38.0	29.7

MSMEG _3859	MSMEG 3862	K.AGLGAAYLAGFDWGLR.R	2	16.9	19.6	33.8	35.6	15.6	15.1	19.4	16.7
MSMEG _3863	MSMEG 3866	K.SEAVEAHAEILDK.S	3	3.4	0.0	5.3	0.0	4.1	3.5	4.7	4.1
		K.YILLTTFTK.D	2	9.0	9.1	14.0	14.1	11.8	11.6	22.5	20.2
MSMEG _3864	MSMEG 3867	R.ALDGGFIPSGPSGSPLR.G	2	55.5	52.2	20.3	18.2	34.8	38.6	18.1	20.8
		R.GGQDAEWLTEEQLAR.N	2	24.3	22.8	0.8	2.0	13.1	15.3	1.4	1.4
		R.IGNAVGLDTPASAVALLR. A	2	52.4	47.5	85.6	99.2	35.2	33.5	84.1	80.6
		R.LAGTEC#EIDQVLR.A	2	19.2	21.1	2.6	2.9	11.5	12.9	1.3	3.4
		R.LVDGELEELLAGADIAVVR .I	2	9.9	9.4	7.5	6.0	6.7	6.8	2.0	3.8
		R.LVDGELEELLAGADIAVVR .I	3	6.4	7.0	0.3	0.5	4.1	4.4	0.5	0.7
MSMEG _3872	MSMEG 3875	R.LGGAVLAIGNAPTALFR.L	2	20.1	21.7	50.8	51.4	35.2	37.3	58.1	61.3
MSMEG _3881	MSMEG 3884	R.LTALVLPADGDPTIVVPR.L	2	148.5	84.2	166.2	112.7	126.0	126.6	194.0	196.1
		R.YALLQQAQQAAVAAVRP GVTAEQVDAAAR.D	3	156.1	149.1	178.0	185.3	121.4	121.6	185.4	188.1
MSMEG 3892	MSMEG 3892	R.PIGPAGAVQKPQDVNLR. D	3	22.1	19.6	7.5	8.0	8.9	11.4	1.7	3.9
MSMEG _3889	MSMEG 3893	R.TTVAGYADSPSDEAFSR. M	2	17.6	19.2	4.6	7.0	4.8	6.7	0.4	2.4
MSMEG _3895	MSMEG 3899	K.LYSQVSDADSALK.V	2	23.8	20.0	15.4	11.6	7.0	9.0	17.1	13.6
		K.VAVEALYDAADDDSATGG PDLVR.G	3	338.8	348.4	273.9	269.9	179.1	188.3	269.4	270.6
		R.GIYPTAVTIGAEGAEEVPE TR.I	2	204.1	243.9	126.9	138.7	129.3	133.2	101.6	135.9
		R.GNLGAALQGFVALPLLVG YDLDDPHPEGAGR.I	3	121.7	124.7	97.1	105.1	48.5	56.7	66.8	78.7
		R.LYAVELEHYEK.L	2	251.3	192.1	158.8	102.2	180.9	167.5	178.4	171.5
MSMEG _3897	MSMEG 3901	K.AHVGALLDSVDSAVELVE QLTN	2	28.2	30.6	41.2	59.7	19.7	27.8	22.9	39.0

		R.VGIGPSGDEPGFQLSQR.A	2	198.5	201.3	262.4	263.4	194.6	203.9	239.3	269.6
MSMEG_3902	MSMEG3906	K.AEVEDLVLEEVPDVSYNDIGGLGR.Q	3	339.9	425.5	89.6	129.0	152.0	212.2	59.3	94.8
		K.ASEGTPVIVFFDEMDSIFR.T	2	17.1	24.6	9.2	14.8	0.0	14.7	0.0	7.3
		K.DFNSGAMIQNVVDR.A	2	338.3	332.5	151.7	150.8	302.1	307.9	110.3	115.9
		K.DLPDEPTDYFDDSRPR.K	2	97.6	88.5	25.6	37.8	76.4	94.8	12.8	32.5
		K.IERPDAEAAQDIFSK.Y	2	62.9	65.6	76.7	72.9	40.7	49.7	75.3	75.9
		K.IKIERPDAEAAQDIFSK.Y	2	20.5	24.1	0.9	1.7	22.5	31.5	1.4	2.2
		K.IKIERPDAEAAQDIFSK.Y	3	132.5	132.6	65.3	79.9	125.6	128.6	80.9	85.9
		K.IKIERPDAEAAQDIFSK.Y	4	57.4	51.8	17.2	14.3	63.5	76.5	18.9	17.8
		K.YLTEDLPVHADDLTEFNGDR.A	3	386.4	430.7	252.5	250.8	341.5	331.9	199.1	204.6
		R.DAVELPFLHK.D	2	91.9	75.9	30.5	26.2	104.8	84.1	31.0	30.6
		R.EQLENAVGPQSGLR.S	2	9.7	9.1	27.2	25.8	6.9	9.4	18.4	26.6
		R.IVWLAEPLVAAK.D	2	333.8	320.5	179.7	166.2	400.2	295.1	203.8	160.1
MSMEG_3906	MSMEG3910	R.GIIAFDDVIGLPEGSVVK.S	2	54.9	46.7	84.4	86.2	88.4	85.6	100.3	93.6
		R.HYTMVLNHGGEFHTHR.G	4	5.0	4.2	4.6	3.7	6.3	8.1	4.0	5.5
MSMEG_3918	MSMEG3923	R.ILELLARFTDDAVAADRIMR.L	3	6.7	6.9	75.7	77.1	10.6	16.7	64.2	81.0
MSMEG_3932	MSMEG3937	R.SVTLPPGADEDAIK.A	2	168.0	149.7	3.9	3.7	10.8	13.7	3.5	4.6
MSMEG_3945	MSMEG3950	K.ALEFAFDAASR.R	2	30.4	28.2	0.3	0.3	3.4	2.2	0.7	0.2
		K.ASAIATTAPVVVGVDGSPASEK.A	2	130.3	131.1	2.5	3.2	27.8	34.2	0.9	2.5
		R.GGFAGLLLGSVSTSVAEAAERPVVVVR.G	4	31.0	34.6	0.1	0.3	2.9	3.6	0.0	0.0
		R.GVLVGVDGSEPSTAAVEWAAK.E	2	88.5	89.3	30.0	29.2	18.8	22.5	37.8	45.9
		R.LLGSVTAGLIR.H	2	93.5	94.5	0.5	0.2	22.8	21.7	0.6	0.5
MSMEG_3950	MSMEG3957	K.VVADC#LGETHGLTVQTEIR.K	3	300.8	257.2	11.7	6.2	94.2	96.1	9.2	11.4

		R.GGFTGMLLGSVSSR.V	2	88.0	89.5	5.6	6.6	18.1	18.4	5.2	7.8
		R.LPVLLGIDGSPASEVATSHAFDEASR.R	3	968.8	1006.7	104.4	100.2	334.3	341.3	101.6	98.9
		R.LPVLLGIDGSPASEVATSHAFDEASRR.G	4	30.1	49.1	20.4	15.6	23.9	30.9	22.2	12.9
		R.VLLGSTSTSLLHYASGPVVVVHGDDQAAHDSR.L	5	333.4	348.2	10.6	15.8	102.3	97.3	7.8	12.0
MSMEG _3962	MSMEG 3970	K.ASGDTPVLFDSGIR.T	2	140.6	149.1	189.9	146.9	478.6	514.7	378.8	405.7
		K.HAGDTPAYFQLYYPEDR.D	2	46.6	75.9	423.9	566.9	366.5	417.5	294.8	384.8
		R.DLSVELWGK.T	2	4.2	5.0	93.3	84.7	153.5	144.9	89.5	87.1
		R.DLTISNFPFLR.G	2	150.7	153.7	729.0	676.4	786.3	844.0	711.1	735.4
		R.GLC*LTNYVTDPVFQK.K	2	106.5	98.9	808.2	842.6	776.4	687.6	912.2	886.0
		R.SLLAEADLIMAVDGYR.N	2	21.1	25.1	163.9	154.7	99.2	95.9	174.5	162.2
		R.TGVPYITSTLAVSSLEDIR.K	2	44.5	46.1	299.2	293.6	240.1	205.0	250.3	222.0
		R.TGVPYITSTLAVSSLEDIRK.H	2	6.4	4.6	245.2	200.6	94.0	94.9	363.2	114.6
MSMEG _3980	MSMEG 3987	R.SLIDYLTEFAYASVK.V	2	10.7	12.9	13.7	10.5	8.0	10.4	8.5	8.5
MSMEG _4042	MSMEG 4047	R.EDEVTAEAGVSR.T	2	2.7	1.9	1.3	0.9	3.9	2.4	2.0	1.1
		R.LQSEGLVQLVR.H	2	104.1	102.6	148.7	142.1	137.0	114.5	129.3	154.0
MSMEG _4075	MSMEG 4082	K.LVSDAAQARAAAESLGYPVVVK.A	3	1.8	2.1	46.5	29.2	10.8	12.1	64.3	61.0
MSMEG _4125	MSMEG 4132	R.AEWSELFQLK.L	2	28.5	27.3	16.1	12.0	39.2	34.0	19.9	18.9
MSMEG _4180	MSMEG 4185	K.GALSESAAEILAEAGYR.R	2	75.8	87.4	52.2	57.2	66.9	72.2	50.0	61.3
		R.IATSFPNLVR.K	2	48.5	50.0	47.4	43.8	44.4	44.1	48.8	46.8
MSMEG _4185	MSMEG 4189	K.AAIFAIETLFEER.G	2	6.8	5.2	1.2	2.2	25.0	13.7	4.9	3.3
		K.AVAYLLPYIEAEK.K	2	92.9	81.8	419.6	214.3	63.0	59.8	86.7	75.5
		K.ETGLADYIGAFAVTAGLGSAEK.I	2	10.4	11.0	2.2	3.3	14.8	13.3	2.4	2.6

		K.ETGLADYIGAFAVTAGLG SAEK.I	3	2.4	3.2	0.7	1.7	3.8	4.1	0.7	1.2
		R.EFENYDLSELR.Q	2	66.8	66.9	13.7	18.8	60.1	56.9	12.5	12.7
		R.EGMDIPLLIGGATTSR.A	2	38.2	36.4	11.6	10.4	33.5	35.4	5.4	9.7
		R.LSGLEPLNIDEDSLFVNIG ER.T	3	5.7	10.1	21.6	21.4	5.2	9.8	5.8	6.4
		R.NVSYDQLAEAYLEAAR.G	2	49.1	44.1	13.4	10.9	48.3	48.5	11.3	11.0
		R.TPIDWDGYTPPVPAQGLG IR.E	2	30.6	25.9	2.3	4.2	27.3	32.0	2.2	5.2
		R.WPVIISGTITDASGR.T	2	19.9	23.1	17.5	27.4	29.4	32.3	11.0	22.5
MSMEG _4188	MSMEG 4192	K.AAVEQFANALR.L	2	23.4	12.5	57.4	39.0	19.0	22.8	47.1	59.4
		K.ADLSTFGEMLSK.L	2	28.1	32.1	15.5	20.5	25.9	27.7	17.3	18.2
		K.LMLVDVDADALK.A	2	30.9	33.3	51.0	59.5	32.8	28.8	27.6	35.1
MSMEG _4189	MSMEG 4193	R.SATALPEAPDATDVIAR.V	2	248.6	269.5	111.2	130.9	181.1	191.1	120.1	128.0
		R.VLPPHDYVAATDAIAEVVE MVEK.L	3	118.8	127.4	72.1	76.2	65.4	72.0	41.6	56.3
MSMEG _4193	MSMEG 4198	K.AVLADALGVHLDGFQR.I	3	28.9	30.6	17.4	14.8	17.3	11.9	5.4	7.7
		R.LTEVDYGSWTGR.K	2	71.0	60.2	89.9	63.6	49.5	52.4	79.2	83.0
MSMEG _4199	MSMEG 4204	K.LDLPENATPAYLGFNLFG HAIAR.A	3	97.1	92.8	83.5	74.3	82.9	82.7	89.5	88.6
		R.YYIAVHAVDVEK.L	2	48.2	52.6	121.4	135.6	32.6	28.4	88.4	113.1
		R.YYIAVHAVDVEK.L	3	0.0	0.0	0.0	0.0	0.0	0.0	0.0	0.0
MSMEG _4205	MSMEG 4210	R.AC*IKLGVADRDELAR.M	2	60.5	64.0	90.9	100.8	35.6	37.5	71.3	81.1
MSMEG _4207	MSMEG 4213	-.MIVVGYSADPFGR.A	2	0.4	0.4	73.1	71.9	0.2	0.9	83.1	93.8
		R.DTGLLVINATAGDAYVDA R.F	2	18.3	23.7	103.2	103.4	7.2	12.1	113.6	108.0
		R.LLLEC*PKPVLAVKPHGF	3	11.6	10.0	181.7	169.4	22.0	20.0	206.0	186.6
		R.SGEVHDVEAHLQDSGVP FEIR.Q	3	9.8	11.4	662.7	737.9	3.0	9.8	616.9	594.5

MSMEG _4208	MSMEG 4214	R.GTAFGFPFGALPAGGAEL PTFLSYITEK.R	3	36.9	39.0	83.3	129.4	6.2	29.1	18.7	77.7
MSMEG _4210	MSMEG 4216	K.IAAGTTGLGEFVDQIEAGQ VR.V	2	18.2	22.3	524.4	552.0	32.9	56.2	471.1	466.3
		K.IAAGTTGLGEFVDQIEAGQ VR.V	3	7.9	6.9	358.8	381.1	5.1	1.8	311.5	321.2
		K.MVEEQEGILVPGDSPFR.T	3	41.8	42.2	26.3	35.9	38.8	38.6	14.1	16.7
		K.NGWSDAFTTGPEFEQFL K.D	2	8.0	7.6	146.2	163.7	6.7	6.0	88.2	114.1
		K.VSIGGGSSPGGPDHLFPM ETAR.A	3	11.4	19.0	909.5	1033.2	14.8	24.9	839.1	857.6
		R.DAMVAALTELHDTDEWK. E	2	26.0	22.6	174.9	185.0	22.8	22.9	78.6	106.2
		R.DAMVAALTELHDTDEWK. E	3	0.7	0.4	206.3	203.3	0.7	0.3	137.6	128.5
		R.DAMVAALTELHDTDEWK EAMVK.N	3	97.0	114.1	102.5	105.5	90.9	90.2	173.3	186.1
		R.IEVFNVIGAGGTVAMAR.L	2	11.7	16.7	287.9	242.1	10.4	9.0	295.2	279.4
		R.M#MVPNSPGGGYDLTAR. T	2	9.9	11.8	125.8	131.1	4.8	5.5	84.0	99.4
		R.MM#VPNSPGGGYDLTAR. T	2	9.8	11.8	124.1	130.1	4.8	5.4	82.0	98.3
		R.MMVPNSPGGGYDLTAR.T	2	36.8	40.3	603.8	576.1	48.2	50.1	536.7	552.2
		R.TIDDLVAAWK.A	2	0.3	0.1	318.5	214.3	0.7	0.6	297.1	289.8
		R.TVNYITYDGGGDLLTALLG K.K	2	34.9	42.1	222.6	216.0	8.6	17.6	212.6	199.6
		R.VPGVDAPTLTEAGIDMTF TNWR.G	2	2.8	2.6	175.0	197.6	1.7	5.5	141.2	157.0
		R.VPGVDAPTLTEAGIDMTF TNWR.G	3	49.4	51.7	119.7	128.9	51.1	58.8	78.1	92.6
MSMEG _4217	MSMEG 4223	K.TYLESQLEELGQR.G	2	354.1	363.4	568.7	573.9	344.1	323.1	541.3	510.1
		R.AQAEAMVSDAR.Q	2	5.7	3.7	7.7	4.9	1.8	1.1	1.4	0.7

		R.GSAAPVDSSANSDASGF GQFNR.G	2	201.5	135.5	316.9	196.2	260.9	256.2	317.0	368.3
		R.GSAAPVDSSANSDASGF GQFNR.G	3	101.2	84.3	172.9	125.0	100.1	141.9	141.4	228.9
		R.GYNEDEVDAFLDLVENEL TR.L	2	82.0	97.9	122.7	163.9	24.3	43.5	38.3	67.8
		R.GYNEDEVDAFLDLVENEL TR.L	3	33.7	49.1	53.6	77.9	11.4	22.6	15.5	34.3
		R.LIEENADLR.Q	2	10.5	8.1	11.0	9.4	6.8	5.7	6.8	5.5
MSMEG _4222	MSMEG 4228	K.AAEDAKDDIEELLR.G	2	58.8	64.8	221.8	228.0	56.5	54.4	253.9	273.6
		R.KAAEDAKDDIEELLR.G	3	11.3	8.4	12.6	12.4	13.8	12.6	24.7	19.1
		R.LLQMGDAAVSLMDAFR.S	2	106.2	104.2	95.6	91.2	39.4	32.4	41.9	45.3
MSMEG _4237	MSMEG 4241	R.MLDQIESALYAEDPK.F	2	27.3	28.2	12.6	15.6	14.5	19.6	12.1	16.6
MSMEG _4240	MSMEG 4244	R.DALDVLAAANVHPQAK.A	3	39.6	33.2	17.9	17.2	36.9	41.8	12.1	11.9
		R.PLQLGAAAAADRPDVQEL FHR.I	4	98.9	89.6	79.8	62.8	84.8	81.8	66.7	64.8
		R.SVIEPADAVAGPDVLR.L	2	320.3	355.3	360.4	381.8	227.7	209.4	320.0	327.2
		R.TVLLAEALELADASDPAAA ALLR.R	2	82.8	78.6	75.7	74.1	55.2	75.5	44.3	70.5
		R.TVLLAEALELADASDPAAA ALLR.R	3	60.3	62.3	47.0	63.6	59.3	62.0	53.3	53.0
MSMEG _4244	MSMEG 4247	K.LGPTTTPELAVEYVER.L	2	364.5	351.8	262.4	271.1	327.3	331.6	276.4	284.2
MSMEG _4251	MSMEG 4254	R.TSVTWSLVSSSLLR.S	2	16.6	20.7	22.2	28.1	16.9	11.6	28.3	21.4
MSMEG _4254	MSMEG 4256	K.GC*QLTHSNLLHEIR.G	3	108.2	93.6	161.2	109.4	57.9	53.4	113.9	73.6
		K.SSDPATLIYTSGTTGQPK. G	2	190.8	161.2	149.1	138.9	89.0	94.2	102.0	117.7
		K.VTLGFTSDIK.N	2	23.4	18.2	26.8	11.3	15.8	12.0	15.9	8.2
		R.AALGGEC#HAAISGGAPL GER.L	3	98.6	84.9	107.0	108.0	82.8	79.6	67.3	53.7

		R.AALGGEC*HAAISGGAPL GER.L	3	98.6	84.9	107.0	108.0	82.8	79.6	67.3	53.7
		R.ILPVDFTEDTGELTPTLK.V	2	570.0	588.6	280.4	274.5	353.1	389.0	180.4	177.7
		R.ILVFLPLAHVLAR.A	3	8.5	4.6	6.9	3.5	16.8	8.1	18.1	4.8
		R.SAALGLIAEGVQPGDR.V	2	187.0	169.4	352.0	332.1	175.5	194.1	376.6	424.0
MSMEG _4258	MSMEG 4262	R.AASSLSGGADTLEALGVR .I	2	276.2	245.9	189.6	213.4	207.8	187.7	280.4	206.8
		R.AVEAIDSGAAEQLLAR.W	2	326.4	335.4	212.7	212.4	278.3	258.7	218.7	245.9
		R.LTFDPAAFGFK.R	2	92.4	82.1	66.9	60.0	69.3	60.9	80.4	72.3
MSMEG _4262	MSMEG 4265	K.AVLWTSGWTPR.F	2	103.6	94.8	21.1	10.7	66.5	76.8	8.2	12.9
		K.TVAANLTDALEGSTLK.R	2	395.8	361.7	242.4	238.5	307.2	308.6	191.6	205.1
		K.VC#SHLGC#PSSLYEQQT YR.I	3	113.4	69.9	104.1	22.1	200.5	240.7	219.8	125.0
		K.VCSHLGCPSSLYEQQTYR .I	3	112.3	71.1	100.6	23.9	197.3	239.9	214.8	124.9
		R.ILC*PC*HQSQFDALEFAK. P	3	182.9	185.1	74.1	94.1	149.7	176.2	55.9	80.8
MSMEG _4263	MSMEG 4266	R.SDREVLEHGVETGIIK.R	3	43.7	44.5	45.3	41.4	52.7	48.2	83.7	73.0
		R.SDREVLEHGVETGIIK.R	4	4.0	0.2	0.0	0.2	16.7	13.2	0.4	0.7
MSMEG _4268	MSMEG 4271	K.IETLGTSSEIPVLVLPAGK. R	2	98.6	103.3	26.7	30.9	41.7	40.9	44.4	53.2
MSMEG _4269	MSMEG 4272	R.EPLSLRPFDYLPR.P	3	25.7	25.8	8.3	6.1	21.1	16.2	4.5	5.9
		R.LGLDLGIDQR.A	2	28.1	30.3	10.0	14.6	22.9	18.3	16.6	13.5
		R.VPFLDPEVFAVASR.L	2	104.4	96.3	161.7	156.7	112.1	74.3	221.8	145.9
		R.YDEITAALEDSVAK.H	2	56.6	71.4	58.1	60.5	62.6	54.1	122.0	115.2
MSMEG _4270	MSMEG 4273	K.FSEQLLADHLQK.V	2	73.9	56.2	43.2	51.8	36.2	37.1	23.9	42.6
		K.FSEQLLADHLQK.V	3	144.9	115.3	16.5	15.8	125.6	110.8	22.1	16.6
		K.LGLAFAADPSQQLAR.L	2	1083.1	1129.9	681.7	710.1	1417.2	1408.9	679.4	669.4
		K.VSLSFLVDDLVIHR.G	3	42.8	38.9	5.8	4.2	15.5	15.8	0.7	1.5

		R.DGTFVHVDVVPETHKEDPTGIGDAFR.A	4	167.0	149.0	212.7	204.1	187.1	172.6	189.6	213.1
MSMEG_4272	MSMEG 4275	R.M*SAPYVQGATIDFVDTIEK.Q	2	26.3	26.0	1.1	1.9	11.4	11.0	0.4	0.6
		R.MSAPYVQGATIDFVDTIEK.Q	2	33.7	33.7	2.8	5.3	20.7	19.0	5.6	4.2
		R.TLDGDLTAEFGGVTLTVDR.M	2	57.1	61.8	3.7	11.8	41.6	48.1	4.5	6.5
		R.YNLFFDDR.T	2	58.0	56.2	7.3	6.8	38.7	39.6	3.3	4.3
MSMEG_4273	MSMEG 4276	K.NNDDDKNVASDAADSAVDEQNGPAAEDRPR.T	4	13.7	17.2	7.8	10.0	13.1	17.2	6.7	10.2
MSMEG_4276	MSMEG 4280	K.GGIKPVSVWLSHEYVR.A	3	94.6	98.6	159.6	141.2	51.7	58.2	136.0	167.2
		R.LAIPELPEEVFIESLR.Q	2	505.0	445.9	494.7	459.0	303.1	293.9	419.2	379.7
		R.LAIPELPEEVFIESLR.Q	3	40.5	42.9	38.5	49.6	26.5	24.9	44.7	31.9
		R.LVTPELSGSLLPGITR.D	2	460.9	414.1	481.3	452.7	373.3	350.0	469.6	433.7
		R.RLAIPELPEEVFIESLR.Q	3	14.6	13.8	11.9	8.7	32.9	27.2	35.5	23.7
		R.YLLIASPAGAYFK.G	2	78.1	66.7	137.0	146.4	65.9	41.8	79.8	195.0
MSMEG_4278	MSMEG 4281	K.GPGAAAYVNSALTNDLGK.I	2	269.4	260.1	288.7	295.7	126.4	114.1	132.4	140.2
		R.AGVVFDALVAAVR.A	2	75.1	62.2	30.3	26.7	37.1	32.8	23.2	25.2
		R.STVGLFDVSHLGK.A	2	109.7	92.5	170.4	169.2	73.5	71.6	147.5	175.0
		R.VGVTTSGTFSPTLK.V	2	265.5	242.7	211.5	235.9	123.1	121.6	189.4	214.5
MSMEG_4281	MSMEG 4283	K.GITFDTGGISIK.P	2	82.0	77.7	190.4	137.2	55.8	59.5	94.1	109.3
		K.GPGLTAITVLAHDTK.A	2	303.4	237.9	140.4	137.6	138.1	141.9	276.8	285.9
		K.GPGLTAITVLAHDTK.A	3	395.6	269.5	123.0	71.1	205.3	184.6	206.7	159.8
		K.LLTPAPFFDATVVTAIESGLR.A	2	596.9	640.4	237.9	239.1	168.9	255.4	181.8	277.4
		K.LLTPAPFFDATVVTAIESGLR.A	3	333.1	379.9	134.8	159.5	138.3	158.4	143.8	190.8
		K.LSQDVGENGWPMPLPEELRDDLK.S	3	447.8	423.8	122.6	127.1	163.3	139.4	56.8	64.9
		R.DFVNTPPSHLFPDEFAK.R	2	531.6	617.9	153.7	177.4	208.0	225.3	160.8	185.0
		R.FAGMLVAGTYLR.E	2	124.9	103.9	16.6	13.6	22.4	32.1	14.8	24.2
		R.GVDIATAVATAR.D	2	59.0	42.2	17.8	17.2	15.8	12.1	111.9	130.9

		R.LILADAIVR.A	2	62.1	41.9	30.8	23.8	28.7	20.5	15.8	12.2
		R.LVVDALPVGSVLAVGLGK.D	2	1705.9	1623.2	699.8	653.4	678.4	581.7	707.7	608.2
		R.LVVDALPVGSVLAVGLGK.D	3	146.0	152.3	28.7	28.5	63.3	49.5	39.9	32.3
MSMEG_4283	MSMEG4286	K.AEPAPAASSDGSPYVTPLVR.K	2	1581.2	1581.6	2298.1	2277.0	1669.5	1927.3	2454.4	2972.1
		K.AEPAPAASSDGSPYVTPLVR.K	3	258.3	364.5	481.3	567.4	262.4	370.8	580.1	638.3
		K.IGDAGAAAEPKPEPKPEPK.A	3	0.2	0.2	0.1	0.1	0.0	0.0	0.0	0.0
		K.IGDAGAAAEPKPEPKPEPK.A	4	1.3	1.4	0.9	1.1	1.0	1.7	0.7	1.8
		K.IHPNVNASYNEDTK.E	2	2.0	0.7	2.3	0.7	3.0	2.1	4.3	2.9
		K.IHPNVNASYNEDTK.E	3	9.4	5.8	11.5	7.4	8.3	8.8	11.2	11.3
		K.LAAENGIDLSTVK.G	2	138.7	112.2	275.3	251.1	126.3	98.8	282.0	197.7
		K.QAPAASPAAAAPAAPAAASATPAPALAHLR.G	3	474.5	467.4	662.5	642.0	588.8	741.4	935.7	1100.7
		R.EGVNLTFLPFIAR.A	2	70.1	76.1	99.7	110.8	368.3	312.1	525.8	484.0
		R.ESLQATAQLTQTHEVDM#TK.I	3	245.0	231.8	358.2	310.7	203.5	185.2	305.0	264.4
		R.ESLQATAQLTQTHEVDM*TK.I	3	242.9	230.0	357.4	309.6	200.7	183.5	302.5	263.7
		R.ESLQATAQLTQTHEVDMTK.I	2	87.2	80.8	95.4	81.8	71.2	108.8	103.0	138.1
		R.ESLQATAQLTQTHEVDMTK.I	3	838.1	813.0	1122.8	1173.7	705.7	846.3	924.5	1261.3
		R.KLAAENGIDLSTVK.G	2	29.6	27.6	305.0	267.2	55.1	37.6	443.2	326.9
		R.LEEGAFEADLGL	2	7.0	10.6	15.0	19.4	21.7	6.4	27.5	18.1
		R.RLEEGAFEADLGL	2	366.4	370.9	791.3	779.0	536.1	402.8	1207.5	903.3
		R.SVC#YLPLTYDHR.L	2	453.1	416.6	592.0	513.4	449.3	477.5	702.7	679.3
		R.SVC*YLPLTYDHR.L	2	452.9	416.6	542.7	466.7	449.3	477.5	694.9	679.3
		R.VIVDEYGNESIGVR.S	2	499.8	461.7	803.9	724.1	633.7	580.4	1056.6	957.3
MSMEG_4287	MSMEG4290	R.VIGRPGVIFVGEGAPNR.V	3	47.5	43.4	29.1	17.6	72.9	75.7	31.3	38.1
MSMEG_4288	MSMEG4292	R.LGLPEDGPGSIAR.T	2	13.7	15.4	6.3	7.0	14.6	20.8	2.6	7.6

MSMEG _4290	MSMEG 4293	K.DGQPLFHDESGYAGLSDI AR.H	2	546.6	628.0	2076.3	2438.5	429.4	496.6	2056.0	2296.7
		K.DGQPLFHDESGYAGLSDI AR.H	3	1281.9	1288.9	5231.3	5510.4	978.5	980.5	4446.5	4487.0
		K.FNTLLAAADDVLLFK.Y	2	452.3	449.3	1924.0	1900.7	475.5	406.0	2028.8	1721.5
		K.FNTLLAAADDVLLFK.Y	3	138.1	179.5	490.6	602.8	193.3	175.4	726.5	687.2
		K.GGYFPVAPYDHYVDLR.D	2	883.4	975.3	2947.7	3292.6	731.6	766.4	2940.5	3067.4
		K.LIKDENVEYVDIR.F	2	474.2	478.9	1675.7	1727.2	505.2	452.1	2068.2	1901.2
		K.LIKDENVEYVDIR.F	3	719.0	736.2	2386.3	2504.4	737.7	693.8	2747.0	2595.3
		K.PLFGDNGSGMHAHQSLW K.D	2	44.5	56.8	142.1	178.2	15.5	22.3	117.4	128.8
		K.PLFGDNGSGMHAHQSLW K.D	3	191.2	167.8	475.9	439.1	83.1	106.4	287.3	373.5
		K.PLFGDNGSGMHAHQSLW K.D	4	196.2	193.9	716.2	671.5	137.8	140.5	639.7	616.5
		K.TLNM#NFFVHDPFTR.E	3	70.7	75.3	154.3	148.7	39.0	42.9	143.3	147.6
		K.TLNMNFFVHDPFTR.E	2	1121.5	1090.0	834.2	844.4	271.1	271.1	712.9	639.6
		K.TLNMNFFVHDPFTR.E	3	30.6	22.5	10.8	12.7	1.9	0.7	16.4	16.5
		R.DQM#ATNLQNAGFTLER. G	2	235.5	265.1	404.4	418.8	141.1	180.1	444.6	426.3
		R.DQM*ATNLQNAGFTLER. G	2	432.4	462.7	1274.3	1222.9	284.5	334.5	981.3	1012.6
		R.DQMATNLQNAGFTLER.G	2	1008.4	1006.7	3346.9	3269.1	601.7	574.9	2193.4	2155.9
		R.DQMATNLQNAGFTLER.G	3	36.6	40.0	133.7	123.7	14.8	18.4	81.1	100.3
		R.GFQSIHESDMMLLPDPNT AR.I	2	96.1	124.0	308.6	368.5	6.1	12.2	66.7	105.0
		R.GFQSIHESDMMLLPDPNT AR.I	3	499.0	456.6	1837.3	1755.9	180.9	188.1	876.4	863.9
		R.GHHEVGTAGQAEINYK.F	2	25.9	14.3	86.3	51.6	26.2	16.9	106.5	72.8
		R.GHHEVGTAGQAEINYK.F	3	202.0	157.4	565.5	466.3	166.5	163.9	531.0	556.8
		R.LVPGYEAPINLVYSQR.N	2	2293.8	2357.9	8525.9	8595.4	1951.0	2008.7	8551.6	8802.9
		R.LVPGYEAPINLVYSQR.N	3	507.3	484.9	1640.8	1647.5	491.0	477.3	1921.8	1841.6

		R.PHPYEFSLYYDV	2	225.2	235.0	1300.0	1506.7	183.1	186.7	1365.2	1307.1
MSMEG _4291	MSMEG 4294	R.RPSWNAAAPGSVAEPLV TEFTEALR.R	3	29.1	12.0	48.0	16.2	21.8	23.9	37.2	39.8
MSMEG _4293	MSMEG 4296	R.APEVIQMYADGPSGPK.L	2	16.8	27.8	84.5	100.6	16.3	22.5	84.2	92.7
MSMEG _4294	MSMEG 4297	K.SFIAGILEHANEISAVTNQ WVNSYK.R	3	97.1	128.8	249.4	314.1	55.3	103.5	176.0	275.1
		R.LWFTDVLGYLK.S	2	122.2	115.8	254.0	253.4	166.6	151.1	344.6	314.9
		R.VFESDTVARPDPSTFQVL PWK.T	3	195.0	182.6	433.7	406.0	320.8	306.8	679.3	659.4
		R.YADALSMADNVMTFR.Y	2	49.9	48.0	127.5	124.9	16.4	15.6	30.5	26.8
MSMEG _4298	MSMEG 4301	K.GAPHALVIGDLPFGSYED GPSQALATATR.F	3	1834.9	1352.4	109.8	140.5	8312.1	8667.8	75.0	104.6
		K.GAPHALVIGDLPFGSYED GPSQALATATR.F	4	171.1	157.3	100.7	74.7	632.4	642.3	176.9	115.3
		K.WAM*LTC#YDYSSAR.V	2	19.5	22.5	2.4	1.9	91.3	108.6	4.6	2.5
		K.WAMLTC#YDYSSAR.V	2	236.4	268.4	6.5	6.8	721.8	815.2	17.3	15.4
		R.AATQFADDVAAGVFPAEE HSY	2	1209.1	1289.8	1.2	1.2	4244.8	4475.2	2.5	1.0
		R.AATQFADDVAAGVFPAEE HSY	3	1516.7	1630.8	27.8	31.7	5285.7	5456.9	91.9	47.5
		R.FGDVGGELHR.A	2	1.6	1.8	1.2	0.4	7.3	5.8	1.5	1.0
		R.RFGDVGGELHR.A	3	15.4	13.1	0.1	0.0	25.7	21.3	0.0	0.0
		R.VADQIATLSR.A	2	6.9	4.6	2.2	1.6	29.9	38.1	2.0	2.3
MSMEG _4299	MSMEG 4302	K.VGLSPDGSASYYLPR.L	2	160.7	169.6	47.2	45.6	129.8	138.6	47.7	46.3
		R.ITLNRPDAANGMNDTMTR .E	3	50.5	52.0	16.4	18.9	28.7	37.2	6.6	14.3
MSMEG _4301	MSMEG 4304	K.ELGASDVPTFAAAPNFAY ELAAHR.G	3	205.3	213.9	163.1	183.9	215.4	206.1	118.7	135.8
		K.GYWGRPHETEVSFHNK.L	4	12.3	11.7	6.8	7.5	8.8	11.7	2.2	4.7
		R.LPQGSHAAGAPDGAAWF R.T	3	127.3	117.3	17.6	20.4	112.3	110.3	8.9	13.7
MSMEG _4302	MSMEG 4305	R.ALASAPGAPSDTPEPAQP TDALHR.A	3	124.6	127.3	182.0	190.6	94.1	111.1	183.4	206.3

MSMEG _4306	MSMEG 4311	R.DALVNAIDDQLVELYEK.Q	2	26.0	31.2	25.0	24.9	30.6	35.1	29.6	31.0
		R.LAALGIALEDLDAQVAK.Y	2	28.1	26.5	40.4	43.8	46.8	41.6	43.5	43.8
MSMEG _4313	MSMEG 4318	R.ALIGADPVLDER.T	2	247.7	233.0	208.0	210.2	225.5	214.8	227.2	209.4
		R.DLAVSGPWYR.A	2	74.2	81.4	59.5	64.7	57.3	69.3	64.8	66.7
		R.LDELGIAHGGIVDAPYGS GLSFR.D	3	538.8	583.9	449.8	474.6	443.9	463.4	464.9	444.1
		R.TDAGFHHIVWALPGGTLF GIHQHDK.R	4	47.2	0.0	30.1	0.0	27.4	31.6	21.6	27.6
		R.VGLDHVSFGVGSR.D	2	45.1	43.2	54.0	57.1	28.2	30.3	29.4	44.9
		R.VGLDHVSFGVGSR.D	3	147.4	149.2	81.5	80.3	117.0	127.4	79.2	85.0
MSMEG _4320	MSMEG 4325	K.VWTAALQALR.A	2	19.3	0.0	97.0	0.0	18.4	21.3	55.5	57.9
		R.DYGVFNEDAGYPNR.G	2	31.4	33.5	30.8	37.0	22.5	23.7	13.6	21.1
MSMEG _4321	MSMEG 4326	R.LMDAITPLAEDGVIWVLTP K.T	2	36.8	43.2	60.9	73.2	29.7	35.8	62.1	74.1
MSMEG _4323	MSMEG 4328	K.ALVADM#SDQEIWNLK.R	2	25.3	22.9	239.9	210.2	10.1	8.2	324.4	245.0
		K.ALVADMSDQEIWNLK.R	2	150.7	138.7	248.5	230.7	109.4	93.3	267.3	213.3
		K.ALVADMSDQEIWNLKR.G	3	26.3	26.7	38.2	36.5	60.2	47.1	69.4	63.1
		K.DTSDQHVWAFLGDGEMD EPESR.G	3	370.2	399.6	215.8	227.8	312.3	299.7	131.2	186.2
		K.IIQELESFFR.G	2	368.3	360.7	604.2	545.9	368.3	285.3	770.7	554.5
		K.SAQILASGVAM*PEALR.A	2	392.5	411.8	516.6	513.6	330.8	344.8	538.0	536.1
		K.SAQILASGVAMPEALR.A	2	521.2	541.3	644.0	634.1	486.4	500.5	657.2	638.8
		K.SHPGGGDHVFIQGHASP GIYAR.A	3	33.8	27.1	78.2	70.8	35.0	45.6	72.5	87.1
		K.SHPGGGDHVFIQGHASP GIYAR.A	4	150.7	156.1	187.0	191.0	120.9	174.4	188.7	264.9
		R.AVPEQIRPWVPGTYVTLG TDGFGFSDTRPAAR.R	4	407.6	455.4	689.4	731.3	276.4	444.9	671.5	977.0
		R.EWDVLLHADR.D	2	81.0	78.1	94.2	88.2	60.4	51.8	84.9	78.9

		R.IDDVSAAGVSYADTGSA	2	55.2	82.0	111.6	170.7	31.8	50.9	88.6	138.5
		R.LTTDQLDGFR.Q	2	21.3	19.4	33.2	27.9	14.0	10.8	22.0	22.0
		R.TFGMDSWFPSLK.I	2	150.3	159.3	205.8	214.3	101.7	106.7	197.3	202.1
		R.TGDGLWAAADQMAR.G	2	528.1	539.1	496.0	509.6	484.3	461.1	348.3	347.8
		R.VPVSDAQLEEDPYLPPYY HPGPEAPEIR.Y	3	702.3	747.0	1029.1	1104.4	523.2	564.5	879.5	1083.4
		R.YFNTDAESVVVAVLQGLA R.D	2	80.9	95.8	103.7	113.5	45.8	83.4	87.2	128.0
		R.YFNTDAESVVVAVLQGLA R.D	3	73.5	96.9	119.9	161.4	55.5	68.9	120.0	145.6
MSMEG _4325	MSMEG 4330	R.LEALDLVPANR.N	2	206.3	215.5	97.9	95.2	245.1	225.4	78.1	66.2
		R.NAAGQIVAAGAVAALDK.L	2	321.0	328.7	381.2	396.0	338.8	320.6	546.9	500.5
		R.NAAGQIVAAGAVAALDK.L	3	31.3	33.9	11.4	11.4	47.0	39.0	6.6	7.5
		R.WDLC#TATLR.D	2	30.8	35.7	14.6	13.0	28.0	30.2	5.3	9.6
MSMEG _4326	MSMEG 4331	K.IPDEDLAGLR.T	2	184.7	155.3	195.0	173.4	202.7	249.3	249.2	286.4
		K.LEEENPEAAAALR.E	2	96.6	86.5	117.2	102.8	104.5	122.1	141.7	147.8
		R.TVGDVVAYIQK.L	2	727.1	824.0	814.6	950.7	731.2	746.7	976.0	936.2
		R.TVGDVVAYIQK.L	3	0.0	0.0	17.4	14.8	0.0	0.0	25.1	20.2
MSMEG _4328	MSMEG 4334	K.GALGHSVGAVGAVESILT VLALR.D	3	6.0	8.8	9.1	10.9	0.3	0.8	0.2	0.7
		R.AIELAGLKPTDIDHVNAHA TGTNVGDVAESK.A	4	688.6	872.7	590.3	602.1	667.3	669.0	663.2	638.9
		R.IGGHLLEEFDSELTR.V	2	134.2	127.3	135.2	141.1	68.4	88.5	128.7	148.4
		R.IGGHLLEEFDSELTR.V	3	64.5	48.1	46.8	33.0	42.9	57.3	47.6	55.9
		R.NLNNLDPEIDLDVVAGEP R.Q	2	183.8	192.9	235.9	272.2	151.8	144.7	204.2	225.3
		R.TLTDPFVEEYNLPVR.I	2	740.1	710.1	836.4	788.0	711.9	684.2	871.1	847.8
MSMEG _4329	MSMEG 4335	K.WAPSIVVGLGR.L	2	59.9	46.6	17.6	15.2	69.6	62.3	45.4	44.0
		R.AYDVHPLVHALLDEGVPF EEFQAK.W	3	175.7	159.2	134.4	127.8	198.4	204.0	180.4	214.6
		R.EALHEQLAVEHEK.I	2	2.6	1.1	0.7	0.4	3.2	1.7	1.7	0.3
		R.LSTFFDDGSVELLHER.D	2	102.4	107.3	260.5	242.4	207.1	167.4	189.9	233.5

MSMEG_4330	MSMEG 4336	R.GVNVAVHYNSASTEADADK.T	3	3.7	3.4	7.5	5.4	6.3	4.9	8.0	5.3
MSMEG_4337	MSMEG 4343	K.LLGGADAAGFGVTSTVSDLAR.F	2	87.8	85.4	59.8	58.2	69.6	76.0	78.9	82.8
		R.YLTEAVFEPLGMSTSK.L	2	233.5	251.2	90.5	91.2	222.5	231.4	100.7	107.2
MSMEG_4340	MSMEG 4346	K.LEM*PLIDFFSR.G	2	102.6	99.7	62.3	59.9	73.5	70.4	65.1	64.6
		K.LEMPLIDFFSR.G	2	296.2	276.4	332.3	302.3	210.5	166.5	349.6	271.3
		K.MTLTDGTELTPALGIGAFADK.T	2	321.1	332.9	308.2	300.4	187.6	173.6	282.2	271.3
		R.DFPTLISLYLQGR.L	2	301.6	267.4	297.2	280.8	227.9	188.7	348.1	294.5
		R.GRPHLC#FDTFNATQK.M	3	28.5	20.9	14.1	7.4	44.9	32.0	82.7	55.2
		R.GRPHLC*FDTFNATQK.M	3	25.5	18.0	14.1	7.4	44.6	30.8	81.3	54.9
		R.IGLDGIEDAFHK.M	2	191.0	195.6	169.7	169.7	137.0	136.2	153.0	137.1
		R.IGLDGIEDAFHK.M	3	279.1	233.4	197.9	152.7	208.4	189.2	247.1	197.2
MSMEG_4342	MSMEG 4347	R.SYSDFPTILDSIK.N	2	184.7	235.7	189.5	190.2	99.3	98.2	268.0	237.7
MSMEG_4358	MSMEG 4364	K.TALVTGASAGLGAAVAK.L	2	28.3	34.3	48.5	113.3	28.6	37.6	54.3	53.1
MSMEG_4362	MSMEG 4368	R.TVVSAAVDDGDAEGVDATHHVK.R	3	33.7	40.5	59.9	60.5	31.2	31.6	46.8	50.5
		R.TVVSAAVDDGDAEGVDATHHVK.R	4	26.4	25.8	39.3	38.7	28.1	26.8	45.4	42.0
MSMEG_4367	MSMEG 4372	K.FGYSPEQAYLLLGAAPIEGR.L	2	152.6	168.5	548.1	591.2	130.7	134.6	610.7	601.0
		R.HIPGVEFTGIVHPGLMGTAPSAGLLAK.W	3	258.2	208.3	543.7	511.9	79.5	96.6	244.0	275.2
		R.VPPLALPPEPQDAILGGLTGDAFTK.A	2	107.2	131.4	404.9	451.6	38.4	79.0	326.5	415.9
MSMEG_4381	MSMEG 4385	R.IKPGTVLTLWTEDAYGGR.I	3	18.2	19.8	58.4	67.4	6.3	7.0	31.9	53.3
MSMEG_4396	MSMEG 4399	K.ALGFPPNNVASIPPLAK.H	2	212.3	203.5	498.0	438.2	277.2	276.7	841.7	932.1
		K.ALQTAFVGTDLPGTLIR.Q	2	454.0	474.4	221.2	231.4	482.0	507.6	135.6	150.0
		K.NFTSFNWIGFSVFR.D	2	25.7	22.1	5.6	5.3	15.1	19.7	5.0	3.4

		R.DHGLLPIVIGDATGAAHPDHER.A	3	119.0	122.6	243.3	254.6	125.5	135.6	133.1	135.4
		R.ELVEPGDNELFEK.A	2	399.7	395.3	18.8	22.2	433.9	437.6	10.4	15.8
		R.PAAFLSVSQSNSLYR.P	2	76.2	87.2	121.5	123.5	34.4	37.9	84.4	85.6
		R.PAAFLSVSQSNSLYRPGGVQYAEGHAFR.G	4	270.0	308.4	13.4	20.1	356.7	376.9	23.9	37.8
MSMEG_4401	MSMEG4404	K.AAYAEHPPVPLPGVPDAIR.D	3	157.1	165.3	76.3	91.2	114.0	113.4	77.0	87.7
		R.ALLAPADDAALQVIVEDFR.A	2	118.6	129.7	102.0	109.7	82.8	89.5	78.0	88.3
		R.ALLAPADDAALQVIVEDFR.A	3	46.2	45.0	17.4	16.7	31.4	30.0	15.0	13.1
		R.VLEQTVVAHGGTPSEADIAK.W	2	25.2	23.8	14.1	13.0	20.6	18.3	14.2	12.1
		R.VLEQTVVAHGGTPSEADIAK.W	3	505.6	503.0	348.6	369.0	332.9	368.4	303.4	324.9
MSMEG_4454	MSMEG4449	R.RPELFEGIHFAQK.Q	3	24.5	28.7	0.1	1.0	1.3	3.3	0.9	0.2
MSMEG_4463	MSMEG4458	R.FNESPLDLLR.R	2	14.6	17.7	3.6	5.8	7.9	8.7	3.4	6.9
		R.YTQPTGSVEVNAK.R	2	7.1	4.8	9.5	7.9	2.6	2.1	5.0	4.0
MSMEG_4471	MSMEG4466	K.LLADVAEHPLPEEN	2	38.4	45.4	73.8	83.8	28.9	34.0	58.0	59.5
MSMEG_4474	MSMEG4469	K.AGVQWGLFGGAIENLGTER.H	2	58.9	65.0	51.4	71.7	55.2	58.5	58.6	84.3
		K.ELLTAYADDIK.G	2	107.6	97.2	66.5	60.8	92.6	68.8	100.5	65.8
		K.SYALQFAQNELVAK.C	2	260.1	253.2	260.7	261.0	212.3	194.8	297.0	264.2
		R.ADTDVFTTFEGDNHVLTQLVAK.E	3	170.6	170.8	181.1	181.1	151.7	125.3	234.5	189.9
		R.ENLSNEVFRPHYTPNTAIAR.A	3	70.5	74.4	44.6	51.4	73.0	73.7	57.6	74.0
		R.FFTMLGTLIR.G	2	31.2	34.0	43.6	50.4	22.3	19.5	53.6	46.8
		R.TAAQTIIQTILDTR.Q	2	270.7	216.1	323.8	264.5	270.5	167.0	466.5	309.8
		R.VTVGGSAAAAAR.V	2	7.6	5.3	5.2	3.3	4.5	3.5	4.5	3.8
		R.YADVSPEGVYSSPIENPGR.R	2	134.8	142.7	135.0	132.6	58.5	85.4	90.6	117.7

MSMEG _4475	MSMEG 4470	K.LPAPHGGSQWVQVR.D	3	11.8	8.5	15.9	13.0	6.0	8.4	12.5	16.3
MSMEG _4476	MSMEG 4471	K.AVLYQLQESVDTDK.L	2	19.2	0.0	15.7	0.0	15.6	17.0	8.6	5.6
MSMEG _4485	MSMEG 4480	K.FGFQGNPWGELEGVANR .T	2	321.2	307.9	266.2	276.6	283.4	226.3	302.9	278.1
		K.HSGVDLSFYDQAADTR.Y	2	87.7	89.4	46.3	52.2	45.5	44.6	29.2	31.1
		K.SAWDYGPLGVELK.E	2	189.9	214.0	125.3	147.3	153.8	140.4	86.0	113.3
		R.GLVYQSGEIYGGTK.S	2	95.4	115.6	78.5	107.1	55.3	56.8	59.3	65.1
		R.YVPYVIEPAAGLTR.S	2	0.0	0.0	0.0	0.0	0.0	0.0	0.0	0.0
MSMEG _4486	MSMEG 4481	K.TISNLTEHGEAHDHAGTP APALPSR.D	4	17.7	17.8	8.4	9.0	22.2	19.4	8.3	8.4
		R.DVLETAGELLR.A	2	8.1	8.7	2.7	2.9	8.9	10.0	8.1	8.4
MSMEG _4487	MSMEG 4482	R.AAIADLLNETEGFR.S	2	453.1	513.7	37.8	43.3	392.8	402.7	36.7	35.0
MSMEG _4489	MSMEG 4484	R.YNFPAAGLTDDALR.T	2	28.4	33.5	58.8	56.4	35.5	35.8	63.9	56.6
MSMEG _4490	MSMEG 4485	R.ADIPDVDLFIR.T	2	27.4	27.6	30.0	24.1	25.7	21.7	10.2	8.7
MSMEG _4527	MSMEG 4520	K.RPLIDGPAPEPVTRPIDHV GVQK.L	3	60.1	56.9	19.0	15.1	20.3	28.3	18.0	20.9
		K.RPLIDGPAPEPVTRPIDHV GVQK.L	4	138.8	146.1	12.4	19.0	45.7	64.0	2.6	8.6
		K.VLSSELGDYIER.V	2	140.0	162.2	204.4	201.5	74.1	67.1	102.4	102.0
		R.AGLDALGLPSTPSHWR.R	3	63.5	61.2	4.3	2.0	53.2	44.7	1.3	2.3
MSMEG _4528	MSMEG 4521	K.GYSAWVTGIR.R	2	34.6	32.2	2.5	5.0	16.0	20.1	3.1	1.9
		R.GAAELADASAEELLR.W	2	135.5	160.2	57.7	53.3	106.9	103.2	63.3	71.0
MSMEG _4533	MSMEG 4526	K.GIPFGSVVSLVVR.Q	2	271.2	254.4	191.0	173.2	218.7	192.9	144.8	105.9
		K.IENPVAVVNTTSHSEAANA LK.N	3	953.3	1101.5	805.0	869.8	726.1	845.1	690.5	891.4
		K.IWAEAGFRPVDPAVAAEF AADFPAPEK.L	3	163.8	200.0	50.4	58.8	91.2	114.5	34.1	45.2
		K.PADIVNFSVEPDVTR.L	2	210.7	205.9	153.9	163.1	199.5	176.2	81.1	97.0

		K.WNLLAPYAAK.S	2	48.5	50.6	42.0	28.1	43.3	43.4	84.6	62.3
		R.AVVDGKPADIVNFSVEPDVTR.L	3	462.4	478.9	311.2	339.8	549.1	376.0	366.7	332.7
		R.PVDPAVAAEFAADFPAPEK.L	2	33.6	38.9	16.5	27.3	37.6	45.8	2.1	5.8
		R.PVDPAVAAEFAADFPAPEK.L	3	26.0	22.6	19.5	27.5	9.6	12.5	2.2	12.4
MSMEG_4535	MSMEG 4528	K.ASDGPLTVTAPVPYAAGPTLR.N	2	17.0	21.3	39.9	47.6	25.3	38.5	53.8	107.4
		K.GLTYSPTGALLAAPTTSLPETPQGER.N	2	66.0	88.1	96.4	130.7	29.4	59.5	46.9	95.3
		K.GLTYSPTGALLAAPTTSLPETPQGER.N	3	240.4	290.2	379.0	424.5	151.7	200.8	293.7	348.0
		R.ATVLAIADELTEDGLVLR.Y	2	231.9	226.6	401.8	371.2	268.4	229.0	501.0	425.5
		R.DALVMGPWHDIDTR.S	2	85.5	83.5	137.1	142.5	58.8	75.4	107.8	140.9
		R.DALVMGPWHDIDTR.S	3	71.9	65.8	77.1	71.2	73.4	80.0	67.8	67.6
		R.DSTFALWGLYTLGLDR.E	2	28.2	35.2	48.6	61.0	25.3	38.3	46.8	75.8
		R.EADDFFSFIADVSGANNGER.H	2	91.0	91.2	156.2	153.8	75.1	84.7	141.7	161.0
		R.HLGNFPQAFTHLALINAVVHVIR.A	4	14.5	20.3	20.0	29.7	8.2	12.7	16.1	21.9
		R.LGPYGVSVPAAR.R	2	153.2	99.9	220.0	153.3	152.7	63.4	260.5	105.4
		R.LLSFASPLHLYAEEIEPR.T	2	43.7	40.9	61.2	61.8	43.7	33.5	70.4	50.1
		R.LLSFASPLHLYAEEIEPR.T	3	237.0	271.6	434.2	427.2	236.5	181.3	480.0	356.5
		R.LTEGDNVFVALSWSK.H	2	55.3	57.7	147.5	150.8	73.1	52.3	175.0	133.3
		R.SLVEEELHHLSGYDNSR.P	3	63.9	69.9	102.0	110.6	40.7	28.6	69.6	57.2
		R.SLVEEELHHLSGYDNSRPVR.I	4	60.9	53.1	63.6	80.9	70.3	90.1	91.4	123.7
		R.TPMDWDAEHILLR.T	2	127.2	130.7	202.8	183.3	105.4	90.9	253.9	174.4
		R.TPMDWDAEHILLR.T	3	58.2	54.8	182.1	173.8	56.0	42.7	212.9	155.6
		R.VSATWEYSGPAYGEAIAR.A	2	214.1	209.7	396.4	382.8	159.6	199.9	313.5	365.5

		R.YGDDALDASLLLAVLTR.F	2	79.7	72.7	134.0	121.6	82.9	91.1	161.1	162.1
MSMEG _4553	MSMEG 4547	R.SEPDSMVVAATAGEATK.H	2	18.6	21.8	10.2	12.8	10.9	15.8	5.1	4.8
		R.TDTTPALSPVAANLEAVAEK.L	2	78.3	83.4	88.1	89.3	85.4	84.7	80.8	75.3
MSMEG _4569	MSMEG 4566	R.SELEFLQPGALLESLETR.L	2	12.7	13.6	49.3	51.7	8.1	6.5	33.2	35.2
MSMEG _4570	MSMEG 4567	R.VALPENSLVVNSSQGGGSK.D	2	100.0	102.4	163.6	181.3	99.2	153.1	95.1	224.3
MSMEG _4571	MSMEG 4568	K.SALALALNKL	2	138.4	131.5	43.9	45.1	279.5	262.8	97.2	96.9
MSMEG _4617	MSMEG 4609	K.VGPYALQDFSLFQVLR.F	2	103.5	136.7	146.4	148.3	91.7	111.1	146.6	157.4
		K.VYDVTFENVQAGLR.T	2	200.4	160.9	113.9	121.1	239.4	120.4	143.3	130.7
		R.FPFVPSNYER.L	2	56.6	50.2	59.2	57.6	55.0	44.6	77.2	50.4
		R.IEFQLDPPTGDIGLLR.E	2	60.1	56.2	1508.5	1547.3	46.6	36.2	1044.4	1311.4
MSMEG _4624	MSMEG 4616	K.TVNIVPVARPEA	2	131.6	100.3	48.8	39.1	138.3	171.6	49.3	65.8
		R.GGDDTLFALAPGAVEFGAK.R	2	750.5	802.9	331.1	345.9	669.3	718.3	337.2	323.2
		R.GGDDTLFALAPGAVEFGAK.R	3	49.3	38.7	11.6	6.4	46.9	52.7	11.4	16.8
		R.GTHFHPGVNVGR.G	3	1.0	0.5	0.1	0.1	1.1	0.8	0.2	0.1
MSMEG _4625	MSMEG 4617	K.LDSEPGASVSLPVALVVDGANVTSK.A	2	331.9	394.8	243.4	273.9	426.2	413.5	225.0	224.0
		K.LDSEPGASVSLPVALVVDGANVTSK.A	3	537.5	399.8	424.4	324.5	624.8	779.6	374.0	466.3
		K.VAVTAEVLEHTK.G	2	61.9	58.2	45.9	44.2	80.9	67.2	46.8	41.3
		K.VAVTAEVLEHTK.G	3	163.0	147.6	93.4	82.2	221.4	193.3	97.0	78.6
MSMEG _4626	MSMEG 4618	K.AGAAVALYEEPDVLVK.V	2	77.9	76.8	72.2	66.4	57.8	50.9	70.4	73.8
		K.VWLPSGGTLVIDR.T	2	28.2	26.7	15.0	13.4	22.3	22.7	19.3	19.4
		K.YEPAGPDAPDVFAVHR.I	2	4.9	6.4	35.7	35.7	7.7	6.3	34.6	36.3
		R.EVVPSDAGVIIR.T	2	63.3	61.7	19.1	21.8	51.5	49.0	21.1	19.4

		R.KIEQALKPGDYVVVQVSK.D	3	18.1	25.2	21.1	23.4	17.6	21.6	14.2	15.7
		R.LGTGLVEAFSTAC#THC#GGR.G	3	25.8	27.8	15.6	18.8	32.9	27.2	19.0	16.3
		R.RRPPILSEAEFLAR.R	3	34.0	31.6	6.5	9.4	33.2	34.9	3.6	9.1
		R.VLDALAEFDGR.Q	2	16.6	17.7	6.9	14.4	12.1	13.0	3.1	6.6
		R.YLVYVPGASSTGISR.K	2	77.9	76.3	69.7	74.7	57.7	55.0	86.9	82.9
MSMEG_4627	MSMEG4619	K.GLTLAALELK.N	2	174.6	169.5	92.8	87.9	164.0	157.8	112.1	102.9
		R.GDFALITQDNLVHGSDSPESAAR.E	3	918.3	931.0	526.7	523.3	769.6	812.4	620.2	568.4
		R.KGLTLAALELK.N	2	25.1	39.7	9.6	15.5	21.2	22.9	13.6	12.6
MSMEG_4630	MSMEG4622	K.LLHPVMPFVTETLWK.T	3	13.9	19.5	7.7	12.9	11.3	13.4	6.7	10.0
		K.VTPAHDPNDFEIGMR.H	3	62.7	38.7	28.3	14.5	46.4	19.7	28.6	6.8
		K.YEDVEGELVSFR.Y	2	166.6	161.7	144.1	166.8	156.4	132.9	170.0	165.7
		R.AEVDSAFDNYEFNR.A	2	724.9	711.6	127.2	145.8	676.7	663.1	138.2	173.6
		R.LFDAGLIYQAER.L	2	239.2	226.8	277.1	248.2	218.1	189.1	340.0	273.0
		R.LSLQWWVK.V	2	30.6	28.8	16.4	15.4	37.5	28.4	27.9	22.3
		R.LVNWSPVLETAISDLEVK.Y	2	207.2	241.0	266.1	301.7	165.9	207.0	283.4	366.5
		R.LVNWSPVLETAISDLEVK.Y	3	99.6	119.8	90.1	106.0	81.7	87.2	103.1	114.3
		R.TTAVLAAVLDTLLK.L	2	102.4	102.4	89.9	90.9	119.6	85.0	152.7	102.5
		R.TTAVLAAVLDTLLK.L	3	6.9	6.4	3.1	2.0	6.7	6.5	3.6	3.5
		R.YGSMNDDEPHIVVATTR.V	2	36.2	34.5	24.6	29.1	16.8	37.4	10.3	47.3
		R.YGSMNDDEPHIVVATTR.V	3	155.2	159.1	72.3	79.1	66.5	78.6	63.1	67.3
MSMEG_4636	MSMEG4627	K.AIIEHKPVEPGPAAEGEPTPY	3	0.3	0.0	309.1	0.0	0.0	0.1	297.9	321.0
MSMEG_4645	MSMEG4636	K.TAEVSADDIVVHDAQIDDPAYAFALSR.L	3	251.3	252.4	90.3	93.7	357.4	342.4	65.8	78.1
		R.APTIATGLALAR.P	2	9.7	8.2	60.6	53.4	12.7	13.7	63.4	65.4
		R.LINLTHGEPITFGADGEYAVVK.S	3	395.3	373.6	188.9	171.6	518.0	496.4	156.9	150.2

		R.WC#PGC#GDYVILNTIR.N	2	81.4	84.8	16.3	15.2	99.5	103.6	15.5	11.4
MSMEG _4646	MSMEG 4637	K.TEQADLLQALFGR.N	2	174.9	160.7	78.2	68.3	367.4	271.0	117.5	81.6
		K.TEQADLLQALFGR.N	3	1.6	3.8	0.0	0.0	14.9	9.0	0.1	0.5
		K.VVVPEMNLGQLALLLR.G	2	242.4	268.3	119.8	124.0	520.5	531.9	165.3	170.3
		K.VVVPEMNLGQLALLLR.G	3	23.0	27.4	5.8	6.6	47.6	51.5	8.9	10.3
		R.ELEASEAFIR.E	2	25.4	20.9	8.7	6.1	19.5	33.7	1.3	15.9
		R.FLVDVQSVTK.V	2	187.2	172.7	85.5	83.6	340.2	253.4	103.3	76.0
		R.FTSEAALFGNDLATQPNYPAEIR.A	3	133.6	175.6	57.5	80.9	220.1	231.0	47.6	65.0
		R.GGLIIANSDEFTK.R	2	173.8	185.5	167.5	175.2	185.3	201.7	113.9	143.6
		R.HLNPFPANLGEVLAR.Y	2	136.3	149.0	68.2	93.9	201.1	229.7	72.2	74.5
		R.HLNPFPANLGEVLAR.Y	3	101.9	91.2	21.7	19.5	183.4	169.1	28.1	20.6
		R.IGGLESANGSGNISYEPK.N	2	239.1	240.4	111.5	107.4	296.5	353.3	91.5	109.6
		R.KPEIAEANVLALK.A	2	180.6	154.0	129.1	110.3	311.8	239.8	134.9	115.3
		R.KPEIAEANVLALK.A	3	126.3	102.7	46.0	28.1	222.6	151.6	47.4	32.9
		R.LAAATVEGEANEESAVGANA	2	40.9	43.7	5.2	8.8	47.5	67.6	12.4	9.8
		R.SPSDC*FDIAVEAAR.I	2	155.8	155.2	29.6	29.6	240.2	197.4	34.3	26.4
MSMEG _4647	MSMEG 4638	R.TDFPAETLAALVER.G	2	18.5	17.2	13.1	15.6	22.2	17.2	24.5	13.9
MSMEG _4664	MSMEG 4654	R.LPVLLLPADTFATR.V	2	13.2	10.9	61.0	67.5	27.0	18.9	90.5	51.4
MSMEG _4668	MSMEG 4658	K.APDAFLDALDAHFGITSPR.K	3	8.0	18.8	0.8	1.6	15.4	19.9	0.9	0.8
MSMEG _4672	MSMEG 4662	K.EYGIIDTVLQYR.K	2	796.1	793.6	691.0	640.6	707.9	688.9	671.7	602.3
		R.ADIQTVC#LGQAASAAAVLLAAGTPGK.R	3	179.9	163.0	94.4	86.8	94.8	113.4	54.7	61.5
		R.ADIQTVC#LGQAASAAAVLLAAGTPGKR.L	3	79.0	83.2	41.0	46.4	98.5	91.3	52.9	50.1
		R.ADIQTVC*LGQAASAAAVLLAAGTPGK.R	3	179.9	163.0	94.4	86.8	93.0	113.1	54.4	61.5

		R.VLIHQPALSGVIQGQFSDLEIQAAEIER.M	3	760.7	799.6	368.6	378.6	512.1	617.2	296.8	316.7
		R.YILPSFIEHSSFGVK.E	2	281.0	257.9	313.0	301.1	308.5	292.3	345.9	326.0
MSMEG_4673	MSMEG4663	R.GTGQGLNLVDSVYER.L	2	1947.6	2143.2	1891.5	1992.4	1846.0	1819.3	1966.4	1892.7
		R.IIFLGSQVDDDIANR.L	2	1690.9	1762.6	1701.7	1680.5	1467.7	1446.0	1725.0	1695.8
		R.IIFLGSQVDDDIANR.L	3	160.2	130.7	130.6	98.0	147.3	133.5	101.6	97.3
		R.ILM*HQPLGGVTGSAADIAIQAEQFAVIK.K	3	496.1	474.5	684.9	645.4	389.7	374.0	669.6	635.6
		R.ILM*HQPLGGVTGSAADIAIQAEQFAVIK.K	4	18.8	14.0	10.4	7.4	10.4	10.5	3.7	4.7
		R.ILMHQPLGGVTGSAADIAIQAEQFAVIK.K	3	706.5	687.5	825.9	784.5	507.2	492.6	750.7	721.1
		R.LC#AQILLLSAEDPTK.D	2	212.8	207.3	204.2	208.6	113.9	110.0	106.0	106.8
		R.LC*AQILLLSAEDPTK.D	2	208.3	206.7	204.2	208.6	112.3	106.6	106.0	106.8
		R.LNAEFTGQPIER.I	2	164.6	146.2	127.0	115.2	149.5	169.6	122.5	134.1
MSMEG_4674	MSMEG4664	K.AIEALLEQTEVPLPEK.I	2	604.4	575.3	543.2	546.3	585.2	468.6	549.2	502.5
		K.FAEQLAEQGSSR.E	2	7.3	4.7	8.8	4.0	1.6	2.3	1.3	4.5
		K.ITVDPIEVTDEEVDAELQSLR.A	2	247.1	232.4	214.8	210.3	128.9	144.9	182.5	203.0
		K.ITVDPIEVTDEEVDAELQSLR.A	3	196.0	255.0	181.3	242.6	103.0	116.5	147.7	172.0
		K.IVQAQIDEVVHNAIHGLDHDEEK.F	4	154.0	150.2	99.4	105.9	42.2	45.7	48.8	46.9
		K.LEIQVSQNDLTER.L	2	85.2	84.1	196.3	185.1	34.3	32.4	120.0	120.3
		K.TQLLMDAVADKLEIQVSQNDLTER.L	3	236.7	238.6	214.7	198.8	157.2	133.5	177.1	165.3
		R.ELPEPDDEFAQLASEYDTIEELR.N	3	95.6	157.3	89.0	137.6	23.3	57.0	29.9	75.5
		R.INVEVPFTELEPDFDR.A	2	849.1	851.5	703.7	682.4	809.8	761.0	709.5	653.8
		R.YSEAVSTSDLKPLGQPEIEITK.L	3	1629.6	1791.6	899.5	918.6	1028.6	1139.1	635.8	616.3
MSMEG_4679	MSMEG4669	K.DVDLPWTQDITFSGK.V	2	4.1	6.4	2.4	5.5	7.9	10.6	5.6	2.5
MSMEG_4687	MSMEG4676	K.GLSEGGIPIGAALFSADGELLGSGHNR.R	3	66.5	70.7	107.7	114.0	53.9	60.6	74.7	72.8

		R.RVQDGDPSIHAETDAFR.A	3	4.2	3.3	3.7	1.2	6.4	5.2	5.4	5.6
		R.TFSGGHEWLADNGVSVTLLDDER.C	3	79.0	81.7	189.1	188.0	91.2	95.9	184.5	175.4
		R.VQDGDPSIHAETDAFR.A	3	12.8	0.0	24.6	0.0	13.7	16.5	18.2	24.0
MSMEG_4688	MSMEG4677	R.DLPEEYQEMMK.K	2	59.5	69.9	29.4	44.3	48.3	37.2	40.2	33.5
MSMEG_4690	MSMEG4679	R.AAEAGSDHQLAYVNALTTSVLSAR.H	3	590.2	577.2	403.4	377.8	591.2	523.1	545.6	455.3
		R.ADFDVDSSGAFTR.F	2	303.3	249.9	198.6	158.4	279.3	225.3	259.0	196.9
		R.DHAFANATFGDLLGALEK.A	2	164.7	177.8	126.2	134.4	172.1	177.5	163.7	155.7
		R.DHAFANATFGDLLGALEK.A	3	146.2	162.1	97.8	110.8	170.5	125.1	147.9	103.5
		R.DLSGWGQQWLK.T	2	118.4	120.3	61.4	68.6	124.0	103.5	84.4	90.1
		R.FVDPVDDEVYLYSQFETADAK.R	3	210.9	233.7	146.4	167.3	180.5	190.4	166.1	189.8
		R.GQLILVNDDDLTYC*SLR.L	2	183.4	156.4	95.0	103.0	187.4	166.9	108.2	90.9
		R.HTTLLADLLDKDPGSLGLEGLVVDTDLR.W	3	31.9	39.8	26.8	41.6	31.8	41.9	30.5	49.3
		R.HTTLLADLLDKDPGSLGLEGLVVDTDLR.W	4	89.4	106.6	59.3	66.7	78.6	102.8	64.0	93.4
		R.IADIADPLPR.T	2	67.2	47.8	98.5	79.5	85.0	30.0	138.9	136.8
		R.LAVGIYDDVDGK.L	2	173.5	127.1	89.8	73.2	163.2	112.2	108.9	77.7
		R.LDPDSLSTVVTR.I	2	515.4	481.9	566.0	548.4	471.0	429.6	551.1	540.1
		R.MFAC#FDQPDLK.A	2	48.9	48.7	17.9	26.2	56.7	55.8	51.6	52.5
		R.SGEIDADGPETPFIDAEAQR.D	2	282.4	271.1	142.2	146.0	204.6	245.3	147.4	174.8
		R.SGEIDADGPETPFIDAEAQR.D	3	61.5	72.5	26.7	30.7	49.3	51.9	20.3	32.1
		R.TLAWSAAWEMTR.D	2	116.8	113.1	58.2	63.9	99.6	82.8	86.5	61.5
MSMEG_4692	MSMEG4681	K.EGNLIDGLISAVR.V	2	71.7	71.3	27.2	29.5	70.3	67.5	26.4	24.6
		R.FAVYIGPLGADTAATAR.E	2	275.0	275.2	102.3	102.9	312.0	314.6	106.2	100.2

		R.GIESAAPLGVSAAAASFK.E	2	273.9	282.6	207.2	212.5	275.1	286.3	182.7	181.0
		R.ISAVTEPGELSVHYPFPTMDLVVLDDALK.Y	3	41.6	52.2	21.2	26.7	31.1	44.8	15.3	23.2
MSMEG_4693	MSMEG4682	K.MSDPWTHAPILWAAEEPR.E	3	35.0	35.6	6.3	7.5	30.5	33.6	7.1	5.7
MSMEG_4698	MSMEG4687	R.SVNAAPDSKPSVIGETQLAAVHIEQQR.L	4	169.2	162.7	191.6	250.6	138.0	140.2	158.1	175.7
MSMEG_4699	MSMEG4688	K.APVDLLWNGGIGTYIK.A	2	10.7	12.1	16.3	14.9	14.3	15.4	21.4	23.6
		K.ATGAVVTPPEVVR.R	2	0.4	0.7	18.0	12.8	0.3	0.5	15.7	11.0
		K.GTATFSDIANEVAK.S	2	52.0	49.2	41.6	40.0	53.3	44.9	51.8	45.7
		K.ISPHPSIPHAPDAEAQR.D	3	8.1	5.5	5.0	3.5	5.1	5.8	4.6	5.8
		K.ISPHPSIPHAPDAEAQR.D	4	13.3	9.7	9.9	7.7	6.5	9.5	7.1	9.9
		K.LALKDDVLASDLPDQEVFASR.L	3	60.4	58.8	69.8	64.0	56.4	66.7	102.5	85.8
		K.VIGEGGNLGVTALGR.I	2	68.2	72.8	125.8	152.4	70.7	75.2	124.5	140.5
		R.AFANLIEATLR.T	2	22.2	19.4	17.4	15.9	26.8	22.8	31.9	26.0
		R.FADAVTAIWHGR.V	3	10.1	9.7	5.1	3.9	7.9	9.1	5.2	7.2
		R.GPQADAPGVTSTGPLAVAAHDDLVSDDLVAAHYR.L	4	55.3	53.1	88.7	85.3	67.7	68.4	118.5	130.4
		R.INTDALDNSAGVDC#SDHEVNIK.I	3	17.2	19.9	17.4	20.6	19.3	20.6	31.4	33.7
		R.ITEDVGVGPVDAVR.S	2	40.8	40.7	46.4	49.1	51.7	44.5	71.7	61.2
		R.LEMQDILVR.E	2	15.4	15.0	7.6	6.9	13.9	13.4	12.2	10.3
		R.LFISGLLDVTDNVDK.A	2	14.0	11.6	23.5	25.2	18.5	18.3	36.3	49.5
		R.LPEGTAADSVDTSLENESR.I	2	29.6	32.3	20.1	24.6	33.3	30.2	37.5	32.9
		R.LPEGTAADSVDTSLENESR.I	3	9.8	14.6	9.3	14.1	7.7	12.0	11.0	16.6
		R.LPYYFPTR.L	2	11.3	11.0	9.4	12.7	11.7	12.7	13.2	19.6
		R.LVPGILAEAR.Q	2	18.4	16.6	8.3	6.8	20.0	20.3	13.8	14.9
		R.TALGLDADVEELTPPALIK.A	2	20.3	19.1	21.5	24.3	26.6	23.5	44.1	37.3

		R.YGAYPYIVVVR.E	2	16.5	18.0	8.1	8.8	20.0	24.5	23.8	22.1
MSMEG _4700	MSMEG 4689	K.DLSFTLPR.N	2	102.5	76.1	106.9	65.9	72.9	51.1	124.5	90.0
		K.TVWQVVSDGLDYIEVGQ NEIPSR.A	3	76.1	105.9	72.7	101.8	19.5	39.6	36.2	70.0
		K.VILDDVTLNFLPGAK.I	2	316.5	300.6	458.5	447.5	248.3	157.1	567.6	380.2
		R.AYPYEGNYSTYLEK.K	2	289.7	293.3	384.4	368.9	164.1	185.2	335.0	349.7
		R.KLDFEEIQIPTPPR.L	2	78.4	95.7	245.6	341.4	41.2	49.4	255.6	301.7
		R.KLDFEEIQIPTPPR.L	3	152.6	133.7	228.2	190.3	100.2	82.0	239.8	200.4
		R.LDRYEEMVAEAEK.T	2	55.2	44.8	49.0	49.3	23.8	29.0	41.8	49.9
		R.LDRYEEMVAEAEK.T	3	38.7	30.8	40.7	40.4	13.4	17.2	36.5	37.9
		R.LGSVVVEVEHLDK.G	3	159.9	141.6	183.7	153.8	108.6	93.9	205.3	161.2
		R.LNLALTLK.E	2	51.4	37.2	39.0	22.4	42.3	20.9	59.6	30.3
		R.NGIVGVIGPNGVGK.T	2	12.1	12.9	79.2	85.3	7.5	5.8	91.4	56.5
MSMEG _4709	MSMEG 4698	K.LGIHPGGGATWMLQR.A	3	18.0	24.1	26.8	24.3	12.5	17.1	43.1	33.6
		R.HGLALTVADDPLAAAR.E	2	55.1	61.1	149.0	134.3	44.8	74.2	185.1	232.8
		R.HGLALTVADDPLAAAR.E	3	20.2	17.2	24.2	24.3	13.7	12.4	27.1	23.7
		R.SAVEAAEADPDVHAVVVT GAGK.A	2	12.7	12.7	19.3	25.1	5.9	4.3	11.4	23.1
		R.SAVEAAEADPDVHAVVVT GAGK.A	3	183.6	181.6	244.2	254.5	136.9	140.6	253.8	239.2
MSMEG _4714	MSMEG 4704	K.SRPGEGILTFAHTGR.N	3	15.8	17.2	22.2	20.1	14.1	19.4	7.1	16.2
MSMEG _4716	MSMEG 4706	R.LQVEHPVTELVTGIDLVEQ QIR.I	3	11.0	14.7	26.7	35.6	17.7	22.5	31.0	35.6
MSMEG _4722	MSMEG 4713	K.AFANTFSESLR.G	2	45.9	27.4	36.3	22.4	34.3	35.6	45.5	48.5
		R.AVVTGASQNIGEALATEL AAR.G	2	62.4	72.2	71.5	72.4	48.8	52.5	67.3	76.8
		R.AVVTGASQNIGEALATEL AAR.G	3	124.1	132.1	127.0	143.3	109.0	106.1	150.6	126.7
		R.LIPDFLWIDTEYTAK.L	2	84.2	96.3	131.0	148.1	57.8	68.0	114.5	136.6
		R.TELPDPSEQSLVER.L	2	226.4	235.8	268.7	279.2	205.6	208.4	302.3	322.1
		R.YGVTVEVR.A	2	49.9	26.1	11.4	8.5	99.5	79.9	13.3	9.8

MSMEG _4724	MSMEG 4715	R.STVDLATAEEMVLDYIR.G	2	24.1	27.8	33.3	33.4	18.6	20.0	28.7	31.7
MSMEG _4727	MSMEG 4717	R.GPAIDLGDLAYTLAR.R	2	4.5	4.2	122.7	114.0	7.4	6.1	154.9	164.3
		R.LIAEQVGLLLR.R	2	0.7	1.0	0.0	0.0	3.0	2.8	0.1	0.1
MSMEG _4742	MSMEG 4734	K.AALDEILHSWR.S	2	21.7	23.7	22.0	29.7	38.2	31.7	21.5	26.4
		K.LWVEQGALPAR.M	2	95.6	78.8	194.1	145.1	127.4	134.2	146.0	154.6
		K.TLADSIGGHAFELDVEDPAAITR.V	3	155.2	159.5	262.1	264.6	212.9	190.7	281.5	263.8
		R.LADNGVVLIASSHDVGRPR.A	4	72.0	70.3	98.0	99.9	111.8	107.5	111.6	101.1
		R.LLVVGASSGIGHAVAESAAK.R	2	60.7	57.1	187.8	183.7	78.2	78.7	154.4	146.5
		R.LLVVGASSGIGHAVAESAAK.R	3	225.2	213.5	373.3	332.0	254.7	260.0	378.9	384.3
		R.VGVGPTEDTEILR.G	2	113.8	85.9	205.2	166.4	95.0	121.4	133.3	172.1
MSMEG _4752	MSMEG 4742	R.LALTVDSLAER.A	2	220.0	228.2	229.0	249.5	209.0	205.9	229.1	217.2
MSMEG _4753	MSMEG 4743	K.EGLTFPLLSDPDR.S	2	137.9	126.9	173.9	173.3	106.1	106.4	178.2	169.9
		K.IAVAQYNVR.A	2	3.5	0.0	2.8	0.0	2.0	2.5	2.8	3.3
		K.VIVYFYPAASTPGC#TK.Q	2	380.9	440.4	573.1	641.2	239.7	247.6	492.7	461.8
		K.VIVYFYPAASTPGC*TK.Q	2	379.9	437.9	561.1	636.8	239.3	246.5	476.5	454.7
		R.DKEGLTFPLLSDPDR.S	2	107.6	106.4	132.3	129.2	82.4	69.9	134.5	107.5
		R.DKEGLTFPLLSDPDR.S	3	147.9	128.7	148.1	131.6	102.2	105.4	136.5	143.0
		R.DNLAELNDAGLDVVGISPDKPEK.L	2	64.6	61.6	71.7	67.7	26.2	33.9	70.2	54.0
		R.DNLAELNDAGLDVVGISPDKPEK.L	3	1193.7	1281.6	1195.4	1323.8	895.6	989.8	1197.6	1185.1
		R.KVIVYFYPAASTPGC#TK.Q	2	35.7	37.2	49.5	58.7	32.5	29.4	58.0	49.6
		R.KVIVYFYPAASTPGC*TK.Q	2	35.7	37.2	49.5	58.7	32.5	29.4	58.0	49.6

		R.LQAGDTAPAFSLPDADGN TVSLSDYR.G	2	73.8	93.5	97.2	120.1	39.6	46.1	74.1	79.5
		R.LQAGDTAPAFSLPDADGN TVSLSDYR.G	3	443.9	509.2	490.6	545.5	290.0	342.8	354.0	456.4
		R.SVLTAWGAYGEK.T	2	250.8	241.1	336.0	320.5	186.8	184.4	330.0	313.1
MSMEG _4755	MSMEG 4745	K.AATAEALLLDALGR	2	59.4	49.3	107.4	90.3	44.3	42.9	147.5	110.2
		K.PAVTVIGIDTTPIGK.S	2	121.6	124.4	124.9	139.0	63.1	62.5	96.9	133.9
MSMEG _4757	MSMEG 4747	K.AGWYDTETGELVPEC#EI VER.Y	2	43.3	42.4	46.3	49.3	60.5	65.7	49.6	57.0
		K.AGWYDTETGELVPEC*EI VER.Y	2	43.3	42.4	46.3	49.3	60.5	65.7	49.5	56.9
		K.AREEM*SGAADESDDEAP AGTIR.A	3	3.7	1.6	2.5	1.0	3.3	2.9	1.1	1.4
		K.AREEMSGAADESDDEAP AGTIR.A	3	90.6	63.2	88.5	63.1	65.9	75.7	55.3	60.4
		K.DAQTPTLLFPFAAPR.V	2	193.3	173.0	172.9	145.1	367.3	302.6	286.6	215.0
		K.DAQTPTLLFPFAAPR.V	3	13.4	12.0	7.0	6.4	21.9	25.7	7.6	12.8
		K.DFVAEISER.T	2	11.6	7.7	4.2	1.8	2.6	4.1	0.1	0.4
		K.DVELLALAQLIGAAGTLVA R.R	3	5.8	6.2	3.5	3.9	14.3	10.3	6.8	5.3
		K.ELTESILVR.Q	2	24.0	21.1	18.4	14.3	30.4	27.6	13.5	14.0
		K.GGAAVFQM*MGLC#QILR. D	2	5.0	6.4	1.2	2.3	12.1	10.4	1.6	1.9
		K.GGAAVFQMMGLC#QILR. D	2	25.4	23.0	14.3	12.7	41.6	33.4	28.1	20.3
		K.GGAAVFQMMGLC*QILR. D	2	23.4	21.3	14.2	12.7	40.6	32.4	28.1	20.3
		K.GSIAASVVGQLLDGGATVI ATTSR.L	2	35.7	38.0	45.9	44.2	21.4	37.4	16.9	31.7
		K.GSIAASVVGQLLDGGATVI ATTSR.L	3	97.4	100.1	108.5	116.0	111.1	103.9	115.5	92.5
		K.GSIAASVVGQLLDGGATVI ATTSR.L	4	4.4	5.2	2.3	2.0	6.0	4.9	2.2	1.3
		K.HDTSTLANDPNETELHER. I	3	0.7	0.7	0.3	0.3	1.3	1.1	0.3	0.4
		K.HVTVEFALGTR.E	2	14.8	13.6	152.2	149.0	23.1	20.5	119.9	118.0

K.IVAAAANAGHWAELAGGG QVTEQIFNDR.I	3	287.9	271.0	294.9	281.7	364.6	416.6	340.2	343.4
K.IVAAAANAGHWAELAGGG QVTEQIFNDR.I	4	59.1	55.3	60.5	63.5	81.8	86.2	58.9	67.9
K.LPEYSHSTVEVLNSER.D	2	65.6	51.3	58.3	46.3	116.8	78.2	94.1	53.5
K.LPEYSHSTVEVLNSER.D	3	298.7	312.5	299.3	312.7	338.6	368.3	282.6	292.4
K.LVEWVGTEQTESLGPQSI HLK.D	3	473.0	495.2	609.0	601.7	403.1	448.7	450.3	479.1
K.LWLAEEGDIDAQWEQLS QR.F	3	10.5	9.9	3.6	3.4	20.0	26.3	7.7	12.3
K.M*GLPVLAVVGYAQSFAD GVHTSIPAPGLGALGAAR.G	4	25.3	33.5	20.1	27.2	7.2	19.2	7.9	15.0
K.PVNFVPVIDKDVR.R	3	12.9	10.3	6.9	6.3	7.3	9.8	7.2	8.4
K.PYFGDVAEM*TYLQWLR. R	2	7.5	7.6	4.1	3.4	13.5	11.9	4.2	4.1
K.PYFGDVAEMTYLQWLR.R	2	28.8	39.1	4.1	3.9	35.3	38.6	12.9	9.5
K.QLTSAAVSVPGVLLTQIAA VR.A	2	6.8	5.4	3.6	2.9	20.3	14.6	12.1	7.1
K.QLTSAAVSVPGVLLTQIAA VR.A	3	23.8	25.6	14.9	13.9	46.9	42.3	30.7	23.3
K.RPDSPWLDITWR.D	2	33.6	30.2	21.4	18.3	54.8	52.2	32.5	27.1
K.RPDSPWLDITWR.D	3	40.2	36.0	23.3	19.9	49.4	57.0	23.2	25.9
K.SAPTVAGLATNTLK.L	2	431.8	412.4	670.7	656.7	459.0	461.3	639.9	587.7
K.TLGKPVNFVPVIDK.D	2	22.4	20.3	16.0	16.2	26.6	19.2	16.3	9.0
K.TLGKPVNFVPVIDKDVR.R	3	88.5	78.7	57.6	50.6	166.7	138.9	99.5	80.3
K.TVYAFPGQGIQHK.G	2	24.7	8.4	4.6	4.5	16.9	10.4	5.5	5.0
K.TWELGPGWAK.H	2	117.0	110.4	93.2	91.5	127.1	142.2	84.3	95.0
K.VDLTGGLGDIK.I	2	197.8	186.4	181.2	168.1	227.9	221.3	180.4	166.3
K.VIDGAVAAVAAR.R	2	5.6	4.9	3.5	3.9	4.1	3.4	2.5	4.4
K.VLLWAVQR.L	2	182.4	162.0	148.7	128.8	218.1	224.6	160.3	153.5
K.VTGPDGVLASAAR.L	2	9.2	10.3	7.6	8.6	13.2	13.2	10.7	11.3

K.VVDNTATVTVDWDPER.V	2	130.6	122.2	105.2	109.4	173.6	180.9	125.3	131.2
R.AAALLAGLEGPIVHGMWLSAAAQHVVTATDGKPVPPAK.L	4	56.6	64.2	44.6	42.5	77.2	74.0	52.5	48.5
R.AAALLAGLEGPIVHGMWLSAAAQHVVTATDGKPVPPAK.L	5	58.4	58.6	46.0	47.7	73.5	77.7	46.7	48.5
R.AFVQFDPEHTVAR.P	2	63.1	46.6	43.5	34.6	73.7	42.3	37.2	22.4
R.AFVQFDPEHTVAR.P	3	108.7	97.7	69.3	70.1	79.1	84.0	39.5	41.9
R.AFVQFDPEHTVARPLPDSSDWEVTR.K	3	52.4	43.3	38.7	33.5	135.3	97.8	93.5	58.5
R.AFVQFDPEHTVARPLPDSSDWEVTR.K	4	139.6	153.8	453.3	457.4	225.6	283.6	354.9	392.9
R.AGGHHSWEDLDDLLLATYSELR.S	4	4.7	4.9	1.9	1.8	7.7	8.9	2.7	3.7
R.AIQFNTLFLDPYLWK.L	2	55.5	66.4	59.1	61.6	83.4	109.4	62.1	80.4
R.APGNPLFIVSQK.T	2	231.1	212.3	228.2	200.3	296.3	271.2	241.8	208.8
R.AVLAIAAGVEGPENLPK.V	2	309.9	298.4	293.1	285.5	409.9	387.4	330.3	297.6
R.AVLAIAAGVEGPENLPK.V	3	58.7	55.3	61.2	58.6	67.3	73.4	54.5	60.3
R.DASGPLAVVLDAPDVLWAGR.M	2	87.6	64.4	82.9	62.2	95.3	76.9	69.2	58.6
R.DASGPLAVVLDAPDVLWAGR.M	3	20.8	22.7	11.0	14.0	17.7	26.8	7.4	15.2
R.DEIIAAMAQTAK.P	2	58.1	52.9	43.4	37.1	62.0	55.8	38.5	35.3
R.DLVPAEPLDEVLADYDTWR.N	2	102.5	112.3	97.1	105.0	82.5	129.1	59.2	98.0
R.DLVPAEPLDEVLADYDTWR.N	3	27.6	36.8	23.1	34.8	18.4	43.0	8.9	30.4
R.DVTVGAPVDMRPFAVVSGDHNPIHTDR.A	3	24.7	25.3	2.0	1.0	45.7	34.6	12.9	6.4
R.EEMSGAADESDDEAPAGTIR.A	2	4.5	2.6	2.7	1.3	1.1	1.8	0.3	0.4
R.EFVDDGAIDPDHASPLLVSVFLDK.D	3	53.1	53.7	43.4	45.1	35.6	41.1	30.2	30.2

R.ETLGFSVLHVVR.D	2	48.8	38.9	122.6	104.0	130.2	96.4	152.8	102.2
R.FEGTGHVVATQANWWQGK.A	2	22.9	19.7	13.0	11.3	42.3	38.9	23.2	19.9
R.FEGTGHVVATQANWWQGK.A	3	208.6	204.2	219.9	202.6	190.2	258.0	180.8	193.4
R.FELYC#TQIAEK.E	2	53.4	49.0	48.7	44.4	43.2	39.3	32.1	31.5
R.FELYC#TQIAEKEEAER.K	3	69.5	67.2	79.3	65.6	75.3	86.4	72.4	70.5
R.FELYC#TQIAEKEEAERK.N	4	12.5	13.4	5.6	11.8	35.2	40.5	22.0	25.7
R.FEQAAVDEVLGAGAEPVEVLSR.R	2	55.1	51.4	48.3	48.7	48.0	51.3	33.0	32.4
R.FEQAAVDEVLGAGAEPVEVLSR.R	3	150.3	166.7	131.0	136.7	112.0	145.7	75.4	99.7
R.FLGM*VKPGDQVDFR.V	2	9.9	9.2	23.9	24.4	26.3	13.4	32.6	27.3
R.FLGMVKPGDQVDFR.V	2	67.5	60.8	56.8	33.9	95.1	71.8	71.4	45.5
R.FTLTDVVR.S	2	5.1	6.4	2.4	1.9	6.8	9.3	0.3	0.9
R.GGAVFAPVFDPVQVEVGFHTPR.L	3	225.3	200.5	215.5	173.0	284.7	256.6	225.7	184.7
R.GGDLGGLHAGALASAADVDK.V	2	78.7	76.3	24.0	22.5	83.0	66.7	32.1	20.2
R.GGDLGGLHAGALASAADVDK.V	3	129.2	126.7	111.8	112.3	101.8	107.9	83.3	77.1
R.GGDLGGLHAGALASAADVDKVIDGAVAAVAAR.R	3	30.8	31.8	30.5	38.7	58.2	58.7	36.3	39.4
R.GGDLGGLHAGALASAADVDKVIDGAVAAVAAR.R	4	72.9	73.4	71.7	69.7	147.6	160.3	118.7	121.3
R.GGDLGGLHAGALASAADVDKVIDGAVAAVAAR.R	5	15.9	10.5	1.1	0.7	7.2	16.1	2.0	2.9
R.GISVSLPSAGGASGGVVDSAALGEFAEK.V	3	130.1	144.5	109.1	126.5	107.3	158.7	70.3	109.5
R.GITVLGDRPPM#VSVTNADPER.I	3	99.4	102.0	80.0	77.6	105.2	109.7	69.0	70.1
R.GITVLGDRPPM*VSVTNADPER.I	3	99.0	101.1	73.6	74.6	104.8	106.2	68.2	69.3
R.GITVLGDRPPMVSVTNADPER.I	3	243.4	232.7	197.9	197.2	260.5	257.7	180.4	180.5

R.GLGIGIVPAATR.G	2	217.3	242.8	216.7	230.3	377.3	331.7	336.8	262.9
R.GSQYAIAGTVAGLEALEE EIER.R	2	67.5	61.7	62.2	56.0	74.4	81.5	55.2	62.1
R.GSQYAIAGTVAGLEALEE EIER.R	3	76.9	95.8	59.1	69.5	78.0	122.1	52.9	80.4
R.IAAEVPTKPVIVHIEGGR.A	2	13.4	12.0	3.1	3.3	51.5	23.6	21.6	8.3
R.IAAEVPTKPVIVHIEGGR.A	3	189.9	174.4	112.9	113.4	314.9	257.7	182.0	141.0
R.IAAEVPTKPVIVHIEGGR.A	4	262.8	252.4	259.2	235.6	363.5	369.0	286.3	279.8
R.IAAPTEWQVR.E	2	175.2	161.4	132.1	115.4	182.0	195.3	116.6	121.0
R.IAELETLLEPGR.A	2	287.4	247.3	277.5	235.3	331.1	375.9	291.5	293.1
R.IDQIEALDSIESITDGASSR .R	2	45.8	40.7	6.5	5.4	90.7	77.0	3.0	5.6
R.IGSELVM*AATAR.L	2	4.7	5.1	1.0	1.2	4.7	4.2	0.4	1.0
R.IGSELVMAATAR.L	2	102.3	91.7	101.3	94.2	110.3	100.8	102.8	95.8
R.IYELLEEFSSDVR.T	2	192.0	170.5	166.4	151.0	312.2	248.1	251.9	176.8
R.IYELLEEFSSDVR.T	3	13.1	13.2	5.4	6.3	18.2	21.6	3.6	8.2
R.LAAIRPSQIDLDDADVK.D	2	14.2	11.6	6.3	8.4	17.9	12.4	8.3	4.5
R.LAAIRPSQIDLDDADVK.D	3	85.7	91.0	80.1	83.0	62.4	63.9	50.6	53.9
R.LAAIRPSQIDLDDADVKDF VAEISER.T	3	28.9	34.0	127.2	118.6	72.3	65.4	97.2	105.9
R.LAAIRPSQIDLDDADVKDF VAEISER.T	4	87.0	104.0	82.4	93.1	184.4	157.3	128.3	117.9
R.LEVADDQHVVLSVPLSGT WIEIR.F	3	7.9	9.9	8.1	8.6	23.6	16.5	11.2	10.1
R.LGFLEAQGGGTILLAR.G	2	125.0	108.3	92.0	74.9	189.1	141.8	118.1	86.0
R.LHPQDFGPIETLFDADAD GER.L	3	293.3	270.5	297.1	273.0	348.7	354.5	299.3	296.0
R.LHVVLPGSPNR.G	2	3.4	2.9	4.6	4.8	6.8	5.0	4.7	6.0
R.LLAELPKEPAEFTVTAK.A	2	32.9	25.0	27.1	19.3	110.1	75.6	83.2	49.7
R.LLAELPKEPAEFTVTAK.A	3	190.1	166.0	177.9	157.1	284.7	332.6	218.3	247.4

R.LLDEVAGDADAVAER.R	2	405.4	412.7	395.7	398.1	547.9	522.9	431.6	394.5
R.LLDEVAGDADAVAER.R	3	54.5	55.2	42.6	41.9	56.8	70.1	37.8	42.3
R.LLEDPEAAITALLQR.Y	2	167.2	173.4	142.9	137.0	268.9	241.3	203.4	165.0
R.LLEDPEAAITALLQR.Y	3	122.9	129.4	91.5	97.9	178.5	173.5	114.8	103.1
R.LSDGIEIVGR.W	2	6.7	6.7	4.2	4.2	12.9	11.8	6.1	4.5
R.NAADGALLATLEER.F	2	273.5	252.9	282.5	259.9	384.8	358.1	330.3	301.7
R.NAADGALLATLEER.F	3	23.8	26.6	15.2	15.5	26.7	37.4	12.0	17.4
R.NLFTVGAVPEVAR.P	2	240.7	241.7	322.2	274.6	341.1	286.9	346.0	263.2
R.NLFTVGAVPEVARPWSSYAPTVVK.L	3	6.1	6.6	8.0	12.8	31.1	40.6	26.3	36.6
R.PFAVVSGDHNPIHTDR.A	3	7.7	6.4	8.6	14.6	5.7	5.0	7.7	14.4
R.PFAVVSGDHNPIHTDR.A	4	20.1	18.5	9.3	8.7	11.5	14.2	3.7	4.8
R.PFTLDRDFIQEIR.D	3	51.7	46.3	34.9	31.8	89.9	92.7	49.6	53.7
R.PIGFEPLQWVR.A	2	16.3	15.7	14.3	17.7	16.9	14.3	9.4	9.2
R.PLPDSSDWEVTR.K	2	167.5	184.1	156.4	174.1	120.5	125.2	90.2	85.8
R.PSQIDLDDADVKDFVAEISER.T	3	16.2	22.3	14.2	18.6	18.0	18.6	8.5	11.2
R.PWSSYAPTVVK.L	2	172.3	167.2	179.2	166.3	160.4	168.2	137.3	139.1
R.QVDWVDEITELHEAGAR.W	2	21.7	16.5	19.7	18.5	28.9	22.8	16.2	13.8
R.QVDWVDEITELHEAGAR.W	3	21.7	16.7	16.0	11.3	44.1	41.0	28.4	25.3
R.RDASGPLAVVLDAPDVLWAGR.M	3	34.8	33.2	38.4	33.7	56.9	47.5	46.4	37.6
R.RDEIIAAMAQTAK.P	2	33.4	25.3	16.2	14.5	52.8	40.0	14.5	13.0
R.RDEIIAAMAQTAK.P	3	34.7	24.8	18.6	9.8	39.6	37.7	10.5	12.1
R.RGISVSLPSAGGASGGVVDSAALGEFAEK.V	3	139.1	130.9	114.9	104.6	233.9	218.4	159.9	151.8
R.RYVELAIGDGNSTADTK.R	2	4.3	4.1	4.6	3.7	9.1	6.7	8.1	6.7
R.RYVELAIGDGNSTADTK.R	3	29.5	26.6	21.7	19.4	30.3	39.1	17.1	23.4
R.SFILVPGIDVPFHSSVLR.V	2	28.9	27.7	17.1	16.4	70.4	46.8	39.9	23.3
R.SFILVPGIDVPFHSSVLR.V	3	112.3	129.2	85.9	104.3	165.9	156.1	124.2	101.4

R.SGGAPIVEVDDAATAM#R.A	2	46.7	41.1	93.0	81.4	52.6	45.0	54.9	53.8
R.SGGAPIVEVDDAATAM*R.A	2	46.7	41.1	80.7	72.8	52.6	45.0	50.8	49.8
R.SGGAPIVEVDDAATAMR.A	2	309.5	245.8	246.1	201.3	292.0	259.6	220.0	186.4
R.SGGAPIVEVDDAATAMR.A	3	20.4	23.7	5.4	10.0	18.1	27.3	1.9	9.8
R.SLAQLGVGADDIAVISK.H	2	373.0	361.0	487.8	487.9	503.1	444.4	484.5	495.6
R.SLDC#VDDELATSGHFVWVR.E	3	42.1	36.1	58.2	54.5	75.7	68.3	57.4	58.0
R.SNITIC#VGGGIGTPER.S	2	39.5	35.6	11.9	14.0	55.2	58.5	21.3	21.8
R.SPILLAGM#TPTTVDAK.I	2	95.3	96.3	94.6	97.8	115.4	102.4	88.7	76.9
R.SPILLAGM*TPTTVDAK.I	2	267.5	274.7	521.4	521.7	299.7	226.3	569.8	466.5
R.SPILLAGMTPTTVDAK.I	2	477.8	484.1	830.4	836.5	546.8	479.9	891.6	779.7
R.SQLGADIHEIDNAASR.C	2	99.2	93.8	63.2	51.2	135.8	115.2	79.5	61.4
R.SQLGADIHEIDNAASR.C	3	176.0	191.5	129.8	136.6	219.5	235.1	140.7	130.8
R.SVVITGTPEQLSR.F	2	174.3	159.7	148.9	139.8	129.0	163.1	96.7	113.2
R.TADGNAADTAHALVDR.L	2	1.4	1.1	1.1	0.7	3.6	2.3	1.4	0.4
R.TADGNAADTAHALVDR.L	3	9.5	9.0	5.0	4.8	10.7	11.5	3.7	4.7
R.TGEFLEIVNFNLR.G	2	37.2	38.7	1163.2	1260.7	99.7	68.3	349.7	274.7
R.TVLPPVLSIR.N	2	226.0	197.7	203.7	176.7	324.6	274.5	247.9	202.9
R.TYKPFGPVLSDAINDQLR.T	2	14.3	15.1	28.1	25.1	38.3	28.9	33.7	22.7
R.TYKPFGPVLSDAINDQLR.T	3	306.2	281.9	241.6	235.3	363.4	422.2	271.7	274.2
R.VADHTGVTATFGAPLAPTLTVVPDALVGR.C	3	444.3	353.5	370.6	286.5	424.7	359.5	324.9	239.4
R.VDEPVGELLDR.F	2	134.4	124.1	209.4	212.8	105.9	107.8	150.0	128.9
R.VGIDVGAEVLEVSAR.I	2	152.4	161.5	225.2	227.4	220.4	213.3	272.3	255.5
R.VGIDVGAEVLEVSAR.I	3	5.7	8.2	1.2	1.4	6.9	12.9	0.7	2.1
R.VSLAHALIGWTK.G	2	48.8	39.8	45.3	36.0	89.6	70.5	65.2	46.0
R.VSLAHALIGWTK.G	3	72.5	63.2	44.2	40.7	106.7	115.0	61.5	64.5
R.WAETVGLDVELAK.E	2	14.7	13.1	27.8	78.9	50.5	44.9	88.5	133.0

		R.WIETQDLLFIEEAAGGLGVER.F	3	2.2	2.2	1.1	1.3	15.1	15.4	8.2	11.3
		R.WILDLGPGDILTR.L	2	113.3	119.2	99.4	109.2	268.8	231.7	197.6	178.4
		R.YEADQVC#VIPGTAAVAGITR.V	2	73.5	72.1	68.7	61.7	105.5	102.7	92.7	73.2
		R.YEADQVC#VIPGTAAVAGITR.V	3	53.9	50.0	63.2	61.0	51.7	71.0	47.9	64.4
		R.YEADQVC*VIPGTAAVAGITR.V	2	73.5	72.1	68.7	61.7	105.5	102.7	92.7	73.2
		R.YPDAETVVLHPADVPFFVELC#K.T	3	137.7	148.1	109.1	123.3	193.1	209.5	139.0	146.2
		R.YPDAETVVLHPADVPFFVELC*K.T	3	137.7	148.1	109.1	123.3	193.1	209.5	139.0	146.2
		R.YVELAIGDGNSTADTK.R	2	86.9	68.2	81.8	56.5	72.9	78.1	58.7	56.3
MSMEG_4847	MSMEG4835	K.HELLLALFEDALSR.S	3	11.5	11.3	10.3	8.1	5.6	4.8	4.8	4.6
		K.RPLFTDFAPR.L	3	10.1	3.3	5.3	0.2	8.5	7.1	4.2	1.7
		K.VAIQLLFESSRPDPAAK.R	3	37.9	37.7	41.9	36.5	24.1	20.5	17.0	23.8
MSMEG_4888	MSMEG4874	R.HGIVLSLQFETDDDR.N	3	0.1	0.1	1.7	1.4	1.2	2.0	0.7	1.1
MSMEG_4891	MSMEG4876	K.DFTFVC#PTEIAAFGK.L	2	867.3	840.2	54.4	41.2	358.7	367.0	19.9	20.6
		K.VLGVSVDNEFVHFQWR.A	3	412.2	411.4	3.1	2.2	161.1	172.7	1.1	1.3
		R.ATFIVDPNNEVQFVSVTAGSVGR.N	2	387.9	369.0	4.1	4.7	266.8	245.4	4.2	2.8
		R.ATFIVDPNNEVQFVSVTAGSVGR.N	3	763.4	729.1	24.4	24.6	536.2	511.2	28.9	29.0
		R.ELTAAC#GVLNADGVADR.A	2	1039.7	1268.7	23.4	32.6	617.3	598.1	9.8	21.7
		R.ELTAAC#GVLNADGVADR.A	3	59.8	64.9	2.5	2.2	18.2	28.0	1.9	3.3
		R.VLDALQSDELC#AC#NWK.K	2	1487.5	1614.8	71.2	73.0	847.6	849.0	66.3	78.1
		R.VLDALQSDELC#AC#NWK.K	3	28.6	23.6	0.7	0.0	15.2	16.7	0.4	0.5

MSMEG _4901	MSMEG 4886	K.GSGQGWLTAEYAMLPAA THDR.S	3	38.7	50.3	62.5	74.4	28.7	30.9	36.7	40.7
		R.EALDAPYPGVLPEGPAPK. K	2	134.6	151.1	179.7	214.7	203.2	207.5	204.2	253.0
MSMEG _4905	MSMEG 4890	R.FSVGSFDAVR.R	2	25.8	25.6	6.8	11.1	17.6	23.7	2.5	2.8
		R.IIFSPAEGGSNTAVATAK.E	2	120.6	117.1	68.7	66.9	92.0	102.0	30.3	38.3
MSMEG _4906	MSMEG 4891	K.EVITDLENNYSGISER.I	2	57.2	70.2	25.7	25.3	55.5	59.0	23.6	29.7
		R.FVNIYVNDEDVR.F	2	54.0	62.4	31.7	32.3	51.5	59.9	26.6	33.8
MSMEG _4911	MSMEG 4896	R.GFLDADTLDYLAGYR.F	2	30.3	26.1	61.3	42.3	35.2	35.7	87.0	66.9
MSMEG _4914	MSMEG 4899	R.VTETGEVAVYPVAPQAR. P	2	24.2	26.5	15.0	10.5	26.0	31.2	13.7	14.6
MSMEG _4915	MSMEG 4900	K.RLDELAADHSFLDR.L	3	174.4	153.7	114.0	105.2	119.5	117.1	86.9	112.5
		K.SALNGGLNLSIR.D	2	97.4	84.7	205.8	173.6	88.6	75.7	163.0	154.2
		K.SASDLGLPLIAVGLYYR.S	2	41.2	42.0	98.2	101.6	76.0	68.1	147.3	145.4
		R.AFTEIEGLPAPEVFHMNE GHAGFLGAER.I	4	119.0	130.3	180.0	169.7	129.0	135.1	183.6	200.4
		R.DDLEAAALYDLLENAVAP K.F	2	152.0	171.3	117.4	124.2	165.6	208.4	128.5	166.6
		R.ELIGGELAESALESEVWE R.L	2	78.3	78.6	135.5	136.4	99.0	95.4	173.6	163.3
		R.IKQEILAGIGGVR.A	2	38.8	36.2	57.8	47.2	66.2	50.8	122.6	80.6
		R.IKQEILAGIGGVR.A	3	53.5	38.2	43.6	33.7	82.9	62.2	89.2	69.7
		R.IPLLLLDSDIPENEHDLR.G	3	244.1	252.0	364.1	394.0	342.1	268.7	582.9	406.5
		R.LDELAADHSFLDR.L	2	22.0	20.9	87.7	72.8	24.6	22.4	75.3	67.7
		R.LDELAADLDNYLTR.P	2	36.3	46.7	57.1	69.5	25.9	28.0	29.6	33.0
		R.LLLDPDRPVQLIVAGK.S	2	60.5	51.4	185.0	148.6	103.1	70.6	233.9	147.6
		R.LLTAADGDPVLIELALPEG R.D	2	83.4	98.4	134.7	161.2	128.5	131.5	196.9	205.9
		R.LQQVEPGHLWWIR.S	2	41.0	29.0	109.4	106.7	45.0	31.9	145.8	127.4
		R.LQQVEPGHLWWIR.S	3	77.0	75.3	98.6	100.3	92.7	93.5	140.6	135.1

		R.NDGDPVALLGSVSPK.R	2	92.8	107.5	209.0	222.7	139.1	141.8	288.5	288.5
		R.PLWYQQQLDTGVAMPK.G	2	44.4	41.5	101.2	106.5	23.0	30.6	43.7	88.6
		R.RDDLEAAALYDLLENAVAPK.F	3	41.0	44.3	79.2	88.5	54.9	56.6	192.8	115.2
		R.SSTVFTTHTPVPAGIDR.F	3	96.3	101.3	173.0	153.0	72.4	90.3	155.2	152.4
MSMEG_4916	MSMEG4901	K.FHHLDNDALLAYSK.F	3	25.1	28.1	2.9	3.4	39.3	43.1	4.9	5.0
		K.NEDPDILFLSEAFTR.P	2	17.1	17.9	11.7	9.9	16.7	13.8	8.5	8.4
MSMEG_4918	MSMEG4902	R.LEIDYPGVGDAVVTHTTADAYR.F	3	164.2	157.3	235.9	246.6	129.2	146.2	234.1	256.8
		R.LLAGEHHDPHSVLGAHEYPGHTVIR.A	4	48.6	52.8	35.5	41.0	32.3	55.2	27.0	52.2
		R.SSNQLTDAHLRPDPSDIHR.L	4	31.6	21.1	42.9	29.0	19.1	11.5	40.0	25.2
MSMEG_4917	MSMEG4903	K.LSGSGEEAEQVDPALAQAR.A	2	29.8	26.1	12.8	12.0	13.5	18.0	4.2	6.6
		K.LSGSGEEAEQVDPALAQAR.A	3	31.1	20.3	36.6	21.1	23.4	26.7	30.7	34.8
MSMEG_4920	MSMEG4905	K.DFSGTDLGAIAIR.A	2	702.2	658.4	750.1	700.5	1129.9	1047.3	806.3	751.5
		K.DGVFADEVVPVSIPQR.K	2	1991.6	1942.6	2052.7	1953.9	2667.4	2781.0	2269.7	2299.2
		K.DGVFADEVVPVSIPQR.K	3	45.1	41.1	23.1	28.6	51.9	60.2	18.8	17.2
		K.GDPIEFAEDEGIR.A	2	550.6	525.9	835.5	991.2	832.2	847.9	624.8	654.0
		K.M#C*LSGIDAIALADQLIR.A	2	174.3	164.2	139.7	148.8	126.0	124.6	83.3	86.0
		K.M*C#LSGIDAIALADQLIR.A	2	205.7	205.7	171.7	181.4	163.7	173.7	120.5	127.3
		K.MC#LSGIDAIALADQLIR.A	2	800.4	840.8	473.1	449.3	921.3	983.3	562.3	517.8
		K.MC#LSGIDAIALADQLIR.A	3	41.5	54.1	34.2	42.4	89.4	82.4	48.4	49.2
		K.MC*LSGIDAIALADQLIR.A	2	800.4	840.8	470.6	448.2	921.3	983.3	559.4	516.5

		K.MC*LSGIDAIALADQLIR.A	3	41.5	53.9	34.2	42.4	89.4	82.4	48.4	49.2
		K.VNVNGGAIAIGHPIGMSGAR.I	2	256.4	301.5	289.9	328.7	243.3	225.8	241.3	291.1
		K.VNVNGGAIAIGHPIGMSGAR.I	3	802.9	852.2	872.0	826.9	771.4	589.8	604.3	500.4
		K.YGDATLVDHLAYDGLHDVFTDQPMGALTEQR.N	4	516.4	541.6	514.8	563.3	446.1	408.6	313.7	284.6
		R.AGEFDVIVAGGQESM*SQAPHLLPK.S	3	400.9	418.7	478.4	515.3	528.9	539.9	477.1	435.1
		R.AGEFDVIVAGGQESMSQAPHLLPK.S	3	1817.2	1688.5	1677.6	1620.3	1624.3	1506.8	1096.2	990.9
		R.AGEFDVIVAGGQESMSQAPHLLPK.S	4	67.7	66.6	41.4	35.5	37.5	44.1	4.7	12.2
		R.ANTTAESLAGLKPAFR.K	2	605.6	388.0	696.9	531.5	1029.4	960.9	772.6	774.9
		R.ANTTAESLAGLKPAFR.K	3	1183.0	757.6	853.7	512.0	2144.8	2123.3	1172.1	1160.2
		R.GSGYAVAALC#GAGGQGDALVLR.R	2	314.0	320.3	279.2	274.5	359.5	328.5	236.7	198.1
		R.GSGYAVAALC*GAGGQGDALVLR.R	2	314.0	320.3	266.6	268.3	359.5	328.5	230.2	185.0
		R.IALHAALELAR.R	2	273.9	286.5	162.8	186.2	314.7	281.8	106.8	115.2
		R.KGDPIEFAEDEGIR.A	2	489.9	387.2	526.4	462.0	659.9	719.0	554.7	589.0
		R.KGDPIEFAEDEGIR.A	3	248.1	145.3	102.4	40.0	342.1	326.0	156.7	157.8
		R.QAAVAAGIPWDVAALSINK.M	2	195.2	183.1	189.9	197.9	366.8	317.7	231.7	235.3
MSMEG_4921	MSMEG 4906	K.DAGGVLIELVEPSPEEH	2	127.7	154.0	14.8	24.8	176.5	183.9	13.3	18.0
		K.GSAQIQLLAPIDEK.S	2	194.3	214.4	433.0	484.9	261.7	266.3	403.7	444.5
		R.GPGLQQLAYR.T	2	2.5	2.1	6.1	6.2	3.4	5.6	6.6	7.7
		R.PALASALVTAIDHVGIAVPDLDAAAK.W	3	273.0	319.2	82.4	92.0	297.8	338.9	58.7	65.0
		R.PALASALVTAIDHVGIAVPDLDAAAK.W	4	79.7	51.7	14.6	9.9	93.3	93.5	13.3	12.8
MSMEG_4932	MSMEG 4917	R.AGAGLVLAGLVADGETEVHDVFHIDR.G	4	56.9	50.4	11.6	11.8	46.3	61.0	9.2	10.4
		R.GIPQLSSAPVWSSDIR.A	2	205.3	216.5	273.6	245.7	184.4	196.8	199.1	208.7

		R.GYPLFVENLVSLGAEIER.V	2	67.2	66.0	13.4	10.7	78.9	78.4	5.2	9.9
		R.LSGEVAVGGAK.N	2	4.9	3.6	0.1	0.0	5.0	3.7	0.0	0.0
MSMEG_4934	MSMEG 4918	R.LSDLLFILSR.V	2	63.6	62.1	33.7	40.6	49.1	46.4	22.3	23.2
		R.SAWIAVNEFGDSVSVLPAK.Y	2	127.2	124.9	61.1	65.1	81.9	94.7	39.6	48.7
		R.VANPEGDVLWKPGANQ	2	230.7	236.5	153.0	152.1	225.7	232.6	160.9	170.1
MSMEG_4935	MSMEG 4920	R.ELWSGPATFVFTR.T	2	62.7	63.3	108.7	105.8	239.6	216.5	146.9	123.8
		R.HIPLVAQLVDDAMVR.V	3	7.9	6.5	10.7	11.0	25.6	25.2	9.0	9.5
		R.IAVDGGFLSVTEETVR.I	2	125.8	125.8	103.8	104.5	258.4	248.3	102.4	90.2
		R.ILVENAQFESEIDADAAKEDAASDDER.T	3	64.8	73.1	82.5	104.6	270.0	259.6	121.2	121.0
MSMEG_4936	MSMEG 4921	K.DTALVFGQMDEPPGTR.M	2	246.8	252.3	323.0	300.2	339.2	393.0	136.1	149.3
		K.GEFDHLPEQAFFLIGGLDDLAK.K	2	13.6	20.1	30.9	44.1	55.5	78.7	29.4	40.4
		K.GEFDHLPEQAFFLIGGLDDLAK.K	3	254.0	286.7	529.7	609.3	968.0	1054.0	657.0	727.6
		K.GHVFNALGDC#LDDPGYGK.D	3	35.2	33.3	74.9	65.0	100.1	124.1	58.8	64.7
		K.GIFPAVDPLASSSTILDPAIVGDEHYR.V	3	482.2	517.8	947.7	997.1	1618.7	1798.3	1078.4	1183.7
		K.IALFGGAGVGK.T	2	12.6	12.0	22.3	21.4	114.9	91.3	32.6	17.8
		K.TLTLEVAQHLGDSLVR.C	2	174.3	171.0	730.3	804.7	676.0	622.8	830.1	866.2
		K.TLTLEVAQHLGDSLVR.C	3	166.4	144.9	266.9	237.8	654.7	594.2	300.9	296.2
		K.TVLIQEMINR.I	2	52.2	61.8	104.7	90.9	245.3	249.0	124.2	189.2
		K.VVDLLTPYVR.G	2	120.2	97.8	244.8	193.4	716.4	624.4	384.0	372.6
		R.DEQGQDVLLFIDNIFR.F	2	217.1	233.8	326.7	344.7	442.4	656.4	283.9	418.6
		R.DEQGQDVLLFIDNIFR.F	3	12.1	12.0	8.9	14.1	32.6	44.6	11.7	19.8
		R.EGNDLWVELADANVLK.D	2	196.7	183.8	327.7	294.6	593.0	515.2	336.0	291.2
		R.FTQAGSEVSTLLGR.M	2	537.1	510.8	2106.2	2252.6	1979.5	1976.7	2010.4	2107.6
		R.GVEVTDTGASISVPVGDGVK.G	2	419.4	492.2	917.0	1025.3	1703.3	1603.3	942.3	1001.3
		R.ITGPVVDVEFPR.G	2	394.6	407.1	763.5	773.0	1306.6	1277.7	1110.8	1102.2

		R.NFGGTSVFAGVGER.T	2	188.3	164.1	315.2	239.8	811.6	657.0	502.7	390.2
		R.VALSALTMAEFFR.D	2	123.9	113.1	301.4	350.6	430.6	371.8	285.3	217.5
		R.YKDLQDIIAILGIDELSEEDKQLVNR.A	3	23.6	33.6	81.9	89.5	128.5	180.4	75.7	117.4
MSMEG_4937	MSMEG4922	R.AAPMEVEYVGEVETGPR.T	2	7.8	13.0	16.1	17.7	44.1	47.2	3.4	4.6
		R.AEELFSLLR.D	2	2.9	3.7	15.1	14.4	21.5	23.4	19.7	20.6
		R.PYAAEITNMLTELAGASALDHPLLVER.K	3	28.5	36.3	34.4	38.1	29.0	40.8	7.2	12.7
		R.RAEELFSLLR.D	2	26.1	34.5	46.5	54.9	46.4	45.0	63.3	58.4
		R.TLYSFEPDPETLFDALLPR.Y	2	17.9	25.1	30.8	36.7	48.4	66.4	10.2	17.0
		R.VYAALLEAAASESASR.R	2	28.0	26.6	55.0	63.8	108.7	104.1	71.9	100.9
MSMEG_4940	MSMEG4926	K.SAEQVAAAQADYEKEM*AEAR.A	3	2.0	3.8	3.8	8.2	15.6	19.5	20.2	17.8
		R.AQASGEVAQTLTQADQQLSAQGDQVR.S	3	90.2	111.0	78.2	93.2	218.5	224.3	63.3	65.9
MSMEG_4950	MSMEG4935	R.LQALAEEQAEADASADR.A	2	16.6	18.1	2.5	2.4	7.4	14.7	1.5	0.5
MSMEG_4951	MSMEG4936	K.TGIHPEYVDTTVQC#GC#GHSFTTR.S	3	174.5	198.0	98.3	118.3	214.3	201.0	117.3	115.3
		K.TGIHPEYVDTTVQC#GC#GHSFTTR.S	4	344.3	386.3	163.8	192.4	335.0	377.8	146.9	177.8
		K.TGIHPEYVDTTVQC*GC*GHSFTTR.S	3	174.5	198.0	98.3	118.3	214.3	201.0	117.3	115.3
MSMEG_4955	MSMEG4941	R.AAAMPASAEYLAILR.R	2	46.7	49.8	45.8	42.5	56.2	56.4	63.6	66.4
MSMEG_4956	MSMEG4942	K.AAVHQPWPGLIEAYR.D	3	154.9	132.7	48.6	58.3	101.3	112.1	27.7	53.7
		R.IGSPASWNSAVEAQQQSDGR.F	2	134.4	76.8	73.0	45.0	85.7	102.1	58.2	78.8
		R.IGSPASWNSAVEAQQQSDGR.F	3	120.7	91.1	46.3	30.2	93.9	114.0	27.9	52.5
		R.LPIGDDWTTVTLLEGGTPLIHAK.R	3	176.5	198.9	171.8	175.6	98.5	81.0	109.4	109.9

MSMEG _4957	MSMEG 4943	K.LPIAPIGFIPTR.Y	2	99.9	84.7	81.1	70.9	109.6	94.9	116.3	86.4
		R.EGITTVSAEDFASAR.A	2	199.2	184.3	279.5	258.3	200.9	195.7	348.4	321.3
MSMEG _4958	MSMEG 4944	K.FGTPLFVIDEDDFR.T	2	133.6	121.8	436.9	440.2	144.2	119.5	413.9	425.5
		R.PAVVAVADGQAR.L	2	10.7	9.7	6.5	5.3	8.3	6.6	3.8	2.2
MSMEG _4959	MSMEG 4945	K.GEPTPEDGYAGDYIVDIA QQVIAK.A	3	87.6	76.7	151.0	128.9	113.2	105.8	221.7	168.4
		R.AGTVITLDDLVEAIGVDAA R.Y	2	112.9	112.2	141.7	143.5	96.7	101.4	101.2	134.6
		R.AGTVITLDDLVEAIGVDAA R.Y	3	48.2	51.6	33.0	37.5	56.3	52.3	48.0	37.8
MSMEG _4971	MSMEG 4957	R.AALQNPEFTETVVTR.A	2	33.2	41.9	97.7	96.8	11.1	13.3	149.6	153.0
MSMEG _4974	MSMEG 4960	K.AQGIPAQFLVDILSDLR.T	2	5.4	4.9	5.3	3.9	6.1	7.5	8.4	9.5
		R.PADAISIADVLR.C	2	9.8	11.3	12.1	13.0	10.4	12.6	13.0	11.7
MSMEG _4976	MSMEG 4962	K.NFPNSFVQTDLQQR.L	2	270.3	300.0	232.6	299.5	327.2	351.0	212.1	242.1
		R.EEIGAIVESVAPR.G	2	172.5	188.5	327.6	347.4	192.6	211.5	267.3	295.6
		R.GAFNLGFAPTVVAAATAT R.S	2	599.1	70.5	415.7	181.9	583.6	625.1	361.6	376.3
		R.GVM*ELEGVQAALDATAE LLDR.A	3	37.3	33.1	52.8	51.4	47.0	48.7	42.0	41.3
		R.GVMELEGVQAALDATAEL LDR.A	2	7.3	0.0	7.4	0.0	8.1	10.9	4.2	6.9
		R.GVMELEGVQAALDATAEL LDR.A	3	44.2	39.3	60.5	57.8	56.6	59.7	49.6	49.0
		R.LADSALVLIDC#QNTYTR. G	2	34.6	39.3	21.5	22.8	44.6	46.6	17.3	16.5
		R.LADSALVLIDC*QNTYTR. G	2	34.6	39.3	21.3	22.5	44.6	46.6	17.3	16.5
		R.TAGIPIIHIQHDSGPGSPY DVR.E	3	99.5	115.2	69.2	86.1	132.7	127.0	72.2	59.2
		R.TAGIPIIHIQHDSGPGSPY DVR.E	4	154.0	163.7	96.9	103.2	184.4	196.8	83.9	88.4

MSMEG _4978	MSMEG 4965	R.AGEITHFTGIDSPYQRPK. H	4	9.1	10.2	4.8	3.9	3.9	9.5	1.2	3.9
MSMEG _4985	MSMEG 4972	K.HVAVVAC#MDAR.L	2	2.2	1.8	1.1	1.0	1.2	0.7	0.9	0.3
		K.TFSGPLPLPPSK.H	2	128.9	146.3	74.2	101.4	63.9	73.5	44.2	52.1
		R.AIQGETGIKPEWAAESFT DLEEDVR.Q	3	1191.6	1279.8	948.0	989.3	946.2	975.6	890.5	837.0
		R.GFIFDVATGK.L	2	230.5	214.0	178.2	156.0	191.1	195.3	176.9	163.0
		R.ILGLGDGEAHVIR.N	2	166.0	189.2	133.7	131.3	144.9	134.7	139.8	109.8
		R.ILGLGDGEAHVIR.N	3	503.1	0.0	308.6	0.0	470.1	450.1	305.4	265.2
MSMEG _4997	MSMEG 4983	K.HLAHDVAVMYK.G	3	1.4	0.2	2.3	0.8	0.1	0.0	0.6	0.6
MSMEG _5006	MSMEG 4992	K.FPTSAIAVLR.F	2	3.9	0.0	3.0	0.0	4.4	5.2	2.8	2.6
MSMEG _5005	MSMEG 4993	R.FWYEQGSLDNER.Q	2	41.8	48.8	32.0	33.4	29.4	35.7	27.7	31.1
		R.WQAANPAGLVDITR.F	2	112.2	120.5	77.0	80.4	85.7	95.3	72.2	66.1
MSMEG _5022	MSMEG 5007	K.ADLTFASLPYR.I	2	3.4	3.5	8.7	10.5	9.2	9.3	14.7	10.2
		R.AWTLLTTLQELK.G	2	1.9	1.5	27.7	28.1	18.3	12.4	53.1	33.9
		R.VDAWLADFESALAAR.D	2	5.7	6.3	27.5	31.8	16.3	13.6	44.5	31.7
MSMEG _5037	MSMEG 5024	K.GSLLLEDVGVPLPALADLV SGVEK.I	3	118.4	150.3	46.1	61.0	87.1	113.0	46.7	63.8
MSMEG _5042	MSMEG 5030	K.LIEAELPSVEDVNAQR.V	2	102.7	108.5	99.6	111.0	78.7	88.9	105.8	149.5
		K.TAAFAIPILSK.I	2	29.3	27.8	18.5	18.8	25.6	18.8	24.4	22.8
		K.VGPGHIVGAIANEGGLHR. N	4	62.1	43.6	25.4	22.3	47.9	50.9	38.7	46.5
		K.YLHDPVEVTVK.A	3	6.4	3.0	1.5	0.0	2.5	1.3	2.2	0.9
		R.DSISDSLNAPGIDLFR.R	2	46.2	46.7	34.2	39.5	24.2	26.7	37.8	30.8
		R.ELALQVAEAFGR.Y	2	38.7	37.0	160.4	156.4	36.0	39.9	175.6	159.0
		R.ESNVPLADIAAALAVQTR. N	2	30.8	42.3	25.8	36.5	21.6	28.6	31.6	43.1
		R.ISHVINYDIPHDTESYVHR. I	4	39.0	45.2	29.8	35.0	18.0	34.1	15.0	40.3
		R.ISHVINYDIPHDTESYVHR. I	5	28.7	28.2	19.2	15.6	15.6	28.4	16.1	32.6

		R.LDYSLVELPEK.L	2	22.3	19.6	18.5	16.1	3.0	10.6	3.4	7.7
		R.YGSHLPQVNVLPIYGGSS YTVQLSGLR.R	3	75.4	86.4	66.2	70.2	49.0	66.8	60.5	84.7
MSMEG _5048	MSMEG 5036	K.NLPATVEGANGLSDDPGT AAK.V	2	8.3	12.6	16.4	23.2	3.9	4.5	9.0	17.1
MSMEG _5049	MSMEG 5037	K.DGIQRPLIVFTPK.S	2	91.1	75.0	93.8	88.6	125.3	89.5	151.6	108.1
		K.DGIQRPLIVFTPK.S	3	139.7	125.7	127.1	118.6	130.3	138.1	131.9	129.4
		K.DVVIDM#LC*YR.R	2	8.9	7.6	19.2	22.8	4.5	5.2	20.0	21.7
		K.DVVIDM*LC#YR.R	2	8.8	7.7	18.7	22.8	4.4	5.2	19.3	20.4
		K.DVVIDMLC#YR.R	2	56.8	54.4	55.0	54.7	52.4	44.3	54.7	47.9
		K.DVVIDMLC*YR.R	2	56.7	54.3	55.0	54.7	52.4	44.1	54.7	47.7
		K.HEIEPSESVEADQQIPSK.L	2	5.5	3.7	4.7	2.6	3.8	2.2	2.9	1.3
		K.HEIEPSESVEADQQIPSK.L	3	104.2	86.8	128.4	91.2	38.0	42.9	45.4	48.6
		K.ISFTHLLGYAIVQAVK.K	2	40.6	37.2	51.2	50.0	45.0	32.2	54.8	41.9
		K.ISFTHLLGYAIVQAVK.K	3	94.1	91.5	87.8	86.6	84.7	78.6	85.3	74.7
		K.IYYELAAR.K	2	2.9	3.2	1.1	1.0	3.1	2.2	0.8	1.2
		K.LNAAEAFETFLQTK.Y	2	292.6	244.2	325.1	273.6	305.4	223.3	380.8	261.6
		K.LNAAEAFETFLQTK.Y	3	33.3	38.5	39.2	42.9	32.5	35.4	40.5	40.9
		K.LRDILSVLR.D	2	0.5	0.1	0.1	1.0	3.4	3.4	1.1	1.8
		K.LTAEDFSGVTISLTNPGTL GTVHSVPR.L	3	425.6	389.5	442.8	399.1	396.9	348.7	451.1	405.0
		K.M*IGAPIFHVNGDDPEAC# AWVAR.L	3	43.5	39.9	39.3	34.3	11.3	19.6	10.7	18.5
		K.MIGAPIFHVNGDDPEAC#A WVAR.L	3	43.5	39.9	39.3	34.3	36.4	43.1	36.3	43.7
		K.MIGAPIFHVNGDDPEAC*A WVAR.L	3	43.5	39.9	39.3	34.3	36.4	43.1	36.3	43.7
		K.NM#NASLEVPTATSVR.A	2	42.9	36.9	42.7	39.3	21.4	22.6	40.0	37.8
		K.NM*NASLEVPTATSVR.A	2	88.2	110.6	72.0	78.2	85.3	131.7	83.7	98.6
		K.NMNASLEVPTATSVR.A	2	304.9	326.9	331.7	322.8	288.4	308.0	326.2	300.2
		K.PTAITPAHTNLGLAIDLQG K.D	3	51.9	43.9	36.4	35.4	27.6	27.6	14.0	19.0

K.PYSQIFSEFEGNLNPSQA HGSGDVK.Y	3	158.2	143.8	145.1	125.3	112.6	114.3	103.1	89.7
K.TGEEFTPLQLLATNPDGT PTGGK.F	2	139.8	124.0	152.7	132.1	129.9	108.7	145.5	126.3
K.TGEEFTPLQLLATNPDGT PTGGK.F	3	205.9	211.1	225.9	223.9	119.8	172.1	143.7	198.6
K.VHAVEQQEILDTAFG	2	628.3	600.7	885.8	780.6	491.6	420.5	879.0	677.1
K.WGQLSDVVLLLPHGHEG QGPDHTSGR.I	4	67.5	54.5	72.9	53.4	88.5	87.7	111.0	92.1
K.WGQLSDVVLLLPHGHEG QGPDHTSGR.I	5	42.3	37.2	34.0	29.6	56.0	60.9	54.4	53.2
R.ELEKHEIEPSESVEADQQI PSK.L	3	133.8	125.7	135.1	123.0	201.4	153.2	237.5	163.5
R.ELEKHEIEPSESVEADQQI PSK.L	4	247.1	279.1	224.3	249.2	227.1	300.1	217.4	278.2
R.FGQFIAAYEDIVR.R	2	377.8	327.8	404.8	326.4	400.2	314.6	489.1	311.0
R.GHLMADIDPLR.L	2	33.4	30.6	141.8	145.3	32.8	29.8	108.1	93.2
R.GHLMADIDPLR.L	3	58.3	42.8	38.2	25.9	43.3	43.2	27.8	28.6
R.GHNEGDDPSMTQPYMYD VIDTK.R	3	55.6	58.3	61.7	64.4	31.6	38.5	30.2	42.6
R.HFAVVDGKPTAITPAHTNL GLAIDLQGK.D	4	242.4	228.3	232.6	254.7	168.6	188.4	198.4	258.7
R.HFAVVDGKPTAITPAHTNL GLAIDLQGK.D	5	28.1	28.5	26.7	24.1	14.5	20.1	15.3	25.0
R.HVGVEYTHILEPEQQR.W	2	21.9	17.3	19.5	13.4	32.6	19.6	31.9	18.9
R.HVGVEYTHILEPEQQR.W	3	404.2	422.1	529.7	520.2	271.0	290.7	407.0	447.4
R.HVGVEYTHILEPEQQR.W	4	107.0	100.2	103.9	93.0	88.0	89.2	89.0	85.4
R.IEQLAPLPR.R	2	8.6	8.2	2.3	4.3	9.6	9.0	3.1	3.8
R.IGDAHLALPEGFTVHPR.V	3	87.6	78.2	81.3	72.3	93.7	82.3	95.4	77.1
R.IGDAHLALPEGFTVHPR.V	4	216.8	208.5	207.3	195.5	197.0	216.0	206.2	218.6
R.IIQGAESGDFLR.T	2	298.9	257.8	280.6	244.7	176.4	185.6	173.6	183.0

		R.KTGEEFTPLQLLATNPDGTPTGGK.F	3	120.3	115.2	129.3	110.3	124.3	121.8	139.7	127.6
		R.LNVLANIVGK.P	2	66.7	64.1	55.8	54.2	48.8	51.2	41.6	37.8
		R.LNVLANIVGKPYSQIFSEFEGNLNPSQAHGSGDVK.Y	4	66.5	80.9	61.3	69.3	48.0	72.7	52.4	72.5
		R.RLAETLDRYPNVK.E	3	2.5	2.0	4.4	4.1	2.2	2.7	3.8	5.0
		R.SHPDLDVNSHGLTLWDLDR.E	3	138.8	132.9	191.4	172.1	112.7	118.2	139.8	138.7
		R.SVLEEPMYTDGEGDR.N	2	93.0	79.7	96.3	80.7	30.6	40.8	32.2	41.3
		R.TIHQLLLDDDFFDEIFR.E	2	4.0	4.1	3.0	3.0	7.8	3.8	5.8	4.3
		R.TIHQLLLDDDFFDEIFR.E	3	49.8	46.2	38.7	42.5	54.9	41.8	52.4	45.1
		R.TTTAAPVTPPTPAPAPAPEPK.A	2	254.1	227.3	333.9	292.4	258.1	216.1	363.3	302.7
		R.TTTAAPVTPPTPAPAPAPEPK.A	3	143.3	176.5	208.6	239.4	93.7	155.8	153.0	231.4
		R.VIELIAAYR.N	2	162.2	158.1	171.1	168.5	152.3	156.6	179.3	180.9
MSMEG_5053	MSMEG5041	R.EAFLLYADAVK.E	2	14.6	10.7	21.8	20.6	16.4	16.0	24.2	23.3
MSMEG_5055	MSMEG5043	R.ALSIAYTPGVAQVSR.A	2	493.4	478.2	890.1	836.6	415.8	413.7	985.0	891.1
		R.LRPTFGAVNLEDISAPR.C	3	235.7	220.5	193.9	181.6	192.7	197.8	56.6	69.3
		R.SDFPNQINNVLAFPGVFR.G	2	271.6	251.7	166.9	154.2	233.9	219.6	163.2	163.5
		R.TPQVVIEDSEIFAAHEGGK.L	3	130.9	115.0	22.7	21.0	113.0	122.9	13.5	22.6
MSMEG_5058	MSMEG5046	K.ILDLSELLDR.K	2	37.3	34.8	65.4	73.5	87.7	75.9	86.5	74.3
		K.LSIFDAATGLNLTR.D	2	46.4	48.1	73.9	78.3	77.4	69.2	138.4	112.1
MSMEG_5062	MSMEG5050	R.GWPIGSYPTYAEAQR.A	2	48.0	55.4	90.6	100.4	56.8	66.0	81.0	77.3
MSMEG_5064	MSMEG5052	K.TLPADQVFLDLEDAVAADAK.A	2	82.7	91.8	62.6	63.6	35.6	40.9	30.5	34.2
		R.IGIEPQIEDALGLTNVEAIAAGPR.V	3	40.1	43.5	42.8	52.2	52.2	53.0	32.5	33.1

		R.VQALVLGPGDMAASLR.M	2	34.4	41.4	29.9	29.2	40.4	43.5	32.1	29.4
MSMEG _5073	MSMEG 5061	R.ADDIGAGAVTPAVGALLS VLAR.L	2	20.4	20.6	50.3	54.4	13.5	15.2	34.5	37.3
		R.ADDIGAGAVTPAVGALLS VLAR.L	3	11.1	10.2	24.5	22.7	6.2	5.9	15.4	13.9
		R.LTPVLIPLGDGLLAAVRD	2	0.8	1.0	100.1	113.2	0.4	0.5	96.3	117.7
MSMEG _5086	MSMEG 5074	K.VSSFQPFDGYTDSSATEK .K	2	55.9	55.5	114.9	104.1	18.3	23.6	73.0	76.4
		R.ALAGFGGLGLR.L	2	46.6	40.9	26.9	24.4	39.7	25.5	28.0	21.4
		R.LATTAPTTNPASASAVLAK .D	2	237.9	208.6	259.0	222.0	202.0	238.6	193.5	223.1
		R.LPGFLMDAPVILR.G	2	21.9	26.5	22.8	37.5	17.3	16.4	30.3	26.9
		R.NSVGLLDLATR.L	2	16.5	17.2	16.1	12.1	10.5	7.8	27.8	11.1
MSMEG _5104	MSMEG 5090	R.GAVAVYGIDKFPR.M	2	40.3	38.1	115.7	84.6	34.6	32.7	29.8	21.7
		R.LTGDDVPADLAALTGK.D	2	191.2	193.3	170.5	179.2	65.6	69.4	119.2	137.9
		R.LTGDDVPADLAALTGKDE DRDVEVVAVR.T	4	438.6	456.1	98.7	98.3	304.4	294.2	79.4	77.0
		R.NSLSGAVEVVK.R	2	41.3	27.7	41.3	35.9	18.2	14.3	46.9	46.0
		R.TTIADLNDKPADTHDVWL R.L	3	405.1	441.7	282.4	360.4	190.1	235.9	436.2	385.0
MSMEG _5117	MSMEG 5103	R.GVDDFEYQMLYNIR.D	2	47.7	46.5	40.3	27.0	66.5	60.6	56.2	37.7
MSMEG _5119	MSMEG 5104	K.DFVLAHSSAHPDVLR.T	3	0.6	0.3	226.9	214.1	0.2	1.2	132.4	140.9
		K.MGDDFLSATEALR.Y	2	6.5	10.2	116.7	147.6	5.0	7.9	106.4	121.4
		K.SAYQAEIDAPC*ELIDFWR. F	2	22.2	26.2	56.3	58.8	20.9	25.5	21.5	30.7
		K.YGLTGAVIADDR.T	2	2.3	2.2	149.5	121.9	3.9	3.4	81.4	72.8
		R.AADLLSGPWR.E	2	0.3	0.4	100.4	64.9	0.5	1.3	61.2	31.5
		R.TAVQQALDGLR.Y	2	30.7	23.6	115.3	71.4	43.4	37.7	93.0	47.9
		R.YGDVADLTNYGGAVIDDR .A	2	19.3	16.2	151.0	153.7	23.9	14.7	93.3	77.9
MSMEG _5121	MSMEG 5107	R.AGFVAGDPAVVAELLAVR. K	2	111.5	110.3	114.5	115.2	119.8	105.4	123.4	127.6

		R.DALAAGSSSPGYPTTAGTPELR.E	2	56.4	88.0	36.6	53.9	38.2	53.0	15.3	38.1
		R.GILVAPGEFYGPAGDR.Y	2	135.4	115.7	78.4	84.6	97.5	108.9	74.1	68.7
MSMEG_5122	MSMEG5108	K.AC#IEEC#PVDC#IYEGAR.M	2	99.0	102.4	141.0	138.2	106.3	116.9	158.2	150.3
		K.AC*IEEC*PVDC*IYEGAR.M	2	99.0	102.4	141.0	138.2	106.3	116.9	158.2	150.3
MSMEG_5129	MSMEG5117	R.IAELTAALQSLGLR.G	2	37.5	40.0	48.4	68.6	29.9	27.5	42.2	41.6
		R.LLFVHAHPDDETLTTGGTIAHYVAR.S	4	72.1	91.0	102.0	114.8	41.1	45.1	87.4	87.0
		R.YAQLAVDHADQLGGYR.I	2	90.8	71.1	24.0	19.6	53.1	58.0	26.6	25.0
		R.YAQLAVDHADQLGGYR.I	3	160.9	131.5	120.3	101.0	107.4	123.1	114.1	138.0
MSMEG_5132	MSMEG5120	K.ALDLPTLYASGR.A	2	22.0	22.2	62.8	73.1	18.0	17.1	65.7	66.6
		K.HIPPPSGDPEAPLQALVTNLDASAFLGR.L	3	85.9	106.7	207.3	248.5	43.6	74.9	141.0	234.4
		K.ITELLVTEGVER.T	2	66.1	69.9	203.7	172.8	41.1	42.6	145.4	132.0
		R.VVDIGKPDAWEVQGR.G	2	13.5	13.4	83.2	79.0	6.9	10.5	80.1	68.8
		R.VVDIGKPDAWEVQGR.G	3	77.7	77.4	187.9	156.6	46.8	63.7	147.4	153.7
MSMEG_5136	MSMEG5121	K.NQVVAFEADELDPDLR.T	2	113.0	122.4	0.6	2.5	19.8	20.4	2.2	0.9
		R.LVFTEDALPAVQPVNFR.L	2	150.9	182.2	3.6	4.4	27.2	29.9	2.7	1.6
MSMEG_5150	MSMEG5135	K.FPTFLDGIDAVR.R	2	9.8	10.6	31.6	29.6	15.7	17.1	36.8	34.9
MSMEG_5164	MSMEG5148	R.DAAALPLTTITAWETLFER.F	2	84.6	99.2	46.0	58.2	17.0	29.1	31.6	60.0
		R.LLADAADLVDR.G	2	17.0	4.7	947.0	913.8	2.4	3.0	397.7	444.7
MSMEG_5170	MSMEG5154	R.AQTFQVSITEPR.A	2	139.0	184.4	459.9	676.4	96.1	116.1	353.2	434.9
		R.NISGEHPDWDEFR.Q	2	15.2	18.3	16.0	20.8	8.0	3.2	17.3	10.4

MSMEG _5183	MSMEG 5166	K.DAVAVVTGGASGLGLATT K.R	2	183.4	193.7	194.1	194.0	142.2	148.6	237.3	244.7
		K.FVATDVTDEAGVTEALNV AESLGPVR.I	3	203.3	224.1	195.2	201.1	132.0	143.9	182.8	198.0
		K.GGVVGMTLPIAR.D	2	90.8	85.5	153.2	145.1	86.0	67.7	204.8	162.0
		K.NGPFPLDGFR.K	2	57.6	55.0	39.1	42.1	33.8	39.3	36.6	41.4
		K.TPLLGSLPEEAQK.S	2	290.6	281.3	460.2	443.1	227.3	229.0	458.6	421.6
		R.LLDAGAQVVVIDLK.G	2	83.8	83.7	194.8	189.4	23.3	29.0	271.8	307.3
		R.VVTIAPGLFK.T	2	159.0	156.2	164.5	163.8	124.7	123.8	167.8	178.9
MSMEG _5184	MSMEG 5167	K.GLGLDGADLPAQNDISR. W	2	64.6	71.5	438.2	638.3	41.7	45.7	258.5	260.2
MSMEG _5191	MSMEG 5175	K.AYVHLIDLHR.Q	2	8.1	6.1	8.9	9.8	4.2	3.9	9.4	6.6
		K.AYVHLIDLHR.Q	3	76.1	68.7	32.9	27.1	54.9	53.3	28.7	27.0
		R.ASVWYGATLR.A	2	39.3	35.3	20.2	13.2	20.9	27.3	18.1	21.5
MSMEG _5197	MSMEG 5179	K.SGVHGPALSLHNDVVGPY FK.E	3	35.4	39.6	25.0	39.1	38.0	46.8	34.9	54.5
		R.FNAIIDEEIAK.S	2	35.7	30.2	42.3	65.7	61.0	51.1	26.8	27.7
MSMEG _5198	MSMEG 5180	R.NAINSSVSQAVGDALAEA QNDPEVR.A	3	37.3	41.3	35.5	33.5	68.7	75.1	42.5	57.8
		R.VAYGAEEGTVPAEQPSW K.R	2	33.3	42.1	16.6	24.5	48.9	61.0	10.9	20.8
MSMEG _5199	MSMEG 5181	K.AAAAQDSGAFSDQIVGIK. T	2	242.1	229.0	201.2	162.5	280.2	287.8	181.2	169.6
		R.TAVLSAGWPETVPGVTV DR.Q	2	193.2	206.5	204.9	221.5	238.8	244.0	193.9	265.1
MSMEG _5211	MSMEG 5192	K.NFLVTQGDETVGVLK.I	2	87.7	83.3	127.2	125.9	82.9	73.2	118.8	116.1
		R.FNYASVVEFSER.L	2	62.0	64.3	35.3	38.4	62.2	64.5	46.9	39.0
		R.GYLRPSTVAELGALAGR.V	2	23.7	25.6	36.8	27.3	22.7	35.6	23.3	50.5
		R.GYLRPSTVAELGALAGR.V	3	53.9	49.6	63.1	55.8	54.0	47.7	80.2	86.4
		R.LASLLPDPLDTVFLVNSGS EASDLALR.L	3	44.4	62.6	39.0	54.5	25.7	57.6	29.2	63.4
		R.PLFGLPALATDSTDADLV AR.R	2	124.0	123.6	157.6	146.6	120.1	123.9	159.6	154.2

		R.YADEAVAQIQELAAAGR.A	2	104.1	82.9	88.9	102.9	112.0	85.5	123.0	99.2
MSMEG _5215	MSMEG 5199	R.GLGYEIASAFGR.S	2	3.4	2.7	2.2	3.7	2.9	3.6	2.3	1.5
MSMEG _5225	MSMEG 5209	R.FALYSTGTPDSAESEPAA R.S	2	149.9	143.0	260.0	273.0	235.7	320.3	276.5	305.7
		R.LLVGAAVTTLEEAR.K	2	59.7	57.0	459.5	446.8	326.8	294.3	521.5	471.2
		R.LTSLSIGDLEALLAYEEAG R.A	2	26.7	35.7	114.1	122.7	75.3	103.7	98.2	122.9
		R.LTSLSIGDLEALLAYEEAG R.A	3	8.1	6.6	40.4	39.5	37.5	36.6	40.1	38.8
MSMEG _5239	MSMEG 5224	K.GMPNAISVLAVAER.G	2	201.8	195.1	246.6	259.0	72.9	78.3	164.8	164.4
		K.IAAGPDVADFIDITSPIAANI QR.I	2	332.8	356.6	339.7	358.8	235.3	227.6	275.0	309.0
		K.IAAGPDVADFIDITSPIAANI QR.I	3	349.0	361.3	314.9	327.5	284.3	275.9	344.9	349.0
		R.GAMFDPSAVFYMNK.I	2	135.4	115.6	127.9	114.4	17.7	19.9	21.1	18.0
MSMEG _5240	MSMEG 5225	K.DLGLLDPEKADAIIAAAGEI AEGK.H	2	118.7	129.7	111.6	153.2	104.9	113.3	116.8	147.8
		K.DLGLLDPEKADAIIAAAGEI AEGK.H	3	944.8	898.1	1053.2	1013.3	1106.9	1053.0	1465.8	1299.3
		K.VVEVLVNETGLAELR.T	2	109.2	104.5	443.9	484.3	99.8	97.8	482.9	469.9
		R.AVENFPISFR.G	2	357.1	259.5	300.7	226.2	446.0	383.7	454.2	418.5
		R.DGLVEASGALR.T	2	80.3	63.7	71.8	61.6	77.8	58.0	60.4	49.6
		R.ELAESSPSIVTPLNSAIGY EEAAK.V	2	317.7	357.9	332.2	388.0	286.9	289.5	355.4	383.6
		R.HLIPALEVLHASLAAK.A	3	427.3	292.2	404.8	282.4	325.4	355.1	381.8	446.1
		R.LGELAIGGTAVGTGLNAP EGFGAK.V	2	492.7	536.8	415.2	455.9	591.2	566.1	546.7	550.8
		R.NLLESFTLLSNVSR.L	2	436.2	442.3	535.1	522.5	512.6	422.4	819.2	711.9
		R.TAVDSFEAQAAR.D	2	35.3	36.4	40.9	48.3	16.6	15.1	15.8	17.9
		R.THLMDAVPVTLGQEFGG YAR.Q	3	387.4	343.1	320.5	281.7	219.0	215.8	234.0	227.0
MSMEG _5243	MSMEG 5228	R.IVTTVDNTSHIFPINFVVQN R.T	3	263.5	274.0	15.7	17.8	61.2	71.8	19.4	17.8

MSMEG _5244	MSMEG 5229	R.VLLDLLGEGLTNK.Q	2	6.6	0.0	8.7	0.0	1.2	0.9	2.0	2.7
MSMEG _5245	MSMEG 5230	R.ALATAEVAIR.Q	2	32.2	26.2	1.3	0.7	2.3	2.8	0.2	0.3
		R.GGIGSVASAVLR.S	2	162.9	159.0	3.1	2.8	25.7	25.5	13.4	13.6
		R.ILVEFDGRPSASTALQTGVDEAR.L	3	342.1	370.1	37.3	47.5	48.3	55.4	17.4	23.1
MSMEG _5246	MSMEG 5231	K.VLLEC#TMSGLATC#PVTHVTELHTSR.D	4	226.2	214.0	3.6	4.0	10.3	9.7	16.0	11.0
		R.IGLAPALDEVPPPTPR.R	2	816.7	840.6	250.1	250.1	105.8	113.3	253.6	272.9
		R.LGDGPVHMDTLGEDVREEVAEAAALTESLR.L	4	69.8	75.8	4.7	5.8	4.3	8.2	4.1	7.0
		R.RPVDAFLEVRPR.S	3	34.5	29.8	3.6	4.7	1.6	2.2	4.4	2.3
		R.VALAAAGWDTEVQR.F	2	789.2	794.2	267.5	197.3	145.9	165.0	579.6	563.9
MSMEG _5248	MSMEG 5233	K.SITDALEILLITK.D	2	58.4	47.6	16.2	17.2	40.4	33.4	20.0	18.5
		R.AAGLDVIGADIAPYQDK.V	2	337.7	359.8	114.8	128.6	280.0	293.9	142.2	152.8
		R.IATDEERHEEFFSNLVSYVLDK.H	3	81.3	58.4	19.3	14.5	67.1	70.0	26.4	26.6
		R.ITAWGLAGEASLK.D	2	80.7	87.3	57.6	71.0	69.8	61.4	38.9	56.6
		R.NLQAQITEPVLASLMGR.I	2	148.2	166.5	106.5	107.0	109.3	114.5	117.1	124.3
		R.NLQAQITEPVLASLMGR.I	3	189.1	219.8	78.6	82.1	159.5	213.4	75.5	90.9
MSMEG _5249	MSMEG 5234	K.HINDLSEADWETLR.N	2	316.9	353.5	233.1	279.4	257.8	277.6	219.5	218.0
		K.HINDLSEADWETLR.N	3	136.0	118.8	60.3	53.2	120.9	117.4	69.7	64.8
		R.EFKPLVLVAGYSAYPR.R	3	54.4	52.2	29.1	32.9	54.6	51.9	41.4	47.7
		R.FYAGC#QNVDTVESVAAEHAR.E	3	848.5	970.2	1157.8	1245.4	896.3	907.1	1285.7	1294.4
		R.FYAGC*QNVDTVESVAAEHAR.E	3	848.5	970.2	1157.4	1245.4	896.3	907.1	1285.1	1294.4
		R.GGMVLATEEYAPAVDK.G	2	462.5	447.0	759.9	733.5	226.3	243.6	804.0	913.2

		R.NSIPADPNGAWYTSGVR.L	2	309.0	357.6	291.8	377.0	254.1	258.4	212.0	292.5
		R.SYGTNPETGFLDYDAVAAAAR.E	2	374.2	371.8	237.7	270.8	334.2	330.0	284.6	302.2
		R.VHAASSELLSANPLYPGLTL	2	364.4	360.0	325.9	335.8	333.3	311.0	396.3	367.0
MSMEG_5252	MSMEG 5236	K.LDLLEVEEVYLPLAR.L	2	54.6	55.5	56.5	59.9	26.2	29.6	16.1	25.7
		R.IEDIEQWYISR.F	2	24.7	26.6	20.1	18.7	35.4	32.5	21.5	32.4
MSMEG_5258	MSMEG 5243	R.YLDTVAGGSAADIAALYADDATVEDPVGSGEVHIGR.Q	3	70.4	88.8	64.2	82.7	65.4	85.1	54.9	62.5
MSMEG_5263	MSMEG 5248	K.AELDQLIANRPVIAAEINDR.R	3	297.4	355.6	215.1	244.1	312.7	332.0	193.2	197.2
		K.LEVYSPNSPLGGALLDAK.V	2	123.9	125.2	118.4	117.0	105.2	109.7	98.3	89.2
MSMEG_5265	MSMEG 5251	K.VFTHWGISHTPAALSDLDSVR.S	3	190.6	197.0	145.4	136.7	206.2	155.3	155.5	179.8
		K.VLVDNTFASPALQQPLLLGADIVLHSTTK.Y	3	792.6	663.9	555.2	487.3	513.8	558.1	572.7	605.3
		R.AVLRPGDHVVIPDDAYGGTFR.L	3	387.2	382.8	246.9	270.6	274.3	280.6	257.2	253.8
		R.AVLRPGDHVVIPDDAYGGTFR.L	4	387.1	343.0	169.3	155.7	286.4	285.2	145.4	176.4
MSMEG_5270	MSMEG 5255	K.ITHFVAGVGTGGTITGTGR.Y	3	127.7	116.6	108.0	101.9	106.0	111.1	119.9	113.4
		K.LADAVAEHMSPPLPLIGAGELVSTAAK.T	3	437.0	404.9	345.7	326.6	305.1	255.1	251.6	241.0
		K.SGALPDLVHTHPSETVR.D	4	13.3	10.4	10.4	14.6	4.3	6.5	4.1	4.1
		R.ELLSAVFEGR.A	2	63.3	41.9	54.1	38.5	52.1	54.0	38.1	57.7
MSMEG_5273	MSMEG 5258	K.AGEGHAFISAGVETVSR.F	2	413.5	384.6	290.0	281.8	348.8	321.3	333.9	330.8
		K.AGEGHAFISAGVETVSR.F	3	627.9	514.2	453.2	393.7	502.0	417.0	464.9	427.3
		K.GSLVEMRPDDLAAQMVK.A	2	258.4	266.1	186.6	217.9	40.8	42.2	43.1	46.4

		R.AVAVQLGYDFLPGTTVNR.Y	2	1250.5	1242.7	1422.8	1401.5	1115.8	1045.3	1413.5	1455.9
		R.EITPVTLPDGTVVSK.D	2	2184.3	2184.0	2873.9	3045.1	1553.8	1706.4	2680.3	2906.8
		R.ITATLLNNLQTHDK.T	2	192.9	167.7	267.0	290.4	215.0	197.4	293.5	229.0
		R.ITATLLNNLQTHDK.T	3	181.1	157.0	124.3	109.9	237.0	152.2	219.3	132.9
		R.IVSTGVSGLSPEIMGLGPIEAVK.K	2	727.2	728.0	586.1	596.3	286.5	274.6	273.2	275.0
		R.IVSTGVSGLSPEIMGLGPIEAVKK.A	3	214.9	192.8	200.8	188.1	185.3	184.6	206.7	200.1
MSMEG_5274	MSMEG 5259	R.LAAETLGVPTSPYR.F	2	117.5	114.3	328.1	359.6	150.2	136.3	339.7	360.9
MSMEG_5285	MSMEG 5270	R.IGEIAAQTQTVPESVR.R	2	161.4	164.7	22.7	23.1	39.2	46.7	14.4	17.1
MSMEG_5358	MSMEG 5339	K.GVDESDVLQDAIAVAADYNR.T	2	124.9	138.9	76.7	78.2	77.7	77.2	82.1	71.7
		K.GVDESDVLQDAIAVAADYNR.T	3	67.4	74.2	19.9	21.3	56.8	54.7	35.2	34.4
MSMEG_5359	MSMEG 5340	K.ADAAGLVVAAR.I	2	0.1	0.0	2.4	0.0	0.0	0.1	3.0	2.1
		K.HLDAPLVWC*VAALLGQHPLSAEQAEK.V	3	2.5	0.0	38.9	0.0	4.1	3.1	42.5	41.0
		K.HLDAPLVWC*VAALLGQHPLSAEQAEK.V	4	0.3	0.4	36.1	33.5	0.5	0.6	34.8	38.3
		R.FHEALNVYGPALK.E	2	4.4	4.7	327.2	403.7	6.2	6.4	386.3	342.9
		R.FHEALNVYGPALK.E	3	4.6	6.2	124.8	130.5	9.0	7.3	137.5	124.5
		R.GIDPALQSDPTIYR.F	2	33.3	37.4	456.3	497.8	37.0	41.7	471.9	436.8
		R.NGLSWADIAK.H	2	5.1	4.2	64.6	64.3	3.9	5.6	48.2	56.2
MSMEG_5397	MSMEG 5377	R.EVIDILGLR.K	2	7.9	6.2	35.9	21.3	7.5	5.2	43.1	20.4
MSMEG_5404	MSMEG 5383	R.ALFESSITDPATFWADAAK.A	2	72.6	78.1	42.9	46.5	24.5	31.5	44.7	49.5
		R.GDQAALIYDSPVTGTK.R	2	85.8	77.1	51.6	69.5	30.0	29.3	45.3	86.4
		R.GIADGKDEPVPSTIEDPSVLDDLK.S	3	101.0	107.3	51.4	65.1	38.8	48.3	38.6	62.8
MSMEG_5413	MSMEG 5393	R.LGQGVDATGQFAPEALAR.T	2	22.9	27.1	294.8	325.0	26.0	26.3	171.6	186.4

MSMEG _5415	MSMEG 5395	K.AAAESAGLPLFR.Y	2	37.5	38.6	73.6	84.3	46.3	48.9	76.3	89.4
		K.AALDLISSAIEATGLK.L	2	1929.3	1638.3	2002.6	1797.6	1555.6	1344.0	1924.2	2015.5
		K.AALDLISSAIEATGLK.L	3	127.0	150.5	87.8	111.0	132.8	114.5	117.0	116.4
		K.AVEAVLDEIAPQVIGLSADDQR.L	2	374.3	436.3	256.8	293.1	193.5	265.1	171.4	255.7
		K.AVEAVLDEIAPQVIGLSADDQR.L	3	807.8	723.4	646.4	605.8	712.7	595.3	855.8	755.3
		K.LGSDVALALDVAATEFYTEGSGYAFEK.E	3	84.8	92.2	69.3	78.2	28.7	43.7	36.7	40.6
		K.NQGLATGLGDEGGFAPDVAGTK.A	2	1227.9	1364.3	1038.8	1041.3	813.5	815.0	1168.5	1116.4
		K.VNQIGTLTETLDAVSLAHNSGYR.T	3	2712.1	2479.4	2108.9	1957.8	2099.6	1926.6	2511.8	2277.6
		R.AAVPSGASTGEHEAVELR.D	2	75.7	48.7	55.6	38.0	72.3	49.4	74.5	52.6
		R.GNPTVEVEVALTDGTFAR.A	2	337.9	299.0	338.7	333.4	210.2	213.6	394.5	405.3
		R.IQLVGDDLFVTNPER.L	2	1882.1	1783.1	1891.5	1811.8	1725.9	1573.0	2529.7	2217.1
		R.LVDQALLDLDGTPDK.S	2	1829.6	1778.9	1371.6	1427.5	1380.2	1273.0	1445.0	1443.2
		R.SGETEDTTIADLAVAVGSGQIK.T	2	430.5	434.8	366.7	407.0	331.5	286.4	393.7	375.7
		R.SGETEDTTIADLAVAVGSGQIK.T	3	249.9	221.3	240.4	222.9	156.1	134.2	264.0	224.7
MSMEG _5419	MSMEG 5399	K.ITGEEDIFSHTDLWDFR.A	3	179.6	159.4	83.3	75.5	66.8	57.5	133.9	99.1
		K.SNQITVEASDTEC#K.L	2	35.6	27.5	19.1	14.7	9.0	8.3	13.0	12.3
		K.SNQITVEASDTEC*K.L	2	35.6	27.5	19.1	14.7	9.0	8.3	13.0	12.3
		R.ANVEGSQTAVASVR.P	2	18.1	15.9	13.7	11.3	1.4	1.3	9.4	8.0
		R.ANVEGSQTAVASVRPILDER.D	3	264.3	250.7	72.4	71.6	62.3	52.7	54.3	38.9
		R.FADVEALLEK.Y	2	259.2	216.6	62.0	62.6	95.4	101.4	86.4	80.1
		R.IEPVAESFPDDLDPR.I	2	423.5	435.5	145.4	171.9	152.9	154.3	155.3	149.3
MSMEG _5423	MSMEG 5404	R.AHVATGGYAAVVTPGIGTAHR.V	3	15.4	11.6	8.1	8.5	10.6	9.0	17.9	11.6
		R.EFGITEIGAVSASTVR.L	2	29.8	23.4	60.5	47.2	19.7	16.8	59.8	50.8

		R.GYAYFLYPPNKPLTETAYDR.L	3	49.9	54.2	12.9	17.8	26.1	28.2	12.9	13.0
		R.LAAAADDDAVASVVDELIDR.Y	2	34.5	34.4	12.1	12.2	22.7	23.2	9.5	12.1
		R.LARPDDETLGAPLR.V	2	17.6	14.4	10.7	12.1	11.7	11.9	7.7	10.0
		R.LATIAQNNELGAGM*AVAM*K.D	3	2.6	2.3	30.7	14.7	0.5	0.5	21.7	14.1
		R.LSPMVLPDSAQLR.L	2	28.4	31.8	28.0	43.2	17.0	20.6	33.8	48.8
		R.SIPEVPVQTVVAVPC#R.E	2	20.8	19.6	49.7	57.6	14.4	13.1	32.9	42.1
		R.TLEMSLAGIR.E	2	10.7	11.0	13.3	13.2	5.9	6.9	7.8	9.4
MSMEG_5424	MSMEG 5405	R.VALALLTYVEEHTDGFR.I	3	7.6	6.6	27.0	27.1	2.1	2.5	25.1	34.1
MSMEG_5427	MSMEG 5408	K.DVIIAATHGVLSDPAPQR.L	2	76.9	81.5	41.3	41.2	44.8	50.4	12.8	16.3
		K.DVIIAATHGVLSDPAPQR.L	3	164.2	227.5	119.9	132.0	131.1	141.0	58.9	58.0
		K.ELDVAVTAQTAR.D	2	96.3	89.6	138.1	149.1	81.4	74.2	79.6	78.7
		K.RFPQLTVLSIAPLLANTIR.A	3	70.3	69.6	38.9	37.4	73.6	58.7	24.8	25.6
		R.DFANGEIFVR.F	2	38.8	34.9	5.9	4.4	22.6	25.1	0.7	1.0
		R.EVIVTNTLPITEDK.R	2	377.1	443.8	84.1	115.2	399.5	407.9	65.4	81.2
		R.EVIVTNTLPITEDKR.F	2	19.2	14.9	7.6	11.9	19.1	13.6	5.3	8.5
		R.FPQLTVLSIAPLLANTIR.A	2	49.1	49.8	21.4	22.3	36.3	39.2	9.4	12.5
		R.FPQLTVLSIAPLLANTIR.A	3	25.6	26.8	8.9	8.8	18.5	18.9	1.7	3.0
		R.ITAILPFYPYAR.Q	2	170.5	171.4	104.3	100.9	142.0	142.8	64.6	63.4
MSMEG_5431	MSMEG 5412	K.VPAVLYGHGTDPQHLELNAR.D	3	769.2	805.7	676.1	674.6	825.7	962.2	627.5	708.7
		K.VPAVLYGHGTDPQHLELNAR.D	4	1056.0	982.1	746.8	693.7	1412.5	1348.2	872.8	818.9
		R.NIQHADLLVVQR.G	3	52.6	40.0	22.3	16.2	80.2	65.7	28.7	18.7
MSMEG_5435	MSMEG 5415	K.AVIISEPFLVATPVLEEK.G	2	134.2	140.2	39.4	38.6	163.1	171.8	80.0	69.2
		R.IEHQVAHEVVSEVDVRPR.N	4	327.1	284.8	177.4	170.0	307.0	395.8	134.5	218.1

		R.IEHQVAHEVVSEVDVRPR.N	5	26.3	16.5	0.0	0.2	41.2	47.4	0.5	0.5
		R.LASLGPLLNDLEAR.V	2	78.0	73.1	25.3	33.2	111.2	74.1	20.2	13.7
		R.NVVVLGPGSIPK.T	2	46.3	41.8	67.4	54.3	58.0	34.4	54.3	54.3
		R.RIEHQVAHEVVSEVDVRPR.N	3	38.0	25.9	317.5	378.9	68.2	64.7	30.8	42.1
		R.RIEHQVAHEVVSEVDVRPR.N	4	277.9	275.4	61.6	79.3	335.1	425.7	64.1	80.8
		R.RIEHQVAHEVVSEVDVRPR.N	5	174.2	163.5	15.9	17.6	252.2	293.5	7.8	19.6
MSMEG_5438	MSMEG 5418	R.VTAVEIDPLLAR.Q	2	182.5	162.2	527.8	496.8	219.1	198.8	451.0	444.7
MSMEG_5441	MSMEG 5421	R.VNADLANELGNLAQR.S	2	87.1	87.9	88.3	72.4	87.9	76.1	68.2	69.5
MSMEG_5471	MSMEG 5450	K.ELLPVVDTPGIELVAAEAAEAGAER.L	3	168.6	169.3	151.7	147.0	172.3	207.7	211.6	200.2
		K.GMVEKPRPEDAPSHFAAAGR.Y	4	2.4	1.6	2.3	1.3	2.0	2.0	1.2	1.6
MSMEG_5479	MSMEG 5458	R.FDAVQAFSDDALTTC#PK.C	2	43.2	46.5	0.4	0.6	31.9	30.8	0.0	1.1
MSMEG_5485	MSMEG 5465	R.ASGLAAGIVDAGLSR.G	2	138.6	147.7	156.9	171.0	151.0	158.9	171.1	163.0
		R.ELLGISEALR.A	2	135.7	139.9	134.5	126.8	163.5	155.8	145.5	129.0
		R.GLAGISGSTLVVNIAGSR.A	2	178.8	196.4	402.2	409.4	178.2	179.5	458.2	412.4
MSMEG_5486	MSMEG 5466	R.VIGSADALVAAVR.S	2	29.2	19.7	26.8	14.2	11.2	13.0	14.9	21.4
MSMEG_5488	MSMEG 5468	R.VAGLDAGADDYLPKPFALEELLAR.M	3	45.8	52.3	90.7	88.3	37.2	39.2	112.3	116.7
MSMEG_5489	MSMEG 5469	K.AEAPGLVTVSVAGQQR.K	2	499.5	512.7	205.8	208.7	475.2	531.0	227.4	243.2
		K.AEAPGLVTVSVAGQQR.K	3	51.3	48.8	24.0	20.3	44.4	53.1	28.7	35.5
MSMEG_5512	MSMEG 5490	K.DVVAALPDLPAIDAIAHR.L	3	60.4	50.5	68.8	67.4	36.4	41.0	68.1	70.9
		K.LATPDTSVADLVGDIDPIK.V	2	122.7	124.7	189.6	182.9	91.3	87.6	208.5	201.2

		R.DVWPGILGFEDTVIPQLER.A	2	106.8	126.1	70.1	84.1	62.0	78.9	48.7	69.7
		R.GIVAVNELPDLAER.I	2	111.9	103.3	105.1	106.5	89.1	91.2	156.7	143.4
		R.SLGDPETIAYGLIPR.A	2	76.6	74.2	56.8	55.5	43.1	42.2	62.9	61.8
		R.VVDLGTVIDVLR.G	2	55.9	51.0	53.5	50.6	43.2	34.6	61.0	52.9
MSMEG_5515	MSMEG5493	K.HANPC#GIAISSTSVADAHR.K	3	25.9	27.9	19.9	20.8	23.4	22.4	21.7	25.8
		K.HANPC*GIAISSTSVADAHR.K	3	41.9	38.5	33.1	27.9	35.8	30.9	25.7	30.6
		K.TLEDLVFAWR.A	2	26.3	28.7	17.5	21.2	19.4	20.5	21.1	27.2
		R.GLHQAGVAIVSTGSTAK.T	3	26.7	21.7	24.0	23.2	18.9	18.7	22.0	23.3
MSMEG_5520	MSMEG5498	K.LLQDIWAAAGR.E	2	41.6	41.2	10.7	11.9	72.6	69.5	14.8	18.7
		K.PVQAHIPVLVGAAGNEK.N	3	128.0	112.3	43.6	45.0	153.7	143.8	17.5	23.0
		R.TLDPWVSLGTAAAVTSR.V	2	61.3	56.6	68.6	61.6	97.8	89.5	53.6	62.1
MSMEG_5524	MSMEG5502	K.AGTQIVGGVNAR.K	2	8.4	5.9	10.7	7.2	17.8	10.8	13.5	9.4
		K.ANVTKPVVGYVAGFTAPEGK.T	2	444.1	470.9	472.3	485.5	740.7	745.8	531.8	515.3
		K.DKDGNDIELPVFGSVAEAIK.E	2	227.5	213.7	217.4	197.1	361.6	326.6	209.4	200.0
		K.DKDGNDIELPVFGSVAEAIK.E	3	711.1	668.4	674.1	668.7	1147.4	1067.7	699.4	680.1
		K.ETGADVSIAFVPPAFSK.D	2	1178.8	1243.5	1314.0	1364.1	1720.8	1669.2	1297.8	1237.6
		K.SGTLTYQM#MYELR.D	2	58.1	67.0	510.0	660.3	89.0	85.4	289.7	398.4
		K.SGTLTYQM*MYELR.D	2	57.8	65.4	265.2	219.5	89.0	85.4	289.7	237.2
		K.SGTLTYQMM*YELR.D	2	58.3	66.7	265.2	219.5	92.5	87.4	383.1	237.2
		K.SGTLTYQMMYELR.D	2	243.3	210.4	265.2	219.5	479.0	338.7	383.1	237.2
		K.TM*GHAGAIVSGSSGTAAAK.K	3	14.5	4.7	18.9	8.0	23.9	15.3	15.8	9.8
		K.TMGHAGAIVSGSSGTAAAK.K	2	1.7	0.8	0.9	0.3	5.1	2.1	1.9	0.6

		K.TMGHAGAIVSGSSGTAAAK.K	3	14.5	10.8	18.9	13.9	23.9	15.3	15.8	9.8
		K.VIVMIGEIGGDAEER.A	2	130.1	134.0	93.2	107.6	181.8	170.5	71.7	68.0
		K.VIVQGITGGEGTK.H	2	11.1	10.3	4.5	3.8	20.4	19.9	2.3	4.8
		R.IIGPNC#PGIITPGEALVGITPNNITGK.G	3	267.0	211.1	322.0	284.5	600.0	654.3	470.0	529.3
MSMEG_5525	MSMEG 5503	K.EDATLVEVNPLVR.T	2	2176.5	1973.4	2051.5	2005.9	2448.3	2594.8	2248.8	2272.1
		K.LWEVFVK.E	2	41.0	19.5	6.7	6.2	84.5	87.9	32.0	33.1
		K.TIAEEIGRPVMVK.A	2	5.0	5.2	293.2	289.1	23.3	25.5	223.6	296.8
		K.VTLDENAGFR.Q	2	383.6	285.9	357.4	282.9	373.7	321.9	330.8	286.5
		K.YAATPDDAFTHANNILGLDIK.G	2	470.3	512.4	365.7	402.4	526.2	500.6	369.8	352.8
		K.YAATPDDAFTHANNILGLDIK.G	3	2257.2	2135.8	1733.9	1642.0	2512.2	2498.2	1939.9	1812.3
		R.ILAEANHPLVIQAETM#DAGADK.A	3	425.0	437.6	354.5	376.1	455.9	416.8	311.5	313.3
		R.ILAEANHPLVIQAETM*DAGADK.A	3	481.4	500.1	397.7	434.0	564.2	532.8	397.2	402.7
		R.ILAEANHPLVIQAETMDAGADK.A	3	2364.7	2309.6	2245.4	2242.0	1473.7	1388.9	1292.7	1231.2
		R.SIAEQGHLPAEVLDAAAVTIQK.L	2	657.7	737.3	607.2	669.3	659.1	659.0	580.9	586.8
		R.SIAEQGHLPAEVLDAAAVTIQK.L	3	2703.8	2436.5	2663.4	2432.5	3224.1	2943.5	3125.7	2720.4
		R.SIAEQGHLPAEVLDAAAVTIQK.L	4	85.6	74.2	47.1	37.6	124.1	108.8	43.4	45.0
		R.TPDDQILALDGK.V	2	202.3	218.5	201.1	207.2	199.4	189.2	165.6	166.6
MSMEG_5534	MSMEG 5512	R.GKLDEELGDLVEAVLER.T	3	4.0	4.2	0.1	0.0	8.2	9.5	0.2	0.1
MSMEG_5538	MSMEG 5516	K.GEVAYGAEFFR.W	2	96.2	64.2	66.5	56.1	61.8	54.3	51.8	50.0
		K.GLDPSSTLGPLINSK.Q	2	306.2	319.5	202.2	233.0	161.0	162.3	203.3	238.2
		R.EGGIEGIEEYLDTK.Y	2	150.3	133.8	54.4	46.3	105.6	91.3	90.5	73.2
		R.GVISDPAAPFGGIK.E	2	258.1	253.2	129.5	102.0	234.9	147.2	196.9	126.8
		R.IEDLISSVPTGLWIGGEER.E	2	212.6	213.8	123.5	131.4	143.8	162.3	88.7	130.4

		R.VAETIQSGMVGINR.G	2	103.9	82.2	99.1	70.1	144.0	88.4	63.5	55.3
MSMEG_5541	MSMEG5517	K.LVVDGQDVVADVHDVLDR.M	3	487.6	470.5	342.4	333.6	433.8	362.9	343.3	311.8
		K.TFSTLETLTNATAAR.R	2	603.4	582.3	426.5	416.2	392.1	369.3	444.8	389.0
		R.FVSNVDPADLVAK.L	2	600.1	605.9	350.0	364.6	540.5	498.0	540.2	471.6
		R.HYADAGISAR.F	2	10.4	4.4	5.7	2.2	2.9	2.7	1.6	1.8
		R.TLDLLVDLAR.A	2	102.6	92.3	36.7	44.5	69.5	54.0	27.9	29.0
MSMEG_5553	MSMEG5530	K.IYATDVDEDALTEAR.A	2	60.8	55.7	153.4	138.6	71.0	74.1	110.1	115.2
MSMEG5534	MSMEG5534	K.GVTDVIVGFR.I	2	38.6	40.0	46.4	49.3	28.9	26.7	34.5	32.9
		R.IPYIEGQDTEPLDKK.I	3	73.5	79.9	21.9	23.4	43.5	52.5	10.7	18.2
MSMEG_5592	MSMEG5572	R.EEFEAAELPFPSAR.E	2	46.6	60.2	113.8	139.9	55.0	60.2	145.3	160.6
		R.FLGHLTDEQLLR.H	2	23.2	36.0	58.5	73.7	9.7	27.6	52.8	66.1
		R.FLGHLTDEQLLR.H	3	3.4	4.0	27.7	24.4	4.2	4.9	31.0	35.5
MSMEG_5612	MSMEG5590	R.DLDGNLVGSSSYLNVDGTNR.R	2	6.0	9.0	45.8	53.3	6.4	13.3	24.5	39.5
		R.RLEIGHTWYTAAAR.G	3	132.1	122.2	38.6	41.1	214.5	213.5	34.7	48.6
MSMEG_5615	MSMEG5592	R.SELTVNQTSLPAGYSGPR.S	2	34.2	44.1	58.4	67.9	25.4	33.1	46.4	51.5
MSMEG_5634	MSMEG5613	R.YHEWLSIHR.A	3	23.6	15.5	20.6	13.0	18.2	19.0	10.6	12.9
MSMEG_5639	MSMEG5618	K.LDAETALQTGMANR.I	2	268.4	255.3	184.0	170.3	216.4	203.1	163.1	168.1
		K.LDAETALQTGMANR.I	3	17.8	20.8	7.8	5.6	16.1	15.9	4.5	3.6
		K.WAAEIAGFAPLALQHAK.R	2	65.8	53.4	126.5	127.2	48.9	53.8	71.0	86.6
		K.WAAEIAGFAPLALQHAK.R	3	101.4	97.1	87.3	84.8	76.6	76.0	57.6	63.9
		K.YGLALDNWSIR.R	2	148.3	145.8	90.1	92.3	127.9	119.4	73.3	74.3
		R.DGNVLTLELQRPER.R	2	132.5	119.9	61.2	54.3	118.0	109.5	93.2	91.8
		R.NALNC#ELVDSLR.E	2	215.2	226.5	115.2	116.2	198.0	197.2	112.5	104.2
		R.NALNC*ELVDSLR.E	2	208.0	199.1	112.2	102.4	183.2	188.2	102.4	98.5
		R.VLNDDGAYEEPWPAHK.E	2	45.6	45.2	20.7	11.5	37.4	45.2	7.9	14.9

		R.VLNDDGAYEEPWPAHK.E	3	128.8	134.0	75.9	80.0	90.6	105.1	55.3	68.0
		R.VVVPEAYFQFPVAK.Y	2	609.4	649.6	559.9	568.7	549.4	545.0	508.7	501.4
		R.VVVPEAYFQFPVAK.Y	3	38.4	38.9	22.6	20.5	19.6	26.8	48.9	34.1
MSMEG _5650	MSMEG 5630	K.AYVALAEGWEPNADTAK.A	2	2.0	3.1	22.2	38.4	4.8	5.7	5.4	10.4
MSMEG _5662	MSMEG 5641	K.REFDLLAVLAEHKTAVLSR.A	4	5.2	3.7	0.9	0.6	0.2	0.2	0.7	0.7
		R.EFDLLAVLAEHK.T	2	70.6	81.3	31.8	30.1	67.7	72.9	26.3	24.8
		R.LSGFDVSTAVDGAEALR.S	2	80.5	83.0	134.0	139.5	74.6	73.6	135.1	124.7
		R.VAGLEAGADDYLVKPFVLAELVAR.V	3	50.7	49.1	207.3	192.2	61.3	68.7	288.8	294.6
		R.VLVVDDDPDVLASLER.G	2	236.7	266.5	321.1	345.5	229.2	254.9	312.2	317.3
MSMEG _5664	MSMEG 5643	R.GESIEFPLR.G	2	70.4	60.2	13.8	12.6	47.6	49.4	10.0	11.4
		R.GLIQGWQDGIPGM*R.V	2	143.6	115.8	119.6	104.9	112.8	122.1	131.0	124.8
		R.GLIQGWQDGIPGMR.V	2	98.7	115.8	96.0	104.9	90.6	97.9	113.1	105.1
		R.KLIIPPEQAYGPAGGGHR.L	3	7.5	8.2	22.4	16.2	6.7	6.1	25.7	30.4
		R.KLIIPPEQAYGPAGGGHR.L	4	12.3	0.0	0.3	0.0	8.6	10.3	0.4	0.3
MSMEG _5672	MSMEG 5650	K.KSEGQPFLYPDNSLSLVENFLR.M	3	118.5	123.6	100.6	101.0	148.3	126.6	198.5	165.8
		K.LGGDDELLDIAK.Q	2	812.2	601.5	616.1	499.1	817.3	729.0	866.8	794.1
		K.LPTIAAYAYK.K	2	70.6	54.9	57.1	45.8	133.3	135.6	113.1	118.7
		K.LYPNVDYYTGVIYR.A	2	891.1	878.3	962.1	974.5	573.8	560.4	726.9	762.0
		K.SAITYIDGDAGILR.Y	2	1101.3	1095.5	1329.6	1354.8	970.1	836.1	1479.3	1233.7
		K.SAITYIDGDAGILR.Y	3	41.1	40.0	5.0	1.0	36.6	40.3	15.7	7.0
		K.SEGQPFLYPDNSLSLVENFLR.M	2	764.8	878.1	715.6	786.7	471.5	550.7	593.4	684.3
		K.SEGQPFLYPDNSLSLVENFLR.M	3	498.9	563.1	507.1	575.9	300.1	397.7	376.3	519.3
		K.TGYTTYDGGFVNTASTK.S	2	586.8	491.9	354.7	299.5	476.4	476.7	365.9	341.8

		K.YPGGELDLEIVHATEGADGIALGSLLAK.T	3	2241.5	2336.6	1359.6	1306.0	1015.1	1099.0	1204.8	1276.0
		R.KLYPNVDYYTGVIYR.A	2	103.7	122.5	193.8	228.3	121.0	138.2	218.4	246.9
		R.KLYPNVDYYTGVIYR.A	3	48.5	42.2	81.6	86.2	68.1	72.7	130.0	139.1
		R.LPGWIAHWR.E	2	75.4	53.4	52.1	50.3	38.0	43.6	26.1	43.5
		R.M#TFGFPAEPYEVDPEIVR.A	2	333.3	374.5	233.5	265.2	141.0	140.2	106.1	118.3
		R.MTFGFPAEPYEVDPEIVR.A	2	823.0	847.1	798.4	826.5	571.1	530.9	690.9	708.9
MSMEG_5675	MSMEG5653	K.SVDPDGITFYTNYDSAK.G	2	93.7	103.3	18.5	26.6	95.1	77.7	19.4	23.2
		R.IAPEVVEFWQGR.E	2	132.4	150.2	103.5	121.0	91.0	104.0	115.2	121.4
MSMEG_5676	MSMEG5654	K.VTFADVWGLLVDGR.F	2	47.8	48.3	62.5	62.0	30.7	34.3	50.5	55.5
		R.VIASTGADAAAALSGAVGAMSGPLHGGAPAR.V	3	153.0	138.1	197.0	164.4	53.5	43.9	82.4	70.0
MSMEG_5680	MSMEG5658	K.AFGGTPVTIHLTVTDVDAK.F	2	107.4	95.0	78.7	69.6	78.9	80.3	79.7	66.0
		K.AFGGTPVTIHLTVTDVDAK.F	3	235.2	197.1	273.3	246.0	172.9	201.1	248.7	252.9
		K.AVEAGAEVVM#ELADQFWGDR.Y	2	16.4	0.0	11.7	0.0	6.8	9.5	4.5	9.1
		K.AVEAGAEVVM*ELADQFWGDR.Y	2	16.4	23.9	11.7	20.8	6.8	9.5	4.5	9.1
		R.DPFGHQWSM*GQPVR.E	3	16.0	10.1	8.9	3.1	9.2	14.2	6.4	12.0
		R.DPFGHQWSMGQPVR.E	2	43.6	45.6	24.6	27.2	65.8	50.7	19.6	13.5
		R.DPFGHQWSMGQPVR.E	3	51.6	60.7	47.8	53.3	28.8	30.0	33.0	32.4
		R.EVSPEEIAEAMK.S	2	98.7	92.4	110.9	103.2	64.0	55.6	81.2	73.2
MSMEG_5684	MSMEG5662	K.NFAGDGGLWLAVMSPAALAR.I	2	163.0	165.7	122.9	131.5	80.8	91.8	112.7	118.0
		K.NPFIDEPIVVK.T	2	268.5	206.7	245.9	203.6	209.9	190.6	256.1	270.0
		K.VRPEQLTALAAAGDLFGTSHR.Q	3	434.1	363.5	313.2	216.8	472.8	357.6	335.8	285.1
		K.VRPEQLTALAAAGDLFGTSHR.Q	4	323.3	218.4	194.7	125.0	245.8	235.0	219.4	205.7

		R.WVPDFLSLPIAVENSLK.N	2	394.8	407.0	358.1	356.8	273.9	294.3	310.5	364.9
		R.WVPDFLSLPIAVENSLK.N	3	83.2	93.7	42.2	47.9	51.6	54.8	39.7	36.6
MSMEG _5690	MSMEG 5667	K.TIGPLRPGALTVNLNR.R	3	77.7	83.1	129.5	136.5	86.2	89.8	108.4	117.8
		K.TVWAYELEPTETGTR.V	2	130.2	151.3	149.1	165.7	121.5	142.1	132.7	134.1
		R.VWSLISDLR.R	2	41.2	39.8	46.4	52.8	45.7	46.8	42.6	45.0
MSMEG _5694	MSMEG 5670	K.YPVQLSVPSAIGR.A	2	26.8	0.0	98.4	0.0	13.5	15.9	159.6	132.1
MSMEG _5696	MSMEG 5672	K.GFGFLSQEDGEDVYVR.S	2	173.0	181.3	136.9	136.9	188.7	198.1	143.4	139.4
		R.SSALPAGVEALK.S	2	5.2	5.4	72.0	82.9	8.4	11.2	39.8	51.5
MSMEG _5701	MSMEG 5677	K.IGDAALVAAVAADHR.R	3	23.6	26.0	28.8	28.9	22.0	22.3	27.9	24.8
MSMEG _5702	MSMEG 5678	R.AVLDYEVPGLADAIR.R	2	60.7	68.9	95.6	100.4	28.8	32.0	100.3	100.4
		R.TGPLIVEWLAK.R	2	22.4	22.2	14.4	14.4	11.2	11.3	13.1	11.9
		R.TLIVNLPGSPGGVK.D	2	34.0	32.3	20.4	18.8	15.3	15.4	17.6	14.6
MSMEG _5703	MSMEG 5679	R.TAVAAGTLLTRPDVVALIA SGGLPK.G	3	105.3	0.0	89.6	0.0	42.8	72.2	43.7	74.9
MSMEG _5706	MSMEG 5682	R.YPVPQPLLVDIVDTMAR.Y	2	51.2	52.9	39.0	39.1	35.2	34.6	25.3	34.3
MSMEG _5707	MSMEG 5683	K.LANSEASWEGSPGDLLVI PR.T	2	118.7	136.5	142.0	166.8	121.6	122.3	149.4	150.3
		K.LANSEASWEGSPGDLLVI PR.T	3	47.5	0.0	55.6	0.0	43.0	53.5	58.6	61.0
MSMEG _5715	MSMEG 5691	K.HYQADGAIVSPTPLQEDGI PLWIAGGGEK.V	3	649.8	675.3	837.8	836.3	335.4	328.8	719.0	659.7
		K.YAQYTNFTSEPEAFR.R	2	979.8	1032.5	874.4	857.4	442.2	483.3	645.0	620.7
		R.DLGC*EYAILYFPEAAYDR. S	2	115.2	135.7	112.4	120.7	43.1	56.7	57.6	89.6
		R.FGLFIPQGWR.L	2	121.2	119.4	72.5	56.5	52.0	52.3	29.0	34.6
		R.LDLVDIPTDR.H	2	363.4	280.9	356.6	256.5	229.2	220.4	339.4	348.2
		R.SANFNAVIGESEK.D	2	149.8	132.0	222.5	204.1	66.0	52.8	146.1	120.8
MSMEG _5720	MSMEG 5695	K.AAEATGATYEPHPAEAVV NK.M	3	7.9	6.0	4.1	2.8	5.7	5.1	2.3	2.8

		K.AFMEVLSQGTR.F	2	33.5	24.9	60.9	55.5	39.2	13.1	55.9	46.0
		K.ALEVGLVDQLVSSVDELVPAAK.A	2	210.3	206.0	96.6	90.0	188.8	264.5	86.3	107.5
		K.ALEVGLVDQLVSSVDELVPAAK.A	3	94.9	121.8	44.3	53.0	91.9	118.2	30.8	49.3
		K.ANPETVAVQPWDR.K	2	88.7	60.3	14.2	6.3	93.7	46.7	20.9	8.3
		K.GVVIGLPEVTLGLLPGGGGVAR.T	2	215.6	211.5	97.1	89.5	286.5	286.5	117.7	109.9
		K.GVVIGLPEVTLGLLPGGGGVAR.T	3	32.0	41.3	2.0	7.6	53.1	47.9	9.8	9.1
		K.MPGGTPSSPALASILPSFPALLR.K	3	60.7	86.2	46.4	57.1	72.4	106.1	82.1	67.0
		K.MPLVEIIK.G	2	60.5	51.7	32.5	26.1	114.3	63.2	36.6	19.0
		K.RQEDFIGIHFFSPVDK.M	3	46.7	47.6	18.8	15.8	57.8	71.6	11.6	17.4
		K.VDIPLQDMIDR.M	2	133.6	122.5	50.1	52.0	171.1	125.2	55.5	41.4
		K.VGPENAAESFAEVEFIK.A	2	323.5	295.9	120.9	117.0	457.1	365.3	159.8	132.2
		K.VGPENAAESFAEVEFIK.A	3	19.9	24.4	1.6	2.1	30.5	29.1	3.3	2.2
		R.AGFEVVLK.D	2	7.5	7.1	1.1	0.0	16.8	11.6	0.8	0.3
		R.KAAEATGATYEPHPAEAVVNK.M	3	3.2	3.0	0.8	0.6	3.6	3.7	0.9	2.2
		R.LAAEKDSITGVVITSAK.K	2	9.4	6.8	1.1	1.2	39.9	36.5	3.5	6.4
		R.LAAEKDSITGVVITSAK.K	3	26.3	27.5	6.9	5.4	74.3	65.8	10.5	11.2
		R.MLFAEALETQK.C	2	111.9	103.1	45.5	48.3	190.6	156.6	76.4	61.9
		R.VFDYTLAIGK.T	2	170.2	168.6	62.2	52.0	229.6	199.6	62.7	57.9
		R.YFVSLVTGQTAK.N	2	301.6	261.0	168.7	153.3	419.6	273.8	205.0	143.8
MSMEG_5721	MSMEG5696	R.GGALNEIKPVNLVVGLIDELR.V	3	232.1	246.4	120.8	128.4	230.9	206.4	105.8	105.7
		R.IGFVPQGIGADLIATIEGFSR.E	3	17.4	21.8	4.5	4.5	21.2	24.9	5.5	6.8
		R.TATLVANLPETTGGVQLNR.F	2	184.5	205.8	65.3	85.2	182.9	191.5	64.3	84.8
MSMEG_5725	MSMEG5700	K.ALILNSPHNPTGAVASDAELR.A	3	316.8	324.3	218.1	254.7	198.7	189.8	276.2	317.3
		R.GFAIDVDALR.A	2	14.2	14.7	1.9	2.8	0.8	6.4	0.0	1.6

MSMEG_5727	MSMEG 5702	R.FLVDVADVVSEVR.L	2	51.6	48.0	98.6	98.2	36.1	36.4	89.7	73.2
MSMEG_5732	MSMEG 5707	R.VTAMVPVVLSDDAETAR.R	2	45.9	53.2	59.3	76.5	21.2	29.1	47.9	59.8
MSMEG_5733	MSMEG 5708	K.LGAPLHILHALPTLGR.N	3	54.7	53.3	3.6	2.4	18.3	17.7	0.9	1.5
		K.LGAPLHILHALPTLGR.N	4	70.4	70.1	0.4	0.5	35.7	31.2	1.2	0.5
MSMEG_5734	MSMEG 5709	R.RPGGLTLPAENVDLLHR.G	3	91.4	74.7	77.3	47.4	54.6	43.9	32.7	32.4
		R.STLIDVDGVAQAAPAPR.F	2	272.3	260.1	166.7	201.7	151.3	155.7	117.0	167.9
MSMEG_5739	MSMEG 5713	R.FDTLTYAQVWSR.V	2	30.2	24.9	20.9	19.3	17.6	12.9	13.6	12.7
		R.FLTLQWLER.L	2	16.6	14.2	28.1	23.1	1.7	2.0	20.8	15.9
		R.LAPILAEVEPR.I	2	27.6	25.7	0.3	0.6	0.3	0.1	0.2	0.0
		R.RFETALTALPEKR.R	3	4.3	4.9	0.1	0.3	0.0	0.0	0.0	0.0
		R.RPSFTTVHGADATEIR.A	3	15.4	12.5	0.2	0.2	1.7	6.2	0.2	0.2
		R.VVAGDIGDPNLGLTPEIWHR.L	3	60.9	59.0	3.0	3.7	1.3	3.8	4.2	5.8
MSMEG_5773	MSMEG 5743	K.NYYALGGQDWDPEQSK.L	2	206.6	219.8	2.4	4.5	149.7	144.5	1.9	1.8
MSMEG_5776	MSMEG 5747	R.ATQSLLQADLVLAEQVISDHEK.M	3	145.2	135.8	192.5	177.1	118.2	100.9	192.8	154.0
		R.TVVSAIQIIADVDR.M	2	50.7	51.4	60.6	71.0	28.2	26.6	45.3	60.1
		R.VIFQVTGNYPDGDKVTTQQ	2	99.8	139.1	117.7	155.7	49.3	70.1	137.1	184.7
MSMEG_5784	MSMEG 5755	K.LGPEYEALIGTVR.N	2	56.2	62.7	187.1	200.9	80.5	83.9	208.3	219.3
		R.AQLLQEVWGYDFFGGTR.T	2	20.8	23.3	56.0	64.9	17.6	21.7	35.9	51.5
MSMEG_5789	MSMEG 5759	K.ILAPAHLPQEQSQRPGHIPGAINVPWSK.A	4	2602.6	2734.6	1393.7	1448.8	2671.8	2822.8	1472.4	1501.6
		K.LYAEAGLDGEK.E	2	24.5	19.4	8.3	5.9	17.3	11.3	2.4	2.0
		K.LYAEAGLDGEKETIAYC#R.I	2	160.3	148.0	32.2	38.1	174.2	157.6	37.0	40.4
		K.LYAEAGLDGEKETIAYC#R.I	3	827.7	852.1	328.2	364.2	882.5	919.7	290.1	355.3

		K.LYAEAGLDGEKETIAYC*R.I	2	159.6	147.1	32.1	37.8	172.2	156.6	36.7	40.2
		K.LYAEAGLDGEKETIAYC*R.I	3	827.7	852.1	328.2	364.2	882.5	919.7	290.1	355.3
		K.TVFVEVDEDTSAYDTGHIEGAVK.L	3	335.3	371.7	59.4	69.5	300.9	302.4	24.3	34.8
		R.AFRDEVIAAIGTK.N	2	889.3	784.6	508.0	449.6	947.3	909.3	512.1	501.8
		R.AFRDEVIAAIGTK.N	3	555.0	474.9	153.2	129.7	659.3	597.8	169.2	152.7
		R.DEVIAAIGTK.N	2	22.8	14.2	5.7	3.6	8.9	3.8	1.0	1.9
		R.DFVDAQQFSK.L	2	68.5	57.6	36.6	33.2	46.7	53.7	34.5	38.1
		R.PGHIPGAINVPWSK.A	3	202.3	188.3	30.8	29.1	133.7	114.0	4.0	5.1
		R.SDVLVSTDWAESNLK.A	2	2216.4	2320.6	1018.3	1095.3	1955.1	1670.6	881.8	772.3
		R.SSHTWFVLQELLGHK.N	2	40.6	46.4	83.5	84.4	47.9	43.1	69.9	66.1
		R.SSHTWFVLQELLGHK.N	3	739.0	627.7	273.9	239.0	568.3	569.3	214.6	220.4
		R.SSHTWFVLQELLGHK.N	4	232.2	200.8	71.6	55.2	233.6	194.6	69.6	59.0
MSMEG_5790	MSMEG 5760	R.ALSSAGNGNVTVAPTGAGIHEVDVK.V	3	1529.1	1770.0	290.2	334.9	1246.5	1308.1	354.2	347.6
		R.FFAAPGTWTVR.A	2	463.1	471.3	53.4	54.5	401.7	395.0	73.1	66.9
		R.VVDGSGQAVGGAFVR.L	2	112.8	107.0	10.9	10.3	94.1	89.1	12.6	12.0
MSMEG_5792	MSMEG 5762	R.NIPVFDDLPLPADTANLR.D	2	75.0	74.2	80.8	100.6	66.0	74.3	78.9	89.5
		R.PLNQSSWELVTDALAR.S	2	49.2	52.7	60.6	57.5	38.5	39.7	33.8	29.2
MSMEG_5795	MSMEG 5764	R.EGGGPPTAFATIGALADR.V	2	55.1	54.9	50.6	44.4	49.6	49.5	42.6	46.2
		R.GDGVFETLLVR.D	2	15.0	10.8	1.9	0.9	16.8	19.4	1.8	1.1
MSMEG_5798	MSMEG 5766	R.GGLGGFAGLFALK.R	2	32.5	34.5	21.1	20.4	27.4	22.6	25.3	18.5
		R.TLGEELLEPTLIYAK.D	2	118.6	106.7	580.5	527.8	118.8	99.1	650.4	590.8
MSMEG_5804	MSMEG 5771	R.HLQQHLPVRMSAKHWR.R	2	308.8	303.3	4.5	9.5	336.9	326.2	1.1	4.0
MSMEG_5824	MSMEG 5789	K.KLPSVQVGDPFMEK.V	2	52.2	51.4	149.5	107.5	30.6	37.5	176.3	77.6
		K.KLPSVQVGDPFMEK.V	3	50.8	37.7	17.5	11.8	32.7	29.3	16.4	15.0
		K.LLAEVLTAASR.D	2	352.3	326.5	194.9	168.7	317.1	266.4	232.6	190.6
		K.LPRPTTGDELKETLLK.L	3	85.3	87.2	32.8	33.4	96.5	129.7	61.0	71.1

		K.LPSVQVGDPFMEK.V	2	124.3	133.6	142.0	140.7	98.7	94.6	165.5	171.8
		K.VESHNHPSYVEPYQGAATGVGGIVR.D	3	305.0	305.6	195.2	200.5	186.7	263.9	154.2	213.6
		K.VESHNHPSYVEPYQGAATGVGGIVR.D	4	337.9	346.3	196.2	201.9	222.5	272.8	169.1	193.9
		R.AANMTPAEVLSSESQER.M	2	273.1	279.0	156.0	147.6	186.2	208.9	124.2	176.7
		R.AANMTPAEVLSSESQER.M	3	47.7	47.3	6.4	16.4	37.4	30.8	3.0	11.7
		R.DGLISAAHDLAEGGLIQAVVESALAGETGC#R.V	3	71.5	94.2	39.6	49.5	33.3	77.4	27.6	61.6
		R.GIAVSTDASGR.Y	2	0.7	0.3	0.3	0.1	0.2	0.4	0.1	0.2
		R.TVAHEGPVYERPVERPEC#QDALIADTSAK.L	4	331.2	353.5	353.7	378.7	217.7	354.0	264.7	436.4
		R.VIIPEGADPFVFLFSESSGR.V	2	342.7	419.8	229.1	275.1	223.3	331.8	210.4	307.7
		R.VIIPEGADPFVFLFSESSGR.V	3	168.3	213.6	69.0	110.7	195.2	174.9	68.2	107.9
		R.YTQLDPYTGAQLALAEAYR.N	2	306.4	288.3	166.1	160.9	268.4	244.9	201.0	163.4
		R.YTQLDPYTGAQLALAEAYR.N	3	72.6	103.6	44.5	63.9	69.4	51.1	52.0	39.6
MSMEG_5830	MSMEG 5795	R.EIPDVIAQALSELR.L	2	112.0	114.4	188.5	186.7	156.6	140.3	221.0	206.8
		R.EIPDVIAQALSELR.L	3	12.2	9.9	7.8	7.2	15.9	17.2	6.1	8.4
		R.LAGVDGPYSVLLSAETYTK.V	2	172.5	172.7	247.9	244.1	225.8	225.3	300.1	295.3
		R.VVDVSGPNGPTTASVSTGHLLDVSPPGDGVIAHLR.D	4	295.8	321.9	449.8	440.3	352.2	365.3	435.6	453.2
MSMEG_5831	MSMEG 5796	K.FAPVMGSVVEAANK.G	2	356.0	392.8	220.3	254.6	292.8	269.5	259.9	260.0
		R.VGVITFPGTLDDIDAAR.A	2	99.2	106.1	41.8	47.2	86.8	101.7	48.5	52.5
		R.YETGADLLIPLK.S	2	235.4	232.8	185.8	185.2	168.9	163.1	179.7	160.1
		R.YVASESVLDELEGEDR.V	2	345.4	369.0	177.3	171.7	245.2	259.2	160.7	168.7

MSMEG _5832	MSMEG 5797	K.AEILDPQGQAIVGALGR.L	2	136.9	179.8	233.6	303.6	94.0	107.7	228.0	250.6
MSMEG _5834	MSMEG 5800	R.AAVPIHQGIIAPDAR.G	3	21.5	16.1	22.5	17.7	9.9	14.6	16.2	19.4
MSMEG _5835	MSMEG 5801	R.VPDQLISGVPAHVDGR.M	3	16.3	13.0	5.3	7.8	54.9	53.1	20.5	25.1
MSMEG _5837	MSMEG 5803	K.FLLAADGTVVNR.F	2	203.1	230.1	208.1	238.1	116.9	126.5	163.0	163.1
		R.HPLYAALTETPDAGGEAG DIQWNFEK.F	3	236.0	269.6	130.6	143.9	126.8	140.1	98.3	105.0
MSMEG _5839	MSMEG 5806	K.KNTFTDFIAVAR.H	3	5.0	3.2	0.0	0.0	5.8	3.9	1.5	0.9
		K.NTFTDFIAVAR.H	2	33.4	31.6	17.3	17.3	42.3	30.4	30.4	25.9
		R.DLVEYSVEHAVVGGEDR. F	2	40.4	48.6	14.0	15.3	35.2	51.0	8.5	21.5
		R.FLILHNDGAENFTLVDAPV SDPNDFR.T	3	146.9	151.0	131.5	147.9	116.4	137.6	93.7	142.0
		R.YGDATDPQAEFTSVWGR. R	2	205.0	206.7	95.4	101.1	208.0	190.4	100.1	108.4
MSMEG _5841	MSMEG 5807	-.MRPALSDYQHLASGK.V	3	9.4	9.9	8.2	4.4	6.4	9.8	6.0	6.2
		K.VIELVGAELANQLR.D	2	121.9	137.0	103.0	101.1	115.6	129.1	87.8	86.5
		K.VIELVGAELANQLR.D	3	16.5	16.6	7.6	9.4	13.6	14.3	2.9	3.2
		R.ALVVRRLDM#LPVECVAR. G	2	0.1	0.0	126.4	132.0	0.0	0.2	163.1	158.1
		R.DGTVVLADEVFTPDSSR. Y	2	84.4	97.0	60.6	84.3	78.9	83.3	39.8	51.6
		R.IDDEHLLFVASDR.I	3	54.5	55.3	43.5	39.8	43.1	39.8	34.2	38.8
		R.ISAYDYILDSQIPDK.G	2	156.2	163.0	213.4	229.2	114.1	119.3	147.2	155.8
MSMEG _5847	MSMEG 5813	K.ARIEEFNALAGHEHVHK.G	2	12.6	10.6	202.8	188.9	5.8	13.1	195.1	198.5
		R.DLTENVEQLQIR.Q	2	202.1	189.3	626.8	672.1	193.1	205.7	981.4	819.5
		R.FASAAEETLVALTR.L	2	263.6	223.9	77.3	79.6	142.5	125.3	127.6	99.5
		R.VLENVDLESIAAR.E	2	171.4	156.8	305.1	305.9	203.2	190.0	319.5	462.9
MSMEG _5852	MSMEG 5818	R.FGDPETQAVLALLESPLG QLLR.A	2	11.3	16.9	6.1	13.0	1.6	3.8	1.2	4.3

		R.FGDPETQAVLALLESPLGQLLR.A	3	54.5	57.0	23.1	29.9	0.0	30.7	0.0	19.0
		R.FGPPAGQAAWVVK.D	2	303.3	176.2	261.7	127.8	213.5	226.4	276.6	274.2
		R.IGADLVVIGPEVPLVLGVADAVR.E	3	79.1	69.2	76.0	82.0	43.6	54.0	75.7	85.1
		R.TATSEVVDNPGHLDAALDR.F	2	172.5	146.7	113.3	77.9	129.7	151.4	97.8	107.6
		R.TATSEVVDNPGHLDAALDR.F	3	424.6	425.2	295.3	254.2	411.4	396.7	430.2	429.0
		R.VGDPIHGADADGVLHAGTAR.R	3	55.2	46.3	31.0	33.4	37.2	44.3	43.4	42.4
		R.VGDPIHGADADGVLHAGTAR.R	4	107.1	83.3	62.4	52.0	81.9	82.8	88.2	83.4
		R.VLSVVGTGADLSAAR.E	2	230.2	145.5	280.0	276.2	214.1	182.8	309.2	309.6
MSMEG_5871	MSMEG5835	R.AELDNWQDIEPAVFAR.V	2	287.1	311.4	276.6	288.3	332.3	351.6	315.7	290.4
		R.AELDNWQDIEPAVFAR.V	3	17.4	8.7	11.3	6.7	20.3	22.2	11.5	13.5
		R.NLSDFGFANVDR.N	2	133.8	134.2	115.0	117.2	176.8	141.2	144.7	110.0
		R.NLSDFGFANVDRNPSPESLDEAQAK.I	3	47.1	56.9	32.3	36.3	122.8	135.8	99.4	102.5
		R.SGLIIAGLEVPHLHVHVFPAR.N	3	26.7	37.4	49.2	51.3	28.4	30.5	75.8	70.5
		R.SGLIIAGLEVPHLHVHVFPAR.N	4	45.9	44.9	27.0	28.9	52.1	51.7	33.5	32.3
		R.VMEVSQLIGK.A	2	76.9	70.3	92.8	89.8	71.6	75.6	80.0	78.7
MSMEG_5872	MSMEG5837	K.AGEPVSLSPTEFTLLR.Y	2	389.0	377.4	164.1	168.7	171.4	167.5	133.4	143.6
		R.EVRPDAVILDVMMPGMDGFGLLR.R	3	16.6	17.2	2.4	3.2	2.6	4.2	1.9	2.4
MSMEG_5873	MSMEG5838	R.LLAEGGLLDTLTDPDGPLLK.L	2	39.6	34.0	34.0	36.1	22.4	28.5	29.1	34.3
MSMEG_5884	MSMEG5848	K.VAALAEQGATAAESVR.D	2	46.4	36.6	47.4	38.8	25.0	25.6	34.1	37.7
		R.TFVVGDEPSAASLVK.I	2	190.5	181.0	170.6	180.9	159.1	158.1	135.0	137.6
		R.VPLPVGSLLR.D	2	21.4	22.3	28.5	28.0	6.7	7.1	7.9	8.0

MSMEG_5892	MSMEG 5854	K.VGAFPISIDSAELDGK.A	2	60.2	69.4	77.4	98.2	78.3	80.0	129.9	123.1
		R.SPGGLVTALEPLLR.K	2	30.1	28.9	61.3	54.0	25.4	22.4	27.3	33.6
MSMEG_5893	MSMEG 5856	R.VFGYDYQTVER.S	2	68.6	82.5	23.4	21.2	70.3	73.4	16.6	15.5
MSMEG_5925	MSMEG 5884	K.GFLQDVEIWK.H	2	35.5	31.6	4.7	2.6	20.5	32.5	5.0	5.2
MSMEG_5935	MSMEG 5893	R.NAGLGPTVIVSPLLALMR.N	2	21.1	21.4	29.4	30.1	23.9	28.2	28.5	35.1
MSMEG_5937	MSMEG 5895	K.IAFLMLPGVGTK.E	2	34.3	31.2	13.2	13.1	21.8	20.2	6.0	6.7
		R.FGAGAGNAPVEALIGVFDK.I	2	366.1	329.3	246.7	211.9	387.0	354.8	195.9	182.2
MSMEG_5939	MSMEG 5896	K.AIIVLNPAEPPMIMR.D	2	62.0	67.0	60.3	63.9	85.5	87.1	52.3	62.0
		K.LGLETSAEGVDWLLAQSEKPDLVFEATSAYVHR.D	4	44.7	52.6	26.2	29.1	22.3	34.1	9.7	14.3
		R.AIDLTPAAVGPGVVPPANLR.D	2	72.2	68.4	21.2	25.5	96.4	106.0	34.4	43.5
MSMEG_5940	MSMEG 5897	R.AIDAPAGSDFVADFAGLGSVHLR.F	3	39.4	37.4	45.1	36.4	85.8	67.8	66.0	40.4
		R.SDAVLGNPVTAVAWLAR.K	2	119.1	128.8	250.1	276.1	214.9	198.7	278.1	300.2
MSMEG_5943	MSMEG 5899	K.AAVIVSETTVTDPSGK.V	2	30.7	25.0	21.0	11.2	39.1	34.5	7.4	21.3
		K.AIVDNLLDGDVSR.V	2	63.6	66.0	139.5	129.6	112.8	109.9	178.1	127.2
		K.VLHASEAVSVPGPIPTSGTAK.S	3	125.8	153.2	219.5	248.3	196.2	246.7	189.0	233.1
MSMEG_6008	MSMEG 5965	K.LFATMLNTLER.T	2	13.1	20.8	43.2	50.9	11.4	13.3	13.0	22.5
		R.NAWLAAGYPEEVPGVTVDR.Q	2	110.6	140.9	104.3	93.1	85.1	113.4	162.0	164.0
MSMEG_6024	MSMEG 5983	K.GIMHGHGGVLIEHLK.A	3	18.4	23.1	10.5	12.7	27.0	31.2	11.2	18.7
		R.HVPDEIIAAPGVPHTR.T	3	38.3	28.2	27.7	19.6	19.0	16.0	15.7	16.1
		R.LGQLEPTVLVTADGYHFAGK.T	3	51.6	55.1	33.5	37.5	25.4	32.8	16.0	35.5

MSMEG _6073	MSMEG 6035	R.VTESVQIAADR.G	2	19.1	16.1	13.9	12.1	9.8	9.0	7.6	8.2
MSMEG _6075	MSMEG 6037	R.GEGLAAIATALVAAEPVG DAK	2	5.8	0.0	21.4	0.0	5.4	4.4	12.0	15.9
MSMEG _6076	MSMEG 6038	K.ITTPLDLVLAEAVLAR.G	2	32.8	32.2	80.8	75.2	28.5	28.0	89.7	112.5
		K.ITTPLDLVLAEAVLAR.G	3	8.0	7.0	13.8	12.3	6.2	6.1	11.7	12.5
MSMEG _6077	MSMEG 6039	K.AETILDEVLAAAS	2	30.6	26.1	1.9	1.7	18.5	20.7	0.5	0.2
MSMEG _6082	MSMEG 6044	K.ATLSALDEGEVPSGFVR.D	2	42.9	54.6	235.7	263.5	85.5	85.8	252.1	225.6
		K.VPLIVVLGHDSC*GAVK.A	3	149.4	147.2	30.5	34.0	174.0	166.1	26.0	35.4
		R.STAIAQGLAAGTQAIVGTT YHLADGR.V	3	71.0	80.1	232.3	258.5	123.5	126.0	257.8	238.8
		R.TAGHVIDNAVLGSIEYAVT VLK.V	3	28.4	25.4	113.9	103.1	36.0	39.5	78.8	74.7
MSMEG _6086	MSMEG 6049	K.INAIEVPPNAGPELEK.R	2	216.7	237.9	238.7	258.7	167.5	166.0	282.7	252.0
		K.INAIEVPPNAGPELEKR.F	3	38.2	44.9	34.3	40.5	61.2	62.3	90.6	87.0
		R.AHAVDNQPGFLGFQLLR. P	3	33.1	32.5	22.5	20.8	19.5	18.0	15.4	19.6
		R.AHAVDNQPGFLGFQLLRP VK.G	3	212.7	282.4	159.9	209.1	243.6	214.9	260.2	247.6
MSMEG _6091	MSMEG 6054	K.AHQEIYNSLLQVLEDGR.L	2	54.4	67.2	212.9	232.5	58.9	72.6	246.1	335.7
		K.AHQEIYNSLLQVLEDGR.L	3	105.3	116.2	142.7	164.3	112.6	128.7	172.2	202.3
		K.AVGLGFSQGGSENNYER. M	2	146.9	101.0	151.7	103.0	133.4	118.3	132.2	111.8
		K.HFRPEFLNR.I	3	122.1	45.8	144.8	47.6	99.2	31.9	125.0	35.0
		K.NTVLIFTSNLGTSDISK.A	2	174.4	167.6	277.7	262.0	223.3	186.1	368.5	286.7
		K.RAETAEPDLAGAGAAGAP TAGTE	2	61.3	37.0	98.2	62.7	54.3	33.3	93.4	58.8
		K.RPSGSFIFAGPSGVGK.T	2	196.1	179.8	251.3	230.4	178.7	161.3	275.2	264.4

		K.RPSGSFIFAGPSGVGK.T	3	158.9	119.3	164.7	119.0	184.8	134.8	203.7	141.6
		K.SLESLGISLEGVR.S	2	167.5	165.1	261.4	265.2	216.9	177.8	359.6	351.3
		K.TAVVEGLAQAIVHGEVPE TLK.D	3	226.8	202.2	453.9	409.4	195.9	154.0	552.9	495.9
		K.TAVVEGLAQAIVHGEVPE TLKDK.Q	3	89.5	96.1	123.9	122.5	184.5	161.1	236.1	220.0
		K.TAVVEGLAQAIVHGEVPE TLKDK.Q	4	40.5	37.9	52.7	42.1	90.9	82.1	133.0	116.9
		R.EALQLGHNYIGTEHILLGLI R.E	3	67.6	68.5	120.9	136.7	97.6	86.1	157.1	148.2
		R.GELQTIGATTLDEYR.K	2	126.8	130.1	221.0	184.6	96.4	119.8	245.6	196.8
		R.GELQTIGATTLDEYRK.Y	2	240.1	183.7	240.5	227.1	416.1	265.6	390.3	359.5
		R.GELQTIGATTLDEYRK.Y	3	134.1	121.7	196.2	167.5	187.4	169.9	299.4	266.6
		R.GGESGNPSTSLVLDQFG R.N	2	308.0	288.5	406.7	378.4	286.4	296.5	451.9	436.1
		R.LFGAPPGYVGYEEGGQL TEK.V	2	191.4	182.2	237.6	235.6	212.2	214.8	346.0	311.2
		R.M*LNHNYIGTEHILLGLIHE GEGVAAK.S	4	49.4	56.3	67.2	96.4	24.9	21.2	49.4	80.3
		R.MLNHNYIGTEHILLGLIHE GEGVAAK.S	4	49.4	86.3	67.2	96.4	26.4	133.5	49.4	153.4
		R.RKPFSVVLFDEIEK.A	3	0.6	1.5	15.0	14.8	9.3	18.7	20.8	31.2
		R.RVVVLAQEEAR.M	3	3.0	1.7	2.2	1.4	3.3	4.1	2.7	4.3
		R.SQVEEIIGQGQQAPSGHI PFTPR.A	3	633.3	664.3	1064.5	1050.7	603.2	714.5	1063.6	1261.0
		R.VSITDSAMVAAATLADR.Y	2	152.5	160.8	205.5	242.9	185.3	162.5	339.1	252.5
		R.VVVLAQEEAR.M	2	2.1	1.3	1.9	1.4	1.2	2.4	1.2	2.6
MSMEG _6092	MSMEG 6056	R.GRIPADVIDAFHAAT	2	34.9	42.7	25.0	31.8	53.0	52.2	46.8	54.3
		R.GRIPADVIDAFHAAT	3	63.3	56.7	12.5	11.7	106.6	102.6	38.0	35.2
		R.IPADVIDAFHAAT	2	457.0	349.3	221.7	233.2	342.3	374.5	239.3	226.0
MSMEG _6094	MSMEG 6057	R.AHPDLAADTATGQIVGVS GR.V	2	42.4	53.7	37.7	41.7	10.2	22.8	13.6	29.8
		R.AHPDLAADTATGQIVGVS GR.V	3	220.0	230.3	269.6	281.5	87.2	103.6	158.2	225.6

		R.DFPVETTPLTR.A	2	69.2	54.9	102.0	91.5	18.4	18.3	62.7	63.8
		R.ELIQEVADEAIGTR.Q	2	47.6	35.9	29.7	24.1	60.0	58.1	52.1	45.2
		R.GFLEVETPMLQTLAGGAAAR.P	2	52.9	57.6	66.9	58.3	9.9	23.0	23.8	29.7
MSMEG_6096	MSMEG6059	K.GEFLGGAIAPGVQVSSDAAAAR.S	2	75.4	75.1	41.3	49.5	103.2	93.0	42.8	57.5
MSMEG_6097	MSMEG6060	K.FSAGELNVYSAPADVAAVTR.A	2	868.3	866.9	1192.1	1122.8	2767.7	2758.3	1440.6	1443.0
		R.AVLDEVPALEVDYLQVR.D	2	121.5	137.8	195.3	185.5	120.3	117.5	194.6	173.1
MSMEG_6105	MSMEG6068	K.YPTGYGVTLFNALQDK.G	2	98.7	94.7	38.1	42.5	126.4	97.5	58.6	66.6
		R.AAEELVFREPTTGAVSDIEQATK.I	3	36.0	35.2	52.5	46.6	74.9	94.1	62.2	53.6
		R.AELEAIFGDVK.K	2	57.4	62.2	68.3	67.0	39.9	38.3	72.1	57.3
		R.DVLDILAGELLEK.E	2	184.7	188.3	24.1	28.9	287.7	253.9	32.4	29.6
		R.GYKPVDTTVALAQIR.G	2	18.7	25.6	213.7	214.7	31.8	35.0	89.8	114.9
MSMEG_6110	MSMEG6073	K.GAVMFVTDLAR.S	2	6.9	6.9	1.9	2.6	3.7	5.8	1.0	1.4
		R.DLPFIGLLDPK.V	2	14.6	15.9	25.6	25.6	17.4	18.4	19.0	18.2
		R.DNLGDDDLLLVTVLK.G	2	7.3	9.0	67.8	67.9	4.3	10.4	22.0	39.6
MSMEG_6114	MSMEG6077	K.LLC#VPAGDPR.W	2	5.6	6.3	5.0	5.9	4.6	5.7	3.4	3.3
		K.M#TDEAGGDDKLLC*VPAGDPR.W	3	40.9	49.7	39.2	48.5	54.9	56.7	44.0	51.3
		K.M*TDEAGGDDKLLC#VPAGDPR.W	3	43.1	0.0	40.8	0.0	57.6	59.8	45.4	53.3
		K.MTDEAGGDDKLLC*VPAGDPR.W	3	21.8	0.0	18.1	0.0	40.3	41.5	27.0	31.8
		R.AEAEAELQR.S	2	0.6	0.3	0.2	0.1	0.6	0.3	0.2	0.0
		R.WDHIQDVGDISQFELDAIK.H	3	302.3	289.8	307.6	277.2	267.2	304.2	278.6	292.7
MSMEG_6127	MSMEG6090	R.LVVVADGPATSR.P	2	1.0	1.2	7.0	7.5	0.8	0.9	8.8	7.5
		R.LVVVADGPATSRPLK.L	3	6.9	0.0	5.3	0.0	11.5	15.7	11.2	15.1
MSMEG_6128	MSMEG6092	R.AIDILLVEDDPGDELITR.E	2	16.4	18.1	29.7	36.5	35.6	39.4	30.4	31.4

MSMEG _6142	MSMEG 6105	R.AENLHSAENSDKFEFVK.A	3	75.8	57.5	46.4	37.5	44.8	46.8	6.3	17.0
		R.DYVFVDDVVDAFVR.A	2	90.8	62.1	44.7	35.9	57.0	52.9	24.7	25.6
		R.ELHTAIAGAVGAPDEPEFHPPR.L	3	109.1	98.4	20.5	29.3	55.3	52.2	10.0	11.9
		R.EVLGWQPQVALAEGIAK.T	2	315.4	307.9	277.2	284.8	204.3	208.6	261.9	259.2
		R.FNVGTGVETSTR.E	2	4.8	3.5	0.8	0.7	4.1	2.6	0.2	0.3
		R.LLADGHGVVGLDDLSSGR.A	2	42.9	41.3	5.6	11.2	21.9	20.7	2.0	11.3
		R.LLADGHGVVGLDDLSSGR.A	3	120.0	127.0	54.0	65.3	80.6	84.7	30.3	34.6
		R.SVDDPPFDATVNVVGTVR.L	2	158.1	175.3	9.5	10.5	92.3	113.1	7.2	4.7
		R.TLVTGAAGFIGSTLVDR.L	2	201.2	202.0	283.4	273.5	148.6	153.2	275.5	261.9
		R.TLVTGAAGFIGSTLVDR.L	3	43.0	41.3	6.6	7.3	23.6	25.4	1.4	1.4
MSMEG _6157	MSMEG 6120	K.IAGYLGSNYVVESSR.G	2	66.3	63.3	39.1	44.9	51.7	47.9	27.7	33.0
		K.NAQEAHEAIRPAGDVFQTPGQLHSALDTDEFR.L	4	139.5	148.3	115.0	127.5	156.3	184.3	125.5	147.4
		R.ANLKEDLTPDELTPELAEK.L	3	98.1	97.4	37.9	37.4	30.7	32.0	27.4	33.4
		R.FGPYVTDGETNASLR.K	2	119.2	119.9	156.7	152.1	130.0	129.4	155.0	168.0
		R.GAQLAVTSVEQKPYTR.R	3	4.5	3.7	8.4	7.0	2.7	4.0	2.0	4.3
		R.IGGSASSGEQVVFNASGR.T	2	27.8	28.7	40.0	35.7	27.7	32.4	57.9	84.5
		R.LGVNVDQNFEPLYIVSPEK.K	2	8.9	11.6	13.1	16.0	1.2	3.1	3.8	3.3
		R.LYELIWQR.T	2	17.2	17.2	90.1	86.0	26.0	23.1	87.4	82.9
		R.LYGYEVSPVLWK.K	2	93.2	94.6	176.8	194.5	144.9	102.2	180.8	192.9
		R.MVFHEITEPAIR.N	3	2.7	2.6	6.5	5.4	4.9	6.8	4.7	7.9
		R.SLATEDQIFTITLDEALK.I	2	38.0	40.3	59.9	72.0	51.8	49.7	54.8	63.7
		R.SMDLETVTLEDALK.L	2	46.7	29.5	25.1	23.5	52.0	30.1	23.4	19.2

		R.VVGVDPTTNEEITAQNGR.Y	2	5.9	5.0	16.2	14.3	3.2	4.5	7.1	9.2
MSMEG_6159	MSMEG6122	R.TLEENQKVEFEVGQSPK.G	3	10.7	15.0	7.9	10.0	33.5	38.3	31.9	35.4
MSMEG_6179	MSMEG6139	K.AANALTDLGLVAGDR.V	2	246.8	231.5	1082.1	1028.6	412.7	388.6	1239.4	1192.3
		K.ASAHGGPENMIEELR.A	2	83.4	82.8	552.1	559.2	86.0	93.1	629.5	671.5
		K.ASAHGGPENMIEELR.A	3	61.2	53.5	140.5	105.5	90.9	79.0	153.6	121.9
		K.LGHQIPASHDLSSLR.L	3	62.0	29.6	115.0	53.5	87.6	29.2	92.3	31.9
		K.LGHQIPASHDLSSLR.L	4	76.9	27.9	133.3	47.5	101.6	34.6	104.9	35.6
		K.LVITSDGQYR.R	2	2.8	1.8	4.1	1.9	3.2	5.4	1.4	3.8
		K.YGVTIYYTAPTLIR.T	2	142.0	160.0	322.5	337.9	241.5	239.2	344.6	342.7
		R.EIHIVPELPK.T	2	45.4	33.3	84.1	62.6	102.5	63.7	92.2	50.2
		R.ELGDTSTLVDPSVFEAIR.A	2	200.2	197.6	441.7	398.1	336.6	282.4	474.3	388.3
		R.FAEQGWYFAGDGAR.Y	2	264.7	354.6	277.3	292.2	237.7	378.9	256.7	295.5
		R.LLGSVGEPINPEAWR.W	2	539.7	561.5	756.6	752.7	660.0	631.8	761.5	697.7
		R.LSWQTPFTDVLDWSDAPFAK.W	2	57.2	70.8	93.8	108.5	55.4	112.2	57.2	124.7
		R.SITYAELKDEVC#K.A	2	76.7	81.5	225.9	227.1	106.6	110.1	216.6	219.9
		R.SITYAELKDEVC*K.A	2	76.7	81.5	225.6	227.1	106.6	106.9	216.6	219.2
		R.VAIHWEGEPVGDAR.S	2	60.1	40.8	134.8	79.4	105.4	63.6	90.0	53.6
		R.YDSDGHIWVLGR.I	3	131.3	131.4	60.5	50.5	92.6	94.9	56.7	46.9
MSMEG_6189	MSMEG6148	R.AGIFQGVEPTAVAALTK.Q	2	791.1	816.6	1359.0	1373.8	1027.5	855.3	1413.2	1340.0
		R.AWIADRPEIAEQLLR.V	2	207.6	201.8	386.7	365.9	180.2	184.5	341.7	345.5
		R.AWIADRPEIAEQLLR.V	3	317.3	330.2	497.9	485.4	272.6	292.1	461.2	443.0
		R.TNNNLADLIFTDVPGR.V	2	1637.6	1785.1	1135.0	1116.9	1707.9	1615.3	1191.0	1056.0
		R.TNNNLADLIFTDVPGR.V	3	49.5	46.8	39.3	34.9	37.8	41.8	30.3	34.5
		R.VTHDLTQEEIAQLVGASR.E	2	160.2	154.9	281.4	287.1	159.0	153.6	279.9	299.2
		R.VTHDLTQEEIAQLVGASR.E	3	470.7	456.9	653.2	661.3	418.1	424.5	612.8	631.0
MSMEG_6191	MSMEG6150	K.VGAEVSAEEAK.E	2	4.9	2.4	3.0	1.8	2.9	1.9	3.4	2.0

		K.VVGFVASAPGFGGQPGVVNGASELFGEIFGDAGAHAR.S	3	40.2	58.0	24.9	33.7	18.3	33.1	15.0	25.1
		K.VVGFVASAPGFGGQPGVVNGASELFGEIFGDAGAHAR.S	4	33.9	43.1	22.0	25.7	16.7	23.2	11.3	18.8
		R.VC#GLNALAAVHALVGIDSVVK.V	3	183.4	130.3	119.9	82.1	146.1	119.0	127.9	95.6
		R.VC*GLNALAAVHALVGIDSVVK.V	3	183.4	130.3	119.9	82.1	146.1	119.0	127.9	95.6
MSMEG_6195	MSMEG6153	R.GTQFVVVSAAEPDALR.E	2	150.2	144.7	192.5	208.7	151.8	162.0	206.0	226.5
MSMEG_6207	MSMEG6167	R.FAEDYLVEPLGEGR.A	2	25.1	24.7	56.0	57.3	14.9	19.5	43.8	50.7
		R.FALEPADEAFFGSAPHVFTYR.K	2	0.7	0.4	4.2	3.8	0.3	0.6	1.6	2.8
		R.FALEPADEAFFGSAPHVFTYR.K	3	20.1	20.9	50.4	54.9	26.5	22.2	68.2	57.0
		R.PAFAVPFK.A	2	8.8	7.7	18.2	14.9	8.9	10.0	18.4	17.8
		R.YSFYVDHASVPSLR.R	2	6.6	0.0	14.0	0.0	10.4	9.3	8.9	10.8
		R.YSFYVDHASVPSLR.R	3	11.8	0.0	28.9	0.0	13.2	11.3	26.5	25.8
MSMEG_6210	MSMEG6170	R.LTTPIDGSGLSVDAVADQIR.S	2	2.5	3.9	110.0	124.1	18.0	22.7	99.6	109.6
MSMEG_6213	MSMEG6172	R.FATLLQEQFGGANGELK.A	2	11.3	14.5	93.9	110.0	6.7	12.2	109.7	118.2
MSMEG_6215	MSMEG6174	K.SGGAPEVELAPEDIK.I	2	143.6	135.4	178.9	153.0	166.0	158.7	139.3	161.8
MSMEG_6229	MSMEG6190	R.ATLESIC#YQSR.D	2	12.0	8.9	11.8	4.8	31.6	42.8	2.3	9.4
		R.HQLEHEQILPR.A	3	7.6	5.7	10.7	8.9	41.3	36.5	19.8	20.8
		R.PYYNAIVWQDTR.T	2	246.9	247.1	352.0	375.8	557.8	577.5	324.1	354.9
		R.TDSIAAALDR.D	2	10.5	4.1	24.3	15.9	27.5	26.2	65.7	59.0
MSMEG_6242	MSMEG6203	R.LAQDVGIPDNFSQVR.V	2	449.0	424.8	60.3	73.4	476.7	443.2	109.3	106.8
MSMEG_6243	MSMEG6204	R.FAYEHHDDSGNWFR.S	2	7.4	6.7	10.1	12.2	24.0	10.1	11.8	11.7

MSMEG _6256	MSMEG 6217	K.ILGIPELLVSGTC#VR.V	2	206.3	166.7	249.8	217.5	149.5	165.6	189.8	215.1
		K.ILGIPELLVSGTC*VR.V	2	196.1	156.3	234.4	205.3	126.6	145.4	168.5	188.8
		K.LVDVPTPLAAAGIDDC*LVGR.I	2	143.7	144.2	174.6	185.5	150.7	133.1	214.3	205.4
		R.AVIDGVEQLVHDGSALQYPAPNK.Y	3	410.0	380.4	492.5	458.7	438.8	412.7	494.0	493.7
		R.KDPDVPLVVSEVNFDR.D	2	167.5	197.0	86.7	118.3	121.3	114.2	51.5	68.3
		R.KDPDVPLVVSEVNFDR.D	3	58.4	67.4	51.5	47.6	57.0	61.0	28.7	45.1
		R.KGAALNTIQIAELLAADL	2	123.0	114.8	265.2	254.2	134.4	120.0	267.1	258.8
MSMEG _6257	MSMEG 6218	K.AVSALHEAFGLGGDDEAVVYAGTGR	3	178.9	181.1	208.5	199.8	146.2	137.1	193.1	181.3
		K.SEIGFSQVLYDDHIGK.V	2	69.1	82.0	82.3	90.1	47.7	48.1	74.0	71.1
		K.VTVVGLPDVPGYAAK.V	2	680.3	745.1	955.7	1093.8	559.7	557.0	931.0	914.8
MSMEG _6271	MSMEG 6231	K.IATDAAELVLEEAK.K	2	231.6	234.9	168.5	173.4	202.5	217.0	160.3	170.6
		R.GTGVAAAELGYQAGADR.I	2	29.8	26.6	9.1	7.2	16.0	15.5	3.5	6.5
		R.GVDPQIDFSNIDEIR.R	2	102.5	139.7	22.5	23.0	47.4	163.9	13.0	35.0
		R.HPYGGDLVYTAFSGSHQDAINK.G	3	40.3	38.1	5.4	9.5	34.0	34.3	8.3	12.5
MSMEG _6280	MSMEG 6240	K.GSGEVVSVQIDPK.V	2	2.5	0.0	20.8	0.0	2.5	1.9	22.1	27.2
		K.VVDPDDVETLQDLIVGALSDASK.Q	2	22.0	33.8	30.0	46.6	20.3	24.9	31.6	38.2
		K.VVDPDDVETLQDLIVGALSDASK.Q	3	40.5	35.7	62.4	54.5	37.4	46.7	66.3	79.1
MSMEG _6282	MSMEG 6242	K.ATVDVAGHTVIEK.D	2	28.3	26.9	31.1	30.2	22.2	17.2	34.8	24.1
		K.DANSSLVSNWTVAPAGTGSSVNLK.T	2	116.4	149.4	110.7	132.6	72.0	77.2	92.1	97.1
		K.ILSSHYSGYQVLEGGQGAGTVATWK.L	3	549.1	602.4	530.8	572.2	343.4	376.3	462.0	467.0
		R.IQDEVLENLKK.H	3	6.4	4.5	2.4	1.2	5.0	6.3	3.0	3.6
		R.RIQDEVLENLK.K	2	19.8	25.2	1833.9	2223.9	7.6	13.6	2012.1	1921.3

		R.RIQDEVLENLKK.H	3	64.4	74.5	48.5	56.3	68.1	66.6	76.8	69.8
MSMEG _6283	MSMEG 6243	K.GLDVSGLTGVIGVDPDAR. T	2	32.0	25.0	37.6	34.8	71.0	55.3	23.2	23.2
MSMEG _6284	MSMEG 6244	K.AIAQEGLTDLAEVR.H	2	365.5	364.7	384.9	436.5	352.5	362.4	431.0	461.2
		R.GTTYLATAPGDLGLAR.A	2	229.6	220.3	241.3	262.1	175.7	173.5	248.7	263.5
		R.IITEAQDVGLEVIHEENLR. N	3	435.0	382.6	397.3	316.6	455.9	356.4	532.7	447.8
		R.SIGIEHLKPIAPPPQEALPR .W	4	111.0	109.7	53.3	66.2	103.1	103.7	85.7	84.9
MSMEG _6285	MSMEG 6245	R.LDMSIPAGEASSGVAPAE PAK.Q	2	75.4	86.7	14.6	23.1	59.9	96.1	10.6	31.4
		R.LLLPSAADTESALLQR.I	2	109.1	102.4	159.2	160.5	117.2	89.8	161.4	162.0
		R.RDPEEVALELLQNELGAR. R	3	7.7	9.2	6.4	8.3	15.9	11.0	14.7	15.1
MSMEG _6286	MSMEG 6246	K.DAGIAVTEAGSAFPYR.K	2	184.2	178.9	111.5	117.5	145.7	146.1	125.1	141.9
		K.FALVAEILEDR.L	2	31.6	31.5	16.4	14.7	32.9	30.8	26.0	24.8
		R.NYGGVQGLPELR.A	2	68.4	31.8	19.0	10.6	79.7	79.5	88.6	85.5
MSMEG _6288	MSMEG 6249	K.DTADADEIDKDAADEDEA GEGEDATER.A	3	10.5	13.8	6.9	11.1	1.8	12.4	0.1	9.5
MSMEG _6309	MSMEG 6269	K.TVLQNITEGPIVAQGR.P	2	43.8	44.4	251.4	233.3	60.0	56.1	255.6	195.2
MSMEG _6317	MSMEG 6277	K.VPFFDAGSVISTDGVDGIH FTEANNR.D	3	574.1	636.8	359.4	367.2	366.2	396.1	269.4	282.5
		R.DLGVALAEQVR.S	2	64.5	59.0	94.8	97.3	70.0	64.7	79.6	90.5
MSMEG _6329	MSMEG 6290	R.SSEQTVDDAWPAVLTWR .A	2	22.3	23.1	34.9	42.9	44.1	45.0	37.3	36.9
		R.TDSVSLPVAYVR.L	2	7.4	11.1	3.6	5.2	11.9	9.7	4.8	3.0
MSMEG _6337	MSMEG 6297	R.IDNTVLDAEDATVVR.A	2	45.8	58.6	340.7	332.4	42.3	54.3	231.1	251.7
MSMEG _6351	MSMEG 6314	R.DHTHDLDAMLAAITDR.T	2	63.3	77.5	18.0	21.5	64.3	99.7	4.4	7.2
		R.SFEIYPLQVR.T	2	241.6	208.0	79.7	86.0	223.7	171.6	95.5	79.6
		R.VGYAVADPEIVTALGK.V	2	634.5	624.8	430.7	457.2	480.6	465.4	392.0	370.9
		R.VTIGAPHENDAFLDFAQR. W	3	203.7	181.8	168.7	158.3	165.1	146.3	148.6	153.9

MSMEG _6352	MSMEG 6315	R.DGEIPDAELDAFWALLR.P	2	21.3	26.2	13.5	18.8	14.1	21.8	9.1	15.0
		R.GEVTASM*VYDGRPVHDH FK.V	3	2.8	2.6	1.5	1.2	2.9	2.3	0.8	1.0
		R.HLYFYLER.I	2	63.0	65.9	30.9	38.7	47.6	53.9	31.2	31.1
MSMEG _6353	MSMEG 6316	K.DHVAQVTLIGPGK.G	2	9.6	5.3	124.6	120.9	4.7	4.3	89.3	97.3
		K.DLAEAVTAMFEK.R	2	43.3	46.6	35.3	37.0	38.7	38.0	27.7	36.6
		K.IGLVNDVYDDAEASLAAA HATAAEIAANPPLTVAGVK.D	4	142.4	178.5	131.1	162.1	62.5	109.9	56.8	105.5
		K.LAIVADVGSLAR.L	2	105.0	108.1	94.4	94.5	105.6	105.4	80.1	90.7
		K.MQGAITAVADC#R.T	2	6.6	4.4	3.7	3.4	7.2	5.3	4.9	3.3
		R.LPLILSDGHLR.E	2	40.3	41.5	24.7	20.8	39.6	36.5	20.2	25.2
		R.LPLILSDGHLR.E	3	138.6	123.9	71.8	65.6	169.1	141.5	97.4	76.5
		R.YVAAWNSAFLPSK.D	2	187.0	210.4	198.8	229.1	216.8	216.6	232.2	245.6
MSMEG _6354	MSMEG 6317	R.GTFEPPGVGFVGQAFVD ALR.G	2	5.1	3.7	53.8	48.8	6.1	5.2	61.3	52.2
MSMEG _6363	MSMEG 6325	K.LTHEVGGLVVVDHSAAAP YR.L	3	179.5	175.4	67.3	120.4	116.8	116.4	48.5	81.7
		R.LDDEANIAPWLR.A	2	57.3	56.9	24.1	25.6	41.4	46.5	18.7	13.9
		R.LFDYLQTSLR.S	2	24.2	22.5	51.8	60.3	17.2	15.2	31.2	28.8
MSMEG _6365	MSMEG 6327	R.LTLPFTDDNVPSDAELR.I	2	115.9	124.3	108.2	123.7	123.0	126.3	137.8	142.9
MSMEG _6384	MSMEG 6346	K.AAGFDVTVPFTPGR.G	2	151.2	150.7	245.3	251.2	400.5	349.6	255.7	271.9
		K.ELIADSGLTVSQLVSTAW K.A	2	3.6	6.2	5.5	8.4	16.2	14.2	6.2	7.6
		K.NPDVINPLDEDFDYR.S	2	145.4	161.9	146.5	136.7	325.5	357.2	137.8	141.6
		R.ANLLGLSAPEMTTLVGGL R.V	2	70.0	75.1	85.2	88.0	193.8	180.4	85.6	77.2
		R.FAPLNSWPDNASLDK.A	2	645.1	622.9	242.2	255.2	757.5	745.0	259.6	242.3
		R.GDATQEETDVDSFAYLEP K.A	2	34.0	31.7	28.2	35.7	107.7	90.8	31.4	28.1
		R.SAVQNLDVDALR.A	2	188.1	189.3	253.2	250.3	311.3	188.2	528.0	289.5
		R.VDLVFASNSQLR.A	2	53.2	67.0	81.0	107.3	171.2	168.6	95.2	97.3
		R.WLDHPEELAEEFAK.A	2	53.5	61.8	26.8	29.2	57.8	65.1	24.7	27.9

		R.WLDHPEELAEEFAK.A	3	11.1	9.9	7.5	6.9	50.9	50.9	7.2	11.9
MSMEG _6385	MSMEG 6347	K.AGLDGFYLGLGEALR.E	2	109.1	107.3	132.2	120.2	92.9	87.0	115.6	93.7
		K.ELVWAPGAFR.Y	2	94.8	93.5	71.9	69.5	66.8	67.9	80.4	72.7
		R.IVLAVMPGDPGR.D	2	163.7	152.7	176.0	158.8	114.3	153.6	115.6	177.9
MSMEG _6391	MSMEG 6353	K.DVTGEDVTFDELGGADE QAK.R	2	65.6	73.4	68.5	75.8	40.4	48.0	17.7	19.8
		K.TPGDPNALYGDGVVTGR. G	2	382.7	317.5	467.4	429.4	360.0	308.3	419.7	402.5
		K.TTAELLAELR.E	2	70.7	54.5	154.5	128.8	119.6	112.1	272.8	244.7
		R.DQGYMFITGPDVIK.D	2	244.3	237.1	267.9	258.4	148.5	146.8	116.5	108.3
		R.FFNAIVEADVPK.V	2	367.5	317.3	528.4	512.2	409.3	368.5	607.8	545.3
		R.GLVPEISLIFGK.C	2	90.6	89.7	69.2	76.1	107.5	98.7	70.3	60.7
		R.GYIDAVIQPHETR.L	3	16.7	6.5	35.3	14.6	13.3	3.0	14.7	7.1
		R.IFDDGDVFEIAEQR.G	2	411.0	388.1	415.8	426.6	486.6	411.0	402.7	314.3
		R.INALLDPGSFIEIGALAK.T	2	550.9	530.6	605.3	573.8	653.1	536.6	599.5	539.8
		R.IQDAVTSLAWYAELGR.R	2	181.9	180.9	373.6	369.9	206.2	192.0	293.1	246.2
		R.VDGHPVGVIANQPMVLSG AIDNEASDK.A	3	749.8	752.5	403.0	394.8	596.5	630.8	185.0	209.9
MSMEG _6392	MSMEG 6354	K.EGSNAVPLFVFHPAGGST VVYEPLMK.R	3	83.9	72.0	158.5	141.7	93.1	79.4	139.2	113.3
		K.ESGVQIPGGIIEHQR.T	2	35.2	31.8	154.1	144.9	61.3	41.1	160.6	113.6
		K.GASHTQQM*DPLLGELAA ELQGIEPK.P	3	90.9	98.2	179.1	191.8	95.5	105.6	150.1	155.7
		K.GASHTQQMDPLLGELAAE LQGIEPK.P	3	90.9	98.2	179.1	191.8	95.5	105.6	153.3	158.7
		K.GFDSEFFALSK.M	2	52.2	45.4	95.7	83.6	72.5	62.0	105.0	79.3
		K.LPPSINYAGPNPYIDFEK.E	2	39.8	39.4	72.6	69.0	35.4	32.9	34.2	34.3
		K.PAAVVGQSLGEAAASYFA GGLSLADATR.T	3	104.3	117.9	197.0	217.1	106.8	130.3	140.8	164.8
		K.PLTTGYFSTVHEGTFIR.P	3	18.5	15.3	42.0	38.0	9.1	12.7	19.5	22.9
		K.PLTTGYFSTVHEGTFIRPG SAPIHDVDYWK.K	4	43.6	39.5	105.7	92.5	54.1	54.7	112.7	123.0

K.PLTTGYFSTVHEGTFIRPG SAPIHDVDYWK.K	5	66.2	57.8	99.7	100.2	53.2	75.2	56.6	95.2
K.RLPADVPVYGLER.V	2	9.0	8.3	26.0	22.6	17.1	14.0	27.1	21.0
K.RLPADVPVYGLER.V	3	12.8	12.3	18.5	17.0	23.3	22.7	24.0	24.3
K.SNLGHLESAAGAASLAK. M	2	32.6	27.4	95.8	82.4	59.0	36.5	114.7	74.4
K.SNLGHLESAAGAASLAK. M	3	78.7	74.6	127.4	122.9	88.2	87.5	115.7	108.8
K.SPGGIFNELPTVSEETAK. K	2	83.3	80.0	183.4	163.8	90.9	82.6	145.1	130.3
K.SPGGIFNELPTVSEETAKK .M	3	32.7	37.9	48.3	53.8	39.4	45.2	63.5	55.6
K.TAEEIAAEQSELLGGASTV AELEAK.L	3	81.0	79.6	180.8	179.0	81.6	103.5	158.6	174.1
K.TIEGLATIVR.E	2	66.7	60.0	114.9	103.0	81.0	78.3	115.4	101.3
K.VLTQEAVTEALGADVPPR. D	2	19.9	19.9	41.3	48.5	23.5	30.8	26.6	42.5
K.VLTQEAVTEALGADVPPR. D	3	29.3	32.4	64.0	60.7	29.4	39.9	39.1	54.5
R.AEGTITVEDVLGAK.T	2	105.9	81.6	200.7	167.9	107.3	88.9	158.1	124.7
R.DANLYNVEELIR.Y	2	99.7	87.7	185.9	165.9	128.3	123.1	177.3	161.9
R.DGDQILAVIAGSAVNHDG R.S	2	10.9	9.1	28.9	22.3	22.5	16.8	31.1	24.6
R.DGDQILAVIAGSAVNHDG R.S	3	31.4	27.9	63.2	53.1	44.8	46.1	64.0	57.9
R.DVDYIEAHGTGTILGDPIE ADALGR.I	3	88.4	83.4	176.2	148.3	97.8	113.7	149.4	147.5
R.DVEDIAIVGVATR.F	2	44.1	47.7	80.1	91.2	38.3	42.9	45.4	59.1
R.EQLEEGVVDGFVR.T	2	139.3	136.1	158.6	158.3	124.5	118.4	161.2	142.9
R.EVLAGDLVEPEPEPEPEA KPEK.S	3	239.5	246.8	494.3	511.7	285.3	276.4	462.0	428.9
R.FPGDLNTPDEMWEALLE GK.D	2	16.6	19.1	34.8	34.3	13.1	24.4	15.5	29.2
R.FVGLIDTVLPGEPIDQSK.E	2	159.5	142.5	346.0	306.1	205.5	186.6	363.5	300.4
R.GPSVAVDTAC#SSSLVAT HQGVQALR.A	3	41.8	39.4	149.4	133.4	39.4	31.6	172.8	136.2

		R.GPSVAVDTAC*SSSLVATHQGVQALR.A	3	41.8	39.4	141.6	132.3	39.4	31.6	170.4	136.2
		R.HPGGATVQVHAR.V	3	0.5	0.2	0.5	0.2	0.5	0.4	0.3	0.2
		R.HSVYFTQGIR.N	2	6.0	4.1	9.2	5.3	6.3	4.5	5.4	3.4
		R.HVWEFVPR.G	2	37.0	30.3	59.4	51.7	42.5	41.4	55.9	53.0
		R.LMALVEYSADEIK.T	2	276.0	261.6	159.7	144.7	125.7	142.0	125.7	138.1
		R.LPADVPVYGLER.V	2	85.0	86.1	227.4	224.7	68.3	88.2	163.5	188.8
		R.M#ALELTWEALEHAR.I	3	8.5	6.4	11.9	12.0	6.5	7.2	5.4	5.4
		R.M*ALELTWEALEHAR.I	3	2.0	1.4	2.9	2.7	6.5	6.8	5.4	5.4
		R.MALELTWEALEHAR.I	2	3.4	5.3	7.2	5.8	9.9	9.5	8.7	4.6
		R.MALELTWEALEHAR.I	3	2.0	1.4	2.9	2.7	8.5	6.8	10.7	8.3
		R.NEVFAEWINK.V	2	48.1	45.2	83.0	72.1	52.8	47.0	62.4	57.6
		R.PGSAPIHDVDYWK.K	3	11.0	12.5	19.7	21.1	5.4	7.7	7.5	11.1
		R.RDGDQILAVIAGSAVNHDGR.S	3	12.6	10.3	22.5	19.8	19.4	24.2	30.1	24.7
		R.RDGDQILAVIAGSAVNHDGR.S	4	3.4	3.2	3.1	2.2	5.4	5.7	2.6	2.8
		R.SNGLLAPNPDAQAEVLR.K	2	50.3	52.7	178.4	141.8	45.0	43.9	105.9	69.4
		R.TFNVEIPAIPYEELEK.L	2	24.2	24.2	61.3	64.8	16.2	20.9	34.0	50.0
		R.TFNVEIPAIPYEELEKLDDEGQVK.Y	3	103.9	123.1	158.8	199.5	125.3	153.9	150.9	206.2
		R.TRDVEDIAIVGVATR.F	3	7.4	5.9	9.6	10.6	14.3	14.1	15.0	14.9
		R.VEESFTLVYDAIVAR.A	2	8.1	8.3	13.0	18.9	24.6	19.5	33.7	24.1
		R.VEYDFDLPPIQLTAVR.D	2	27.0	22.9	47.0	38.2	32.6	28.0	39.0	30.6
		R.VSYFYDFR.G	2	57.4	48.4	90.3	84.0	47.3	46.8	75.5	73.0
		R.VTFATWAIVTGK.S	2	26.5	21.8	56.6	42.1	44.9	37.4	69.8	50.0
		R.YAIEHRDEVEQIAESQK.G	2	0.6	0.3	1.4	1.3	6.3	2.8	7.8	3.6
		R.YAIEHRDEVEQIAESQK.G	3	59.9	66.6	148.8	131.4	124.5	127.2	192.1	177.3
		R.YHDDAIVFEPAYATR.K	2	20.2	19.7	57.1	53.1	32.9	30.5	64.3	52.5
MSMEG_6393	MSMEG 6355	K.AIKPSYGLAEATLFVSTTPSAEEPK.I	3	793.4	737.7	1438.7	1283.5	971.3	1016.3	1470.6	1460.1
		K.AVLNGSEPISAATVR.R	2	386.1	309.8	698.0	527.5	431.4	295.5	643.4	409.1
		K.EGDTGGTISVAPNFAFDHAAAR.G	3	430.8	410.1	788.9	739.0	477.6	503.0	603.0	631.4

		K.FPDGSSIVAHVER.W	2	212.1	190.2	226.8	250.5	236.8	229.1	187.4	183.9
		K.FPDGSSIVAHVER.W	3	344.7	307.1	538.9	477.6	346.4	342.9	440.3	447.5
		K.LDIGPITDDIR.A	2	323.0	302.5	523.7	497.8	439.9	360.8	599.8	486.7
		K.RDPDDTSEQLVIVAER.A	2	200.9	189.3	148.6	163.8	169.2	228.5	106.8	224.2
		K.RDPDDTSEQLVIVAER.A	3	202.9	196.9	254.9	235.4	242.2	246.8	267.7	274.8
		R.DLTWAQFSAR.N	2	102.7	94.6	140.9	125.6	187.4	157.9	203.6	173.0
		R.DVLLTAAGAIPR.T	2	466.1	403.8	770.0	650.8	661.4	520.2	851.3	683.3
		R.DVLLTAAGAIPR.T	3	0.4	0.2	17.3	12.0	0.7	0.0	17.0	15.1
		R.FNEAFGPFGFPPK.A	2	22.6	19.3	28.3	24.1	43.0	19.6	33.6	18.4
		R.GVPKPGSPPLDLSNVK.A	2	219.2	200.2	501.1	469.9	277.7	285.9	434.1	480.6
		R.IAVPLFDPSEPGHVGR.L	2	164.8	154.0	231.3	212.7	231.6	231.3	289.8	249.9
		R.IAVPLFDPSEPGHVGR.L	3	15.2	12.4	9.8	8.2	14.9	17.4	5.4	9.0
		R.IPTGVQITHLNLATNVVQVIEALEGEEGDR.G	3	188.8	261.4	267.8	353.0	95.4	283.3	107.6	306.5
		R.KEGDTGGTISVAPNFAFDHAAAR.G	3	200.7	215.6	215.3	239.4	177.1	254.8	182.0	265.7
		R.KEGDTGGTISVAPNFAFDHAAAR.G	4	172.0	151.4	219.8	168.1	211.4	213.2	223.5	195.4
		R.LHAVLDNC#HPSAILTTTEAAEGVR.K	3	286.9	293.6	445.5	449.0	289.8	352.1	366.0	440.5
		R.LHAVLDNC#HPSAILTTTEAAEGVR.K	4	332.2	294.6	593.4	494.7	328.7	365.3	471.7	465.6
		R.LHAVLDNC*HPSAILTTTEAAEGVR.K	4	328.2	292.7	591.3	494.3	324.2	363.0	471.0	465.2
		R.NHYPQDLEYSAQEASK.A	2	29.0	11.1	34.9	7.5	20.1	15.8	12.3	9.5
		R.NHYPQDLEYSAQEASK.A	3	104.0	60.1	118.9	68.1	73.1	58.6	74.2	46.3
		R.TGDYGAFYDGDLYITGR.V	2	230.8	208.8	300.3	257.7	309.0	230.4	359.5	255.7
		R.TGYVAAFSVPANQLPDEVFENAHSGIK.R	3	414.9	434.3	612.3	626.6	651.1	648.7	764.5	785.6
		R.TNPSHAEGATDDATWVR.T	3	3.6	3.6	4.2	3.2	2.7	4.7	1.2	3.6
MSMEG_6398	MSMEG 6360	K.ADDMWGSTNDPNNAWK.A	2	158.9	169.2	51.2	56.8	260.1	322.6	37.8	51.7

		K.ANDPTENVATIANNGTR.I	2	5.1	3.7	0.9	0.5	10.5	12.6	0.4	1.9
		K.WETFLTQELPAWLAANR.D	2	260.3	276.0	107.3	106.6	340.0	367.5	69.5	76.4
		K.WETFLTQELPAWLAANR.D	3	56.0	64.2	21.6	25.5	70.6	85.9	18.5	22.7
		R.PGLPVEYLEVPSAAM*GR.D	2	185.8	207.7	18.9	33.3	134.5	239.7	28.8	37.8
		R.PGLPVEYLEVPSAAMGR.D	2	285.4	287.8	73.8	78.7	341.0	358.7	28.8	37.8
		R.VEFQSGGPGAPALYLLDGM*R.A	2	88.0	84.7	79.4	79.8	97.9	94.4	73.2	67.9
		R.VEFQSGGPGAPALYLLDGMR.A	2	411.7	426.1	158.6	170.8	885.0	864.0	171.5	170.5
		R.VEFQSGGPGAPALYLLDGMR.A	3	69.3	79.2	13.4	14.5	150.6	135.2	15.0	17.6
MSMEG_6399	MSMEG 6361	R.AVYLLDGLR.A	2	4.1	2.3	4.4	4.1	9.5	15.1	7.0	5.5
		R.ELPAWLAANR.N	2	22.1	27.1	25.2	39.9	63.8	54.4	30.7	32.9
MSMEG_6402	MSMEG 6364	R.ERPSHPDIAVNVGTPSR.L	3	5.8	3.7	3.5	2.2	4.2	4.3	2.4	2.5
		R.GEDAVLVAVQAALAGR.P	2	32.2	33.1	29.5	29.9	23.1	21.4	24.2	24.0
		R.GEDAVLVAVQAALAGR.P	3	5.4	2.5	1.3	0.8	2.5	2.6	0.4	1.0
		R.GEDAVLVAVQAALAGRPGVLTGAR.A	3	14.4	3.8	13.8	4.7	15.0	13.8	16.3	15.6
		R.LVLGVHYPTDVVTGVVVGALVGK.A	3	61.8	54.6	44.4	40.0	50.7	48.8	32.7	32.1
MSMEG_6403	MSMEG 6365	K.IAIPVSKPAIAVNLAR.G	3	37.8	37.0	15.5	11.4	58.8	61.8	30.2	35.8
		R.LSIHNQPNLGGSGGYSR.V	3	9.2	6.8	2.8	1.9	7.8	6.9	1.5	2.1
		R.SHDPETHVRPQINVATQDAR.W	3	19.5	4.5	14.9	3.0	18.6	3.7	14.5	2.7
		R.SHDPETHVRPQINVATQDAR.W	4	83.6	31.5	55.0	19.4	51.8	22.5	32.9	13.9

		R.VHSAGWYAPVPAPGR.A	3	46.9	24.3	19.4	22.5	44.0	16.3	31.8	27.9
MSMEG _6404	MSMEG 6366	R.AANPDAPVVYTGPLDR.Y	2	132.1	75.7	36.4	15.4	185.7	192.3	121.9	154.5
		R.ALIAEQASEIDTK.D	2	160.2	154.0	186.1	182.4	119.6	104.6	201.3	212.4
		R.LDTDWFDVR.D	2	38.3	39.9	8.7	7.8	19.2	27.6	4.5	12.2
		R.RPHIGGNAYSEPEPETGIE VHK.Y	3	15.2	3.4	9.7	3.1	48.9	31.2	63.4	37.9
		R.RPHIGGNAYSEPEPETGIE VHK.Y	4	60.2	39.0	57.4	42.1	131.9	127.2	200.5	184.8
		R.YFNDTYEGLPVEGYTK.W	2	132.0	150.0	69.6	94.5	95.5	100.6	78.8	87.9
MSMEG _6408	MSMEG 6371	R.AGELVGVYPEATISR.S	2	110.5	136.2	219.3	283.1	166.5	162.3	228.4	262.4
MSMEG _6409	MSMEG 6372	K.VPIFIAVGEPIAPTLPAPEL TALLHSR.M	3	71.5	73.6	834.4	873.0	56.7	81.7	1192.1	1196.5
MSMEG _6410	MSMEG 6373	K.DAVVLLPDYPVPDLR.R	2	146.6	139.4	170.9	159.1	182.1	159.2	179.8	164.4
		R.VGGYNEYLYTFFK.C	2	85.6	81.5	42.5	34.7	109.5	95.0	81.0	75.1
		R.YGFEIPAALVR.T	2	31.7	28.8	36.4	80.9	32.8	34.2	12.2	13.0
MSMEG _6412	MSMEG 6375	K.GIFPWYAPGR.D	2	27.8	28.2	27.0	24.3	33.7	32.4	16.4	23.5
		K.GIFPWYAPGRDDFLGEFP LSSESIK.L	3	39.5	39.2	31.7	39.5	45.0	59.1	36.9	56.0
		K.GSDDTPLEDVGALMVAS GHPEVVLIGIGATR.Y	3	115.7	128.0	132.3	130.8	91.6	114.7	89.8	110.3
		R.DDFLGEFPLSSESIK.L	2	170.3	163.8	172.0	172.9	166.9	169.4	141.4	155.1
		R.YTPLGESTYLKPGDEAIVR .V	2	38.3	37.3	34.2	31.5	61.3	51.2	44.5	30.8
		R.YTPLGESTYLKPGDEAIVR .V	3	305.4	291.4	354.4	361.5	420.6	395.1	433.1	401.5
MSMEG _6413	MSMEG 6376	R.GEDPALVDALLAADTAR.R	2	128.7	119.4	23.5	26.6	130.4	130.1	17.8	16.8
MSMEG _6418	MSMEG 6381	R.FVLVGLPGTPPPATGADR .T	2	81.2	85.1	66.5	68.8	116.8	110.4	57.4	59.3
		R.LDEATTWLEGLR.A	2	13.6	18.7	22.2	20.4	17.8	25.6	10.8	21.4

MSMEG _6422	MSMEG 6385	R.AGSNLFHIEDFVAR.E	2	16.5	19.9	10.7	22.5	40.1	38.1	16.8	15.3
		R.AGSNLFHIEDFVAR.E	3	12.1	11.5	1.0	1.0	55.2	56.0	2.9	1.2
		R.DALALALDQER.T	2	29.8	39.9	406.4	437.5	71.8	75.9	432.4	428.2
MSMEG _6425	MSMEG 6388	K.ADISAVPTTFDQSVVLLDVR.E	2	31.5	37.7	76.0	56.7	25.0	27.2	46.9	63.3
MSMEG _6427	MSMEG 6390	K.AFWNVVNWDDVQNR.F	2	4535.0	5364.1	2386.5	2610.6	2992.6	3672.6	1784.3	2144.9
		K.AFWNVVNWDDVQNR.F	3	400.2	441.3	219.8	223.9	292.2	293.8	172.2	177.2
		K.NLAFHLGGHINHSIWWK.N	3	332.4	272.6	231.7	211.9	247.2	223.8	158.5	176.9
		K.NLSPNGGDKPTGELAAAIDDQFGSFDK.F	3	6059.2	6722.4	2557.8	3034.8	3237.5	3942.4	1836.6	2229.5
		R.ANGDHAAIFLNEK.N	3	33.3	29.8	8.1	5.8	33.2	22.2	6.1	4.2
MSMEG _6431	MSMEG 6392	K.ELTWLANMR.D	2	18.8	19.5	5.5	6.2	19.6	18.4	6.6	7.1
		K.SEGITM#SAPYLSQLR.S	2	234.1	211.3	70.2	71.3	245.9	229.8	68.9	63.1
		K.SEGITM*SAPYLSQLR.S	2	120.1	123.6	70.2	71.3	102.4	106.5	68.9	63.1
		K.SEGITMSAPYLSQLR.S	2	120.1	123.6	91.8	92.3	102.4	106.5	73.4	76.2
		R.GPHTSAEVIAALK.S	2	27.3	20.4	17.2	14.6	30.7	21.1	19.3	12.2
		R.GPHTSAEVIAALK.S	3	71.3	56.1	41.5	37.0	72.4	63.3	44.1	36.5
		R.IKPAYFTDDEYYEK.L	2	146.5	160.7	104.8	112.5	139.4	135.6	108.1	92.3
		R.IKPAYFTDDEYYEK.L	3	241.9	274.3	173.6	201.8	200.6	239.2	148.7	170.0
		R.TNPSAATM*AALANFFR.I	2	42.6	42.9	27.8	30.7	36.0	33.8	18.2	18.6
		R.TNPSAATMAALANFFR.I	2	61.9	67.9	27.8	30.7	80.0	74.0	41.6	35.4
		R.TNPSAATMAALANFFR.I	3	6.6	5.1	1.4	1.1	7.4	8.0	2.3	2.0
		R.TVGLSPEAQQDLVQK.A	2	403.4	325.7	291.6	232.8	443.2	500.6	336.9	377.9
		R.TVGLSPEAQQDLVQK.A	3	13.2	11.4	3.8	1.9	12.2	18.5	1.6	5.0
MSMEG _6434	MSMEG 6397	R.LLAPVLVPVVYR.A	2	95.1	87.2	459.3	482.6	80.9	93.5	241.1	281.4
		R.LTDLAAAVTAAENMPSAR.R	2	284.0	280.6	56.2	55.7	242.6	251.4	30.4	49.6
MSMEG _6439	MSMEG 6402	K.IMFAGPVTTVR.C	2	265.0	270.3	221.8	219.4	247.4	196.2	260.6	191.8
		R.ATADLVDEIYPDVR.S	2	311.1	316.8	165.8	159.5	196.9	226.5	141.0	153.1

MSMEG _6440	MSMEG 6403	R.ANIGGTWDLFK.Y	2	30.7	22.2	8.4	5.8	6.7	5.3	0.7	1.8
MSMEG _6452	MSMEG 6415	K.FLADEPIELLDGEGSAVIK.R	2	363.0	326.0	355.1	315.9	371.4	335.9	429.0	390.4
		K.FLADEPIELLDGEGSAVIK.R	3	128.1	112.0	86.1	72.0	170.7	128.2	130.2	108.5
		K.LLAAGEGIAPLSSEQAAVR.L	2	757.5	722.1	691.5	677.2	728.4	669.8	745.7	727.2
		R.FAGPNLVVGNTILLK.H	2	217.1	200.9	235.4	199.2	269.9	234.9	354.0	330.2
		R.FIVNESIYDDFLAK.F	2	226.4	202.8	165.8	157.1	243.4	202.6	209.7	165.3
		R.VAAVSLTGSER.A	2	13.6	10.9	12.2	10.3	11.5	9.3	10.4	6.5
MSMEG _6454	MSMEG 6416	K.EITLSAHVR.L	2	11.0	8.0	0.4	0.1	2.6	1.4	0.5	0.2
		K.GLLTQETTTFDGK.F	2	977.8	653.1	108.1	67.9	150.8	167.2	77.0	110.9
		K.TSPQNTTWADM*LAIWK.A	2	50.6	46.8	10.4	19.7	10.1	9.7	11.9	14.4
		K.TSPQNTTWADMLAIWK.A	2	612.4	599.6	81.9	78.9	101.5	107.0	90.8	83.6
		K.TSPQNTTWADMLAIWK.A	3	19.2	20.5	0.1	0.0	0.5	0.2	0.1	0.0
		K.YADHWNFVGGTPEEFAR.K	2	248.3	268.1	5.6	6.7	36.6	41.4	9.0	12.0
		K.YADHWNFVGGTPEEFAR.K	3	489.3	539.1	55.6	64.4	70.4	84.8	58.0	77.8
		R.FDRFEEAC#EVLK.G	2	368.4	306.2	32.8	20.3	63.4	60.5	34.7	27.9
		R.FDRFEEAC#EVLK.G	3	400.8	354.0	40.0	36.9	78.8	56.9	53.9	40.2
		R.FDRFEEAC*EVLK.G	3	400.8	354.0	40.0	36.0	78.8	56.9	53.9	40.2
		R.VGVLVTGIHYR.H	2	35.8	26.3	0.8	3.2	0.3	2.1	13.6	11.7
		R.VGVLVTGIHYR.H	3	144.0	107.3	4.2	0.7	15.8	16.9	4.7	2.3
MSMEG _6457	MSMEG 6419	R.LVLPGDLNGSRYVSELR.E	2	8.9	14.5	37.5	44.9	42.9	52.0	37.4	53.1
MSMEG _6458	MSMEG 6420	K.VAVVGSGPAGLAAAQQLTR.A	2	115.3	124.9	73.6	81.6	129.0	125.8	9.2	14.2
MSMEG _6459	MSMEG 6421	K.AHADVVLISGHDGGTGASPLTSLK.H	3	56.4	55.1	29.5	24.9	78.1	73.8	18.5	18.9
		K.AITALVNLEHR.G	2	5.3	6.1	107.8	153.8	12.6	5.7	53.1	119.7
		K.IAEAEGLEVLGWR.E	2	36.5	32.9	17.9	14.4	57.4	56.9	19.6	23.4

		K.LVSENGVGTVAAGVSK.A	2	27.6	26.5	14.5	16.1	27.5	28.6	18.5	20.7
		K.VGLVVEAGDAR.E	2	2.4	2.3	0.6	0.8	3.7	3.9	0.2	0.3
		R.EGEYHLFNPDTVFK.L	2	29.2	21.4	13.5	7.9	21.8	18.8	3.6	2.5
		R.EGHRPPVPIEEVEPASEIVK.R	3	45.1	47.1	47.5	45.7	69.6	63.2	58.6	53.1
		R.ELMAQLGFR.T	2	2.6	2.8	2.7	2.0	5.8	7.2	1.8	0.9
		R.ETVYFPSLSGQTFVYK.G	2	92.1	70.3	62.0	80.9	108.3	82.8	75.2	112.8
		R.EVPTDDSSLGALAR.D	2	49.9	50.4	24.4	25.1	66.3	65.4	31.3	30.1
		R.FDEVLELLHLGGR.S	2	11.7	14.1	4.6	4.1	22.2	18.4	3.1	6.3
		R.FGVTSHYLANC#TDIQIK.M	3	17.6	17.2	10.8	9.1	18.4	24.0	8.2	12.1
		R.GAQGAEPNTGDGAGILIQVPDAFLR.A	3	16.9	18.0	8.5	8.4	19.5	25.0	7.6	14.4
		R.GILASHVDATDSAVGQR.I	3	19.1	15.7	5.5	8.9	14.1	19.4	4.4	6.6
		R.ILADWDNELK.H	2	34.7	34.6	16.6	19.4	46.2	51.7	13.9	13.8
		R.IVVQVDGQLK.T	2	16.7	14.2	1.1	0.2	20.7	20.4	0.5	0.8
		R.TGAEALGSMGTDTPIAVLSAR.P	2	34.7	39.5	273.9	213.6	47.0	40.8	297.7	243.8
		R.TVNEMVGQVGALDTTK.A	2	33.2	34.9	18.2	18.5	50.6	44.8	24.0	23.5
		R.VC#HLDTC#PVGVATQNPLLR.Q	3	60.8	58.5	14.7	19.2	83.0	89.4	11.8	15.7
MSMEG_6464	MSMEG 6424	R.LAVEVAESGGATK.T	2	1.3	1.4	4.7	3.2	3.9	2.9	5.7	4.4
MSMEG_6467	MSMEG 6427	K.LEDLDLVSQDLLIAHAGELEK.F	3	108.1	106.8	255.7	235.2	62.7	63.2	259.6	226.8
MSMEG_6471	MSMEG 6431	K.ALNEAVDEVIAVADAEGIDAGAVK.G	3	84.6	80.7	30.9	25.1	59.1	45.0	57.7	36.0
MSMEG_6477	MSMEG 6437	R.NHPGHAEAVEIVYDPAQTDYR.A	3	44.5	48.9	6.6	10.7	30.4	31.3	2.9	5.4
MSMEG_6502	MSMEG 6463	R.VIASVQVQQ	2	7.2	5.1	10.5	7.9	7.7	4.3	14.6	8.8
		R.VNSDAADELHVHSNPEHNFPIEPR.S	3	125.1	122.5	231.5	220.4	130.4	113.3	260.4	228.5

		R.VNSDAADELHVHSNPEHNFPIEPR.S	4	135.5	156.4	45.4	53.7	140.9	172.1	34.0	41.6
		R.VNSDAADELHVHSNPEHNFPIEPR.S	5	86.5	0.0	17.5	0.0	115.5	121.7	17.7	18.1
MSMEG_6506	MSMEG6467	R.LTAGVAPESTTAAVDQLR.S	2	39.3	44.9	288.7	309.1	29.9	30.7	430.3	313.7
MSMEG_6507	MSMEG6469	K.AYPLGATYDGSGTNFALFSEVAER.V	3	56.0	67.9	30.6	47.8	25.8	28.9	21.1	35.0
		K.GLSNYWGYNTIGFLAPDSK.Y	2	80.4	80.1	51.3	65.0	44.8	47.8	47.4	57.9
		K.GLTQTHPDIPDNIR.G	2	97.5	73.0	157.0	165.4	70.6	49.9	120.6	115.1
		R.ITGSADLYEQTGR.R	2	37.8	28.0	28.0	20.7	16.3	17.9	21.3	24.8
		R.LATFFELVQQDPVVSQVK.L	2	179.5	181.9	153.9	161.3	105.3	103.6	182.2	165.7
		R.LATFFELVQQDPVVSQVK.L	3	33.3	48.2	16.5	34.4	35.8	30.3	20.8	29.8
		R.NFITTLLLSQGVPMLAHGDELGR.T	3	110.8	131.6	119.0	135.7	53.5	71.8	99.2	129.4
		R.VELC#LFDDDGDGGLR.E	2	39.0	50.7	16.6	27.1	12.1	15.4	13.0	30.8
		R.VTETGLEIWR.G	2	143.7	131.0	191.2	170.8	103.9	92.4	204.4	143.2
MSMEG_6511	MSMEG6473	R.VPADQLVGEENQGWTYAK.F	2	185.1	194.2	136.8	138.7	143.6	153.9	101.3	106.3
MSMEG_6512	MSMEG6474	R.ISAGLAAEALGAMEEALR.L	2	163.8	141.1	125.0	109.0	87.0	88.4	71.0	70.4
		R.ISAGLAAEALGAMEEALR.L	3	27.1	31.6	13.8	16.8	18.3	15.1	12.3	7.1
		R.LAPEPVLHGALAPGAIIAER.G	3	200.0	177.7	199.3	177.5	170.7	160.1	170.3	176.5
		R.LTAIEHTLGSAQDQLR.T	2	99.1	104.1	182.4	184.1	76.7	87.1	134.7	137.2
		R.LTAIEHTLGSAQDQLR.T	3	144.6	147.3	408.2	359.0	134.1	111.6	437.2	413.9
MSMEG_6515	MSMEG6477	R.FFSHQPDLNYDNPAVQEAMLDVLR.F	3	50.3	72.7	58.3	96.5	19.1	42.7	33.5	94.0
MSMEG_6520	MSMEG6479	R.DLGIEPVKDV	2	10.8	11.0	12.2	10.4	4.0	8.6	11.7	14.4
		R.GVPFAALVTYR.D	2	71.5	37.6	75.6	44.6	80.3	83.6	78.8	76.0

		R.VTAAGAVVSGVIPMVDR.G	2	48.0	61.1	217.4	216.4	46.6	54.0	182.8	200.6
MSMEG_6524	MSMEG6483	K.AAFADLPVVHGPVTNPTPEQTEAATK.Y	3	103.0	109.7	374.6	390.2	178.1	167.1	350.4	406.2
MSMEG_6561	MSMEG6523	R.GDFPLNTAGGQLGFGQAGLAGGMHHVCDATR.Q	3	3.7	0.0	6.0	0.0	1.6	1.5	5.1	5.7
MSMEG_6565	MSMEG6527	R.DITDALLSALNR.R	2	71.9	65.6	44.2	44.8	57.6	52.1	77.6	53.0
		R.NAAIDNVANLLGTSR.T	2	306.3	333.0	297.3	314.7	237.7	235.0	300.9	283.1
		R.SEMASYFPAVPMVVR.A	2	179.8	177.4	164.0	160.1	176.7	179.0	137.9	151.4
		R.TSDVGASGDGSGAPAADLDEEIR.A	2	119.1	139.6	97.3	111.8	84.3	98.9	79.1	96.9
		R.TSDVGASGDGSGAPAADLDEEIR.A	3	59.0	68.1	46.8	54.8	28.5	33.3	23.9	37.5
MSMEG_6574	MSMEG6535	R.VEWVLAHPTGITEIQEGTLTVGGDR.I	3	66.4	66.7	49.4	52.2	29.2	31.0	33.9	35.4
MSMEG_6587	MSMEG6549	R.LPIEPISQASAAQR.A	2	3.7	4.4	5.9	6.5	1.8	2.7	3.2	5.0
MSMEG_6596	MSMEG6557	K.TTEHRPEILLSFGFVPK.M	3	82.5	77.6	38.5	41.8	56.3	59.6	46.8	42.9
		K.VLAIAAETGIEGR.I	2	78.5	81.1	42.8	60.0	67.4	89.8	25.5	26.8
		R.GLC#TQVTSFLGEEALR.E	2	108.1	115.7	59.2	56.9	129.0	126.3	37.7	39.0
		R.GLC*TQVTSFLGEEALR.E	2	107.5	114.9	58.3	56.9	129.0	119.8	37.7	37.8
		R.LTALSGAGFDGISFVGVPR.T	2	332.8	356.7	79.2	74.0	222.1	305.7	43.4	48.4
MSMEG_6622	MSMEG6584	R.GAHFC#LGASLARVEGR.V	2	235.3	286.3	35.2	46.3	177.9	164.1	42.8	45.9
MSMEG_6630	MSMEG6591	K.EIEATGGAIIK.G	2	6.1	5.1	8.6	7.9	8.8	5.9	11.3	8.0
		K.FYETLGFSLK.F	2	3.8	3.3	4.2	5.3	7.0	6.0	10.8	9.3
		K.M#VVLSTENLDESIK.F	2	37.5	44.0	55.2	61.3	34.3	38.3	52.1	49.2
		K.MVVLSTENLDESIK.F	2	221.2	0.0	177.7	0.0	215.0	232.4	232.3	205.6
		R.AVVYDNTGNGLVFYSPLK.R	2	29.4	35.8	59.0	64.7	28.2	26.1	50.3	42.3

MSMEG _6636	MSMEG 6597	K.AIWNVVNWQDVQVR.F	2	562.2	593.3	504.3	523.8	439.7	423.2	376.4	362.1
		K.AIWNVVNWQDVQVR.F	3	89.7	82.3	55.3	48.3	61.5	61.9	31.4	32.6
		K.NLSPHGGDKPTGDLAAAI DDQFGSFDNFR.A	3	257.4	273.5	210.8	218.8	222.7	220.1	178.6	174.0
		K.NLSPHGGDKPTGDLAAAI DDQFGSFDNFR.A	4	829.6	822.0	652.9	604.9	682.5	687.4	530.2	526.9
MSMEG _6638	MSMEG 6600	K.AVGGAVAPISR.L	2	0.1	0.1	0.0	0.0	0.6	1.0	0.0	0.0
		K.GM*LTGPVTILAWSFVR.D	2	37.2	46.5	15.3	26.9	55.2	77.9	13.1	21.4
		K.GMLTGPVTILAWSFVR.D	2	45.2	69.8	15.3	26.9	87.3	127.0	13.1	21.4
		K.GMLTGPVTILAWSFVR.D	3	0.9	4.2	0.0	0.0	4.8	11.4	0.0	0.0
		K.LQEDLGLDVLVHGEPER. N	2	98.8	89.9	27.8	22.1	240.9	194.0	17.4	14.8
		K.LQEDLGLDVLVHGEPER. N	3	303.7	284.3	78.2	70.5	695.2	497.8	95.2	63.2
		K.RPALFVASYFGELTDALPA LAR.T	3	217.5	296.9	166.3	228.4	347.7	515.8	106.6	197.9
		K.VTEVVALAR.G	2	1.9	1.7	0.0	0.0	8.8	8.8	0.1	0.0
		K.WFDTNYHYIVPELGPDTS FALHPAK.V	4	8.5	8.1	5.8	6.7	18.9	23.1	5.3	5.5
		R.ALGIPARPVVVGPITFLAL SK.A	3	325.0	322.7	93.1	90.0	1249.5	1171.4	213.8	188.0
		R.DDQPLADTAAQVALAIR.D	2	196.1	161.8	56.0	53.2	397.2	196.7	72.9	51.8
		R.DDQPLADTAAQVALAIR.D	3	40.6	37.7	0.5	1.0	65.6	48.7	0.8	0.7
		R.EAIAGEIAASNDAIAAR.K	2	638.5	593.7	243.5	220.0	1263.1	1290.5	285.4	270.4
		R.GGTYDGTDIAPLEM*TK.W	2	48.4	42.3	50.4	39.3	87.2	68.8	40.8	32.5
		R.GGTYDGTDIAPLEMTK.W	2	543.2	540.3	233.8	228.0	973.0	1021.0	233.1	224.4
		R.LGLPPLPTTTIGSYPQTSA IR.V	2	493.2	558.2	134.2	156.2	1045.4	1135.6	139.8	172.6

		R.LGLPPLPTTTIGSYPQTSA IR.V	3	300.3	414.9	120.5	138.0	589.5	734.7	251.7	128.8
		R.LWVNPDC#GLK.T	2	10.7	7.9	3.8	5.3	12.4	14.9	1.3	4.2
		R.SHMEVLDDLNAAGFGNG VGPGVYDIHSPR.V	4	273.1	319.3	123.0	141.1	460.6	548.3	117.9	121.3
		R.SWLAFGFEK.V	2	159.2	149.9	38.4	35.4	409.8	328.8	64.4	44.5
		R.TPVEAIGVDLVAGTAAAV AAVPELTTK.T	2	39.3	43.8	6.3	9.7	68.9	114.6	3.7	16.7
		R.TPVEAIGVDLVAGTAAAV AAVPELTTK.T	3	687.0	874.3	253.5	305.6	1573.6	1623.5	328.8	387.4
		R.VPTTEEMATALR.N	2	268.0	217.5	106.7	76.1	562.3	296.6	122.6	68.3
MSMEG _6733	MSMEG 6692	R.VTPATATSTEDDVDYLR.H	2	82.1	87.0	92.6	97.4	78.6	97.0	95.6	112.9
MSMEG _6740	MSMEG 6701	K.SMAGLIDLVR.S	2	10.4	10.8	1.0	0.1	0.3	1.4	0.1	0.0
		R.LEGVILDPVYEGK.S	2	183.2	212.1	311.2	385.9	156.7	167.0	301.2	305.5
		R.YPLTFGPSPIHPLER.L	3	49.9	58.3	29.9	24.6	10.6	17.4	39.6	30.7
MSMEG _6741	MSMEG 6702	R.LVDIAGQIIESR.-	2	36.9	43.1	56.3	65.9	32.2	38.9	66.3	83.8
MSMEG _6743	MSMEG 6704	K.SFYQTVFGFDVLR.E	2	40.0	39.7	35.8	38.0	53.1	49.8	36.6	31.5
MSMEG _6746	MSMEG 6707	K.AGWDMWPGPYGQLGSR. A	2	183.2	181.1	120.7	132.1	57.7	78.0	41.8	52.9
		R.ATARPTFDDDLVTDQVR. E	2	147.1	163.5	53.3	60.5	97.2	118.8	91.1	72.5
		R.ATARPTFDDDLVTDQVR. E	3	315.9	256.8	375.2	303.2	230.9	215.4	431.0	358.3
		R.AYVLASLDESLDR.M	2	180.8	165.4	246.3	200.6	152.9	130.8	301.9	266.4
		R.GITHFDLANNYGPPYGSA EENFGR.M	3	381.0	376.9	413.6	414.8	244.7	260.4	407.4	414.0
		R.IDPVTPLEETIGALDTAVR. A	2	509.3	483.6	533.8	501.2	335.4	318.0	557.1	528.3
		R.IDPVTPLEETIGALDTAVR. A	3	188.9	200.4	205.8	215.7	155.5	136.6	245.9	223.2
		R.LGTPLVIHQPSYSLLNR.W	2	75.5	93.8	55.2	86.6	30.1	33.7	50.7	71.4

		R.LGTPLVIHQPSYSLLNR.W	3	226.0	216.5	253.2	263.7	134.9	136.2	282.9	259.4
		R.PTFDDDLVTDQVR.E	2	173.8	182.3	152.1	156.5	107.5	112.5	151.6	154.1
MSMEG _6754	MSMEG 6715	R.GDLVNYAGVAGDANPLH WDENIAK.L	3	160.5	162.2	98.3	94.4	132.4	145.8	61.4	82.3
		R.LSQPAVVPAEGTEIEYSG R.I	2	230.1	240.2	99.1	136.3	163.2	176.5	80.4	94.0
MSMEG _6756	MSMEG 6717	R.TAGLLPAIAAELDDEILLAR .C	2	24.8	20.2	51.0	46.8	30.3	28.4	47.0	43.2
MSMEG _6759	MSMEG 6720	K.IQWILENVPGVR.E	2	924.9	900.1	2431.6	2345.9	3642.6	3208.6	2918.5	2753.7
		K.IQWILENVPGVR.E	3	27.2	19.5	29.7	22.7	160.1	147.0	28.9	34.2
		K.LQPSDLAALGVTNQR.E	2	2156.1	2218.6	3735.5	3781.1	7395.5	7365.3	4468.6	4460.5
		K.LQPSDLAALGVTNQR.E	3	142.2	138.2	186.7	172.2	646.0	612.5	259.0	250.4
		K.NTYGTGNFLLLNTGTTPV R.S	2	1248.6	1298.1	2063.3	2062.3	3895.4	3932.4	2192.6	2288.3
		R.DQM*HVIDSAAESESLAR. L	3	736.8	581.6	1304.8	1052.2	1553.8	1488.4	892.7	919.0
		R.DQMHVIDSAAESESLAR.L	2	682.4	635.0	1219.4	1181.9	1343.5	1475.1	774.4	845.3
		R.DQMHVIDSAAESESLAR.L	3	757.0	600.9	1327.7	1073.0	1584.9	1518.1	899.4	931.1
		R.DVAEAM*EADSGVHLDVL K.V	2	172.1	214.0	298.5	301.5	239.8	234.2	232.0	218.0
		R.DVAEAMEADSGVHLDVLK .V	2	420.5	403.5	298.5	301.5	678.9	570.0	232.0	218.0
		R.DVAEAMEADSGVHLDVLK .V	3	71.1	73.5	123.8	122.9	330.5	232.4	104.2	79.7
		R.FMIFDHAGNEVAR.H	2	251.8	214.5	750.3	654.4	586.8	568.3	1347.4	1397.0
		R.LGDDAPVYALEGSIAVTG SAVQWLR.D	3	64.7	57.5	97.9	101.3	227.9	230.3	96.9	114.8
		R.LVDDNGGVYFVPAFSGLF APYWR.S	2	70.7	85.7	107.6	143.1	103.9	222.6	40.2	111.8
		R.LVDDNGGVYFVPAFSGLF APYWR.S	3	75.4	101.6	84.3	127.6	102.3	210.3	28.7	94.1
		R.PGWVEHNPVEIWER.S	2	620.6	604.5	704.1	713.7	1505.2	1518.0	782.9	774.6
		R.PGWVEHNPVEIWER.S	3	710.7	665.9	909.2	847.7	2198.1	2413.3	881.9	1043.2

		R.SM*LPQIRPSSDPAGYGT TTSSR.P	3	121.0	97.4	293.0	259.8	425.3	483.1	327.8	354.5
		R.SMLPQIRPSSDPAGYGTT TSSR.P	3	623.9	647.1	1039.1	1108.5	1339.7	1269.3	787.9	802.2
		R.SSSAVQSALNAAK.L	2	18.7	16.3	26.4	27.8	32.8	40.2	14.9	21.4
		R.TGRPYYNAIVWQDTR.T	2	610.2	724.4	502.3	434.9	966.3	1112.2	571.2	622.8
		R.TGRPYYNAIVWQDTR.T	3	478.7	462.6	318.5	216.9	929.5	992.4	443.0	496.3
MSMEG _6761	MSMEG 6721	R.EHAVASPVPGLTVIAGGK. Y	2	41.2	54.2	31.3	42.1	129.0	144.7	25.5	37.8
		R.EHAVASPVPGLTVIAGGK. Y	3	30.5	27.1	0.2	3.9	119.6	113.3	0.0	2.0
		R.EVADLIAPVLGWSQTDIA R.E	2	26.7	29.4	7.6	9.5	52.1	48.0	1.0	6.2
		R.TAEDTGLAVTTVAHLLGR. Y	3	17.2	18.0	0.6	1.4	45.2	34.5	0.3	0.0
		R.VPLLGADGYPAAWNAR.R	2	226.5	237.4	135.6	141.1	362.6	401.7	92.9	116.5
MSMEG _6760	MSMEG 6722	R.IWQIYADPR.Q	2	78.4	76.9	22.0	17.6	36.1	43.3	17.1	19.4
MSMEG _6776	MSMEG 6737	R.WLRAAGVDTTGAYSGMK .Y	2	81.7	61.5	181.3	117.4	71.2	62.4	77.3	74.0
MSMEG _6787	MSMEG 6749	K.EWLGLEFDPQSASAAK.V	2	23.2	21.0	195.3	205.7	16.4	18.9	250.9	257.2
		K.IVIGGDNAGFNYK.E	2	13.5	0.0	49.5	0.0	10.7	13.5	55.5	37.4
MSMEG _6816	MSMEG 6779	K.FGEIC*RPDWIPEGTR.V	3	0.5	0.3	117.9	101.0	0.1	0.1	152.4	129.1
		K.LAYEYATTQPAAIR.Q	2	46.7	40.5	359.1	339.5	38.6	66.3	301.5	273.2
		K.SLFVYNSNPVSQGPAQEK .T	2	141.6	181.9	530.3	542.7	53.2	99.0	411.2	483.7
		R.AITC*LPALVGAWR.H	2	2.2	1.5	121.9	123.9	2.9	1.4	149.3	120.3
		R.DDLFTVVSEHFITDTAK.F	2	8.1	8.4	223.7	218.5	3.3	5.1	189.9	158.1
		R.DDLFTVVSEHFITDTAK.F	3	0.2	0.0	69.7	61.8	0.0	0.1	83.7	46.5
		R.EMLIDFHDWDAPALAGIT YEK.L	3	7.5	3.5	102.2	146.4	3.4	3.4	78.2	96.1
		R.GSFQGPAAYETGLIPGLV MANVGHWQGK.T	3	2.4	2.0	279.5	226.7	0.9	2.3	226.7	320.3
		R.ITWDEALAEIK.T	2	0.2	0.1	186.3	165.3	0.0	0.3	242.9	171.0

		R.VEEITGIPADDVR.K	2	0.4	0.3	222.9	167.4	0.0	0.3	183.8	84.4
MSMEG _6822	MSMEG 6786	K.LFIADDLLLQESEGK.T	2	63.4	59.3	123.0	131.5	48.5	56.1	122.6	121.8
		R.FGIPDGLSGEAVAVK.Q	2	75.4	88.5	330.1	389.7	94.7	95.9	386.5	382.0
		R.YLMAISSLDPTSAGAAR.E	2	450.8	570.4	313.5	345.6	359.3	522.9	236.0	296.0
MSMEG _6854	MSMEG 6817	R.KIDQAFIGSCANGQLDDL R.I	2	71.5	80.5	1.6	1.5	44.1	60.9	0.7	1.1
		R.KIDQAFIGSCANGQLDDL R.I	3	62.4	74.6	0.0	0.5	49.0	59.6	0.1	0.2
MSMEG _6875	MSMEG 6839	R.AAFDAAIAQPGAVEPVK.V	2	71.0	71.4	102.1	115.8	30.0	32.6	70.0	112.0
MSMEG _6894	MSMEG 6855	K.LFGSVTAADVVNAIK.K	2	660.7	842.1	475.0	620.1	619.3	655.5	465.4	453.3
		K.LFGSVTAADVVNAIK.K	3	123.9	72.6	55.8	31.2	112.6	120.4	52.2	54.5
		K.LFGSVTAADVVNAIKK.A	2	23.8	21.9	5.0	7.4	36.9	33.8	12.8	4.7
		K.LFGSVTAADVVNAIKK.A	3	46.6	42.2	27.2	22.0	81.6	78.5	45.9	41.1
		K.LILTAEVEHLGAAGDTVEV K.D	2	202.6	219.2	121.7	123.3	191.7	179.9	119.8	105.4
		K.LILTAEVEHLGAAGDTVEV K.D	3	988.5	1136.7	655.6	738.3	1000.8	1067.5	753.3	649.3
		K.TALEGLGDVTLSVNAAGD TGK.L	2	250.1	222.2	133.6	105.2	237.7	238.3	129.4	120.7
		K.TALEGLGDVTLSVNAAGD TGK.L	3	58.3	0.0	27.6	0.0	92.0	80.0	47.3	33.8
MSMEG _6896	MSMEG 6857	K.DDPWGSAPASGSFSGAD DEPPF	2	59.5	78.0	23.1	34.2	33.8	52.9	6.9	18.5
		R.EAAENVAESLTR.G	2	21.4	22.1	13.2	13.7	18.2	22.8	7.0	15.2
		R.FTPSGAAVANFTVASTPR. M	2	800.0	840.1	478.2	501.6	766.0	812.5	575.6	569.7
		R.FTPSGAAVANFTVASTPR. M	3	377.3	430.2	89.1	84.1	251.4	328.0	85.2	79.2
		R.TVVEVEVDEIGPSLR.Y	2	291.3	310.4	200.8	207.3	223.7	241.6	165.8	172.7
MSMEG _6897	MSMEG 6858	K.DGGTVDKVDIWGR.R	3	3.2	0.5	1.7	0.7	9.9	9.8	2.7	4.7
		K.HAEGIYAVIDVK.A	2	33.6	31.8	13.2	14.9	41.7	32.9	19.3	15.5
		K.HAEGIYAVIDVK.A	3	13.5	10.5	9.0	5.2	18.8	15.2	7.9	7.9

		R.TVAPSLETFLNVIR.K	2	351.7	315.9	320.6	283.9	395.5	399.5	367.5	332.6
		R.TVAPSLETFLNVIR.K	3	28.1	17.3	16.7	9.1	28.2	29.9	16.2	16.8
MSMEG _6904	MSMEG 6866	K.IAKDRGIGGPIEAASAYLM #K.S	3	3.5	6.6	6.6	3.8	2.4	1.4	8.5	4.6
		K.KFEDAGVPIVGDDIK.S	2	418.2	454.3	248.7	283.3	441.6	443.1	259.2	273.4
		K.KFEDAGVPIVGDDIK.S	3	199.2	189.7	7.1	7.8	236.0	221.7	74.2	82.9
		K.LEVWDSPNSAGVIIDAVR. A	2	381.0	381.4	253.8	267.8	446.9	480.1	177.0	272.1
		K.NVHIGPSDHVAWLDDR.K	2	161.1	175.8	24.7	19.4	126.1	130.0	14.7	32.8
		K.NVHIGPSDHVAWLDDR.K	3	143.7	122.9	3.2	4.2	116.6	125.9	0.1	3.9
		K.NVHIGPSDHVAWLDDR.K	4	84.4	57.4	0.8	0.0	66.2	65.2	0.0	0.5
		K.NVHIGPSDHVAWLDDRK. W	4	13.7	1.0	0.3	0.0	145.7	131.4	0.5	0.0
		K.TQAVTSNLSGALAGK.V	2	153.1	135.3	98.2	116.3	161.6	148.1	53.4	75.9
		K.YYADTIEVSDAEPVDVVK. V	2	725.9	766.7	122.0	165.0	993.3	994.1	163.9	207.6
		R.AFGDVPLNLEYK.L	2	434.9	400.5	125.3	111.4	503.9	491.4	162.4	193.6
		R.GIGGPIEAASAYLMK.S	2	256.4	228.0	39.5	38.3	160.9	132.8	48.7	43.4
MSMEG _6907	MSMEG 6868	R.LDDLAAALPR.I	2	80.4	65.3	104.3	87.5	66.9	73.5	118.6	96.7
		R.LILGIGSGWK.Q	2	83.2	82.9	87.4	87.8	58.0	54.4	90.8	79.7
MSMEG _6913	MSMEG 6872	K.LSDEDLATLAR.A	2	11.2	0.0	12.9	0.0	11.1	12.1	9.2	9.9
MSMEG _6917	MSMEG 6875	K.FFWPGPDGEIEVNQEFGK .I	2	162.8	145.0	133.3	112.2	133.9	128.9	146.4	135.7
		K.FFWPGPDGEIEVNQEFGK .I	3	13.6	14.7	2.7	4.8	2.6	3.8	0.8	3.4
		K.NSVSPDEIC#DNYGADTL R.V	2	14.1	15.2	26.2	26.7	7.7	7.3	20.5	20.8
		K.NSVSPDEIC*DNYGADTLR .V	2	14.1	15.2	26.2	26.7	7.7	7.3	20.5	20.8
		K.TGVFLGAYATNPADGK.Q	2	284.9	285.8	207.4	219.5	264.3	262.2	254.3	235.3
		K.VLYDLGYVSSR.E	2	163.3	169.2	134.5	145.5	109.6	119.2	137.2	138.0

		R.AAIEPLVLM*VAPLAPHLAEELWK.R	3	49.5	58.4	32.7	42.5	43.4	63.8	39.4	64.9
		R.AAIEPLVLMVAPLAPHLAEELWK.R	3	51.1	59.0	32.9	42.5	45.6	65.3	45.4	68.2
		R.DTNVMPQWAGSSWYELR.Y	2	13.5	13.3	13.1	14.9	3.1	6.2	5.1	8.8
		R.GTFNVANPVGSLAPTDGSDVPADK.M	2	36.9	40.1	34.8	37.4	20.0	23.0	34.4	34.3
		R.GTFNVANPVGSLAPTDGSDVPADK.M	3	48.1	65.4	39.9	66.4	12.1	27.1	13.3	41.0
		R.GTYVPAAEVIER.D	2	308.9	13.3	291.8	266.9	186.2	198.3	193.6	202.5
		R.ISELVEEFESGK.R	2	27.6	28.2	27.7	32.7	8.1	11.6	12.0	17.2
		R.ISELVEEFESGKR.T	3	14.6	15.4	15.3	13.5	9.1	9.3	13.4	13.8
		R.LLEDLDVLDWPEK.V	2	184.3	209.2	198.9	209.3	136.7	147.9	233.5	216.9
		R.NVLHALGFDAFGLPAEQYAVQTGTHPR.T	3	139.6	167.7	613.1	715.6	112.5	138.7	432.3	740.8
		R.NVLHALGFDAFGLPAEQYAVQTGTHPR.T	4	102.9	99.5	123.5	111.8	49.2	51.6	98.4	94.0
		R.VYEMSMGPLEASRPWATK.D	3	21.4	24.0	24.9	24.7	5.2	8.9	11.2	13.9
		R.YNADLAGQIER.A	2	4.7	4.1	5.2	4.0	1.6	1.8	2.3	3.2
		R.YWGEPFPIVYDADGR.A	2	61.0	60.0	70.2	67.1	32.0	40.0	46.3	54.4
MSMEG_6926	MSMEG6882	R.KHEPDGGVSFHHHEVVGAK.M	4	0.7	0.6	1.8	1.4	1.2	1.2	0.3	0.4
MSMEG_6929	MSMEG6886	R.SAPTLLNLLQQATAIADR.T	2	16.0	13.3	42.5	46.3	16.8	16.9	33.2	43.7
MSMEG_6933	MSMEG6889	K.ITFLTNTEITQIEGDPK.V	2	241.4	263.0	88.1	91.7	241.4	241.1	57.5	60.2
		R.AVILAMGAAAR.H	2	15.5	10.5	16.4	14.1	16.5	11.2	14.7	14.7
		R.MEDVDAVQLEGPVK.T	2	160.5	160.1	125.6	112.8	148.7	171.8	47.4	56.3
MSMEG_6934	MSMEG6890	K.M*VAPVLEEIAAEK.G	2	33.0	27.8	78.3	64.2	12.6	17.2	55.1	61.7
		K.MVAPVLEEIAAEK.G	2	38.4	35.1	78.3	64.2	29.6	31.3	55.1	61.7
		R.DFQVVSIPTM#ILFK.D	2	33.1	0.0	28.2	0.0	15.7	20.6	21.0	20.1
		R.DFQVVSIPTMILFK.D	2	42.2	40.3	49.7	47.3	35.3	33.0	52.6	49.8
MSMEG_6941	MSMEG6896	K.IVHDAVAAIDGVR.S	2	29.1	15.0	20.6	11.5	25.3	19.8	29.3	23.9

		R.EELAPMTPFER.K	2	82.6	90.7	39.5	45.6	68.7	72.8	36.3	42.6
		R.KGEVLDALQELTR.L	2	224.1	224.5	198.6	190.8	242.1	239.3	238.5	250.7
		R.KGEVLDALQELTR.L	3	62.1	57.4	18.2	14.7	61.2	54.3	18.3	18.0
		R.KIVHDAVAAIDGVR.S	2	32.6	35.9	8.9	10.2	55.7	49.9	22.5	19.5
		R.KIVHDAVAAIDGVR.S	3	86.8	82.8	22.1	24.4	99.3	113.1	37.2	37.3
MSMEG _6947	MSMEG 6901	K.YFSRPEPDTSSDDSNAVN LNR.R	3	25.6	27.9	91.4	96.6	7.7	13.6	31.0	42.3
		R.LDVPDDVLELIASSIER.N	2	6.4	10.5	16.8	27.3	0.9	5.5	2.7	8.6
